# Surface‐Decoupled Altitudinal and Azimuthal Triptycene‐Fused Tetrapodal Molecular Motors

**DOI:** 10.1002/anie.202513922

**Published:** 2025-10-23

**Authors:** Kateřina Bezděková, Lukáš Severa, Eva Kaletová, Katarina Majerová Varga, Milan Mašát, Liang‐Ting Wu, Jyh‐Chiang Jiang, Ivana Císařová, Jiří Kaleta

**Affiliations:** ^1^ Institute of Organic Chemistry and Biochemistry of the Czech Academy of Sciences Flemingovo nám. 2 Prague 6 160 00 Czech Republic; ^2^ Department of Organic Chemistry Faculty of Chemical Technology University of Chemistry and Technology, Prague Technická 5 Prague 166 28 Czech Republic; ^3^ Department of Chemical Engineering National Taiwan University of Science and Technology Taipei 106 Taiwan; ^4^ Department of Inorganic Chemistry Faculty of Science Charles University Hlavova 2030 Prague 2 128 40 Czech Republic

**Keywords:** Molecular devices, Molecular motor, Photoresponsive surface, Self‐assembled monolayer, Triptycene

## Abstract

Two light‐driven molecular motors, fused to a triptycene‐based tetrapodal platform, with rotational axes oriented either parallel or perpendicular to the surface, were successfully designed and synthesized. Both systems demonstrated complete 360° rotation cycles, efficient photoswitching at 385 ± 5 nm (reaching ∼90% at the photostationary state), and quantitative thermal helix inversion with half‐lives of ∼7 min at 20 °C. When assembled as monolayers on gold surfaces, the motors retained their full rotational functionality, demonstrating the ability of the tetrapodal platform to minimize surface interactions. These findings highlight the potential of these systems for applications in surface‐integrated molecular devices and machines.

## Graphical abstract

Two tetrapodal triptycene‐based light‐driven molecular motors with precisely oriented rotation axes—parallel or perpendicular to a gold surface—achieve full 360° rotation in both solution and as monolayers. The design minimizes surface quenching, enabling functional integration into surface‐mounted nanomachines.

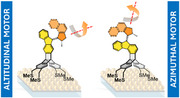

## Introduction

Numerous molecular photoswitches and light‐driven rotary motors, representing fundamental modes of motions such as translation and rotation, have naturally become integral components of molecular machines and devices.^[^ ^1^, ^2^, ^3^, ^4^ ^]^ Realizing systems capable of converting light energy into macroscopic action, however, requires control across multiple hierarchical levels, enabling the amplification of individual molecular events. Substantial progress has been achieved in 3D systems, where non‐exhaustive examples include contractile hydrogels,^[^ ^5^, ^6^, ^7^, ^8^ ^]^ artificial molecular muscles,^[^ ^9^, ^10^ ^]^ and materials with switchable porosity,^[^ ^11^ ^]^ among others.

An equally dynamic and rapidly evolving area of research focuses on surface‐mounted molecular machines, which offer unique opportunities for nanoscale addressability and collective function. Surface‐confined systems have already enabled stimuli‐responsive surfaces with switchable wettability^[^ ^12^ ^]^ and components relevant to nanoelectronics.^[^ ^13^, ^14^, ^15^, ^16^ ^]^ Nevertheless, designing molecular machines, preferably unidirectional light‐driven motors, that operate efficiently on surfaces remains challenging and requires careful consideration of seven critical parameters^[^ ^17^ ^]^: (i) Defined initial orientation of the machine on the surface to ensure uniform behavior and facilitate directional response. (ii) Precise positioning of the rotation axis relative to the surface—altitudinal systems bring the rotor closer to or further from the surface during the rotation cycle, while azimuthal systems rotate within the surface plane and can sequentially interact with defined sites. (iii) Strong surface adhesion, preferably via multidentate anchoring groups, to suppress lateral diffusion and ensure positional stability. (iv) Sufficient electronic decoupling of the chromophore from the metallic substrate to avoid quenching of excited states and loss of photoreactivity. (v) Prevention of tilting or bending, which could compromise structural integrity and motor function. (vi) Suppression of the rotation of the entire motor unit, ensuring that only the rotor undergoes motion while the stator remains immobilized. (vii) Adequate separation between neighboring chromophores to avoid undesired π–π stacking and minimize steric interference, thereby preserving the individual functionality of each motor unit.^[^ ^17^ ^]^

While controlling the absolute orientation of individual molecules with respect to compass direction (e.g., north/south/east/west) remains a significant unresolved challenge, the remaining six criteria have already been addressed, either individually or in combination, in prior studies.^[^ ^12^, ^18^, ^19^, ^20^, ^21^, ^22^, ^23^, ^24^, ^25^, ^26^ ^]^ As molecular nanomachinery continues to grow in complexity, there is an increasing demand for more sophisticated and geometrically refined molecular building blocks with well‐defined photophysical and mechanical properties that simultaneously satisfy these six implementable design requirements.

**Figure 1:**
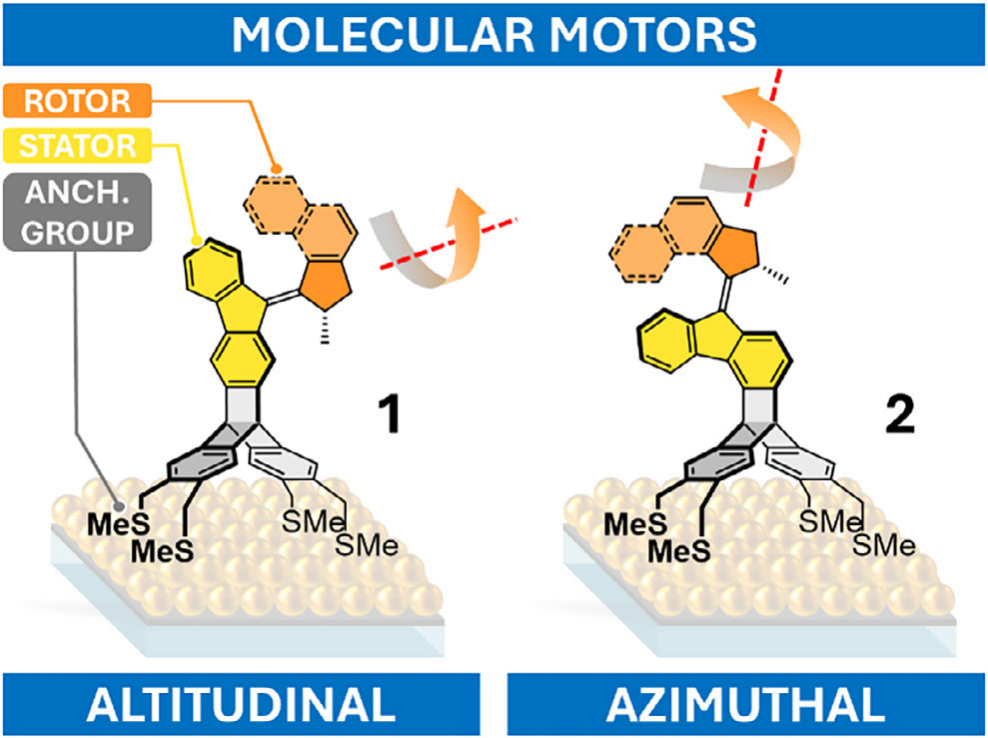
Triptycene‐based tetrapodal unidirectional light‐driven molecular motors with nearly altitudinal (**1**) and azimuthal (**2**) rotation of rotors installed on a gold surface. Only the (*S*)‐enantiomers of a racemic mixture are depicted for clarity.

Building on our^[^ ^20^, ^27^, ^28^ ^]^ and others’^[^ ^29^, ^30^, ^31^ ^]^ previous expertise in triptycene chemistry, photoswitch design,^[^ ^32^, ^33^ ^]^ and surface chemistry,^[^ ^34^, ^35^ ^]^ we now report two geometrically unique, photoresponsive mechanical units. These newly developed systems, denoted as **1** and **2** (Figure 1), are based on a tetrapodal triptycene core and represent versatile additions to the molecular nanomachinery toolkit. Both molecules are designed to address all six aforementioned parameters. The two prototypes differ in the orientation of their rotation axes, dictated by the position of the double bond connecting the indanone‐based rotor (orange) and the fluorenone‐based stator (yellow). In the altitudinal system **1**, the axis forms an angle of about 14° with the surface, corresponding to an almost parallel alignment. By contrast, in the azimuthal system **2**, the axis is inclined by approximately 78°, in line with a nearly perpendicular arrangement.

Strong adhesion to gold surfaces is ensured by four peripheral anchoring groups, while the rigid triptycene‐based pedestal provides both mechanical support and spatial separation from neighboring chromophores, thereby reducing undesired π–π stacking. Owing to its slightly larger footprint (∼750 × 630 pm) compared to the chromophore itself (the indanone‐based rotor is ∼550 pm in length), the pedestal further diminishes inter‐rotor interactions, although this effect is less pronounced for the altitudinal motor. Importantly, undesired energy transfer cannot be completely excluded.^[^ ^17^ ^]^ The inherent stiffness of the architecture prevents tilting or bending, and the stator is firmly embedded within the triptycene core, effectively eliminating rotation of the entire motor upon surface immobilization while leaving the rotor mobile under light stimulation.

In this study, we report the modular synthesis of both altitudinal **1** and azimuthal **2** molecular motors, their photochemical characterization in solution, including determination of kinetic parameters for the thermal steps of the rotary cycle, their formation of self‐assembled monolayers (SAMs) on gold surfaces, and most importantly, their light‐induced functionality upon immobilization on Au(111).

## Results and Discussion

### Synthesis

**Scheme 1:**
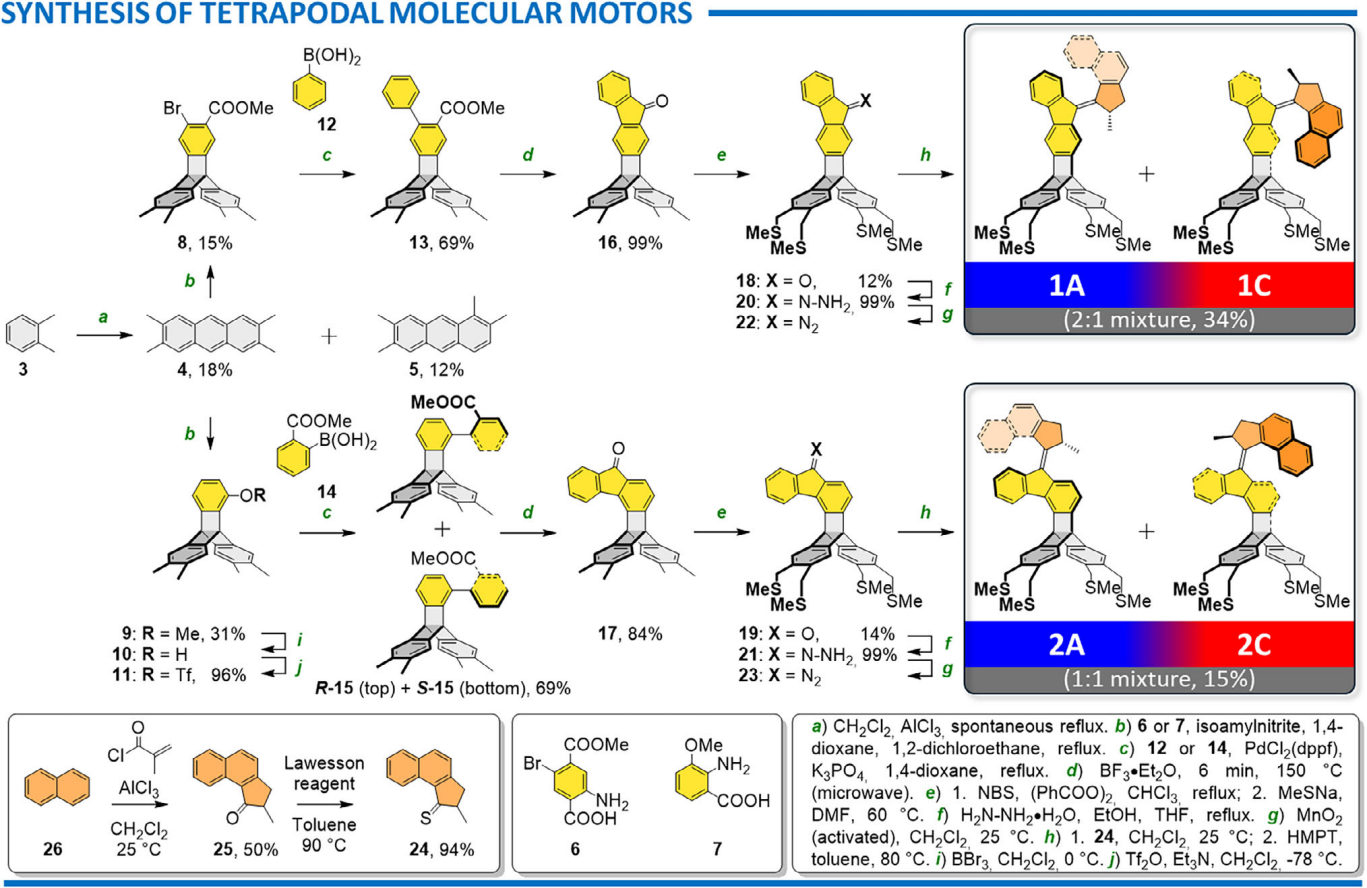
Construction of triptycene‐based altitudinal **1** and azimuthal **2** light‐driven molecular motors. Capital letters A and C refer to individual rotation stages depicted in Figure 3, and only the (*S*)‐enantiomers of a racemic mixture of both molecular motors are depicted for clarity.

Both **1** and **2** were synthesized in eight and ten steps from *o*‐xylene (**3**) (Scheme 1). The synthetic pathway started with an AlCl_3_ catalyzed reaction between **3** and CH_2_Cl_2,_ affording a 3:2 mixture of anthracenes **4** ^[^ ^36^ ^]^ and **5**, with an overall yield of 30%. Simple trituration with CH_2_Cl_2_ effectively separated **5** from poorly soluble **4**. The subsequent step involved Diels–Alder reaction between **4** and benzynes generated in situ either from **6** or **7** and isoamyl nitrite, resulting in triptycenes **8** and **9** in 15% and 31%, respectively. The choice of benzyne precursor determined the geometry of the final product; **6** lead to **1**, while **7** to **2**. The BBr_3_ induced demethylation of **9** gave phenol **10**, which was converted to triflate **11** in nearly quantitative yield over two successive steps. Subsequent Suzuki cross‐coupling between triptycene **8** and phenylboronic acid (**12**) gave **13** in 69% yield. The same coupling between triptycene **11** and **14** afforded two atropisomers ***R‐*15** and ***S‐*15** in 69% isolated yield. The experimentally determined barrier for their mutual interconversion in DMSO is 80 ± 4 kJ mol^−1^, which reasonably agrees with the density functional theory (DFT) calculated value of 83 kJ mol^−1^ (see Supporting Information for more details), thus preventing their separation. Ester **13** was quantitatively cyclized in BF_3_·Et_2_O at 150 °C in microwave to fluorenone **16** and the same reaction conditions were used to convert both atropisomers of **15** to **17**.

The next synthetic effort involved installation of thioether‐based anchoring units. Both **16** and **17** were brominated on all four methyl groups using NBS in the presence of dibenzoylperoxide, and the mixture of brominated compounds was converted to thioethers **18** and **19** using MeSNa in DMF in an overall ∼13% yield.

The final assembly of molecular motors **1** and **2** relied on the well‐established Barton–Kellogg olefination. In this reaction series, the stators **18** and **19** underwent complete conversion (as determined by ^1^H NMR) to the corresponding hydrazones **20** and **21** upon reacting with hydrazine hydrate in a refluxing mixture of ethanol and THF or DMF. Subsequently, oxidation by MnO_2_ yielded the corresponding diazo compounds **22** and **23**, which, upon reaction with thioketone **24**,^[^ ^37^ ^]^ followed by *N*,*N*,*N*′,*N*′,*N*′′,*N*′′‐hexamethylphosphanetriamine (HMPT) addition, resulted in a 2:1 mixture of the desired motors **1A**/**C** and a 1:1 mixture of **2A**/**C**. Thioketone **24** was prepared using Lawesson's reagent from ketone **25**, which is easily accessible from anthracene (**26**) and methacryloyl chloride in the presence of AlCl_3_.^[^ ^38^ ^]^

Both **1A** and **1C** were smoothly separated using HPLC on a silica gel column. In contrast, all attempts to separate **2A**/**C** on a preparative scale yielded only enriched fractions (**2A**:**2C** = 4:1 and **2C**:**2A** = 3:1).

**Figure 2:**
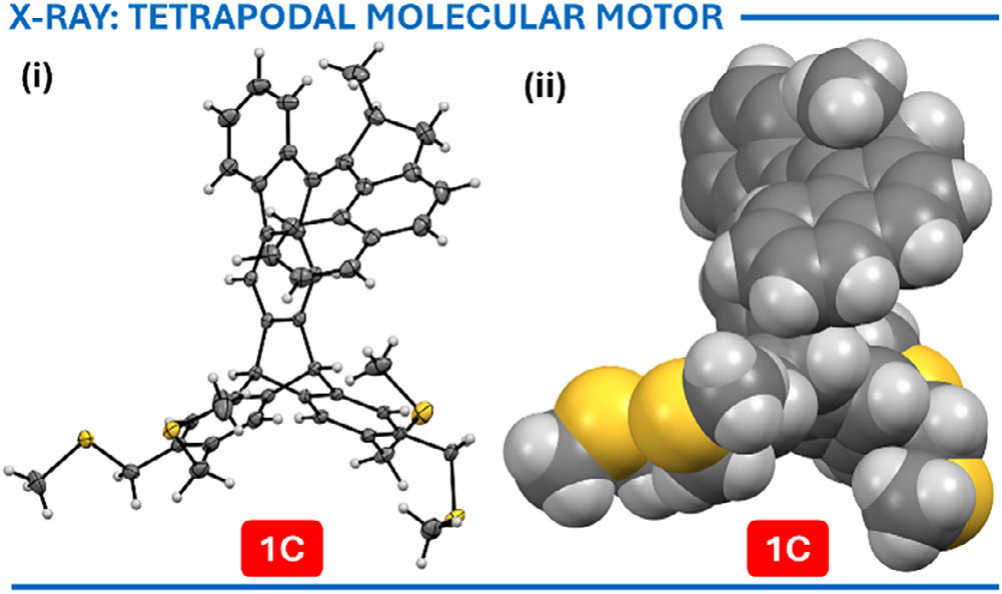
X‐ray structure of single enantiomer ***S*‐1C**: i) ORTEP (ellipsoids shown on 30% probability level), and ii) space‐filling model.

The geometry of nearly all stable intermediates (**4**, **5**, **9**, **13**, ***R*‐15**, ***S*‐15**, **16**, **17**, **18**, and **S2**), as well as the configuration of the final motor **1C**, was unambiguously confirmed by X‐ray diffraction of the corresponding monocrystals. Interestingly, the enantiomers of racemic **1C** crystallized separately, and only the structure of enantiomer ***S***‐**1C** depicted in Scheme 1 was obtained (Figure 2).

### Switching in Solution

**Figure 3:**
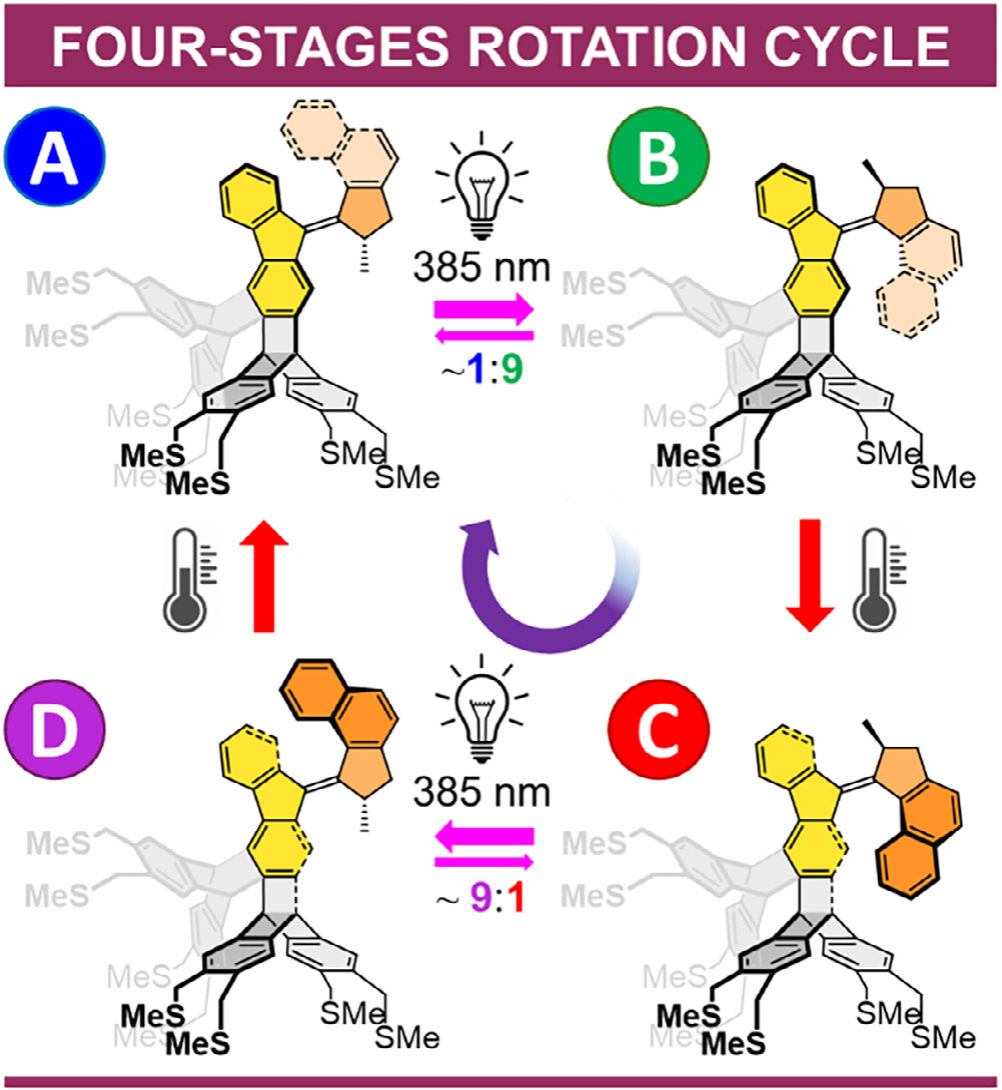
Four‐stage unidirectional rotation cycle of molecular motors **1** and **2**, consisting of two photochemical (A→B and C→D) and thermal (B→C and D→A) steps. For clarity, both chromophores are shown overlapped, with the second structure displayed in reduced contrast; only the (*S*)‐enantiomers of the racemic mixture are depicted. Separate high‐resolution depictions of both motors are provided in the Supporting Information (Figures S16 and S17).

The functional cores of both triptycene‐based systems **1** and **2** are derived from a previously reported unidirectional light‐driven molecular motor.^[^ ^20^, ^39^ ^]^ Its four‐stages 360° rotation cycle (Figure 3) consists of two photochemical double bond *E/Z* isomerizations (A→B and C→D) alternated with two thermal relaxations characterized by helicity inversion (B→C and D→A).^[^ ^40^ ^]^

**Figure 4:**
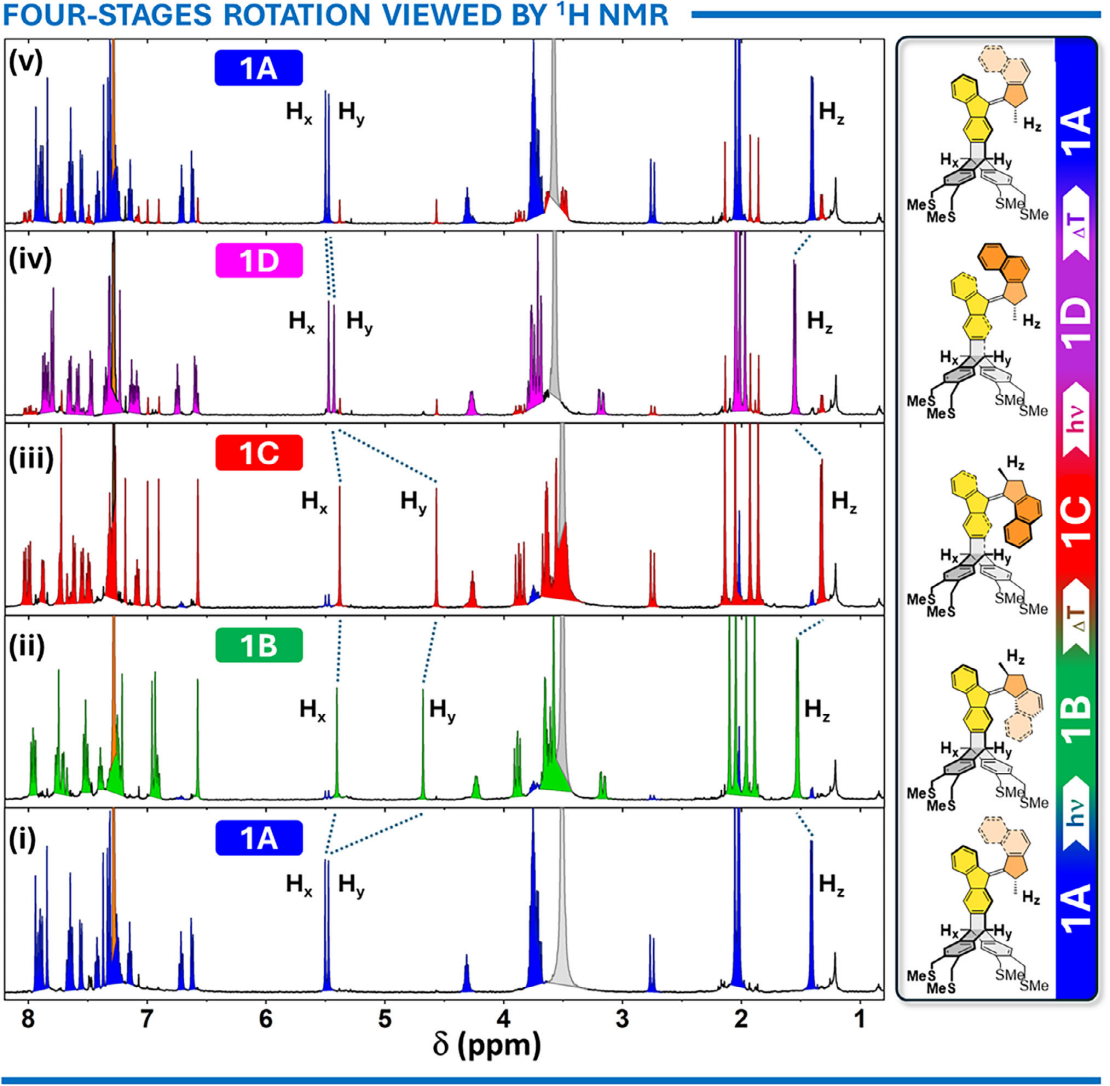
The ^1^H NMR spectra in CDCl_3_ describing the full rotation cycle **1A**→**1B**→**1C**→**1D**→**1A** are as follows: i) **1A** at −20 °C, ii) a 9:1 mixture of **1B** and **1A** in PSS obtained by irradiating pure **1A** at 385 ± 5 nm and −20 °C, iii) **1C** obtained after thermal relaxation of compounds (ii), iv) a 9:1 mixture of **1D** and **1C** in PSS obtained by irradiating pure **1C** at 385 ± 5 nm and −20 °C, and v) **1A** obtained after thermal relaxation of **1D**. The blue, green, red and purple peaks correspond to the color scheme used to distinguish between individual isomers in Figure 3. The orange peak at 7.26 ppm is residual CHCl_3_ in CDCl_3_ and gray peak at 3.30 ppm is due to water at −20 °C. Only the (*S*)‐enantiomers of the racemic mixture are depicted for clarity.

The smooth functionality of both tetrapods **1** and **2** in chloroform solutions was independently confirmed through ^1^H NMR and UV–vis spectroscopies. Figure 4 shows a complete unidirectional rotation cycle **1A**→**1B**→**1C**→**1D**→**1A** as monitored by ^1^H NMR. This process can be tracked using several hydrogen atoms within the molecule (see Figure S16). Here, the two bridgehead protons H_x_ and H_y_ on the stator and the methyl signal H_z_ on the rotor were selected for analysis.

In **1A**, H_x_ and H_y_ exhibit nearly identical chemical shifts around ∼5.5 ppm, while H_z_ resonates at ∼1.4 ppm. Irradiation of **1A** (blue peaks in Figure 4) in a CDCl_3_ solution cooled to −20°C using a LED with a 385 ± 5 nm emission maximum resulted in the formation of the thermally unstable motor **1B** (green peaks in Figure 4) at 90% photostationary state (PSS). The naphthyl portion of the rotor approaching the triptycene stator caused a significant upfield shift of H_y_ to ∼4.7 ppm and a downfield shift of H_z_ to ∼1.6 ppm, while H_x_, located on the opposite side of the molecule, remained largely unaffected. Allowing the sample to equilibrate in the dark at 20 °C for 30 min quantitatively converted **1B** to **1C** (red peaks in Figure 4) through thermal helix inversion. This transition further shielded H_y_, shifting it upfield to ∼4.6 ppm, and moving H_z_ upfield to ∼1.3 ppm.

The second half of the rotation cycle was initiated by irradiating **1C** at 385 ± 5 nm, producing the thermally unstable motor **1D** (purple peaks in Figure 4) at 89% PSS. In this step, the naphthyl rotor moved away from the triptycene stator, causing H_y_ to deshield and shift from ∼4.6 to ∼5.5 ppm, while H_z_ shifted downfield to ∼1.7 ppm. Finally, thermal helix inversion at 20 °C for 30 min quantitatively regenerated **1A**, completing the full rotation cycle. During this final step, H_x_ and H_y_ returned to their original chemical shifts (∼5.5 ppm), and H_z_ moved back upfield to ∼1.4 ppm. Spectral evidence for photoswitching in **2A/C** is presented in the Supporting Information (see Figure S17).

**Figure 5:**
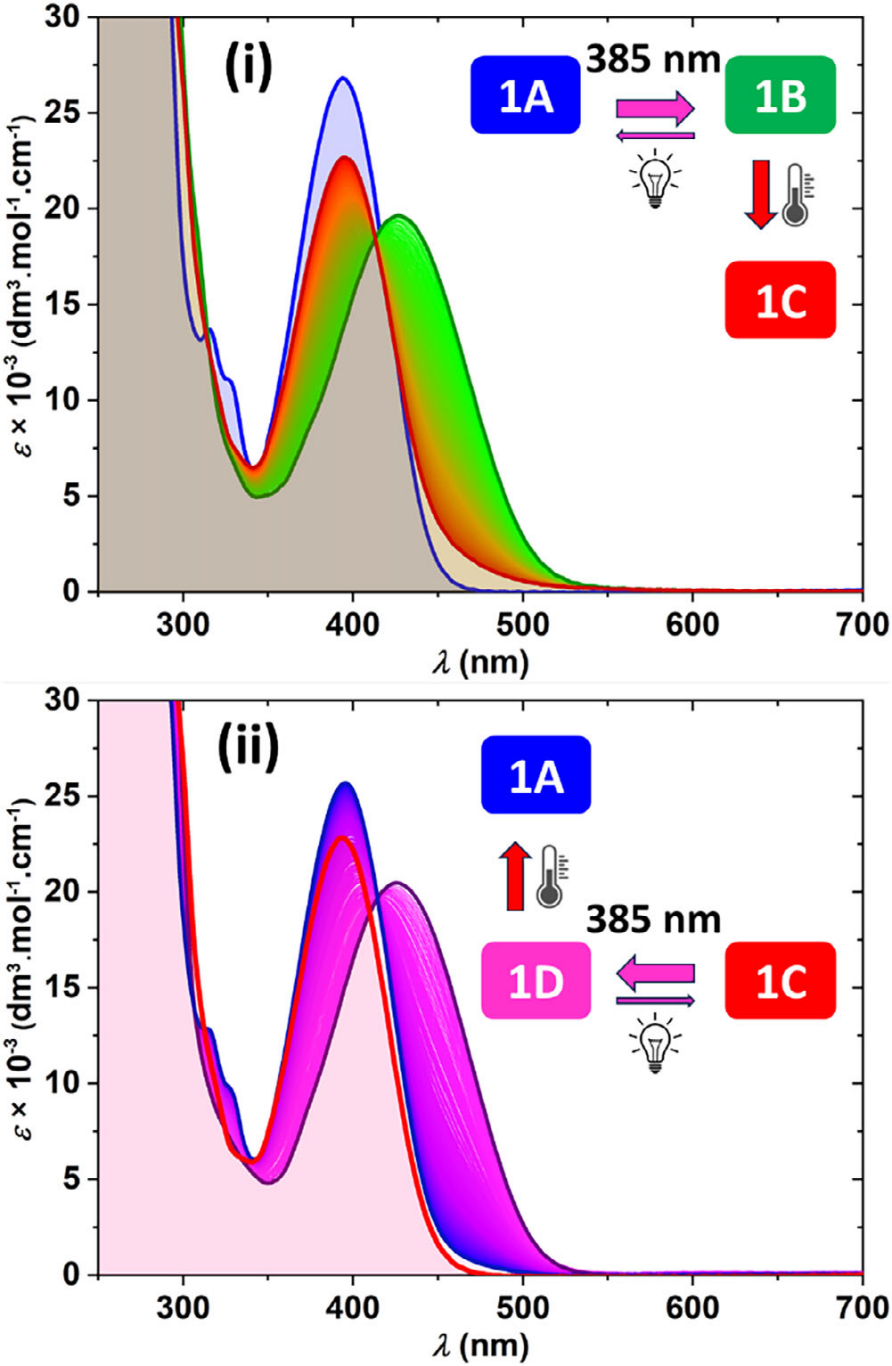
Rotation cycle **1A**→**1B**→**1C**→**1D**→**1A** followed by UV–vis at 0 °C. i) First half of the rotation cycle showing **1A** (blue line), the same sample in PSS after irradiation at 385 ± 5 nm (**1B**, green line), and after subsequent thermal relaxation to **1C** (red line). ii) Second half of the rotation cycle showing **1C** (red line), the same sample in PSS after irradiation at 385 ± 5 nm (**1D**, purple line), and after subsequent thermal relaxation back to initial **1A** (blue line).

The UV–vis spectra of both **1A/C** and **2A/C** are nearly identical and are characterized by an absorption band centered at ∼390 nm. However, only **1A**/**2A** also contain indicative absorption bands at ∼310 and ∼340 nm, which aid in determining the directionality of the rotary motion. Figure 5 shows a representative example of photoswitching followed by thermal relaxation observed in **1A** and **1C** through UV–vis analysis (spectra of **2A/C** are in the Supporting Information, see Figures S12 and S14). Upon irradiation of **1A/C** at 385 ± 5 nm, a noticeable bathochromic shift of the absorption band from ∼390 to ∼430 nm occurs. This shift is attributed to the formation of unstable isomers **1B/D** with an increased twist of the central double bond. Subsequent thermal helix inversion, conducted in darkness at 0 °C, converts the unstable **1B/D** to thermally stable **1C/A**. Importantly, the presence of isosbestic points at ∼410 nm for **1A**→**1B** and **1C**→**1D**, and ∼420 nm for **1B**→**1C** and **1D**→**1A** indicates that both the photochemical as well as thermal isomerization are clean processes.

Although the excited‐state processes underlying photoisomerization take place on the picosecond time scale,^[^ ^41^, ^42^ ^]^ the overall rotational speed is governed by the quantum yield of productive photoisomerization and the rate of the subsequent thermal helix inversion.^[^ ^43^, ^44^, ^45^ ^]^ The kinetics of the slow step were studied by time‐dependent UV–vis spectroscopy at six temperatures (0, 5, 10, 15, 20, and 25 °C), and results were validated by ^1^H NMR. Corresponding basic kinetic parameters (see Tables S2–S5) like enthalpy (Δ*H* ^‡^) and Gibbs free energy of activation (Δ*G* ^‡^), together with a half‐life at 20 °C (*t*_1/2_) and a corresponding rate constant (*k* ^0^) are summarized in Table 1. Generally, the thermal isomerization of the indene rotor above the phenyl ring fused to the triptycene core (B→C transition in Figure 3) exhibited higher activation energy and longer half‐lives (∼8 min for **1B**→**1C** and ∼9 min for **2B**→**2C**) compared to the isomerization above the terminal phenyl ring of the fluorene stator (D→A transition: ∼6 min for **1D**→**1A** and ∼7 min for **2D**→**2A**).

**Table 1:** Photostationary states (PSS), enthalpy (Δ*H* ^‡^) and Gibbs free energy of activation (Δ*G* ^‡^), rate constants (*k* ^0^), and half‐life (*t*_1/2_) of **1A**, **1C**, **2A**, and **2C** determined in chloroform, along with corresponding values for **1** and **2** on a gold surface.

		Kinetic parameters			
Cmpd.	PSS^a)^	Δ*H* ^‡^ (kJ mol^−1^)	Δ*G* ^‡^ (kJ mol^−1^)	*k* ^0^ (s^−1^)	*t*_1/2_ ^b)^(min)
**1A**	10:90	79 ± 1	88 ± 2	1.43 ± 0.05 × 10^−3^	8.1 ± 0.3
**1C**	11:89	79 ± 1	87 ± 3	1.98 ± 0.09 × 10^−3^	5.8 ± 0.3
**2A**	12:88	81 ± 1	88 ± 1	1.34 ± 0.02 × 10^−3^	8.6 ± 0.1
**2C**	11:89	81 ± 2	87 ± 4	1.71 ± 0.11 × 10^−3^	6.8 ± 0.4
**1** on Au	–^c)^	58 ± 21	88 ± 40	1.3 ± 0.3 × 10^−3^	9 ± 3
**2** on Au	–^c)^	64 ± 21	87 ± 42	1.7 ± 0.4 × 10^−3^	7 ± 2

^a)^ PSS between A and B, and C and D isomers achieved at 385 ± 5 nm as determined by ^1^H NMR.

^b)^ Half‐life (*t*_1/2_) of thermal B→C and D→A reactions reported at 20 °C.

^c)^ PSS was not determined for surface‐adsorbed motors.

Importantly, both structures are very stable, and no traces of decomposition were detected even after more than 20 rotation cycles initiated by irradiation at 385 ± 5 nm at room temperature.

### Motors on a Gold Surface

Both motors **1** and **2**, along with ketones **18** and **19** as references, were adsorbed onto an Au(111) substrate on glass from 100 µM acetonitrile or THF solutions. All four compounds, which share tetrathioether‐based anchors but differ in height, formed compact SAMs with seldom defects, as confirmed by atomic force microscopy (AFM).

The theoretically predicted SAM thickness aligns well with experimental values obtained through ellipsometry and AFM scratch test (Table S7). The SAMs formed from **1** were ∼1.6 nm thick, while those from **2** measured ∼1.3 nm, and ketones **18** and **19** produced monolayers with a thickness of ∼1.2 nm. In all cases, the monolayer thickness corresponded to the expected orientation of tetrapods, with the thioether anchors binding them to the gold surface. The presence of characteristic vibrations within SAMs was confirmed through polarization‐modulation infrared reflection‐absorption spectroscopy (PM‐IRRAS) and surface‐enhanced Raman spectroscopy (SERS). The expected elemental composition was confirmed via photoelectron spectroscopy (XPS). For further information and spectral data, see Supporting Information.

**Figure 6:**
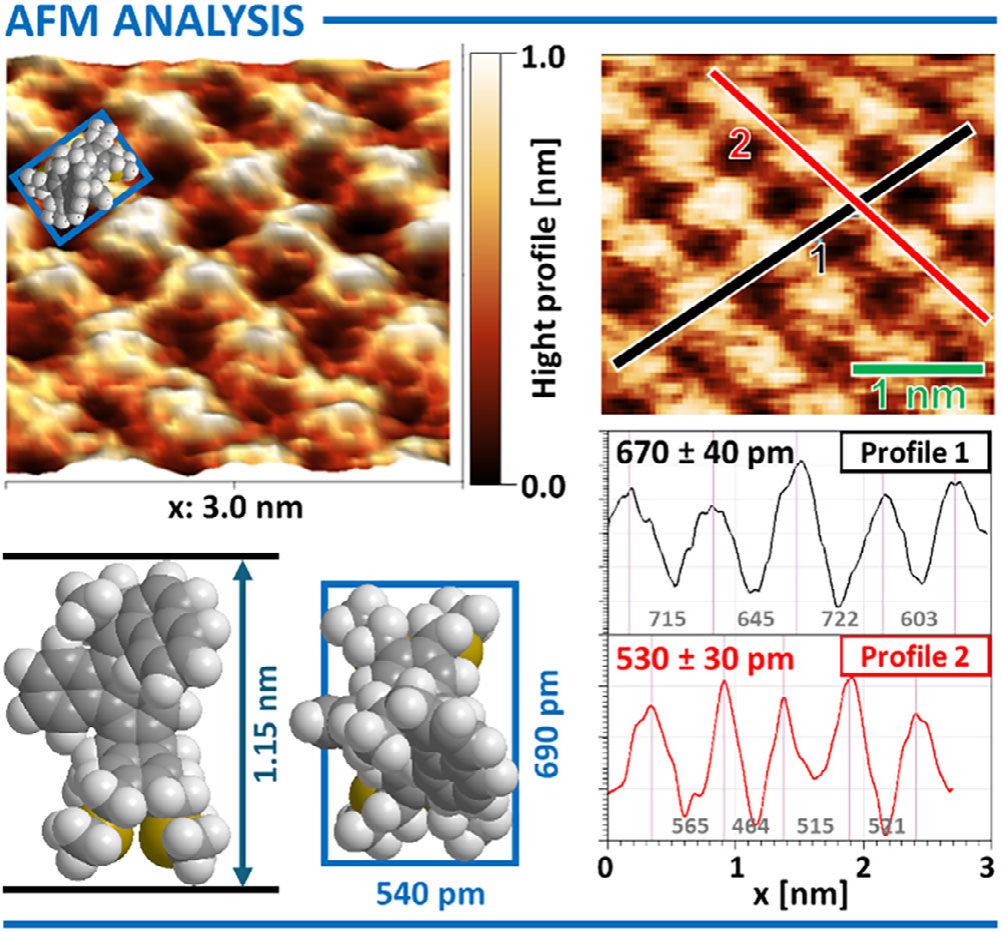
AFM visualization of SAMs formed by tetrapodal motor **2** on an Au(111), including top and side views of the space‐filling model of gas‐phase optimized **2C** with annotated molecular dimensions, and topography charts illustrating the periodicity of the resulting film.

Furthermore, high‐resolution AFM imaging of the SAM formed by motor **2** revealed fine topographic features, including periodically arranged domains with line spacings of 670 ± 40 pm along one axis and 530 ± 30 pm along the nearly orthogonal axis. The observed height variation was ∼1 nm (Figure 6). These periodic features are in good agreement with the dimensions of the gas‐phase DFT‐optimized structure of motor **2C**, which has a molecular footprint of ∼690 × 540 pm and a height of ∼1.2 nm. This correlation suggests that the observed surface pattern likely reflects an ordered molecular packing of the motors within the SAM (Figure 6).

To better understand the adsorption behavior of tetrapodal platforms on an Au(111), DFT calculations were carried out using ketone **19** as a representative model. Upon surface adsorption, the four sulfur atoms from the thioether moieties were found to form strong Au–S bonds with surface gold atoms.

**Figure 7:**
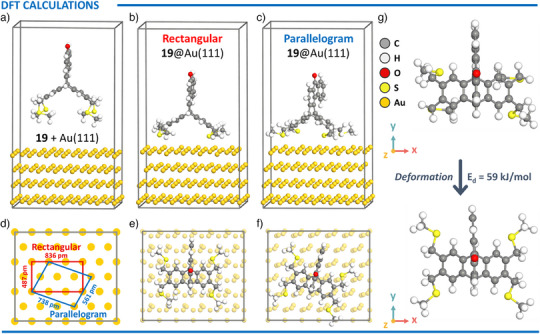
Optimized structures of model tetrapod **19**: a) The **19** away from the Au(111) surface. Adsorption of **19** on Au(111) via b) rectangular‐type and c) parallelogram‐type configurations. d) Schematic representation of the two adsorption configurations. Top views of **19**@Au(111) via e) rectangular‐type and f) parallelogram‐type configurations. g) Deformation of **19** following parallelogram‐type adsorption.

Two distinct adsorption geometries were identified: a rectangular and a parallelogram configuration (Figure 7b,c, respectively). In the rectangular arrangement, the bound gold atoms occupied the vertices of a rectangle with sides measuring 487 and 836 pm. In contrast, the parallelogram configuration—closely matching the periodicity observed in AFM experiments—featured gold atoms positioned at the corners of a parallelogram with side lengths of 561 and 738 pm (Figure 7d). Top views of both configurations are shown in Figure 7e,f.

The calculated adsorption energies of the **19**@Au(111) system were −266 kJ mol^−1^ (−2.76 eV) for the rectangular configuration and −343 kJ mol^−1^ (−3.55 eV) for the parallelogram configuration, indicating a strong interaction between the tetrapodal platform and the Au(111) surface in both cases. The parallelogram configuration, thermodynamically favored due to its higher adsorption energy, exhibited a deformation of the tetrapodal anchor upon adsorption. As shown in Figure 7g, the four legs of **19** rotated to align with the gold atoms, positioning the four methyl groups of the thioether segments outward and the four sulfur atoms inward. This deformation, introducing a strain energy penalty of 59 kJ mol^−1^ (0.62 eV), was largely compensated by the high adsorption stabilization of the parallelogram configuration. See Supporting Information for more details.

**Figure 8:**
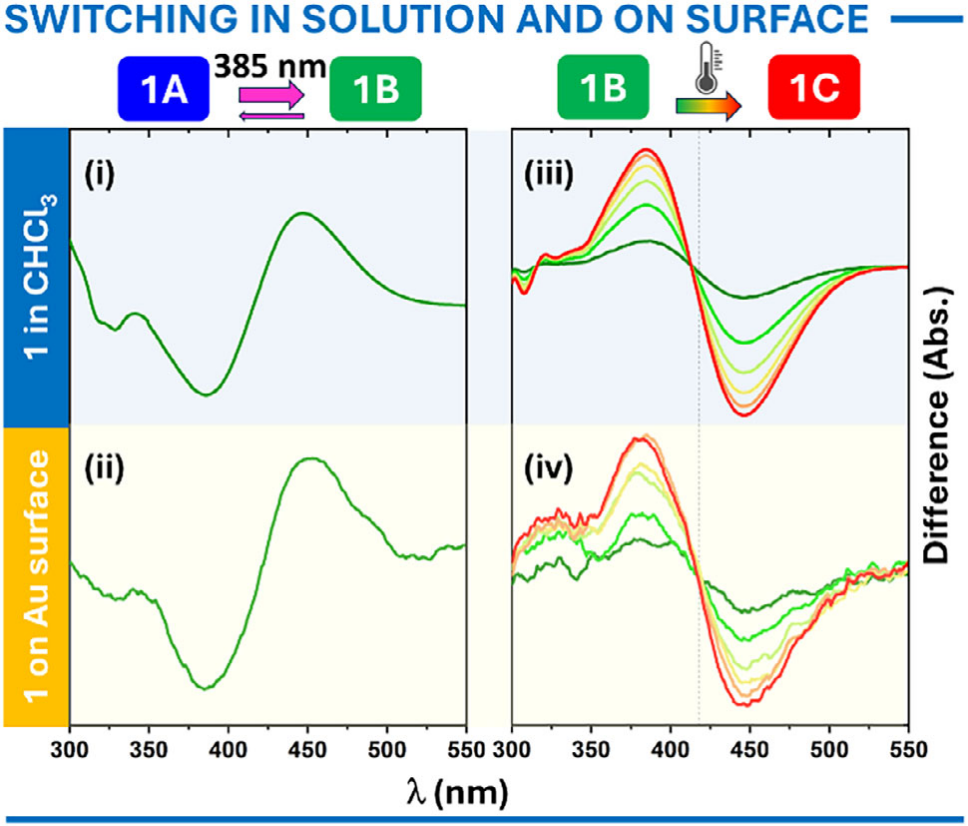
UV–vis differential absorption spectra of **1A** in chloroform solution (i) and within a SAM (ii) before and after illumination at 385 ± 5 nm, recorded at 10 °C. The subsequent thermal relaxation of predominantly **1B** to **1C** in chloroform solution (iii) and in SAM (iv) at 10 °C is also observed through differential UV–vis absorption spectra.

The final, and perhaps most intriguing, questions explored were whether the surface‐anchored molecules retained their functionality and if the triptycene scaffold effectively decouples them from the metallic surface. To investigate this, UV–vis absorption spectroscopy was employed. For these measurements, optically semi‐transparent substrates were prepared by depositing a 5 nm gold layer onto a silylated quartz surface (details in the Supporting Information).^[^ ^46^, ^47^ ^]^ As both motors showed similar behavior, the results for **1A** are shown here, while data for **2A** can be found in the Supporting Information (Figure S30). When the SAM of **1A** was illuminated at 385 ± 5 nm, a bathochromic shift in the absorption band was observed, shifting from ∼390 nm to ∼450 nm. This shift, evident in the differential spectrum (Figure 8ii), matches the photoisomerization observed in solution (Figures 5 and 8i). Subsequent thermal helix inversion, characterized by a decrease in the absorption band at ∼450 nm and an increase at ∼390 nm, was clearly detected during time‐dependent UV–vis analysis of SAMs (Figure 8iv) and aligns well with differential spectra in solution (Figure 8iii). Kinetic analysis performed at six temperatures (0, 2.5, 5, 7.5, 10, and 12.5 °C) yielded a half‐life of 9 ± 3 min at 20 °C, mirroring the solution results (Table 1). The isosbestic point at ∼420 nm confirmed the process proceeded cleanly without detectable side reactions.

## Conclusion

In conclusion, two light‐driven molecular motors fused to a triptycene‐based tetrapodal pedestal—differing in the orientation of their rotational axes (parallel and perpendicular to the surface)—were designed and successfully synthesized. Their complete 360° rotation cycles were thoroughly characterized in solution using ^1^H NMR and UV–vis spectroscopy. Both motors, **1** and **2**, exhibited efficient photoswitching at 385 ± 5 nm to unstable forms, achieving a high PSS (∼90%). These structures then quantitatively underwent thermal helix inversion with average half‐lives of ∼7 min at 20 °C. Surface characterization using AFM, ellipsometry, SERS, and PM‐IRRAS confirmed the formation of compact and well‐ordered SAMs on Au(111). Notably, high‐resolution AFM imaging of the SAM derived from motor **2** revealed periodic topographical features with lateral spacings and molecular heights that correlate well with dimensions predicted by DFT calculations, providing evidence for ordered molecular packing on the surface. Crucially, UV–vis analysis demonstrated that the surface‐mounted motors retained their full rotational functionality. A comparison of the kinetic parameters in solution and on the surface revealed that the tetrapodal platform effectively decouples the chromophores from the metallic surface. Combined with the established chemical functionalization of these rotors, these systems are promising candidates for the development of advanced surface‐mounted molecular machines.

## Supporting Information

The Supporting Information includes synthetic procedures, copies of ^1^H and ^13^C NMR spectra of all new compounds, assignments of ^1^H and ^13^C NMR signals for **1**, UV–vis and ^1^H NMR analysis of photoswitching followed by thermal helix inversion in solutions of **1** and **2**, preparation of gold substrates, characterization of SAMs (AFM visualizations and scratching experiments, ellipsometry, contact angle goniometry, PM‐IRRAS, SERS, XPS, photoswitching monitored by UV–vis), computational details, ORTEP views of a single molecule, and packing in all 10 crystal structures (**1C**, **4**, **5**, **9**, **13**, **15**, **16**, **17**, **18**, and **S2**) (PDF).

Deposition Number(s) 2434427 (for **1C**), 2434426 (for **4**), 2434429 (for **5**), 2434425 (for **9**), 2434424 (for **13**), 2434430 (for **15**), 2434428 (for **16**), 2434431 (for **17**), 2434423 (for **18**), 2411038 (for **S2**) contain(s) the supplementary crystallographic data for this paper. These data are provided free of charge by the joint Cambridge Crystallographic Data Centre and Fachinformationszentrum Karlsruhe Access Structures service.

Optimized geometries of **19**@Au(111), both atropisomers ***R*‐15** and ***S*‐15**, and corresponding transition states shown in Figure S36 (xyz).

The authors have cited additional references within the Supporting Information.^[^ ^48^, ^49^, ^50^, ^51^, ^52^, ^53^, ^54^, ^55^, ^56^, ^57^, ^58^, ^59^, ^60^, ^61^, ^62^, ^63^, ^64^, ^65^, ^66^, ^67^ ^]^

## Conflict of Interests

The authors declare no conflict of interest.

## Supporting information



Supporting Information

Supporting Information

## Data Availability

The data that support the findings of this study are available in the Supporting Information of this article.
