# Supplementary material for: Surface‐Decoupled Altitudinal and Azimuthal Triptycene‐Fused Tetrapodal Molecular Motors
Source: Angew Chem Int Ed Engl. 2025 Oct 23;65(2):e202513922. doi: 10.1002/anie.202513922 (PMC12790369; doi:10.1002/anie.202513922)

Supplementary Information

Surface-Decoupled Altitudinal and Azimuthal Triptycene-Fused Tetrapodal Molecular Motors

Kateřina Bezděková,[a,b] Lukáš Severa,[a] Eva Kaletová,[a] Katarina Majerová Varga,[a] Milan Mašát,[a] Liang-Ting Wu,[c] Jyh-Chiang Jiang,[c] Ivana Císařová,[d] and Jiří Kaleta[a,]*

[a] *Institute of Organic Chemistry and Biochemistry of the Czech Academy of Sciences, Flemingovo nám. 2, 160 00 Prague 6, Czech Republic.*
[b] *Department of Organic Chemistry, Faculty of Chemical Technology, University of Chemistry and Technology Prague, Technická 5, Prague 166 28, Czech Republic*
[c] *Department of Chemical Engineering, National Taiwan University of Science and Technology, Taipei 106, Taiwan.*
[d] *Department of Inorganic Chemistry, Faculty of Science, Charles University in Prague, Hlavova 2030, 128 40 Prague 2, Czech Republic.*

Table of Contents

1. General Information....................S2
2. Synthesis of **1** and **2**....................S3
3. Synthesis of Anthranilic Acid **6**....................S15
4. HPLC Separation of Compounds **1** and **2**....................S18
5. X-Ray Diffraction....................S20
6. UV-vis Analysis in Solution....................S22
7. ^{1}H NMR Analysis in Solution....................S31
8. Monolayer Preparation....................S34
9. Ellipsometry and Contact Angle Goniometry....................S35
10. Polarization Modulation-Infrared Reflection Absorption Spectroscopy (PM-IRRAS)...S36
11. Raman Spectroscopy....................S38
12. Atomic Force Microscopy (AFM)....................S40
13. XPS Analysis of SAMs on Gold Surface....................S43
14. UV-vis Analysis of SAMs on Gold Surface....................S45
15. Calculations....................S49
16. Assignments of ^{1}H and ^{13}C NMR Signals....................S54
17. NMR Spectra of Prepared Compounds....................S56
18. X-Ray Crystallographic Data....................S192

1. General Information

Materials. All reactions were performed under a nitrogen atmosphere using anhydrous solvents freshly distilled under strictly anhydrous conditions, unless otherwise specified. Standard Schlenk techniques and vacuum line procedures were employed for the handling of air- and moisture-sensitive compounds. Reported yields correspond to isolated materials that were chromatographically and spectroscopically pure, unless stated otherwise. Unless noted, all reagents were obtained from commercial suppliers and used without further purification.

THF and diethyl ether were dried over sodium/benzophenone and distilled under argon prior to use. Toluene and 1,4-dioxane were dried over sodium and distilled under argon and nitrogen, respectively. Dichloromethane (CH_2Cl_2) was dried over $CaCl_2$ and distilled under nitrogen, while chloroform ($CHCl_3$) was dried over CaH_2 and also distilled under nitrogen. Acetonitrile was stored over 4 Å molecular sieves and distilled under argon before use. All other reagents were used as received, unless otherwise stated.

Procedures. Analytical thin-layer chromatography (TLC) was performed using precoated TLC aluminium sheets (Silica gel 60 F_{254}). TLC spots were visualized using either UV light (254 nm) or a 5% solution of phosphomolybdic acid in ethanol, and heat (400 °C) as a developing agent. Flash chromatography was performed using silica gel (high purity grade, pore size 60 Å, 70–230 mesh). The volume/volume (v/v) ratios of solvents were used to prepare mobile phases for column chromatographies. Preparative HPLC were performed using Pure FlashPrep C-835 chromatography system using a PrepPure Silica 250 × 30 mm column with the flow rate of 30 mL/min.

Melting points. Melting points were determined using a Stuart SMP 3 melting point apparatus, and the reported values are uncorrected.

Infrared spectra (IR). IR spectra were recorded using NICOLET 6700 FTIR spectrometer in KBr pellets.

Nuclear Magnetic Resonance (NMR) Spectroscopy. Characterization of prepared compounds by NMR was carried out using a Bruker Avance III^{TM} HD 400 MHz Prodigy spectrometer, a Bruker Avance III^{TM} HD 400 MHz spectrometer or a Bruker Avance III^{TM} HD 600 MHz spectrometer. Irradiation experiments were carried out using a Bruker Avance II^{TM} HD 500 MHz spectrometer. Chemical shifts in ^{1}H, and ^{13}C spectra are reported in ppm on the δ scale relative to $CHCl_3$ (δ = 7.26 ppm for ^{1}H and δ = 77.0 ppm for ^{13}C) and DMSO-d_6 (δ = 2.50 ppm for ^{1}H, and δ = 39.52 ppm for ^{13}C) as internal references. Coupling constants (J) are reported in hertz (Hz). Splitting patterns are assigned as s = singlet, d = doublet, t = triplet, p = pentet, m = multiplet, br = broad signal, dd = doublet of doublets. Structural assignments were made with additional information from APT, COSY, HSQC, and HMBC experiments.

Mass Spectrometry. High-resolution mass spectra (HRMS) using electrospray ionization (ESI) or atmospheric-pressure chemical ionization (APCI) were obtained on ORBITRAP XL (Thermo). EI and CI spectra were recorded using Waters or Agilent 7250 GC/Q-TOF instruments.

2. Synthesis of 1 and 2

Compound 1. A solution of thioketone **24** (165 mg, 0.777 mmol, 1.8 equiv) in anhydrous toluene (5 mL) was prepared in a dry, nitrogen-purged 25 mL Schlenk flask. To this, a solution of diazo compound **22** (270 mg, 0.443 mmol, 1.0 equiv) in toluene (8 mL) was added dropwise. The mixture was stirred at room temperature in the dark for 1 h, after which *N,N,N′,N′,N″,N″*-hexamethylphosphanetriamine (HMPT; 250 µL, 1.38 mmol, 3.1 equiv) was introduced. The reaction was then heated to 80 °C and stirred overnight. Then volatiles were removed using a rotary evaporator and the orange residual solids were subjected to a column chromatography (hexane/CH_2Cl_2 1:1, R_f = 0.4). The fraction containing product was further purified using preparative HPLC on silica gel (55% to 75% CH_2Cl_2 in hexane). Motor **1** (2:1 mixture of **1A**:**1C**, 114 mg, 0.150 mmol, 34%) was obtained as an orange powder and separation of the *E*/*Z* isomers was smoothly achieved using preparative HPLC on silica gel (15% ethyl acetate in hexane, chromatogram in **Figure S1**, **Section 4**).

Compound 1A. Mp 190-195 °C (dec.). ^{1}H NMR (600 MHz, $CDCl_3$): δ 7.98 (s, 1H), 7.93-7.92 (m, 1H), 7.91-7.90 (m, 1H), 7.85 (s, 1H), 7.70-7.67 (m, 2H), 7.58-7.57 (m, 1H), 7.45-7.43 (m, 1H), 7.42 (s, 1H), 7.38 (s, 1H), 7.37 (s, 1H), 7.36 (s, 1H), 7.29-7.26 (m, 1H), 7.18-7.16 (m, 1H), 6.75-6.73 (m, 1H), 6.67-6.65 (m, 1H), 5.50 (s, 1H), 5.48 (s, 1H), 4.37 (p, *J* = 6.6 Hz), 3.82-3.74 (m, 8H), 3.55 (dd, J_1 = 5.7 Hz, J_2 = 15.0 Hz, 1H), 2.78 (d, *J* = 15.0 Hz, 1H), 2.09 (s, 3H), 2.062 (s, 3H), 2.056 (s, 6H), 1.47 (d, *J* = 6.6 Hz, 3H). ^{13}C {^{1}H} NMR (150 MHz, $CDCl_3$): δ 150.7, 147.3, 144.19, 144.16, 144.12, 144.06, 144.0, 139.3, 137.8, 137.5, 137.1, 136.4, 133.07, 133.03, 132.6, 130.8, 130.3, 129.8, 128.6, 127.4, 126.7, 126.5, 125.67, 125.65, 125.60, 125.55, 125.47, 125.2, 123.9, 119.6, 118.4, 115.2, 53.9, 53.4, 45.5, 42.0, 35.54, 35.53, 35.52, 35.50, 19.5, 15.87, 15.85, 15.75. IR (KBr): 3048, 3006, 2959, 2910, 2842, 1610, 1577, 1514, 1468, 1446, 1428, 1340, 1315, 1234, 1212, 1190, 1144, 1055, 1025, 977, 958, 900, 866, 813, 784, 778, 736, 710, 685, 627, 601, 559, 527, 517, 464, 458, 449 cm^{-1}. MS, *m/z* (%): 760.2 (100, M − H). HRMS (APCI−) *m/z*: $[M]^-$ calcd for $C_{49}H_{44}S_4^-$ 760.2331; found 760.2325. Anal. calcd. for $C_{49}H_{44}S_4$: C, 77.32; H, 5.83. Found: C, 76.93; H, 5.74.

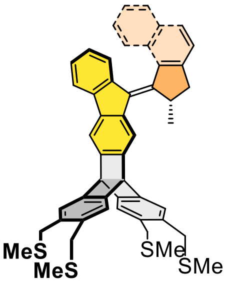

Compound 1B was generated as a metastable transient in 90% yield upon irradiation of compound **1A** at 385±5 nm in $CDCl_3$ and was not isolated (**Section 7**). ^{1}H NMR (500 MHz, $CDCl_3$): δ 7.96-7.94 (m, 1H), 7.93-7.92 (m, 1H), 7.75-7.73 (m, 1H), 7.72 (s, 1H), 7.70-7.68 (m, 1H), 7.51-7.48 (m, 2H), 7.39-7.36 (m, 1H), 7.26 (s, 1H), 7.26-7.24 (m, 2H), 7.19 (s, 1H), 6.94 (s, 1H), 6.91 (s, 1H), 6.91-6.87 (m, 1H), 6.55 (s, 1H), 5.38 (s, 1H), 4.66 (s, 1H), 4.23-4.19 (m, 1H), 3.89-3.84 (m, 2H), 3.64-3.55 (m, 7H), 3.17-3.12 (m, 1H), 2.08 (s, 3H), 2.02 (s, 3H), 1.94 (s, 3H), 1.87 (s, 3H), 1.51 (d, *J* = 6.5 Hz, 3H).

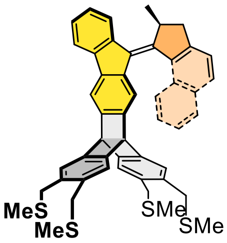

Compound 1C. Mp 190-195 °C (dec.). ^{1}H NMR (600 MHz, $CDCl_3$): δ 8.07-8.05 (m, 1H), 8.02-8.00 (m, 1H), 7.93-7.91 (m, 1H), 7.76-7.75 (m, 1H), 7.74 (s, 1H), 7.64-7.62 (m, 1H), 7.59-7.58 (m, 1H), 7.57-7.54 (m, 1H), 7.36-7.31 (m, 2H), 7.32 (s, 1H), 7.24 (s, 1H), 7.13-7.10 (m, 1H), 7.06 (s, 1H), 6.97 (s, 1H), 6.60 (s, 1H), 5.38 (s, 1H), 4.57 (s, 1H), 4.31 (p, *J* = 6.6 Hz, 1H), 3.91 (d, *J* = 23.7 Hz, 1H), 3.87 (d, *J* = 23.7 Hz, 1H), 3.74 (d, *J* = 4.2 Hz, 1H), 3.72 (d, *J* = 4.2 Hz, 1H), 3.68 (br s, 2H), 3.61 (br s, 2H), 3.54 (dd, J_1 = 5.7 Hz, J_2 = 15.0 Hz, 1H), 2.78 (d, *J* = 15.0 Hz, 1H), 2.16 (s, 3H), 2.09 (s, 3H),

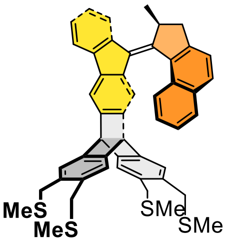

1.97 (s, 3H), 1.90 (s, 3H), 1.38 (d, J = 6.6 Hz, 3H). ^{13}C {^{1}H} NMR (150 MHz, $CDCl_3$): δ 150.6, 147.4, 144.2, 143.9, 143.78, 143.72, 142.7, 140.1, 139.9, 137.1, 136.3, 134.3, 132.83, 132.81, 132.77, 132.73, 132.6, 130.8, 130.2, 129.7, 128.7, 127.8, 126.8, 126.39, 126.37, 125.6, 125.48, 125.41, 125.3, 125.2, 124.0, 123.9, 121.6, 119.1, 114.3, 53.27, 53.24, 44.9, 41.9, 35.6, 35.43, 35.37, 35.31, 19.2, 15.9, 15.66, 15.63, 15.61. IR (KBr): 3048, 3007, 2959, 2911, 2843, 1609, 1579, 1561, 1514, 1468, 1445, 1429, 1341, 1316, 1300, 1234, 1211, 1179, 1055, 1023, 979, 958, 901, 867, 813, 784, 776, 735, 706, 627, 597, 559, 535, 460 cm^{-1}. MS, *m/z* (%): 760.2 (100, M − H). HRMS (APCI−) *m/z*: $[M]^-$ calcd for $C_{49}H_{44}S_4^-$ 760.2331; found 760.2327. Anal. calcd. for $C_{49}H_{44}S_4$: C, 77.32; H, 5.83. Found: C, 77.25; H, 5.38.

Compound 1D was generated as a metastable transient in 89% yield upon irradiation of compound **1C** at 385±5 nm in $CDCl_3$ and was not isolated (Section 7).

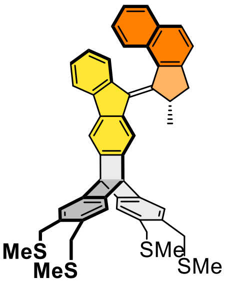

^{1}H NMR (500 MHz, $CDCl_3$): δ 7.86-7.84 (m, 1H), 7.83-7.81 (m, 1H), 7.79 (s, 1H), 7.77 (s, 1H), 7.64-7.62 (m, 1H), 7.57-7.55 (m, 1H), 7.46-7.44 (m, 1H), 7.34-7.31 (m, 1H), 7.30 (s, 1H), 7.29 (s, 1H), 7.27 (s, 1H), 7.21 (s, 1H), 7.13-7.10 (m, 1H), 7.08-7.05 (m, 1H), 6.74-6.71 (m, 1H), 6.58-6.55 (m, 1H), 5.45 (s, 1H), 5.41 (s, 1H), 4.27-4.25 (m, 1H), 3.78-3.66 (m, 9H), 3.18-3.14 (m, 1H), 2.03 (s, 3H), 2.02 (s, 3H), 1.99 (s, 3H), 1.95 (s, 3H), 1.53 (d, J = 6.4 Hz, 3H).

Compound 2. To a solution of thioketone **24** (213 mg, 1.00 mmol, 4.2 equiv) in anhydrous CH_2Cl_2 (10 mL), a solution of diazo compound **23** (145 mg, 0.238 mmol, 1.0 equiv) in CH_2Cl_2 (5 mL) was added dropwise at room temperature and the reaction mixture was further stirred for 15 h. Afterwards, it was adsorbed on silica gel, and flash column chromatography (hexane/CH_2Cl_2 = 1:1, R_f = 0.3) gave episulfide (84 mg, 0.106 mmol) intermediate (HRMS (APCI+) *m/z*: $[M + H]^+$ calcd for $C_{49}H_{45}S_5^+$ 793.2119; found 793.2105.), which was immediately used for the subsequent reaction.

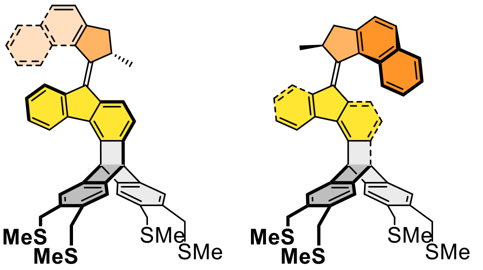

To a 50 mL round bottom flask containing episulfide (84 mg, 0.106 mmol, 1 equiv.) was added anhydrous toluene (15 mL). The mixture was purged with nitrogen, *N*,*N*,*N*′,*N*′,*N*″,*N*″-hexamethylphosphanetriamine (HMPT, 150 μL, 0.827 mmol, ~8 equiv.) was added, and the reaction mixture was stirred for 13 h at 80 °C. Afterwards, volatiles were removed on rotary evaporator, and the residual orange solids were adsorbed on silica gel in CH_2Cl_2. Flash column chromatography (hexane/CH_2Cl_2 = 1:2, R_f = 0.6, yellow spot) afforded orange solid whose further purification by preparative HPLC on silica gel (heptane/CH_2Cl_2 = 1:1) gave a 1:1 mixture **2A** and **2C** as an orange solid (27 mg, 0.036 mmol, 15% yield related to ketone **19**). Partial separation of the isomers was achieved by preparative HPLC on silica gel, yielding an A:C ratio of 4:1 for isomer A and 1:3 for isomer C (15% ethyl acetate in hexane, **Figure S2**, **Section 4**).

2A, **2C**: ^{1}H NMR (400 MHz, $CDCl_3$): δ 8.54 (dd, J_1 = 1.1 Hz, J_2 = 7.9 Hz, 1H), 8.45 (td, J_1 = 1.0 Hz, J_2 = 8.1 Hz, 1H), 8.02 (d, J = 7.9 Hz, 1H), 7.93-7.87 (m, 4H), 7.69 (d, J = 7.9 Hz, 1H), 7.69-7.66 (m, 2H), 7.57-7.53 (m, 3H), 7.47 (s, 1H), 7.46-7.41 (m, 6H), 7.38-7.34 (m, 4H), 7.30 (s, 1H), 7.30-7.25 (m, 2H), 7.20 (s, 1H), 6.85-6.78 (m, 2H), 6.78 (d, J = 7.9 Hz, 1H), 6.48 (d, J = 7.9 Hz, 1H), 6.47 (s, 1H), 6.42 (s, 1H), 5.48 (s, 1H), 5.27 (s, 1H), 4.32-4.22 (m, 2H), 3.86-3.65 (m, 16H), 3.53-3.44 (m, 2H), 2.71 (d, J = 15.1 Hz, 1H), 2.70 (d, J = 15.0 Hz, 1H), 2.10 (s, 3H), 2.10 (s, 3H), 2.07 (s, 3H), 2.06 (s, 3H), 2.06 (s, 3H), 2.03 (s, 3H), 2.00 (s, 3H), 1.95 (s, 3H), 1.35 (d, J = 6.7 Hz, 3H), 1.30 (d, J = 6.7 Hz, 3H). ^{13}C {^{1}H}

NMR (100 MHz, $CDCl_3$): δ 150.21, 150.15, 147.3, 147.2, 144.8, 144.6, 144.5, 144.4, 144.3, 143.69, 143.66, 143.6, 143.5, 140.9, 140.0, 139.41, 139.36, 138.5, 138.3, 138.0, 136.50, 136.48, 135.1, 134.0, 133.5, 133.3, 133.2, 133.12, 133.10, 133.07, 133.03, 132.99, 132.8, 132.54, 132.47, 130.70, 130.66, 130.3, 130.2, 129.78, 129.76, 128.63, 128.59, 127.5, 127.4, 126.9, 126.7, 126.6, 126.5, 126.0, 125.94, 125.88, 125.80, 125.77, 125.74, 125.66, 125.6, 125.4, 125.21, 125.18, 124.3, 124.0, 122.33, 122.29, 122.1, 121.7, 121.5, 120.7, 53.4, 53.3, 49.4, 49.3, 46.1, 45.7, 42.10, 42.06, 35.65, 35.58, 35.53, 35.51, 35.49, 35.42, 35.39, 19.4, 19.0, 16.00, 15.95, 15.81, 15.78, 15.6. IR (KBr): 3436, 3046, 3007, 2960, 2911, 2844, 1618, 1604, 1578, 1560, 1514, 1467, 1421, 1348, 1316, 1235, 1210, 1190, 1149, 1054, 1026, 979, 957, 907, 812, 784, 733, 709, 625 cm^{-1}. MS, *m/z* (%): 759.2 (100, M − H). HRMS (APCI−) *m/z*: $[M-H]^-$ calcd for $C_{49}H_{43}S_4^-$ 759.2253; found 759.2255.

2A: ^{1}H NMR (400 MHz, $CDCl_3$): δ 8.43 (td, J_1 = 1.0 Hz, J_2 = 8.0 Hz, 1H), 7.93-7.87 (m, 2H), 7.68-7.65 (m, 2H), 7.55 (d, J = 8.1 Hz, 1H), 7.45-7.39 (m, 2H), 7.42 (s, 1H), 7.41 (s, 1H), 7.37-7.33 (m, 1H), 7.36 (s, 1H), 7.34 (s, 1H), 7.29-7.24 (m, 1H), 6.84-6.77 (m, 2H), 6.45 (s, 1H), 5.47 (s, 1H), 4.24 (p, J = 6.3 Hz, 1H), 3.81-3.71 (m, 8H), 3.50 (dd, J_1 = 5.3 Hz, J_2 = 15.2 Hz, 1H), 2.71 (d, J = 15.1 Hz, 1H), 2.07 (s, 3H), 2.05 (s, 3H), 2.05 (s, 3H), 2.02 (s, 3H), 1.30 (d, J = 6.4 Hz, 3H).

2C: ^{1}H NMR (400 MHz, $CDCl_3$): δ 8.53 (dd, J_1 = 1.1 Hz, J_2 = 7.8 Hz, 1H), 8.01 (d, J = 7.7 Hz, 1H), 7.93-7.87 (m, 2H), 7.66 (d, J = 8.5 Hz, 1H), 7.56-7.51 (m, 2H), 7.46 (s, 1H), 7.45-7.40 (m, 2H), 7.32 (s, 1H), 7.29 (s, 1H), 7.27-7.25 (m, 1H), 7.19 (s, 1H), 6.77 (d, J = 7.9 Hz, 1H), 6.46 (d, J = 7.9 Hz, 1H), 6.41 (s, 1H), 5.26 (s, 1H), 4.28 (p, J = 6.3 Hz, 1H), 3.85-3.64 (m, 8H), 3.46 (dd, J_1 = 5.5 Hz, J_2 = 15.0 Hz, 1H), 2.70 (d, J = 15.0 Hz, 1H), 2.10 (s, 3H), 2.09 (s, 3H), 1.99 (s, 3H), 1.94 (s, 3H), 1.34 (d, J = 6.7 Hz, 3H).

2,3,6,7-Tetramethylanthracene (**4**).[36] A 1 L three-necked flask, equipped with a magnetic stirring bar, was charged with *o*-xylene (100 mL, 87.9 g, 0.828 mol, 1 equiv.) and CH_2Cl_2 (100 mL, 132.5 g, 1.56 mol, 1.9 equiv.). This mixture was purged with nitrogen and cooled using ice-NaCl bath. Anhydrous $AlCl_3$ (80.0 g, 0.600 mol, 72 mol%) was then slowly added under a nitrogen atmosphere. The cooling bath was removed, upon reaching room temperature the nitrogen flow was stopped, and the reaction flask was fitted with a reflux condenser. The color of the reaction mixture gradually changed to dark red, and the mixture started to reflux spontaneously. When the spontaneous reflux stopped (approx. 1 h), additional CH_2Cl_2 (100 mL) was added, and the mixture was heated under reflux for another hour using an oil bath. After cooling to 0 °C using ice-water bath, CH_2Cl_2 (150 mL) was added, and the reaction mixture was carefully quenched, first with ice and then with water (note: the quenching was very exothermic). All material from the reaction flask was transferred to a frit using water and CH_2Cl_2 and the remaining solids were washed with water, MeOH and CH_2Cl_2. Product **4** was obtained as a light beige powder on the frit (17.7 g, 75.5 mmol, 18%).

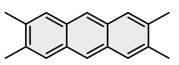

Mp 290-292 °C. ^{1}H NMR (400 MHz, $CDCl_3$): δ 8.15 (s, 2H), 7.70 (q, J = 0.9 Hz, 4H), 2.45 (d, J = 0.8 Hz, 12H). ^{13}C {^{1}H} NMR (100 MHz, $CDCl_3$): δ 134.9, 130.8, 126.9, 123.4, 20.4. IR (KBr): 3004, 2979, 2935, 2913, 2883, 2843, 2720, 1813, 1754, 1690, 1641, 1529, 1466, 1457, 1414, 1378, 1289, 1203, 1105, 1025, 999, 906, 884, 872, 837, 795, 476, 411 cm^{-1}. MS, *m/z* (%): 234.1 (100, M), 219.1 (40, M − CH_3), 203.1 (15). HRMS (EI) *m/z*: $[M]^+$ calcd for $C_{18}H_{18}^+$ 234.1403; found 234.1404. Anal. calcd. for $C_{18}H_{18}$: C, 92.26; H, 7.74. Found: C, 92.10; H, 7.56.

[36] Y.-H. Xiao, Y. Shao, X.-X. Ye, H. Cui, D.-L. Wang, X.-H. Zhou, S.-L. Sun, L. Cheng, "Microporous Aromatic Polyimides Derived from Triptycene-Based Dianhydride" *Chin. Chem. Lett.* **2016**, *27* (3), 454.

1,2,6,7-Tetramethylanthracene (**5**) was obtained as a side product during synthesis of **4** and was isolated from CH_2Cl_2 washes as a beige powder (11.5 g, 49.1 mmol, 12%).

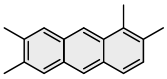

Mp 164-166 °C. ^{1}H NMR (400 MHz, $CDCl_3$): δ 8.43 (s, 1H), 8.23 (s, 1H), 7.79 (s, 1H), 7.76 (d, *J* = 8.6 Hz, 1H), 7.72 (s, 1H), 7.25 (d, *J* = 8.6 Hz, 1H), 2.71 (s, 3H), 2.52 (s, 3H), 2.48 (s, 3H), 2.47 (s, 3H). ^{13}C {^{1}H} NMR (100 MHz, $CDCl_3$): δ 135.3, 135.1, 131.5, 131.3, 131.1, 130.5, 130.2, 130.1, 128.7, 127.4, 126.6, 125.9, 125.2, 120.7, 20.7, 20.42, 20.38, 14.8. IR (KBr): 3440, 3042, 3002, 2965, 2941, 2913, 2855, 1779, 1640, 1621, 1535, 1461, 1445, 1381, 1373, 1338, 1322, 1198, 1175, 1025, 1002, 892, 788, 764, 613, 566, 473 cm^{-1}. MS, *m/z* (%): 234.1 (100, M), 219.1 (40, M − CH_3), 203.1 (15). HRMS (EI) *m/z*: $[M]^+$ calcd for $C_{18}H_{18}^+$ 234.1403; found 234.1401. Anal. calcd. for $C_{18}H_{18}$: C, 92.26; H, 7.74. Found: C, 92.27; H, 8.00.

General Procedure for Diels-Alder Reaction (GP1)

A three-necked flask equipped with a reflux condenser was charged with 2,3,6,7-tetramethylanthracene (**4**), suspended in either 1,4-dioxane (30 mL) or a mixture of 1,4-dioxane/1,2-dichloroethane (60 mL), and heated to reflux. Then isoamyl nitrite (0.5-2.0 equiv.) was added followed by dropwise addition of an anthranilic acid **6** or **7** (0.5-2.0 equiv.) in 1,4-dioxane (15-30 mL) and refluxed for 20-30 min. This process was repeated 2-4 times. Then volatiles were removed using rotary evaporator, remaining solids were washed by MeOH and CH_2Cl_2 and finally purified by flash column chromatography.

Compound 8 was prepared by reacting anthracene **4** (10.0 g, 42.7 mmol, 1 equiv.) in 1,4-dioxane (60 mL) with anthranilic acid **6** (12.0 g, 43.8 mmol, 1.03 equiv.) and isoamyl nitrite (6 mL, 44.8 mmol, 1.05 equiv.) added in two equal portions according to **GP1**. Volatiles were removed using rotary evaporator and the solids were washed with MeOH (100 mL) and CH_2Cl_2 (100 mL). The MeOH phase containing product was evaporated and subjected to column chromatography on silica gel (hexane/CH_2Cl_2 = 2:1, R_f = 0.1) affording a 3:1 mixture (3.73 g, 8.34 mmol) of triptycene **8** (~2.91 g, ~6.50 mmol, ~15%) and regioisomer **S1** (~0.821 g, ~1.84 mmol, ~4%) as a white powder. This mixture was usually used for the next steps. An analytical sample of triptycene **8** was obtained using preparative HPLC (30% to 90% CH_2Cl_2 in hexane).

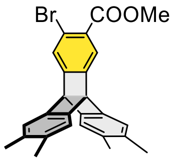

Mp 126-131 °C. ^{1}H NMR (400 MHz, $CDCl_3$): δ 7.78 (s, 1H), 7.62 (s, 1H), 7.16 (s, 2H), 7.15 (s, 2H), 5.28 (s, 1H), 5.26 (s, 1H), 3.87 (s, 3H), 2.15 (s, 12H). ^{13}C {^{1}H} NMR (100 MHz, $CDCl_3$): δ 166.4, 151.1, 145.4, 142.2, 141.6, 133.5, 133.4, 129.3, 127.8, 126.0, 125.3, 125.2, 118.6, 52.6, 52.5, 52.3, 19.5. IR (KBr): 3006, 2951, 2920, 2856, 1733, 1707, 1624, 1603, 1559, 1471, 1458, 1433, 1386, 1325, 1274, 1243, 1191, 1128, 1105, 1053, 1018, 995, 932, 886, 873, 820, 803, 781, 625, 610, 534 cm^{-1}. MS, *m/z* (%): 469.1 (100, M + Na). HRMS (ESI+) *m/z*: $[M + H]^+$ calcd for $C_{26}H_{24}O_2Br^+$ 447.0954; found 447.0953. Anal. calcd. for $C_{26}H_{23}BrO_2$: C, 69.80; H, 5.18. Found: C, 68.46; H, 5.05.

Compound 9.[47] Anthracene **4** (5.00 g, 21.3 mmol, 1 equiv.) was treated according to **GP1** with 1,4-dioxane/1,2-dichloroethane (20/100 mL). Isoamyl nitrite (5.80 mL, 43.3 mmol, 2.0 equiv.) and anthranilic acid **7** (7.13 g, 42.7 mmol, 2.0 equiv.) were added four times with 20 min reflux in between with occasional addition of isoamyl nitrite excess. The volatiles were evaporated on rotary evaporator

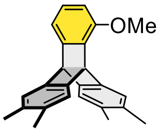

[47] M. Rybáčková, M. Bělohradský, P. Holý, R. Pohl, V. Dekoj, J. Závada, "Synthesis of Highly Symmetrical Triptycene Tetra- and Hexacarboxylates" *Synthesis* **2007**, *2007* (10), 1554-1558.

and the solid residue was triturated with MeOH (50 mL) to remove majority of impurities including regioisomer **S2**. The remaining solids were washed with CH_2Cl_2 (3 × 20 mL) leaving a clean solid anthracene **4** (970 mg, 4.14 mmol). The combined CH_2Cl_2 washes containing crude **9** were sorbed onto silica gel and purified by flash column chromatography (hexane/CH_2Cl_2 = 4:1, R_f = 0.3) yielding **9** (2.28 g, 6.70 mmol, 31%) as a yellowish powder. The byproduct **S2** (0.436 g, 1.28 mmol, ~6%) was isolated from methanol washes using preparative HPLC (hexane/CH_2Cl_2 = 5:1).

Mp 264-266 °C. ^{1}H NMR (400 MHz, $CDCl_3$): δ 7.21 (s, 2H), 7.17 (s, 2H), 7.02-7.00 (m, 1H), 6.92 (dd, J_1 = 7.3 Hz, J_2 = 8.3 Hz, 1H), 6.57 (dd, J_1 = 1.0 Hz, J_2 = 8.2 Hz, 1H), 5.78 (s, 1H), 5.29 (s, 1H), 3.85 (s, 3H), 2.16 (s, 12H). ^{13}C {^{1}H} NMR (100 MHz, $CDCl_3$): δ 154.2, 148.0, 143.7, 143.2, 133.4, 132.6, 132.5, 125.8, 125.0, 124.9, 116.2, 108.3, 55.6, 53.3, 45.9, 19.44, 19.43. IR (KBr): 2999, 2973, 2932, 2921, 2855, 2837, 1601, 1592, 1481, 1471, 1441, 1405, 1379, 1312, 1265, 1255, 1187, 1076, 1069, 1050, 1016, 996, 954, 884, 880, 868, 856, 825, 814, 763, 756, 724, 647, 637, 589, 563, 463, 440 cm^{-1}. MS, *m/z* (%): 379.1 (7), 363.2 (53, M + Na), 358.2 (71), 341.2 (100, M + H). HRMS (ESI+) *m/z*: [M + H]$^+$ calcd for $C_{25}H_{25}O^+$ 341.1900; found 341.1897. Anal. calcd. for $C_{25}H_{24}O$: C, 88.20; H, 7.11. Found: C, 88.46; H, 7.15.

Compound 10. A 500 mL three-necked flask, equipped with a magnetic stirring bar, was charged with triptycene **9** (2.20 g, 6.46 mmol, 1 equiv.) The flask was purged with nitrogen and the compound was dissolved in anhydrous CH_2Cl_2 (140 mL). After cooling to 0 °C in an ice-water bath, BBr_3 (1.90 mL, 19.4 mmol, 3 equiv.) was added dropwise to the stirring solution. After 5 min, the cooling bath was removed, and the reaction mixture was stirred for 16 h at room temperature. The reaction mixture was then cooled again to 0 °C using an ice-water bath and carefully quenched with ice (200 g). The reaction mixture was transferred to separatory funnel, an additional portion of CH_2Cl_2 (60 mL) was added, and organic phase was separated. The aqueous phase was extracted with an additional portion of CH_2Cl_2 (60 mL). The combined organic parts were washed with a saturated aqueous solution of $NaHCO_3$ (100 mL), and the aqueous phase was extracted with CH_2Cl_2 (60 mL). The combined organic phases were dried over Na_2SO_4, and volatiles were removed using a rotary evaporator. The crude phenol **10** (2.10 g, 6.46 mmol, 100%) was immediately used in the next step.

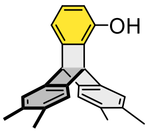

^{1}H NMR (400 MHz, $CDCl_3$): δ 7.19 (s, 2H), 7.15 (s, 2H), 6.97-6.95 (m, 1H), 6.79 (dd, J_1 = 7.3 Hz, J_2 = 8.1 Hz, 1H), 6.41 (dd, J_1 = 1.0 Hz, J_2 = 8.1 Hz, 1H), 5.67 (s, 1H), 5.26 (s, 1H), 4.67 (s, 1H), 2.14 (s, 12H). ^{13}C {^{1}H} NMR (100 MHz, $CDCl_3$): δ 149.8, 148.5, 143.4, 142.9, 132.75, 132.71, 131.4, 125.8, 125.0, 124.9, 116.4, 112.8, 53.3, 46.0, 19.5, 19.4. MS, *m/z* (%): 349.2 (91, M + Na), 327.2 (100, M + H). HRMS (ESI+) *m/z*: [M + H]$^+$ calcd for $C_{24}H_{23}O^+$ 327.1743; found 327.1747.

Compound 11. A 250 mL round-bottom flask containing phenol **10** (2.10 g, 6.46 mmol, 1 equiv.) was purged with nitrogen followed by addition of anhydrous CH_2Cl_2 (90 mL). The stirring solution was cooled to -78 °C (acetone and dry ice) and Et_3N (1.00 mL, 7.23 mmol, 1.1 equiv.) was added. The reaction mixture was stirred for 30 min, then Tf_2O (1.20 mL, 7.23 mmol, 1.1 equiv.) was added dropwise. The reaction mixture was stirred for an additional 30 min, then the cooling bath was removed and after 1.5 h, the reaction was quenched with a saturated aqueous solution of $NaHCO_3$ (100 mL). Subsequently, the layers were separated, and the aqueous phase was extracted with CH_2Cl_2 (2 × 50 mL). The combined organic phases were dried over Na_2SO_4, and volatiles were removed using a rotary evaporator. Flash column

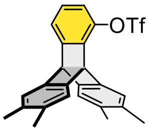

chromatography on silica gel (hexane/CH_2Cl_2 = 4:1, R_f = 0.4) afforded triflate **11** (2.83 g, 6.20 mmol, 96%) as a white powder.

Mp 90-92 °C. ^{1}H NMR (400 MHz, $CDCl_3$): δ 7.36 (d, *J* = 7.1 Hz, 1H),7.25 (s, 2H), 7.21 (s, 2H), 7.03 (dd, J_1 = 7.3 Hz, J_2 = 8.4 Hz, 1H), 6.92 (d, *J* = 8.3 Hz, 1H), 5.67 (s, 1H), 5.38 (s, 1H), 2.192 (s, 6H), 2.186 (s, 6H). ^{19}F NMR (377 MHz, $CDCl_3$): δ -73.13 (s, 3F). ^{13}C {^{1}H} NMR (100 MHz, $CDCl_3$): δ 150.0, 144.4, 142.6, 141.3, 138.2, 133.5, 133.4, 126.6, 125.6, 125.0, 123.1, 118.8 (q, *J* = 321 Hz), 117.8, 53.0, 47.3, 19.49, 19.48. IR (KBr): 3009, 2959, 2923, 2859, 1614, 1577, 1474, 1463, 1447, 1422, 1386, 1251, 1213, 1173, 1147, 1137, 1019, 996, 974, 896, 882, 838, 830, 821, 813, 771, 753, 663, 613, 572, 525 cm^{-1}. MS, *m/z* (%): 458.1 (76, M), 443.1 (14, M − CH_3), 325.2 (71), 310.1 (100, M − OTf), 267.1 (21). HRMS (EI) *m/z*: $[M]^+$ calcd for $C_{25}H_{21}F_3O_3S^+$ 458.1158; found 458.1160. Anal. calcd. for $C_{25}H_{21}F_3O_3S$: C, 65.49; H, 4.62. Found: C, 65.30; H, 4.45.

General Procedure for Suzuki Coupling (GP2)

A two-necked flask was charged with either triptycene **8** or **11** (1 equiv.), boronic acid **12** (1.4 equiv.) or **14** (1.1 equiv.), respectively, Pd(dppf)Cl_2 (6-8 mol%) and K_3PO_4 (4-5 equiv). The flask was then purged with nitrogen and anhydrous 1,4-dioxane (35-100 mL) was added. Then the reaction mixture was heated to gentle reflux for 16 h until complete conversion according to ^{1}H NMR.

Compound 13 was prepared from bromide **8** (2.91 g, 6.50 mmol, 1 equiv.), boronic acid **12** (1.12 g, 9.17 mmol, 1.4 equiv.), Pd(dppf)Cl_2 (305 mg, 0.417 mmol, ~6 mol%), and K_3PO_4 (7.09 g, 33.4 mmol, ~5 equiv.) in 1,4-dioxane (35 mL) according to **GP2**. The volatiles were evaporated using rotary evaporator and the remaining solids were washed with MeOH (10 mL). Ester **13** (2.00 g, 4.50 mmol, 69%) was obtained as a white powder.

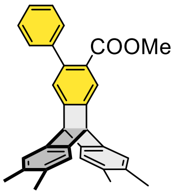

Mp 304-308 °C. ^{1}H NMR (400 MHz, $CDCl_3$): δ 7.81 (s, 1H), 7.35-7.27 (m, 5H), 7.19 (s, 2H), 7.18 (s, 1H), 7.17 (s, 2H), 5.36 (s, 1H), 5.32 (s, 1H), 3.54 (s, 3H), 2.16 (s, 6H), 2.15 (s, 6H). ^{13}C {^{1}H} NMR (100 MHz, $CDCl_3$): δ 168.8, 149.2, 144.9, 142.6, 142.2, 141.8, 140.4, 133.3, 133.2, 128.4, 127.8, 127.0, 126.9, 126.0, 125.2, 125.1, 124.6, 53.1, 52.7, 51.7, 19.5. IR (KBr): 3379, 3027, 3006, 2945, 2917, 2857, 2726, 1753, 1699, 1657, 1624, 1611, 1600, 1578, 1560, 1494, 1469, 1447, 1430, 1403, 1383, 1334, 1313, 1277, 1235, 1189, 1158, 1137, 1085, 1053, 1026, 990, 943, 924, 907, 885, 875, 869, 844, 825, 809, 789, 768, 737, 701, 668, 647, 632, 619, 614, 600, 581, 535, 514, 506, 451, 430 cm^{-1}. MS, *m/z* (%): 467.2 (100, M + Na). HRMS (ESI+) *m/z*: $[M + H]^+$ calcd for $C_{32}H_{29}O_2^+$ 445.2162; found 445.2162. Anal. calcd. for $C_{32}H_{28}O_2$: C, 86.45; H, 6.35. Found: C, 86.23; H, 6.29.

Compound 15 was prepared from triflate **11** (1.51 g, 3.28 mmol, 1 equiv.), boronic acid **14** (650 mg, 3.61 mmol, 1.1 equiv.), Pd(dppf)Cl_2 (183 mg, 0.250 mmol, ~8 mol%) and K_3PO_4 (2.79 g, 13.1 mmol, 4 equiv.) in 1,4-dioxane (100 mL) according to **GP2**. The volatiles were removed using a rotary evaporator and the residue was sorbed onto silica gel in CH_2Cl_2. Flash column chromatography on silica gel (hexane/CH_2Cl_2 = 1:1, R_f = 0.42) gave ester **15** as a 1:1 mixture of two atropisomers *R*-**15** and *S*-**15** (1.01 g, 2.27 mmol, 69%) in form of a white powder.

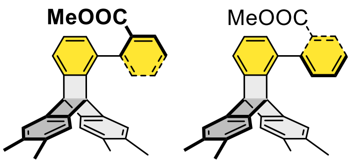

Mp 218-220 °C. ^{1}H NMR (400 MHz, $CDCl_3$): δ 8.08 (dd, J_1 = 1.5 Hz, J_2 = 7.7 Hz, 1H), 7.62 (dt, J_1 = 1.5 Hz, J_2 = 7.5 Hz, 1H), 7.55 (dt, J_1 = 1.4 Hz, J_2 = 7.6 Hz, 1H), 7.33 (dd, J_1 = 1.4 Hz, J_2 = 7.3 Hz, 1H), 7.26 (dd, J_1 = 1.4 Hz, J_2 = 7.5 Hz, 1H), 7.17 (s, 1H), 7.15 (s, 1H), 6.97 (t, *J* = 7.5 Hz, 1H), 6.94 (s, 1H), 6.87 (s, 1H), 6.82 (dd, J_1 = 1.2 Hz, J_2 = 7.6 Hz, 1H), 5.32 (s, 1H), 4.97 (s, 1H), 3.07 (s, 3H), 2.14 (s, 3H), 2.14 (s, 3H), 2.13 (s, 3H), 2.10 (s, 3H). ^{13}C

{^{1}H} NMR (100 MHz, $CDCl_3$): δ 167.9, 145.4, 143.7, 143.2, 143.1, 142.7, 141.5, 136.8, 132.7, 132.6, 132.5, 132.3, 131.6, 131.2, 131.1, 130.1, 127.4, 125.3, 125.1, 124.9, 124.7, 124.1, 122.3, 53.4, 51.4, 50.0, 19.5, 19.45, 19.41, 19.36. IR (KBr): 3056, 3003, 2982, 2947, 2919, 2857, 1713, 1595, 1474, 1461, 1445, 1432, 1425, 1284, 1257, 1241, 1188, 1159, 1143, 1123, 1109, 1074, 1052, 1039, 1015, 995, 953, 888, 881, 872, 844, 835, 820, 783, 760, 739, 733, 722, 712, 645, 636, 621, 609, 589, 584, 554, 475, 440 cm^{-1}. MS, *m/z* (%): 467.2 (100, M + Na), 445.2 (9, M + H), 413.2 (29). HRMS (ESI+) *m/z*: $[M + H]^+$ calcd for $C_{32}H_{29}O_2^+$ 445.2162; found 445.2159. Anal. calcd. for $C_{32}H_{28}O_2$: C, 86.45; H, 6.35. Found: C, 85.64; H, 6.63.

General Procedure for Cyclization Reaction (GP3)[48]

Esters **13** or **15** (~500 mg) were suspended in $BF_3{\cdot}Et_2O$ (~4 mL) and transferred into 5 mL microwave reactors. The mixtures were heated in a microwave reactor at 150 °C for 2-6 minutes. [*Note: Higher substrate loading results in significantly longer reaction times and more complex mixtures.*] Reaction progress was monitored every 2 minutes by ^{1}H NMR (aliquots were diluted with 500 µL water, extracted with $CDCl_3$ (3 × 300 µL), and dried over $MgSO_4$). Upon completion, reactions were quenched in a separatory funnel containing ~100 mL water and extracted with CH_2Cl_2 (3 × 60 mL). Fluorenones **16** and **17** were purified by silica gel column chromatography.

Compound 16 was synthesized from ester **13** (2.00 g, 4.49 mmol, 1 equiv.) in $BF_3{\cdot}Et_2O$ (16 mL) according to **GP3**. This reaction mixture was equally divided in four microwave tubes and each of them was heated for 2 min at 150 °C. Column chromatography on silica gel (hexane/CH_2Cl_2 = 1:1, R_f = 0.3) yielded fluorenone **16** (1.84 g, 4.46 mmol, 99%) as an orange powder.

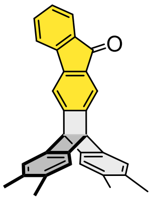

Mp 313-315 °C (dec.). ^{1}H NMR (400 MHz, $CDCl_3$): δ 7.58 (s, 1H), 7.54-7.52 (m, 1H), 7.49 (s, 1H), 7.42-7.37 (m, 2H), 7.21-7.17 (m, 1H), 7.18 (s, 4H), 5.35 (s, 1H), 5.32 (s, 1H), 2.16 (s, 12H). ^{13}C {^{1}H} NMR (100 MHz, $CDCl_3$): δ 193.7, 153.7, 147.5, 144.3, 143.1, 142.3, 141.6, 134.7, 134.4, 133.5, 133.3, 131.4, 128.5, 125.1, 125.0, 123.9, 119.6, 119.3, 116.0, 53.9, 53.2, 19.5. IR (KBr): 3407, 3051, 3002, 2954, 2919, 2855, 2729, 1713, 1650, 1611, 1602, 1493, 1471, 1448, 1431, 1383, 1370, 1360, 1294, 1286, 1269, 1257, 1227, 1190, 1175, 1159, 1140, 1095, 1067, 1054, 1018, 995, 984, 952, 908, 883, 856, 818, 803, 795, 766, 735, 719, 708, 684, 657, 649, 635, 628, 610, 604, 544, 520, 511, 485, 451, 435. MS, *m/z* (%): 413.2 (100, M + H). HRMS (APCI+) *m/z*: $[M + H]^+$ calcd for $C_{31}H_{25}O^+$ 413.1899; found 413.1899. Anal. calcd. for $C_{31}H_{24}O{+}CH_2Cl_2$: C, 77.26; H, 5.27. Found: C, 77.22; H, 5.03.

Compound 17 was synthesized from ester **15** (983 mg, 2.21 mmol, 1 equiv.) in $BF_3{\cdot}Et_2O$ (10 mL) according to **GP3**. This reaction mixture was equally divided in two microwave tubes and each of them was heated for 6 min at 150 °C. Column chromatography on silica gel (hexane/CH_2Cl_2 = 1:2, R_f = 0.5) yielded fluorenone **17** (762 mg, 1.85 mmol, 84%) as an orange powder.

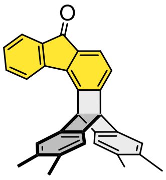

Mp 214-216 °C. ^{1}H NMR (400 MHz, $CDCl_3$): δ 8.16 (td, J_1 = 0.9 Hz, J_2 = 7.7 Hz, 1H), 7.68-7.66 (m, 1H), 7.61 (dt, J_1 = 1.3 Hz, J_2 = 7.6 Hz, 1H), 7.35 (d, J = 7.2 Hz, 1H), 7.32 (dt, J_1 = 1.0 Hz, J_2 = 7.5 Hz, 1H), 7.25 (s, 2H), 7.24 (d, J = 7.1 Hz, 1H), 7.20 (s, 2H), 6.00 (s, 1H), 5.33 (s, 1H), 2.17 (s, 12H). ^{13}C {^{1}H} NMR (100 MHz, $CDCl_3$): δ 193.0, 153.8, 144.8, 141.8, 141.4, 140.9, 136.8, 135.3, 134.4, 133.7, 133.6, 131.8, 128.6, 125.24, 125.19, 124.4, 123.7, 122.7, 122.1, 53.6, 49.2, 19.51, 19.49. IR (KBr): 3399, 2956, 2916, 2854, 1709, 1610, 1584, 1475, 1470, 1424, 1381, 1310, 1291, 1260, 1205, 1172, 1018,

[48] P. Kancharla, R. A. Dodean, Y. Li, J. X. Kelly, "Boron Trifluoride Etherate Promoted Microwave-Assisted Synthesis of Antimalarial Acridones" *RSC Advances* **2019**, *9* (72), 42284-42293.

996, 973, 917, 770, 738, 712, 682, 628, 607, 473 cm^{-1}. MS, *m/z* (%): 435.2 (44, M + Na), 413.2 (30, M + H). HRMS (ESI+) *m/z*: $[M + H]^+$ calcd for $C_{31}H_{25}O^+$ 413.1900; found 413.1901. Anal. calcd. for $C_{31}H_{24}O$: C, 90.26; H, 5.86. Found: C, 89.85; H, 6.00.

General Procedure for Installing Anchoring Groups (GP4)
A two-necked round-bottom flask equipped with a reflux condenser containing ketone **16** or **17** (1 equiv.) was charged with NBS (5 equiv.) and benzoyl peroxide (9-10 mol%). The flask was purged with nitrogen after which the solids were dissolved in anhydrous $CHCl_3$, and the reaction flask was refluxed in an oil bath during which the progress was monitored using ^{1}H NMR spectroscopy (solution aliquot was taken, evaporated using a rotary evaporator and redissolved in 500 µL of $CDCl_3$). When over-brominated products were observed, the reaction was terminated, solvent was evaporated and remaining solids containing tetrabromides were used directly in the next step.

A 2-necked round-bottom flask containing the crude tetrabromides was charged with MeSNa (6-8 equiv.). The flask was purged with nitrogen after which anhydrous DMF and toluene (added to remove residual NBS) were then added. The reaction mixture was stirred for 15-40 minutes. All volatiles were evaporated using a rotary evaporator, and the solids were suspended in CH_2Cl_2 and sorbed onto silica gel. Flash column chromatography on silica gel (CH_2Cl_2, R_f = 0.6) followed by preparative HPLC on silica gel (CH_2Cl_2) afforded tetrapods **18** or **19** as an orange solid material.

Compound 18. Ketone **16** (1.82 g, 4.52 mmol, 1 equiv.) was refluxed for 3 days with NBS (4.02 g, 22.6 mmol, 5 equiv.) and benzoyl peroxide (159 mg, 0.492 mmol, 9 mol%) in $CHCl_3$ (250 mL) following **GP4**. Crude tetrabrominated derivative (3.29 g, 4.52 mmol, 1 equiv.), HRMS (APCI+) *m/z*: $[M + H]^+$ calcd for $C_{31}H_{21}OBr_4^+$ 724.8320; found 724.8323, reacted with MeSNa (1.90 g, 27.1 mmol, 6 equiv.) in a mixture of DMF (45 mL) and toluene (1 mL) at room temperature for 15 min according to **GP4**. Tetrapod **18** (310 mg, 0.519 mmol, 12% over two steps) was obtained as an orange powder.

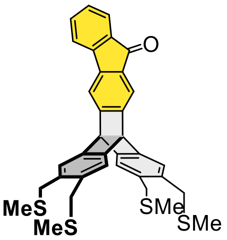

Mp 185-188 °C (dec.). ^{1}H NMR (400 MHz, $CDCl_3$): δ 7.58 (s, 1H), 7.53 (ddd, J_1 = 1.1 Hz, J_2 = 1.1 Hz, J_3 = 7.3 Hz, 1H), 7.50 (s, 1H), 7.40-7.39 (m, 2H), 7.33 (s, 2H), 7.30 (s, 2H), 7.20 (ddd, J_1 = 2.8 Hz, J_2 = 5.7 Hz, J_3 = 7.3 Hz, 1H), 5.42 (s, 1H), 5.38 (s, 1H), 3.76 (s, 4H), 3.75 (s, 4H), 2.04 (s, 6H), 2.03 (s, 6H). ^{13}C {^{1}H} NMR (100 MHz, $CDCl_3$): δ 193.5, 152.6, 146.6, 144.1, 143.3, 142.7, 134.6, 134.4, 133.6, 133.5, 131.6, 128.7, 125.82, 125.78, 124.0, 119.7, 119.5, 116.3, 53.8, 53.2, 35.5, 35.4, 15.83, 15.79. IR (KBr): 2959, 2911, 1709, 1609, 1487, 1468, 1447, 1429, 1316, 1289, 1232, 1189, 1173, 1094, 1066, 984, 958, 911, 767, 737, 512 cm^{-1}. MS, *m/z* (%): 597.1 (34, M + H), 549.1 (100, M − SMe). HRMS (APCI+) *m/z*: $[M + H]^+$ calcd for $C_{35}H_{33}OS_4^+$ 597.1408; found 597.1404. Anal. calcd. for $C_{35}H_{32}OS_4$: C, 70.43; H, 5.40. Found: C, 69.68; H, 5.34.

Compound 19 Ketone **17** (748 mg, 1.81 mmol, 1 equiv.) was refluxed for 3 days with NBS (1.61 g, 9.07 mmol, 5 equiv.) and benzoyl peroxide (59 mg, 0.183 mmol, 10 mol%) in $CHCl_3$ (30 mL) following **GP4**. Crude tetrabrominated derivative (1.32 g, 1.81 mmol, 1 equiv.), HRMS (APCI+) *m/z*: $[M + H]^+$ calcd for $C_{31}H_{21}OBr_4^+$ 724.8320; found 724.8320, reacted with MeSNa (1.02 g, 14.5 mmol, 8 equiv.) in a mixture of DMF (15 mL) and toluene (1 mL) at 60 °C for 40 min according to **GP4**. Tetrapod **19** (149 mg, 0.250 mmol, 14% over two steps) was obtained as an orange powder.

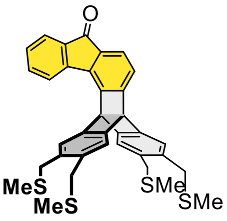

Mp 145-147 °C (dec.). ^{1}H NMR (400 MHz, $CDCl_3$): δ 8.13 (td, J_1 = 0.9 Hz, J_2 = 7.7 Hz, 1H), 7.69-7.67 (m, 1H), 7.62 (dt, J_1 = 1.3 Hz, J_2 = 7.6 Hz, 1H), 7.39 (s, 2H), 7.39 (d, J = 7.3 Hz, 1H), 7.33 (dt, J_1 = 0.9 Hz, J_2 = 7.5 Hz, 1H), 7.33 (s, 2H), 7.27 (d, J = 7.7 Hz, 1H), 6.06 (s, 1H), 5.39 (s, 1H), 3.81-3.72 (m, 8H), 2.05 (s, 6H), 2.04 (s, 6H). ^{13}C {^{1}H} NMR (100 MHz, $CDCl_3$): δ 192.7, 152.7, 144.5, 142.8, 142.4, 140.0, 137.2, 135.2, 134.6, 133.9, 133.7, 132.1, 128.8, 126.0, 125.9, 124.6, 124.0, 122.7, 122.2, 53.5, 49.2, 35.5, 15.9, 15.8. IR (KBr): 3007, 2963, 2911, 2852, 2826, 1708, 1608, 1585, 1468, 1425, 1380, 1310, 1292, 1261, 1235, 1207, 1175, 1090, 974, 920, 769, 735, 691, 622 cm^{-1}. MS, *m/z* (%): 597.1 (83, M + H), 565.1 (13), 549.1 (100, M − SMe). HRMS (APCI+) *m/z*: $[M + H]^+$ calcd for $C_{35}H_{33}OS_4^+$ 597.1409; found 597.1414. Anal. calcd. for $C_{35}H_{32}OS_4$: C, 70.43; H, 5.40. Found: C, 65.99; H, 5.11.

General Procedure for Synthesis of Hydrazones 20 and 21 (GP5)

A solution of ketones **18** or **19** (1 equiv.) either in DMF (1 mL) or THF (2 mL) were added to a round-bottom flask containing anhydrous EtOH (6-17 mL). Subsequently, hydrazine hydrate (28-35 equiv.) was added, and the reaction flasks were refluxed using an oil bath until completion followed by ^{1}H NMR (solution aliquot evaporated on rotary evaporator, redissolved in 500 µL of $CDCl_3$). All volatiles were evaporated using a rotary evaporator, solids were dissolved in CH_2Cl_2 (20 mL) and washed with water (3 × 30 mL). The combined water phases were extracted with CH_2Cl_2 (3 × 30 mL) and combined organic phases were dried over $MgSO_4$. Volatiles were evaporated on rotary evaporator giving hydrazones **20** or **21** as a yellowish solid.

Compound 20 was synthesized from solution of ketone **18** (265 mg, 0.444 mmol, 1 equiv.) in DMF (1 mL) and hydrazine hydrate (600 µL, 12.4 mmol, 28 equiv.) in EtOH (17 mL) according to **GP5**. The reaction mixture was refluxed for 3 h in an oil bath, then volatiles were evaporated on rotary evaporator. The yellowish solid residue was dissolved in CH_2Cl_2 (20 mL) and washed with water (3 × 30 mL). The combined water phases were extracted with CH_2Cl_2 (3 × 30 mL) and combined organic phases were dried over $MgSO_4$. Volatiles were evaporated on rotary evaporator giving hydrazone **20** as a 1:1 mixture of *E*/*Z* isomers (271 mg, 0.444 mmol, 100%) in the form of yellowish solid.

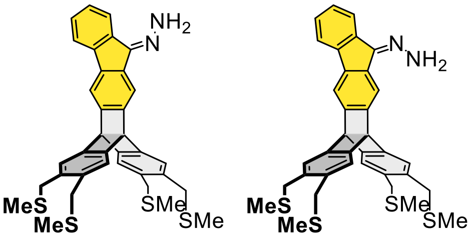

^{1}H NMR (400 MHz, $CDCl_3$): δ 7.94 (s, 1H), 7.82-7.80 (m, 1H), 7.75 (s, 1H), 7.70 (s, 1H), 7.66-7.63 (m, 2H), 7.62 (s, 1H), 7.56-7.54 (m, 1H), 7.39-7.35 (m, 1H), 7.32 (s, 2H), 7.31 (s, 2H), 7.30 (s, 2H), 7.29-7.26 (m, 1H), 7.28 (s, 2H), 7.25-7.20 (m, 2H), 6.34 (s, 4H), 5.44 (s, 1H), 5.40 (s, 1H), 5.39 (s, 1H), 5.37 (s, 1H), 3.75 (s, 12H), 3.740 (s, 2H), 3.735 (s, 2H), 2.033 (s, 6H), 2.030 (s, 6H), 2.028 (s, 6H), 2.01 (s, 6H). MS, *m/z* (%): 633.1 (56, M + Na), 611.2 (100, M + H). HRMS (ESI+) *m/z*: $[M + H]^+$ calcd for $C_{35}H_{35}N_2S_4^+$ 611.1677; found 611.1679.

Compound 21 was synthesized from solution of ketone **19** (142 mg, 0.238 mmol, 1 equiv.) in THF (2 mL) and hydrazine hydrate (400 µL, 8.3 mmol, 35 equiv.) in EtOH (6 mL) according to **GP5**. The reaction mixture was refluxed for 4.5 h in an oil bath, then volatiles were evaporated on rotary evaporator giving hydrazone **21** as a 1:1 mixture of *E*/*Z* isomers (145 mg, 0.238 mmol, 100%) in the form of yellowish solid.

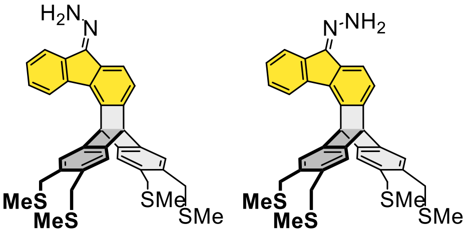

^{1}H NMR (400 MHz, $CDCl_3$): δ 8.39 (td, J_1 = 0.9 Hz, J_2 = 7.9 Hz, 1H), 8.27 (td, J_1 = 0.9 Hz, J_2 = 7.9 Hz, 1H), 7.96 (td, J_1 = 0.9 Hz, J_2 = 7.7 Hz, 1H), 7.81-7.79 (m, 1H), 7.63 (d, J = 7.5 Hz, 1H), 7.57 (dt, J_1 = 1.1 Hz, J_2 = 7.7 Hz, 1H), 7.46 (dt, J_1 = 1.2 Hz, J_2 = 7.6 Hz,

1H), 7.46 (d, J = 7.5 Hz, 1H), 7.40-7.28 (m, 16H), 6.28 (s, 1H), 6.20 (s, 1H), 5.43 (s, 1H), 5.40 (s, 1H), 3.80-3.70 (m, 16H), 2.04-2.03 (m, 24H). IR (KBr): 3397, 3227, 2958, 2911, 2853, 1628, 1605, 1578, 1468, 1423, 1316, 1234, 1187, 1130, 1111, 1088, 1068, 1024, 978, 958, 901, 841, 811, 770, 734, 696, 670, 623, 572 cm^{-1}. MS, m/z (%): 611.2 (100, M + H), 563.2 (61, M − SMe). HRMS (APCI+) m/z: $[M + H]^+$ calcd for $C_{35}H_{35}N_2S_4^+$ 611.1678; found 611.1674.

General Procedure for Synthesis of Diazo Compounds 22 and 23 (GP6)
Hydrazones **20** or **21** (1 equiv.) were dissolved in anhydrous CH_2Cl_2 in a dry and nitrogen purged two-necked flask. Activated MnO_2 (~4.3 equiv.) was added, and the reaction mixture was stirred at room temperature for 1 h and monitored using ^1H NMR spectroscopy. The reaction mixture was filtered through a syringe filter and solvent was evaporated yielding **22** or **23** as a pink solid residue, which was immediately used in the following reaction.

Compound 22 was synthesized from hydrazone **20** (271 mg, 0.444 mmol, 1 equiv.) and MnO_2 (166 mg, 1.91 mmol, 4.3 equiv.) in CH_2Cl_2 (4 mL) according to a **GP6**. The reaction mixture was filtered through a syringe filter and solvent was gently evaporated on rotavapor yielding **22** as a pink solid residue, which was immediately used in the following reaction.

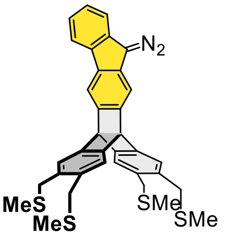

^1H NMR (400 MHz, $CDCl_3$): δ 7.90 (s, 1H), 7.85-7.82 (m, 1H), 7.50 (s, 1H), 7.44-7.42 (m, 1H), 7.32 (s, 2H), 7.30 (s, 2H), 7.28-7.27 (m, 1H), 7.25-7.23 (m, 1H), 5.44 (s, 1H), 5.41 (s, 1H), 3.75-3.68 (m, 8H), 2.027 (s, 6H), 2.025 (s, 6H). MS, m/z (%): 631.1 (65, M + Na), 626.2 (100). HRMS (ESI+) m/z: $[M + H]^+$ calcd for $C_{35}H_{33}N_2S_4^+$ 609.1521; found 609.1519.

Compound 23 was synthesized from hydrazone **21** (145 mg, 0.238 mmol, 1 equiv.) and MnO_2 (86 mg, 0.989 mmol, 4.2 equiv.) in CH_2Cl_2 (10 mL) according to a **GP6**. The reaction mixture was filtered through a syringe filter and solvent was gently evaporated on rotavapor yielding **23** as a pink solid residue, which was immediately used in the following reaction.

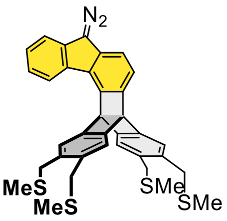

^1H NMR (400 MHz, $CDCl_3$): δ 8.31-8.29 (m, 1H), 7.66-7-64 (m, 1H), 7.55 (dt, J_1 = 1.6 Hz, J_2 = 7.7 Hz, 1H), 7.41-7.29 (m, 7H), 6.21 (s, 1H), 5.72 (s, 1H), 3.80-3.70 (m, 8H), 2.05-2.02 (m, 12H).

2-Methyl-2,3-dihydro-1H-benz[e]inden-1-thione (**24**). A 50 mL round-bottom flask was charged with ketone **25** (210 mg, 1.07 mmol, 1 equiv.) and Lawesson's reagent (653 mg, 1.61 mmol, 1.5 equiv.). Anhydrous toluene (15 mL) was added, and the reaction mixture was refluxed under nitrogen atmosphere for 2 h. Then, toluene was evaporated, and the dark green honey-like residue was subjected to flash column chromatography on silica gel (hexane/CH_2Cl_2 = 1:1, R_f = 0.7) yielding unstable thioketone **24** (213 mg, 1.00 mmol, 94%) as a green honey-like substance.

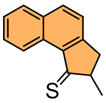

^1H NMR (400 MHz, $CDCl_3$): δ 10.11 (qd, J_1 = 0.9 Hz, J_2 = 8.6 Hz, 1H), 8.08 (d, J = 8.3 Hz, 1H), 7.93-7.90 (m, 1H), 7.73 (ddd, J_1 = 1.4 Hz, J_2 = 6.9 Hz, J_3 = 8.5 Hz, 1H), 7.57 (ddd, J_1 = 1.3 Hz, J_2 = 6.9 Hz, J_3 = 8.2 Hz, 1H), 7.55 (d, J = 8.4 Hz, 1H), 3.54 (dd, J_1 = 6.6 Hz, J_2 = 18.1 Hz, 1H), 3.21-3.13 (m, 1H), 2.94 (dd, J_1 = 2.4 Hz, J_2 = 18.1 Hz, 1H), 1.51 (d, J = 7.2 Hz, 3H). HRMS (EI+) m/z: $[M]^+$ calcd for $C_{14}H_{12}S^+$ 212.0654; found 212.0647.

2-Methyl-2,3-dihydro-1H-benz[e]inden-1-one (**25**).[38,49] A nitrogen-filled 1 L three-necked round-bottom flask was charged with anhydrous $AlCl_3$ (40.0 g, 300 mmol, 2 equiv.) and CH_2Cl_2 (240 mL). Subsequently, methacryloyl chloride (14.7 mL, 150 mmol, 1 equiv.) was added to the suspension at room temperature and the reaction mixture was stirred for 10 min after which it was cooled to -78 °C using an acetone/dry ice bath. Naphthalene (19.2 g, 150 mmol, 1 equiv.) was added portion wise over 3 minutes and the reaction mixture was stirred for an additional 5 min at the same temperature. The cooling bath was removed, and the reaction mixture was allowed to reach room temperature. After 2 h, the mixture was cooled in an ice-water bath, and the reaction was carefully quenched with ice and water (~100 g). The mixture was washed with water (400 mL), the aqueous phase was separated and extracted with CH_2Cl_2 (2 × 60 mL). The combined organic layers were washed with a saturated aqueous solution of $NaHCO_3$ (200 mL). The aqueous layer was extracted with CH_2Cl_2 (2 × 60 mL). The combined organic layers were dried over Na_2SO_4, and the solvent was removed using a rotary evaporator. The residue was sorbed onto silica gel in CH_2Cl_2, and flash column chromatography on silica gel (hexane/ethyl acetate = 20:1, R_f = 0.3) afforded ketone **25** (14.7 g, 75.0 mmol, 50%) as a beige oil, which solidified in a fridge.

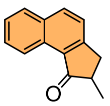

^{1}H NMR (500 MHz, $CDCl_3$): δ 9.16 (qd, J_1 = 0.9 Hz, J_2 = 8.4 Hz, 1H), 8.04 (d, J = 8.3 Hz, 1H), 7.90-7.88 (m, 1H), 7.67 (ddd, J_1 = 1.3 Hz, J_2 = 6.9 Hz, J_3 = 8.3 Hz, 1H), 7.55 (ddd, J_1 = 1.3 Hz, J_2 = 6.9 Hz, J_3 = 8.2 Hz, 1H), 7.50 (d, J = 8.4 Hz, 1H), 3.48 (dd, J_1 = 8.0 Hz, J_2 = 18.0 Hz, 1H), 2.85-2.79 (m, 2H), 1.38 (d, J = 7.4 Hz, 3H). ^{13}C {^{1}H} NMR (126 MHz, $CDCl_3$): δ 210.0, 156.6, 135.7, 132.7, 130.2, 129.6, 128.8, 128.1, 126.5, 124.0, 123.9, 42.4, 35.3, 16.6. IR (KBr): 3375, 3054, 2962, 2928, 2870, 2845, 1738, 1693, 1628, 1593, 1572, 1517, 1456, 1439, 1371, 1346, 1312, 1213, 1202, 1170, 1152, 1116, 1073, 1024, 989, 921, 881, 821, 788, 747, 629, 611, 566, 554, 512, 494, 428 cm^{-1}. MS, *m/z* (%): 197.1 (100, M + H), 141.1 (6). HRMS (APCI+) *m/z*: $[M + H]^+$ calcd for $C_{14}H_{13}O^+$ 197.0961; found 197.0960. Anal. calcd. for $C_{14}H_{12}O$: C, 85.68; H, 6.16. Found: C, 85.67; H, 6.23.

Compound S1 was identified as a byproduct (~821 mg, ~1.84 mmol, ~4%) of Diels-Alder reaction leading to triptycene **8**. This compound was identified in ^{1}H NMR spectra of **8** but has never been isolated as a clean chemical specie.

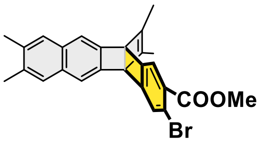

^{1}H NMR (400 MHz, $CDCl_3$): δ 7.73 (s, 1H), 7.57 (s, 1H), 7.52 (s, 1H), 7.51 (s, 1H), 7.43 (br s, 2H), 4.78 (s, 1H), 4.76 (s, 1H), 3.88 (s, 3H), 2.36 (s, 6H), 1.84 (s, 6H).

Compound S2 was obtained as a byproduct during synthesis of triptycene **9** and was isolated using preparative HPLC (hexane/CH_2Cl_2 = 4:1) as a white solid (436 mg, 1.28 mmol, 6%).

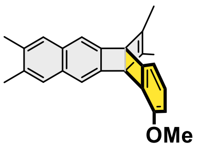

Mp 256-258 °C. ^{1}H NMR (400 MHz, $CDCl_3$): δ 7.55 (s, 1H), 7.51 (s, 1H), 7.43 (br s, 2H), 6.99-6.94 (m, 2H), 6.62-6.60 (m, 1H), 5.28 (s, 1H), 4.79 (s, 1H), 3.86 (s, 3H), 2.37 (s, 6H), 1.88-1.86 (m, 6H). ^{13}C {^{1}H} NMR (100 MHz, $CDCl_3$): δ 153.8, 147.9, 142.6, 142.0, 138.6, 138.5, 134.58, 134.55, 133.1, 130.3, 130.2, 127.01, 126.98, 125.53, 119.2, 119.1, 115.7, 108.1, 56.7, 55.7, 49.2, 20.07, 16.2, 16.1. IR (KBr): 3428, 3059, 3005, 2989, 2964, 2949, 2918, 2903, 2849, 2838, 2729, 1617, 1602,

[38] U. Dietrich, M. Hackmann, B. Rieger, M. Klinga, M. Leskelä, "Control of Stereoerror Formation with High-Activity "Dual-Side" Zirconocene Catalysts: A Novel Strategy to Design the Properties of Thermoplastic Elastic Polypropenes" *J. Am. Chem. Soc.* **1999**, *121* (18), 4348–4355.

[49] P. Štacko, J. C. M. Kistemaker, B. L. Feringa, "Fluorine-Substituted Molecular Motors with a Quaternary Stereogenic Center" *Chem. Eur. J.* **2017**, *23* (27), 6643-6653.

1589, 1498, 1480, 1441, 1380, 1372, 1359, 1327, 1292, 1266, 1234, 1212, 1196, 1176, 1153, 1131, 1117, 1104, 1071, 1020, 1001, 973, 951, 930, 895, 887, 860, 788, 767, 743, 733, 713, 643, 596, 588, 546, 521, 480, 469, 416 cm^{-1}. MS, *m/z* (%): 341.2 (100). HRMS (APCI+) *m/z*: $[M + H]^+$ calcd for $C_{25}H_{25}O^+$ 341.1900; found 341.1896. Anal. calcd. for $C_{25}H_{24}O$: C, 88.20; H, 7.11. Found: C, 88.28; H, 7.06.

3. Synthesis of Anthranilic Acid 6

Anthranilic acid **6** was synthesized on a large scale in three steps from dimethyl 2-aminoterephthalate (**Scheme S1**). Bromination with NBS produced a mixture of primarily *p*-brominated **S3**,[50] along with *o*-brominated **S4** and dibrominated **S5**. The desired **S3** was easily separated via column chromatography in 78% yield. Subsequent hydrolysis of **S3** with LiOH·H_2O afforded diacid **S6** in nearly quantitative yield. The final step involved a selective monoesterification of **S6** using TMSCl in MeOH, yielding anthranilic acid **6** and diester **S3** as a side product. A simple sequence of deprotonation, extraction, acidification, and a second extraction enabled full separation, providing **6** and **S3** in yields of 76% and 22%, respectively (**Scheme S1**).

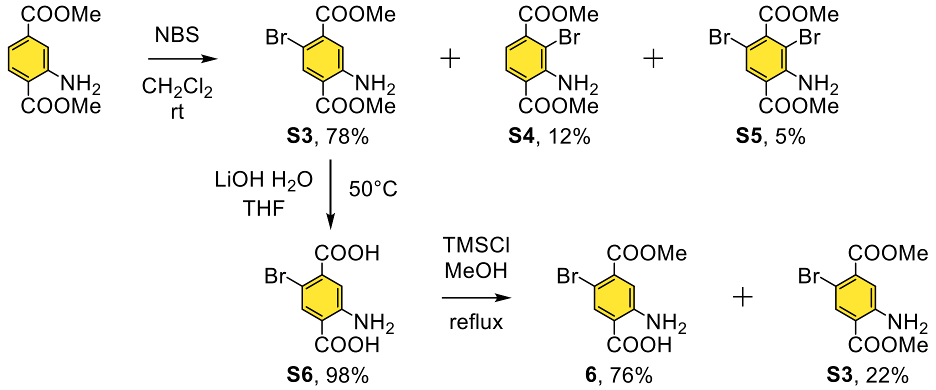

Scheme S1. Synthesis of anthranilic acid **6**.

Compound 6.[50] Compound **S6** (58.9 g, 227 mmol, 1 equiv.) was placed in a three-necked flask equipped with a reflux condenser and purged with nitrogen. Then MeOH (600 mL) followed by TMSCl (38 mL, 299 mmol, 1.3 equiv.) were added upon which the suspension dissolved with immediate color change (yellow suspension to orange solution). The reaction mixture was then refluxed overnight. Afterwards the reaction mixture was cooled down to room temperature, concentrated on rotary evaporator and placed into a separatory funnel. Saturated solution of Na_2CO_3 (500 mL) was added and extracted using ethyl acetate (3 × 300 mL). Combined organic layers were washed with saturated aqueous $NaHCO_3$ (3 × 200 mL), dried over $MgSO_4$ and volatiles were removed on rotary evaporator, which provided side product **S6** (12.8 g, 44.4 mmol, 22%) as a yellow powder. Combined aqueous layers were acidified (pH ~5) with acetic acid. Resulting smooth suspension was placed into a separatory funnel and extracted with ethyl acetate (3 × 500 mL), dried over $MgSO_4$ and volatiles were remover on rotary evaporator. Compound **6** (41.6 g, 152 mmol, 76%) was obtained as a light-yellow powder.

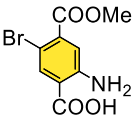

Mp 194-203 °C. ^{1}H NMR (400 MHz, DMSO-d_6): δ 7.88 (s, 1H), 7.14 (s, 1H), 3.84 (s, 3H). ^{13}C {^{1}H} NMR (100 MHz, DMSO-d_6): δ 167.9, 166.0, 150.1, 136.2, 135.5, 118.5, 113.7, 101.6, 52.6. IR (KBr): 3468, 3384, 3346, 2950, 2620, 1719, 1678, 1620, 1582, 1544, 1475, 1436, 1421, 1316, 1224, 1120, 1004, 939, 906, 810, 792, 778, 705, 646, 603, 573, 497, 476 cm^{-1}. MS, *m/z* (%): 296.0 (44, M + Na), 274.0 (100, M + H). HRMS (ESI+) *m/z*: $[M + H]^+$ calcd for $C_9H_9BrNO_4^+$ 273.9709; found 273.9711. Anal. calcd. for $C_9H_8BrNO_4$: C, 39.44; H, 2.94; N, 5.11. Found: C, 39.33; H, 2.84; N, 5.15.

[50] P. Štacko, J. C. M. Kistemaker, B. L. Feringa, "Fluorine-Substituted Molecular Motors with a Quaternary Stereogenic Center" *Chem. Eur. J.* **2017**, *23* (27), 6643-6653.

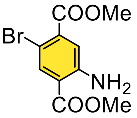

Dimethyl 2-amino-5-bromoterephthalate (**S3**).[50] Dimethyl 2-aminoterephthalate (77.1 g, 369 mmol, 1 equiv.) and NBS (72.3 g, 406 mmol, 1.1 equiv.) were placed into a round-bottom flask and dissolved in CH_2Cl_2 (3.5 L). The reaction mixture was stirred at room temperature for 28 h after which the solvent was evaporated. The solid residue was separated using a column chromatography on silica gel (hexane/CH_2Cl_2 = 1:1, R_f = 0.1) yielding bromide **S3** (82.9 g, 289 mmol, 78%) as a yellow powder.

Mp 119-122 °C. ^{1}H NMR (400 MHz, $CDCl_3$): δ 8.09 (s, 1H), 7.05 (s, 1H), 5.81 (s, 2H), 3.92 (s, 3H), 3.89 (s, 3H). ^{13}C {^{1}H} NMR (100 MHz, $CDCl_3$): δ 166.9, 166.2, 148.8, 136.8, 136.2, 119.2, 113.7, 105.2, 52.6, 52.0. IR (KBr): 3470, 3361, 3163, 3024, 2952, 2844, 1724, 1686, 1617, 1583, 1546, 1475, 1437, 1413, 1313, 1235, 1190, 1107, 1004, 958, 929, 901, 877, 811, 792, 786, 779, 706, 686, 644, 611, 567, 438 cm^{-1}. MS, *m/z* (%): 310.0 (17, M + Na), 288.0 (100, M + H). HRMS (ESI+) *m/z*: $[M + H]^+$ calcd for $C_{10}H_{11}O_4NBr^+$ 287.9866; found 287.9868. Anal. calcd. for $C_{10}H_{10}BrNO_4$: C, 41.69; H, 3.50; N, 4.86. Found: C, 41.30; H, 2.87; N, 4.74.

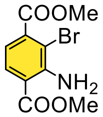

Dimethyl 2-amino-3-bromoterephthalate (**S4**) was isolated using recrystallization (hexane) from a mixed fraction of **S4** and **S5**. Collected crystals were washed by hexane (5 mL) and dried on rotary evaporator. Compound **S4** was obtained as a light-yellow powder (9.07 g, 43.4 mmol, 12%).

Mp 106-109 °C. ^{1}H NMR (400 MHz, $CDCl_3$): δ 7.89 (d, J = 8.3 Hz, 1H), 6.87 (d, J = 8.3 Hz, 1H), 6.61 (s, 2H), 3.93 (s, 3H), 3.90 (s, 3H). ^{13}C {^{1}H} NMR (100 MHz, $CDCl_3$): δ 167.5, 167.2, 148.1, 138.2, 130.3, 116.1, 112.9, 108.8, 52.7, 52.2. IR (KBr): 3418, 3324, 3014, 2959, 1744, 1700, 1605, 1582, 1533, 1469, 1434, 1417, 1302, 1262, 1204, 1189, 1160, 1105, 1058, 1008, 964, 905, 842, 831, 804, 797, 750, 728, 609, 559, 488, 455 cm^{-1}. MS, *m/z* (%): 286.9 (56, M + H), 254.9 (100, M − OMe). HRMS (EI+) *m/z*: $[M]^+$ calcd for $C_{10}H_{10}BrNO_4^+$ 286.9788; found 286.9788. Anal. calcd. for $C_{10}H_{10}BrNO_4$: C, 41.69; H, 3.50; N, 4.86. Found: C, 41.44; H, 3.48; N, 4.71.

Dimethyl 2-amino-3,5-dibromoterephthalate (**S5**) was isolated using recrystallization (hexane) from a mixed fraction of **S4** and **S5**. Volatiles were evaporated on rotary evaporator and dibromide **S5** was obtained as a white powder (7.38 g, 20.1 mmol, 5%).

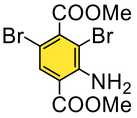

Mp 109-111 °C. ^{1}H NMR (400 MHz, $CDCl_3$): δ 8.06 (s, 1H), 6.50 (s, 2H), 3.98 (s, 3H), 3.90 (s, 3H). ^{13}C {^{1}H} NMR (100 MHz, $CDCl_3$): δ 166.5, 166.3, 146.8, 141.8, 134.0, 112.6, 107.8, 102.8, 53.1, 52.4. IR (KBr): 3474, 3347, 3091, 3009, 2954, 1739, 1699, 1600, 1566, 1517, 1449, 1434, 1416, 1297, 1245, 1200, 1173, 1107, 1082, 1013, 956, 917, 838, 807, 791, 750, 730, 688, 656, 573, 515, 496, 414 cm^{-1}. MS, *m/z* (%): 366.8 (92, M + 2H), 334.8 (100, M − OMe). HRMS (EI+) *m/z*: $[M]^+$ calcd for $C_{10}H_9Br_2NO_4^+$ 364.8893; found 364.8891. Anal. calcd. for $C_{10}H_9Br_2NO_4$: C, 32.73; H, 2.47; N, 3.82. Found: C, 33.07; H, 2.53; N, 3.78.

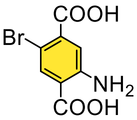

2-Amino-5-bromo-1,4-benzenedicarboxylic acid (**S6**).[50] Diester **S3** (58.0 g, 0.202 mol, 1 equiv.) was placed in a round-bottom flask together with $LiOH{\cdot}H_2O$ (84.8 g, 2.02 mol, 10 equiv.), THF (150 mL) and water (300 mL) and stirred at 50 °C overnight. Organic solvent was removed on rotary evaporator and the water phase was cooled down in an ice bath. Upon acidification (pH ~3) with concentrated HCl bright yellow precipitate appeared, which was then filtered on frit, washed with water (700 mL) and dried on a rotary evaporator. Diacid **S6** was obtained as a light-yellow powder (58.9 g, 0.199 mol, 98%).

Mp 270-275 °C. ^{1}H NMR (400 MHz, DMSO-d_6): δ 7.85 (s, 1H), 7.11 (s, 1H). $^{13}C\{^{1}H\}$ NMR (100 MHz, DMSO-d_6): δ 167.9, 167.2, 150.2, 138.3, 135.3, 118.1, 112.4, 101.7. IR (KBr): 3498, 3385, 3166, 3044, 2871, 2590, 1906, 1819, 1681, 1606, 1561, 1481, 1436, 1389, 1340, 1251, 1146, 1110, 1033, 964, 920, 812, 801, 793, 756, 693, 632, 544, 496, 425 cm^{-1}. MS, *m/z* (%): 262.0 (100, M + 2H). HRMS (ESI+) *m/z*: $[M + H]^+$ calcd for $C_8H_7BrNO_4^+$ 259.9553; found 259.9557. Anal. calcd. for $C_8H_6BrNO_4$: C, 36.95; H, 2.33; N, 5.39. Found: C, 36.92; H, 2.51; N, 5.26.

4. HPLC Separation of Compounds 1 and 2

Prep

Method Name: Bezdekova/Motory
Run Name: Bezdekova/2024-06-11_KB336-HPLC3
Run Date: 2024-06-11 10:54

BUCHI

Column: PrepPure Silica 250x30m
Flow Rate: 30 mL/min
Equilibration: 15.0 min
Run Length: 22.0 min
Instrument Type: C-835
Mode: Prep
Sample type: Liquid

Solvent A: Hexane
Solvent B: Dichloromethane
Solvent C: Ethyl acetate
Solvent D: Empty
Slope Detection: Off

UV Threshold: 0.02 AU
UV Sensitivity: Low
UV1 λ: 254 nm
UV2 λ: 265 nm
UV3 λ: 280 nm
UV4 λ: 320 nm
UV scan start λ: 254 nm (M)
UV scan end λ: 400 nm (M)

Collection: Collect Peaks
Per-Vial Volume: 20 mL
Non-Peak Volume: 20 mL

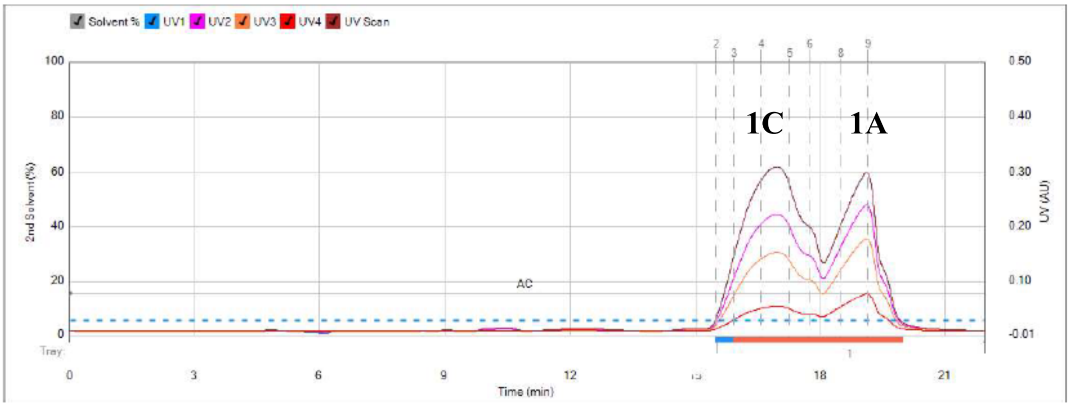

1 - 610E

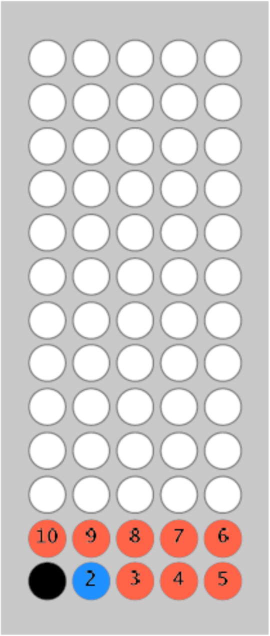

Gradient Table

	Min	Solvents	% 2nd
1	0.0	AC	15
2	25.0	AC	15

Vial Mapping Table

Peak #	Start Tray:Vial	End Tray:Vial
1	1:2	1:2
2	1:3	1:10

Page 1

Figure S1. HPLC separation of motors **1A** and **1C**.

Prep

Method Name: Bezdekova/Motory
Run Name: Severa/2024-06-11-LS559
Run Date: 2024-06-11 15:43

Column: PrepPure Silica 250x30m
Flow Rate: 30 mL/min
Equilibration: 15.0 min
Run Length: 25.0 min
Instrument Type: C-835
Mode: Prep
Sample type: Liquid

Solvent A: Hexane
Solvent B: Dichloromethane
Solvent C: Ethyl acetate
Solvent D: Empty
Slope Detection: Off

UV Threshold: 0.01 AU
UV Sensitivity: Low
UV1 λ: 254 nm
UV2 λ: 265 nm
UV3 λ: 280 nm
UV4 λ: 320 nm
UV scan start λ: 254 nm (M)
UV scan end λ: 400 nm (M)

Collection: Collect Peaks
Per-Vial Volume: 20 mL
Non-Peak Volume: 20 mL

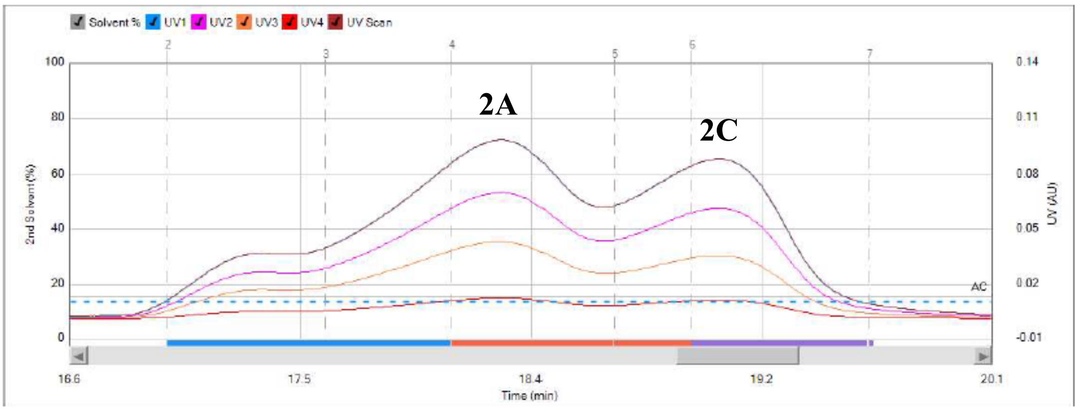

1 - 610E

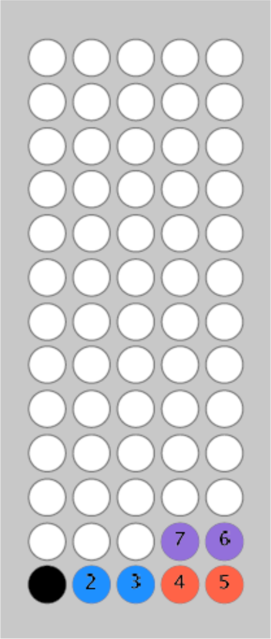

Gradient Table

	Min	Solvents	% 2nd
1	0.0	AC	15
2	25.0	AC	15

Vial Mapping Table

Peak #	Start Tray:Vial	End Tray:Vial
1	1:2	1:3
2	1:4	1:5
3	1:6	1:7

Page 1

Figure S2. HPLC separation of motors **2A** and **2C**.

5. X-Ray Diffraction

The diffraction data for single crystal structure determination of **1C**, **4**, **5**, **9**, **13**, **15**, **16**, **17**, **18** and **S2** were performed on Bruker D8 VENTURE Kappa Duo PHOTONIII by IμS micro-focus sealed tube either with MoKα (λ = 0.71073 for **1C**, **5**, **15**, **16**, **17**) or CuKα (λ = 1.54178 for **4**, **9**, **13**, **18**, **S2**) radiation at low temperature preserved by Cryostream Cooler. The phase problems were solved by direct methods (XT)[51] and structures were refined by full matrix least squares based on F^2 (SHELXL2019).[52] The hydrogen atoms on carbon were fixed into idealized positions (riding model) and assigned temperature factors $H_{iso}(H) = 1.2\ U_{eq}$(pivot atom). Basic crystallographic data are given in **Table S1**.

Single crystals were grown either by slow diffusion of pentane to CH_2Cl_2 solution of **1C**, **13**, **16**, **18**; hexane to $CDCl_3$ solution of **9**, **15**, **17**; hexane to $CHCl_3$ solution of **5**; methanol to $CHCl_3$ solution of **4**; or methanol to 1,2-dichloroethane solution of **S2**.

The compounds in this study are inclined to form disordered crystals:

In the unit cell of **1C**, one of the thiomethyl groups exhibits complex disorder, with two modeled positions failing to fully account for the electron density, resulting in large displacement ellipsoids for the disordered atoms. The absolute structure was determined using anomalous scattering of sulfur atoms, with the refined chirality parameter[53] reaching a value of 0.07(4).

The displacement parameters of the carbon atoms in compound **5** indicate molecular disorder within the crystal packing.

For compound **9**, the entire molecule was found in two positions with an occupancy ratio of 0.91:0.09.

The crystal of compound **17** contains a disordered dichloromethane solvate. Additionally, the structure is complicated by non-merohedral twinning, with block volume ratios of 0.7:0.3.

In compound **18**, disorder affects not only the thiomethyl groups but also the opposite part of the molecule. The sample was measured at different temperatures, but this approach did not lead to a less disordered structure.

X-ray crystallographic data has been deposited with the Cambridge Crystallographic Data Centre (CCDC), the deposition numbers are given in **Table S1** and can be obtained free of charge from the Centre via its website (www.ccdc.cam.ac.uk/structures/).

[51] G. M. Sheldrick, "*SHELXT* – Integrated Space-Group and Crystal-Structure Determination" *Acta Cryst.* **2015**, *A71* (1), 3-8.
[52] G. M. Sheldrick, "Crystal Structure Refinement with *SHELXL*" *Acta Cryst.* **2015**, *C71* (1), 3-8.
[53] S. Parsons, H. D. Flack, T. Wagner, "Use of Intensity Quotients and Differences in Absolute Structure Refinement" *Acta Cryst*. **2013**, *B69* (3), 249-259.

Table S1. Parameters of Single Crystals of **1C**, **4**, **5**, **9**, **13**, **15**, **16**, **17**, **18** and **S2**.

**Cmpd.** (deposition #)[a]	**Crystal System**	**Space Group**	**Cell Lengths (Å)**	**Cell Angles (°)**
**1C** (2434427)	orthorhombic	P $2_12_12_1$	*a* 18.2485(7)<br>*b* 20.4419(7)<br>*c* 10.5804(4)	*α* 90<br>*β* 90<br>*γ* 90
**4** (2434426)	monoclinic	P $2_1/n$	*a* 6.1522(2)<br>*b* 7.6178(2)<br>*c* 26.3596(8)	*α* 90<br>*β* 91.9710(10)<br>*γ* 90
**5** (2434429)	monoclinic	P $2_1/c$	*a* 7.1158(6)<br>*b* 24.2444(17)<br>*c* 7.4595(5)	*α* 90<br>*β* 94.952(3)<br>*γ* 90
**9** (2434425)	monoclinic	P $2_1/c$	*a* 8.4789(7)<br>*b* 16.2429(13)<br>*c* 14.1317(12)	*α* 90<br>*β* 107.535(3)<br>*γ* 90
**13** (2434424)	monoclinic	P $2_1/c$	*a* 18.9367(6)<br>*b* 12.4162(4)<br>*c* 10.2691(3)	*α* 90<br>*β* 100.7180(10)<br>*γ* 90
**15** (2434430)	monoclinic	C $2/c$	*a* 33.1691(11)<br>*b* 9.2744(3)<br>*c* 16.6402(6)	*α* 90<br>*β* 111.7950(10)<br>*γ* 90
**16** (2434428)	monoclinic	P $2_1/c$	*a* 14.9302(7)<br>*b* 12.4356(5)<br>*c* 13.9786(7)	*α* 90<br>*β* 103.653(2)<br>*γ* 90
**17** (2434431)	monoclinic	P $2_1/c$	*a* 15.5462(13)<br>*b* 8.1008(6)<br>*c* 20.7250(17)	*α* 90<br>*β* 96.950(3)<br>*γ* 90
**18** (2434423)	monoclinic	C $2/c$	*a* 17.4713(5)<br>*b* 12.1442(3)<br>*c* 16.1694(7)	*α* 90<br>*β* 122.5030(10)<br>*γ* 90
**S2** (2411038)	triclinic	P -1	*a* 8.2393(2)<br>*b* 14.4118(4)<br>*c* 16.4789(4)	*α* 105.0240(10)<br>*β* 98.4780(10)<br>*γ* 97.9160(10)

[a] Deposition numbers from Cambridge Crystallographic Data Centre (CCDC). Structures can be obtained free of charge from the Centre via its website (www.ccdc.cam.ac.uk/structures/).

6. UV-vis Analysis in Solution

Temperature-dependent UV-visible spectra were acquired using a Varian Cary 5000 spectrophotometer equipped with Cary WinUV software. Cooling, temperature stabilization, and irradiation were achieved with a custom-made cell holder utilizing a water-cooled Peltier element and integrated LED modules. The system uses two commercial thermometers TM-RS232 for temperature measurements with a precision of 0.1 °C.

The thermometer and the power supply were connected to a PC via USB ports and controlled with custom-written software for temperature stabilization. The standard temperature deviation was 0.06 °C over the whole measured region.

Two different modules can be installed at the same time, and due to the integration into the UV-vis spectrometer, there is no need to manipulate the sample during the irradiation-measurement cycle, eliminating interference from ambient laboratory lighting and minimizing delay between irradiation and measurement. Replaceable LED modules with desired wavelength are controlled through the aforementioned software, which is used to set time and intensity of irradiation. The holder has an integrated stirrer to ensure uniform irradiation of the whole volume. The distance from the diodes to the cuvette is 15 mm as shown in **Figure S3**.

The total duration of each experiment corresponded to three half-lives at the respective temperatures, with measurement intervals of 80 s at 0 °C, 40 s at 5 °C, and 25 s at 10–25 °C.

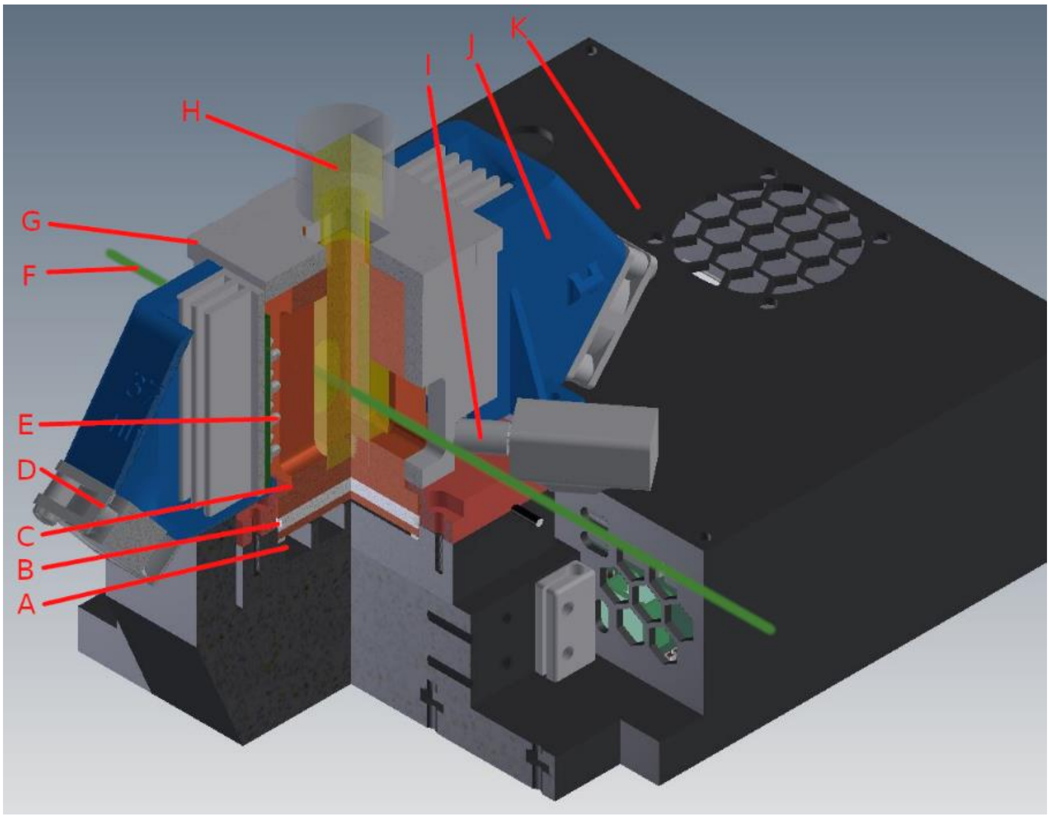

Figure S3. Schematic representation of the Thermophotoreactor setup: a) Core thermal management assembly showing the copper holder (**C**) mounted on the Peltier element (**B**), equipped with water cooling (**A**), and encased in insulating foam (**G**) for optimal thermal control. b) Illumination system comprising two diode modules: the first module shown in cross-section reveals its internal components - cooling fan (**D**) and diodes (**E**), while the second module is depicted as a complete sealed unit (**J**). c) Measurement configuration displaying the measuring beam (**F**) path through the cuvette with sample (**H**). d) Additional components including the stirrer (**I**) and electronic controller case (**K**) for system operation and control.

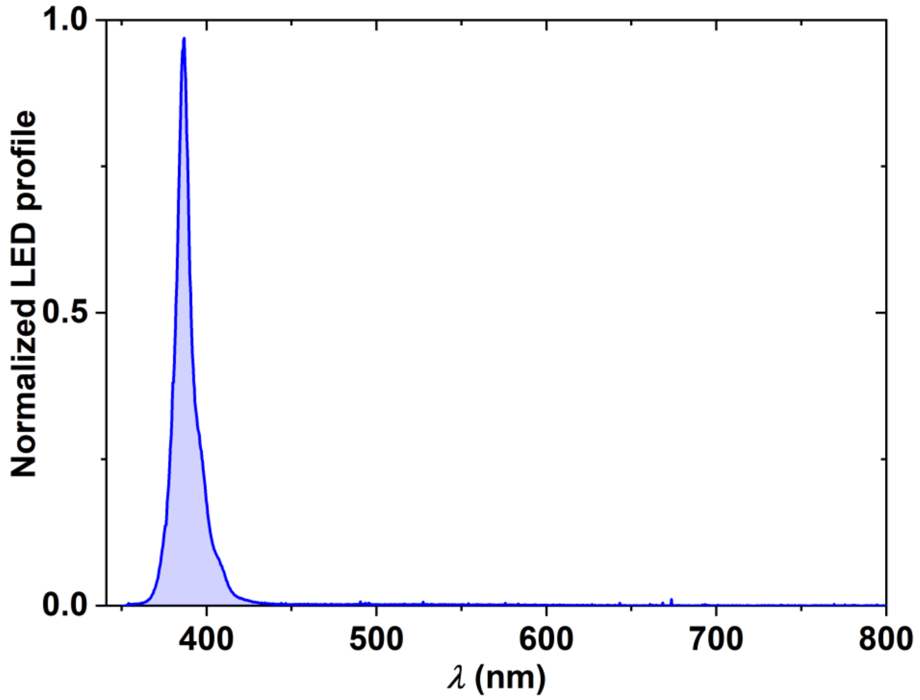

Figure S4. Emission profile of the LED 385±5 nm (light flux 0.394 W/cm^2) used in all photochemical experiments in this study.

Compound 1

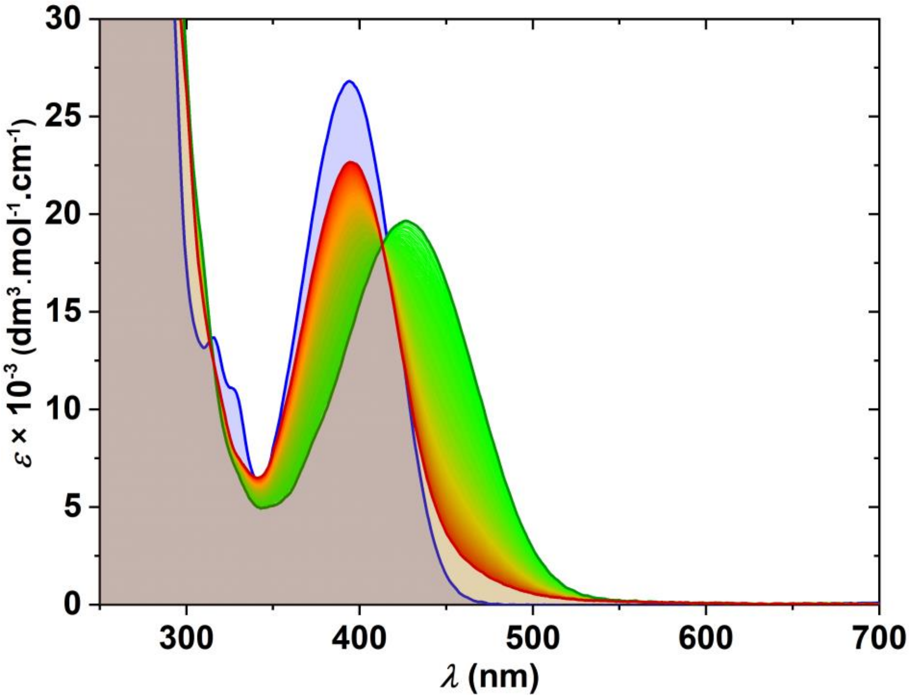

Figure S5. Rotation cycle **1A→1B→1C** followed by UV-vis at 0 °C. First half of the rotation cycle showing **1A**, the same sample in PSS after irradiation at 385±5 nm (**1B**), and after subsequent thermal relaxation to **1C**.

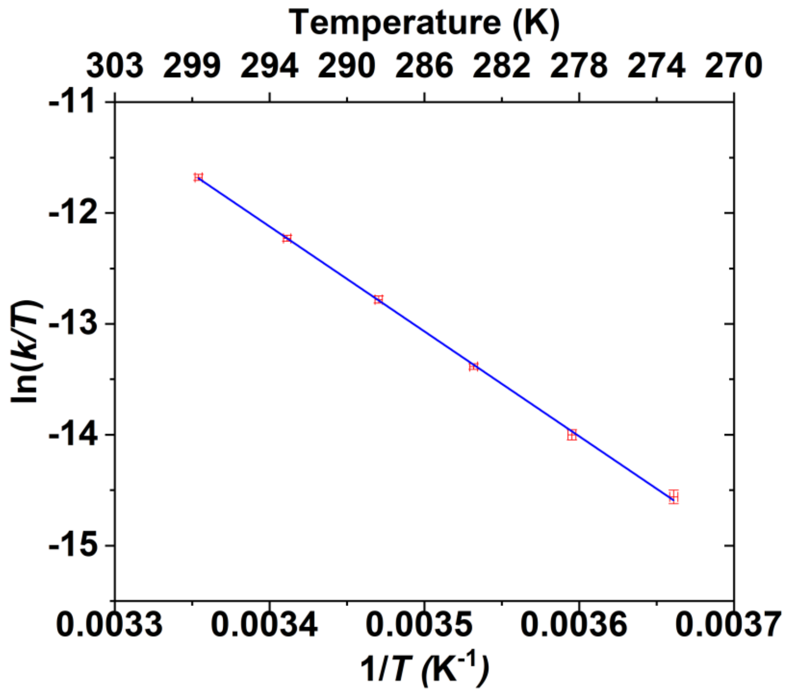

Figure S6. Eyring plot for the **1B→1C** isomerization in $CHCl_3$ (c = 1×10^{-5} M), with error bars shown in red.

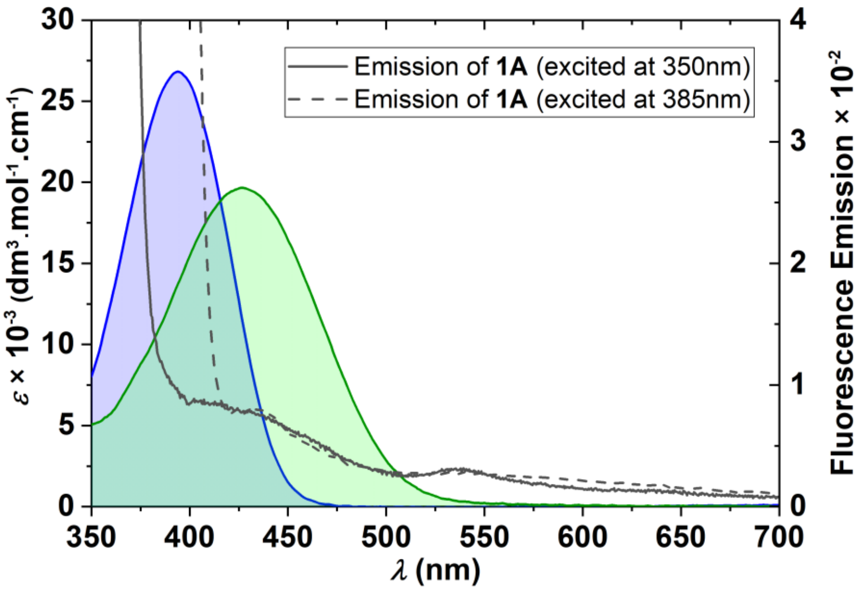

Figure S7. Absorption spectra of compound **1A** (blue), **1B** (green) and fluorescence emission spectra of **1A** excited at 350 nm (grey solid line) and at 385 nm (grey dashed line) in $CHCl_3$ (c = 1×10^{-5} M).

Table S2. Kinetic parameters of **1A** at 20 °C extracted from Eyring plot.

Eyring equation	ln(k/T) = −9462.63/T + 20.05
R^2 value	0.9996
$\Delta H^{\ddagger}$	79 ± 1 kJ/mol
$\Delta G^{\ddagger}$	88 ± 2 kJ/mol
$\Delta S^{\ddagger}$	-31 ± 4 J/mol·K
k^0	$(1.43 \pm 0.05)\times10^{-3}$
$t_{1/2}$	8.1 ± 0.3 min

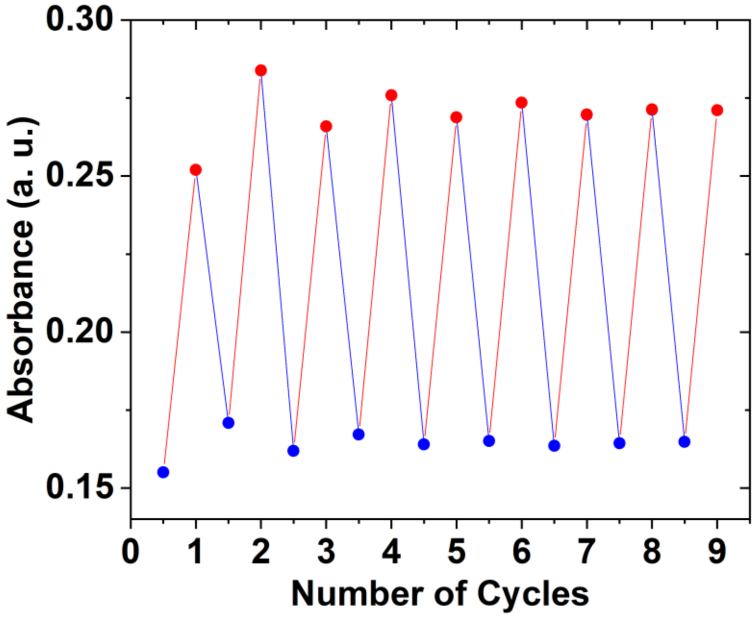

Figure S8. Stability of **1A** in $CHCl_3$ ($c = 1\times10^{-5}$ M) at 20 °C during nine consecutive cycles initiated by irradiation at 385±5 nm.

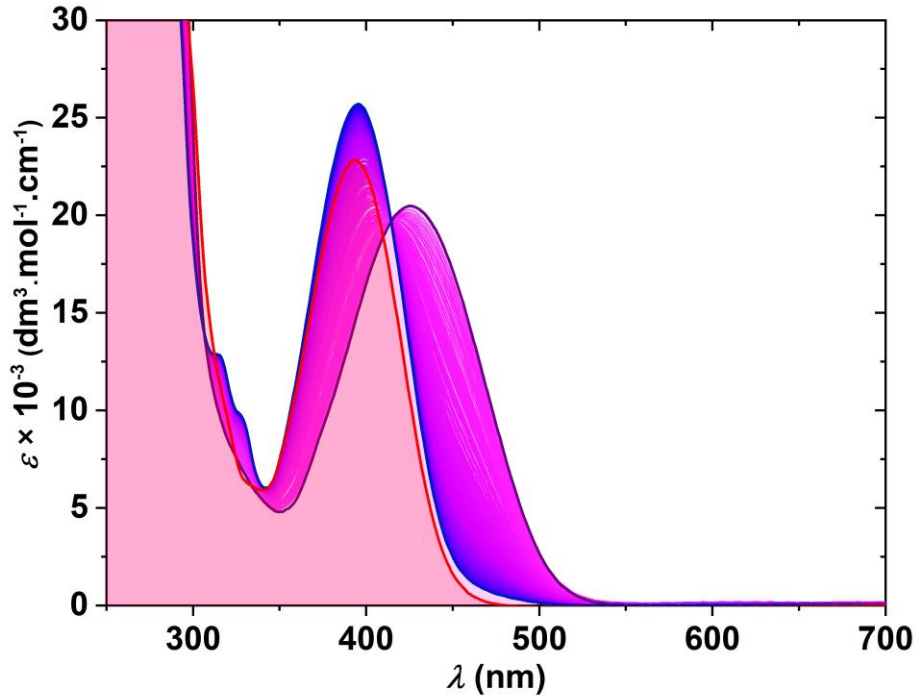

Figure S9. Rotation cycle **1C→1D→1A** followed by UV-vis at 0 °C. First half of the rotation cycle showing **1C**, the same sample in PSS after irradiation at 385±5 nm (**1D**), and after subsequent thermal relaxation to **1A**.

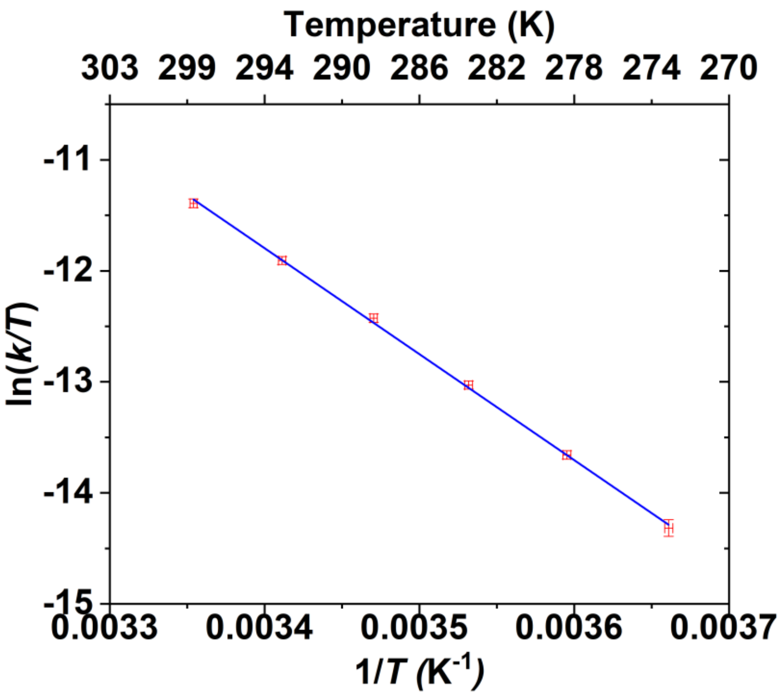

Figure S10. Eyring plot for the **1D→1A** isomerization in $CHCl_3$ (c = 1×10^{-5} M), with error bars shown in red.

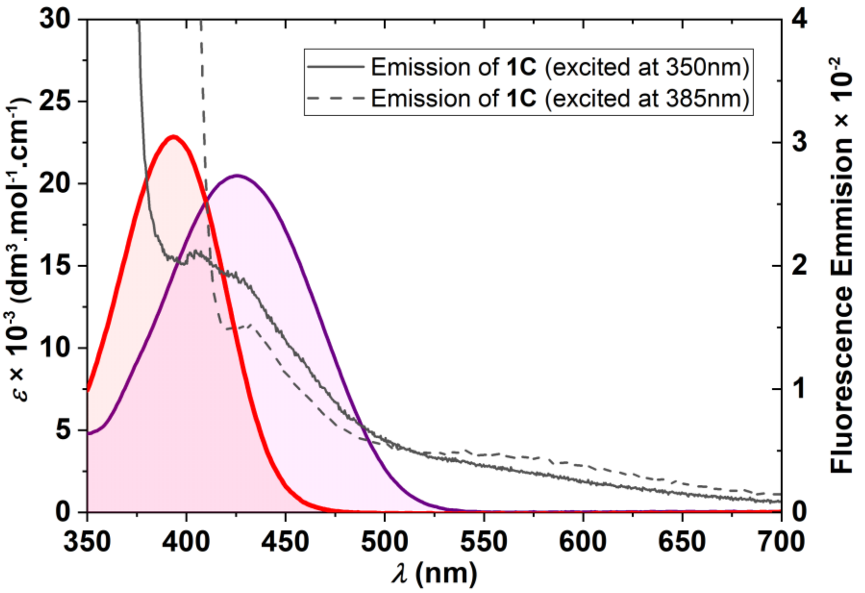

Figure S11. Absorption spectra of compound **1C** (red), **1D** (purple) and fluorescence emission spectra of **1C** excited at 350 nm (grey solid line) and at 385 nm (grey dashed line) in $CHCl_3$ (c = 1×10^{-5} M).

Table S3. Kinetic parameters of **1C** at 20 °C extracted from Eyring plot.

Eyring equation	$\ln(k/T) = -9539.56/T + 20.64$
R^2 value	0.9993
$\Delta H^{\ddagger}$	79 ± 1 kJ/mol
$\Delta G^{\ddagger}$	87 ± 3 kJ/mol
$\Delta S^{\ddagger}$	-26 ± 5 J/mol·K
k^0	$(1.98 \pm 0.09)\times10^{-3}$
$t_{1/2}$	5.8 ± 0.3 min

Compound 2

The rate of thermal conversion of isomer B to C (k_B) and isomer D to A (k_D) may differ. The standard method for measuring this rate is to isolate one of the stable isomers A or C and then measure the kinetics using UV spectroscopy. However, in this case, we were unable to completely isolate the isomers; instead, we had two mixtures - one with a predominance of isomer A and the other with a predominance of isomer C. We measured kinetic data (rates k_1 and k_2) for both mixtures and thus obtained mixed rate constants under the simplified assumption that the decay is monoexponential.

To obtain the values of k_B and k_D, we used a mathematical model based on the assumption that there is no transition of isomer B to isomer A or isomer D to isomer C (Equations 1-4).

$$A = D_0(1 - e^{-k_D t}) + A_0 \quad \text{Eq. 1}$$
$$B = B_0 e^{-k_B t} \quad \text{Eq. 2}$$
$$C = B_0(1 - e^{-k_B t}) + C_0 \quad \text{Eq. 3}$$
$$D = D_0 e^{-k_D t} \quad \text{Eq. 4}$$

A, B, C, D – amount of isomer A, B, C, D in time t

A_0, B_0, C_0, D_0 – starting amount of isomer A, B, C, D

The monoexponential decay fitted on measured UV spectroscopy data is shown in equations 5 to 6.

$$B + D = (B_0 + D_0)e^{-k_1 t} \quad \text{Eq. 5}$$
$$B' + D' = (B'_0 + D'_0)e^{-k_2 t} \quad \text{Eq. 6}$$

B, D, B_0, D_0 – first mix

B', *D'*, B_0', D_0' – second mix

The parameters k_B and k_D were then obtained by fitting equations 7 and 8.

$$(B_0 + D_0)e^{-k_1 t} = B_0 e^{-k_B t} + D_0 e^{-k_D t} \qquad \text{Eq. 7}$$

$$(B_0' + D_0')e^{-k_2 t} = B_0' e^{-k_B t} + D_0' e^{-k_D t} \qquad \text{Eq. 8}$$

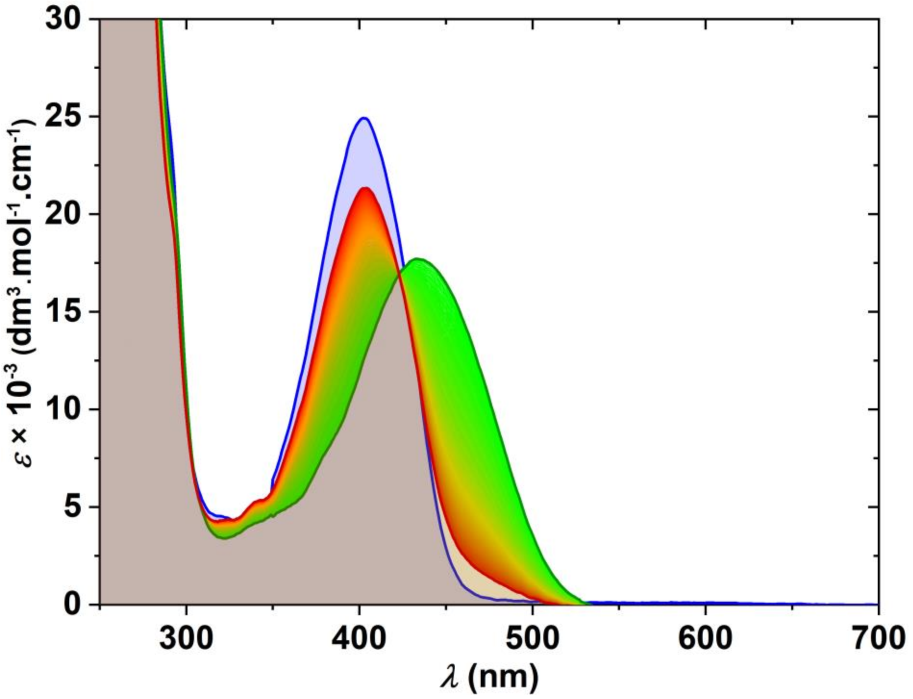

Figure S12. Rotation cycle **2A→2B→2C** followed by UV-vis at 0 °C. First half of the rotation cycle showing **2A**, the same sample in PSS after irradiation at 385±5 nm (**2B**), and after subsequent thermal relaxation to **2C**.

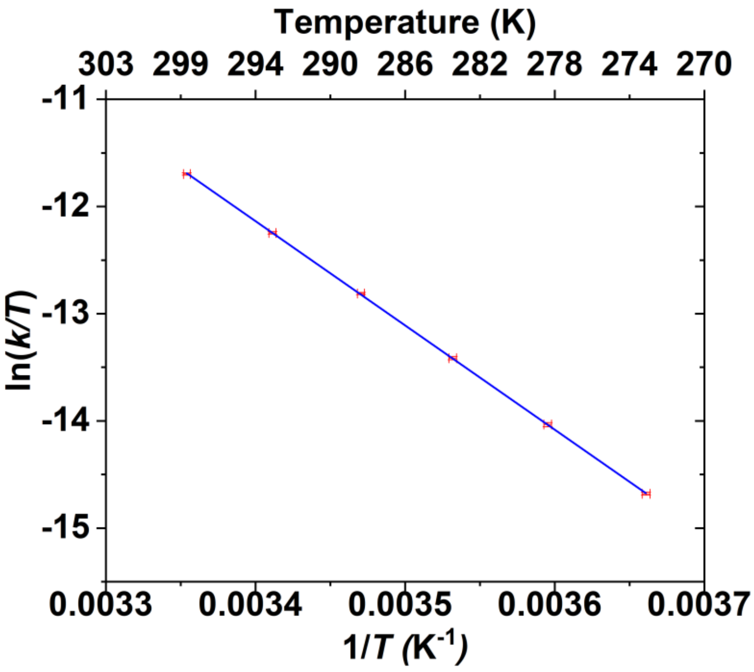

Figure S13. Eyring plot for the **2B→2C** isomerization in $CHCl_3$ (c = 1×10^{-5} M), with error bars shown in red.

Table S4. Kinetic parameters of **2A** at 20 °C extracted from Eyring plot.

Eyring equation	$\ln(k/T) = -9721.45/T + 20.92$
R^2 value	0.9999
$\Delta H^{\ddagger}$	81 ± 1 kJ/mol
$\Delta G^{\ddagger}$	88 ± 1 kJ/mol
$\Delta S^{\ddagger}$	-24 ± 2 J/mol·K
k^0	$(1.41 \pm 0.02)\times10^{-3}$
$t_{1/2}$	8.2 ± 0.1 min

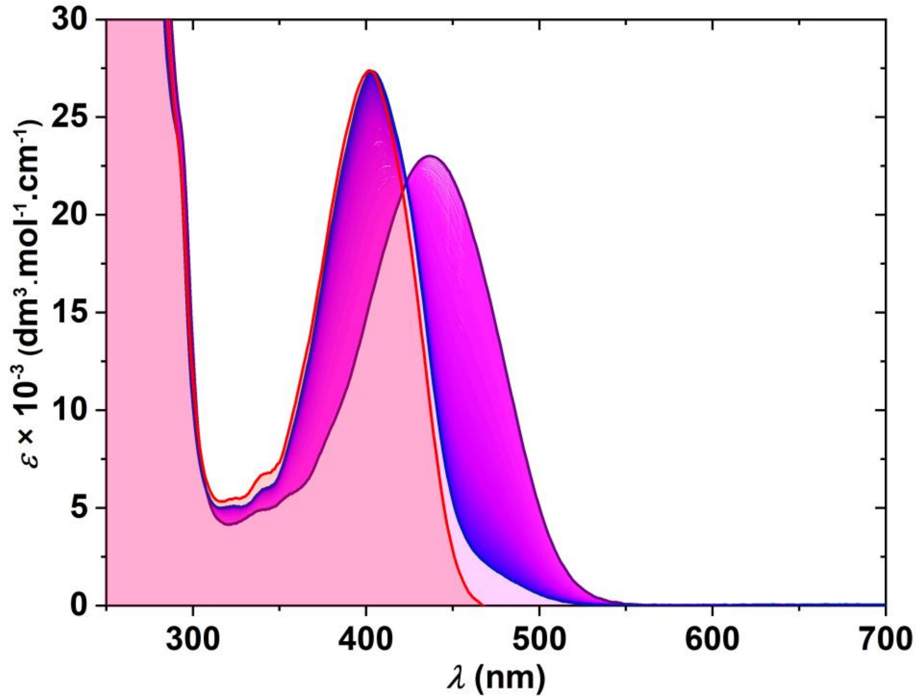

Figure S14. Rotation cycle **2C→2D→2A** followed by UV-vis at 0 °C. First half of the rotation cycle showing **2C**, the same sample in PSS after irradiation at 385±5 nm (**2D**), and after subsequent thermal relaxation to **2A**.

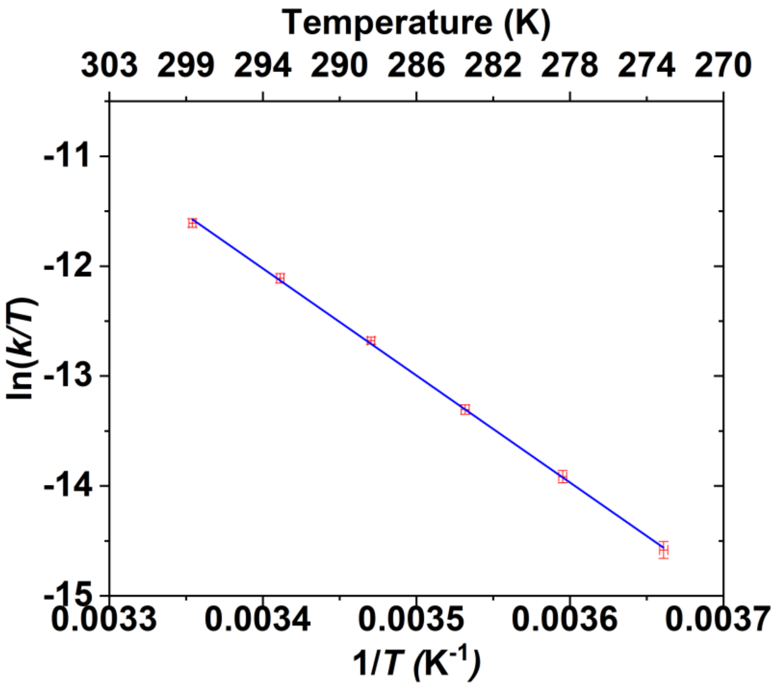

Figure S15. Eyring plot for the **2D**→**2A** isomerization in $CHCl_3$ ($c = 1\times10^{-5}$ M), with error bars shown in red.

Table S5. Kinetic parameters of **2C** at 20 °C extracted from Eyring plot.

Eyring equation	ln(k/T) = −9751.29/T + 21.21
R^2 value	0.9989
$\Delta H^‡$	81 ± 2 kJ/mol
$\Delta G^‡$	87 ± 4 kJ/mol
$\Delta S^‡$	-22 ± 6 J/mol·K
k^0	$(1.71 \pm 0.11)\times10^{-3}$
$t_{1/2}$	6.8 ± 0.4 min

7. ^{1}H NMR Analysis in Solution

Samples were prepared in Norell quartz NMR 500MHz tubes, dissolved in $CDCl_3$ with addition of a drop of deuterated pyridine. The sample concentrations were maintained at 1.5 mM for all experiments.

Irradiation of compounds for ^{1}H NMR analysis was performed using the same LED module (385±5 nm) described in the **UV-vis analysis section 6**. The module was connected to a power supply and placed in a polystyrene box containing a dry ice block, which cooled the irradiation area to -50 °C. The temperature was monitored with a thermometer. The NMR tube was positioned 20 mm from the LED, and all four samples were irradiated at maximum module power for 60 minutes to reach the PSS.

After irradiation, the NMR tubes were quickly transferred to a Bruker Avance IITM HD 500 MHz spectrometer, pre-cooled to -20 °C, for ^{1}H NMR measurements. For each isomer, three states were analyzed: before irradiation, at PSS, and after heating the samples, all measured at -20 °C, as shown in **Figures S16-S17**.

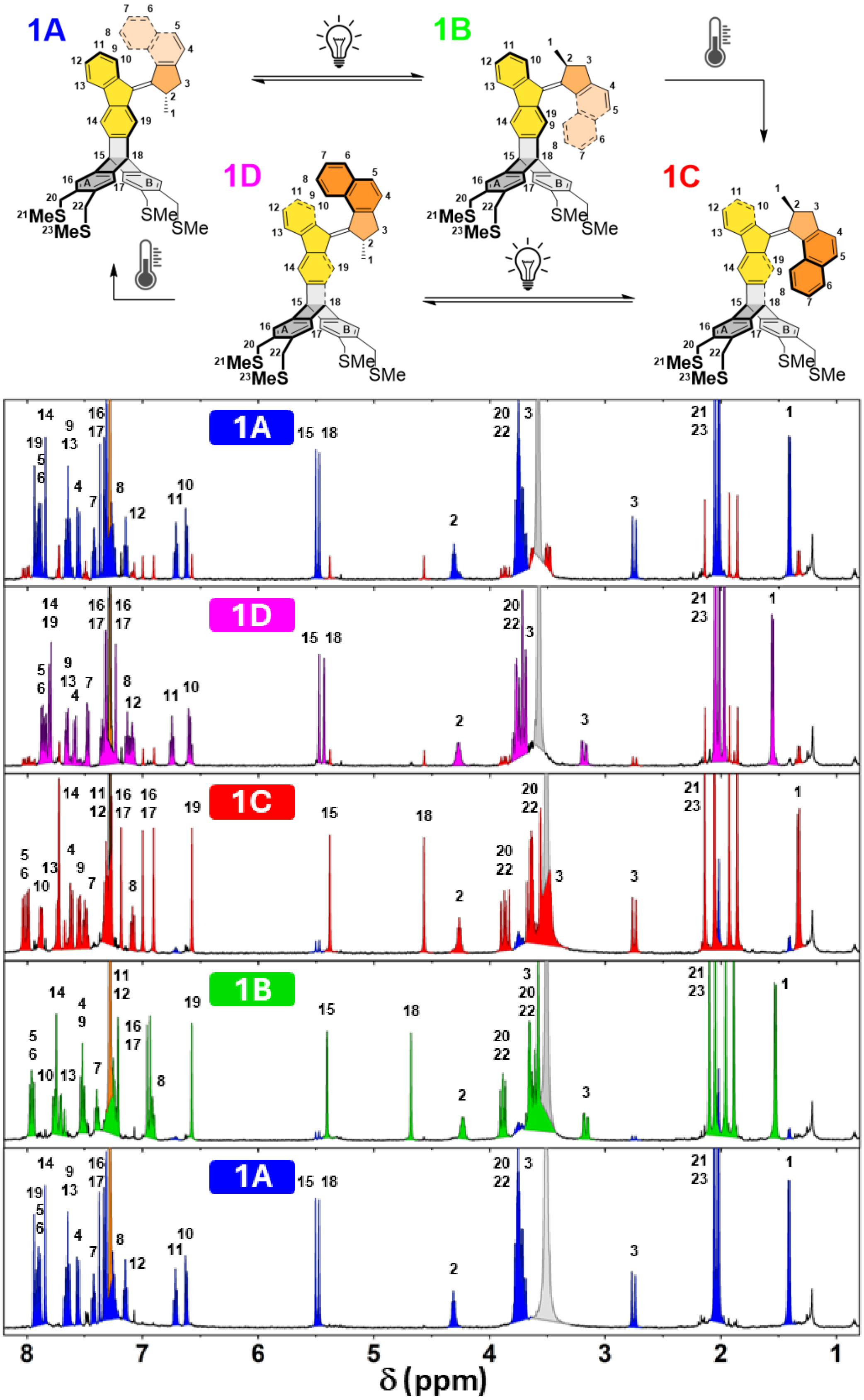

Figure S16. Four-stages rotation cycle of molecular motor **1** with assigned hydrogen atoms (upper part) and corresponding ^{1}H NMR spectra in $CDCl_3$ at -20 °C (lower part).

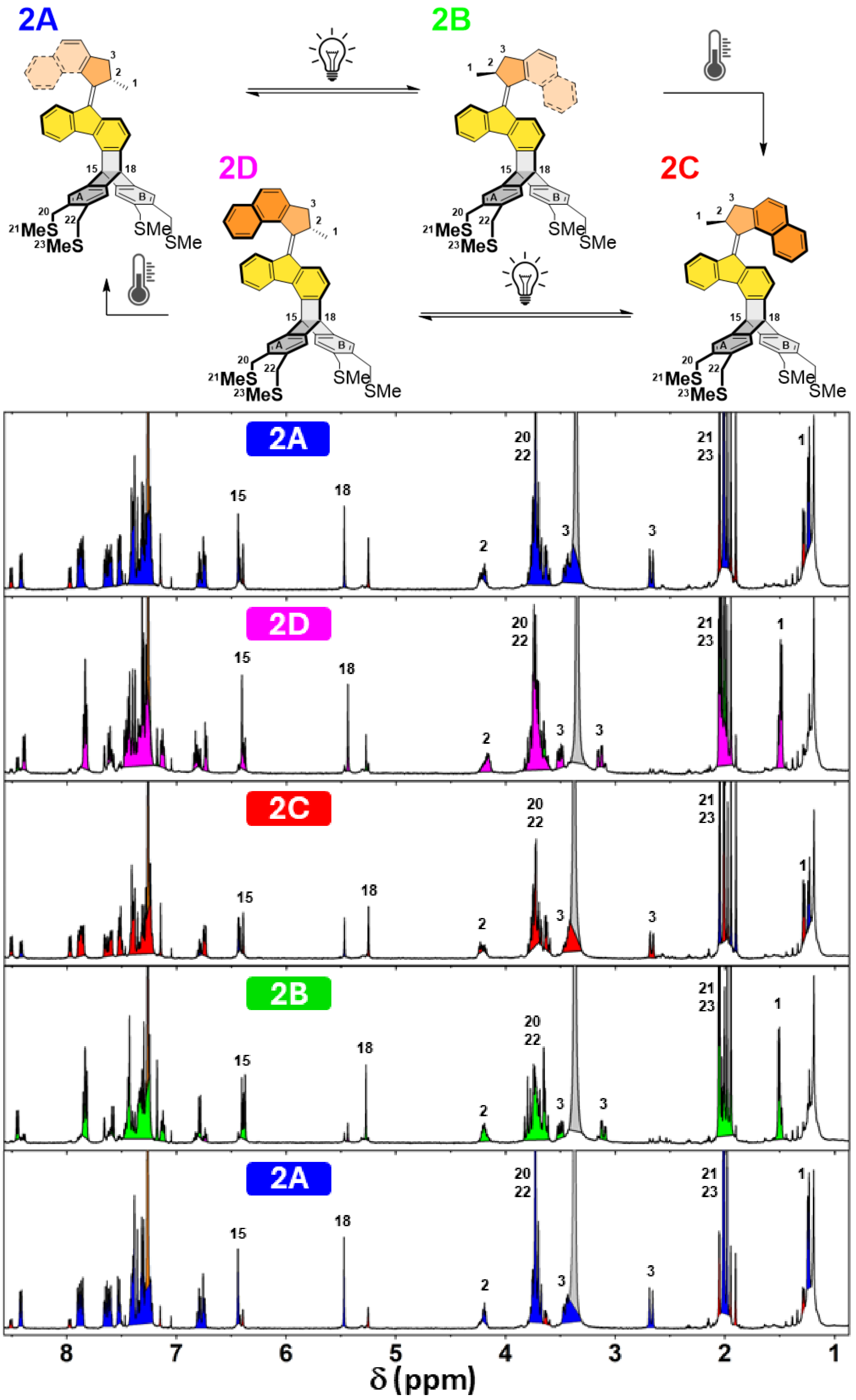

Figure S17. Four-stages rotation cycle of molecular motor **2** with partially assigned hydrogen atoms (upper part) and corresponding ^{1}H NMR spectra in $CDCl_3$ at -20 °C (lower part).

8. Monolayer Preparation

Preparation of semi-transparent gold films

Quartz substrates (UV Fused Silica Plate PFS-2521 form UQG optics) were cut into 1.0 cm × 2.0 cm pieces, cleaned by immersing in a piranha solution at 90 ºC for 1 h and rinsed with mQ water (3 times), then sonicated in water (2 × 5 min) and then rinsed with acetone and dried under a stream of N_2 before surface modification.

A previously published procedure[12] was adapted as follows: Before gold deposition, the quartz substrates were silanized by immersing them in a 1 mM solution of (3-aminopropyl)-triethoxysilane (Aldrich, 99%) in toluene for five days. The substrates were then thoroughly rinsed with toluene, sonicated in toluene, rinsed with methanol, and dried under a stream of nitrogen.

Gold films were prepared by two various methods (i) vapor deposition of a 5 nm thick layer of gold at the speed of 0.5 Å/s and (ii) vapor deposition of a 10 nm thick layer of gold at the speed of 0.2 Å/s, followed by a deposition of a 3 nm thick layer of gold nanoparticles at the speed of 4 Å/s.

Preparation of gold films on glass

Gold films were prepared by vapor deposition of a 185 nm thick gold layer on glass slides at a rate of 0.1 Å/s. A 5 nm titanium interlayer was deposited at 1 Å/s as an adhesive coating. The substrates were cleaned in an ultrasonic bath with Hellmanex and hot water for 5 minutes, followed by six ultrasonic washes in distilled water (5 minutes each). Finally, they were rinsed twice with propyl alcohol. These substrates were used for ellipsometry, contact angle, and PM-IRRAS measurements.

Monolayer preparation

Monolayers of **1** and **2** were prepared by immersing the substrates in a 1×10^{-4} M solution in freshly distilled THF or acetonitrile for 24-48 hours. After removal, the substrates were thoroughly rinsed with THF or acetonitrile (4 × 10 mL) and dried under a nitrogen stream before analysis.

---

[12] K.-Y. Chen, O. Ivashenko, G. T. Carroll, J. Robertus, J. C. M. Kistemaker, G. London, W. R. Browne, P. Rudolf, B. L. Feringa, "Control of Surface Wettability Using Tripodal Light-Activated Molecular Motors" *J. Am. Chem. Soc.* **2014**, *136*, 3219–3224.

9. Ellipsometry and Contact Angle Goniometry

For compounds **1A** and **2A**, ellipsometric thickness was determined by analyzing three samples per compound, with at least seven measurements per sample, using a Variable Angle Stokes Ellipsometer (Gaertner Scientific, USA). A refractive index of 1.47 was used, based on previous studies.[28] The characteristic ellipsometric thickness for each film was calculated as the average of these measurements, excluding any outliers.

For each monolayer, at least five contact angle measurements were performed across two samples using a CAM 101 Contact Angle Goniometer (KSV Instruments Ltd., Finland). The characteristic contact angle for each film was calculated as the average of all recorded values, excluding any outliers.

Table S6. Experimentally Determined Contact Angles of SAMs made of **1A**, **2A**, **18** and **19**.

Cmpd.	Contact Angle (°)
**1A**	60 ± 2
**2A**	64 ± 3
**18**	52 ± 3
**19**	53 ± 2

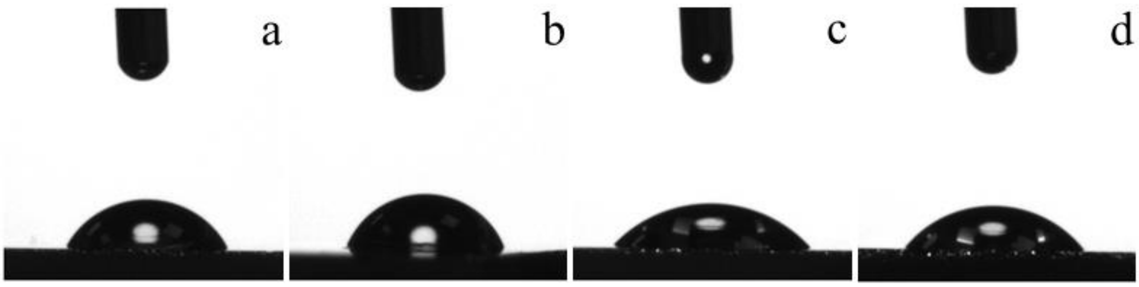

Figure S18. Pictures of a single water droplet on a SAMs of compounds **1A** (a), **2A** (b), **18** (c) and **19** (d) on gold substrates.

[28] J. Kaleta, E. Kaletová, I. Císařová, S. J. Teat, J. Michl, "Synthesis of Triptycene-Based Molecular Rotors for Langmuir–Blodgett Monolayers" *J. Org. Chem.* **2015**, *80* (20), 10134–10150.

10. Polarization Modulation-Infrared Reflection Absorption Spectroscopy (PM-IRRAS)

Infrared spectra were recorded on freshly produced monolayer samples immediately after immersion, rinsing and drying. The acquisition time was 2 hours (6,000 scans), during which the detector was cooled with liquid nitrogen. At least three spectra per compound were recorded. The raw data was background subtracted, and baseline corrected. The Polarization-Modulation Infrared Reflection Absorption Spectrometer (Thermo Fisher Scientific, USA) was used for these experiments.

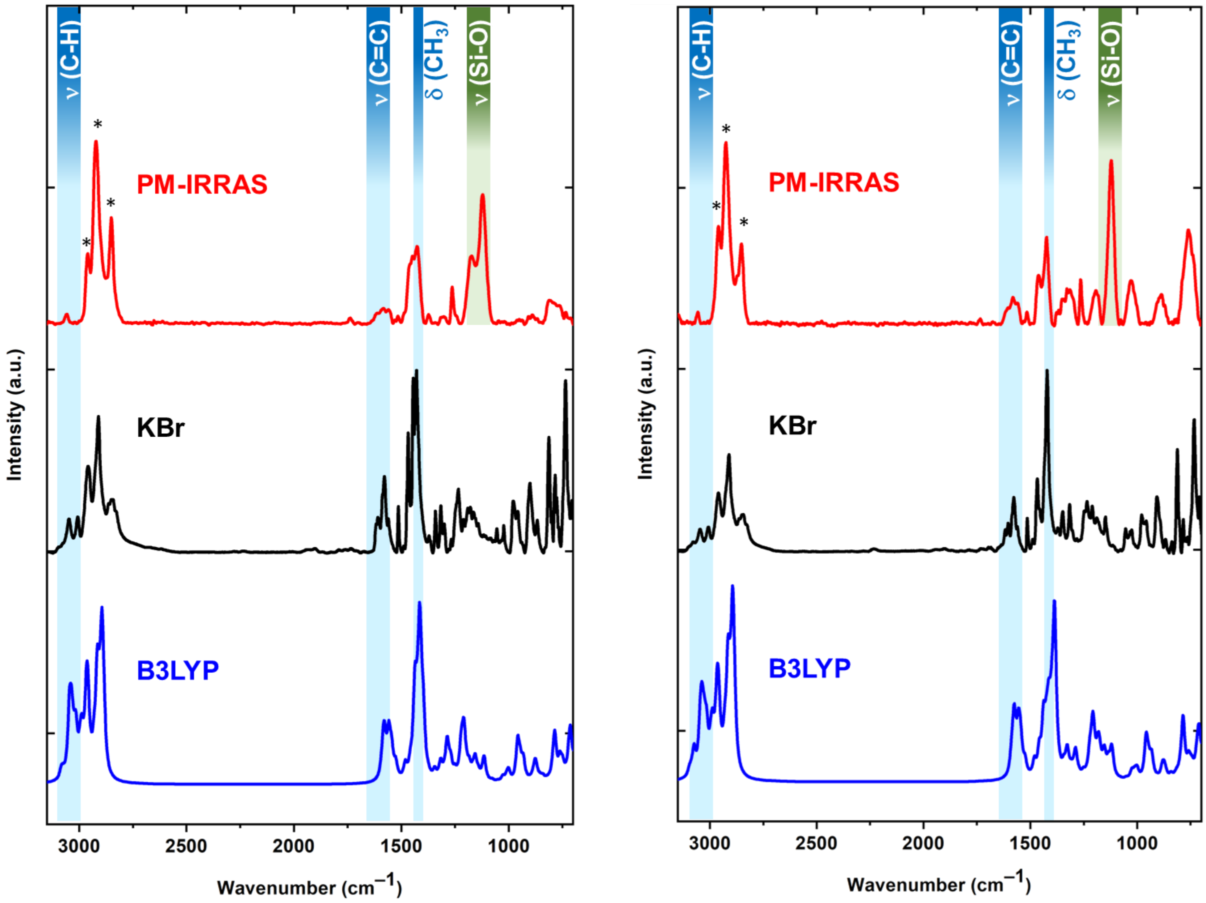

Figure S19. IR spectra of motors **1A** (left) and **2A** (right): monolayer recorded by PM-IRRAS (red), isotropic sample in KBr (black) and DFT calculated (blue). Asterisks indicate peaks whose intensities might be affected by common surface contamination.

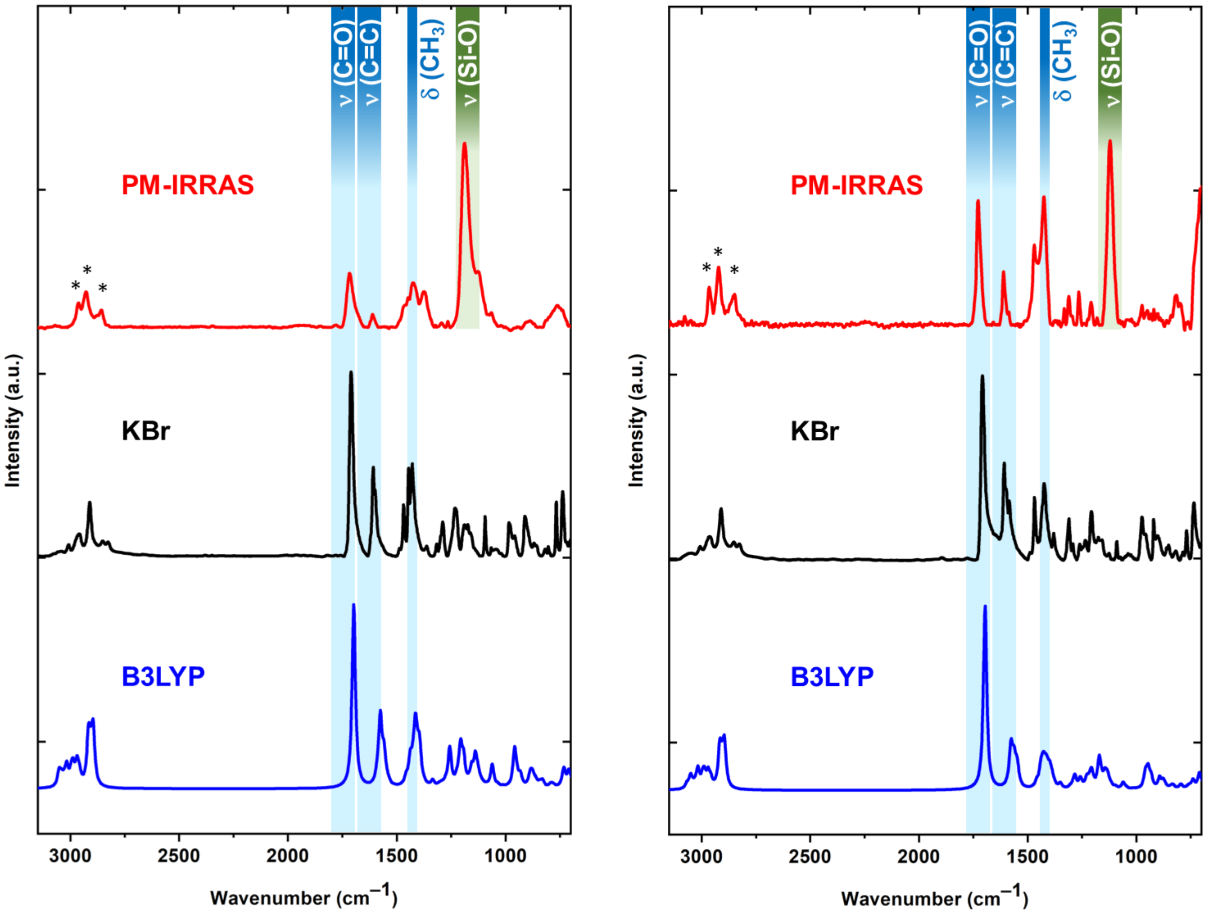

Figure S20. IR spectra of stators **18** (left) and **19** (right): monolayer recorded by PM-IRRAS (red), isotropic sample in KBr (black) and DFT calculated (blue). Asterisks indicate peaks whose intensities might be affected by common surface contamination.

11. Raman Spectroscopy

Raman measurements were carried out on a Renishaw inVia™ Qontor® upright confocal Raman microscope equipped with 532, 633, and 785 nm laser sources. For each sample, the experimental parameters (laser wavelength, laser power, acquisition time, and number of accumulations) were individually optimized to ensure the best spectral quality. Initial characterization of the prepared layers was performed by collecting spectra at randomly selected surface points, followed by acquisition of a 100 × 100 µm Raman map to assess the uniformity of the films. The spectra presented in this study were recorded under the following conditions: 785 nm excitation, 1200 l/mm grating, and a 100× objective (NA 0.85, WD 0.33 mm, Leica). Specific measurement parameters were as follows: laser power 8.8 mW, 1 s acquisition, 200 accumulations for compound **1A**; 44 mW, 1 s acquisition, 200 accumulations for compound **25**; 44 mW, 1 s acquisition, 200 accumulations for compound **18**; 4.4 mW, 1 s acquisition, 100 accumulations for a self-assembled monolayer (SAM) of **1A**; and 0.9–1.25 mW, 1 s acquisition, 200 accumulations for a gold-on-quartz reference sample.

Raman spectra were first recorded for compound **1A** in its solid form, as well as for the precursors **18** and **25** (**Figure S21**). To characterize SAMs made of **1A** on quartz substrates (**Figure S22**), multiple surface points were selected and a Raman surface map was acquired over an area of 100 × 100 mm. To enhance the Raman signal, the quartz substrates used for **1A** deposition were pre-treated with gold nanoparticles (method b, **Section 8**). The raw Raman data were processed by background subtraction and baseline correction.

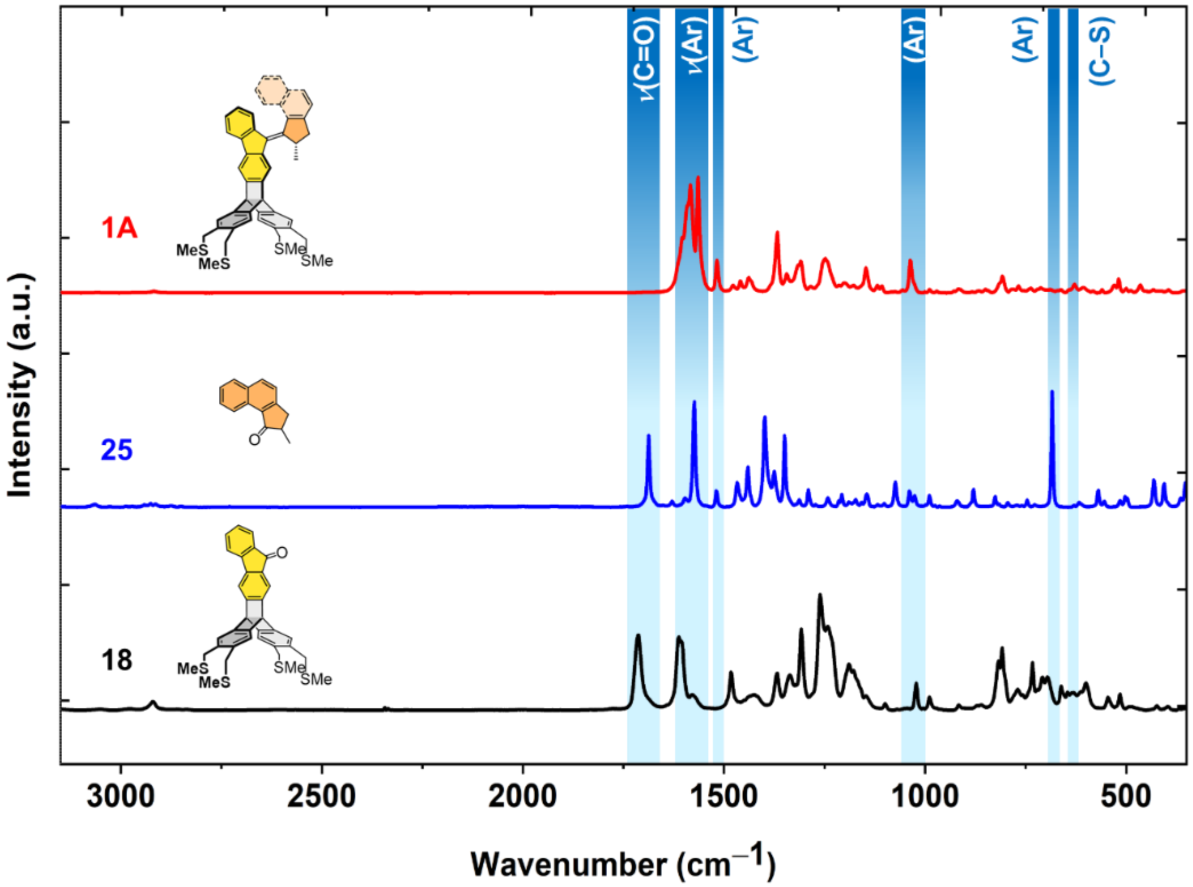

Figure S21. Comparison of Raman spectra of motor **1A** (red) with rotor **25** (blue) and stator **18** (black).

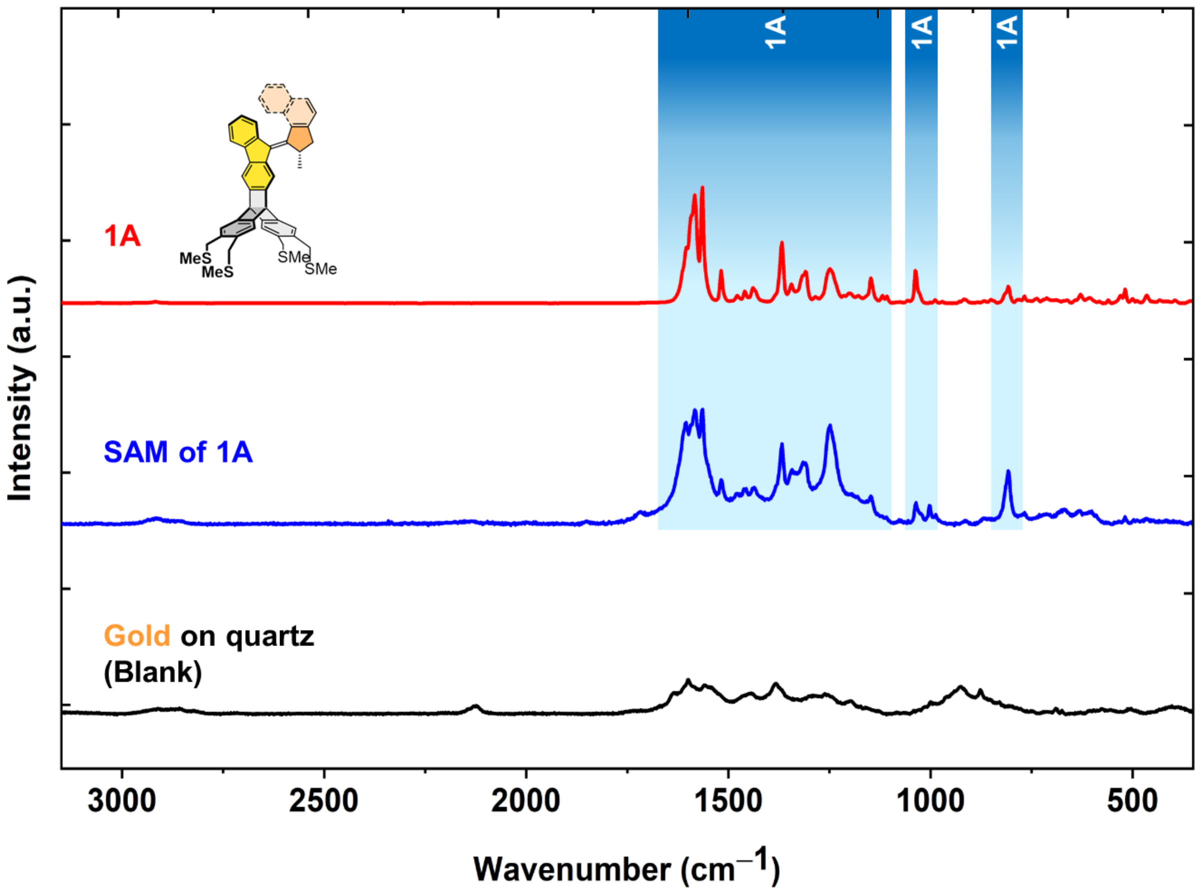

Figure S22. Raman spectra of clean gold on quartz substrate (black), neat **1A** (red) and SAM of **1A** on a gold on quartz (blue).

12. Atomic Force Microscopy (AFM)

The substrates were prepared by resistive evaporation of 100 nm gold 99.99% (Kurt Lesker) in vacuum (base pressure 10^{-7} mbar) onto a freshly cleaved muscovite V4 substrate. To obtain the Au(111) surface, the substrates were thermally annealed in a butane flame immediately after removal from the vacuum chamber.

Compounds **1A** and **2A** were subsequently deposited on the gold surface following the general procedure described for the **Monolayer Preparation** (Section 8). AFM was measured on Veeco-Bruker Multimode in PeakForce mode with a positive setpoint of 20-350 pN, using Bruker SNL-B cantilevers with 2 nm tip nominal diameter.

All thickness measurements were performed by "nanoshaving" (contact mode with the maximum setpoint applied to scratch off any material above the gold layer) an area clear of any material and subsequent topography assessment.

The AFM data were further processed in Gwyddion software to remove periodical noise and imaging artefacts.

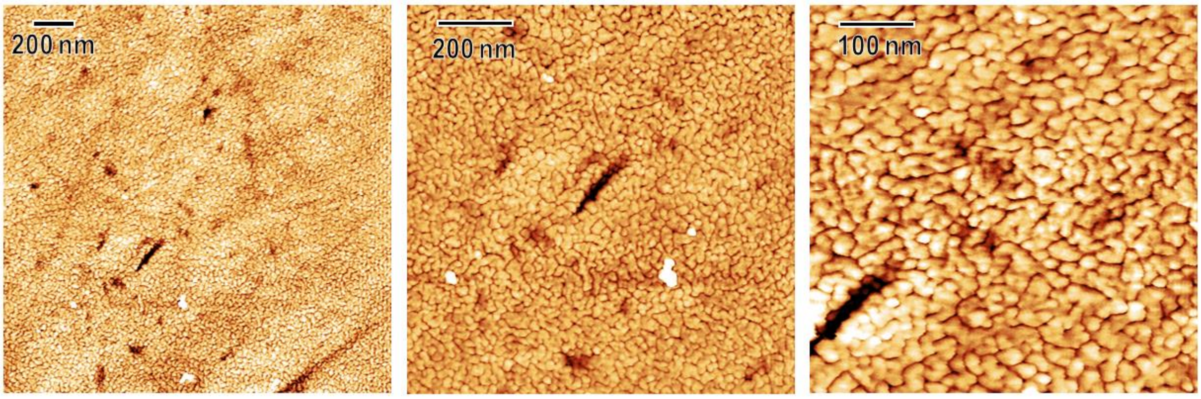

Figure S23. AFM visualization of a 5 nm gold film on a quartz substrate.

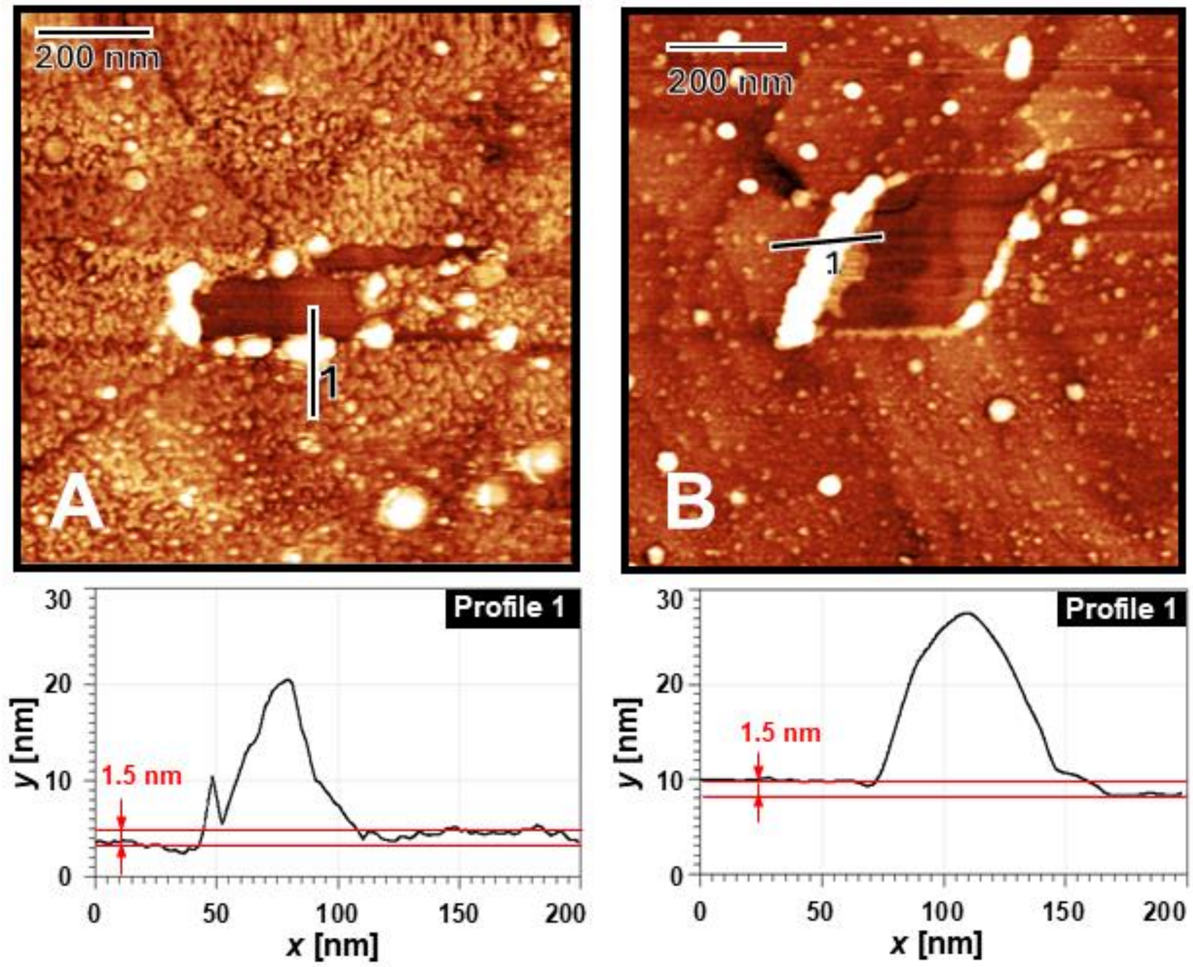

Figure S24. AFM visualization after scratching of SAMs made of **1A** (A) and **2A** (B) on gold on mica, and their respective topography chart along profile 1.

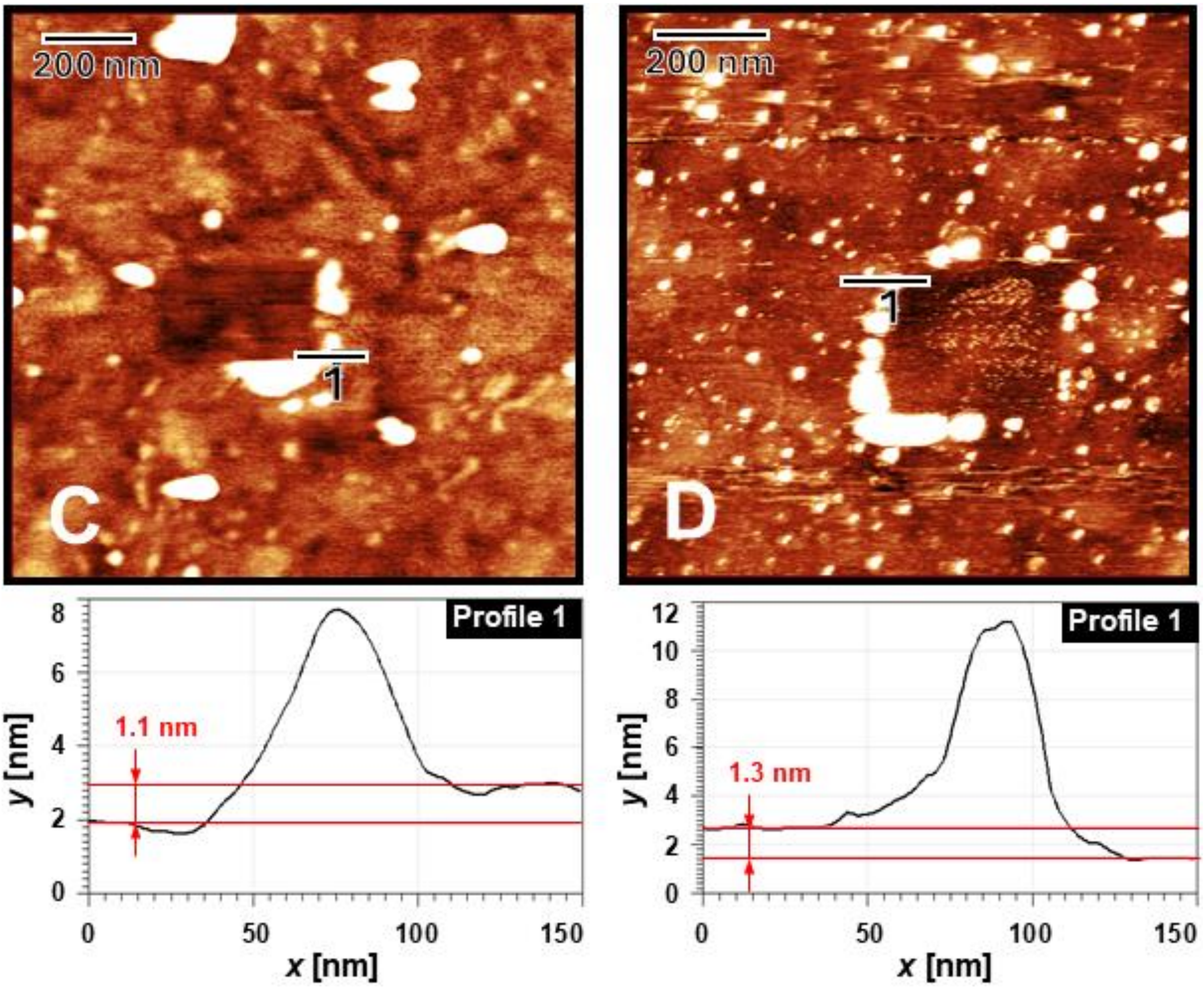

Figure S25. AFM visualization after scratching of SAMs made of stators **18** (C) and **19** (D) on gold on mica, and their respective topography chart along profile 1.

Table S7. Calculated and experimental Thickness determined by Ellipsometry and AFM.

Cmpd.	Monolayer Thickness (nm)		
	Calculated	Ellipsometry	AFM[a]
**1A**	1.6	1.6 ± 0.2	1.5 ± 0.2
**2A**	1.5	1.2 ± 0.2	1.3 ± 0.2
**18**	1.4	1.3 ± 0.1	1.2 ± 0.2
**19**	1.2	1.2 ± 0.2	1.2 ± 0.2

[a] Based on scratching experiment.

13. XPS Analysis of SAMs on Gold Surface

The surface composition of the samples was further studied with X-ray photoelectron spectroscopy (XPS) using SPECS spectrometer equipped with a monochromatic Al Kα X-ray source (1486.7 eV) and a hemispherical electron analyzer Phoibos 150. The survey spectra were recorded with E_p set to 100 eV, the high-resolution spectra of the C 1s core line with E_p set to 40 eV. The base chamber pressure during the acquisitions was at 10^{-9} mbar or lower. Due to a charge development on the samples' surface, a low-energy electron flow generated by an electron flood gun has been used to compensate the charge buildup (emission 10 µA, energy 1.0 eV). The spectra were referenced to the Au $4f_{7/2}$ energy 84.0 eV.

The survey spectrum of the sample is dominated by the signals that are assigned to electrons originating in the gold substrate (**Figure S26**). Besides these, also the signals assigned to the O 1s and Ti 2p electrons originating in the TiO_2 interlayer could be observed at 531.6 and 465.5/459.3 eV, respectively. To assess the interaction of the tetrapod molecule with gold surface, a parallel experiment involving the same reaction conditions as for the SAM preparation was carried out. The ratio of the C 1s signal intensity vs. Au $4d_{5/2}$ gives a good indication of a presence of additional source of the C 1s electrons in addition to the adventitious carbon contamination. While in the case of the 'blank' gold substrate is the C/Au ratio approximately 18:82, in the case of the SAM on gold is the C/Au ratio approximately 53:47. The presence of the sulfur-containing organic material on the surface of the gold film is also confirmed by a low-intensity signal at 162.4 eV assigned to the S 2p electrons of the -SMe groups of the tetrapod molecule coordinated to gold atoms. Due to the low intensity of this signal, the corresponding signal of the S 2s electrons is not observed.

There were four components identified in the high-resolution spectrum of the C 1s core line (**Figure S27**). They were tentatively assigned as originating from the sp^2 carbon atoms (284.6 eV), sp^3 carbon atoms (285.4 eV), C-S and/or C-O atoms (287.2 eV) and C=O atoms (289.3 eV). The oxygen-containing carbonaceous functional groups could have been introduced to the sample by the thermal annealing involving butane flame and/or from the environmental contamination.

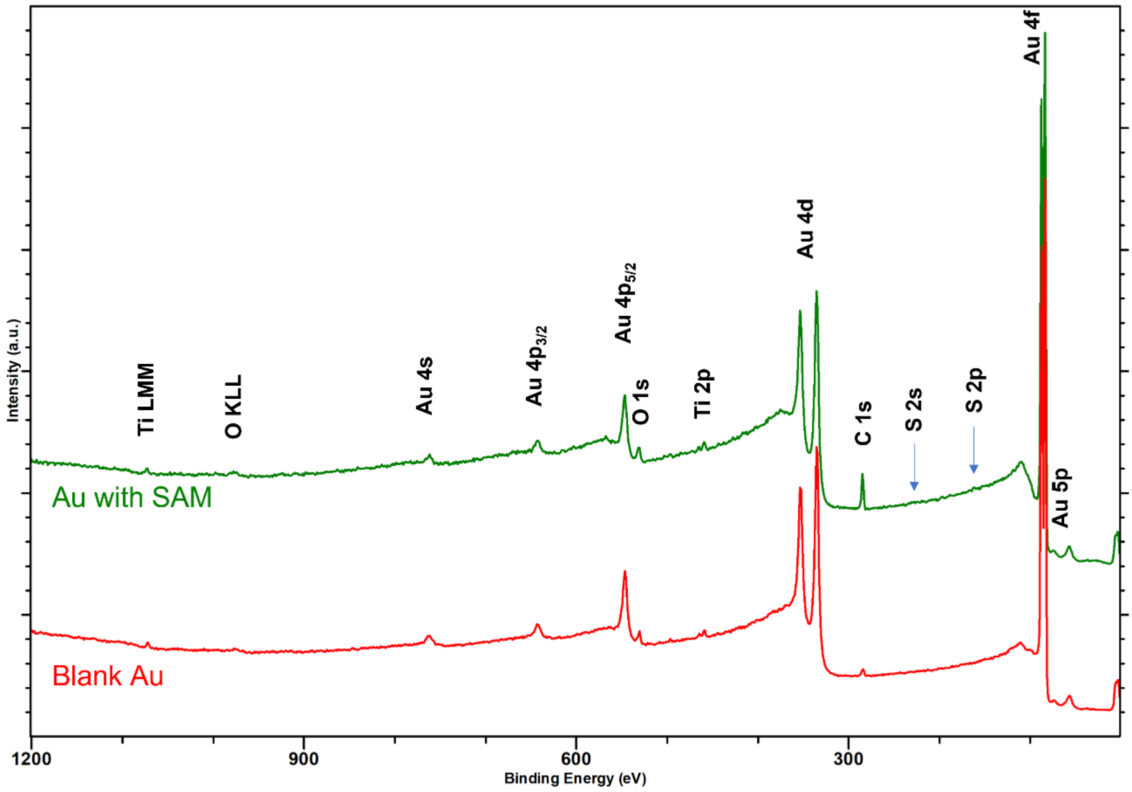

Figure S26. Survey photoelectron spectrum of **2A** on gold with SAM (green) and blank gold (red).

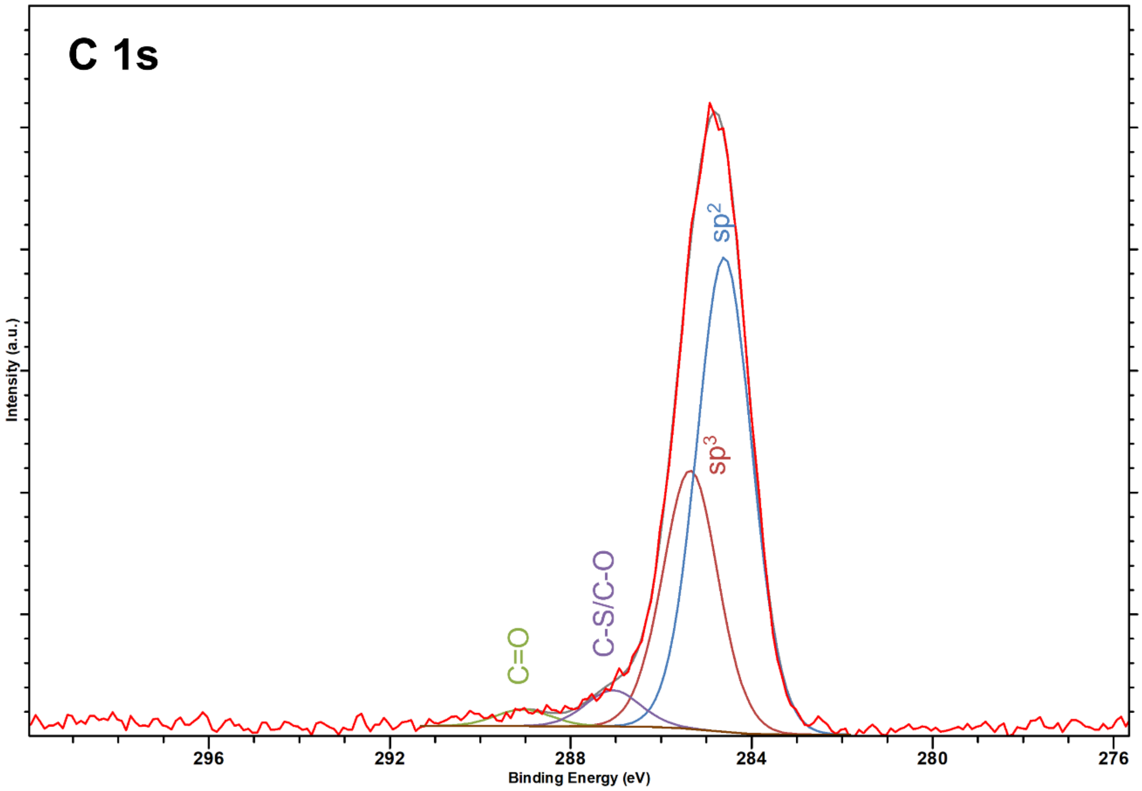

Figure S27. High-resolution spectrum of the C 1s photoelectrons of **2A**.

14. UV-vis Analysis of SAMs on Gold Surface

The same equipment as described in **Section 6** was used for the UV-vis analysis of monolayers on the semi-transparent Au films. The distance from the diodes to the cuvette is 15 mm. The substrate was placed in a quartz cuvette (10 × 2mm). All measurements were performed in *n*-decane, (used as an inert solvent to transfer heat from samples to the cooling unit) which was added to the substrate in the cuvette. The LED module (385±5 nm) was used for irradiation with the light flux of 0.394 W/cm^2 and the sample was irradiated for 20 s. Each kinetic dataset was independently collected for three different samples, with at least one temperature point re-measured for reproducibility on the same sample.

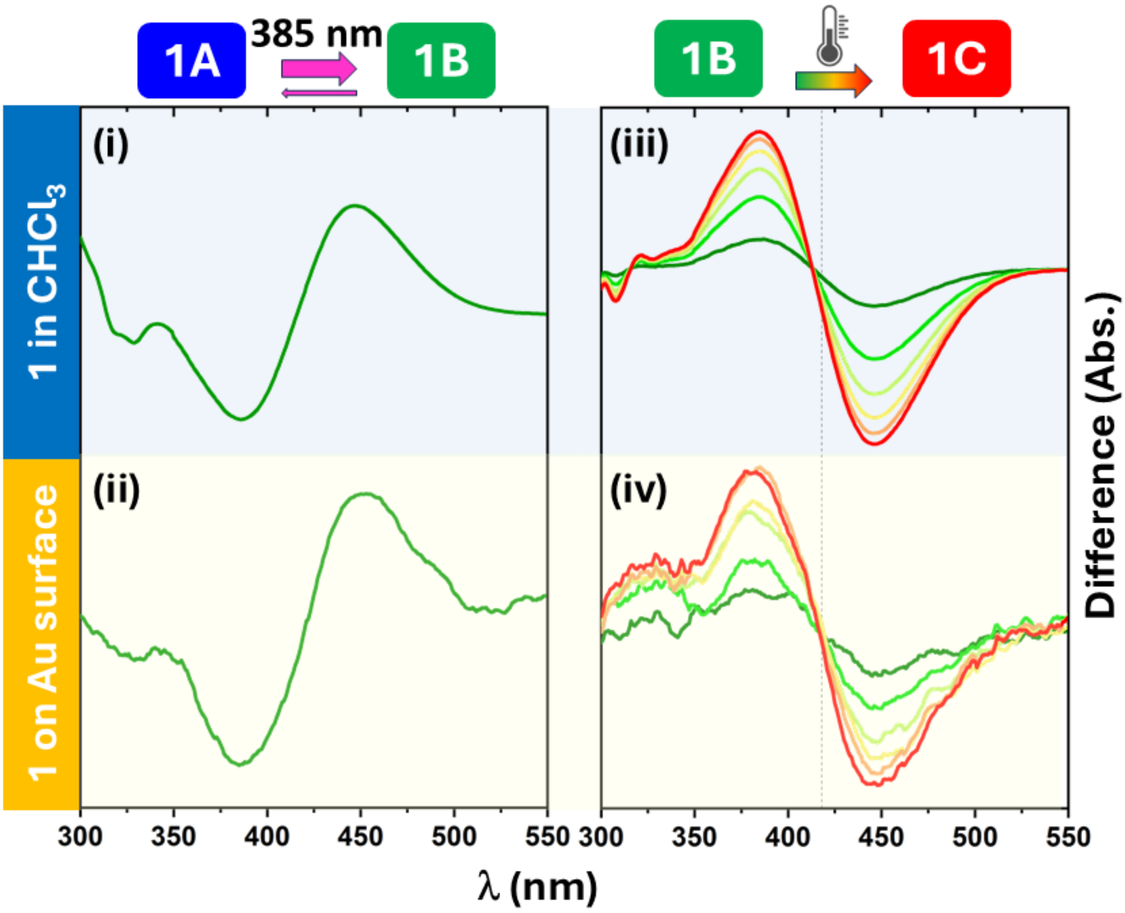

Figure S28. UV-vis differential absorption spectra of **1A** in chloroform solution (i) and within a SAM (ii) before and after illumination at 385±5 nm, recorded at 10 °C. The subsequent thermal relaxation of predominantly **1B** to **1C** in chloroform solution (iii) and in SAM (iv) at 10 °C is also observed through differential UV-vis absorption spectra.

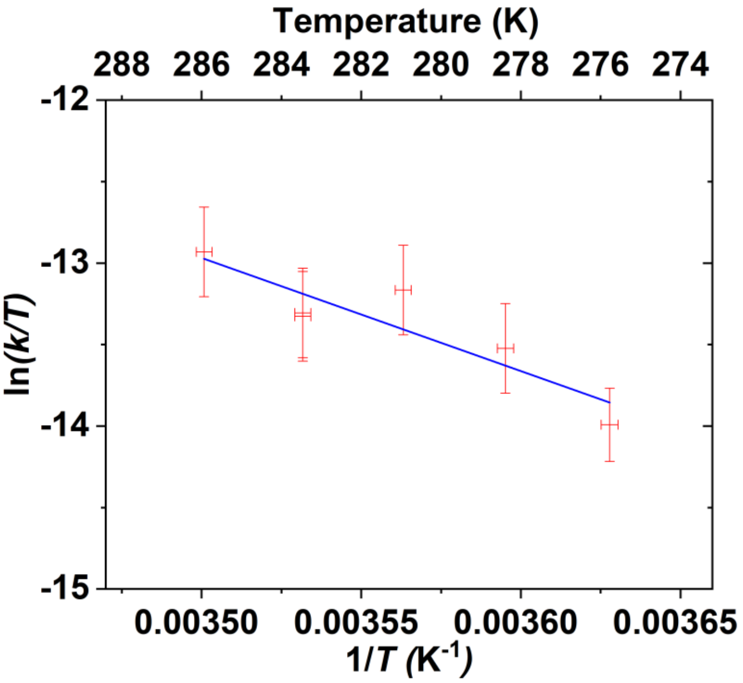

Figure S29. Eyring plot of motor **1A** in SAM on a semitransparent gold surface including error bars (red). In some cases, multiple sets of error bars are shown when data points were re-measured.

Table S8. Kinetic parameters of **1A** in SAM on a semitransparent gold surface at 20 °C extracted from Eyring plot.

Eyring equation	ln(k/T) = −6950.95/T + 11.36
R^2 value	0.8121
$\Delta H^{\ddagger}$	58 ± 21 kJ/mol
$\Delta G^{\ddagger}$	88 ± 40 kJ/mol
$\Delta S^{\ddagger}$	-103 ± 75 J/mol·K
k^0	$(1.27 \pm 0.30)\times10^{-3}$
$t_{1/2}$	9.1 ± 2.5 min

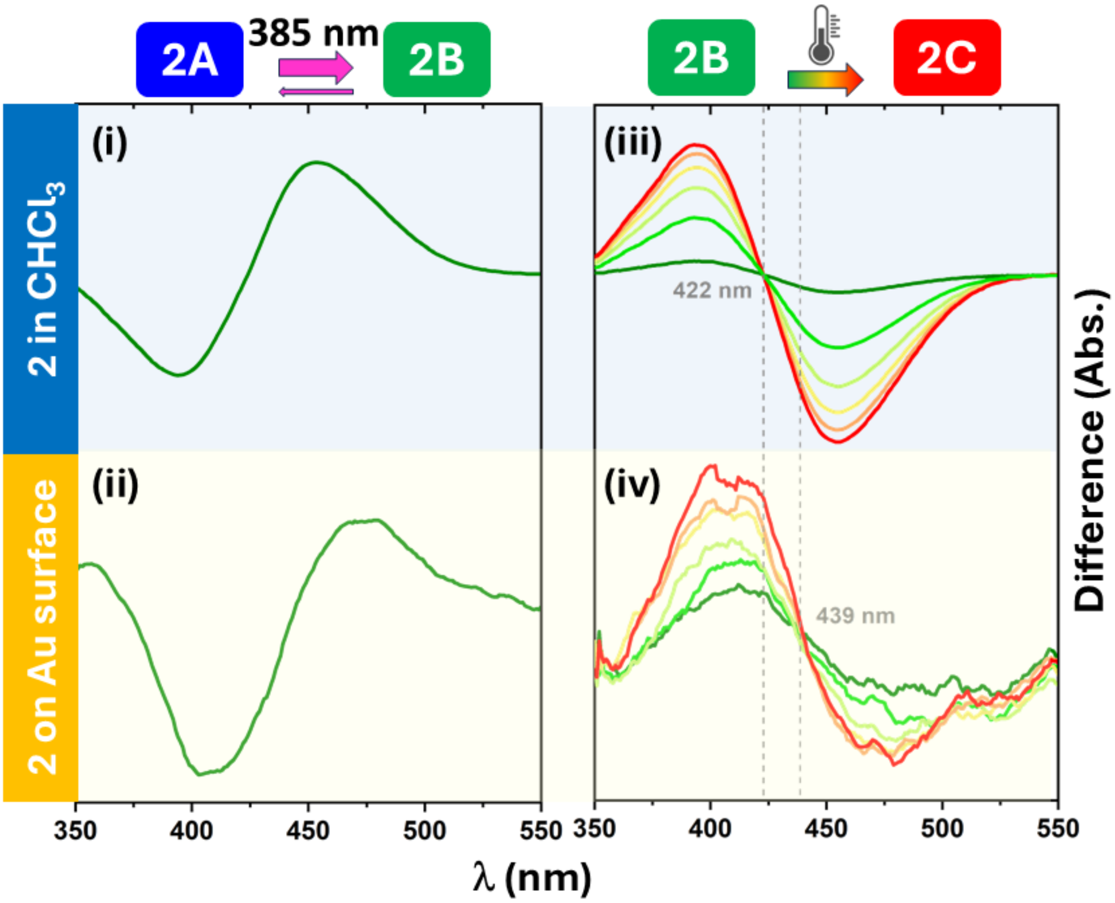

Figure S30. UV-vis differential absorption spectra of enriched **2A** in chloroform solution (i) and within a SAM (ii) before and after illumination at 385 ± 5 nm, recorded at 10 °C. The subsequent thermal relaxation of predominantly **2B** to **2C** in chloroform solution (iii) and in SAM (iv) at 10 °C is also observed through differential UV-vis absorption spectra.

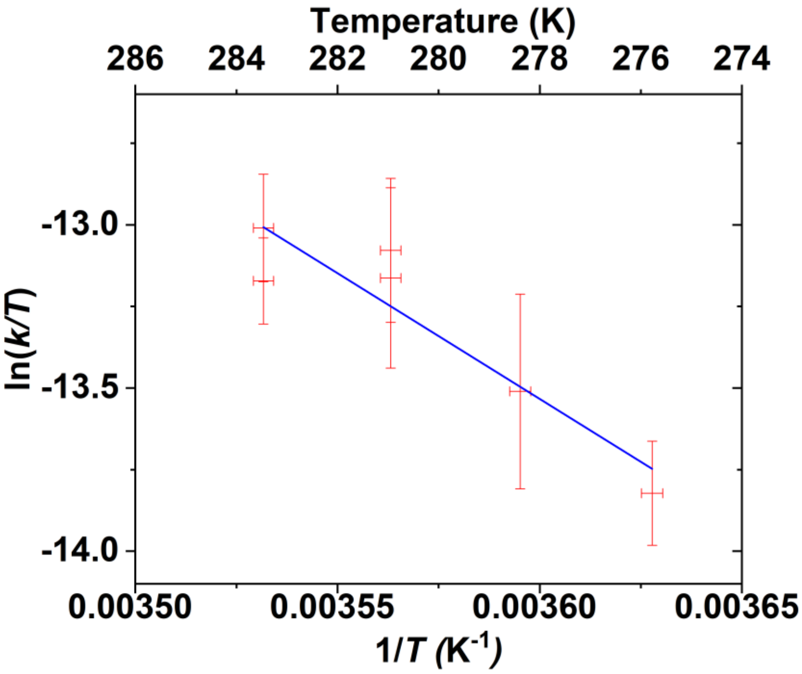

Figure S31. Eyring plot of motor **2A** in SAM including error bars (red). In some cases, multiple sets of error bars are shown when data points were re-measured.

Table S9. Kinetic parameters of **2A** in SAM on a semitransparent gold surface at 20 °C extracted from Eyring plot.

Eyring equation	ln(k/T) = −7708.58/T + 14.22
R^2 value	0.8562
$\Delta H^{\ddagger}$	64 ± 21 kJ/mol
$\Delta G^{\ddagger}$	87 ± 42 kJ/mol
$\Delta S^{\ddagger}$	-79 ± 74 J/mol·K
k^0	(1.67 ± 0.35)×10^{-3}
$t_{1/2}$	6.9 ± 1.5 min

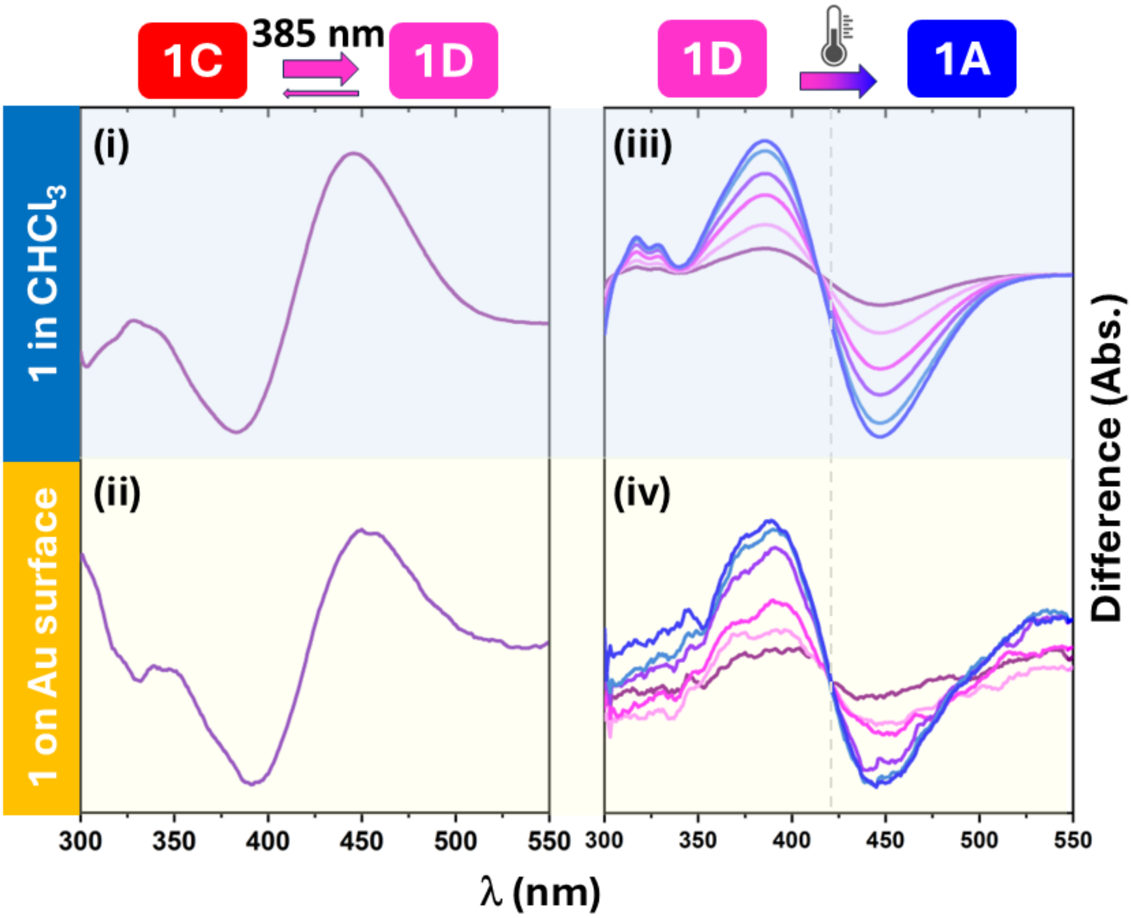

Figure S32. UV-vis differential absorption spectra of **1C** in chloroform solution (i) and within a SAM (ii) before and after illumination at 385±5 nm, recorded at 10 °C. The subsequent thermal relaxation of predominantly **1D** to **1A** in chloroform solution (iii) and in SAM (iv) at 10 °C is also observed through differential UV-vis absorption spectra.

15. Calculations

Density Functional Theory Calculation

In this work, all the density functional theory (DFT) calculations were conducted using the Vienna Ab initio Simulation Package (VASP 6.4.1).[54, 55] The projector augmented wave (PAW) method[56] and the generalized gradient approximation (GGA) functional of Perdew-Burke-Ernzerhof (PBE)[57] were employed. The plane wave cutoff energy was set to 500 eV and van der Waals interactions were considered using the DFT-D3 method.[58] The Brillouin zone was sampled with a 2×2×1 mesh for structural optimization and a 5×5×1 mesh for electronic property calculations, including the density of states (DOS) and electron density difference (EDD) calculations. The electronic energy and force convergence criteria were set to 10^{-4} eV and 0.01 eV/Å for self-consistent iteration and ionic relaxation, respectively. The adsorption energy was calculated using the following equation:

$$E_{ads} = E_{\mathbf{19}@Au(111)} - E_{\mathbf{19}+Au(111)}$$

where E_{ads} represents the adsorption energy, $E_{\mathbf{19}@Au(111)}$ represents the energy of adsorbed **19** on the Au(111) surface, and $E_{\mathbf{19}+Au(111)}$ represents the energy of the free tetrapodal anchor **19** and the Au(111) surface when they are far apart.

Ab initio Molecular Dynamics Simulation

The *ab initio* molecular dynamics (AIMD) simulation was conducted using the NVT canonical ensemble at 450 K. The Brillouin zone was sampled at the Γ point. To accelerate the AIMD simulation, an on-the-fly machine-learned force field (MLFF)[59, 60, 61] was employed. The total simulation time and time interval were set to 20 ps and 1 fs, respectively.

To further investigate the interaction between **19** and the gold surface, the density of states (DOS) of the system before (**Figure S33a**) and after adsorption (**Figure S33b**) was calculated. The DOS was projected onto the entire tetrapodal anchor molecule **19** and the four sulfur atoms. Before adsorption on the Au(111) surface, the electrons of the sulfur atoms were highly localized within the range from -0.2 to 0.5 eV. The peaks located at approximately 0 and 0.25 eV can be considered as the lone pair electrons of sulfur atoms, which are also highly spatially localized. After adsorption and interaction with the gold atoms, the valence band of **19** experienced a downshift, and the lone pair electron peaks disappeared. The states belonging to the sulfur atoms were dispersed across the energy scale, indicating that the states of sulfur atoms were highly hybridized with the d-bands of the gold atoms, forming S-Au dative bonds. Furthermore, we calculated the electron density difference (EDD) of the system before

[54] G. Kresse, J. Furthmüller, "Efficiency of Ab-Initio Total Energy Calculations for Metals and Semiconductors Using a Plane-Wave Basis Set" *Comput. Mater. Sci.* **1996**, *6* (1), 15-50.

[55] G. Kresse, J. Furthmüller, "Efficient Iterative Schemes for Ab Initio Total-Energy Calculations Using a Plane-Wave Basis Set" *Phys. Rev. B* **1996**, *54* (16), 11169-11186.

[56] P. E. Blöchl, "Projector Augmented-Wave Method" *Phys. Rev. B* **1994**, *50* (24), 17953-17979.

[57] J. P. Perdew, K. Burke, M. Ernzerhof, "Generalized Gradient Approximation Made Simple" *Phys. Rev. Lett.* **1996**, *77* (18), 3865-3868.

[58] S. Grimme, J. Antony, S. Ehrlich, H. Krieg, "A Consistent and Accurate Ab Initio Parametrization of Density Functional Dispersion Correction (DFT-D) for the 94 Elements H-Pu" *J. Chem. Phys.* **2010**, *132* (15).

[59] R. Jinnouchi, J. Lahnsteiner, F. Karsai, G. Kresse, M. Bokdam, "Phase Transitions of Hybrid Perovskites Simulated by Machine-Learning Force Fields Trained on the Fly with Bayesian Inference" *Phys. Rev. Lett.* **2019**, *122* (22), 225701.

[60] R. Jinnouchi, F. Karsai, G. Kresse, "On-the-Fly Machine Learning Force Field Generation: Application to Melting Points" *Phys. Rev. B* **2019**, *100* (1), 014105.

[61] R. Jinnouchi, F. Karsai, C. Verdi, R. Asahi, G. Kresse, "Descriptors Representing Two-and Three-Body Atomic Distributions and Their Effects on the Accuracy of Machine-Learned Inter-Atomic Potentials" *J. Chem. Phys.* **2020**, *152* (23).

and after the S-Au interactions for visualization. **Figure S33c** presents the 2D EDD plot of the four S-Au interactions, where the red and blue lines represent electron accumulation and depletion, respectively. All four S-Au interactions exhibit electron redistribution: the electron densities at the upper and lower sides of the sulfur and gold atoms decrease, while the electron densities at the middle of the sulfur and gold atoms increase. The electron depletion regions correspond to the p_z orbital of sulfur and the d_{z^2} orbital of gold, verifying that the lone pair electrons of sulfur fill in the S-Au binding orbital, forming the dative bond. Interestingly, the level of electron redistribution among the four S-Au interactions was different. Two out of the four S-Au interactions showed strong interactions, with S-Au distances of 2.696 and 2.657 Å, evident from the dense contour lines in the EDD plot. The other two interactions were moderate and weak due to their longer interaction distances of 2.855 and 3.058 Å, respectively. Their interaction strength is also reflected in their moderate and sparse contour lines, respectively.

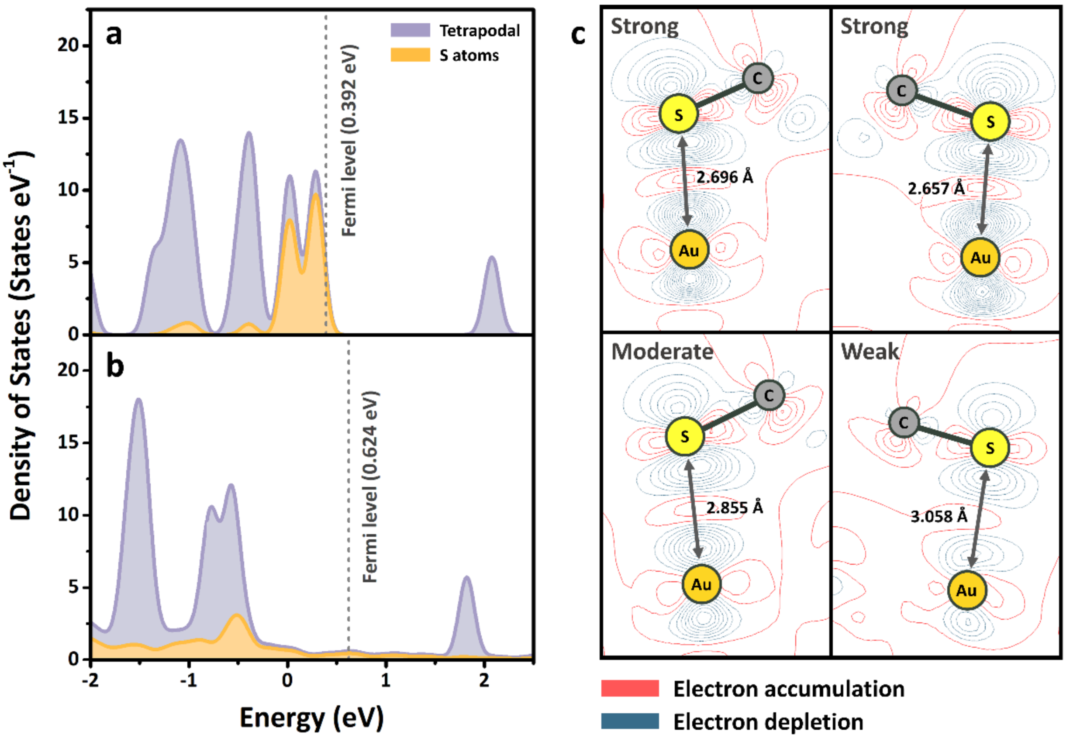

Figure S33. Projected density of states (PDOS) of the tetrapodal anchor **19** and sulfur atoms: (a) Before and (b) after adsorption on Au(111) surfaces. The black dotted vertical line represents the Fermi level. (c) 2D electron density difference (EDD) plots for the adsorption of the tetrapodal anchor on the Au(111) surface.

Additionally, the stability of **19** on the Au(111) surface by conducting *ab initio* molecular dynamics (AIMD) simulations was explored. After 20 ps of simulation, the energy approached a constant with small oscillations, implying that the system reached thermal equilibrium (**Figure S34**). The z-coordinate variation of the four sulfur atoms during the simulation is depicted in **Figure S35**. Generally, the variation of the z-coordinates within 2 Å (compared to their initial values) in the presence of the thermal motions of both the gold surface and the tetrapodal indicates strong interactions between the sulfur and gold atoms, even at the high simulation temperature of 450 K. However, we observed a dramatic rise in the z-coordinate of one sulfur atom at approximately 15 ps, with the variation reaching 4.5 Å compared to its initial value. It indicated the cleavage of one S-Au dative bond. Nevertheless,

this broken S-Au bond reformed at approximately 17.5 ps, showing the dynamic nature of the S-Au interaction and excellent thermal stability.

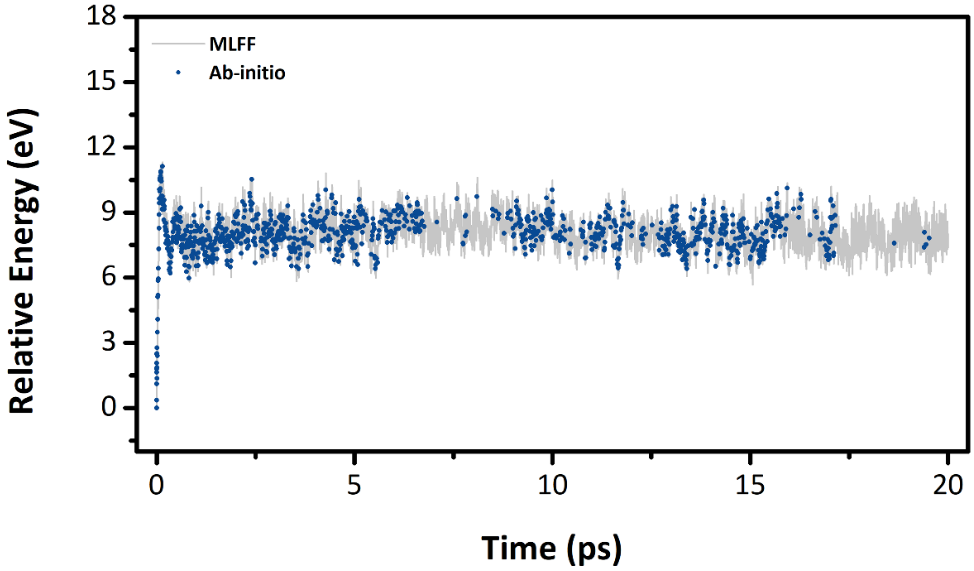

Figure S34. Energy plot over the simulation time for tetrapodal anchor **19** on Au(111) surface.

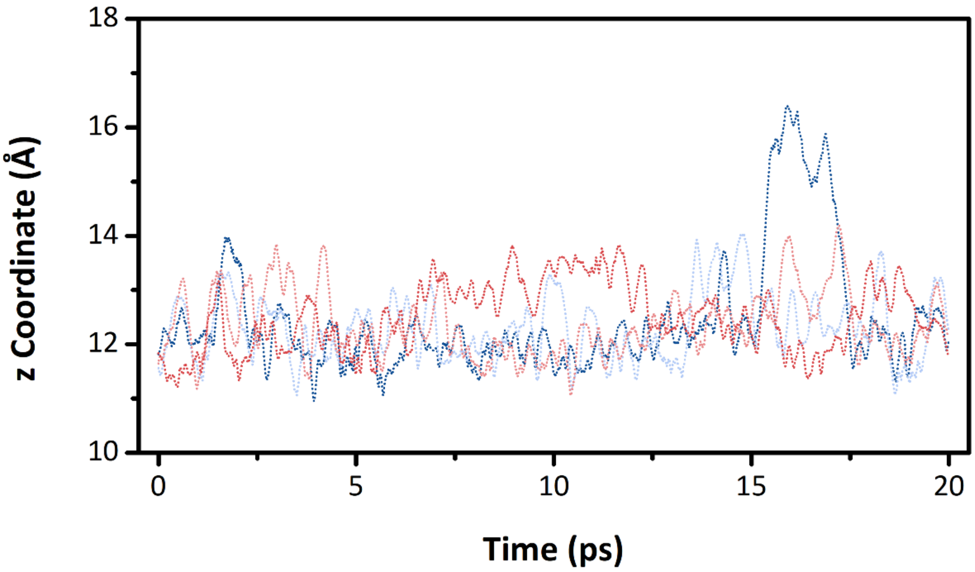

Figure S35. Z-direction trajectory variations of sulfur atoms on Au(111) surface during the simulation.

In summary, the DFT results show that all the sulfur atoms interacted with gold atoms, albeit with varying strengths. The unequal S-Au interaction strengths are attributed to the introduced stress after adsorption on the Au(111) surface, with a deformation energy of 0.616 eV. This stress results from the compromise between the spatial alignment of the S and Au atoms and the deformation of the tetrapodal anchor. Conversely, during the AIMD simulation with injected thermal energy, the S-Au bonds can be broken, and sulfur atoms can detach from the surface. However, the cleavage of one S-Au bond does not cause the desorption of the tetrapodal anchor, as the broken S-Au bond recovers after a certain time interval due to the forces exerted by the remaining three S-Au bonds, which keep the molecule tethered to the surface. The simulation results reveal the dynamic nature of S-Au dative bonds, suggesting that introducing more contact points (more thioether segments) helps prevent the desorption of **19**. Additionally, the diffusion and rearrangement of the adsorbed **19** are possible through a sequence of S-Au bond-breaking and re-bonding events.

Atropisomers and Anisotropic IR Spectra Calculations

All calculations were performed using the Gaussian 16, Revision C.01 program package.[62] To simulate anisotropic IR spectra, we optimized the molecular geometries in the gas phase at the B3LYP-D3BJ/6-311+G(d,p) level of theory[63,64] and applied a scaling factor of 0.9567 to the calculated harmonic frequencies, following our previous work.[65]

For atropisomers *R*-**15** and *S*-**15**, geometry optimizations and frequency calculations were carried out at the B3LYP-D3BJ/6-31+G* level of theory in DMSO as a solvent, employing the SMD solvation model.[66] Single-point energies were recalculated using a larger basis set (B3LYP-D3BJ/6-311++G(2d,p)) also employing the SMD solvation model for DMSO. The global minimum was obtained by optimizing the X-ray geometry and was confirmed by optimizing the alternative conformation.

For atropisomer conversion (racemization), two transition states were identified (**Figure S36**). The lower-energy transition state, which determines the process, has $\Delta G^{\neq}$ = 83 kJ/mol (19.9 kcal/mol) at 25 °C. The experimental barrier obtained from variable-temperature ^{1}H NMR measurements (**Figure S37**) is 80 kJ/mol (19.0 kcal/mol), corresponding to a racemization half-life of 5 s at 25 °C. The second transition state has $\Delta G^{\neq}$ = 110 kJ/mol (26.3 kcal/mol) at 25 °C.

---

[62] Gaussian 16, Revision C.01, Frisch, M. J.; Trucks, G. W.; Schlegel, H. B.; Scuseria, G. E.; Robb, M. A.; Cheeseman, J. R.; Scalmani, G.; Barone, V.; Petersson, G. A.; Nakatsuji, H.; Li, X.; Caricato, M.; Marenich, A. V.; Bloino, J.; Janesko, B. G.; Gomperts, R.; Mennucci, B.; Hratchian, H. P.; Ortiz, J. V.; Izmaylov, A. F.; Sonnenberg, J. L.; Williams-Young, D.; Ding, F.; Lipparini, F.; Egidi, F.; Goings, J.; Peng, B.; Petrone, A.; Henderson, T.; Ranasinghe, D.; Zakrzewski, V. G.; Gao, J.; Rega, N.; Zheng, G.; Liang, W.; Hada, M.; Ehara, M.; Toyota, K.; Fukuda, R.; Hasegawa, J.; Ishida, M.; Nakajima, T.; Honda, Y.; Kitao, O.; Nakai, H.; Vreven, T.; Throssell, K.; Montgomery, J. A., Jr.; Peralta, J. E.; Ogliaro, F.; Bearpark, M. J.; Heyd, J. J.; Brothers, E. N.; Kudin, K. N.; Staroverov, V. N.; Keith, T. A.; Kobayashi, R.; Normand, J.; Raghavachari, K.; Rendell, A. P.; Burant, J. C.; Iyengar, S. S.; Tomasi, J.; Cossi, M.; Millam, J. M.; Klene, M.; Adamo, C.; Cammi, R.; Ochterski, J. W.; Martin, R. L.; Morokuma, K.; Farkas, O.; Foresman, J. B.; Fox, D. J. Gaussian, Inc., Wallingford CT, 2016.

[63] P. J. Stephens, F. J. Devlin, C. F. Chabalowski, M. J. Frisch, "Ab Initio Calculation of Vibrational Absorption and Circular Dichroism Spectra Using Density Functional Force Fields" *J.Phys.Chem.* **1994**, *98*, 11623–11627.

[64] S. Grimme, S. Ehrlich, L. Goerigk, "Effect of the Damping Function in Dispersion Corrected Density Functional Theory" *J. Comp. Chem.* **2011**, *32*, 1456–1465.

[65] I. Rončević, E. Kaletová, K. Varga, I. Císařová, Z. Bastl, J.-C. Jiang, J. Kaleta, "Molecular Bending - An Important Factor Affecting the Packing of Self-Assembled Monolayers of Triptycene-Based Molecular Rods on a (111) Gold Surface" *J. Phys. Chem. C* **2022**, *126*, 7193–7207.

[66] Marenich, A. V.; Cramer, C. J.; Truhlar, D. G. Universal Solvation Model Based on Solute Electron Density and a Continuum Model of the Solvent Defined by the Bulk Dielectric Constant and Atomic Surface Tensions. *J. Phys. Chem. B* **2009**, *113*, 6378-6396.

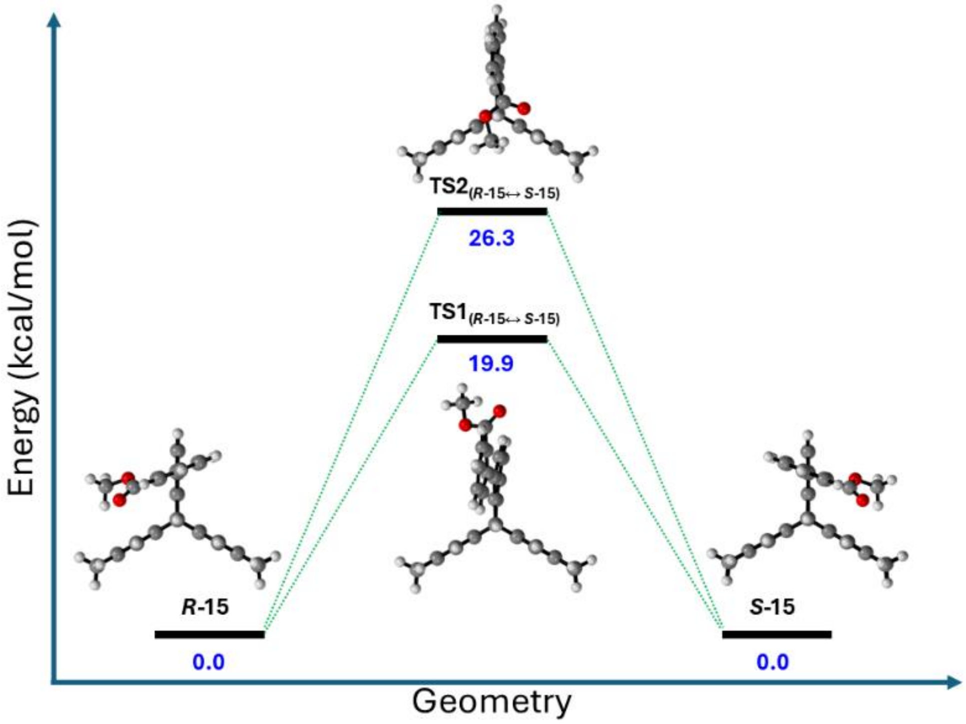

Figure S36. Visualization of atropisomers ***S*-15** and ***R*-15** and two possible transition states including their relative energies (kcal/mol).

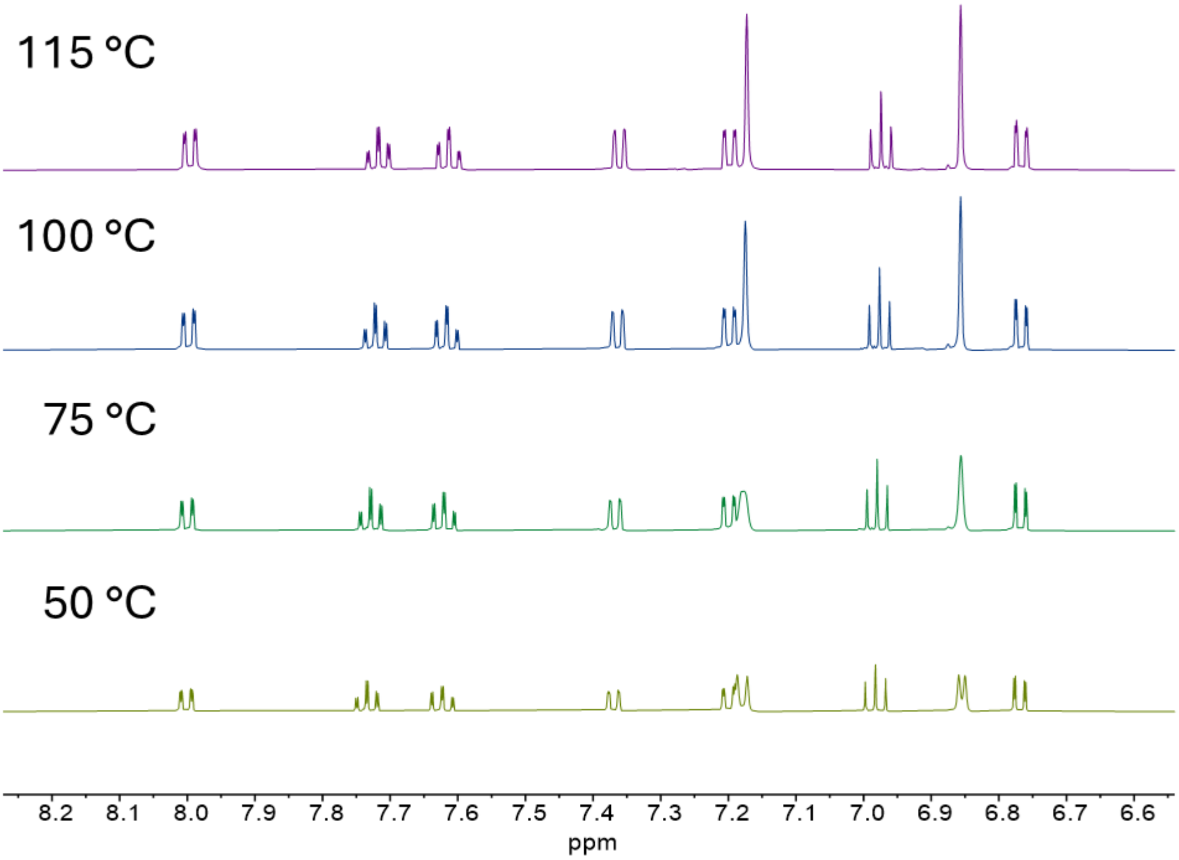

Figure S37. Variable-temperature ^{1}H NMR spectra used to determine the experimental value of transition state energy for interconversion of atropisomers ***S*-15** and ***R*-15**.

16. Assignments of ^{1}H and ^{13}C NMR Signals

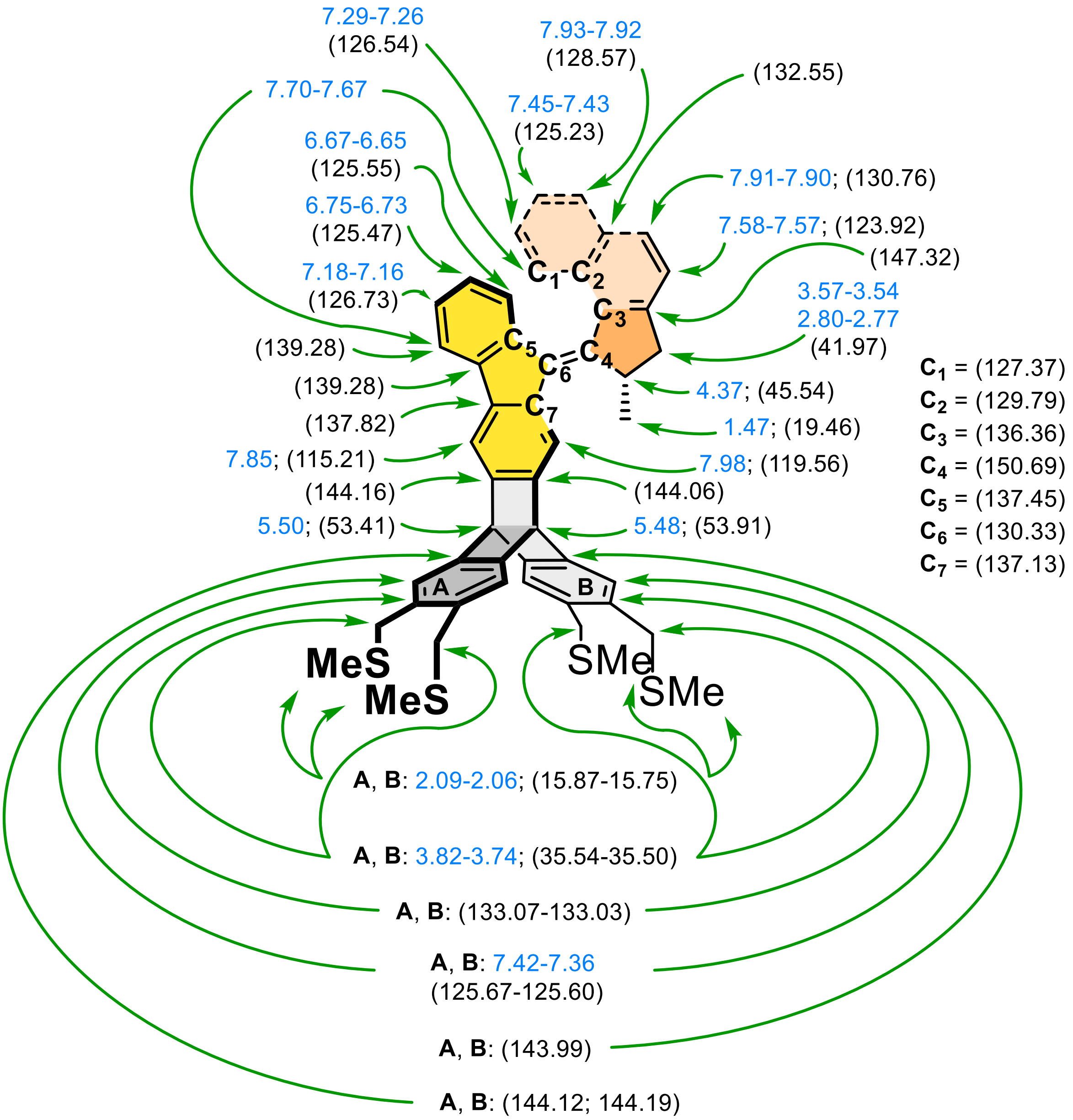

Figure S38: Assignment of ^{1}H and ^{13}C NMR signals for compound **1A** in $CDCl_3$ at 20 °C. Chemical shift values are reported in ppm.

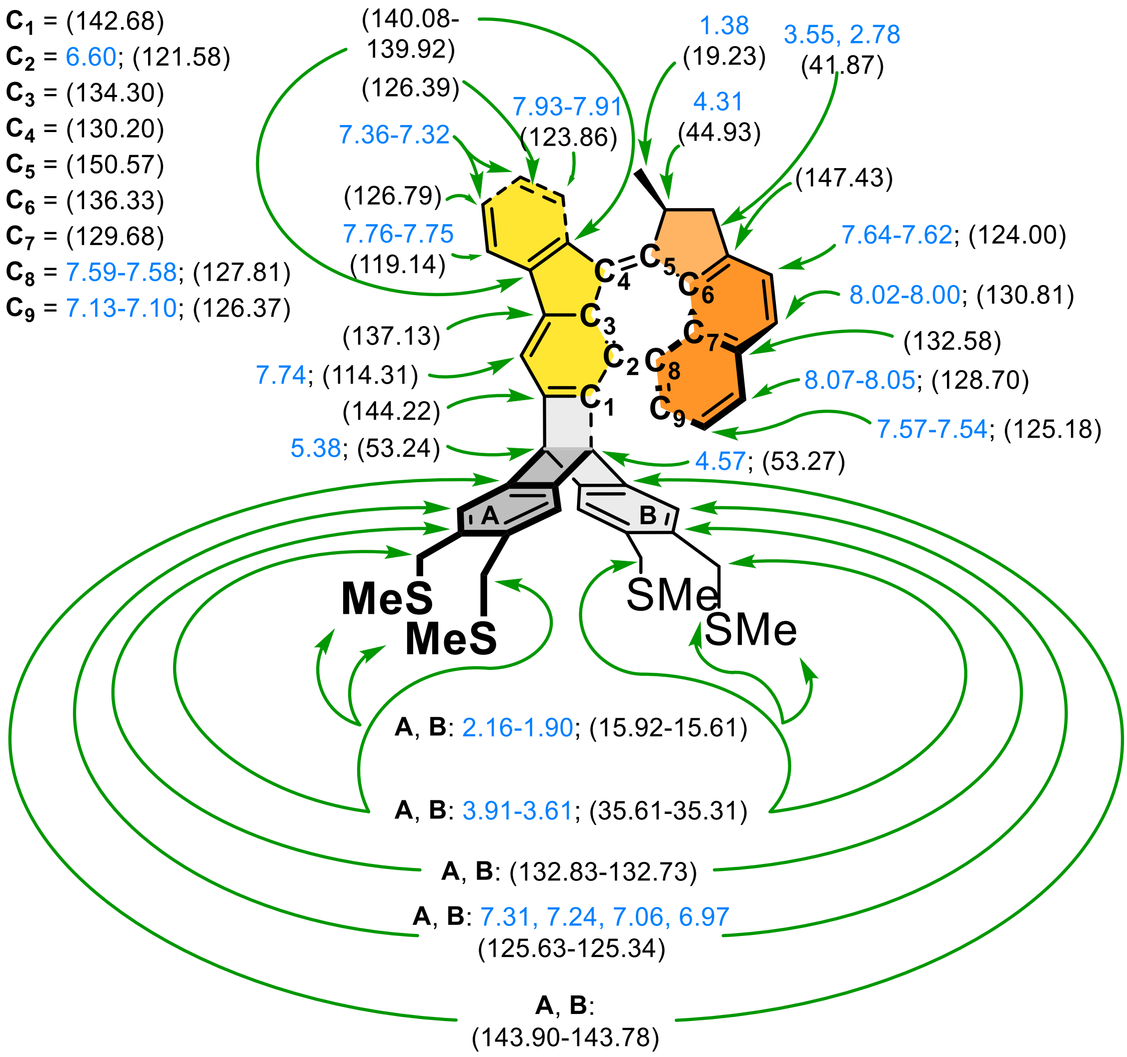

Figure S39: Assignment of ^{1}H and ^{13}C NMR signals for compound **1C** in $CDCl_3$ at 20 °C. Chemical shift values are reported in ppm.

17. NMR Spectra of Prepared Compounds

^{1}H NMR (600 MHz, $CDCl_3$): Compound **1A**

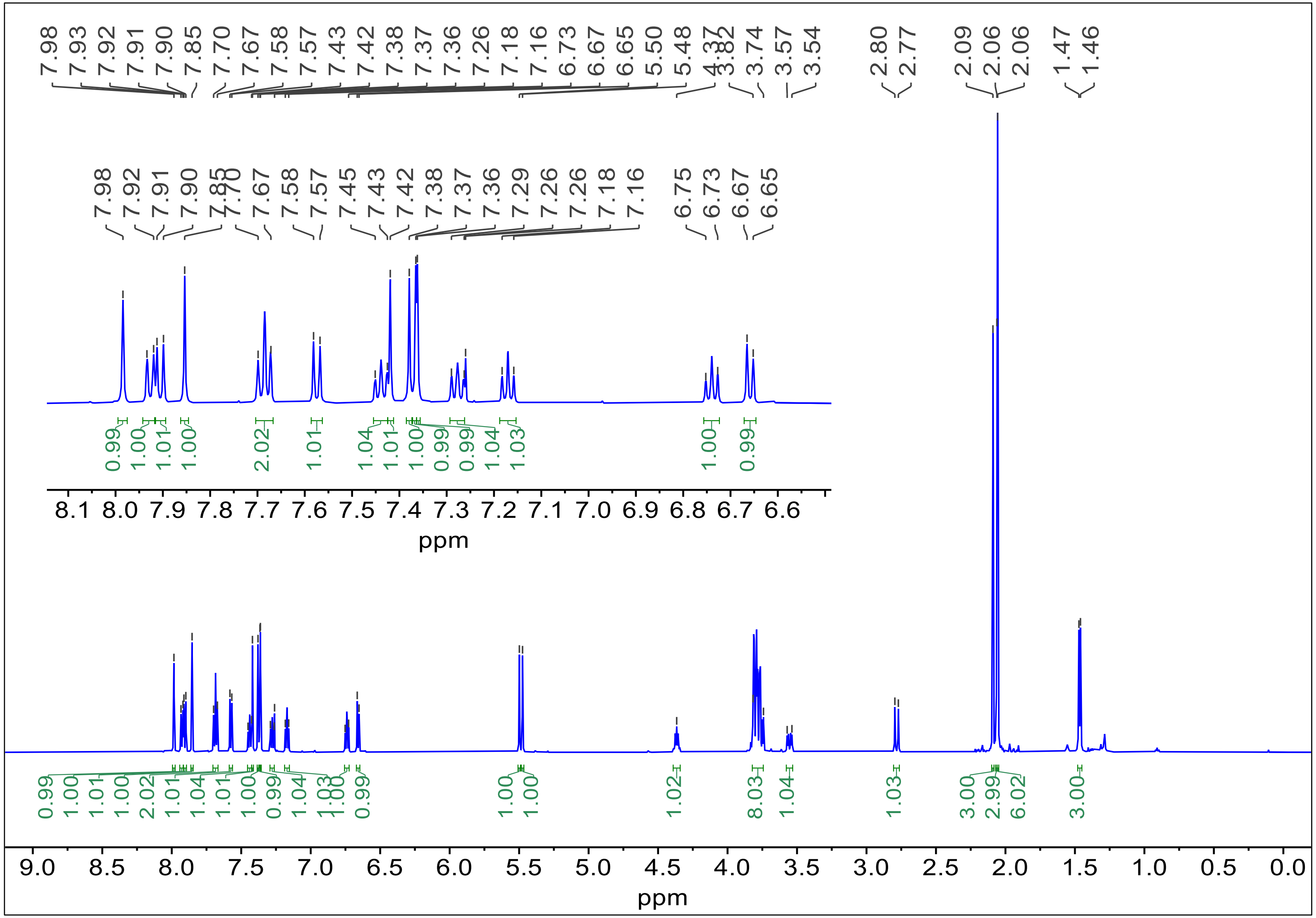

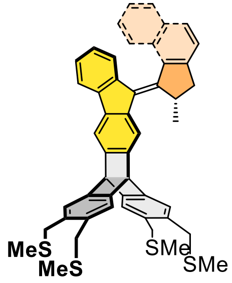

$^{13}C\{^{1}H\}$ APT NMR (150 MHz, $CDCl_3$): Compound **1A**

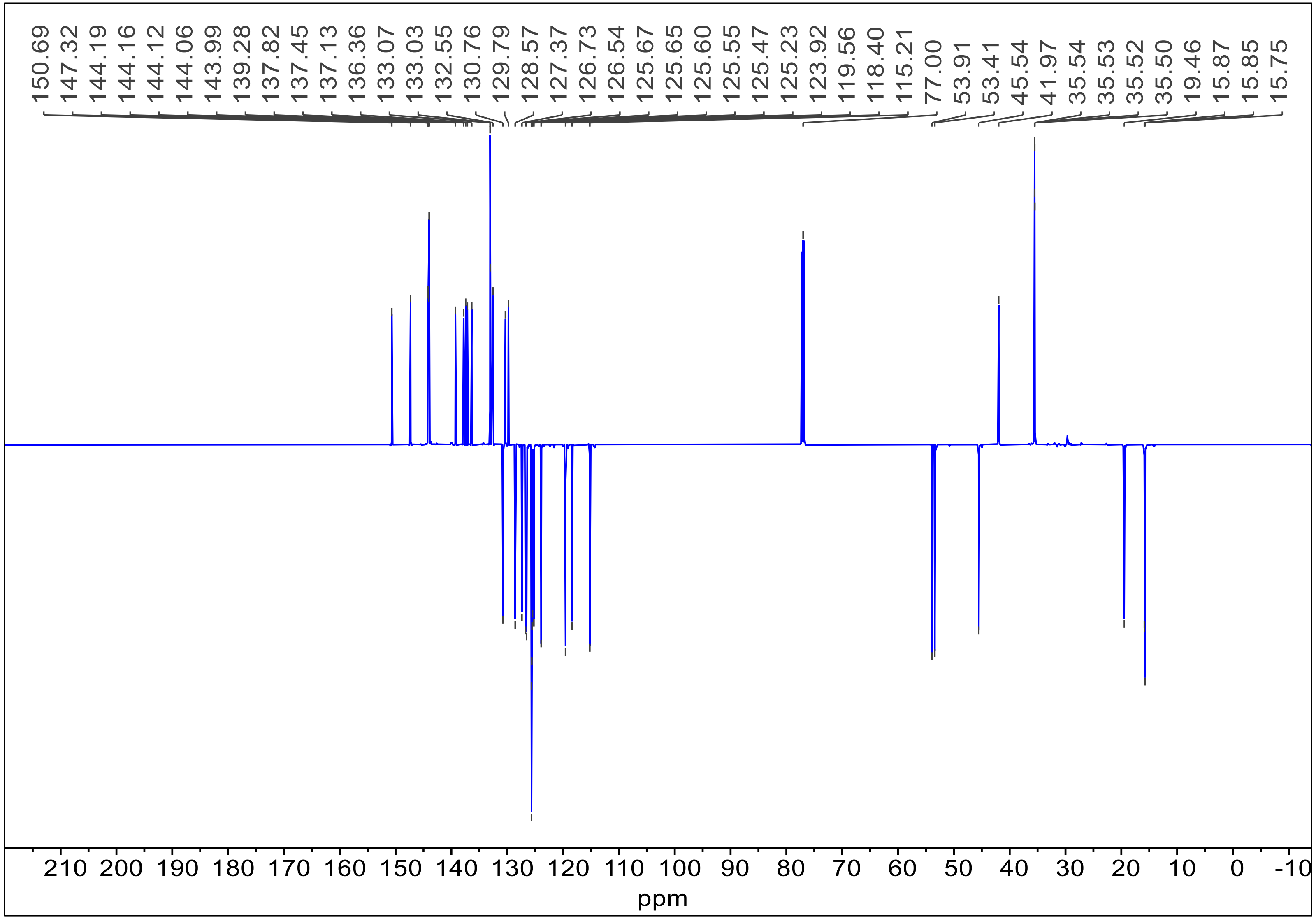

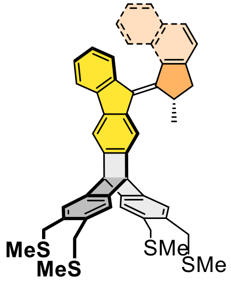

^{1}H – ^{1}H COSY ($CDCl_3$): Compound **1A**

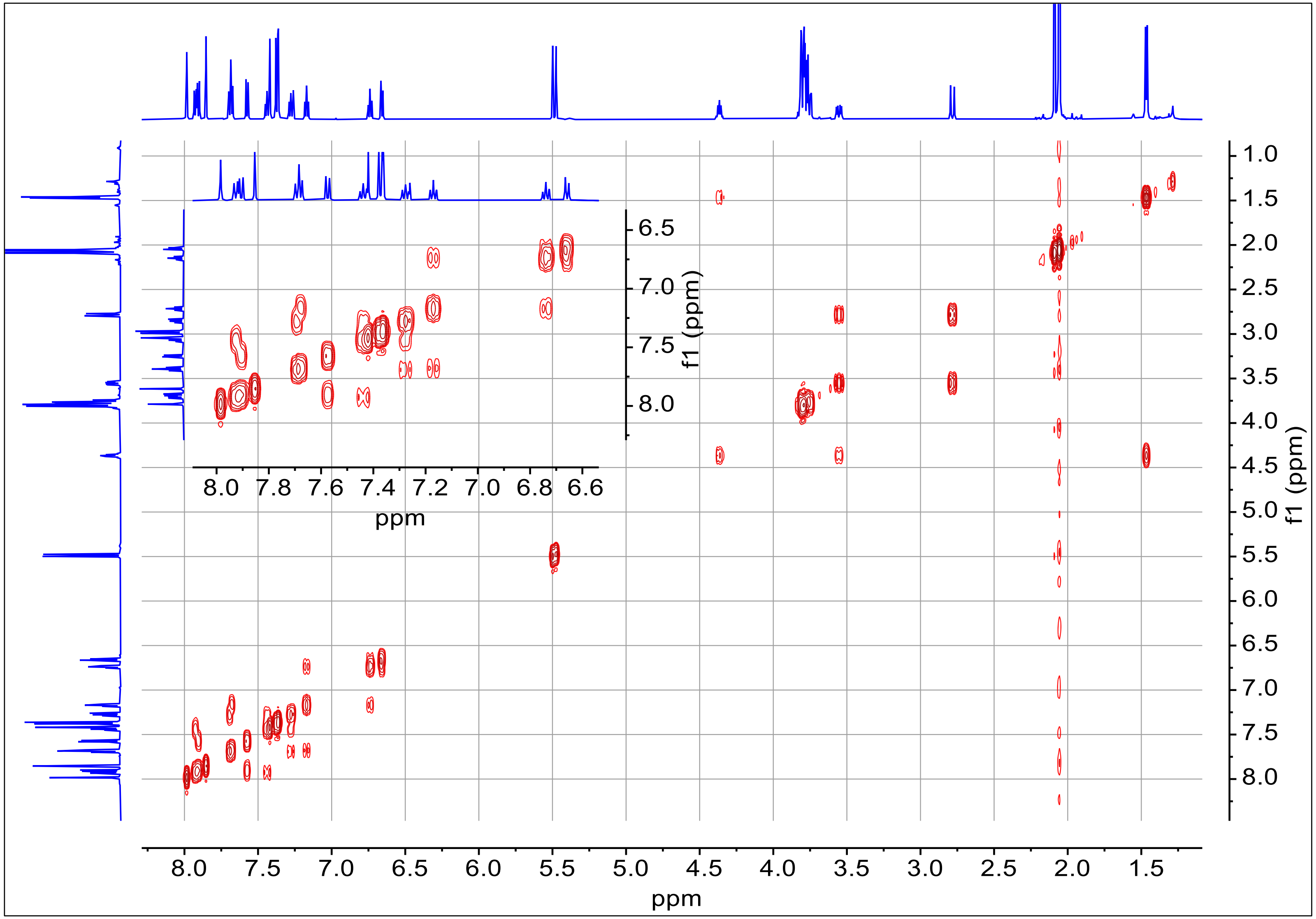

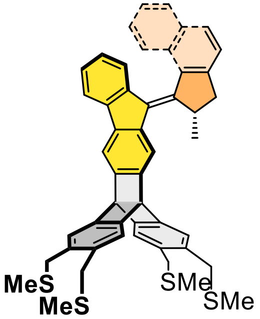

HSQC ($CDCl_3$): Compound **1A**

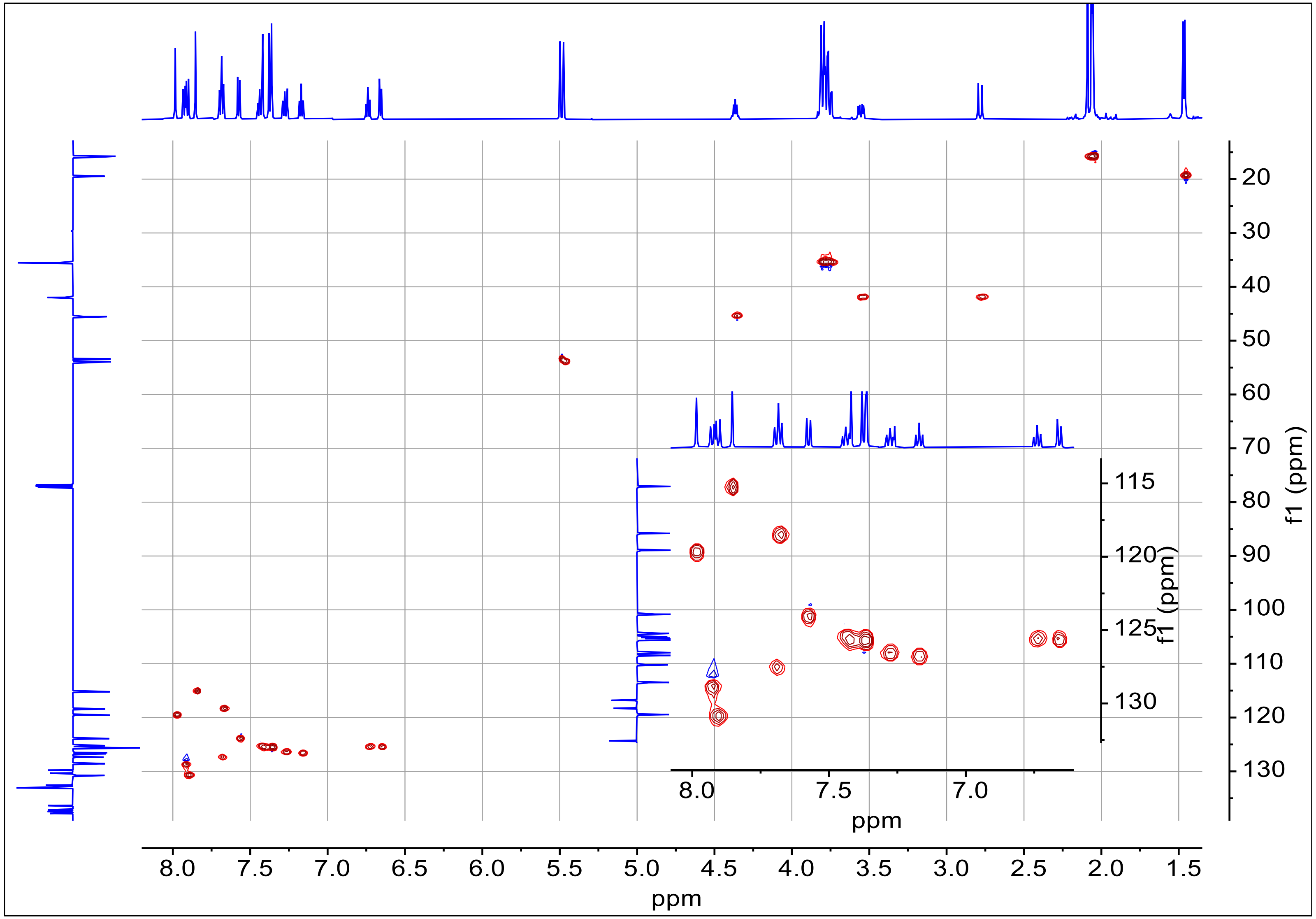

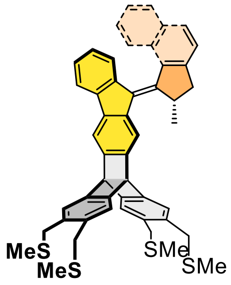

HMBC ($CDCl_3$): Compound **1A**

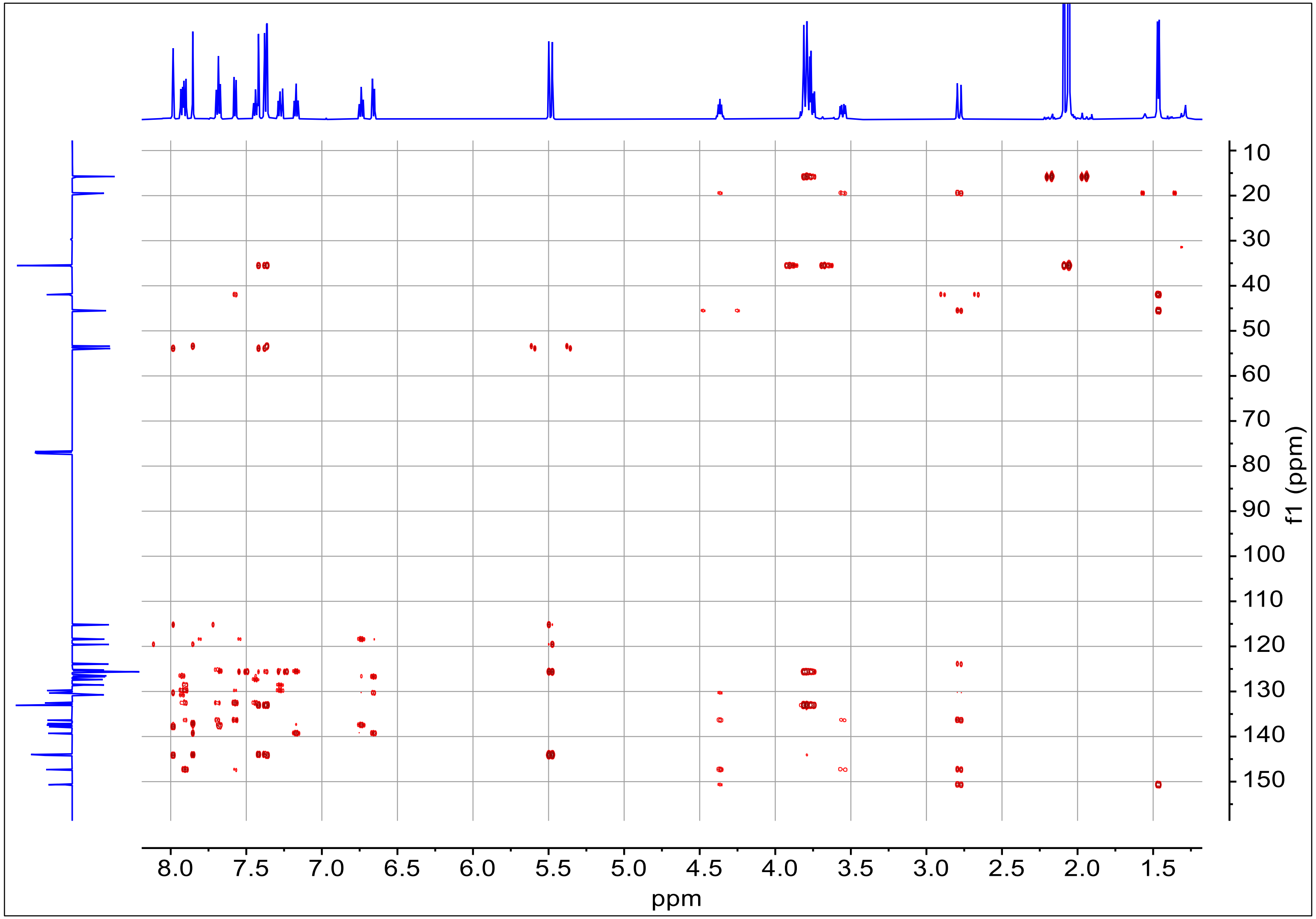

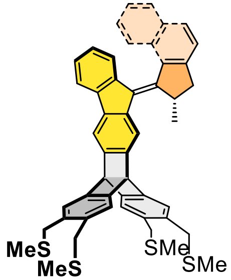

^{1}H NMR (500 MHz, $CDCl_3$): Compound **1B** (obtained after irradiation of **1A** with 385 ± 5 nm at -20 °C)

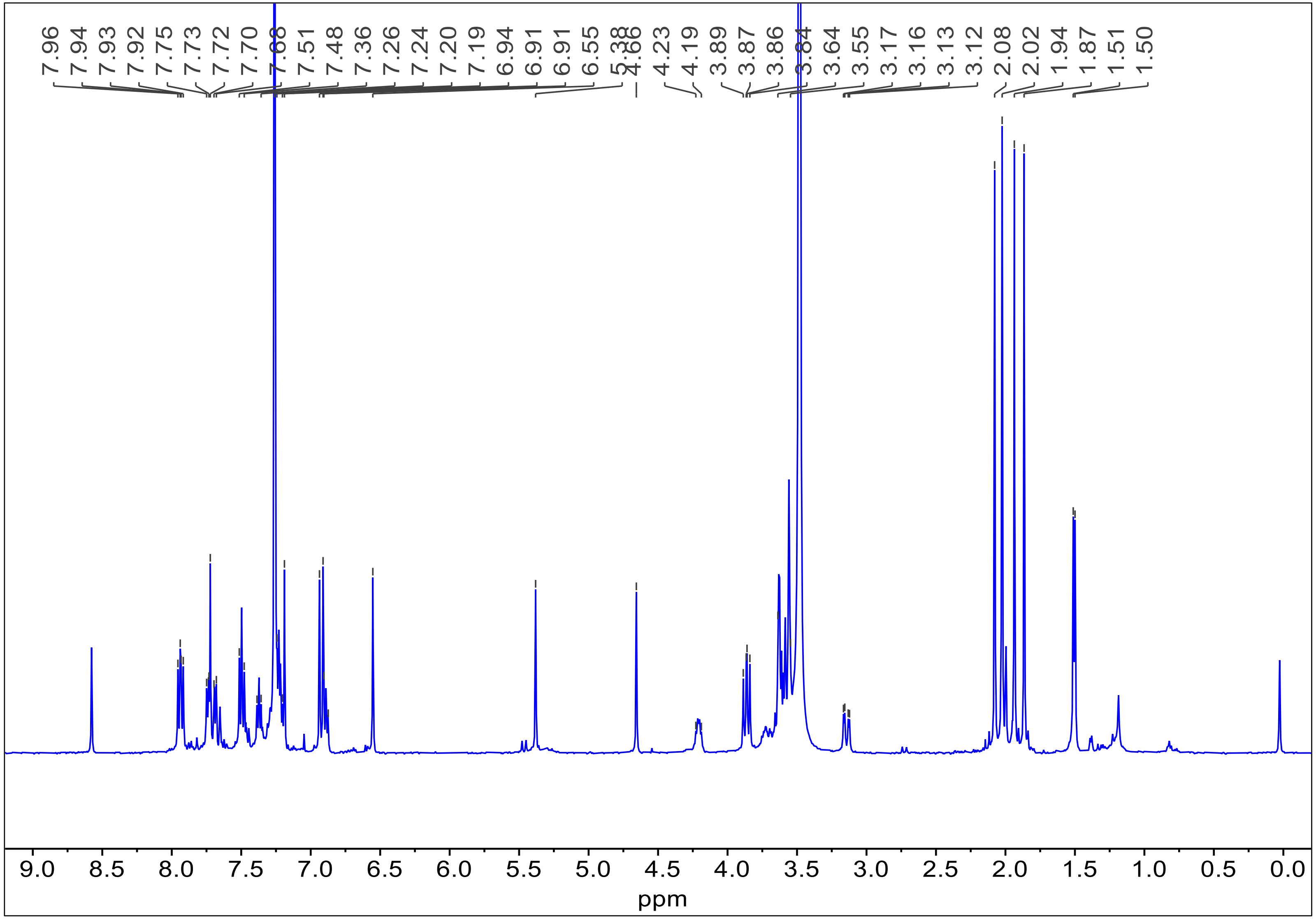

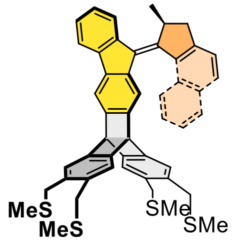

^{1}H NMR (600 MHz, $CDCl_3$): Compound **1C**

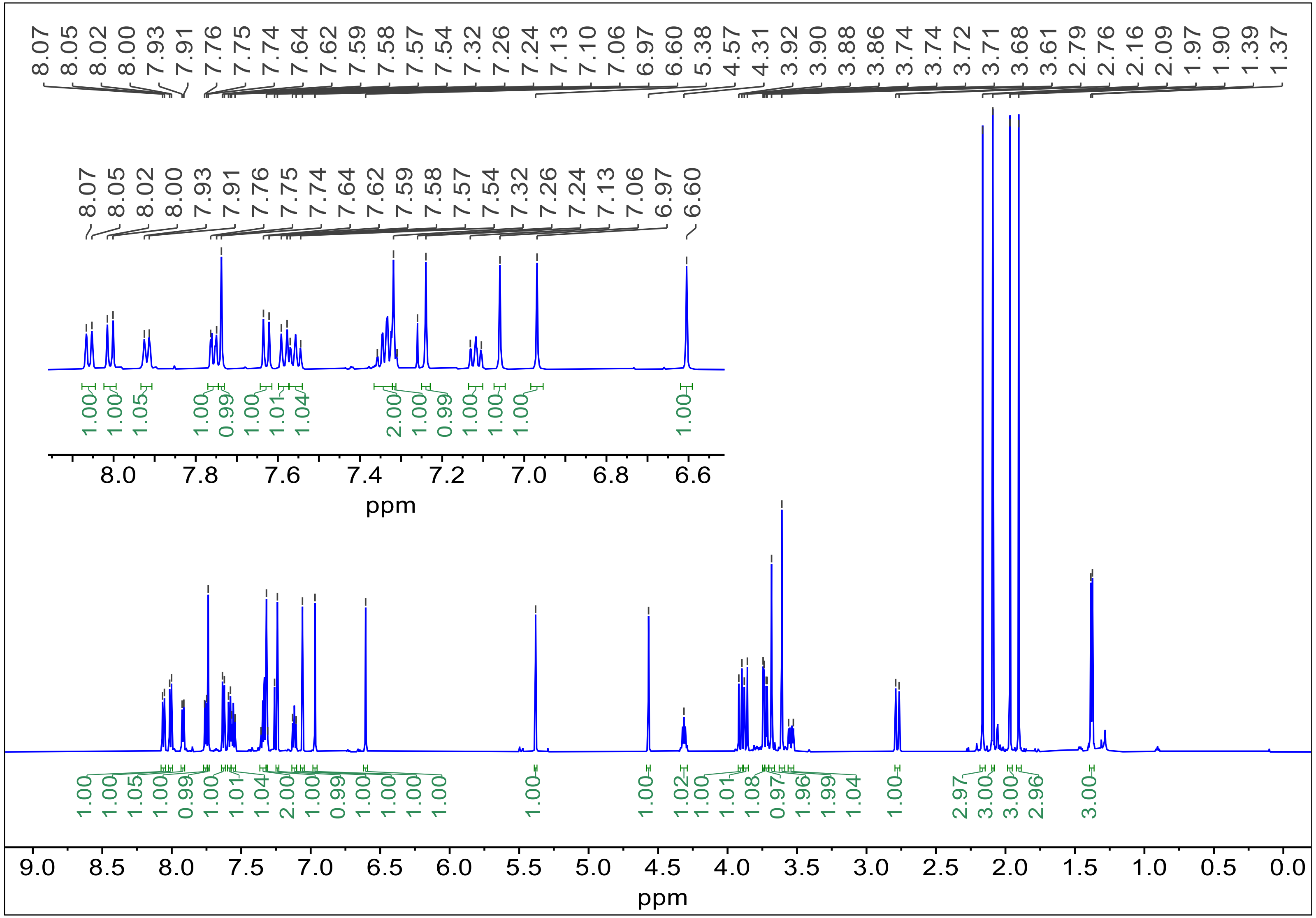

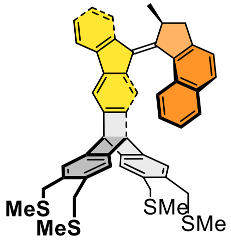

^{13}C {^{1}H} APT NMR (150 MHz, $CDCl_3$): Compound **1C**

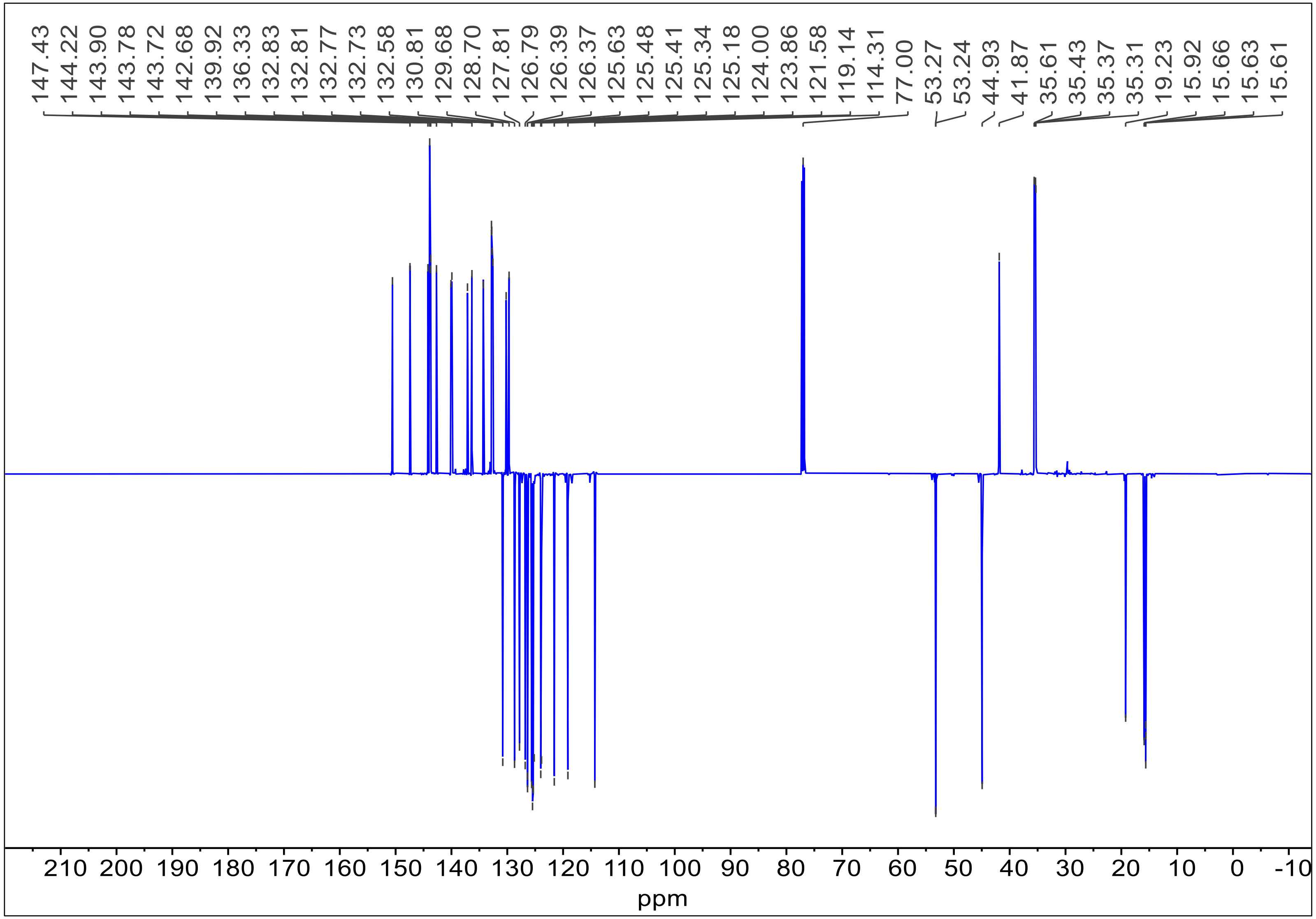

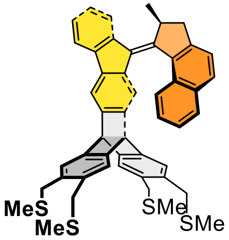

^{1}H – ^{1}H COSY ($CDCl_3$): Compound **1C**

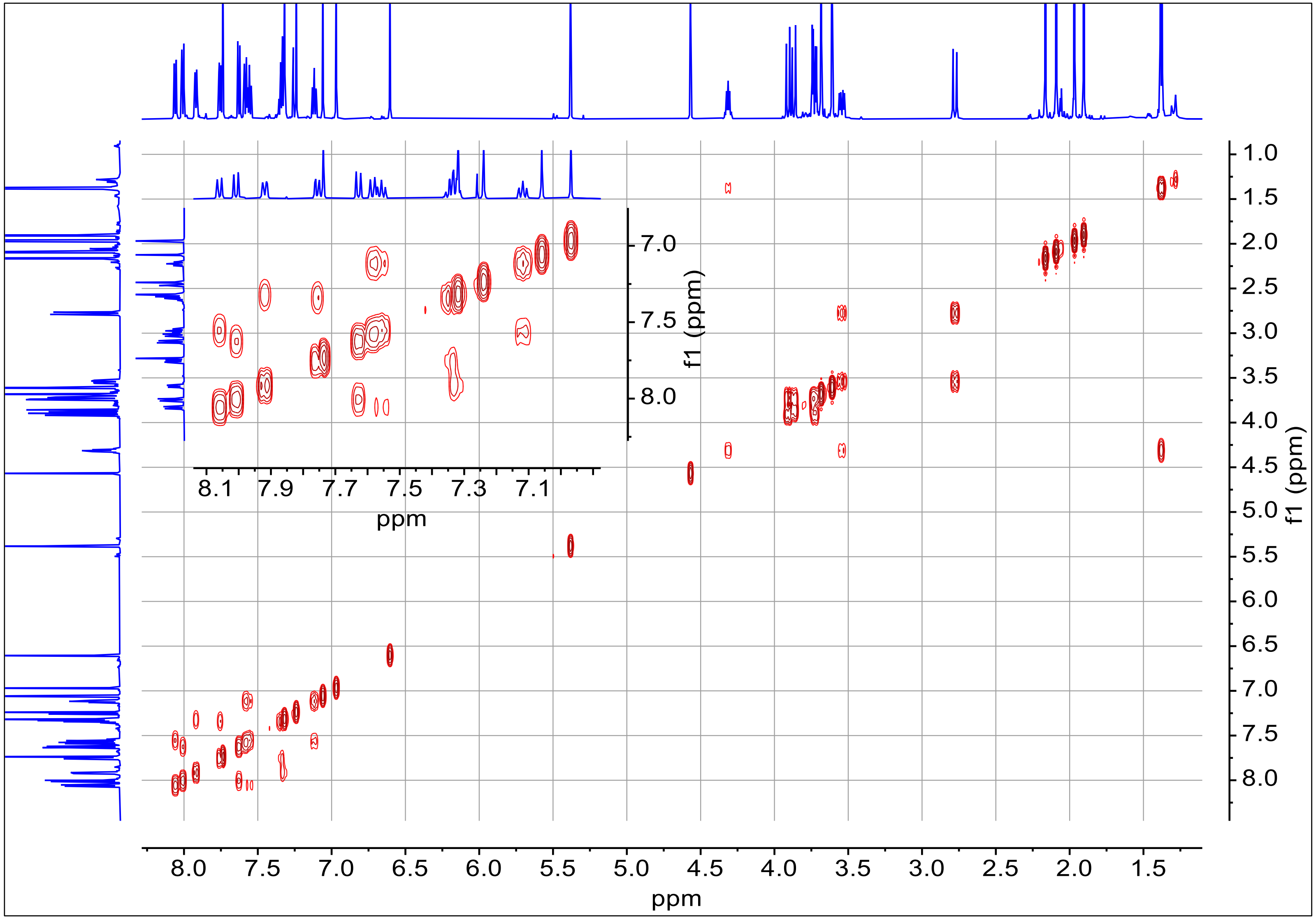

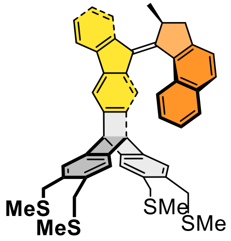

HSQC (CDCl₃): Compound **1C**

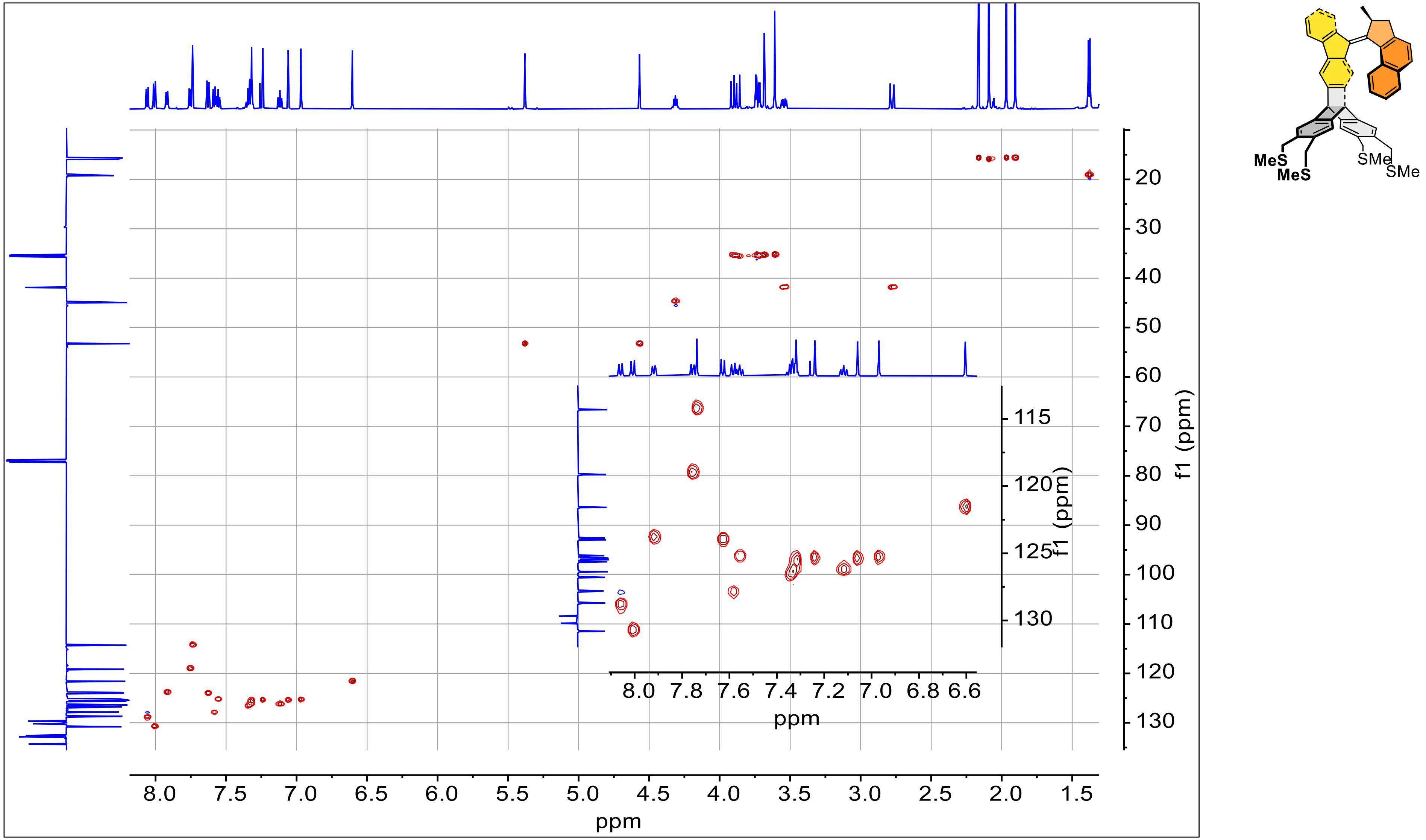

HMBC ($CDCl_3$): Compound **1C**

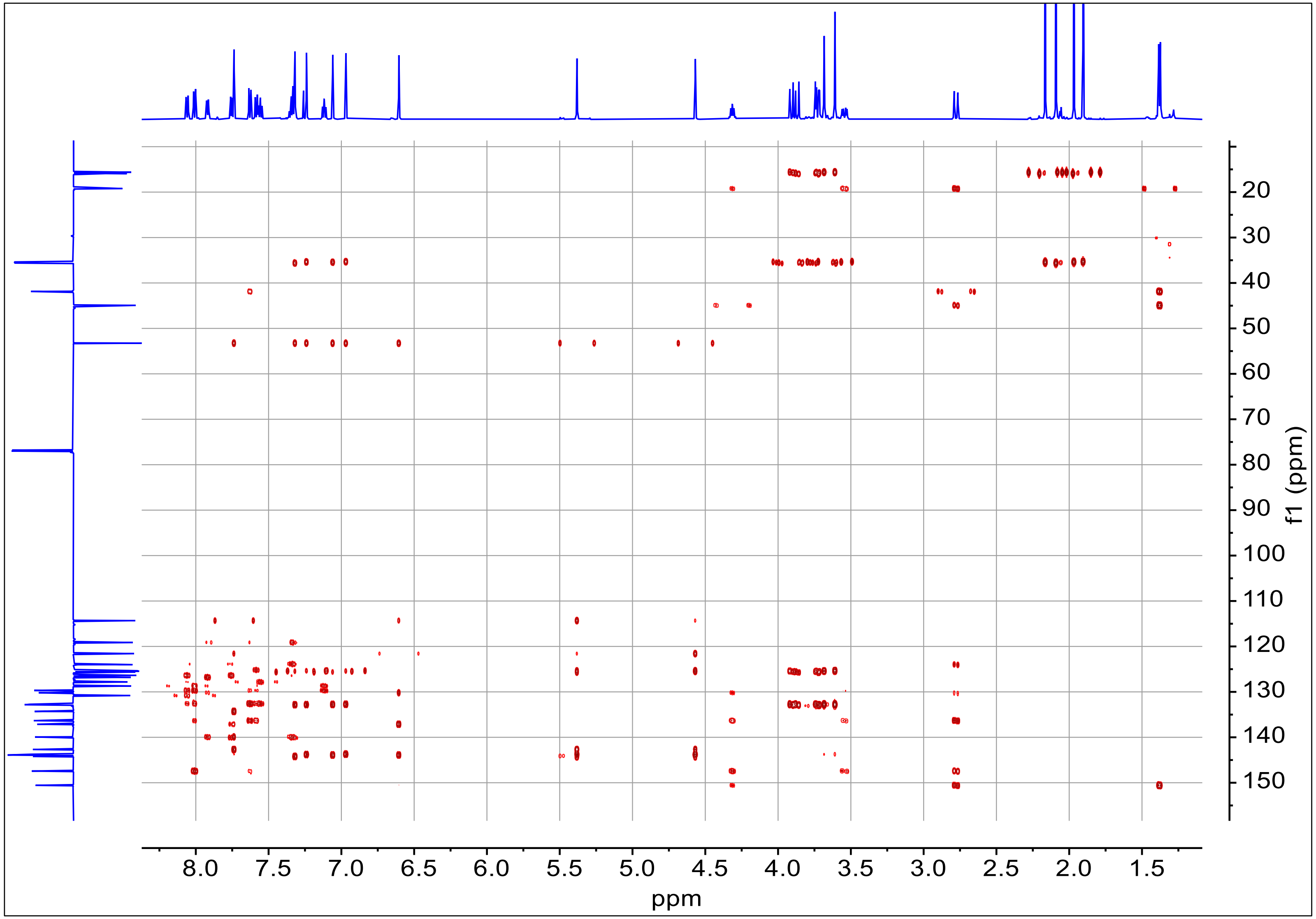

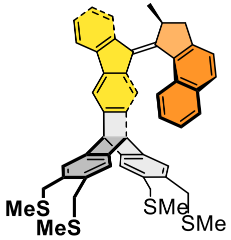

^{1}H NMR (500 MHz, $CDCl_3$): Compound **1D** (obtained after irradiation of **1C** with 385 ± 5 nm at -20 °C)

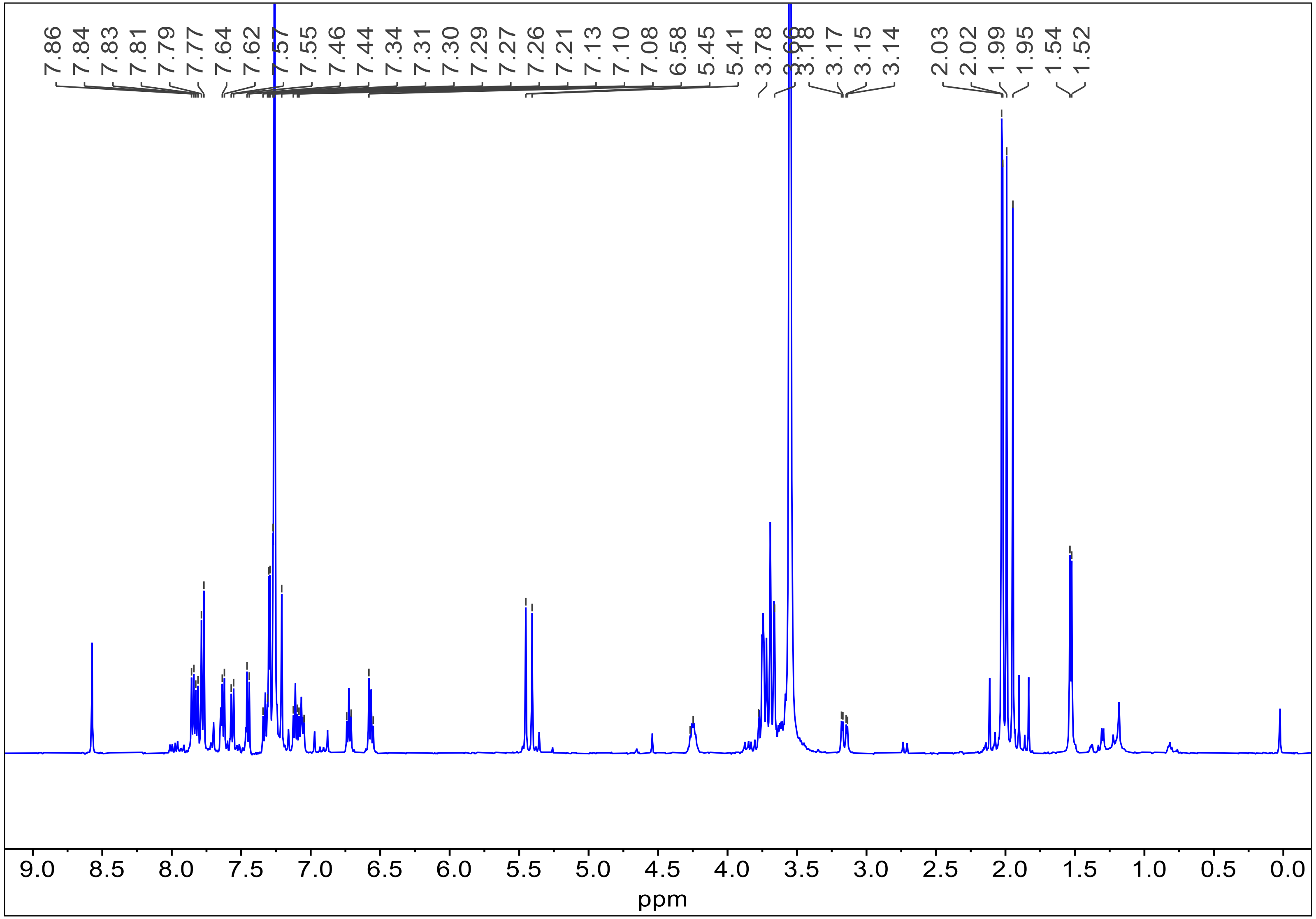

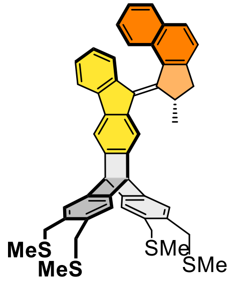

^{1}H NMR (400 MHz, $CDCl_3$): Compounds **2A** and **2C** (1:1 ratio)

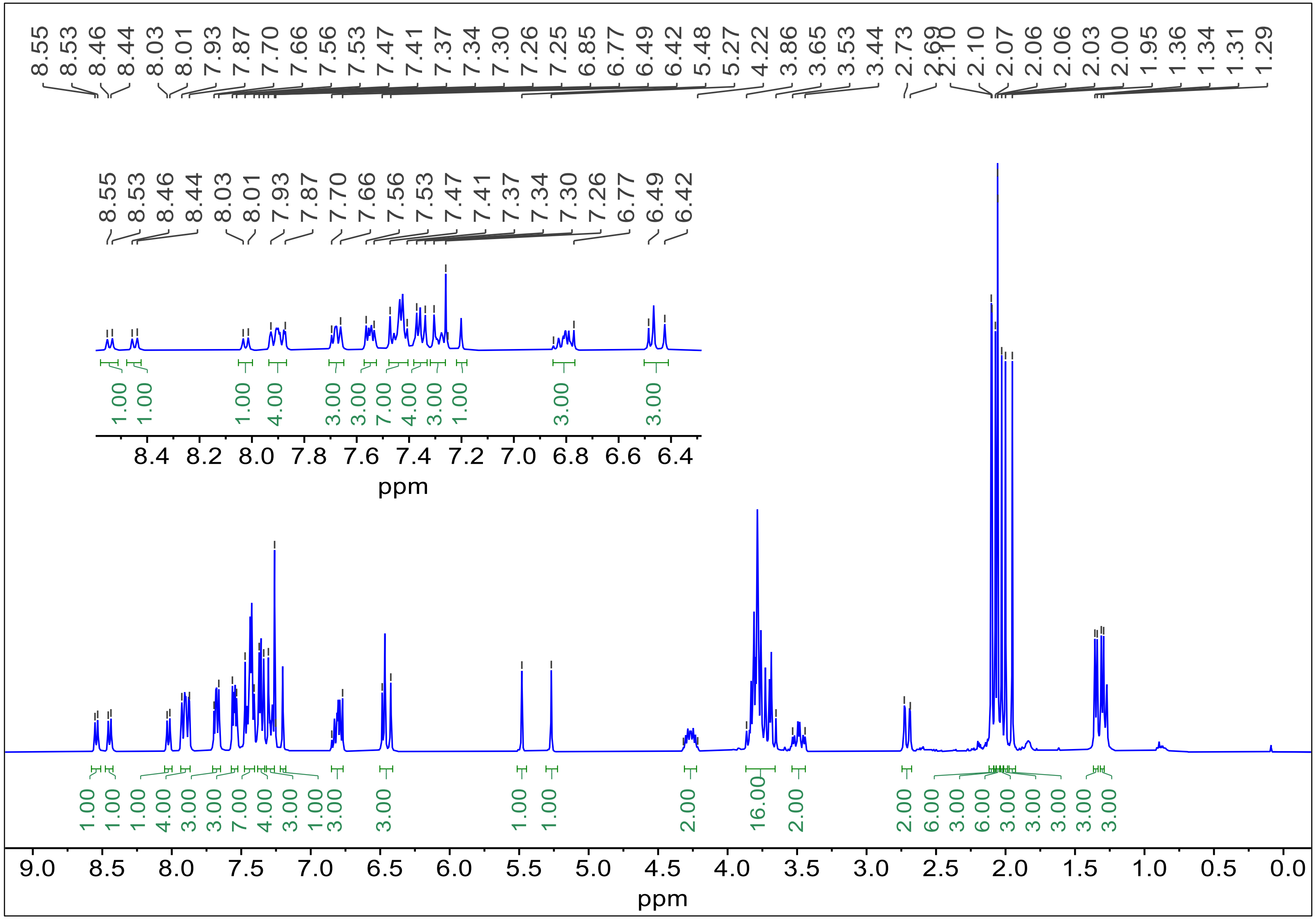

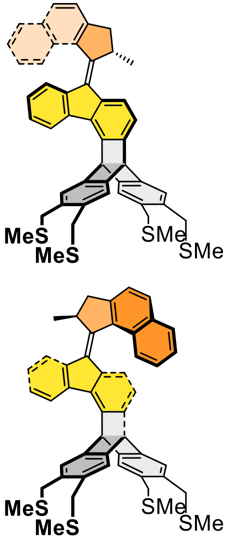

^{1}H NMR (400 MHz, $CDCl_3$): Compounds **2A** and **2C** (4:1 ratio)

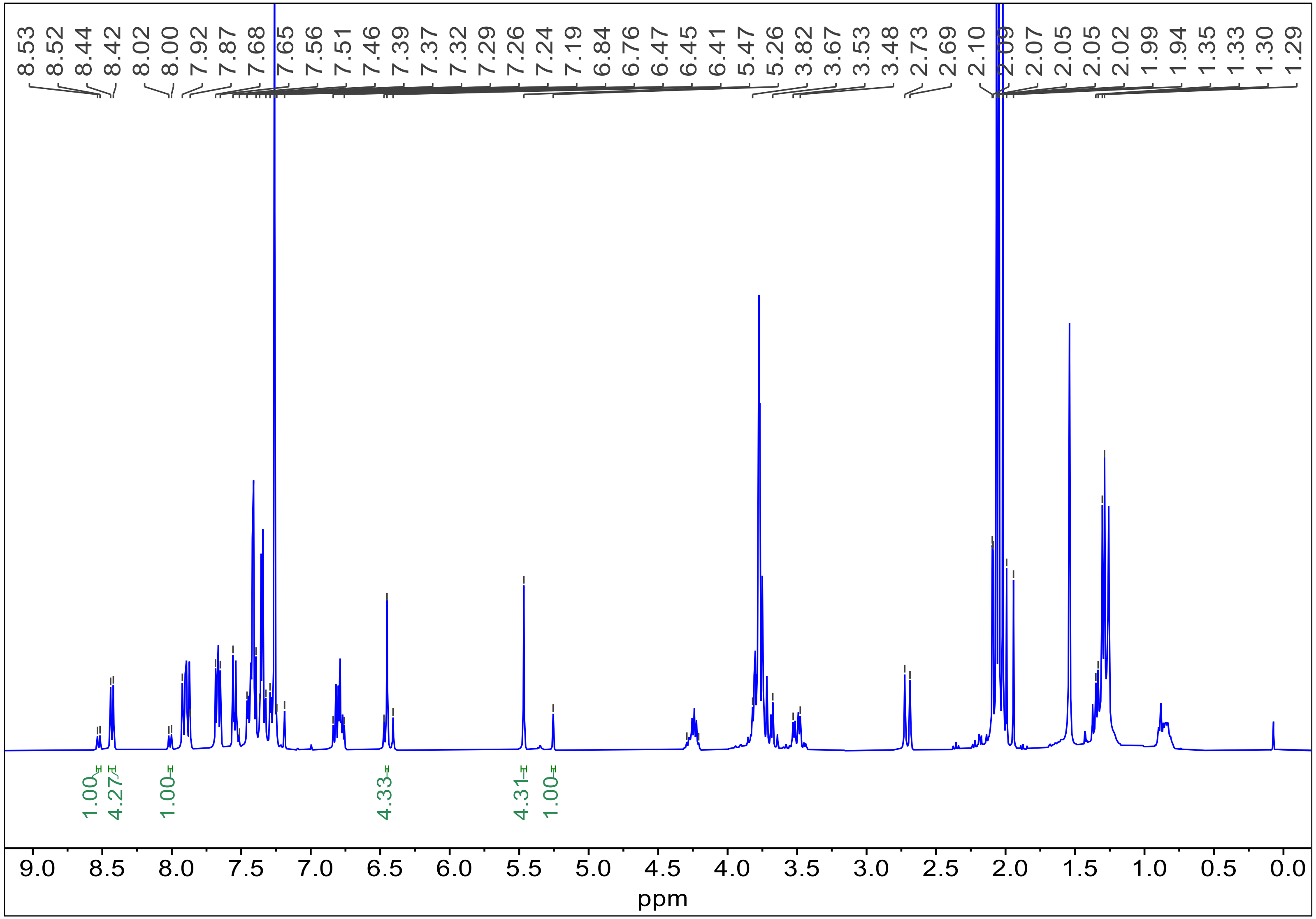

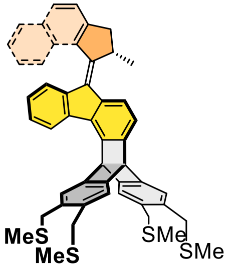

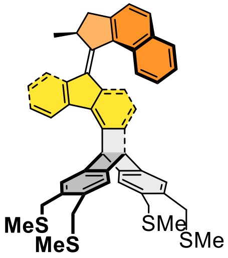

^{1}H NMR (400 MHz, $CDCl_3$): Compounds **2A** and **2C** (1:3 ratio)

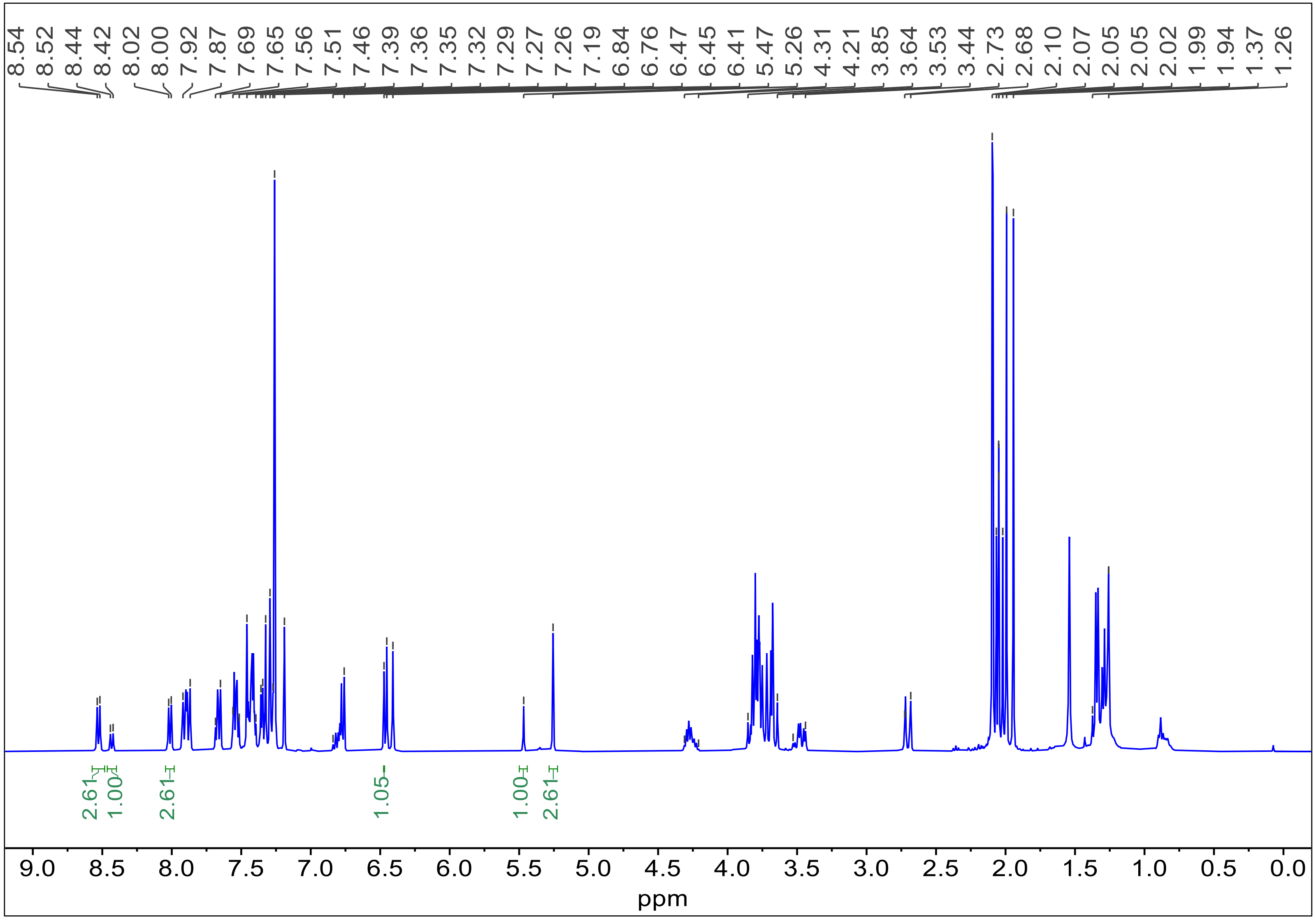

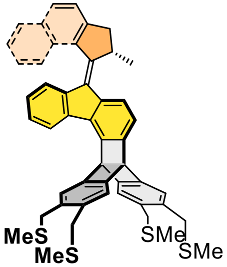

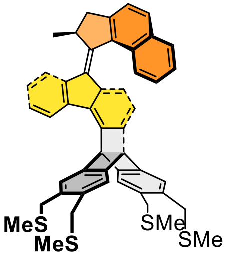

$^{13}C\ \{^{1}H\}$ NMR (100 MHz, $CDCl_3$): Compounds **2A** and **2C** (1:1 ratio)

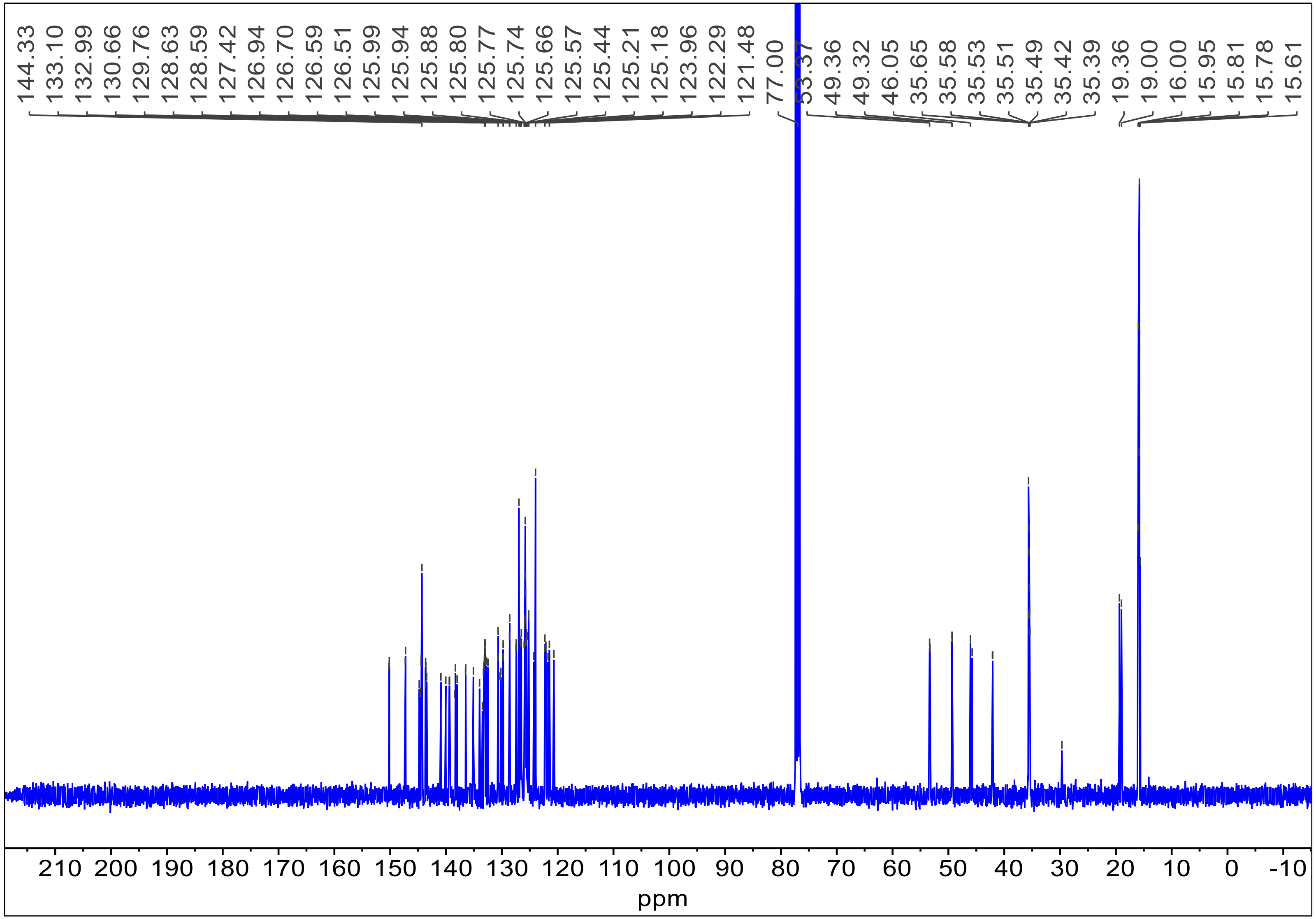

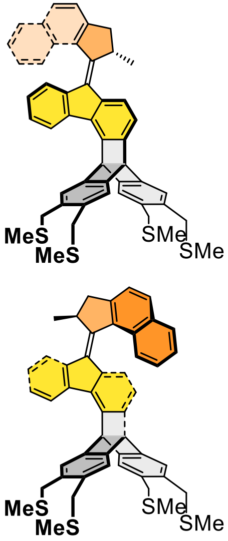

^{13}C {^{1}H} APT NMR (100 MHz, $CDCl_3$): Compounds **2A** and **2C** (1:1 ratio)

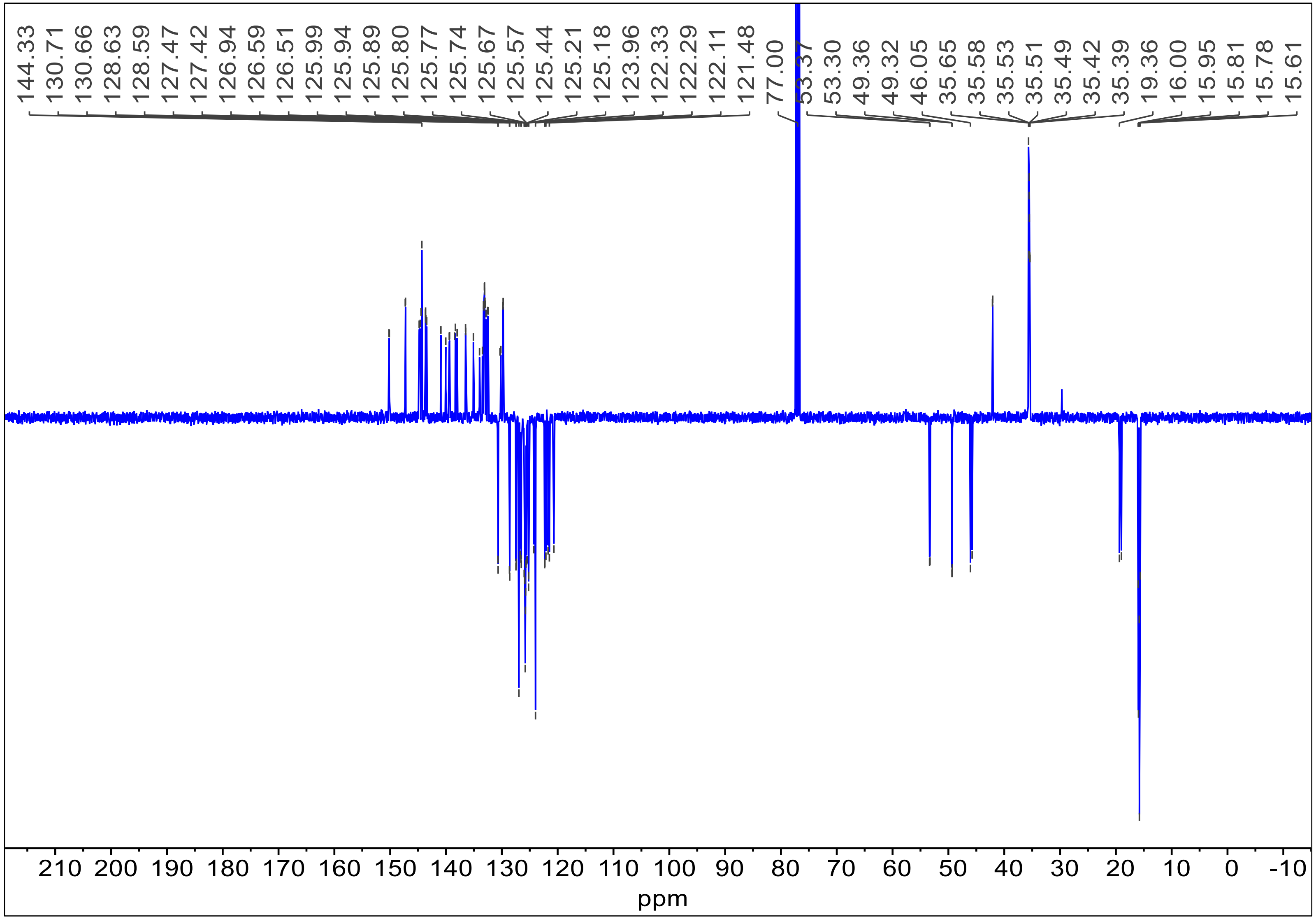

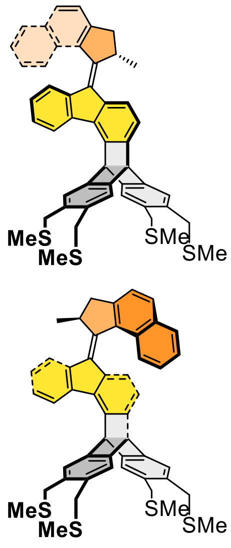

^{1}H – ^{1}H COSY ($CDCl_3$): Compounds **2A** and **2C** (1:1 ratio)

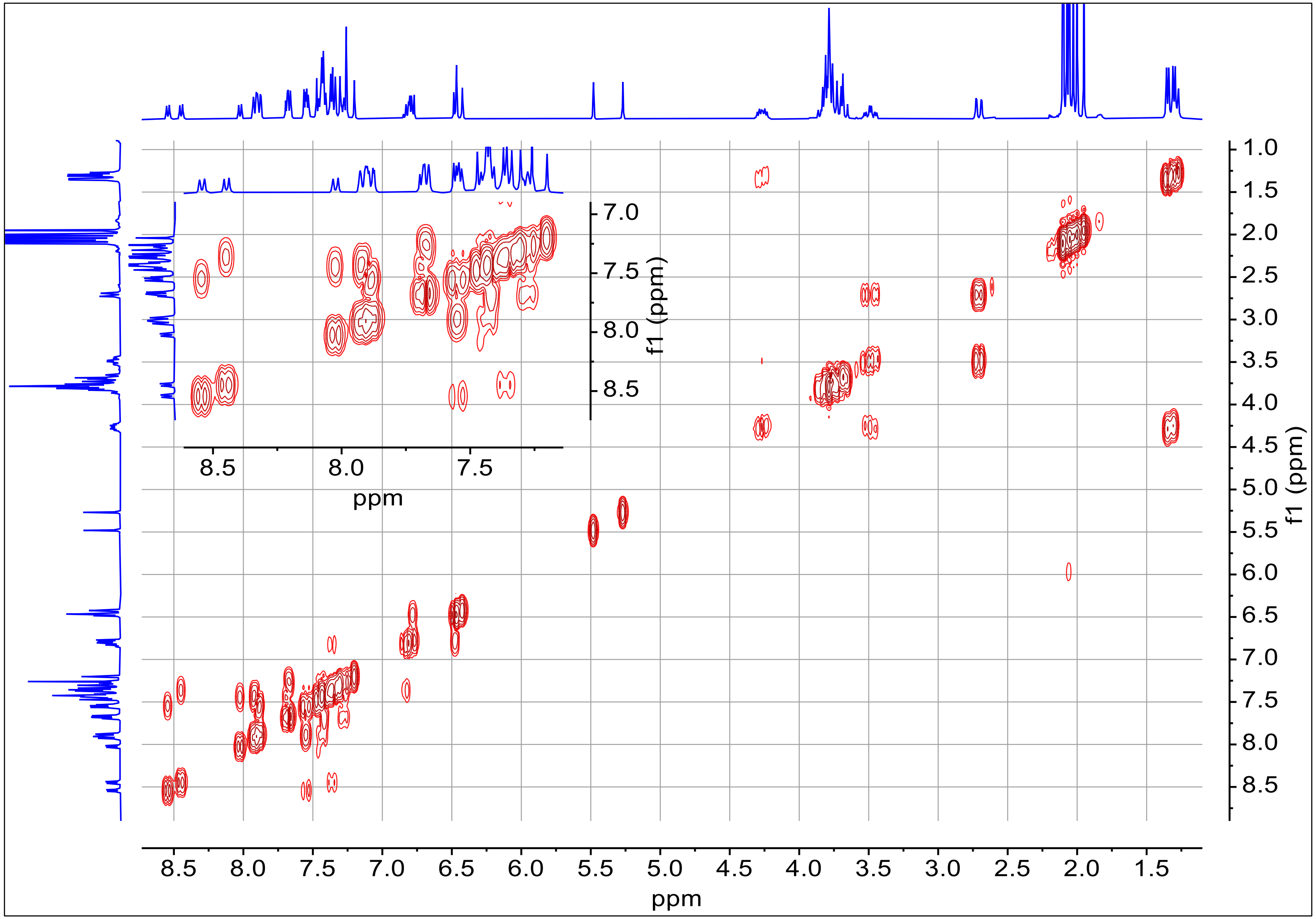

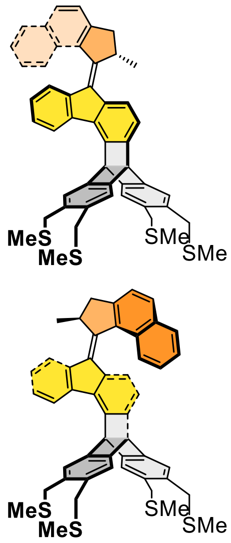

HSQC (CDCl$_3$): Compounds **2A** and **2C** (1:1 ratio)

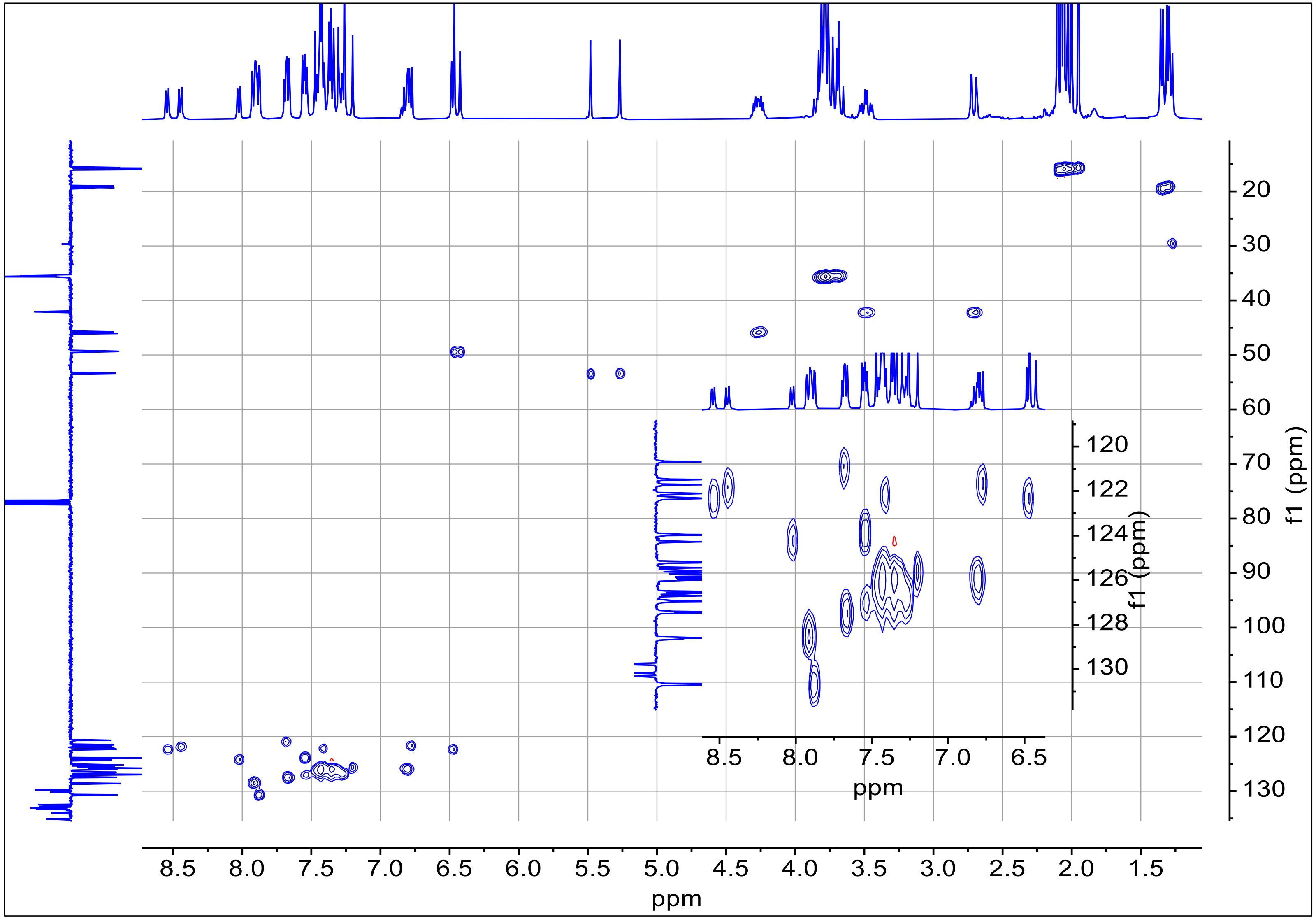

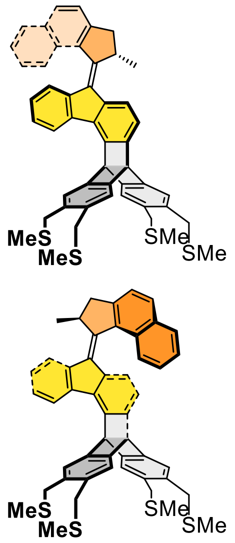

HMBC ($CDCl_3$): Compounds **2A** and **2C** (1:1 ratio)

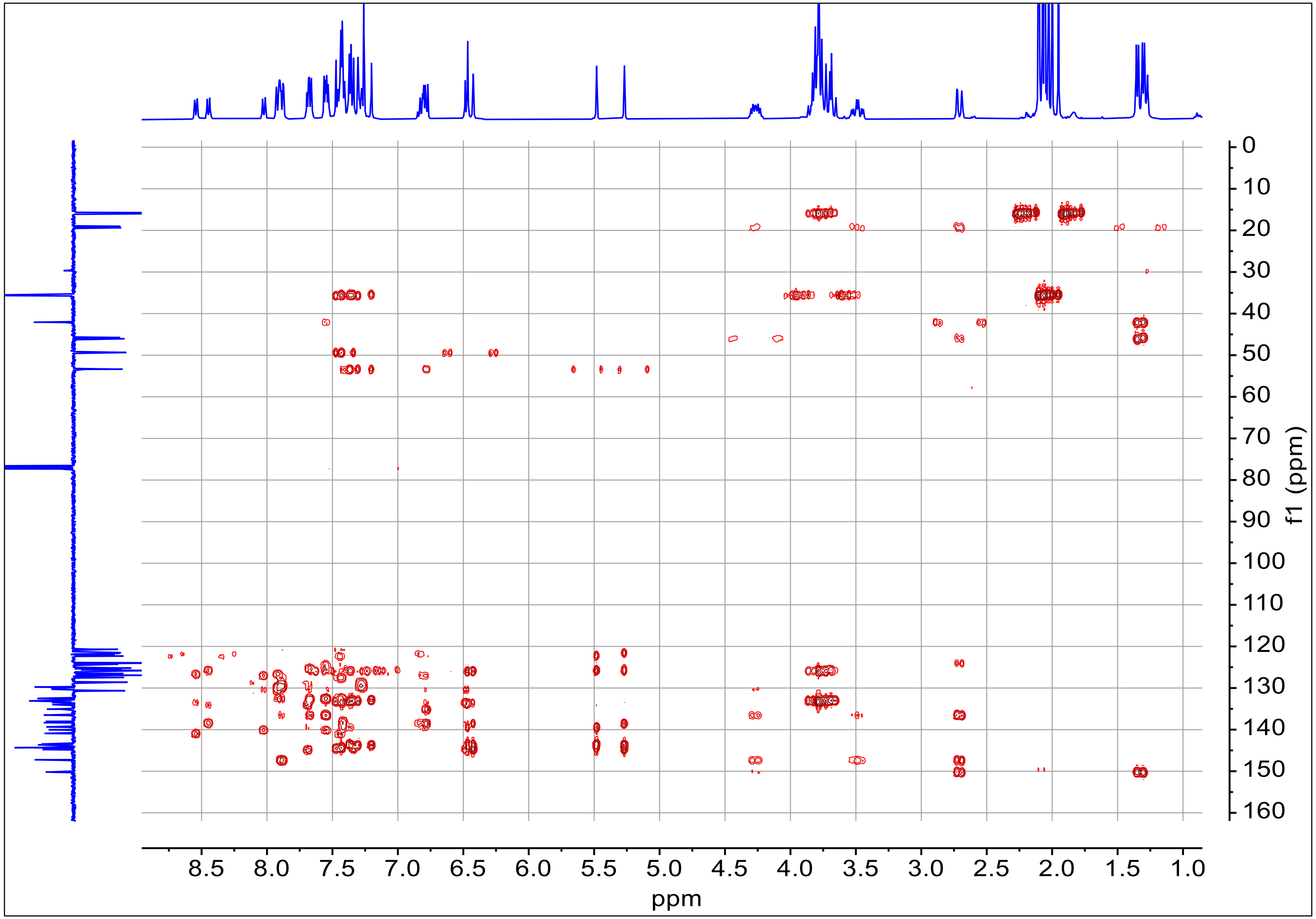

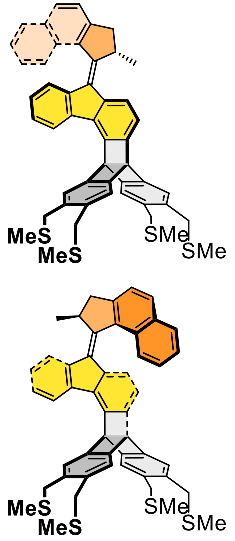

^{1}H NMR (400 MHz, $CDCl_3$): 2,3,6,7-Tetramethylanthracene (**4**)

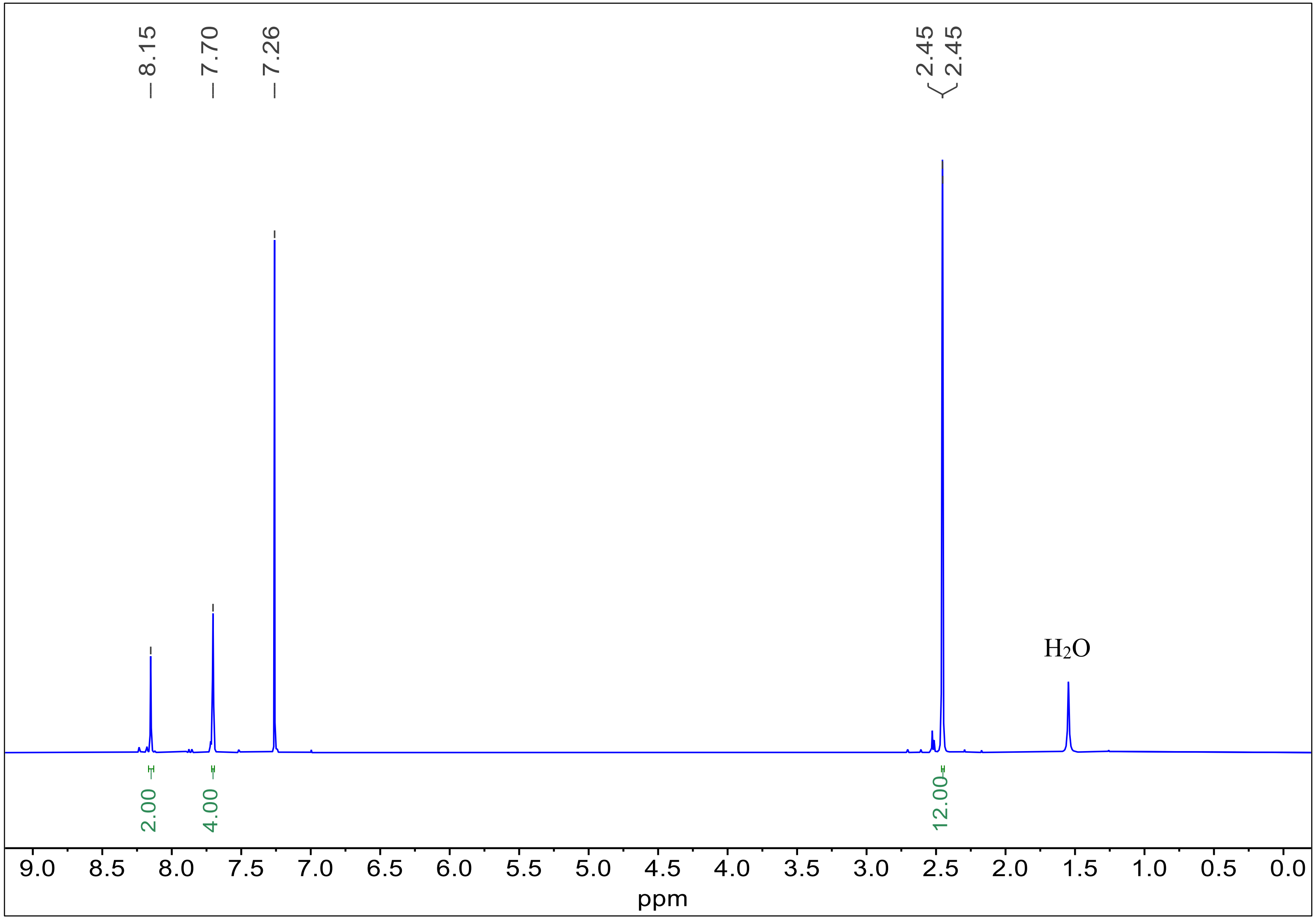

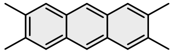

^{13}C {^{1}H} NMR (100 MHz, $CDCl_3$): 2,3,6,7-Tetramethylanthracene (**4**)

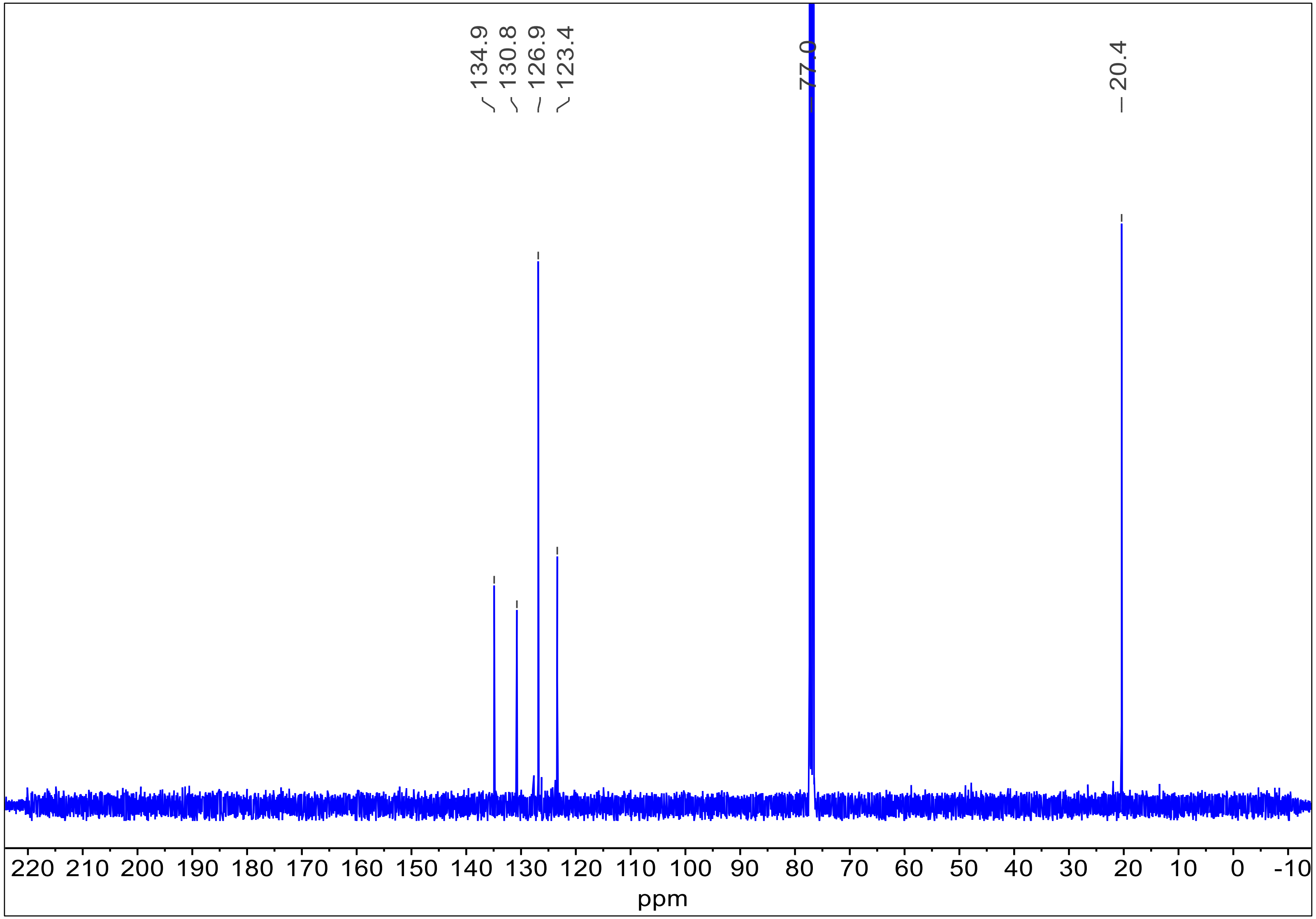

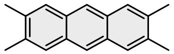

$^{13}C\ \{^{1}H\}$ APT NMR (100 MHz, $CDCl_3$): 2,3,6,7-Tetramethylanthracene (**4**)

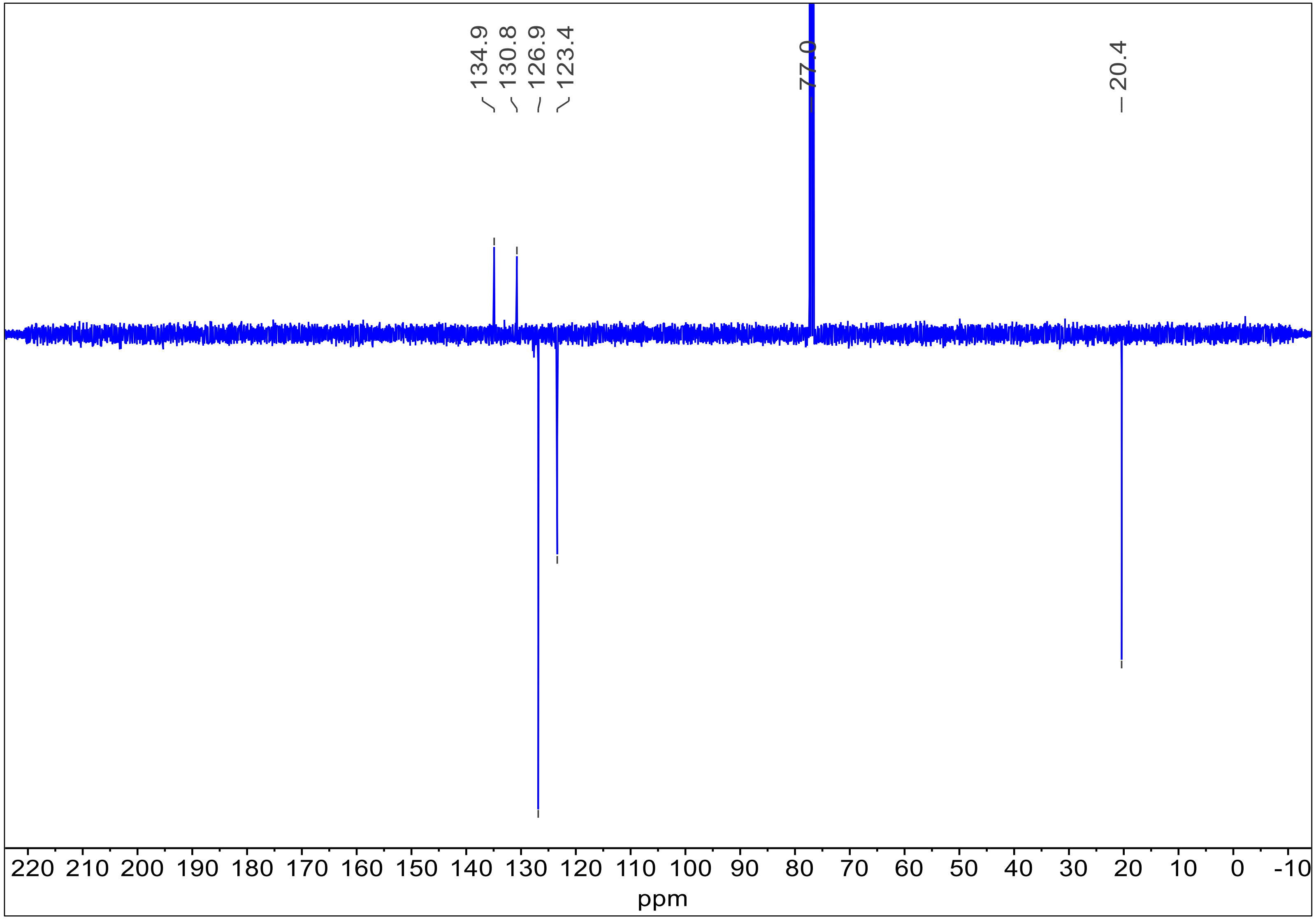

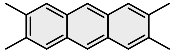

^1H – ^1H COSY ($CDCl_3$): 2,3,6,7-Tetramethylanthracene (**4**)

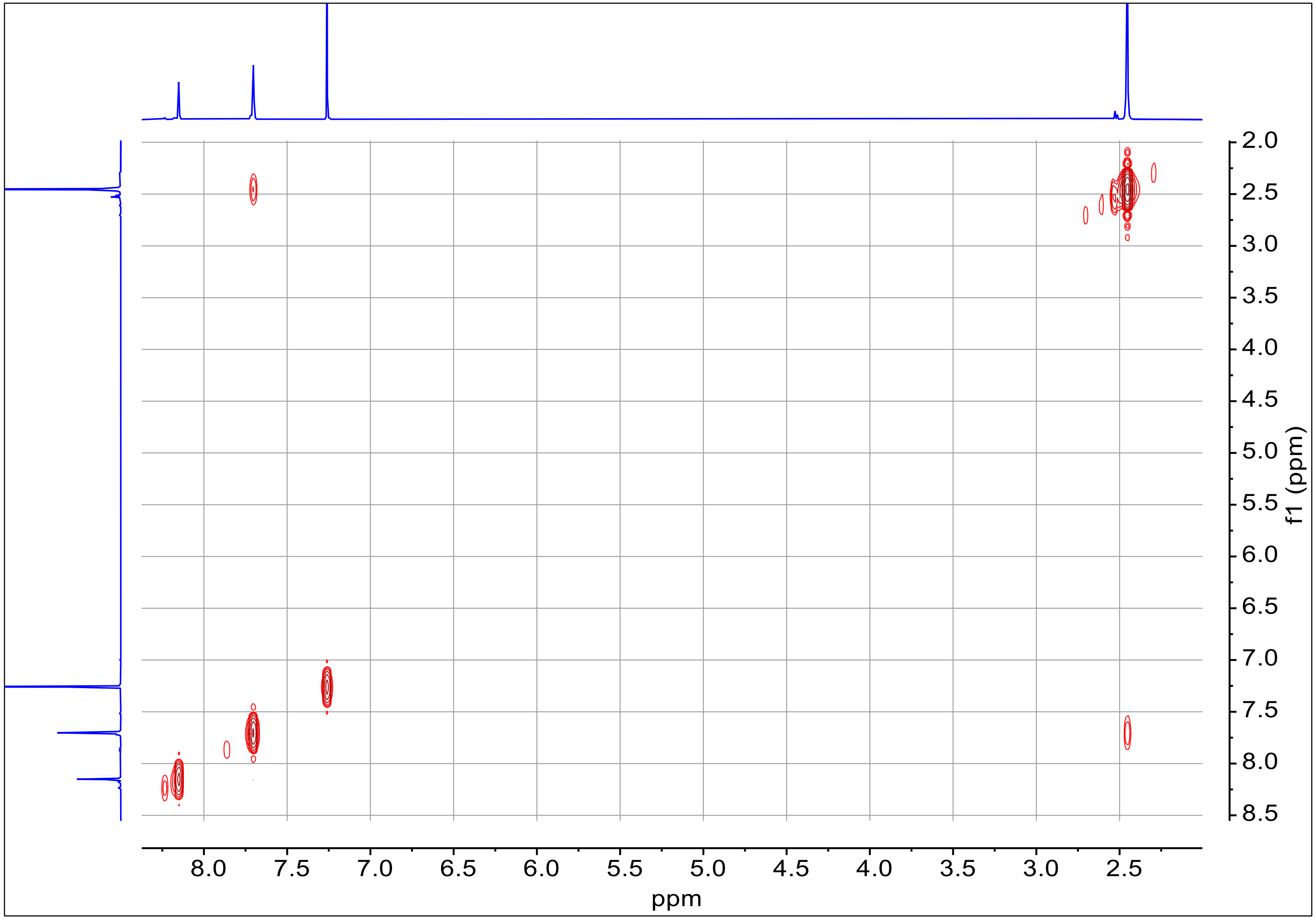

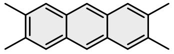

HSQC ($CDCl_3$): 2,3,6,7-Tetramethylanthracene (**4**)

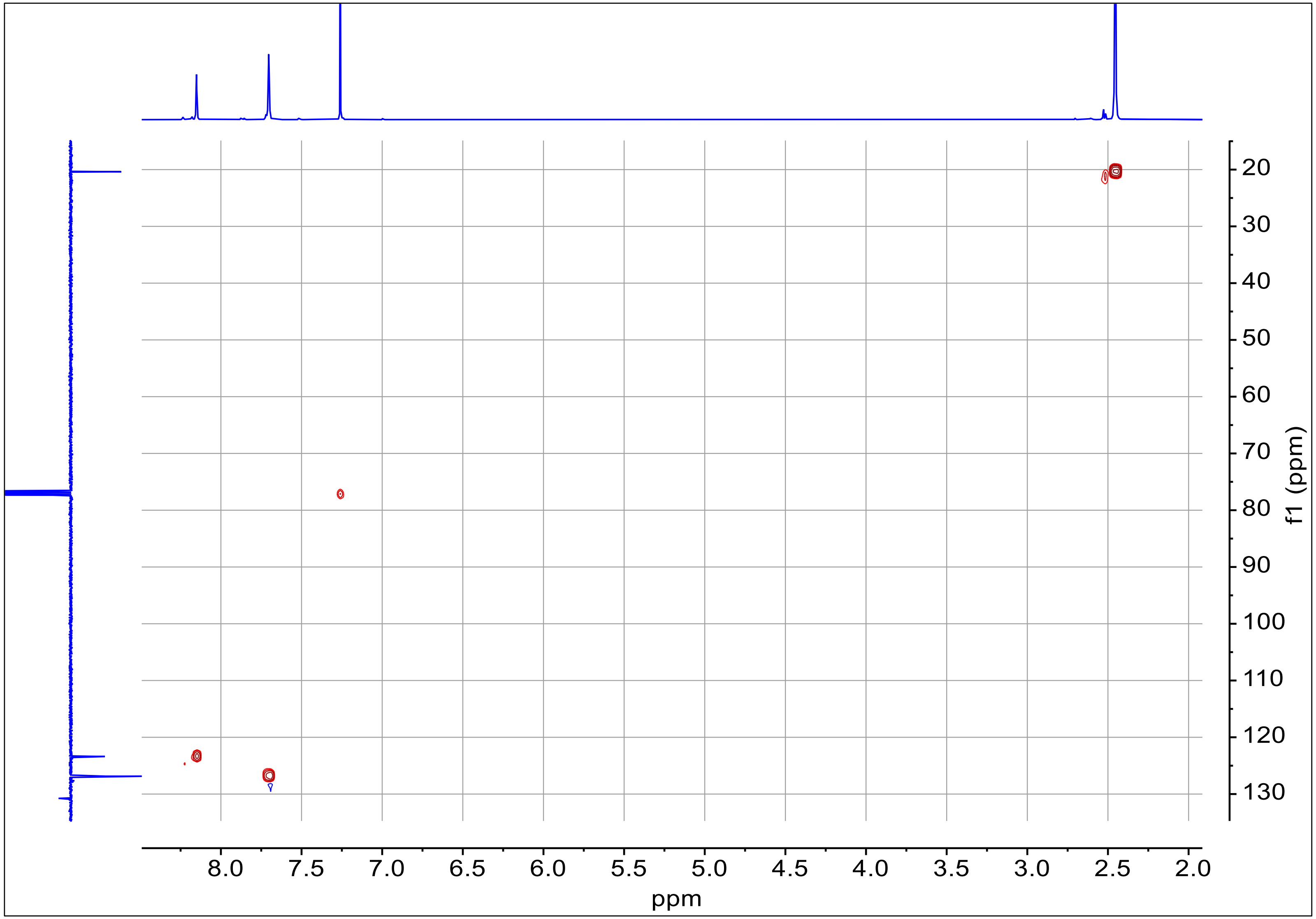

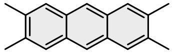

HMBC ($CDCl_3$): 2,3,6,7-Tetramethylanthracene (**4**)

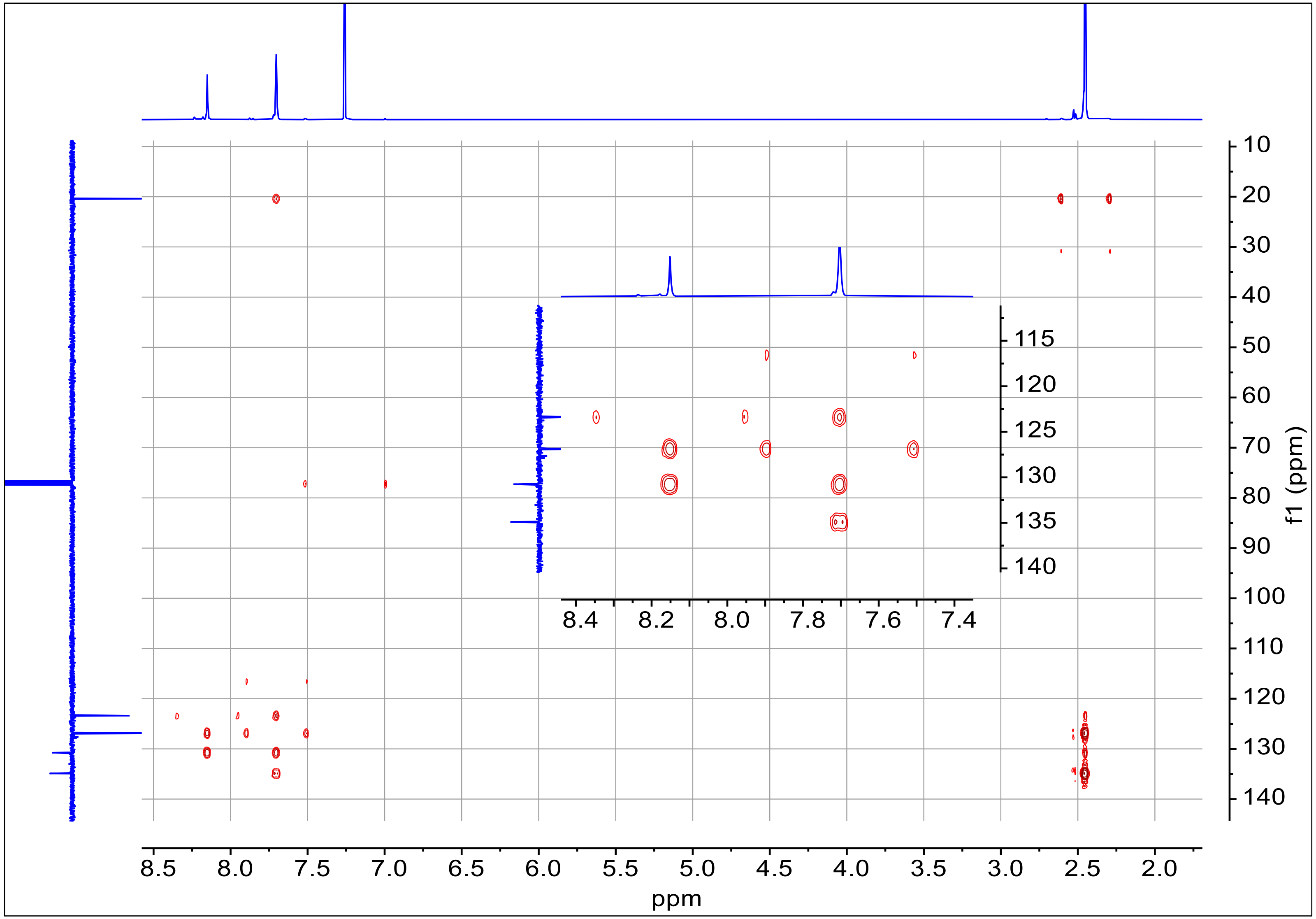

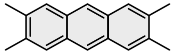

^1H NMR (400 MHz, $CDCl_3$): 1,2,6,7-Tetramethylanthracene (**5**)

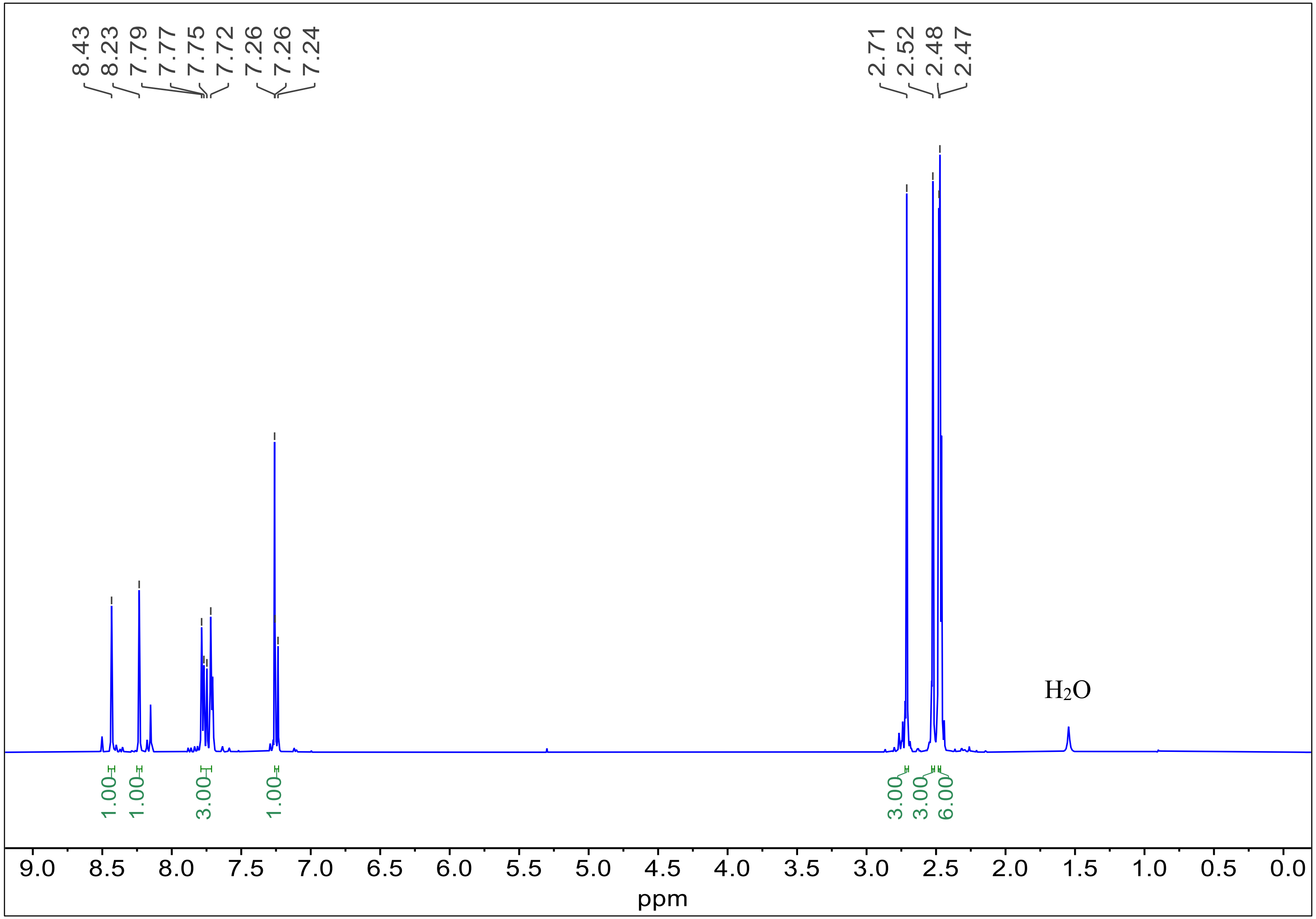

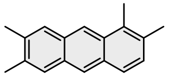

$^{13}C\ \{^1H\}$ NMR (100 MHz, $CDCl_3$): 1,2,6,7-Tetramethylanthracene (**5**)

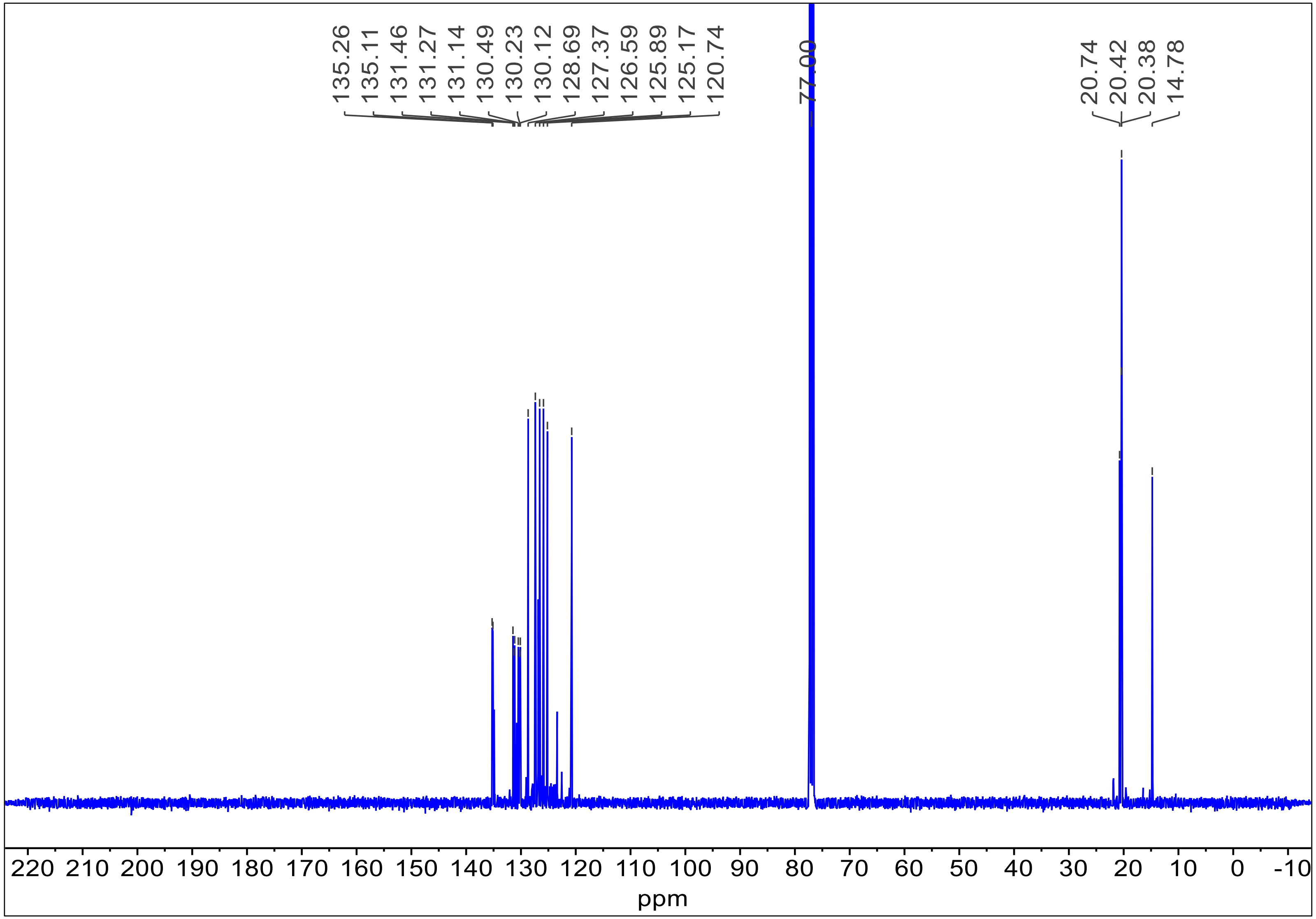

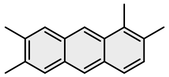

^{13}C {^{1}H} APT NMR (100 MHz, $CDCl_3$): 1,2,6,7-Tetramethylanthracene (**5**)

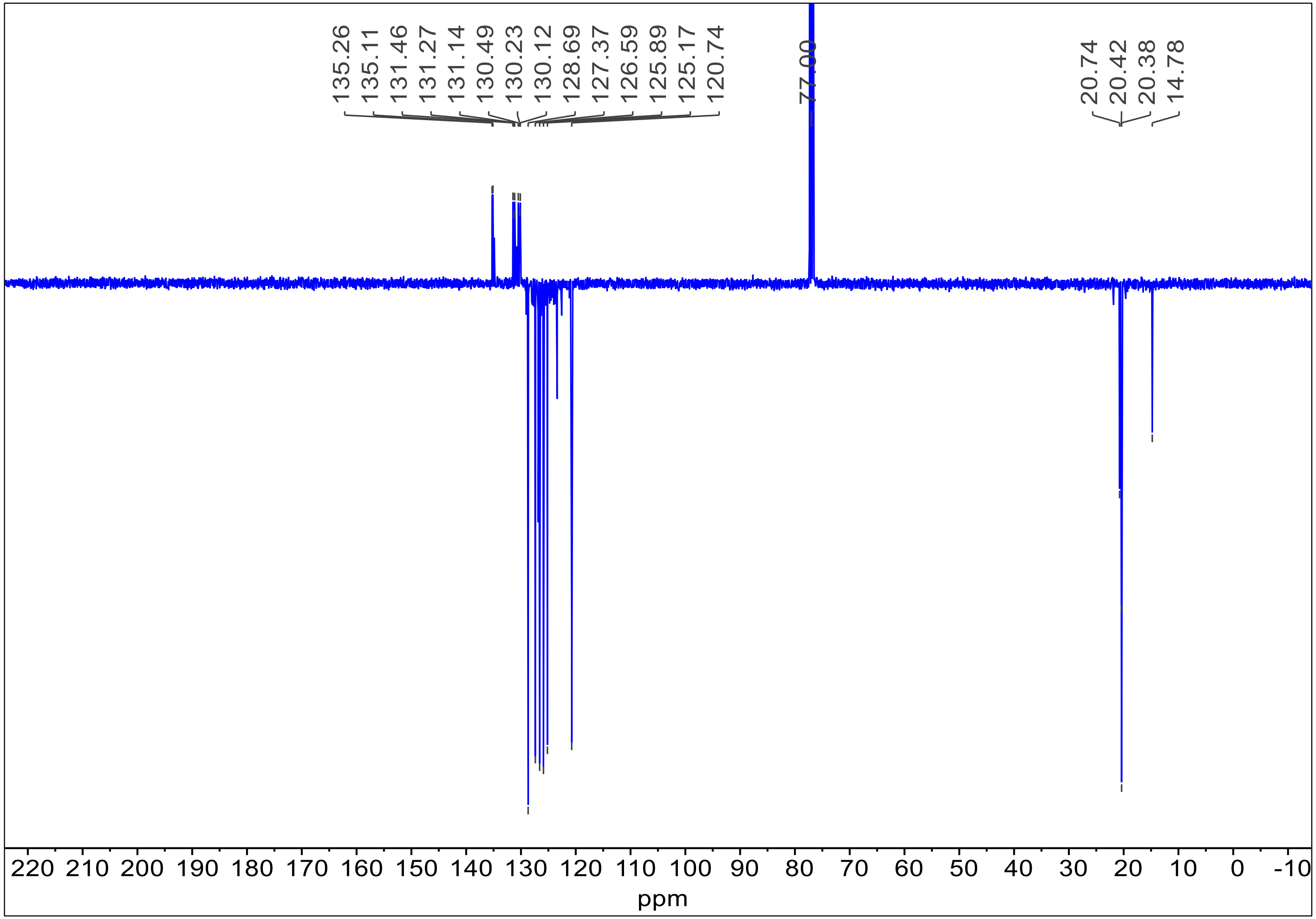

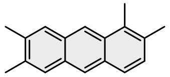

^{1}H – ^{1}H COSY ($CDCl_3$): 1,2,6,7-Tetramethylanthracene (**5**)

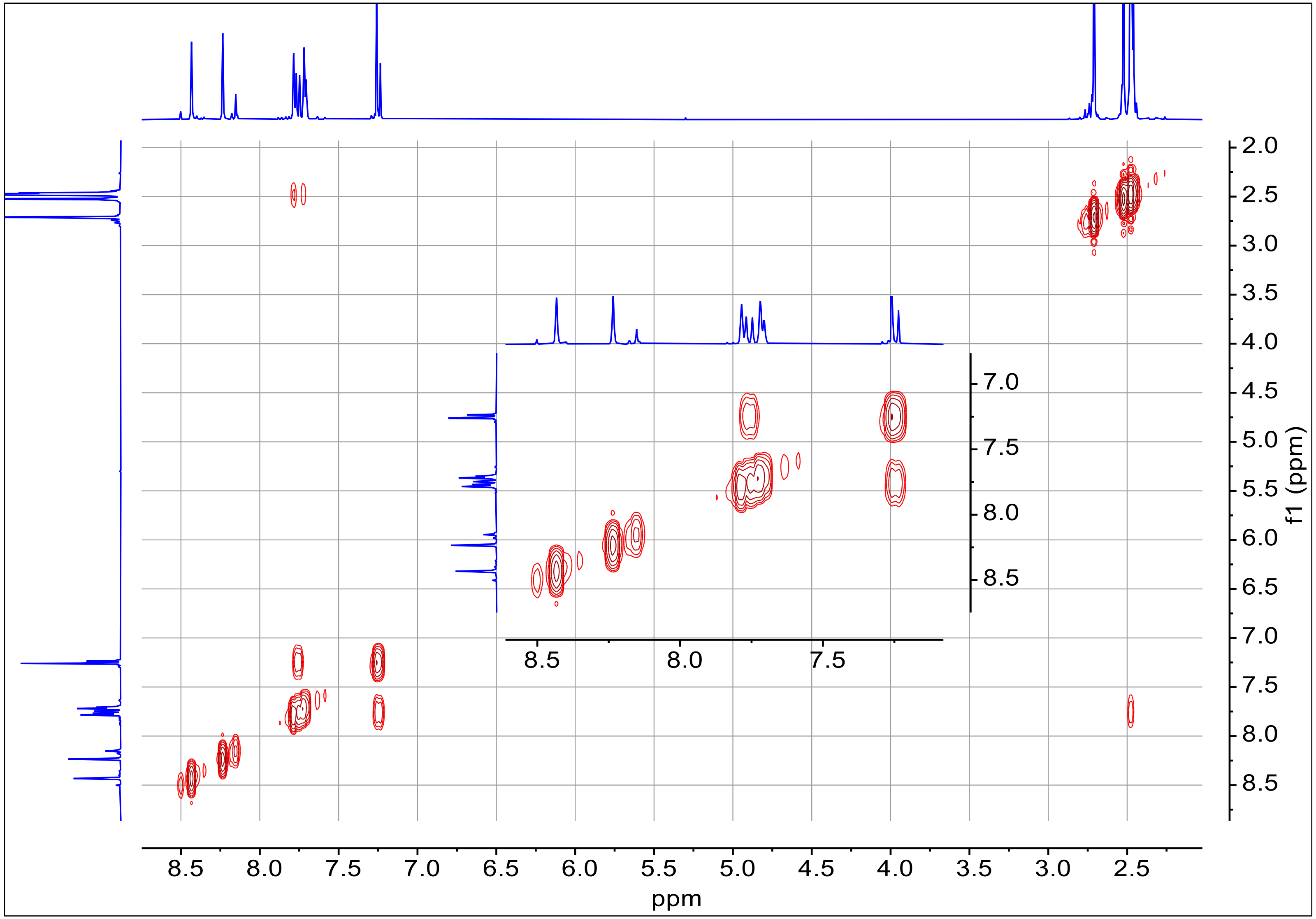

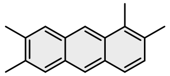

HSQC ($CDCl_3$): 1,2,6,7-Tetramethylanthracene (**5**)

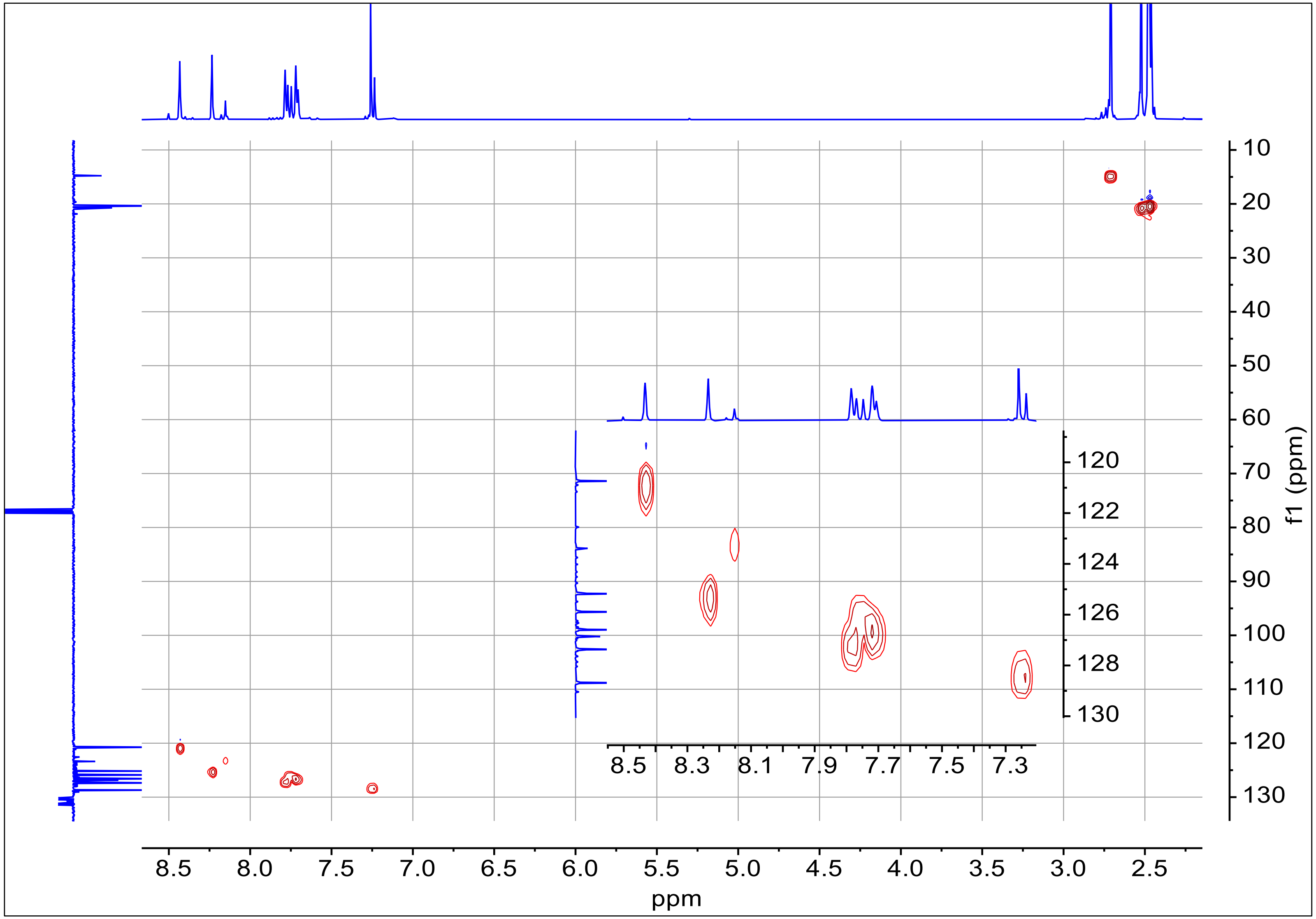

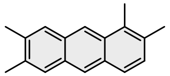

HMBC ($CDCl_3$): 1,2,6,7-Tetramethylanthracene (**5**)

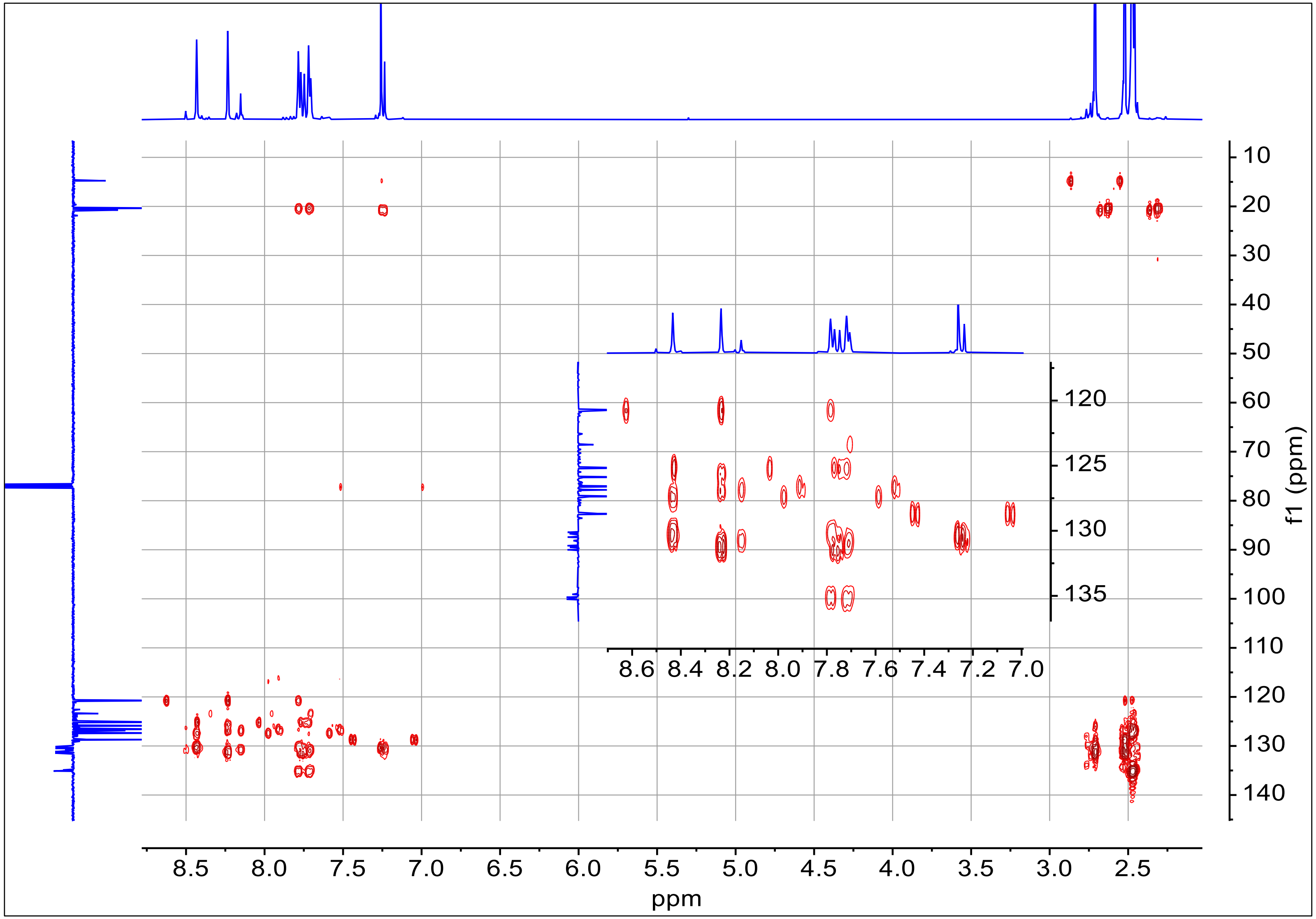

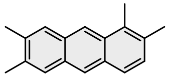

^{1}H NMR (400 MHz, $CDCl_3$): Compound **8**

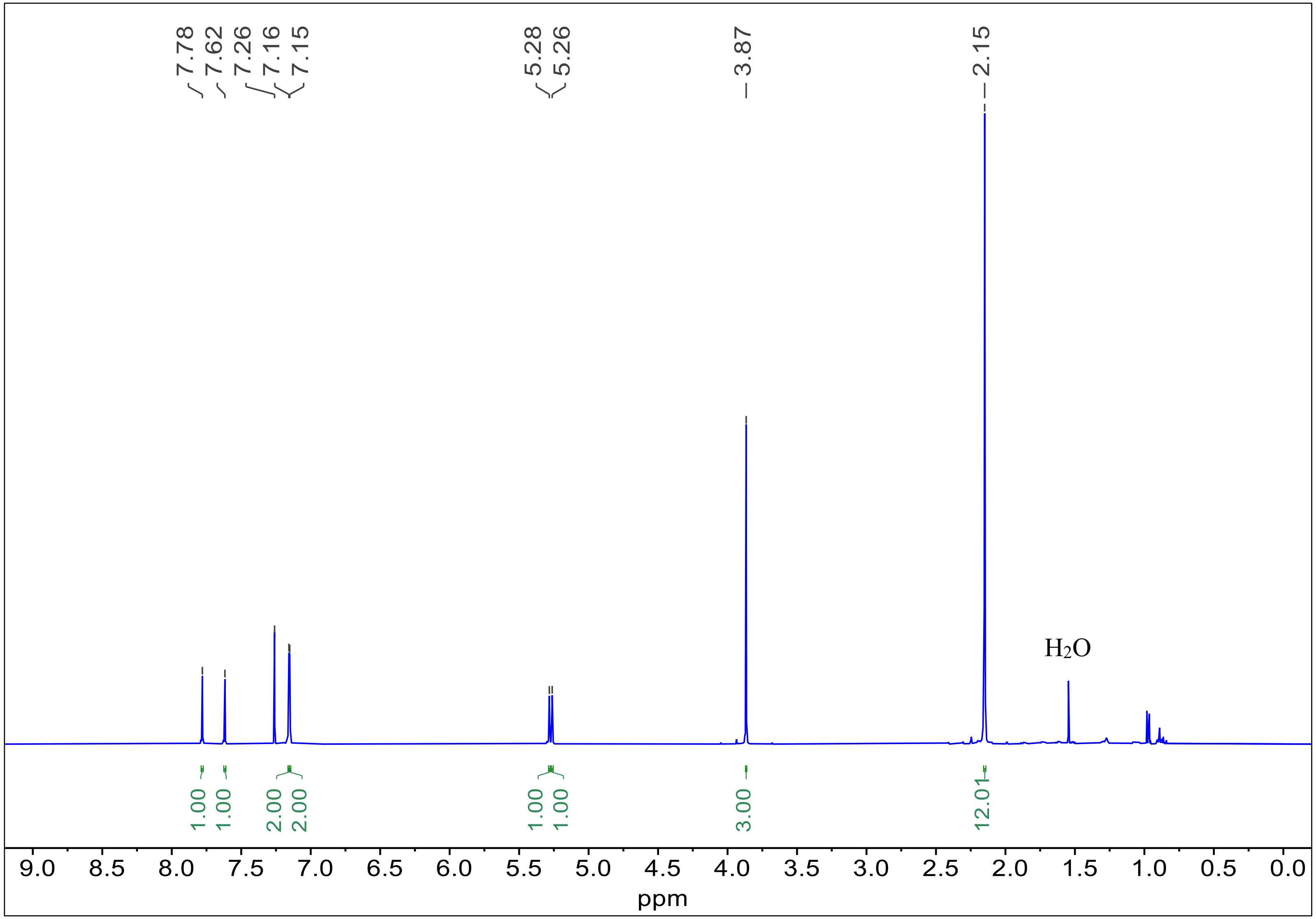

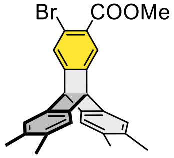

$^{13}C\ \{^{1}H\}$ APT NMR (100 MHz, $CDCl_3$): Compound **8**

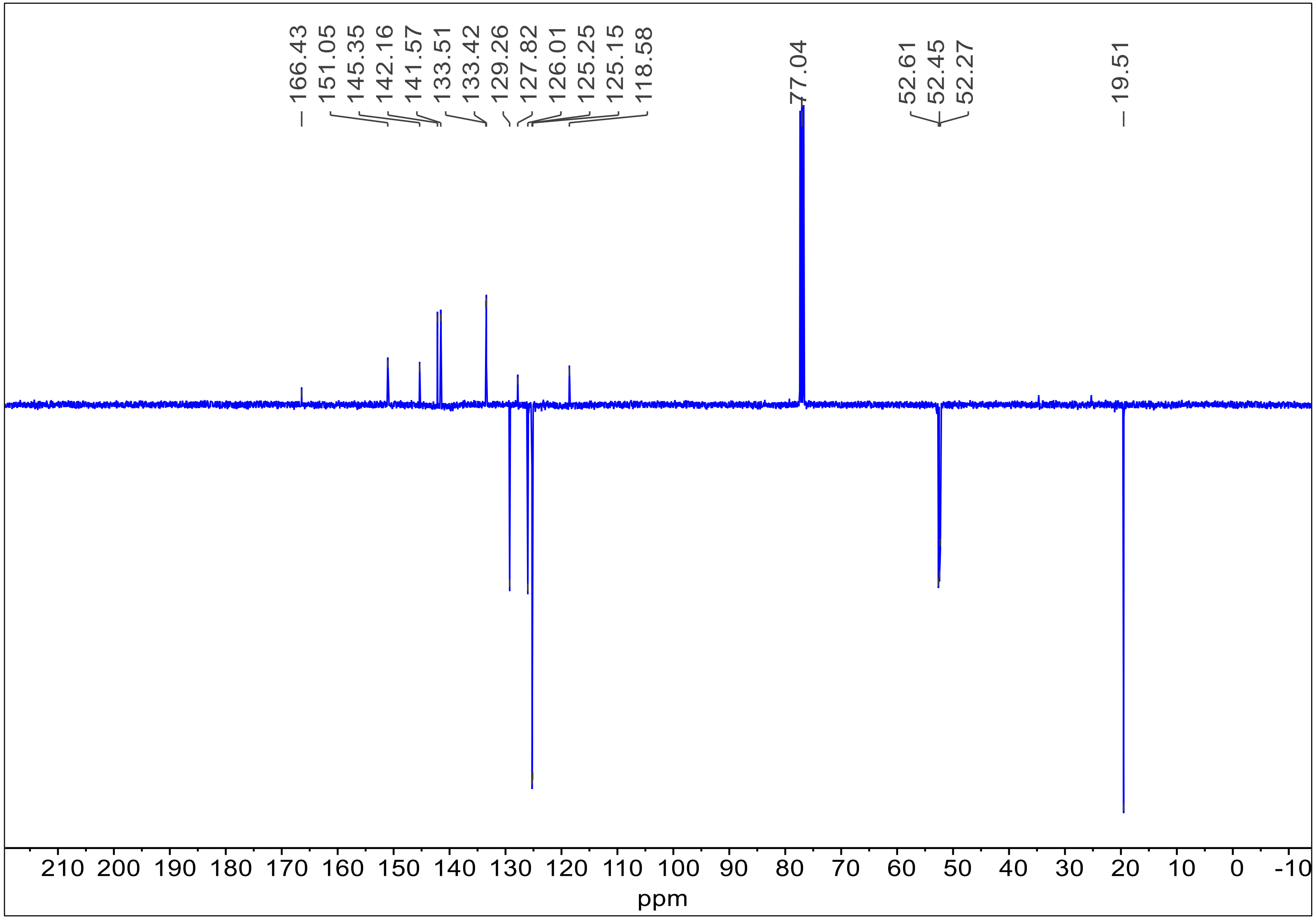

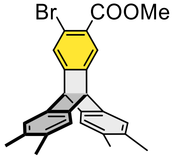

^{1}H – ^{1}H COSY ($CDCl_3$): Compound **8**

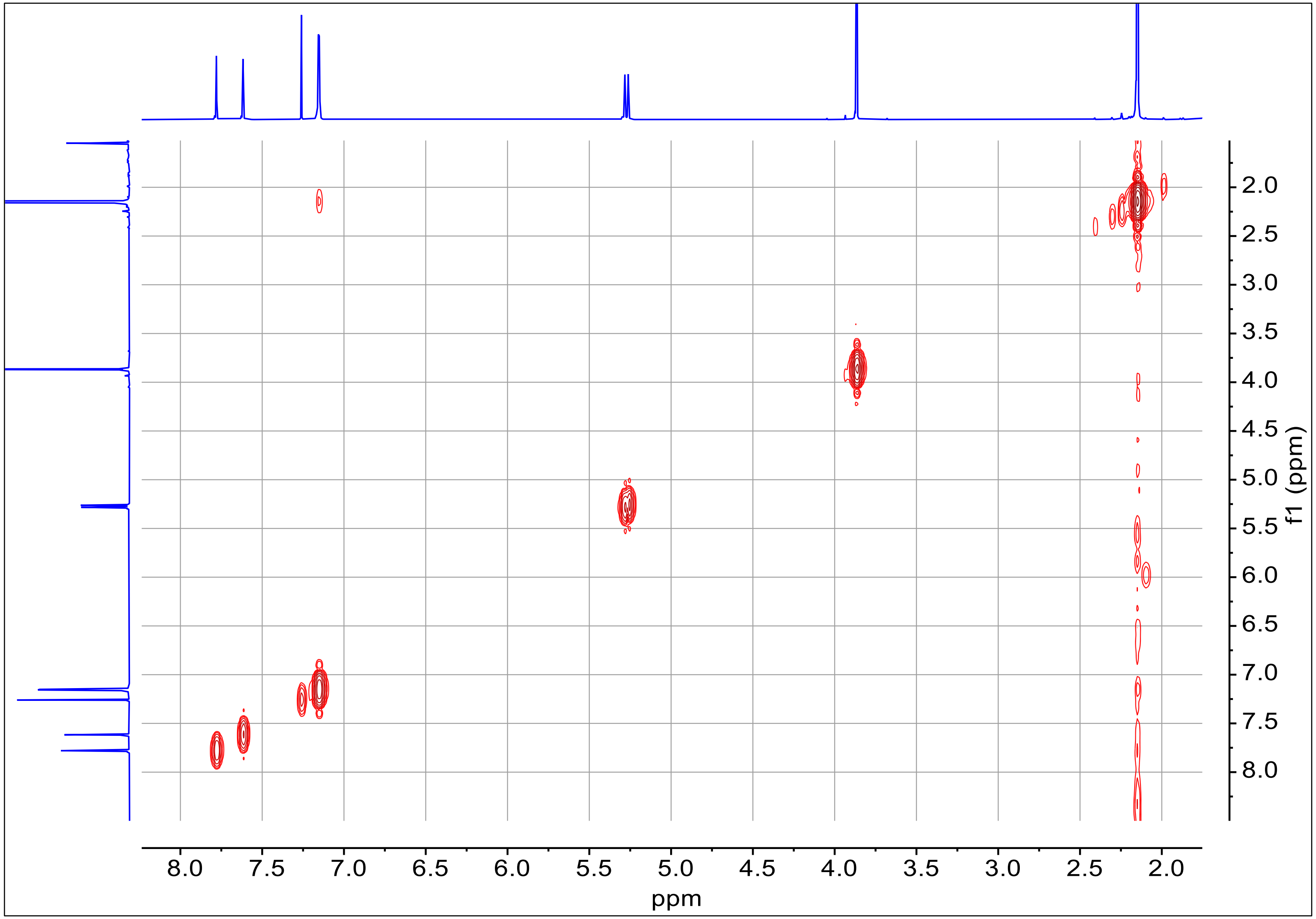

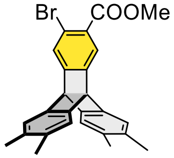

HSQC ($CDCl_3$): Compound **8**

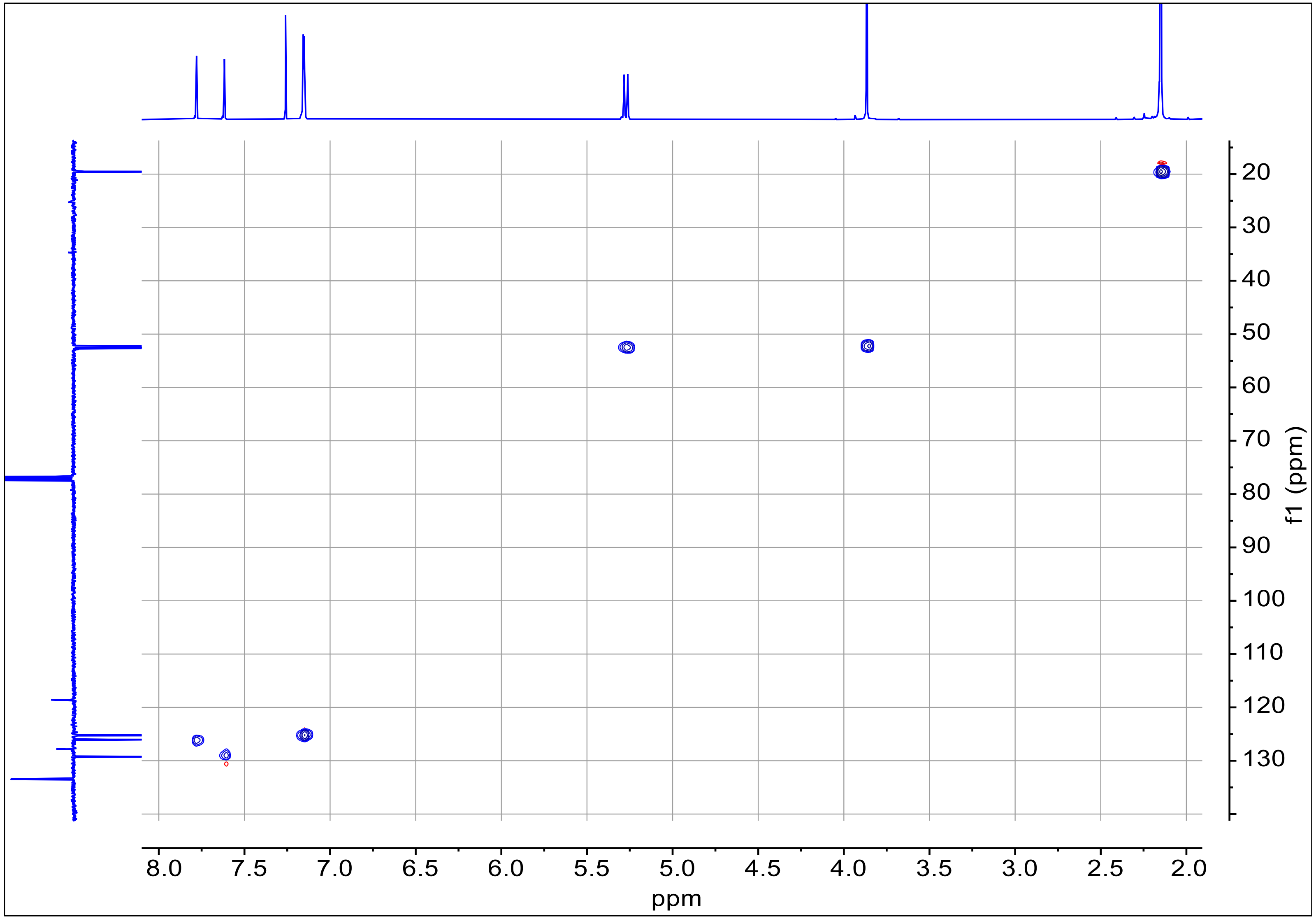

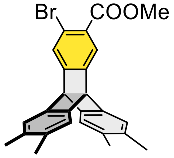

HMBC ($CDCl_3$): Compound **8**

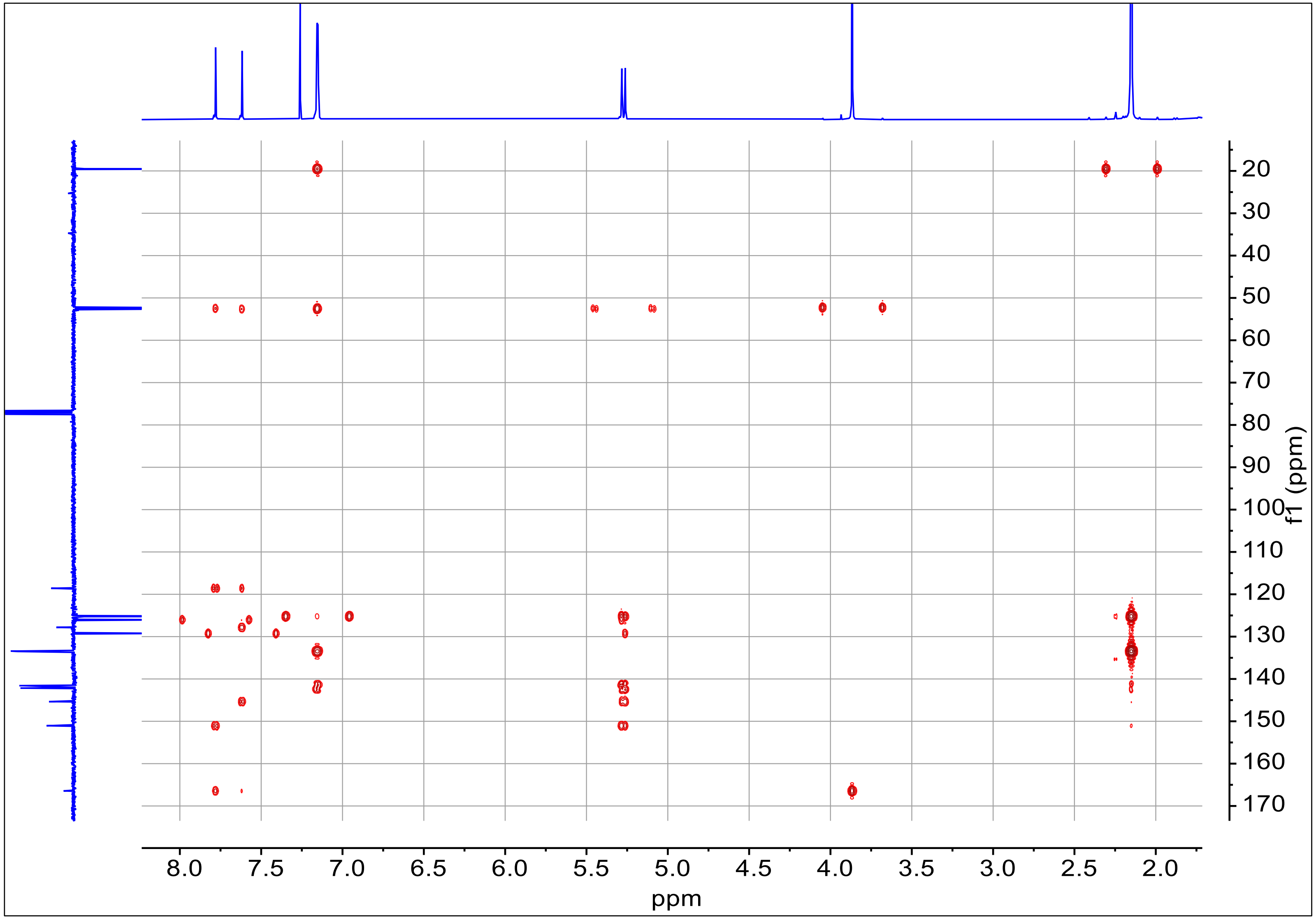

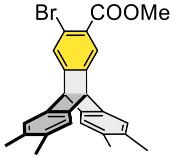

^{1}H NMR (400 MHz, $CDCl_3$): Compound **9**

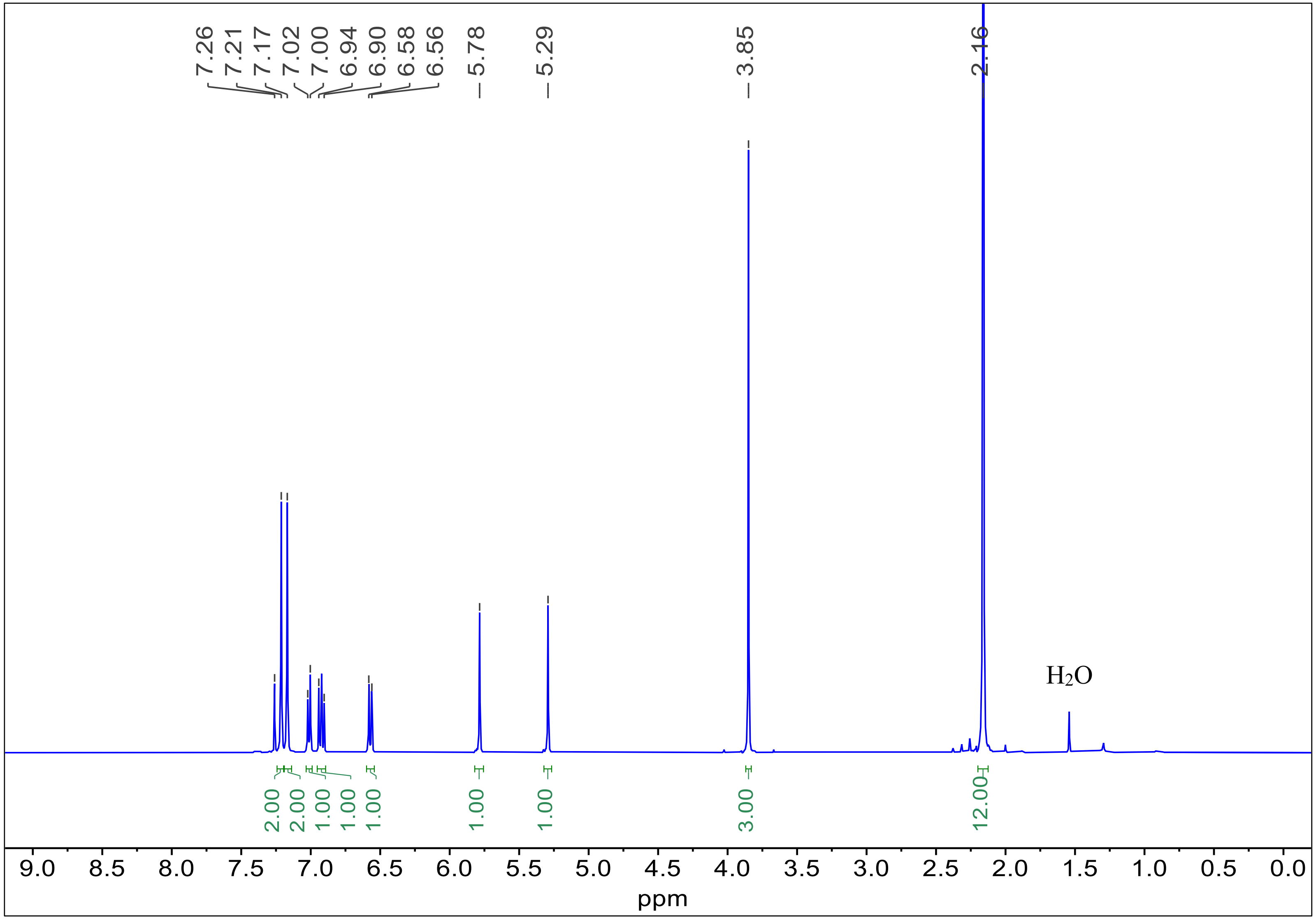

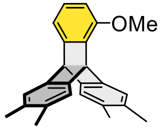

^{13}C {^{1}H} NMR (100 MHz, $CDCl_3$): Compound **9**

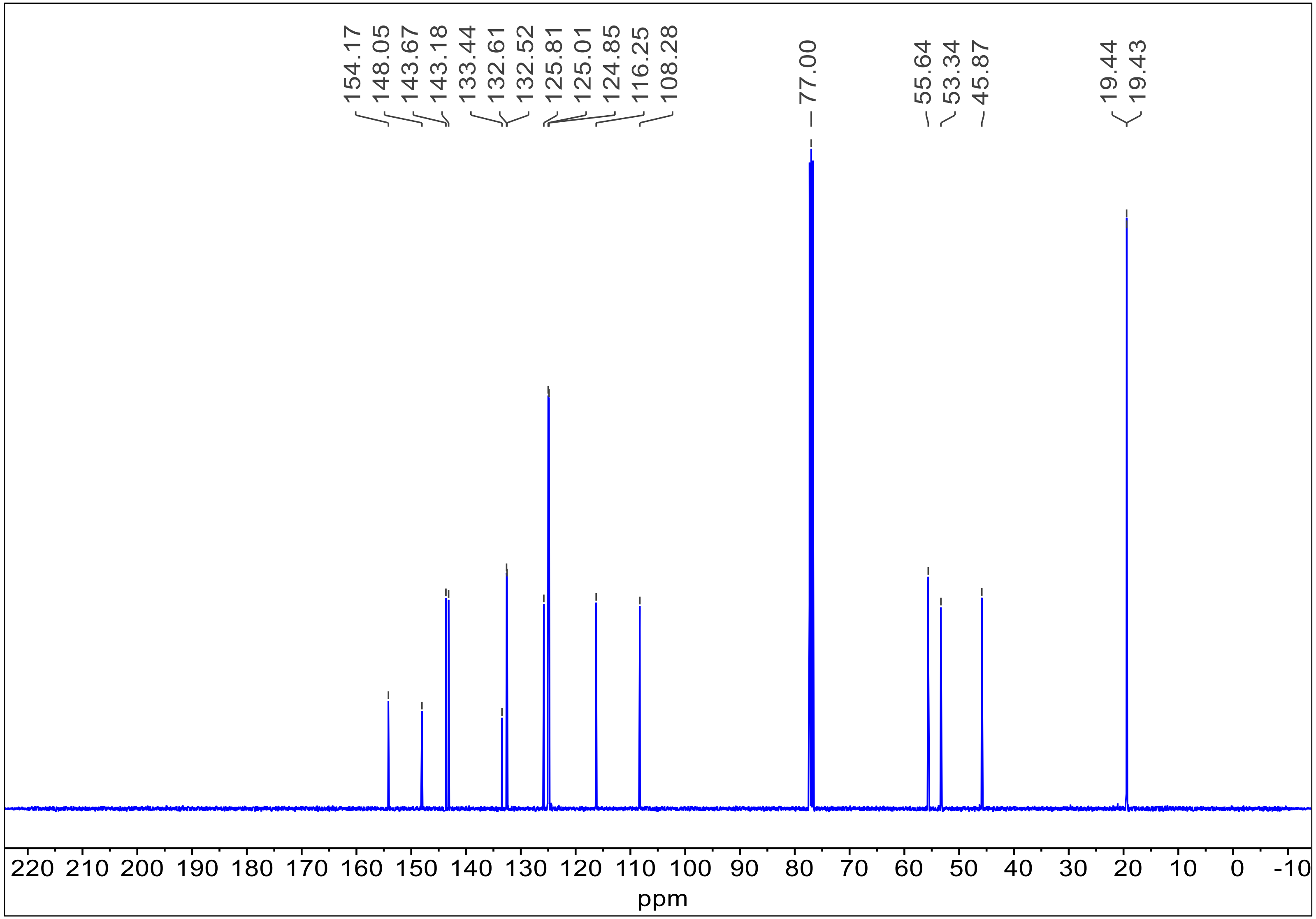

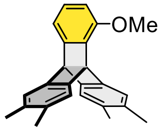

^{13}C {^{1}H} APT NMR (100 MHz, $CDCl_3$): Compound **9**

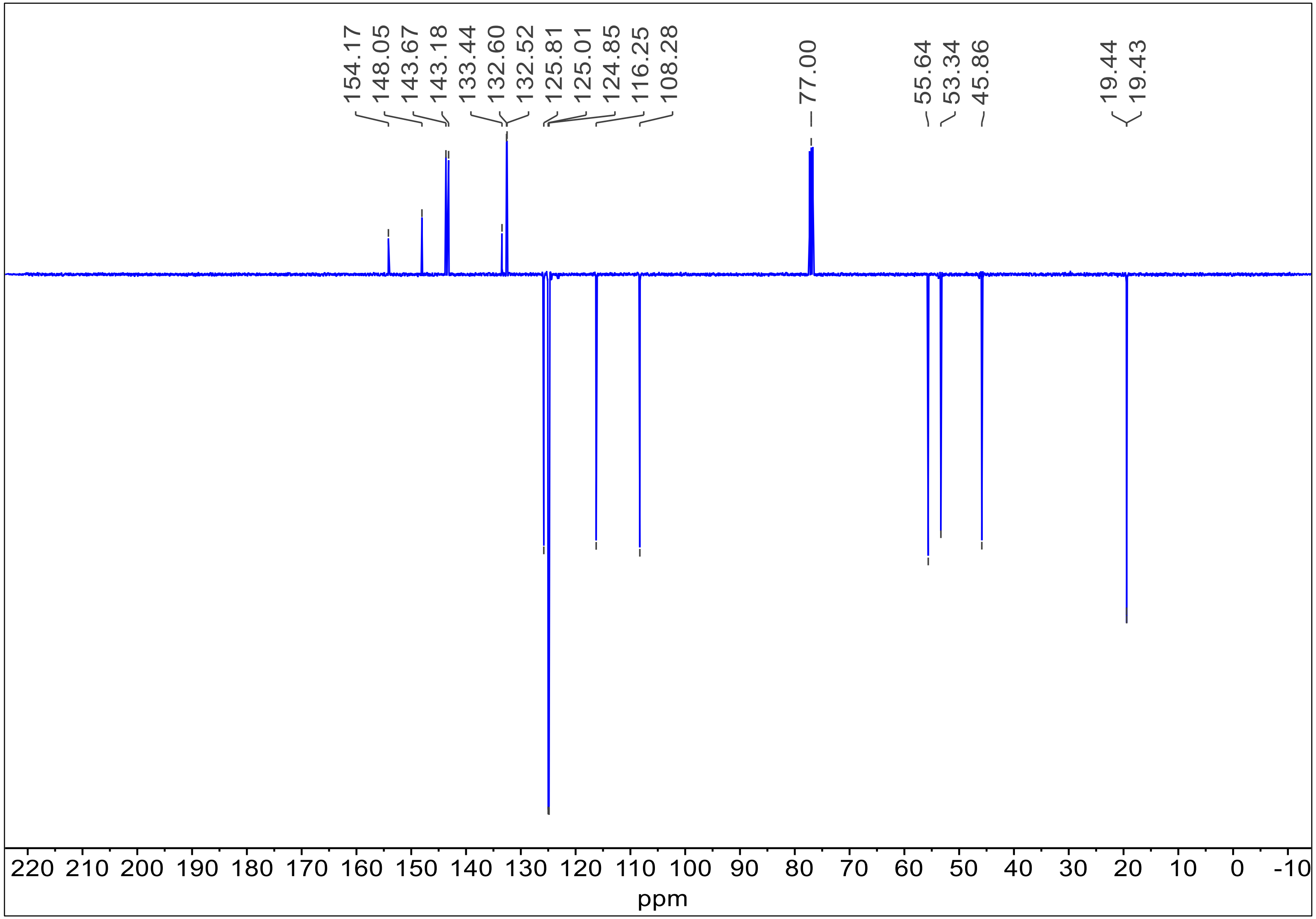

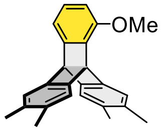

^{1}H – ^{1}H COSY ($CDCl_3$): Compound **9**

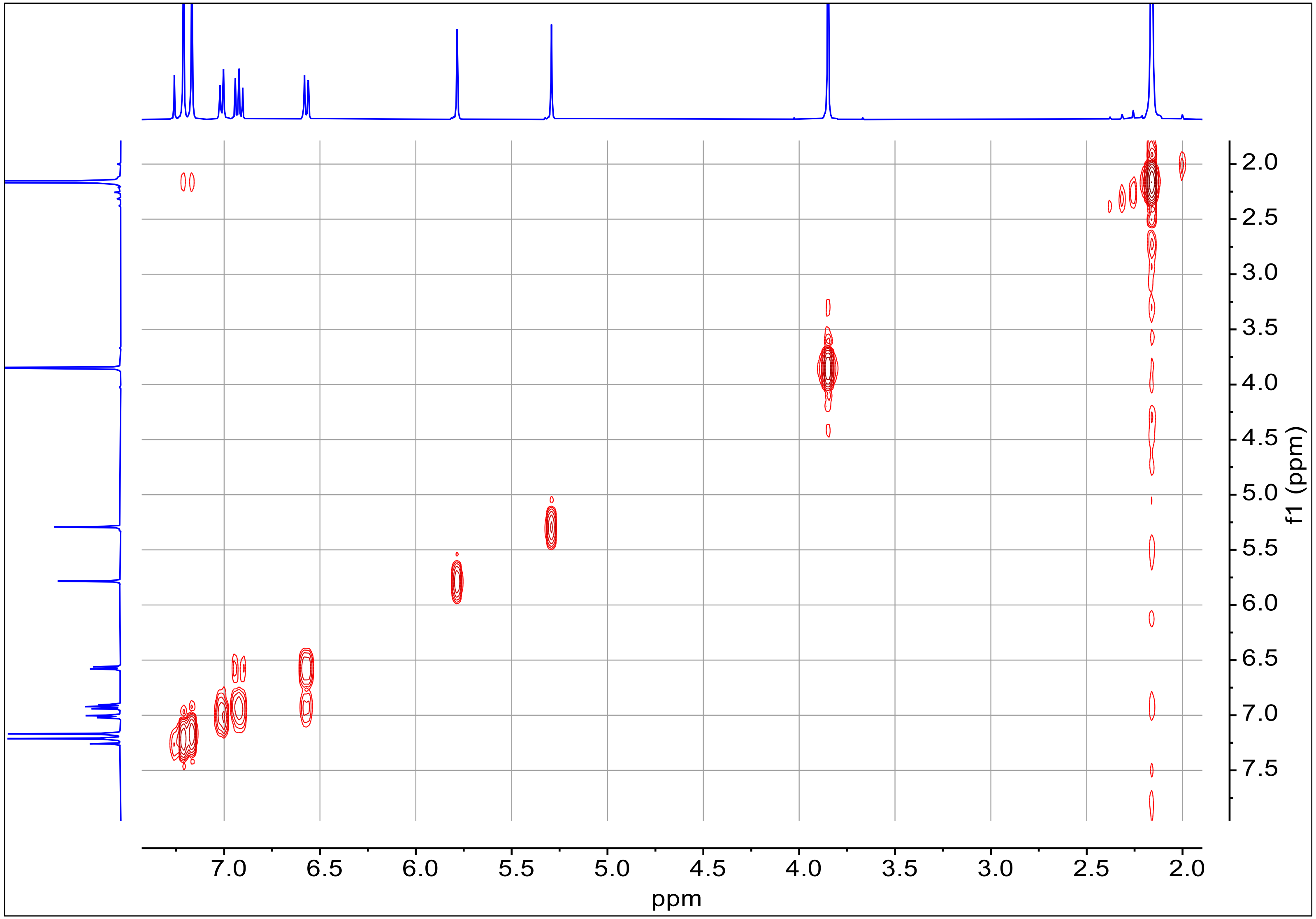

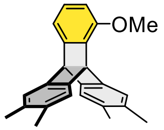

HSQC ($CDCl_3$): Compound **9**

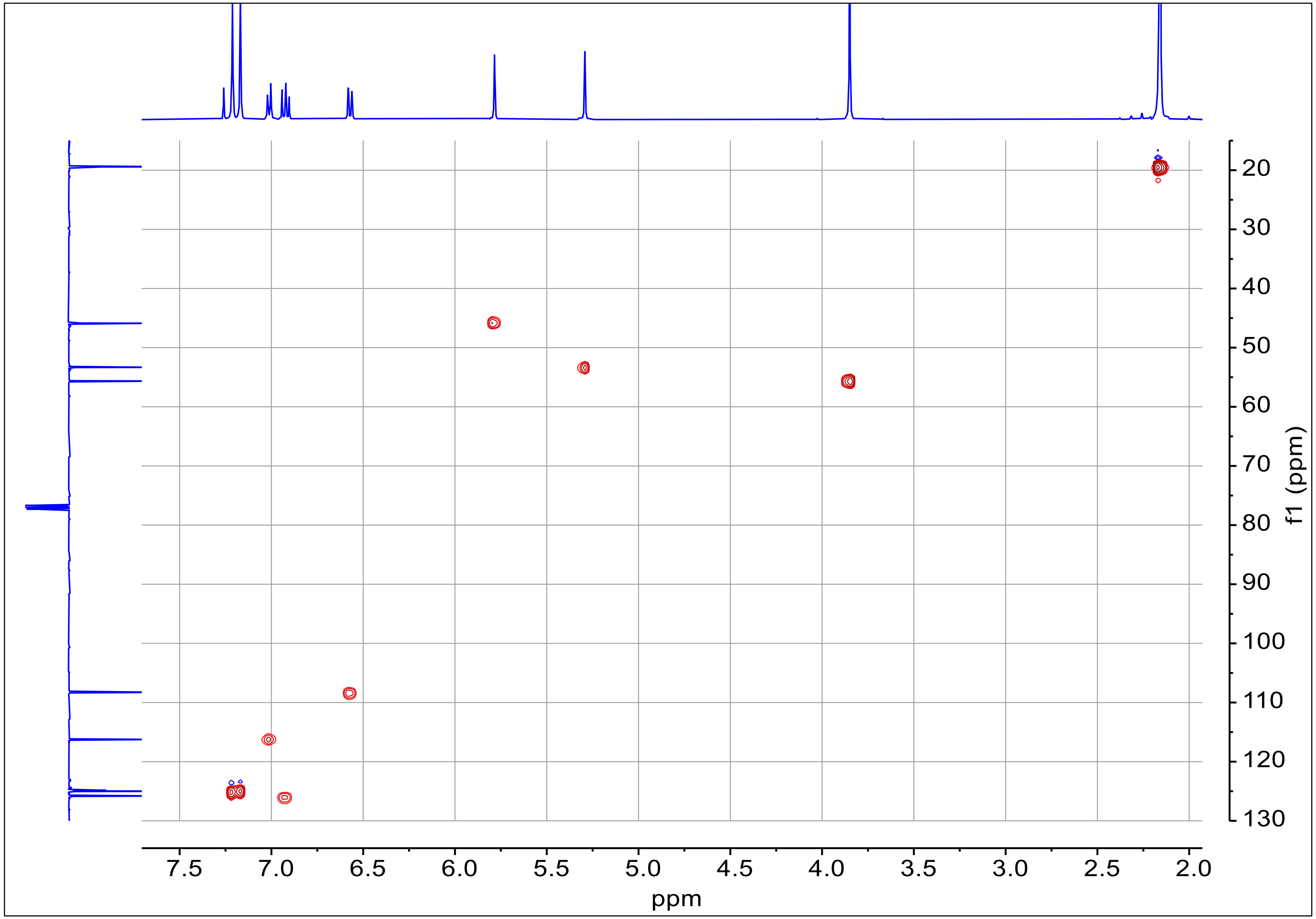

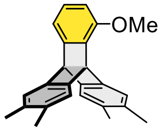

HMBC ($CDCl_3$): Compound **9**

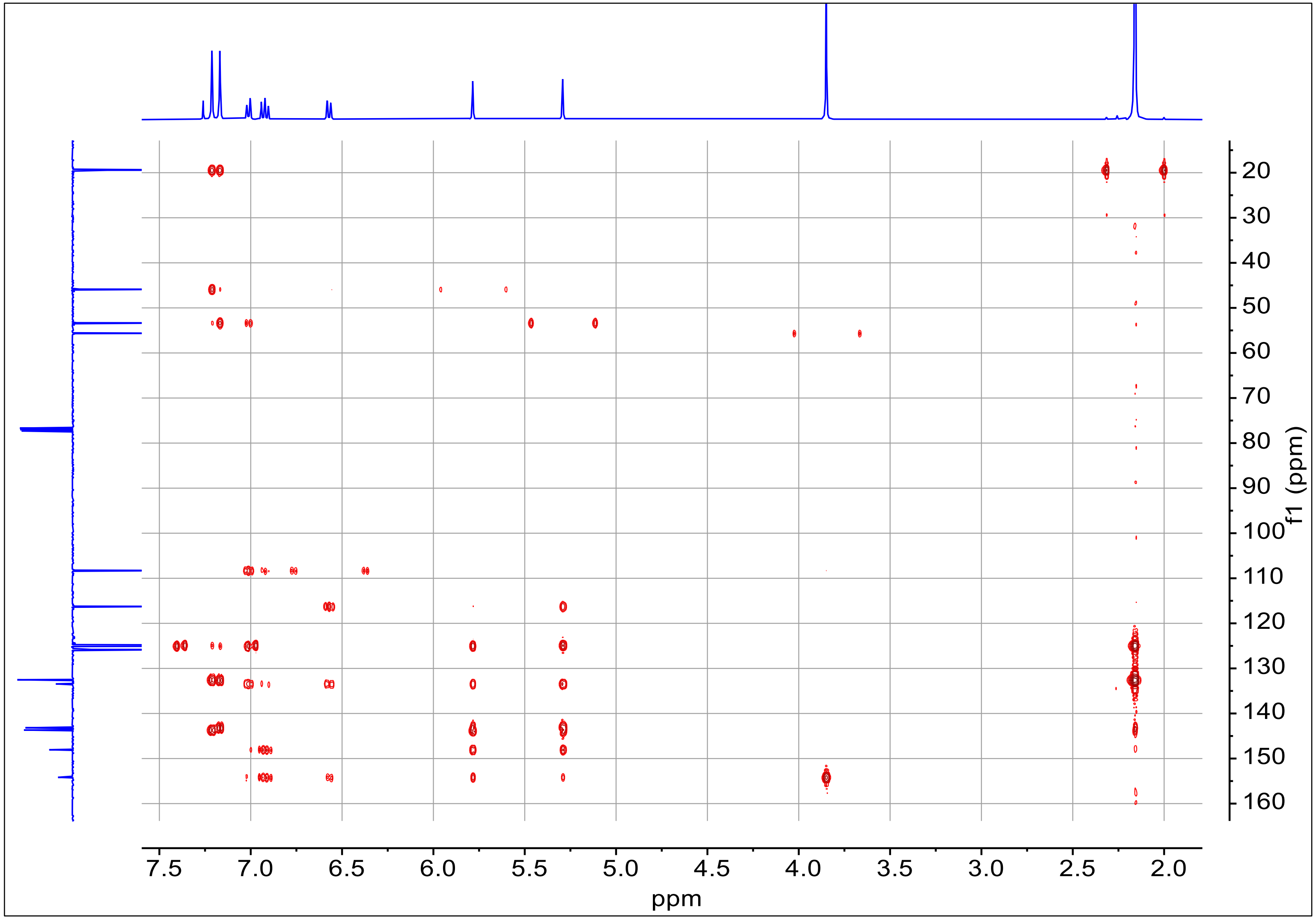

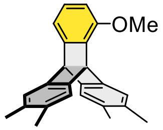

^{1}H NMR (400 MHz, CDCl3): Compound **10**

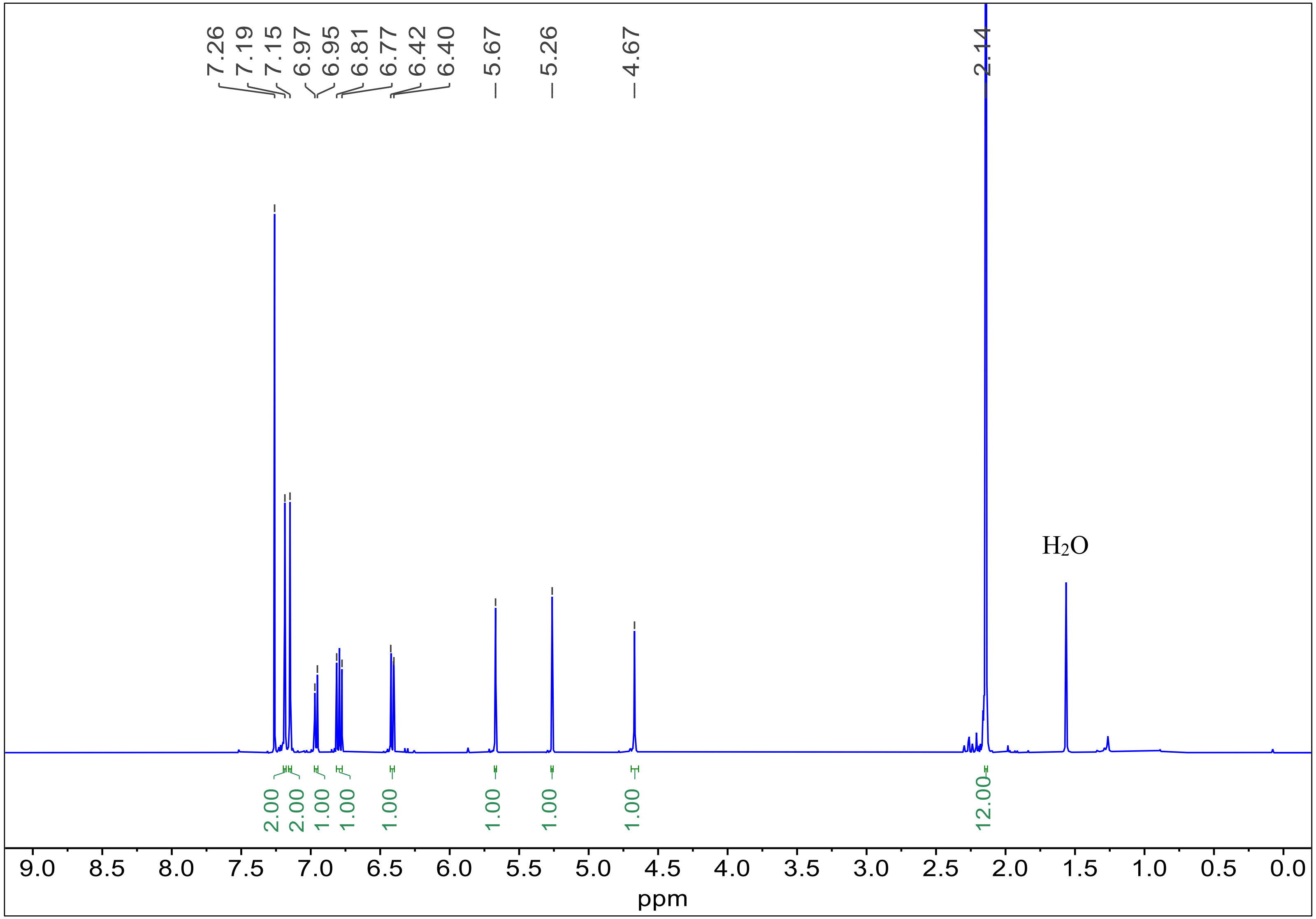

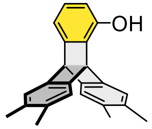

^{13}C {^{1}H} NMR (100 MHz, $CDCl_3$): Compound **10**

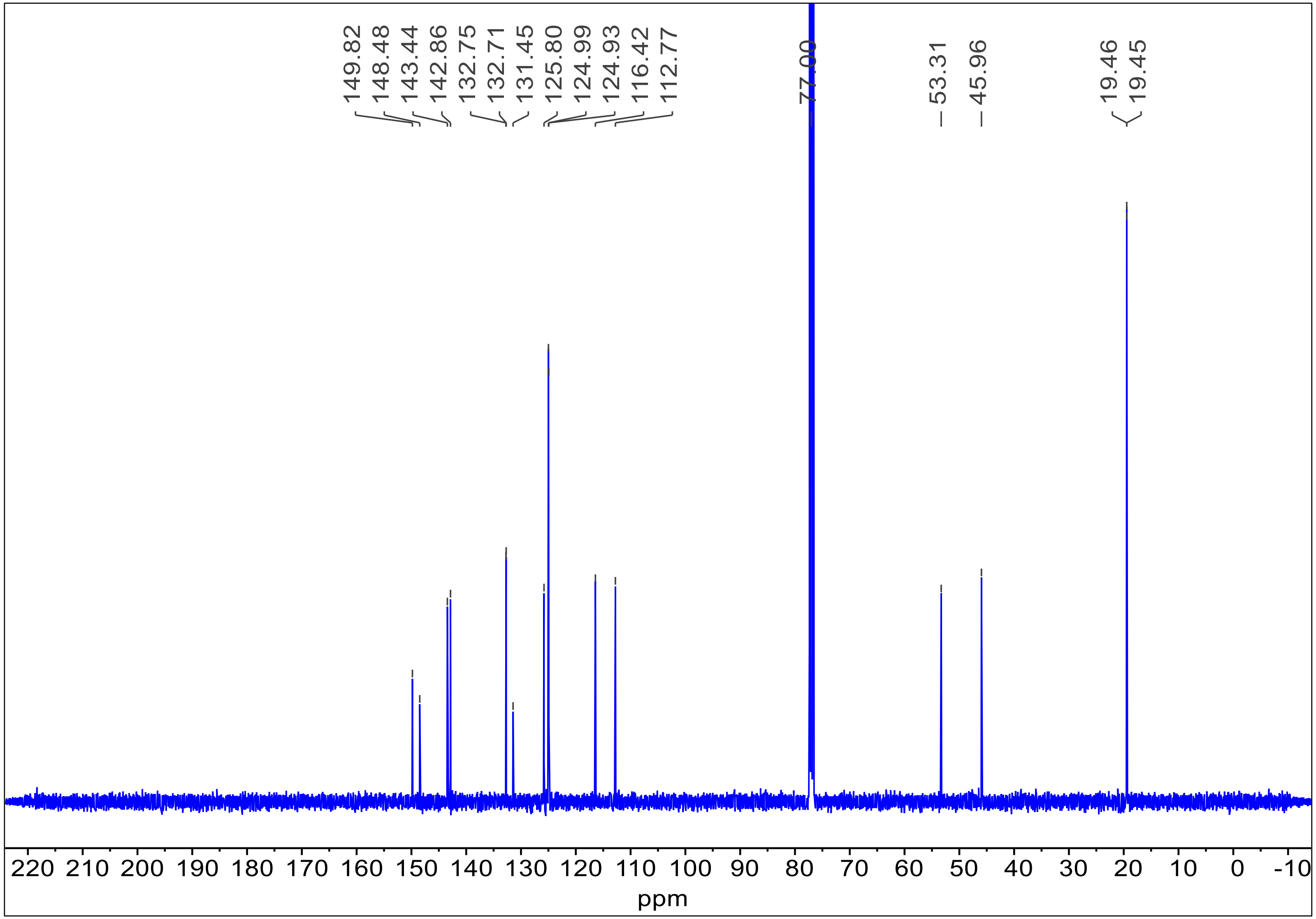

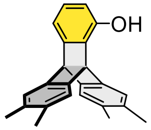

^{13}C {^{1}H} APT NMR (100 MHz, $CDCl_3$): Compound **10**

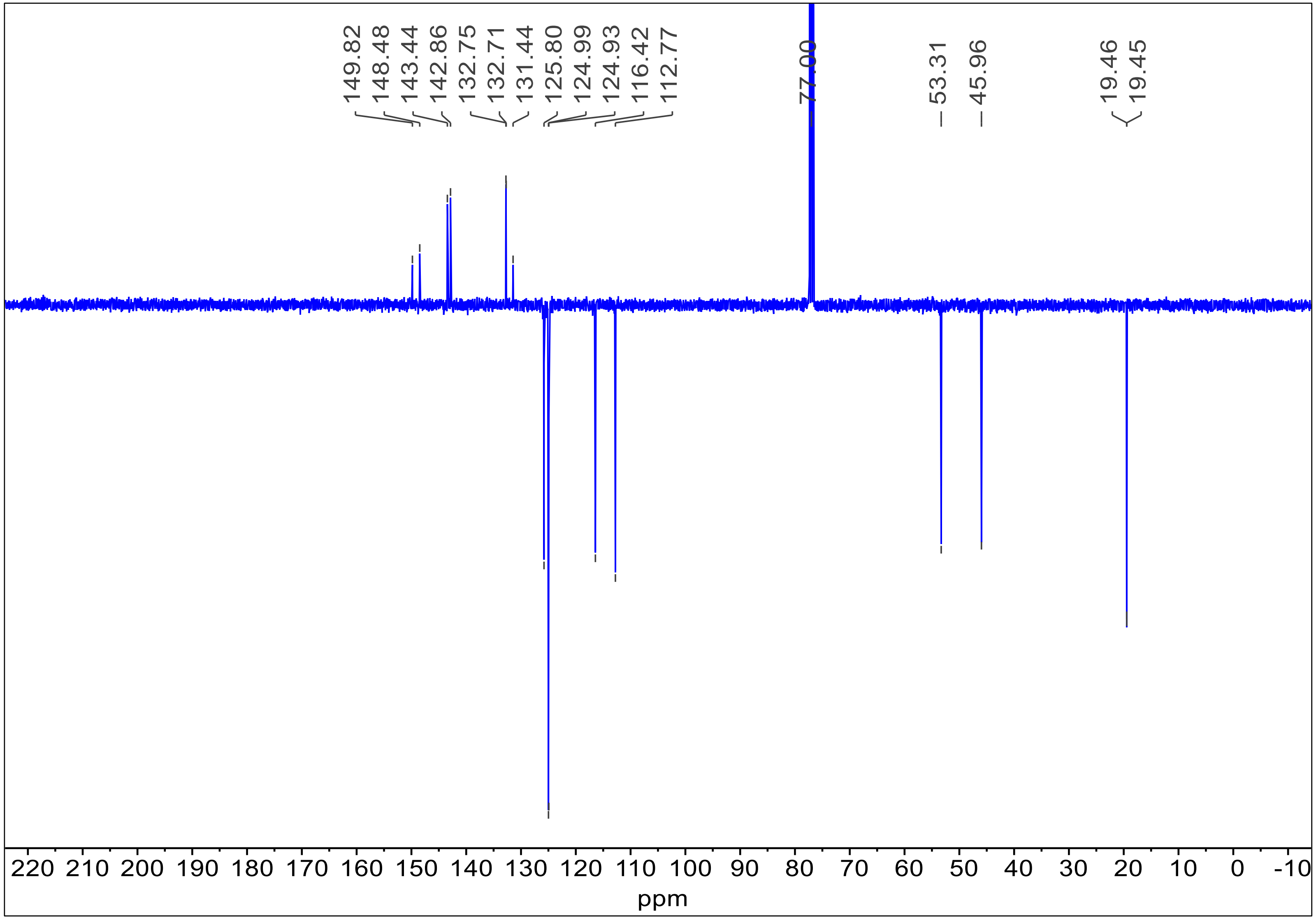

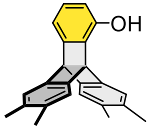

^{1}H – ^{1}H COSY ($CDCl_3$): Compound **10**

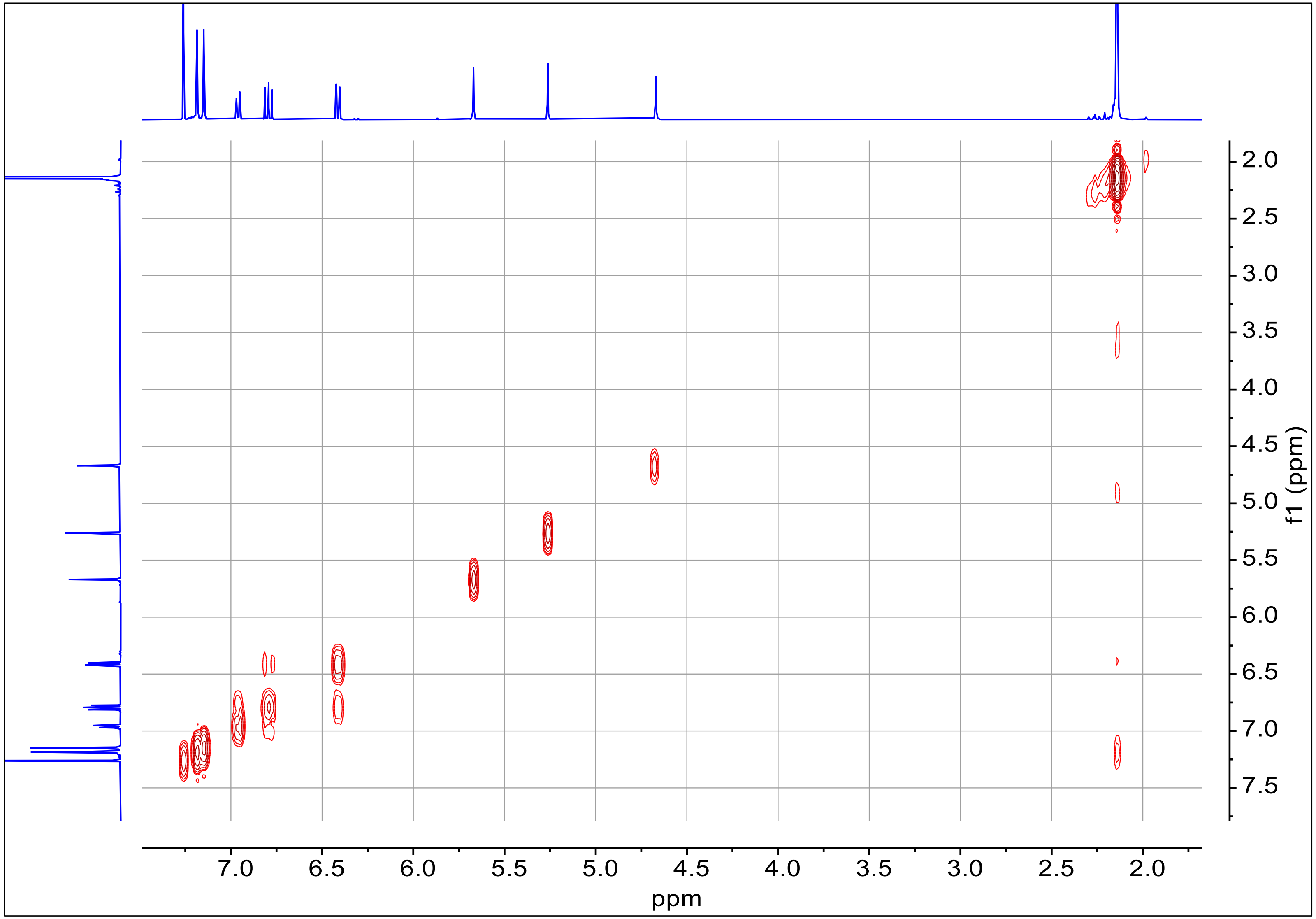

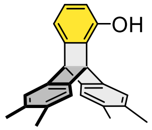

HSQC ($CDCl_3$): Compound **10**

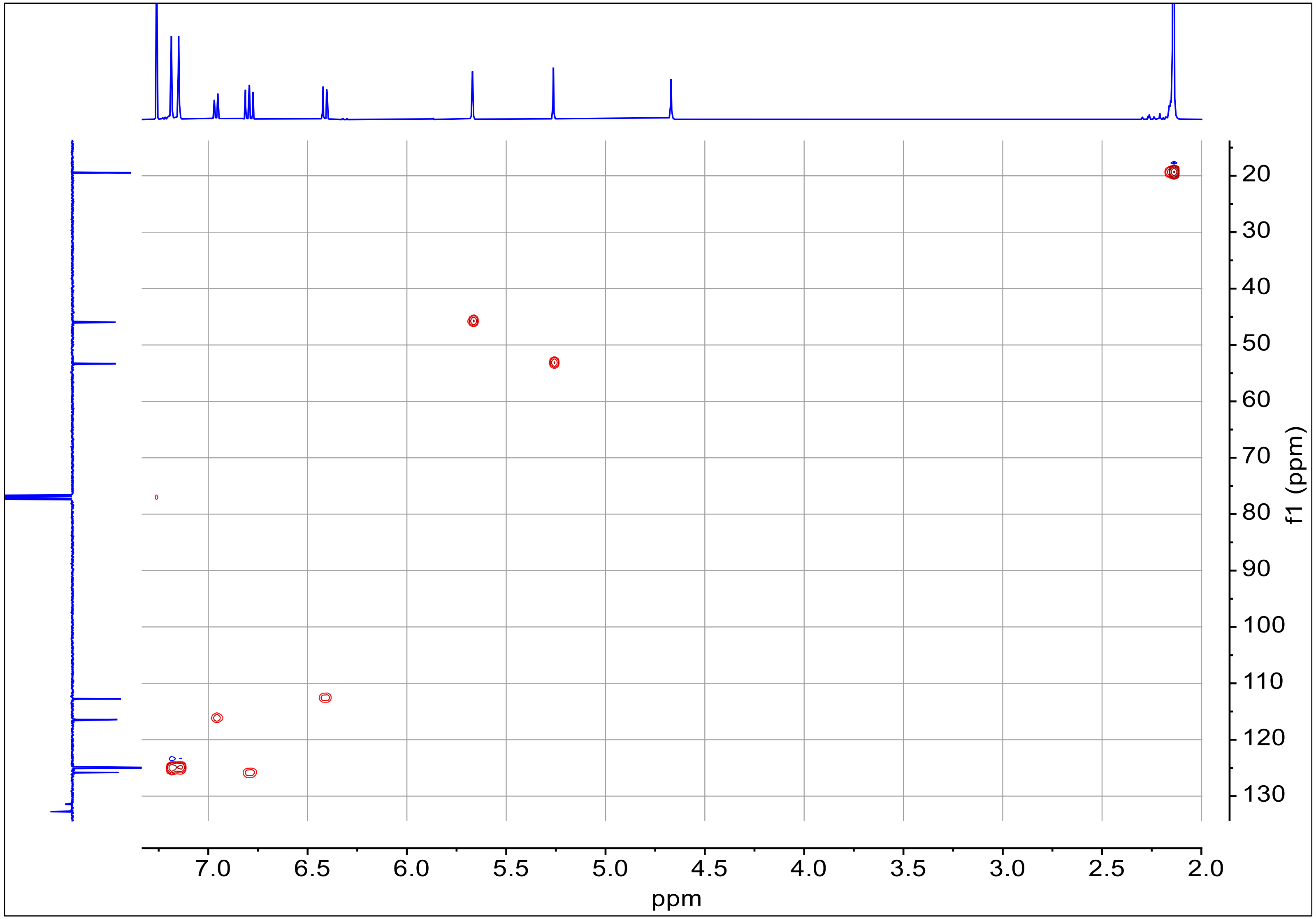

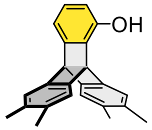

HMBC ($CDCl_3$): Compound **10**

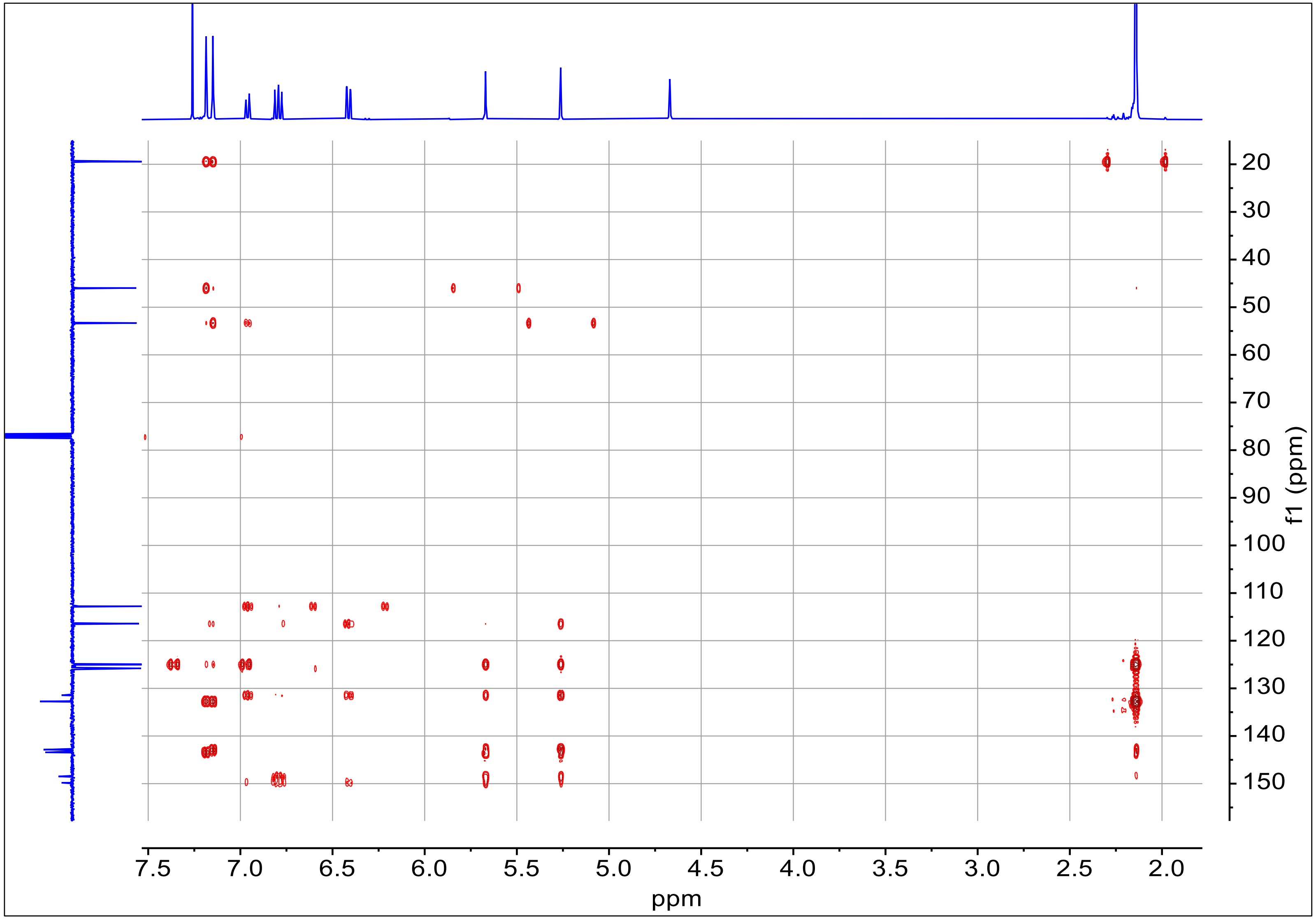

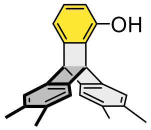

^{1}H NMR (400 MHz, $CDCl_3$): Compound **11**

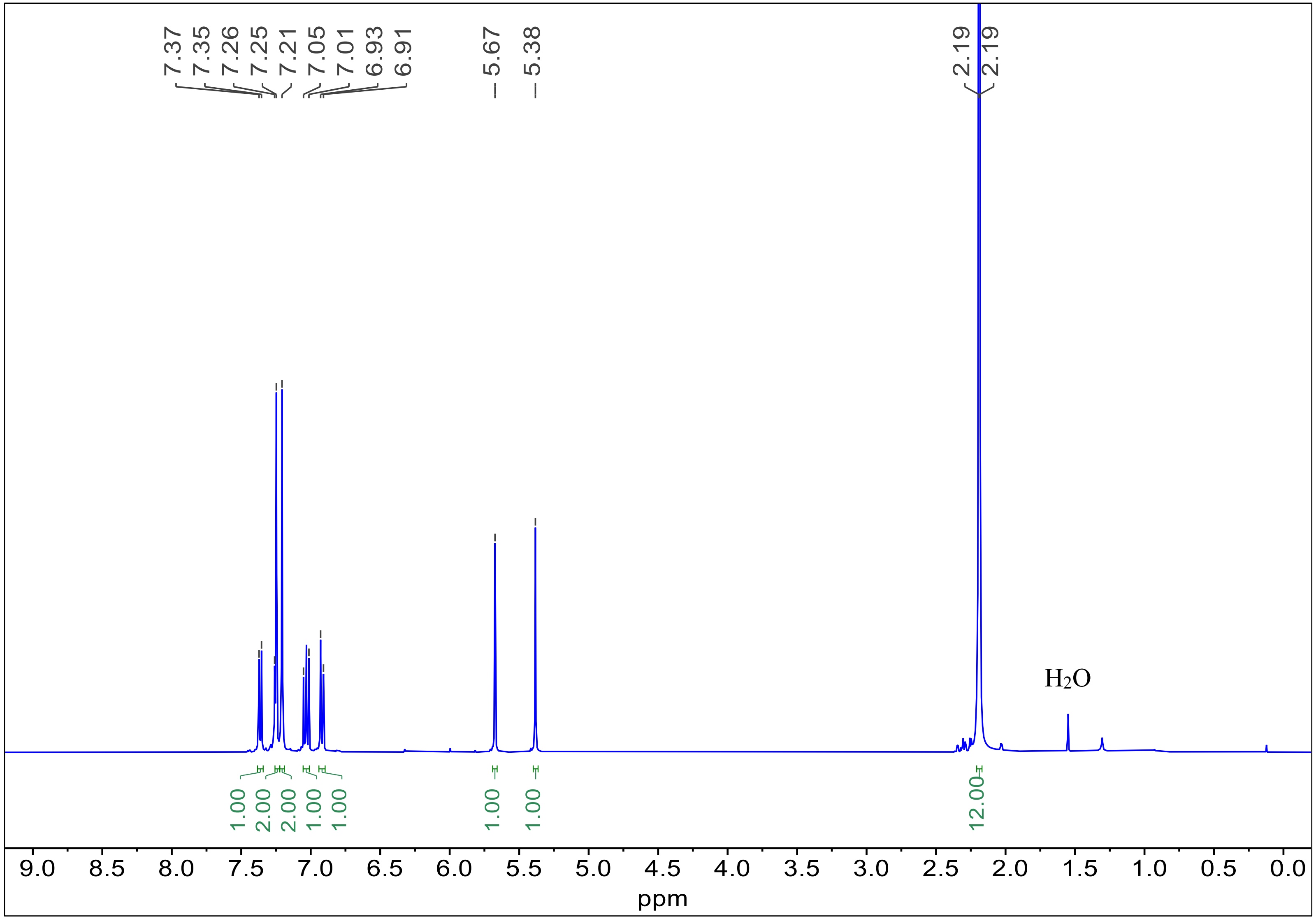

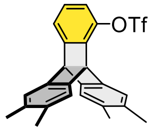

^{19}F NMR (377 MHz, $CDCl_3$): Compound **11**

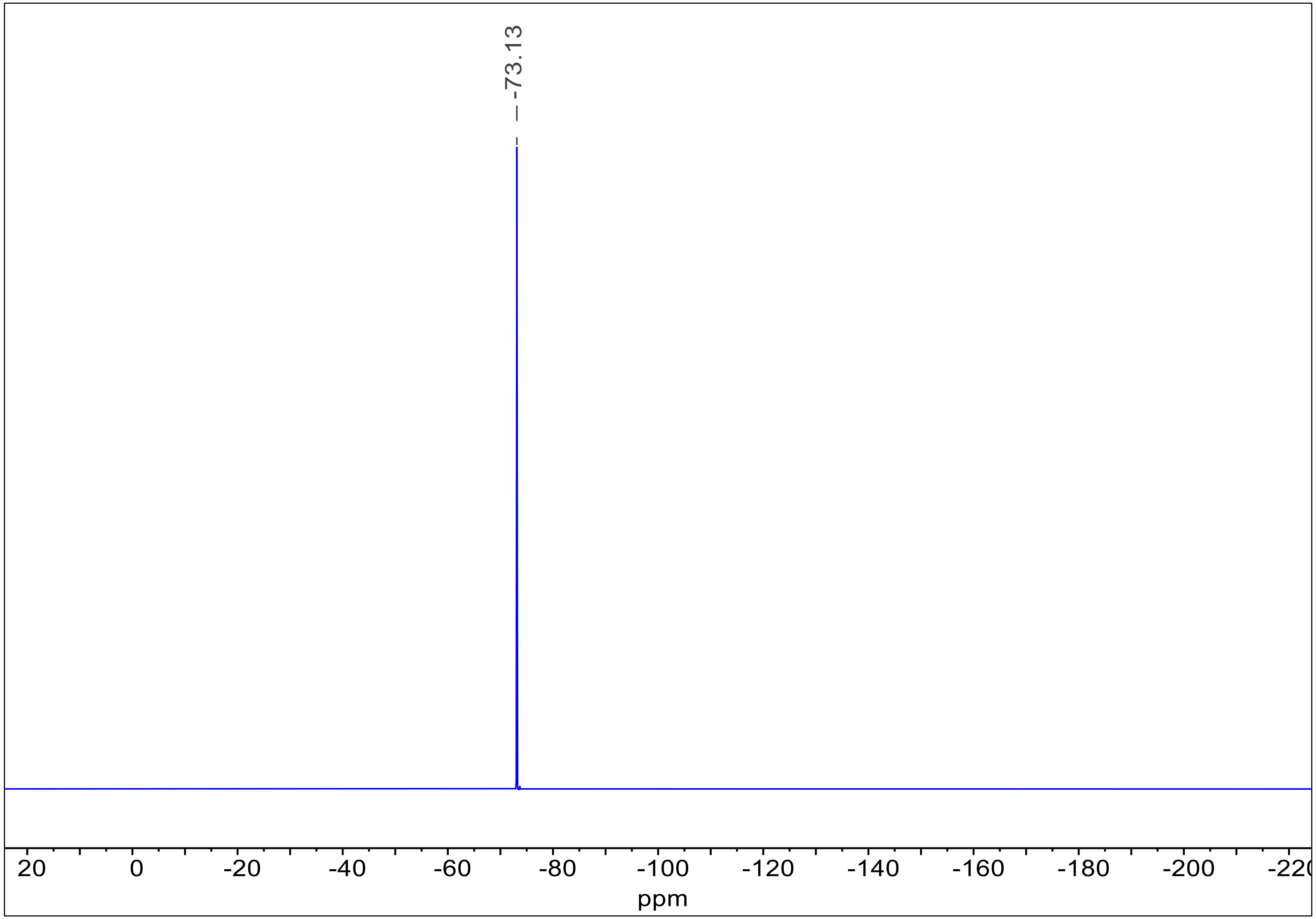

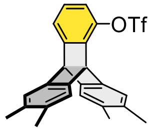

$^{13}C\ \{^1H\}$ NMR (100 MHz, $CDCl_3$): Compound **11**

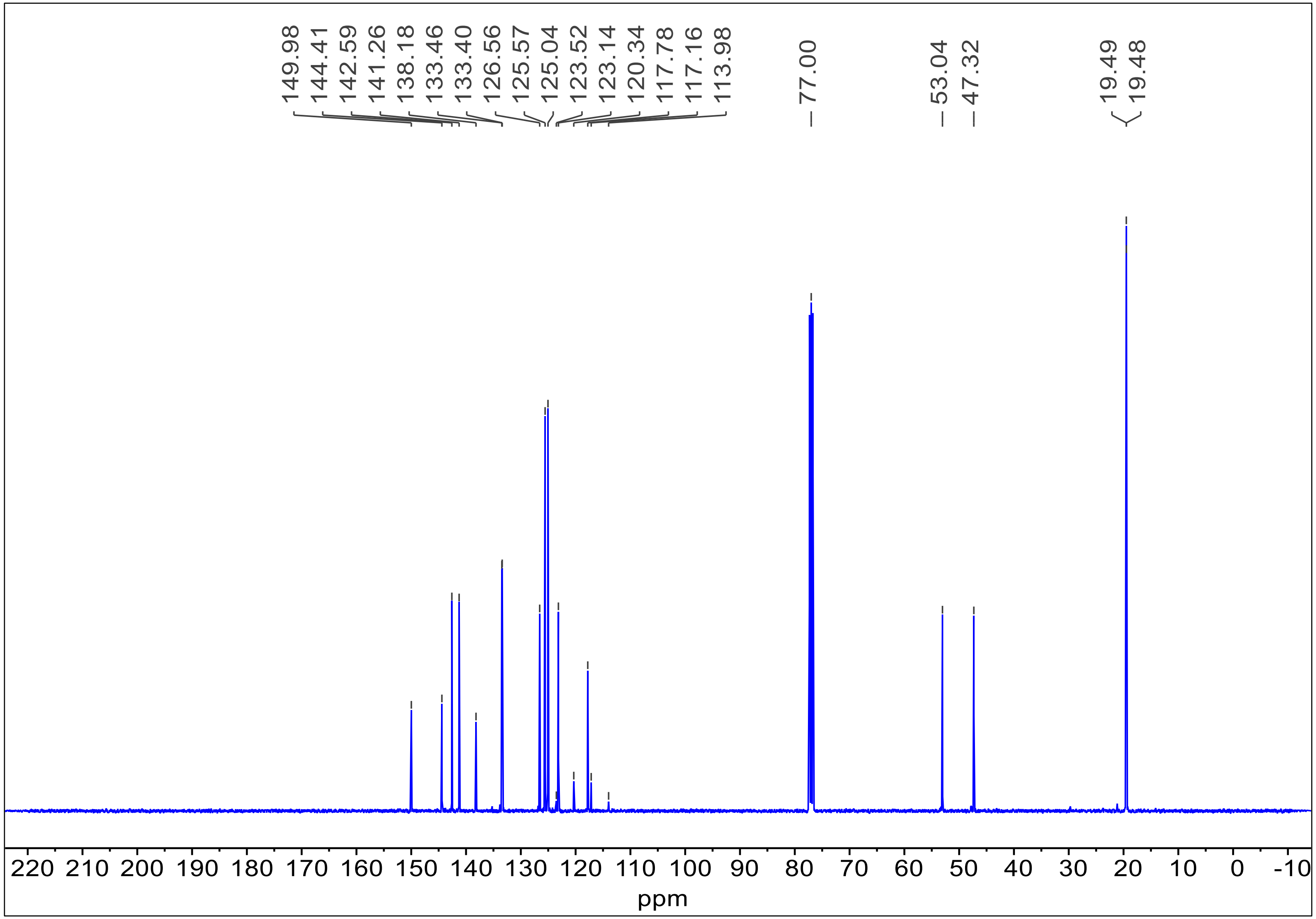

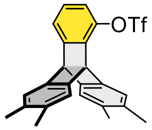

^{13}C {^{1}H} APT NMR (100 MHz, $CDCl_3$): Compound **11**

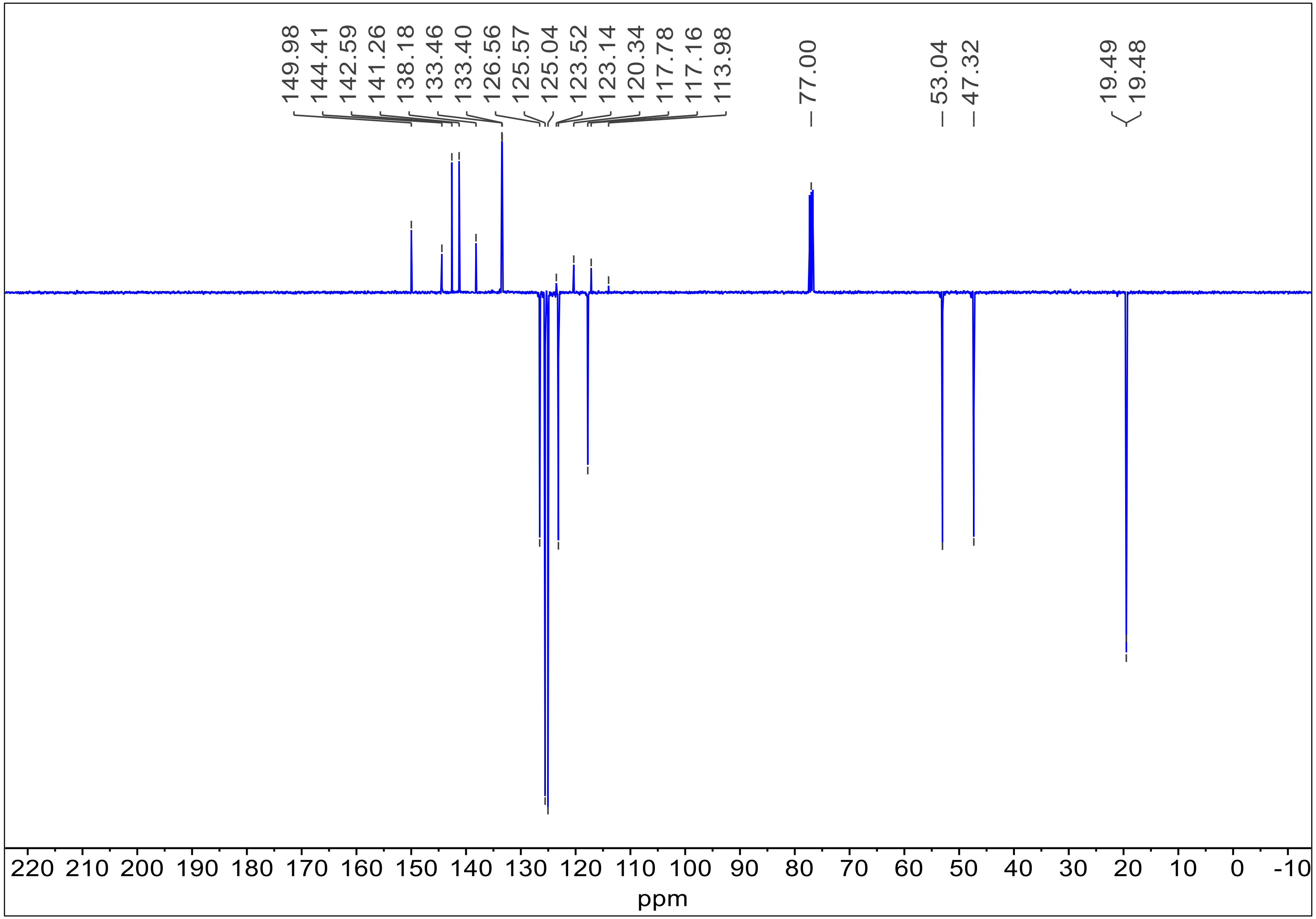

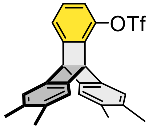

^{1}H – ^{1}H COSY ($CDCl_3$): Compound **11**

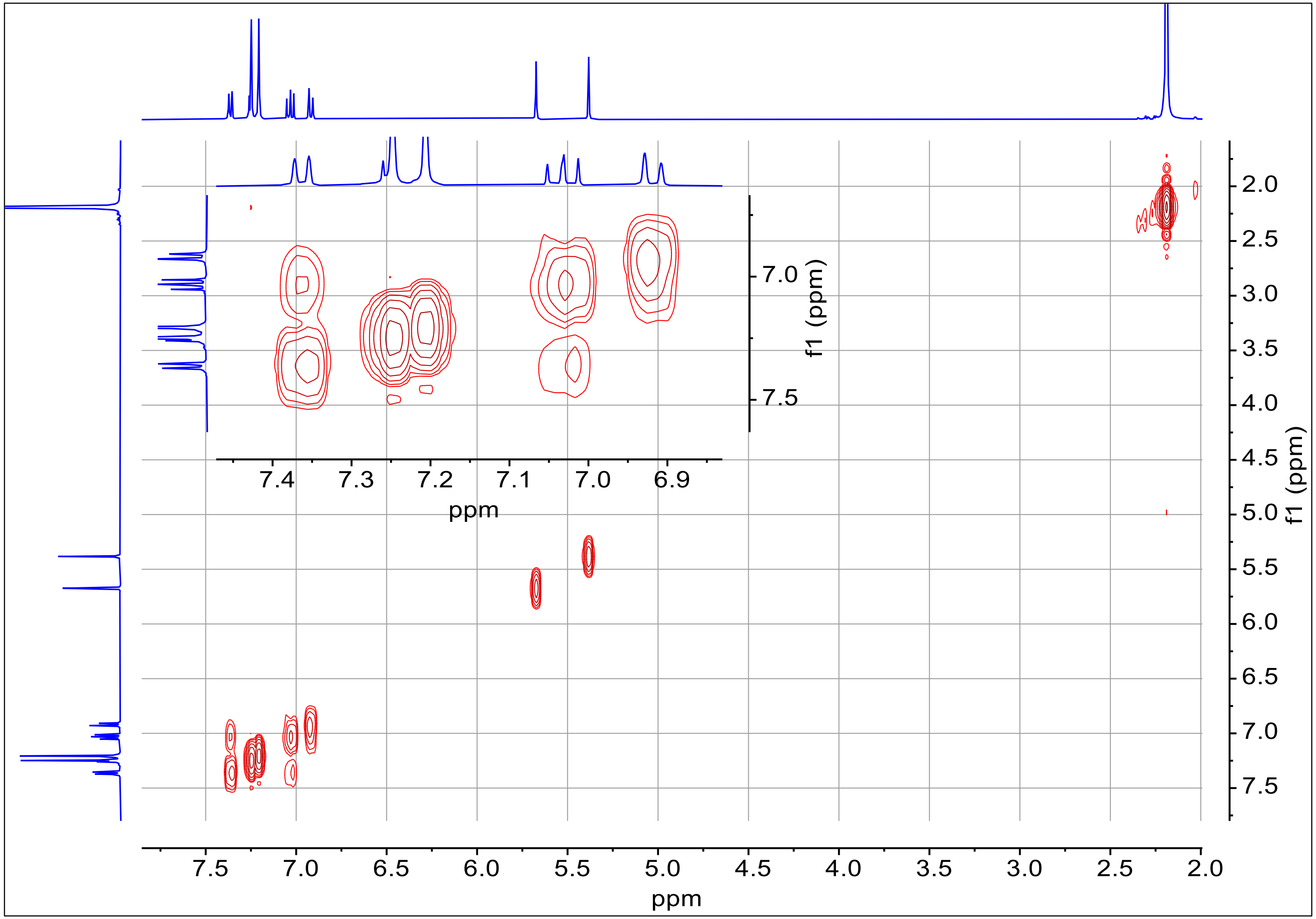

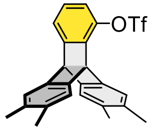

HSQC ($CDCl_3$): Compound **11**

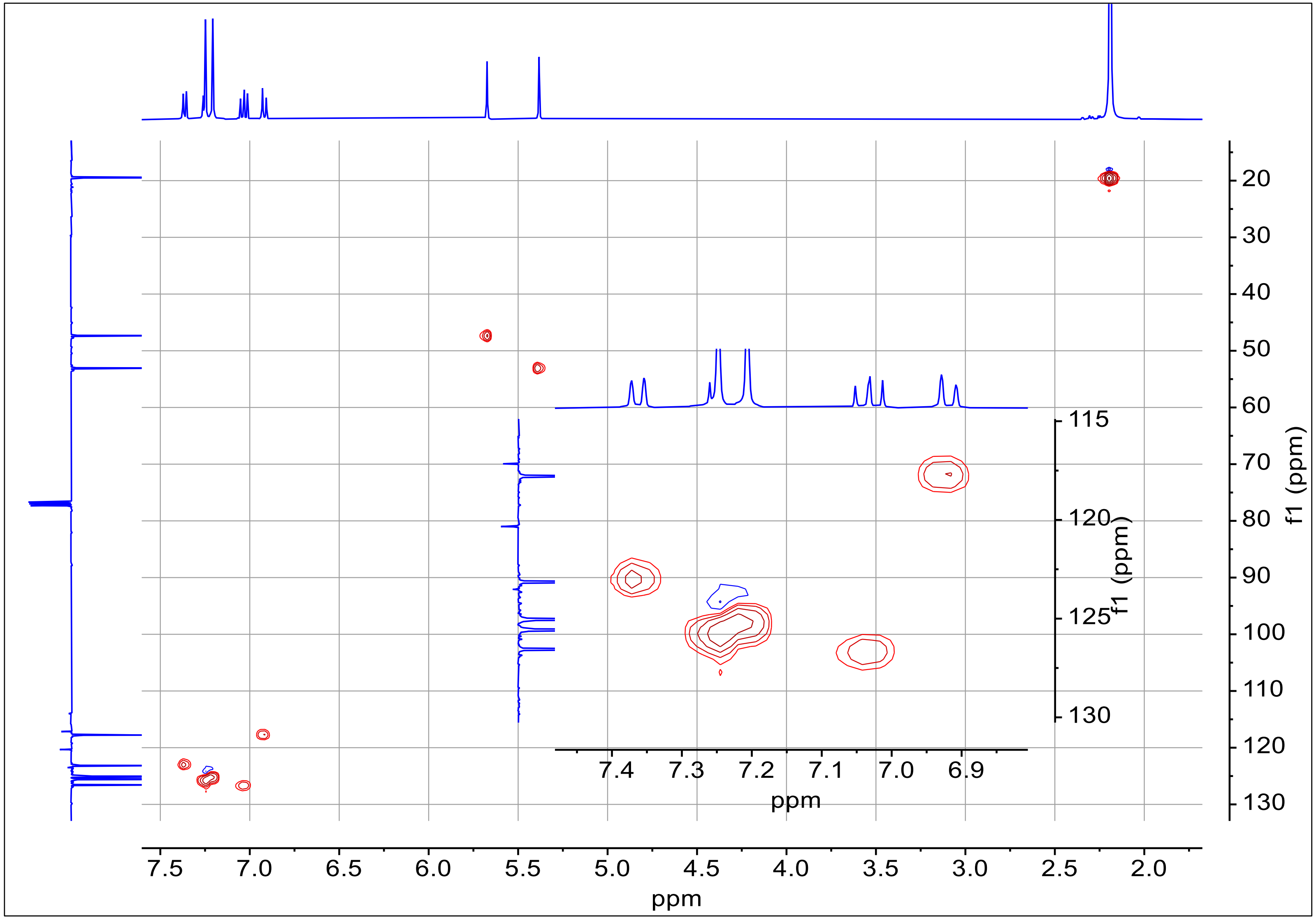

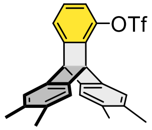

HMBC ($CDCl_3$): Compound **11**

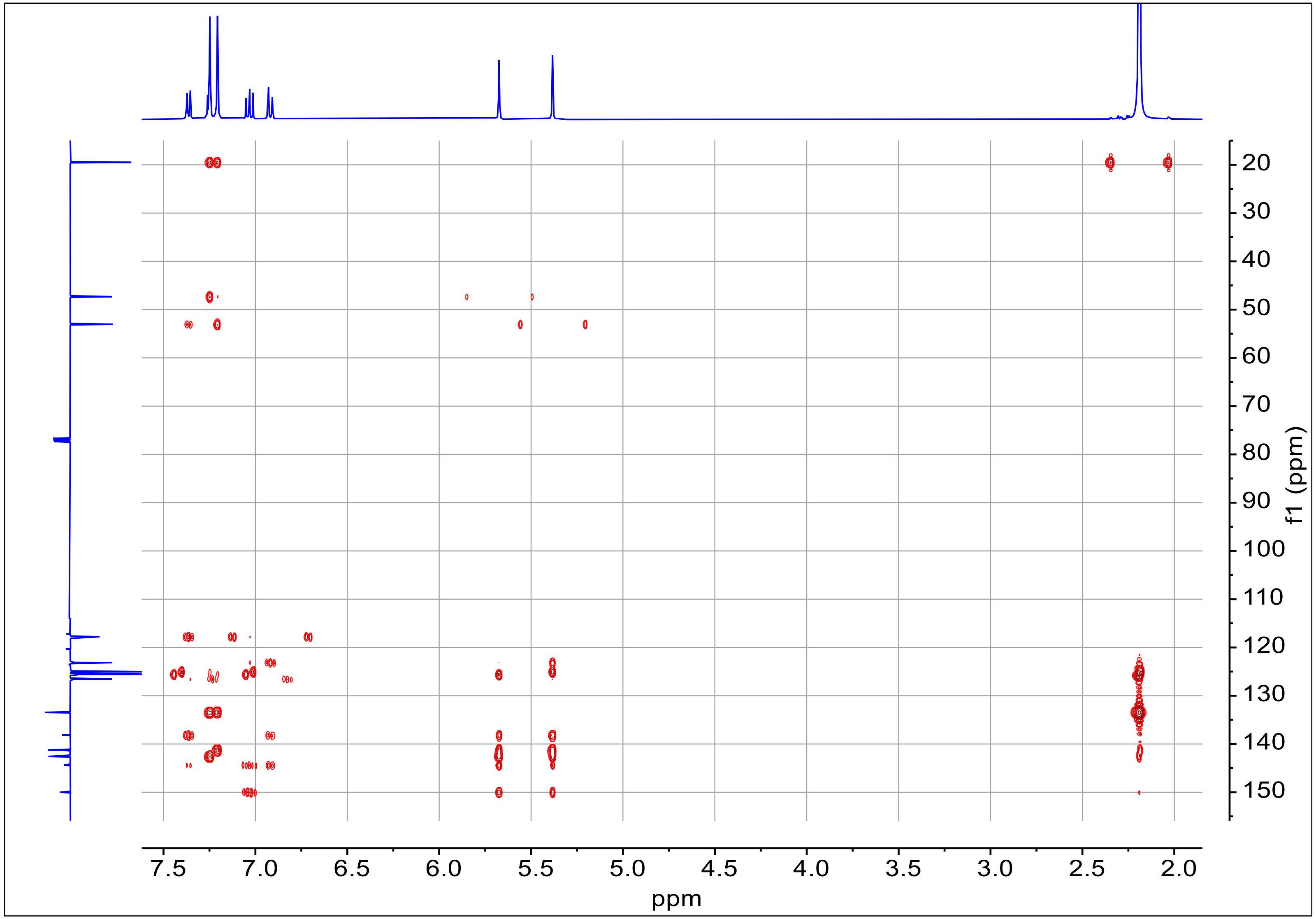

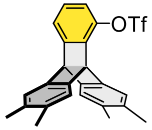

^{1}H NMR (400 MHz, $CDCl_3$): Compound **13**

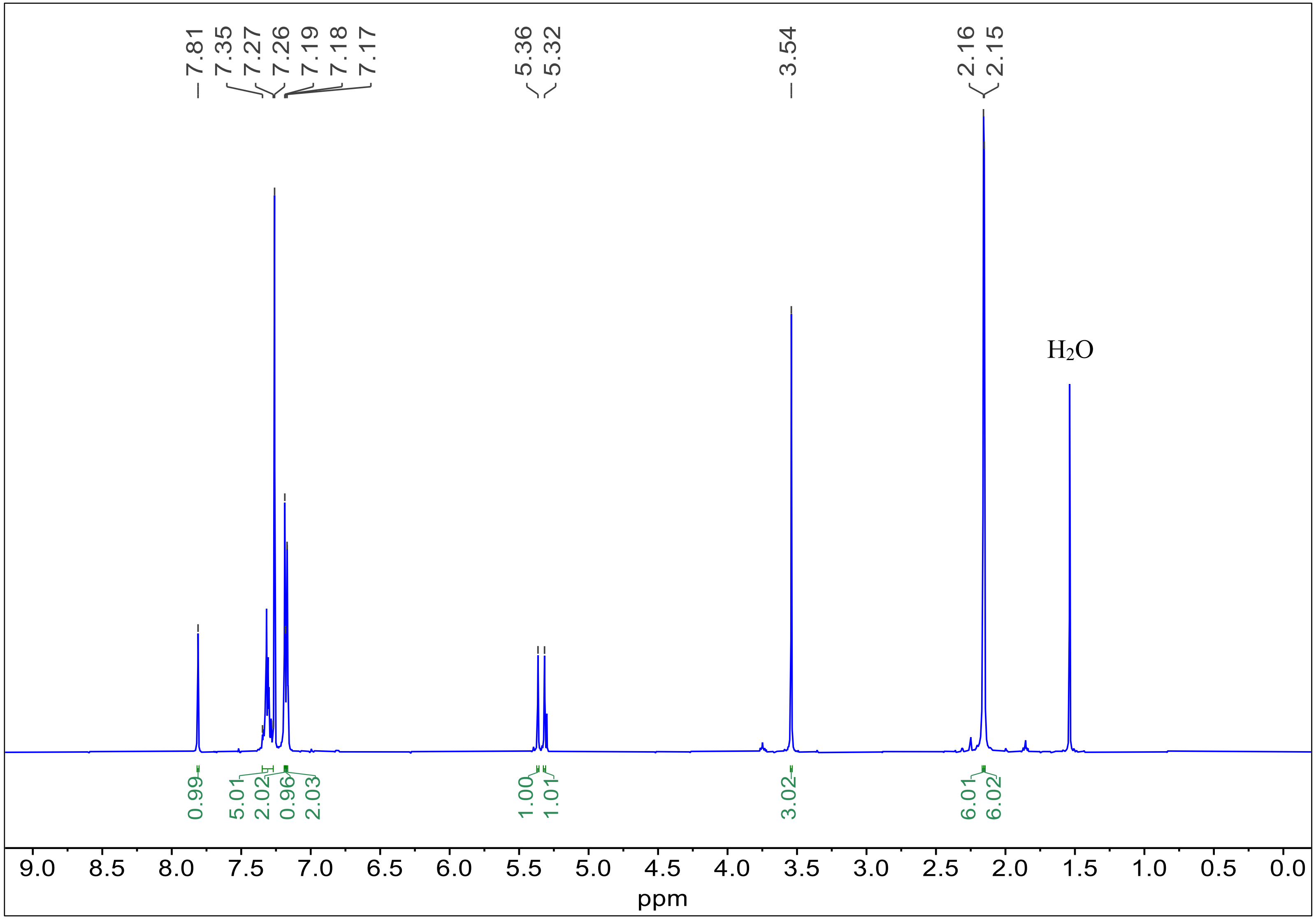

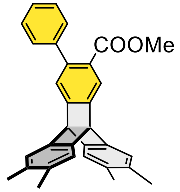

^{13}C {^{1}H} APT NMR (100 MHz, $CDCl_3$): Compound **13**

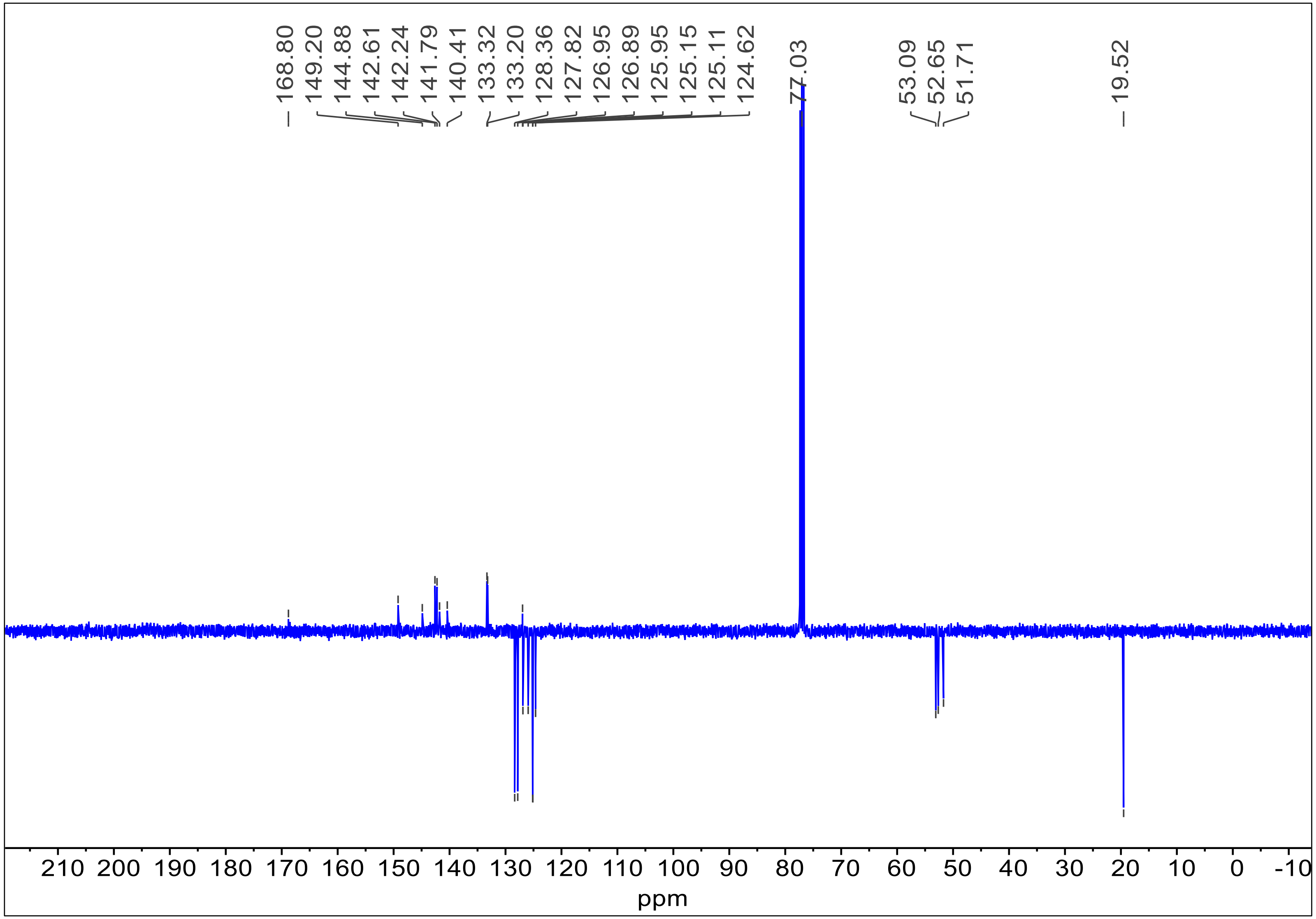

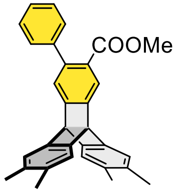

^{1}H – ^{1}H COSY ($CDCl_3$): Compound **13**

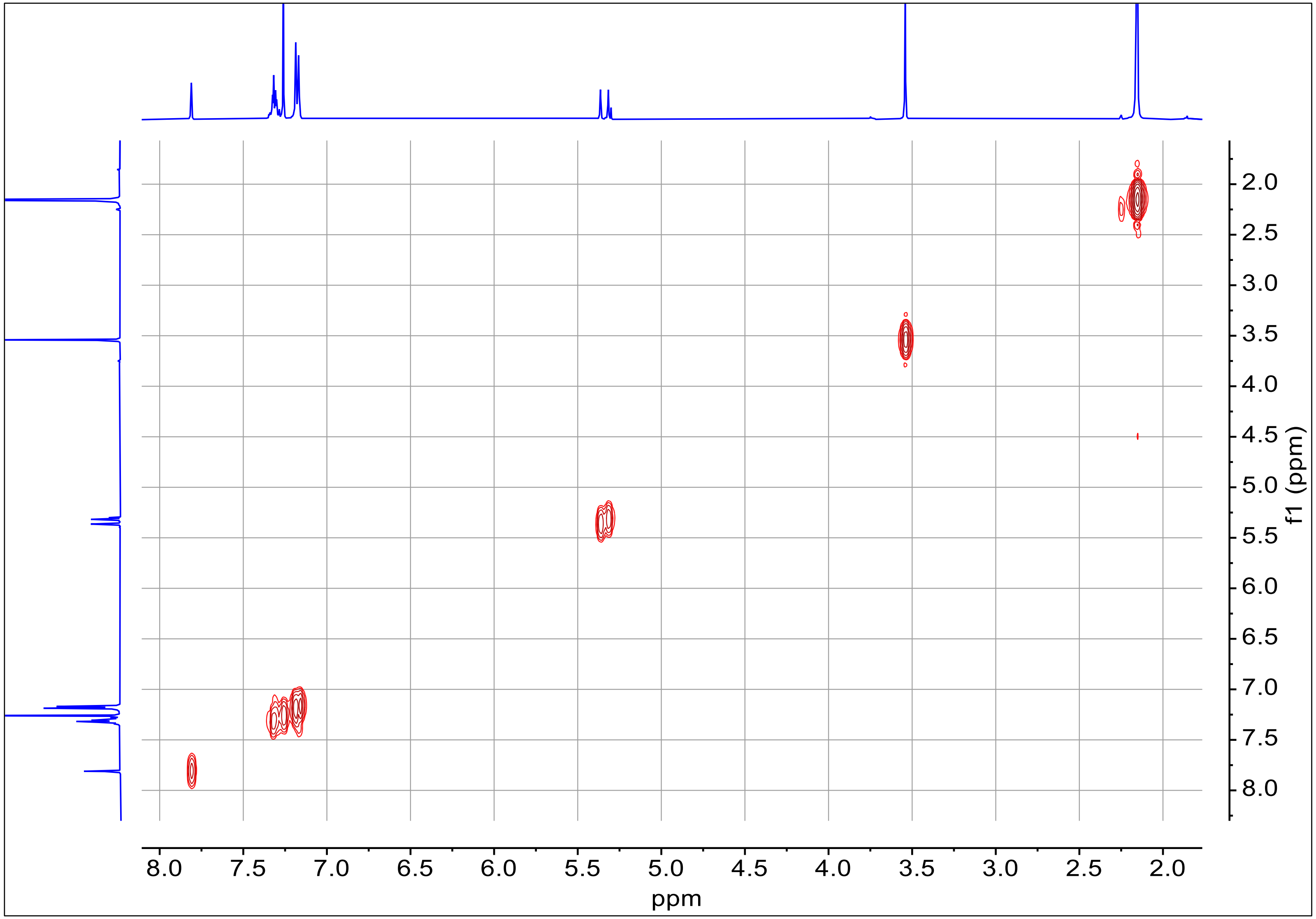

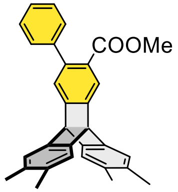

HSQC ($CDCl_3$): Compound **13**

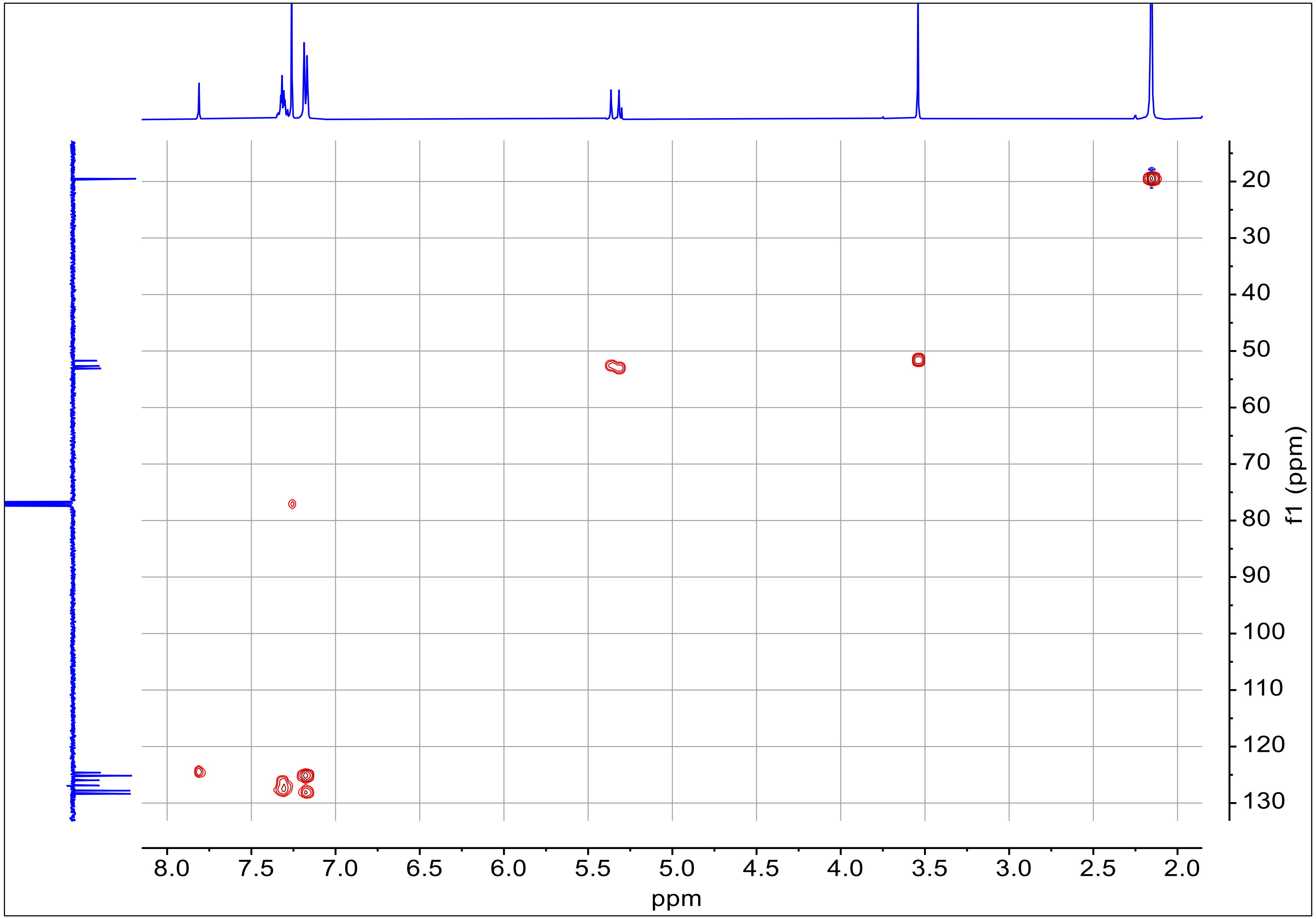

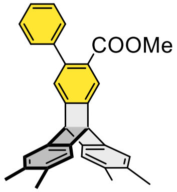

HMBC ($CDCl_3$): Compound **13**

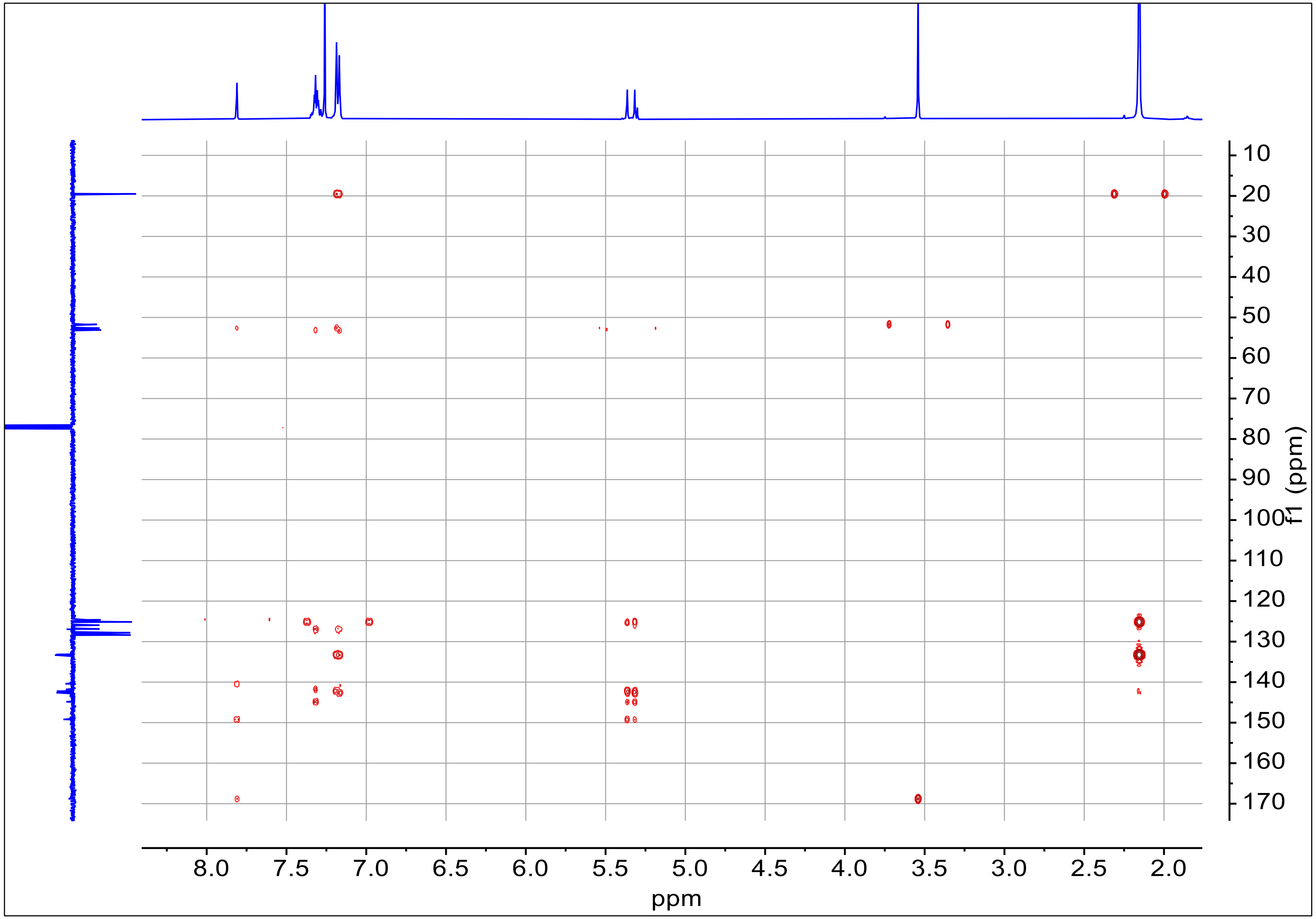

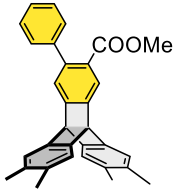

^{1}H NMR (400 MHz, $CDCl_3$): Compound **15**

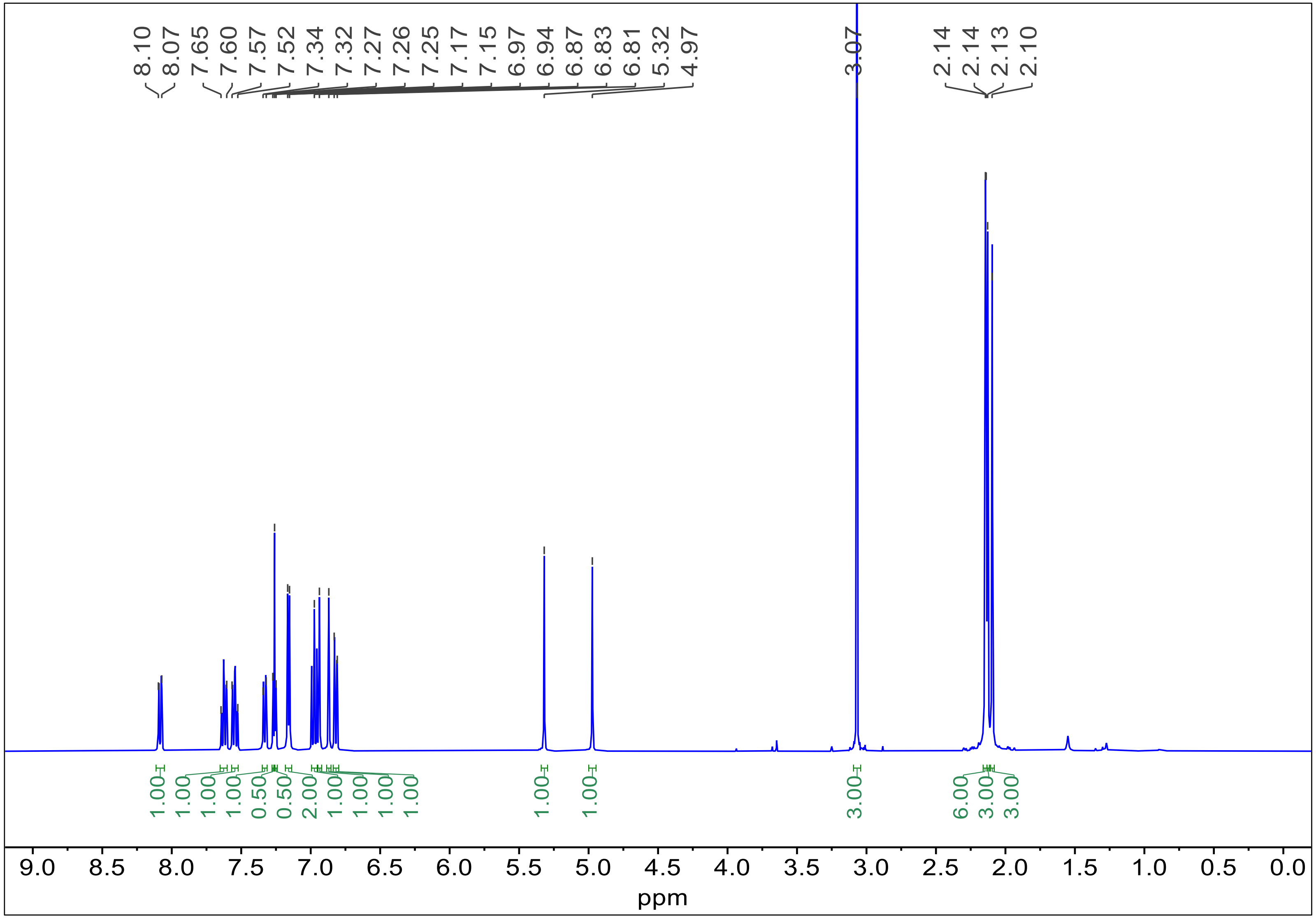

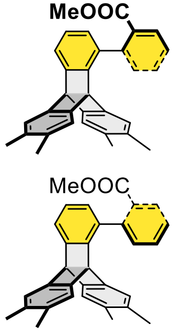

^{13}C {^{1}H} NMR (100 MHz, $CDCl_3$): Compound **15**

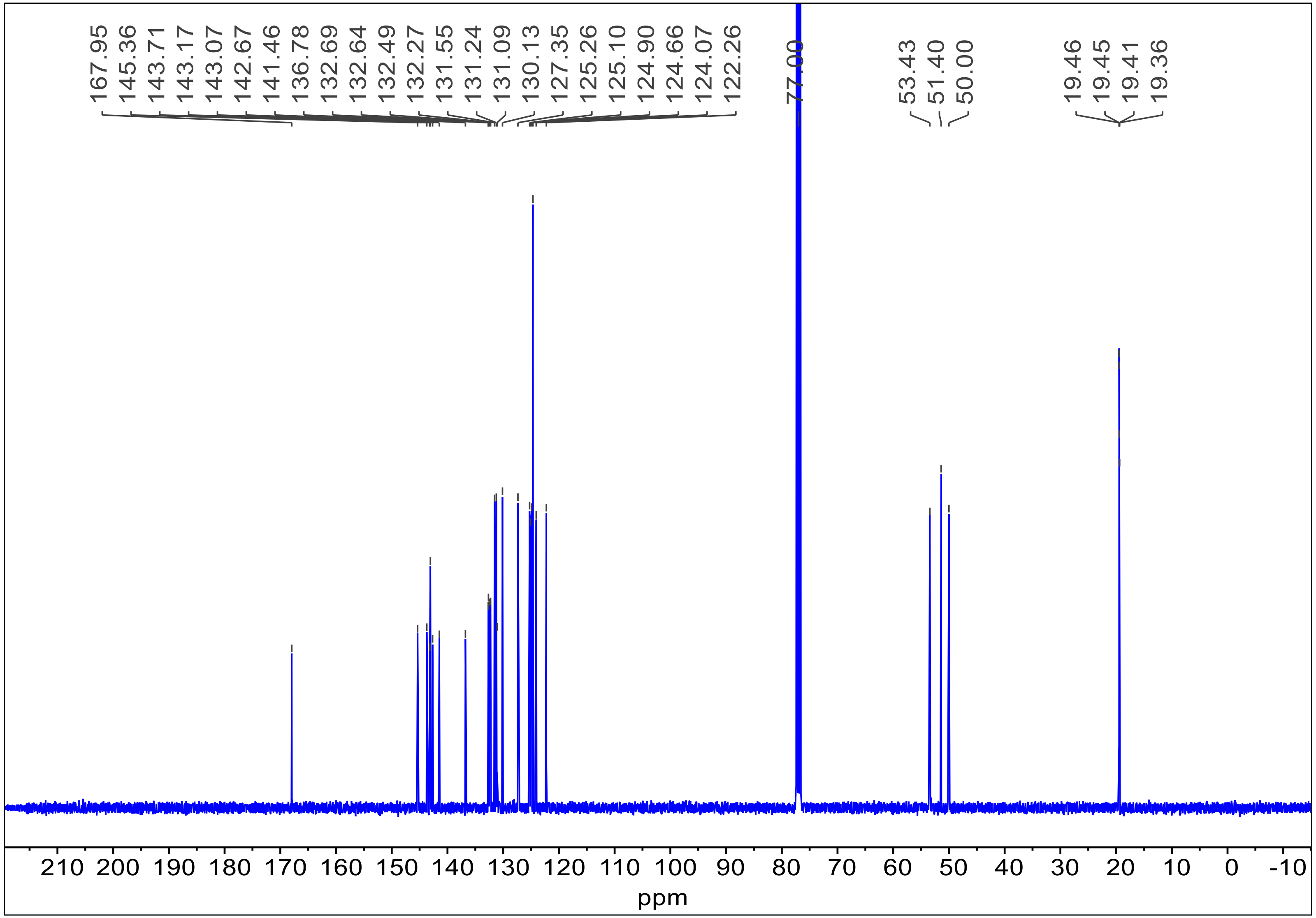

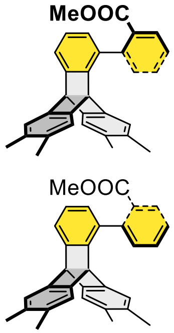

$^{13}C\ \{^1H\}$ APT NMR (100 MHz, $CDCl_3$): Compound **15**

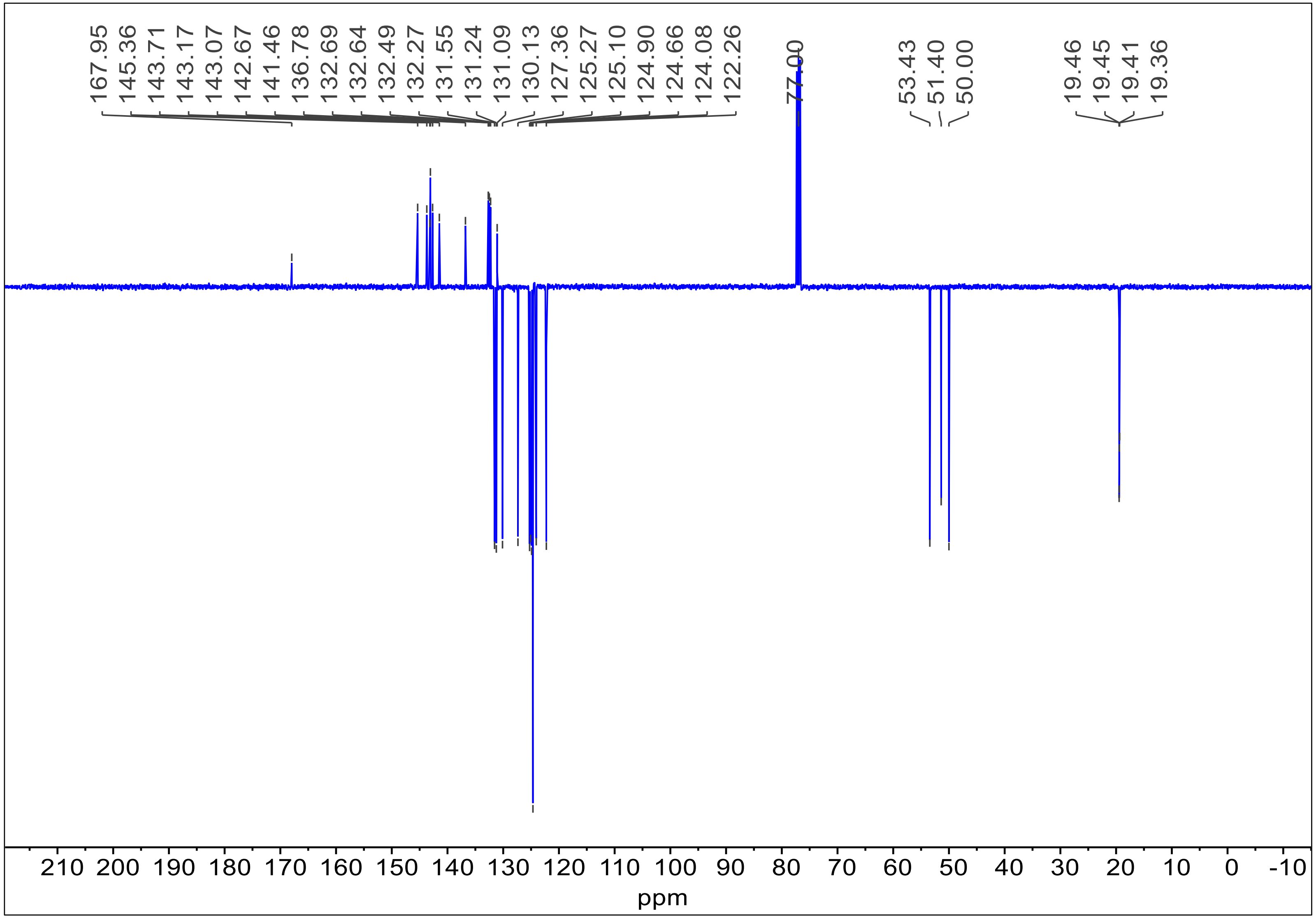

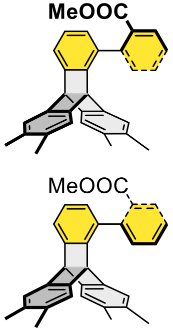

$^{1}H – ^{1}H$ COSY ($CDCl_3$): Compound **15**

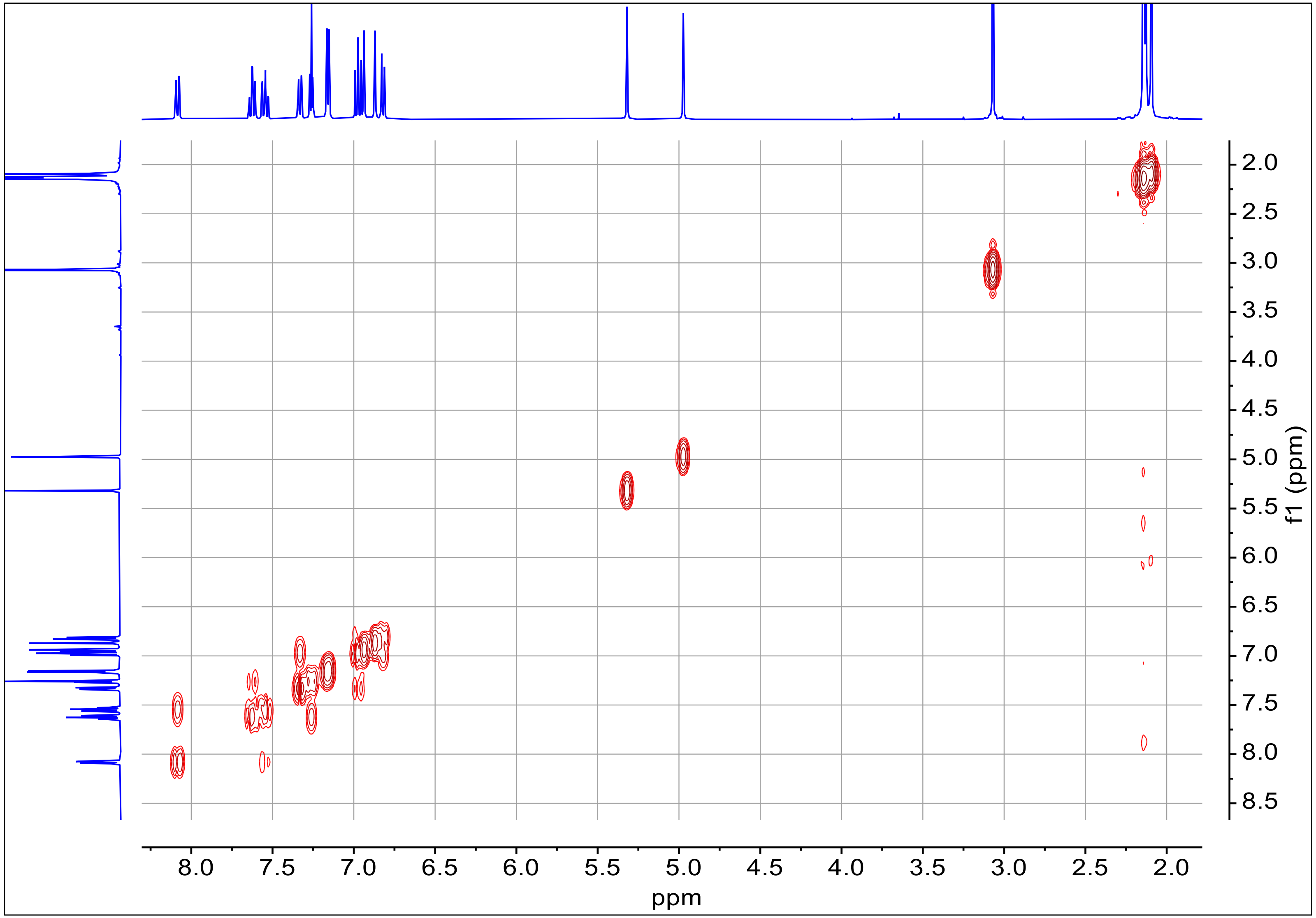

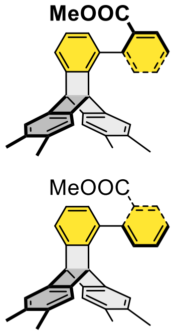

HSQC (CDCl3): Compound **15**

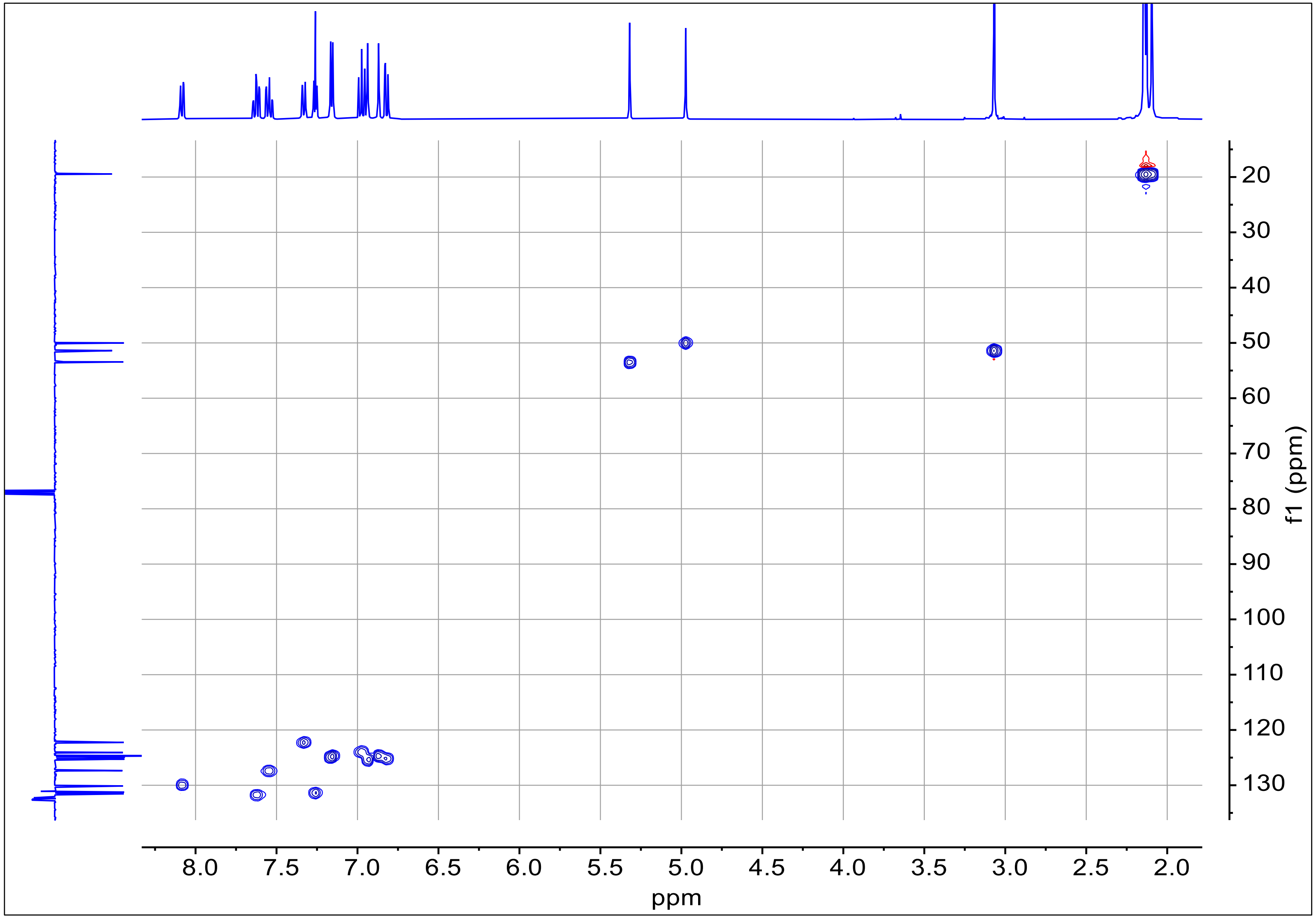

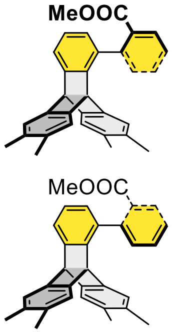

HMBC ($CDCl_3$): Compound **15**

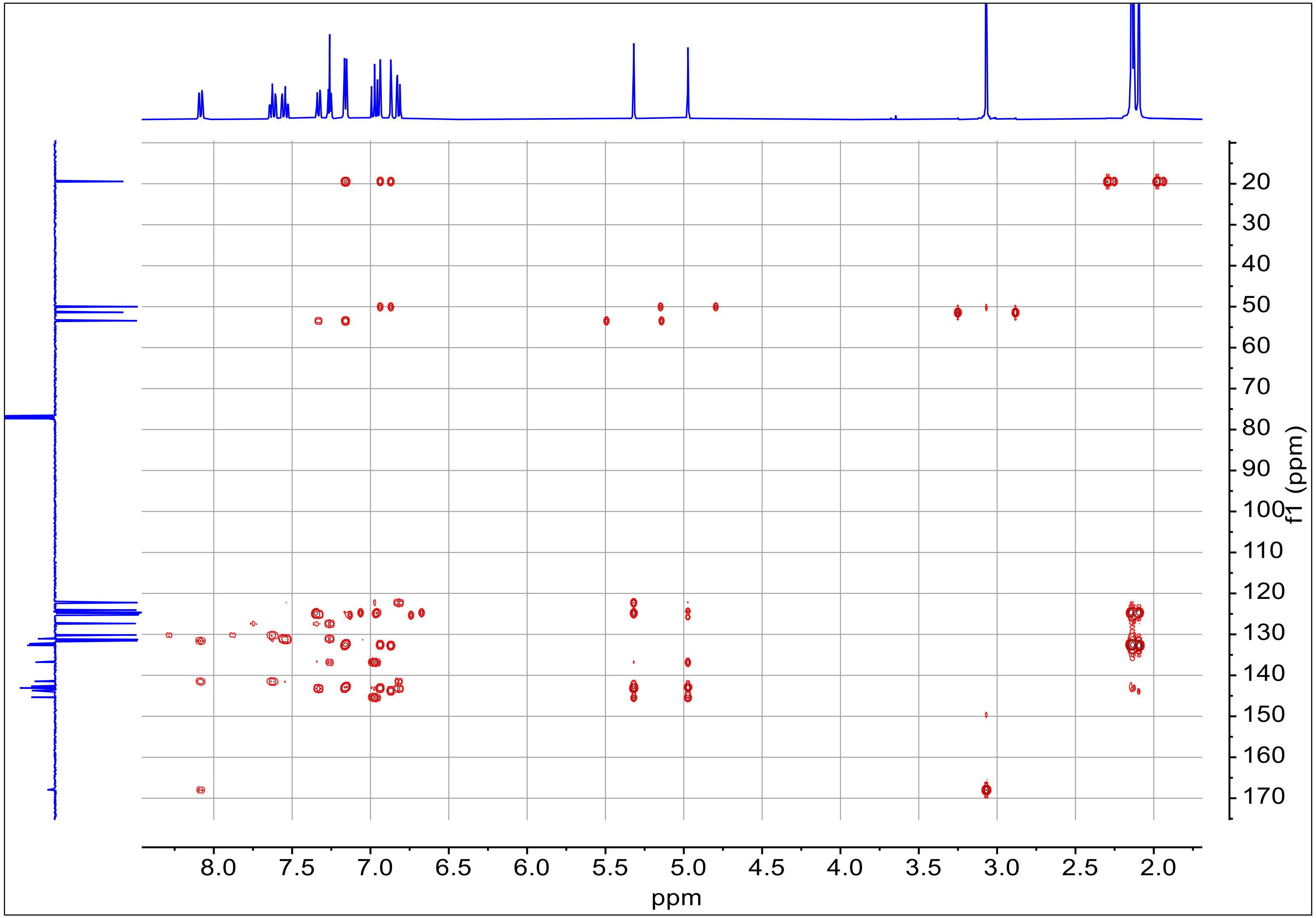

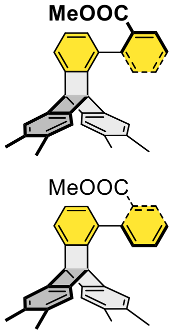

^{1}H NMR (400 MHz, CDCl$_3$): Compound **16**

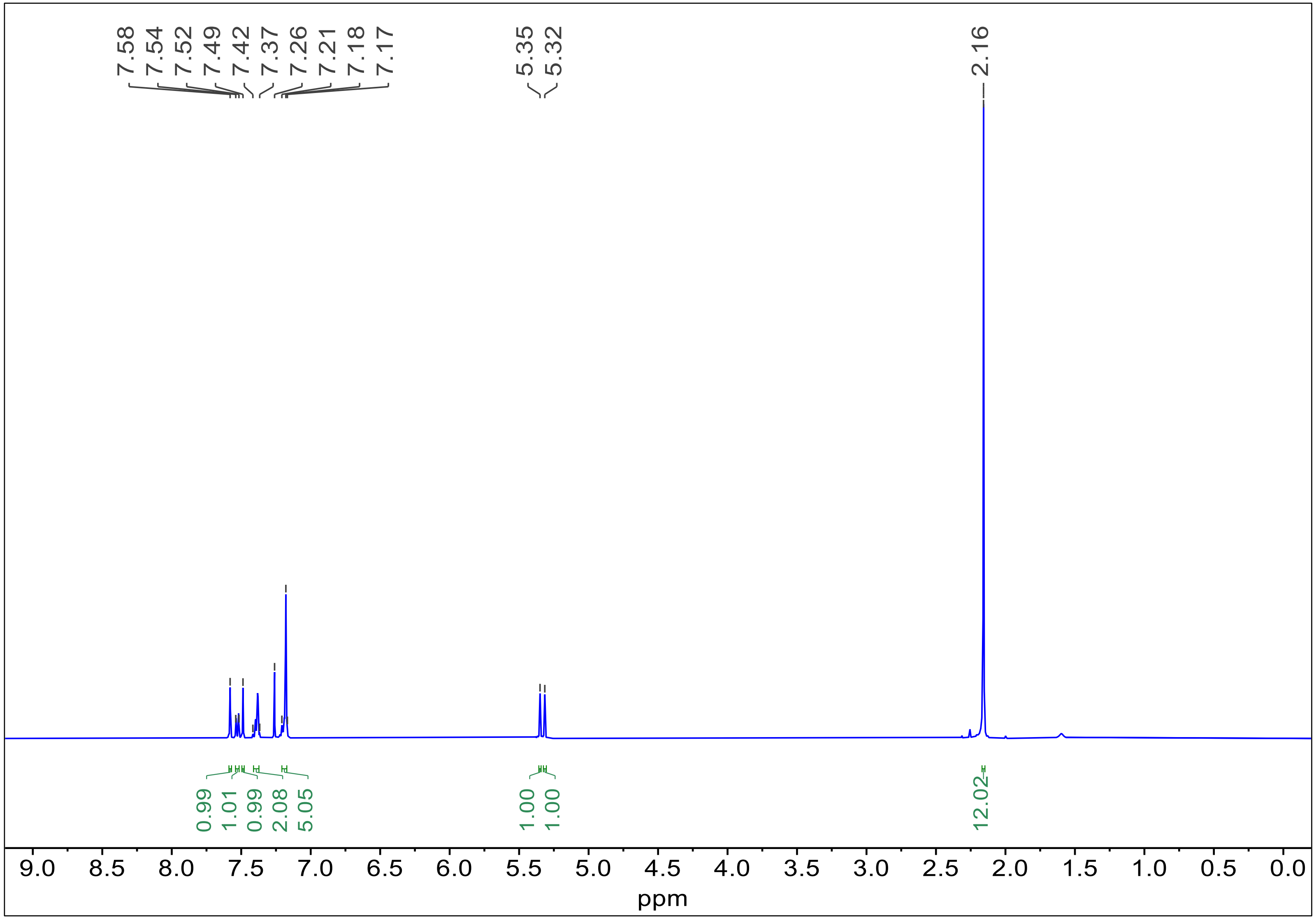

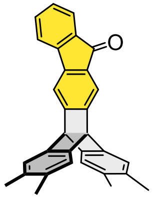

^{13}C {^{1}H} NMR (100 MHz, $CDCl_3$): Compound **16**

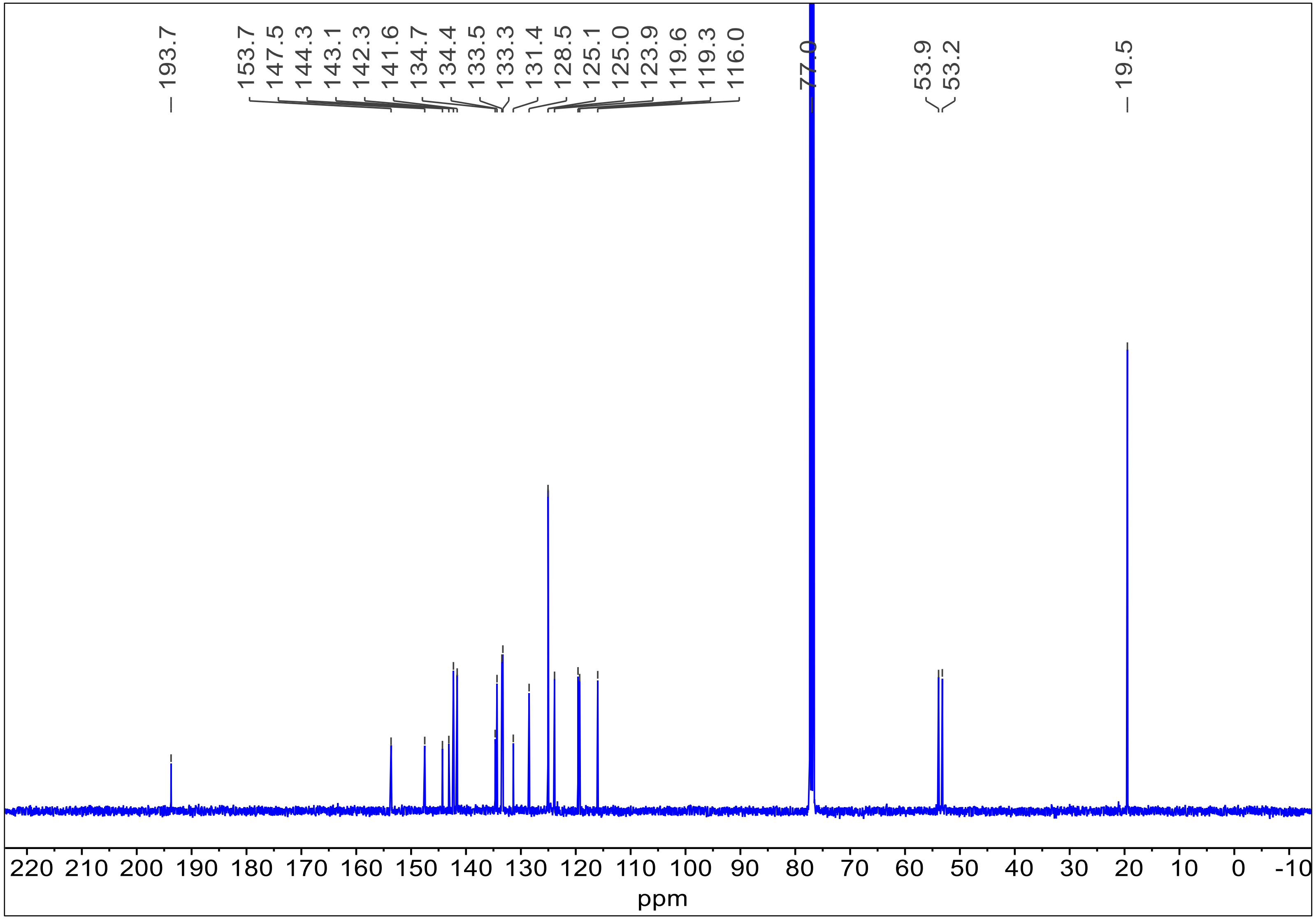

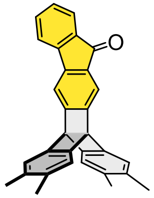

$^{13}C\ \{^{1}H\}$ APT NMR (100 MHz, $CDCl_3$): Compound **16**

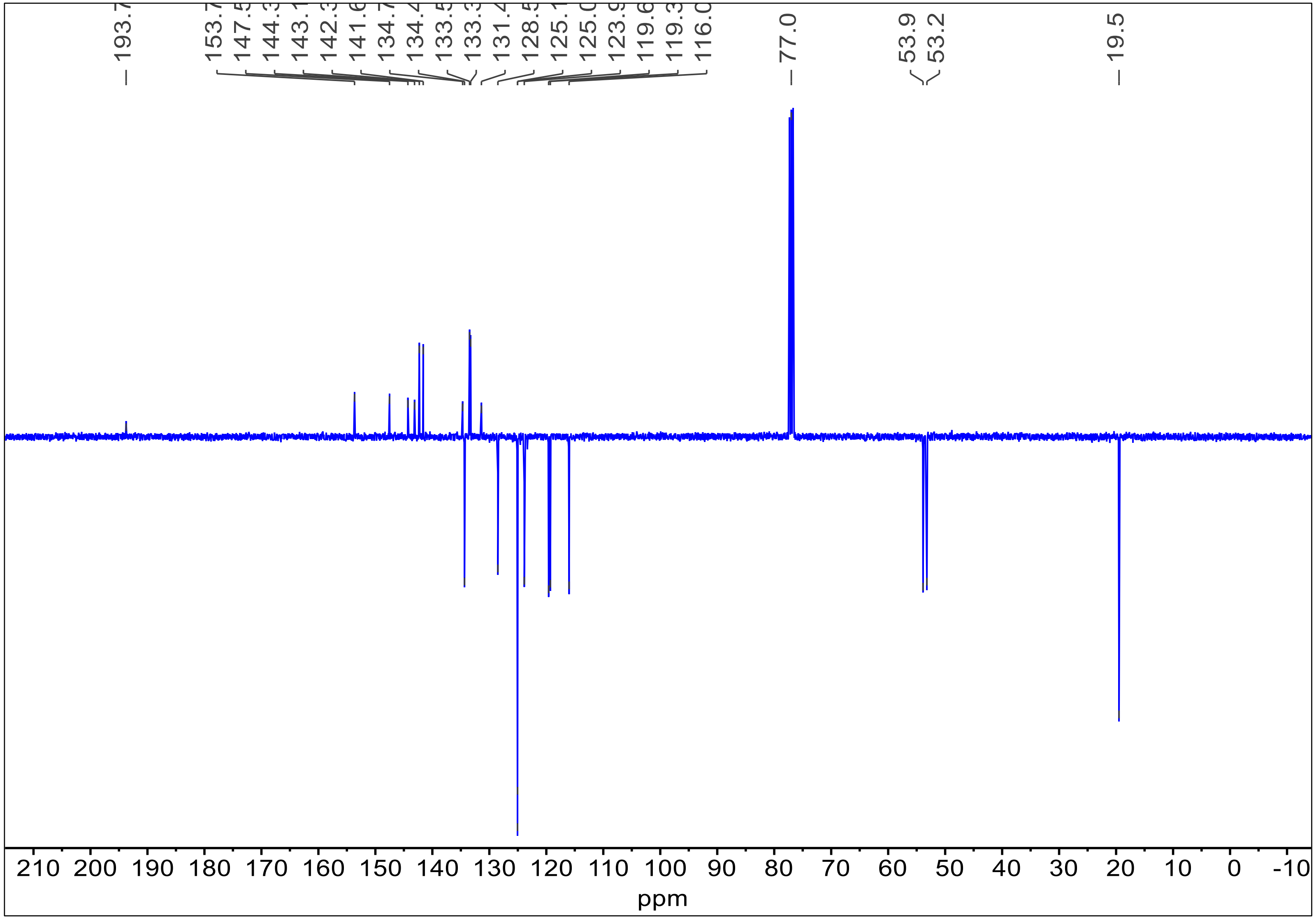

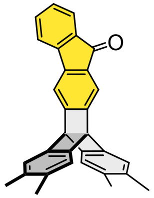

$^{1}H - {}^{1}H$ COSY ($CDCl_3$): Compound **16**

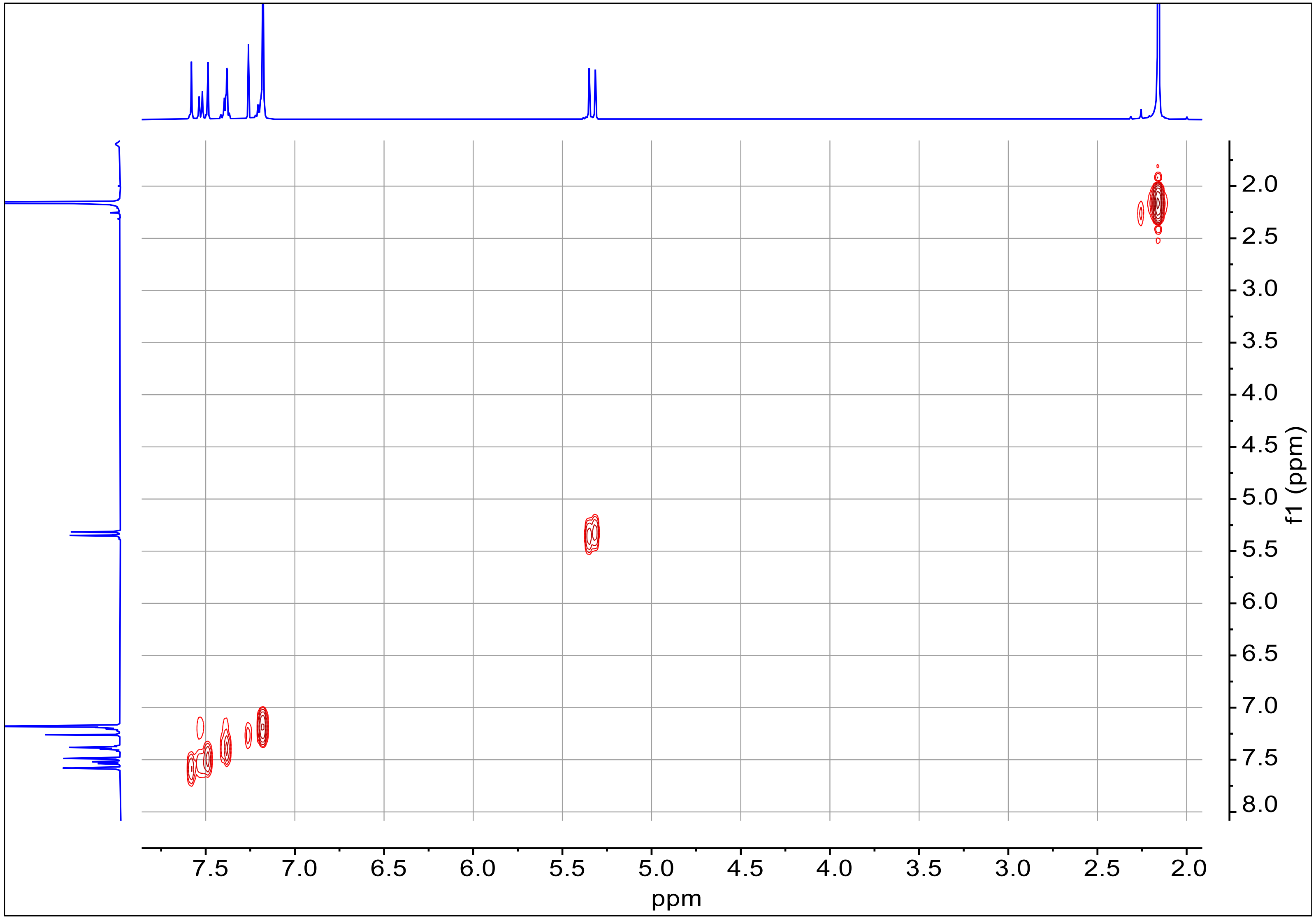

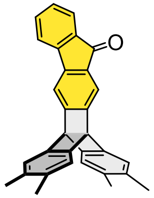

HSQC ($CDCl_3$): Compound **16**

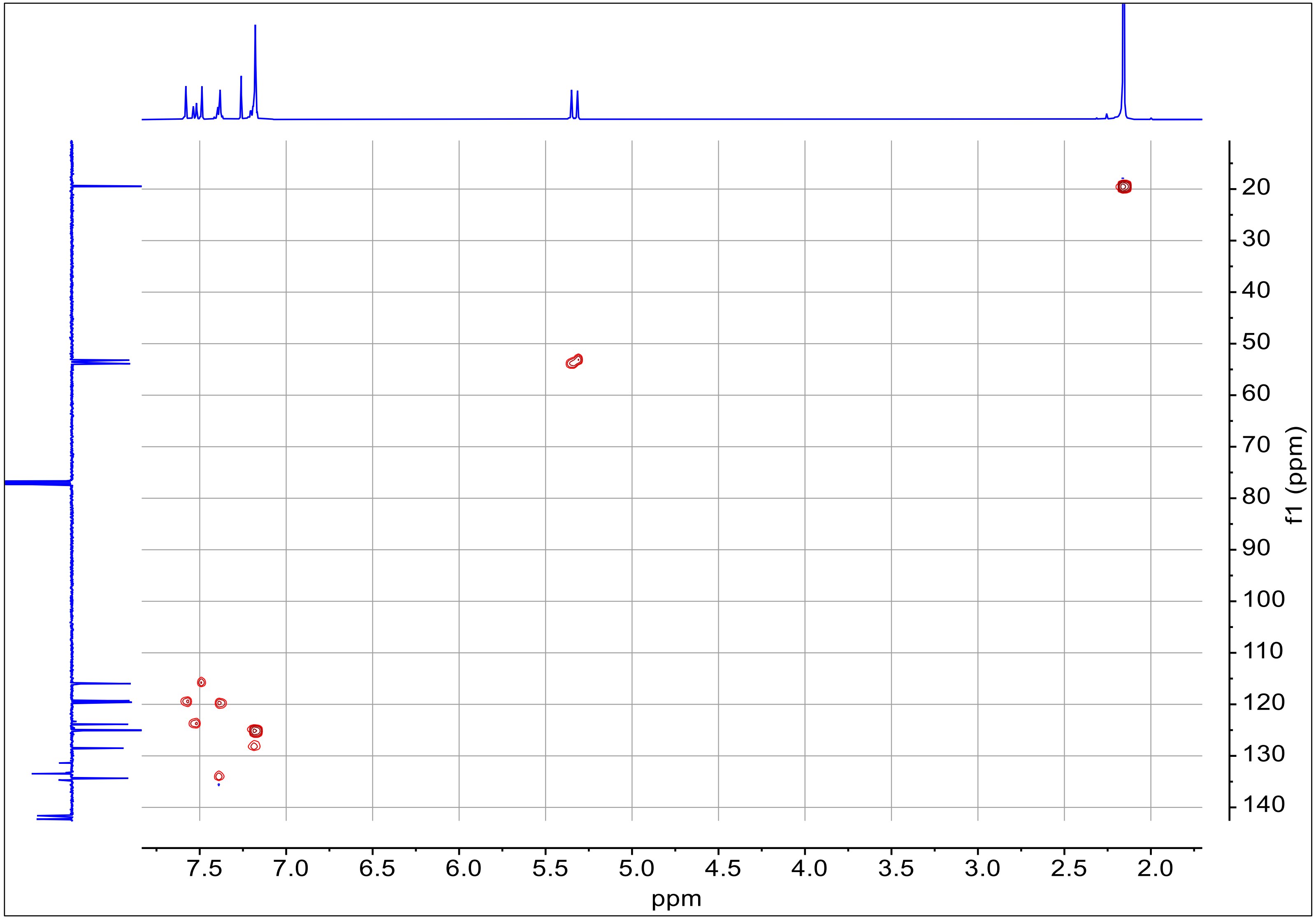

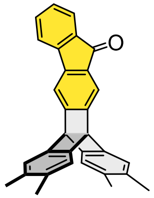

HMBC ($CDCl_3$): Compound **16**

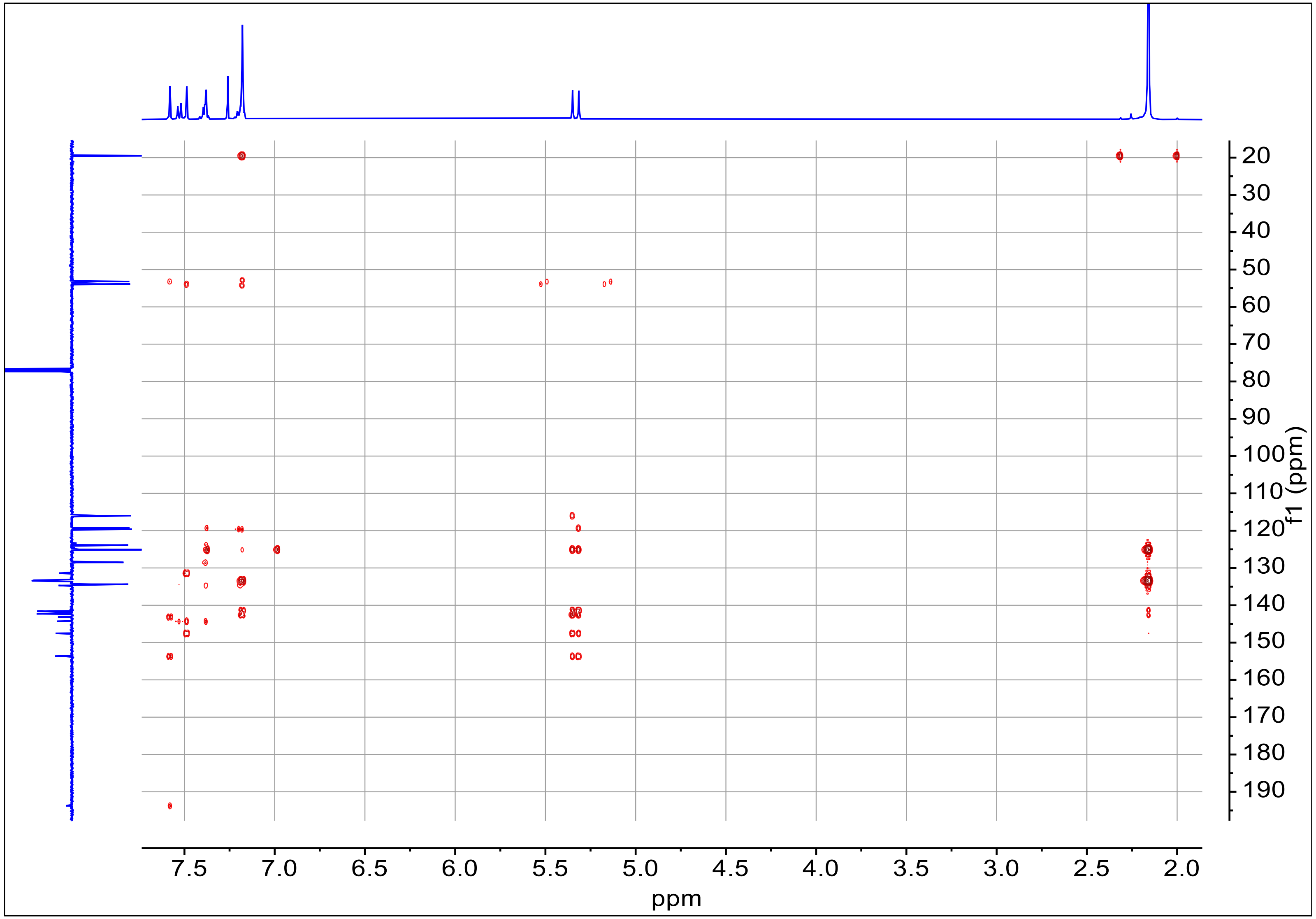

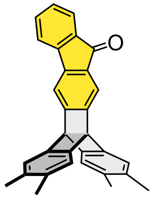

^1H NMR (400 MHz, $CDCl_3$): Compound **17**

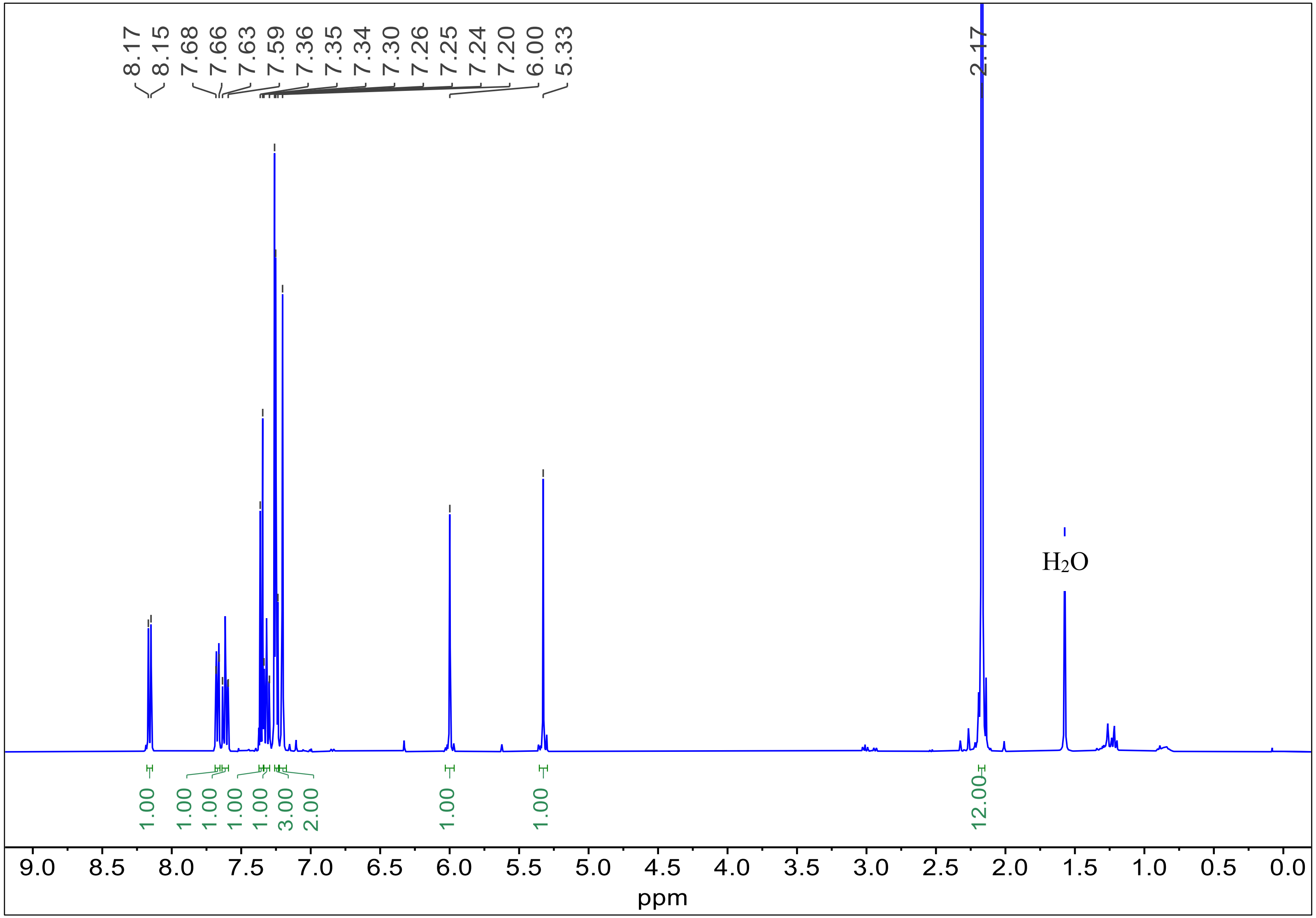

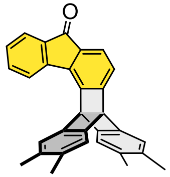

$^{13}C\ \{^{1}H\}$ NMR (100 MHz, $CDCl_3$): Compound **17**

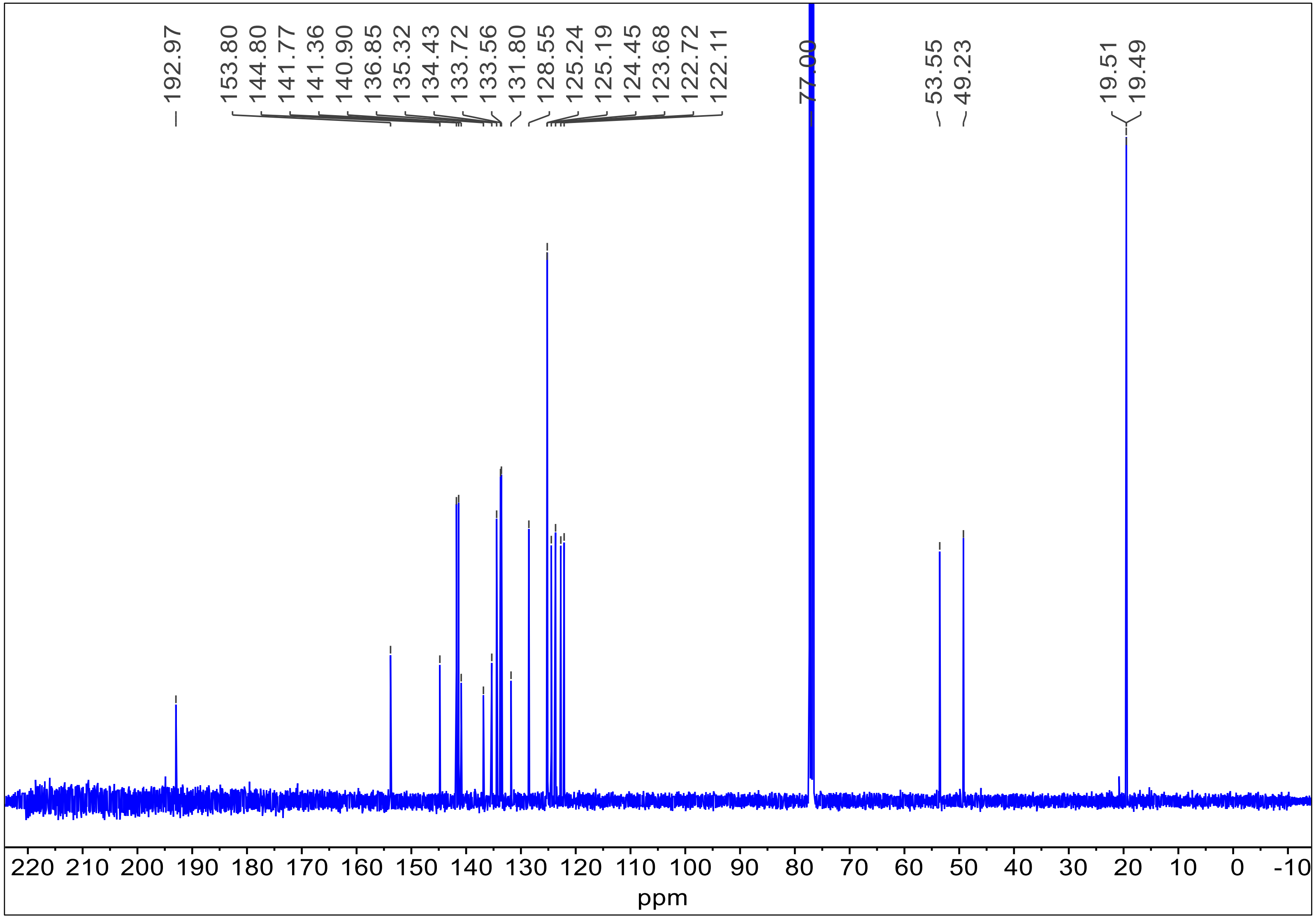

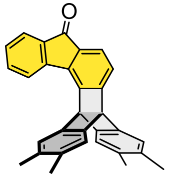

$^{13}C\ \{^1H\}$ APT NMR (100 MHz, $CDCl_3$): Compound **17**

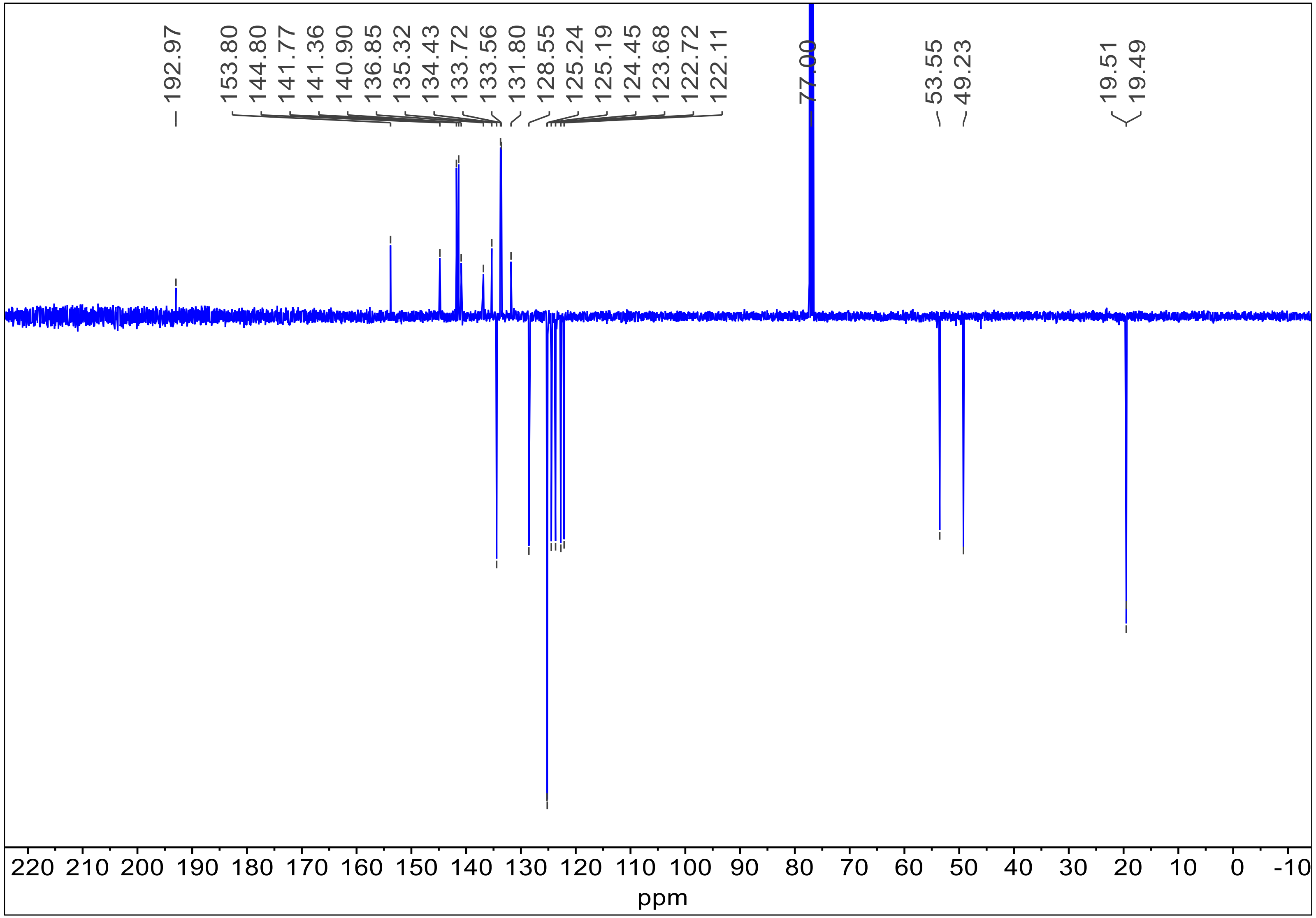

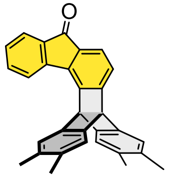

$^1H - ^1H$ COSY ($CDCl_3$): Compound **17**

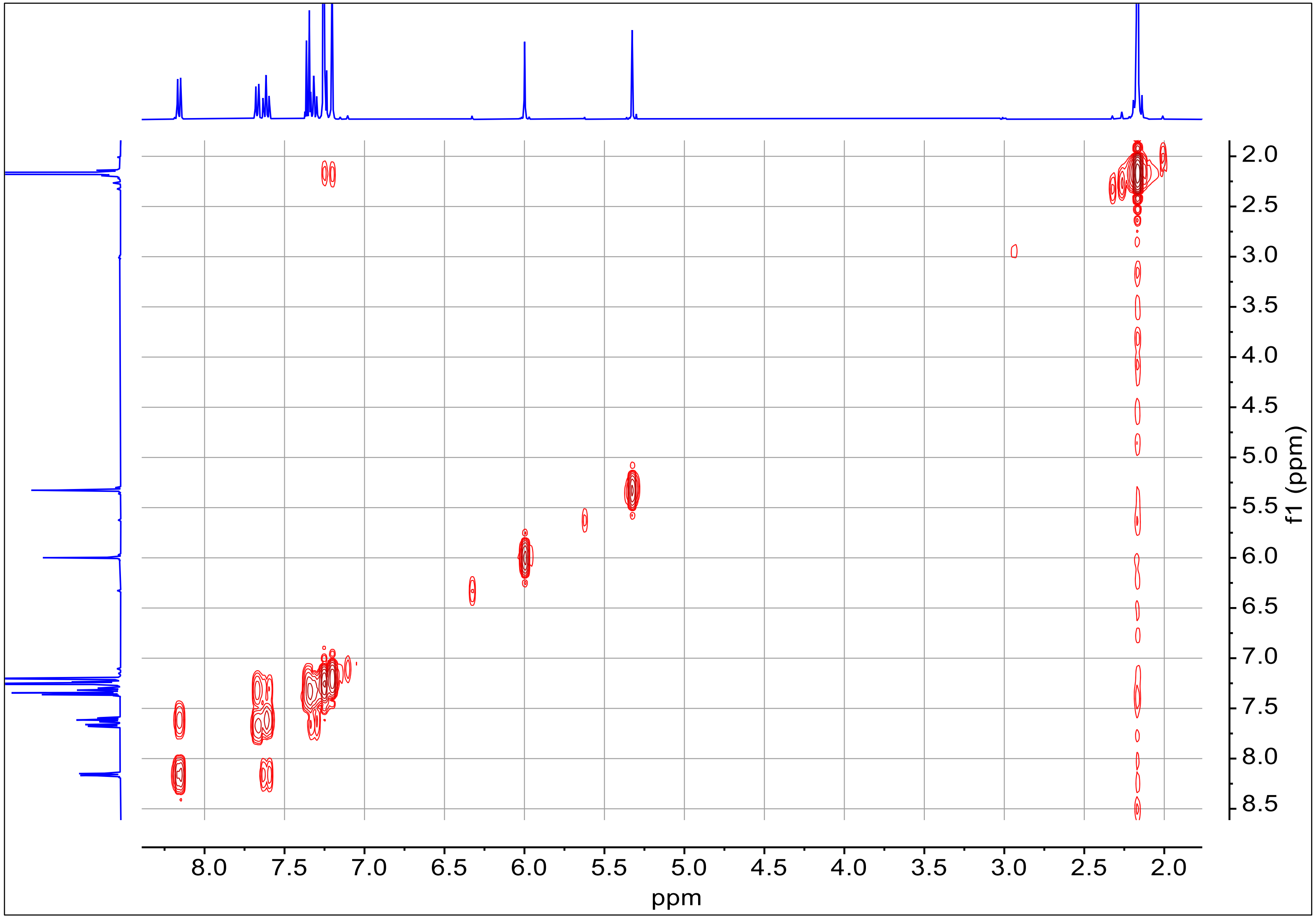

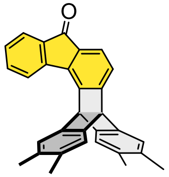

HSQC ($CDCl_3$): Compound **17**

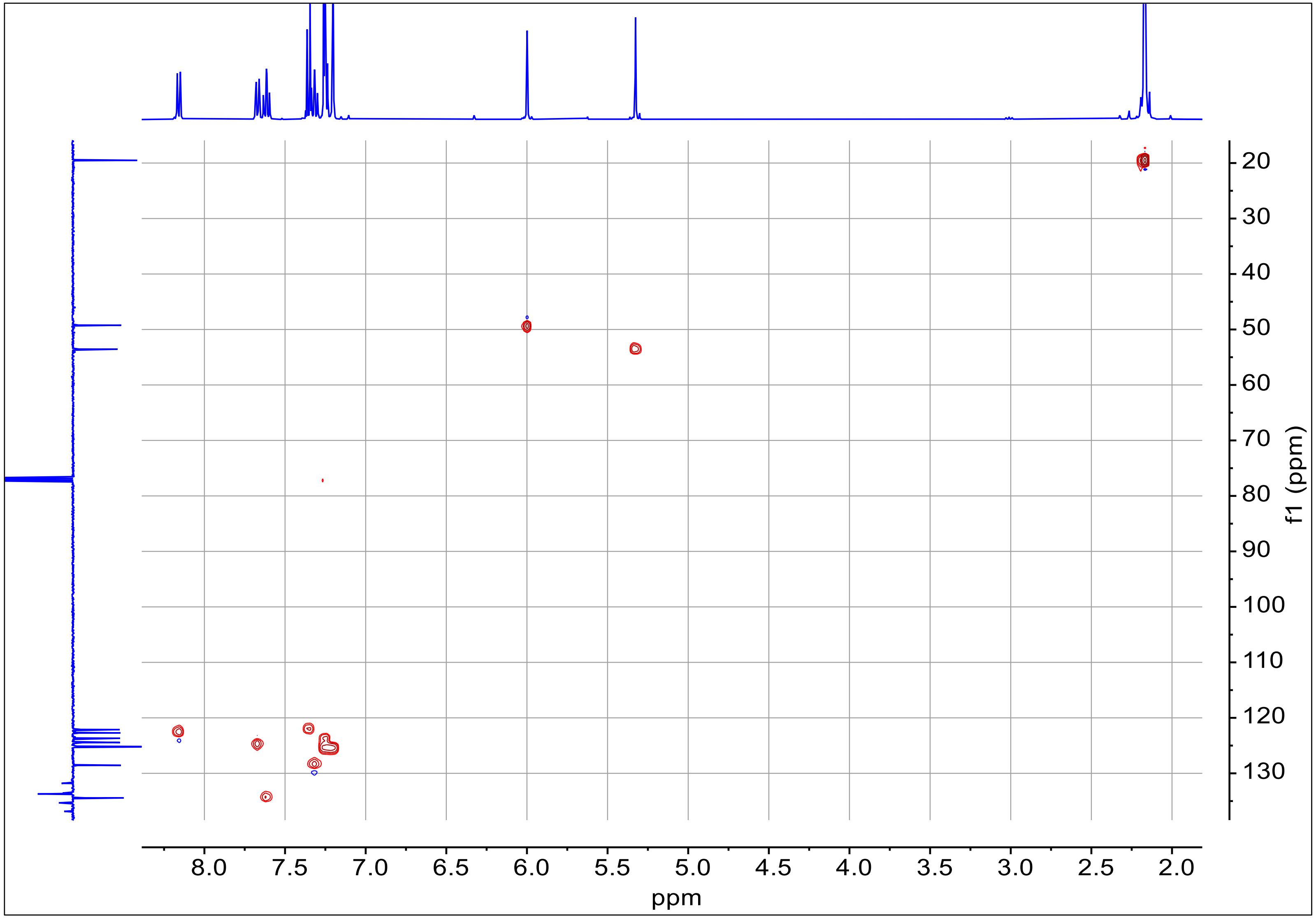

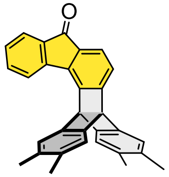

HMBC (CDCl₃): Compound **17**

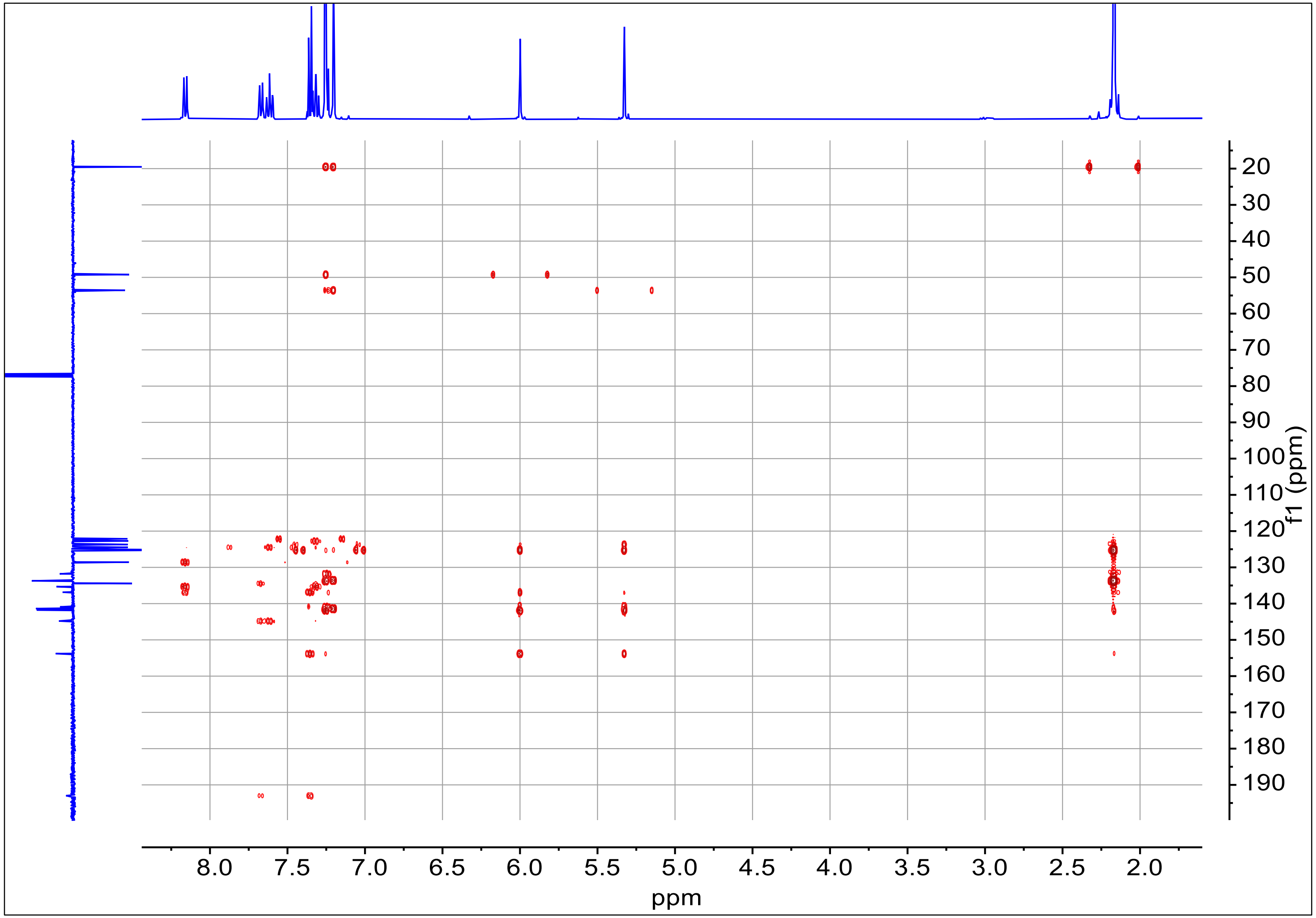

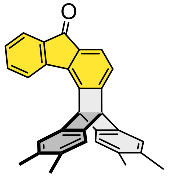

^{1}H NMR (400 MHz, $CDCl_3$): Compound **18**

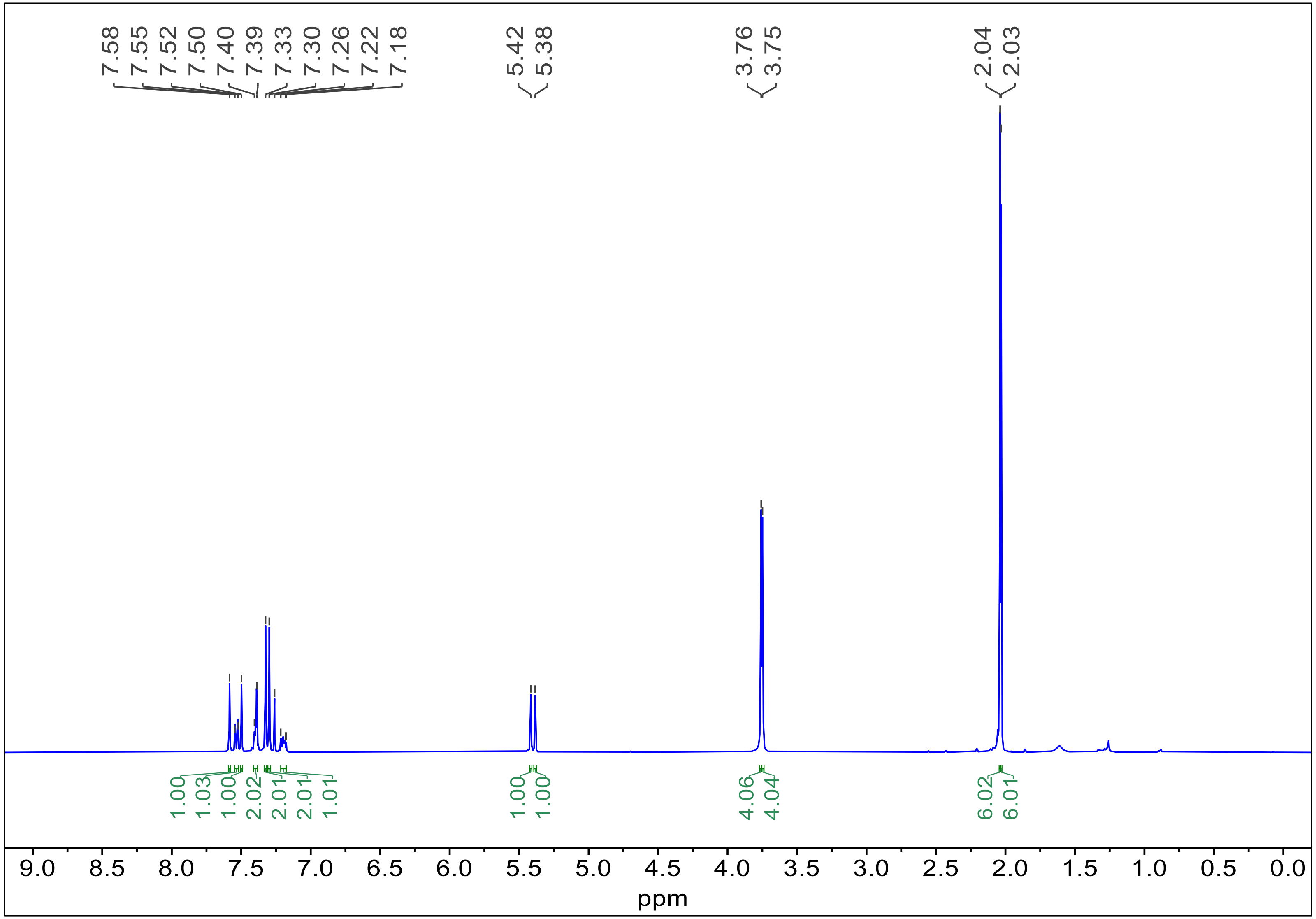

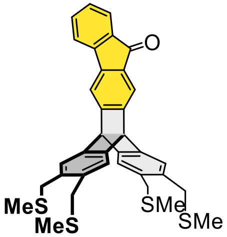

$^{13}C\ \{^{1}H\}$ APT NMR (100 MHz, $CDCl_3$): Compound **18**

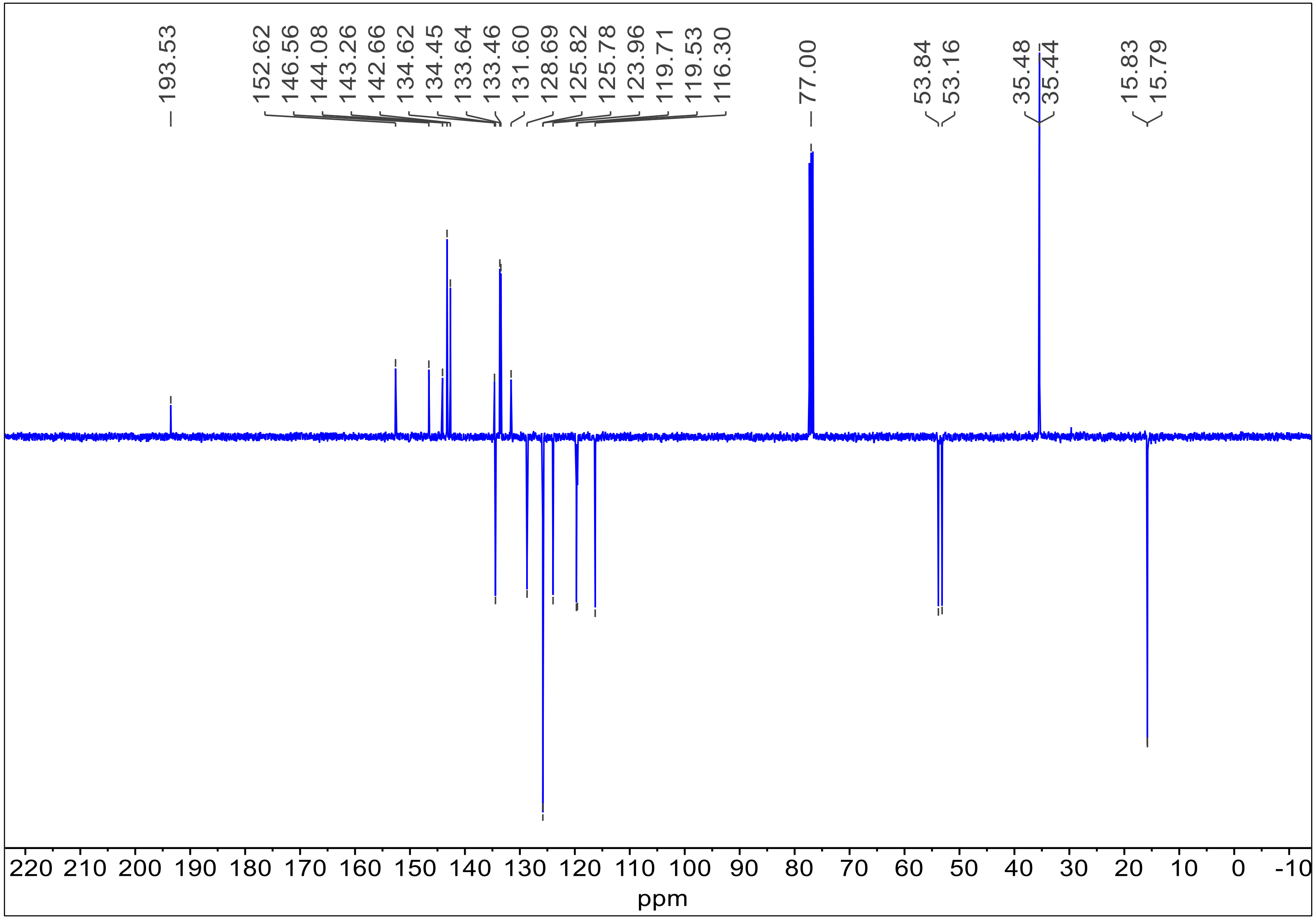

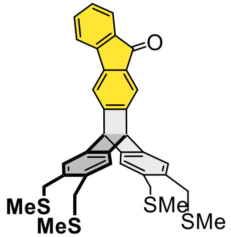

^1H – ^1H COSY ($CDCl_3$): Compound **18**

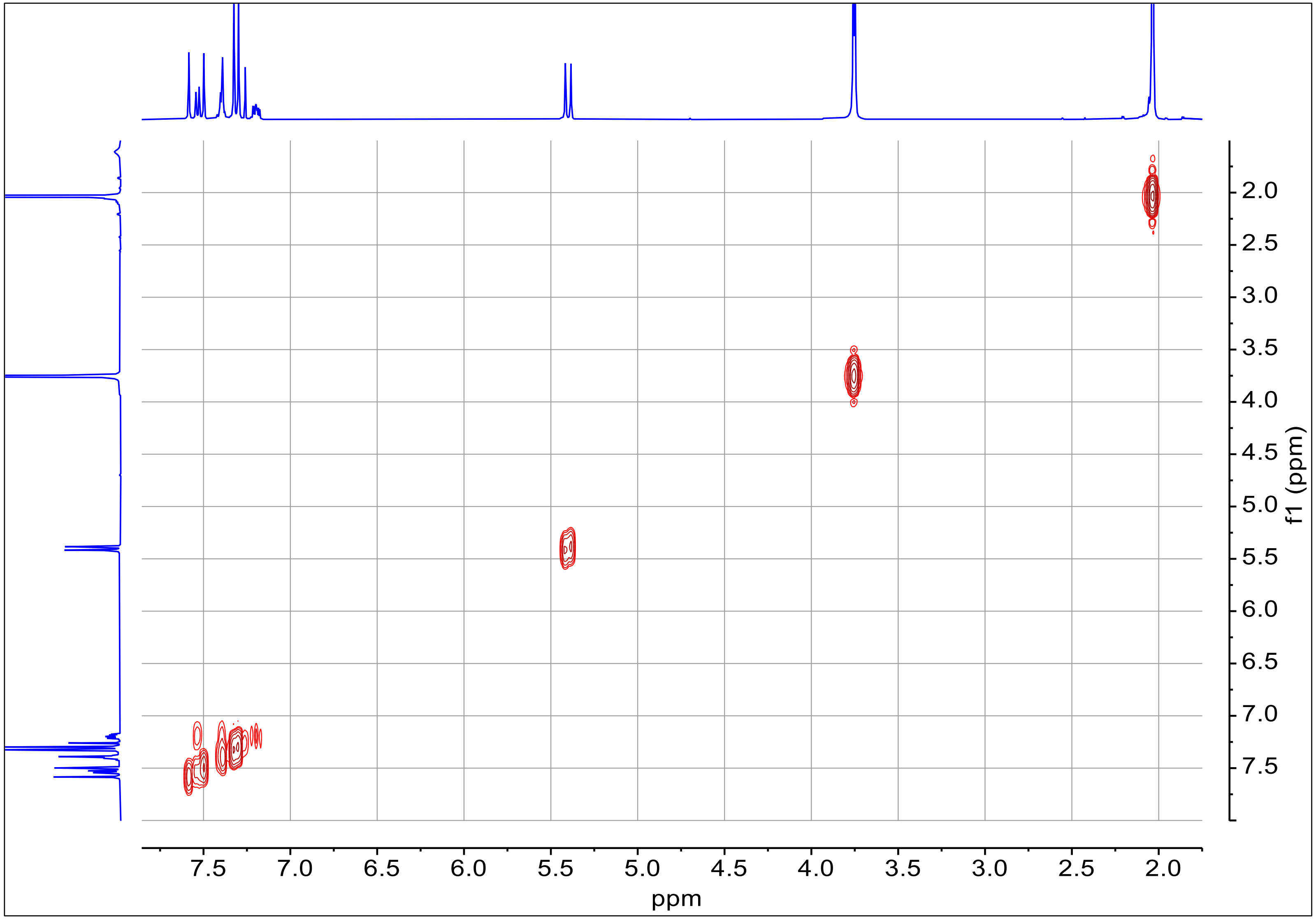

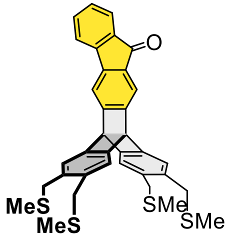

HSQC ($CDCl_3$): Compound **18**

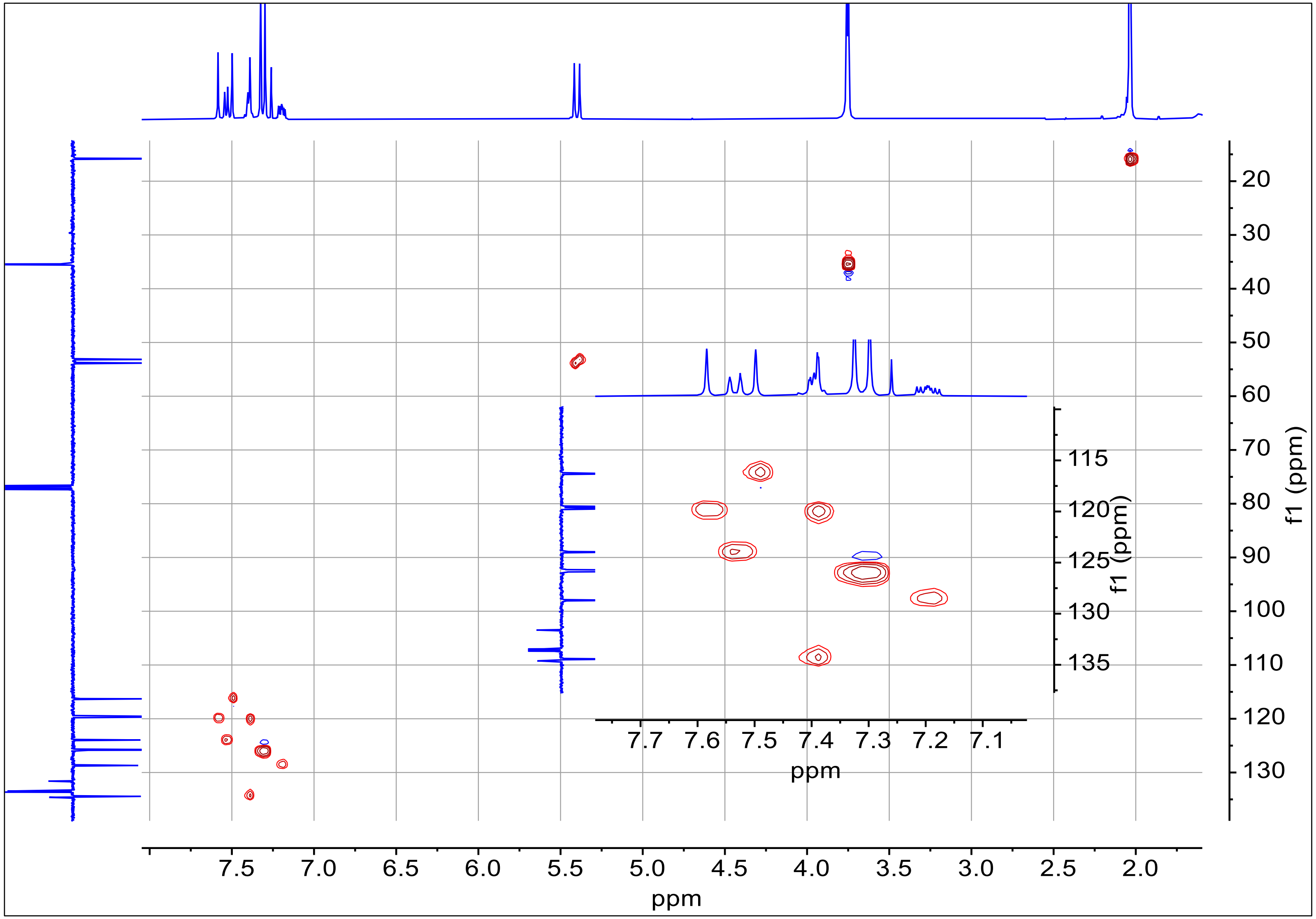

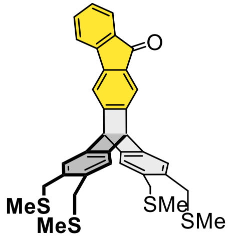

HMBC ($CDCl_3$): Compound **18**

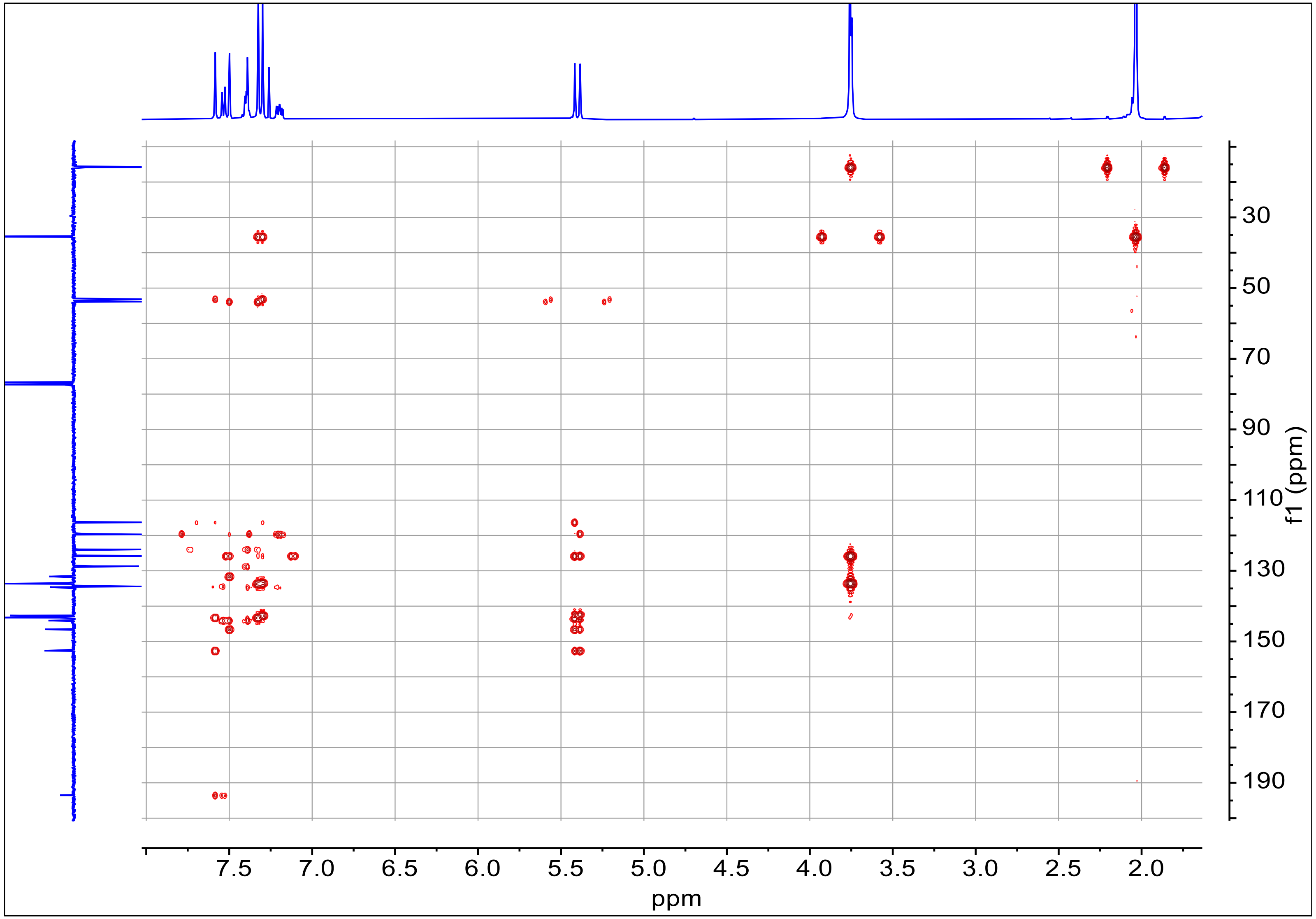

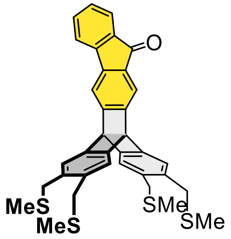

^{1}H NMR (400 MHz, $CDCl_3$): Compound **19**

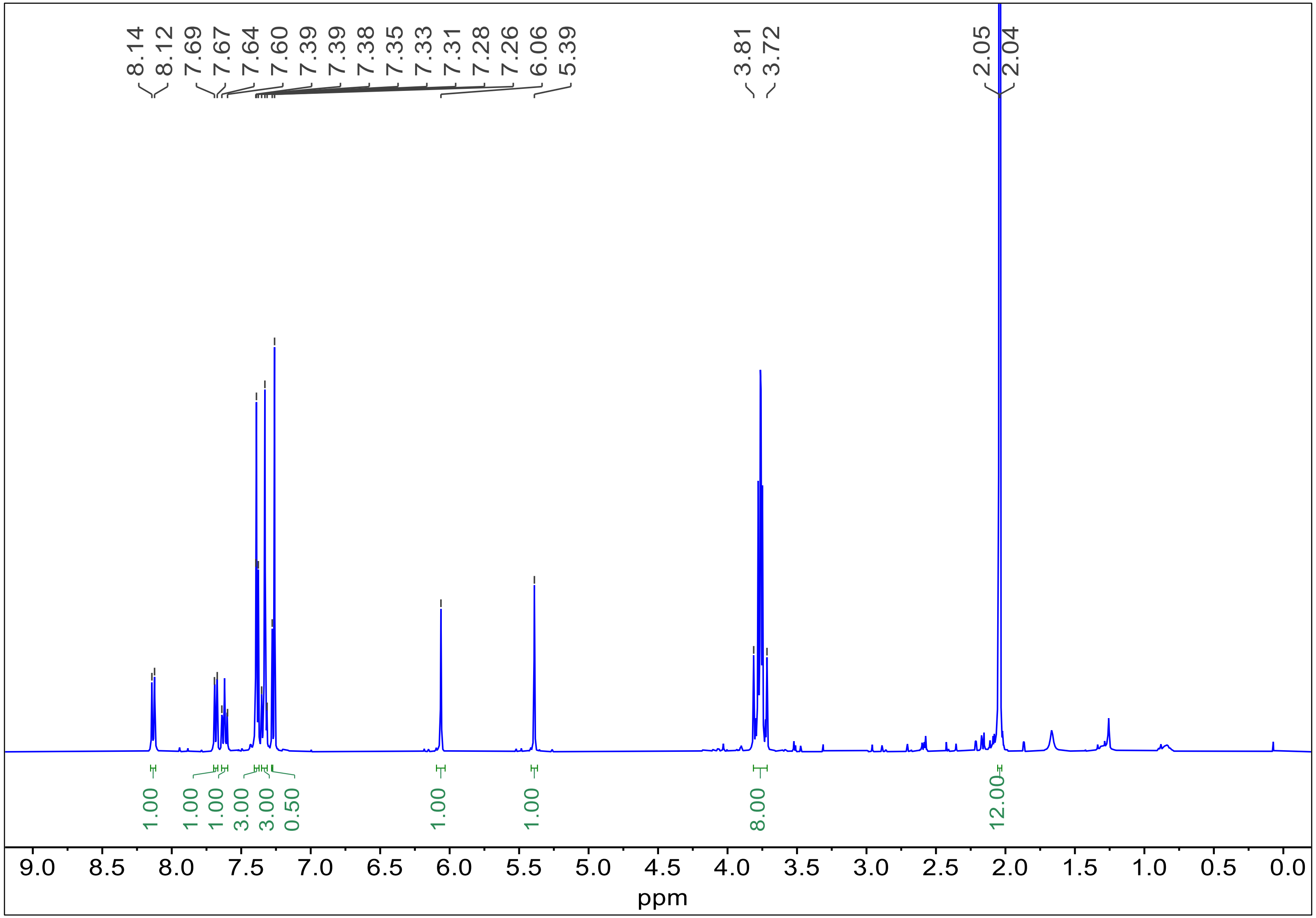

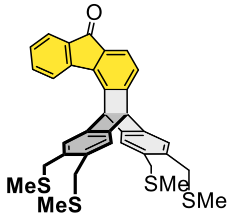

^{13}C {^{1}H} NMR (100 MHz, $CDCl_3$): Compound **19**

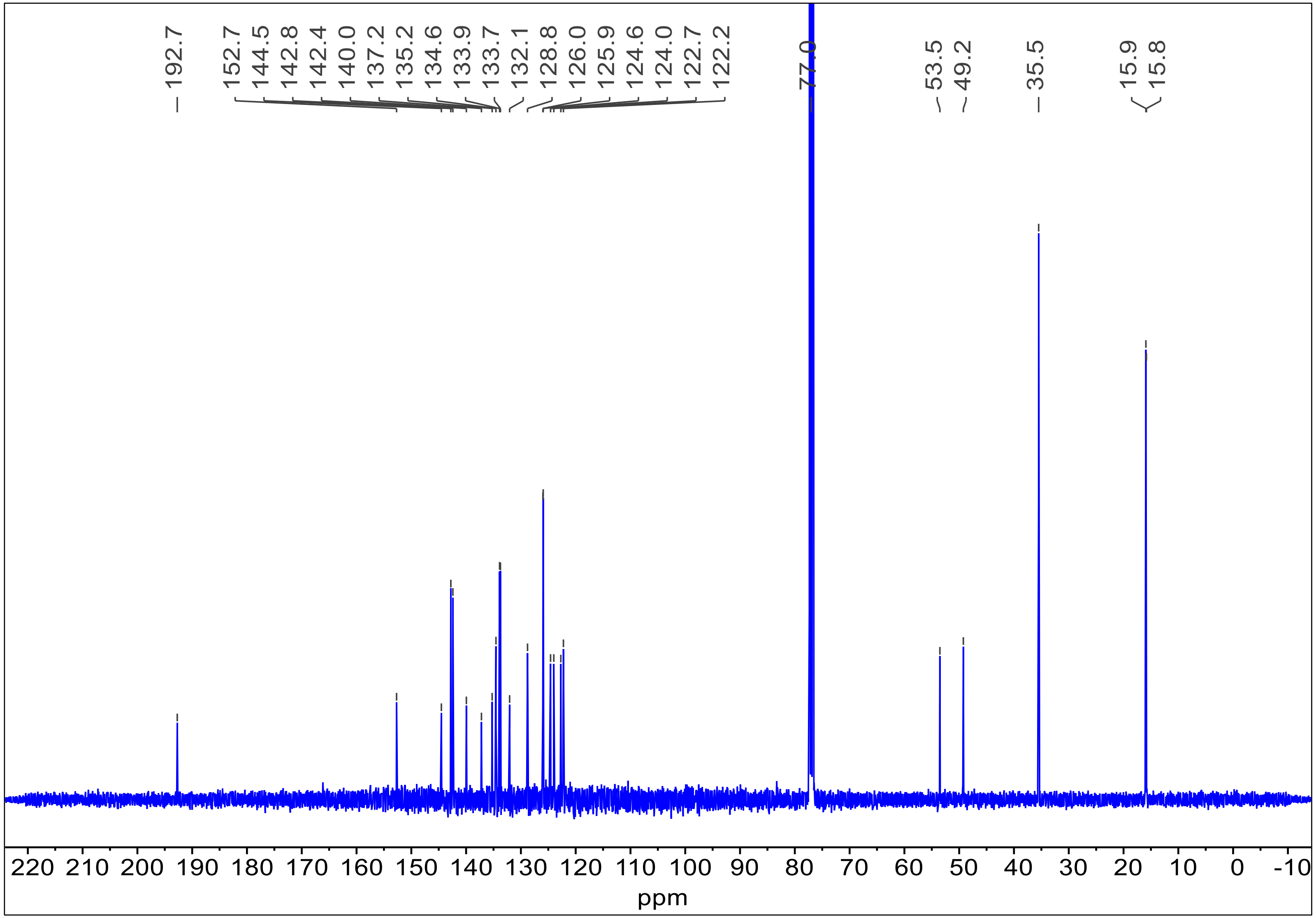

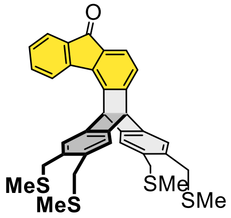

$^{13}C\ \{^{1}H\}$ APT NMR (100 MHz, $CDCl_3$): Compound **19**

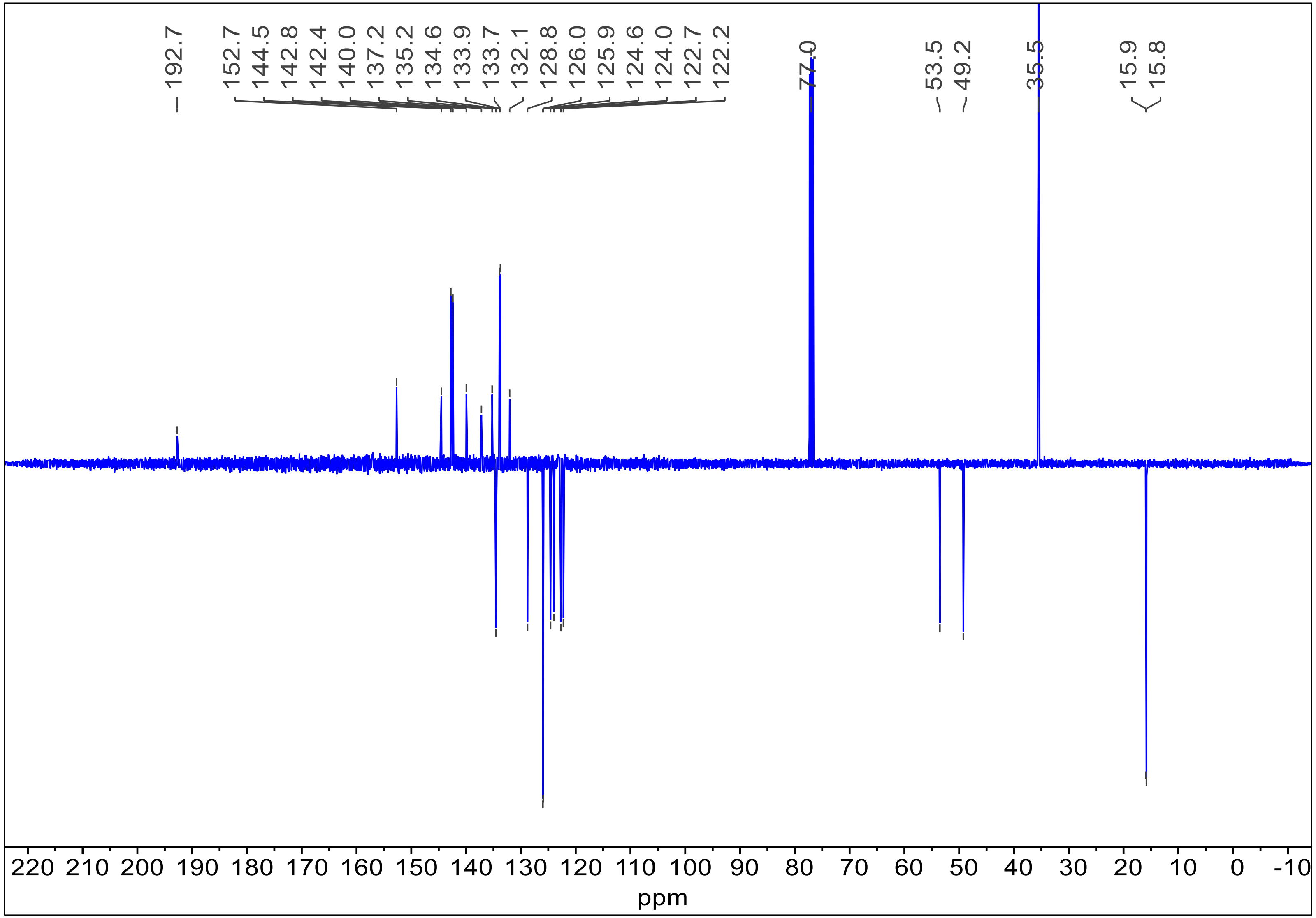

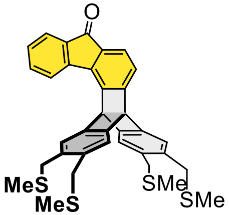

$^{1}H - ^{1}H$ COSY ($CDCl_3$): Compound **19**

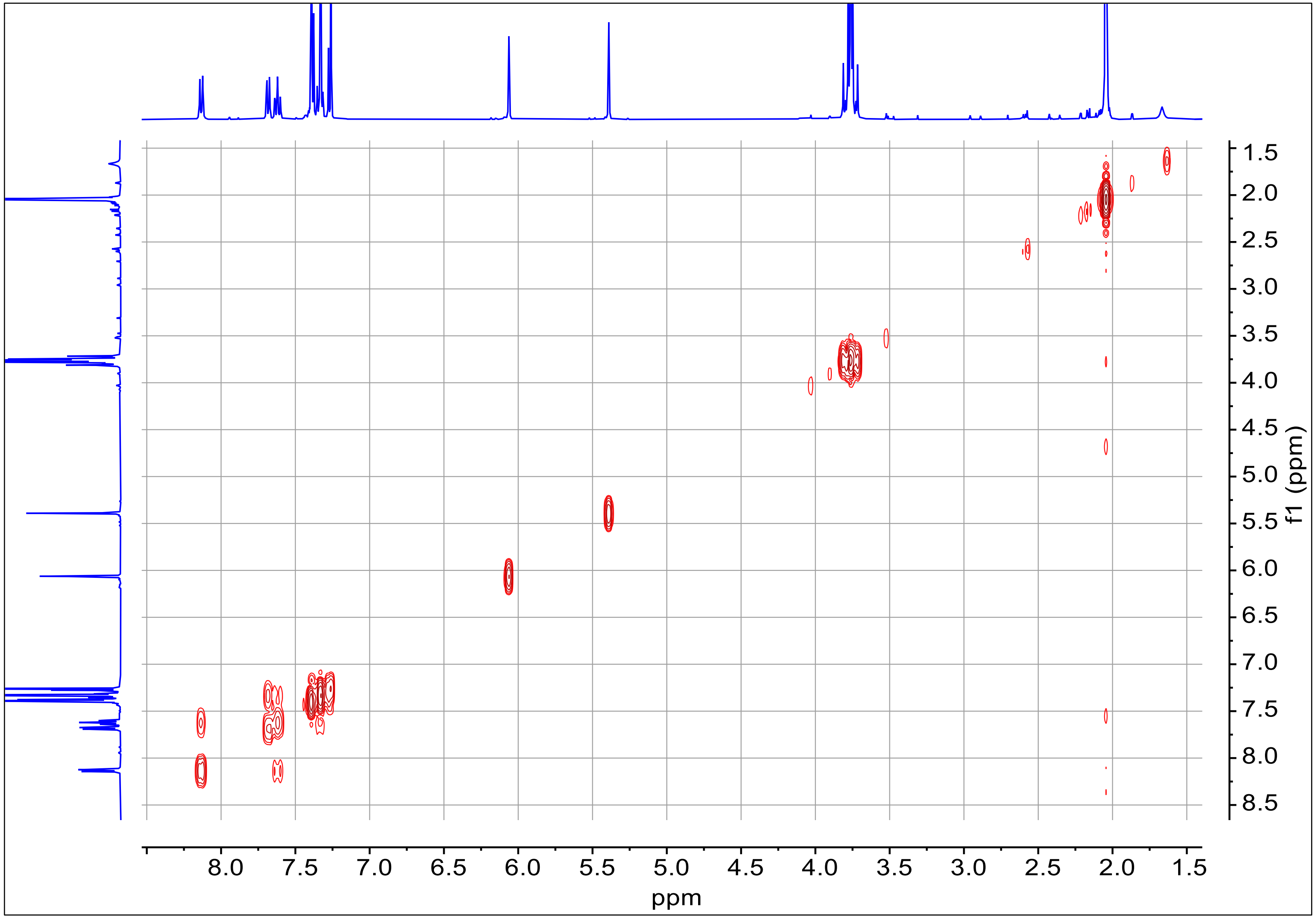

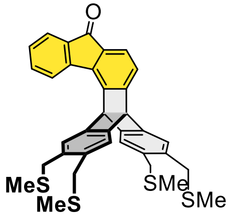

HSQC ($CDCl_3$): Compound **19**

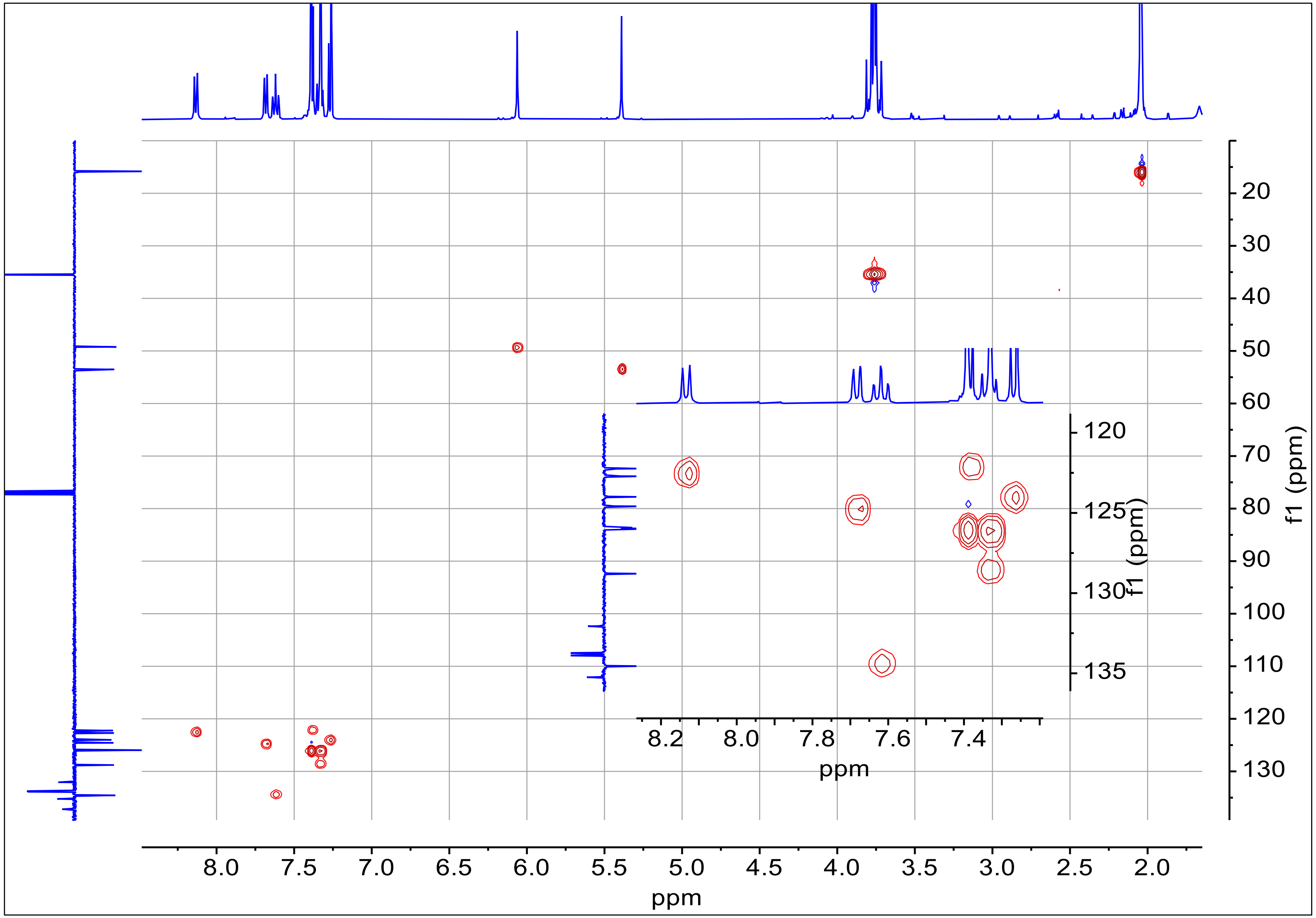

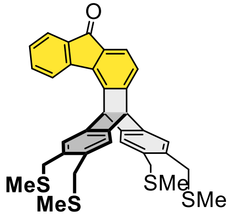

HMBC ($CDCl_3$): Compound **19**

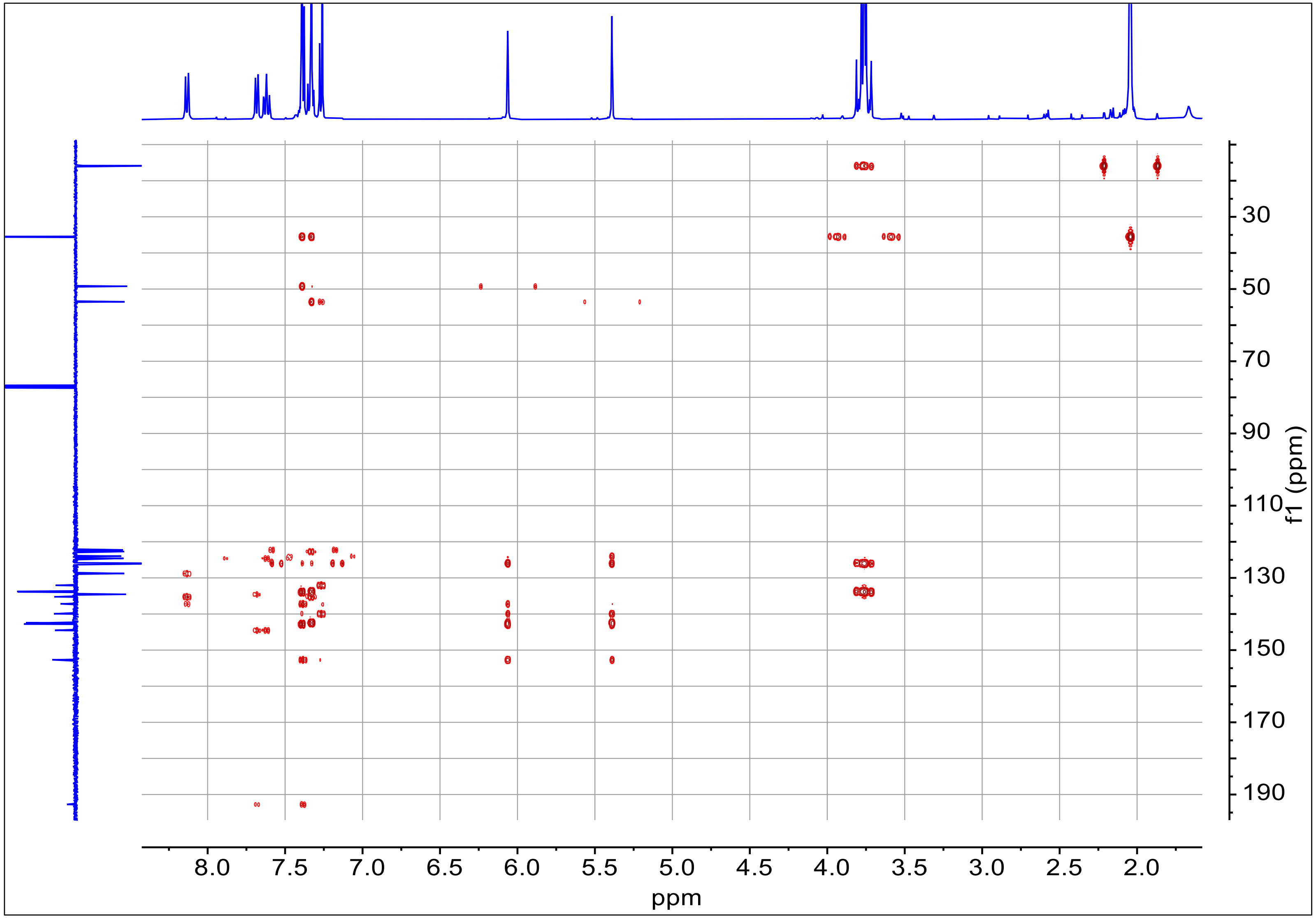

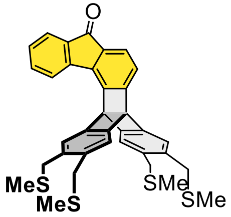

^{1}H NMR (400 MHz, $CDCl_3$): Compound **20** (crude, 1:1 ratio)

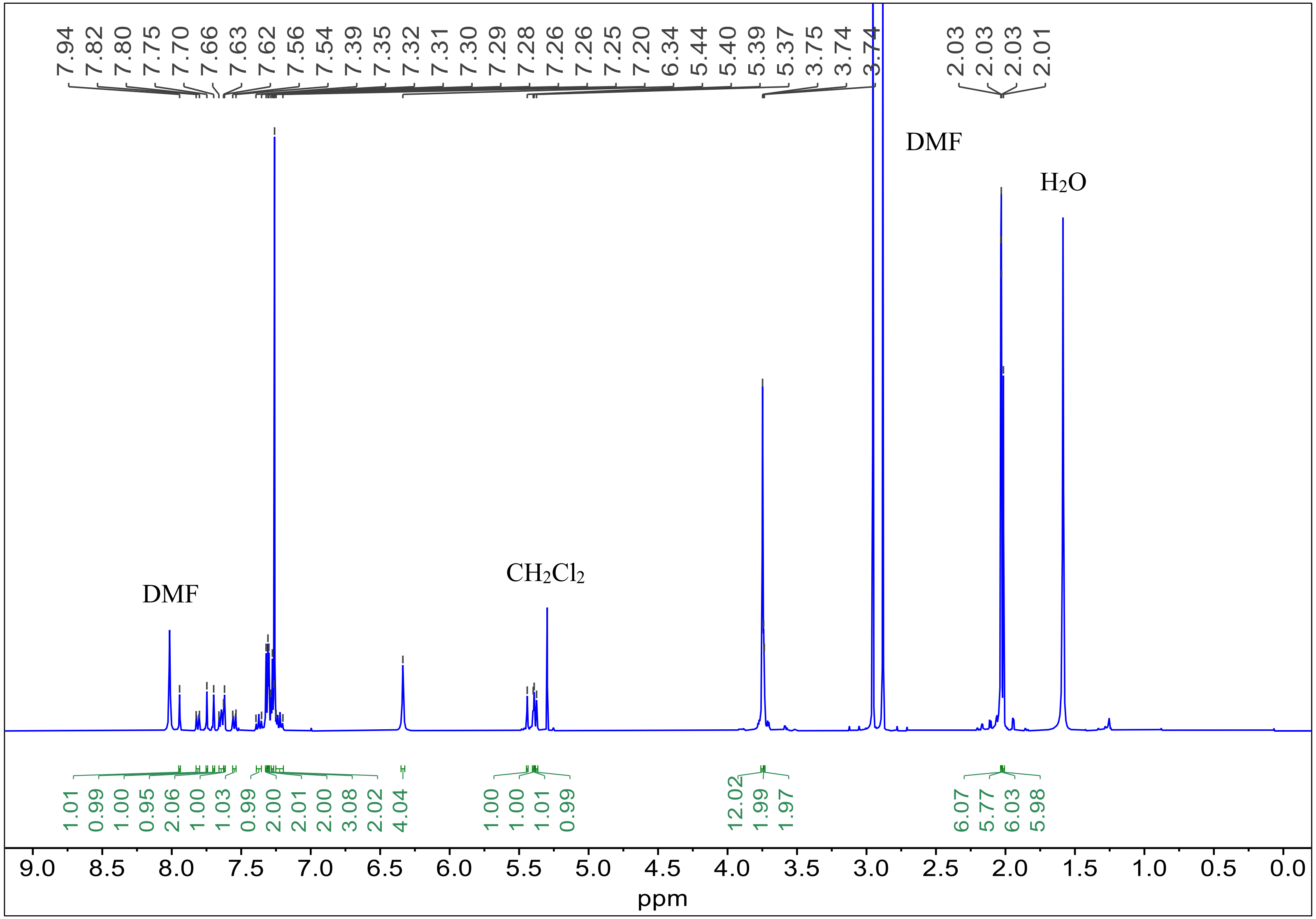

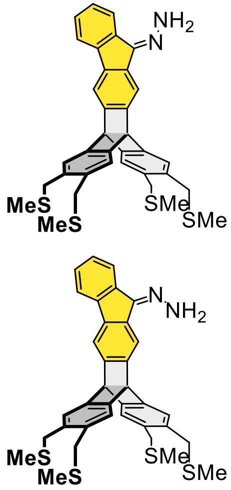

^{1}H NMR (400 MHz, $CDCl_3$): Compound **21** (crude, 1:1 ratio)

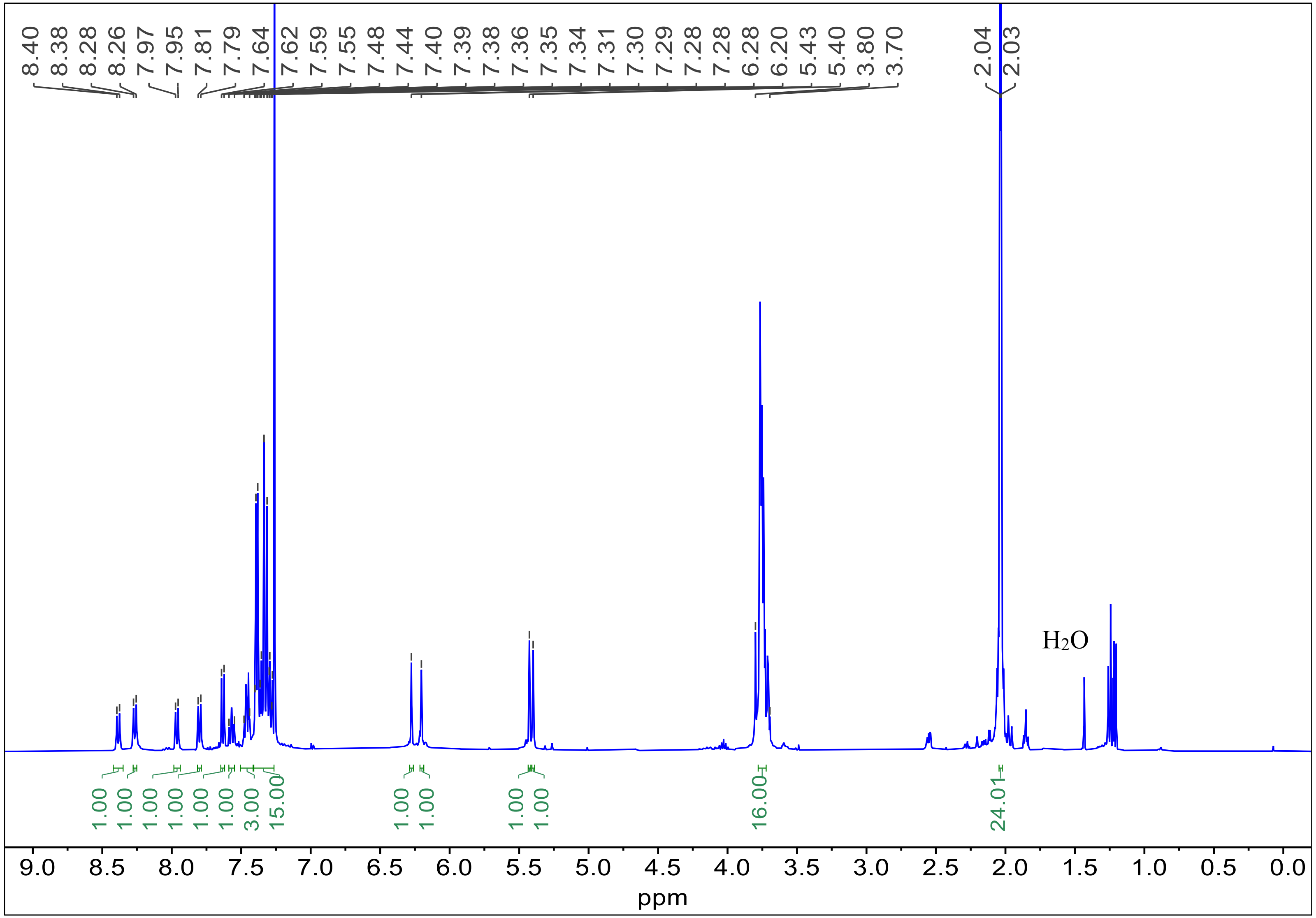

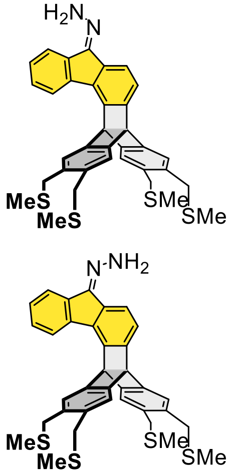

^{1}H NMR (400 MHz, $CDCl_3$): Compound **22** (crude)

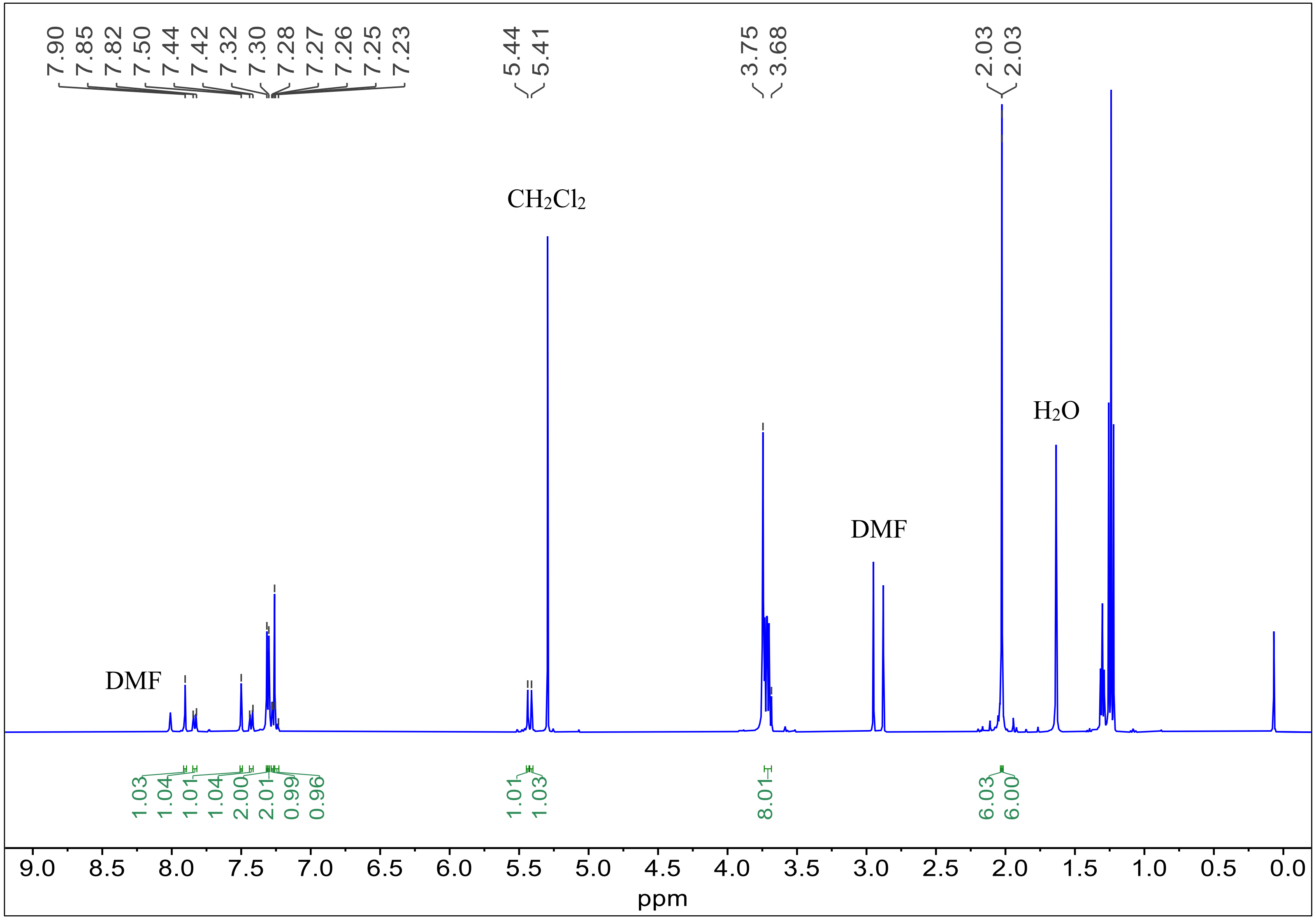

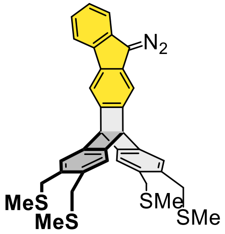

^{1}H NMR (400 MHz, $CDCl_3$): Compound **23** (crude)

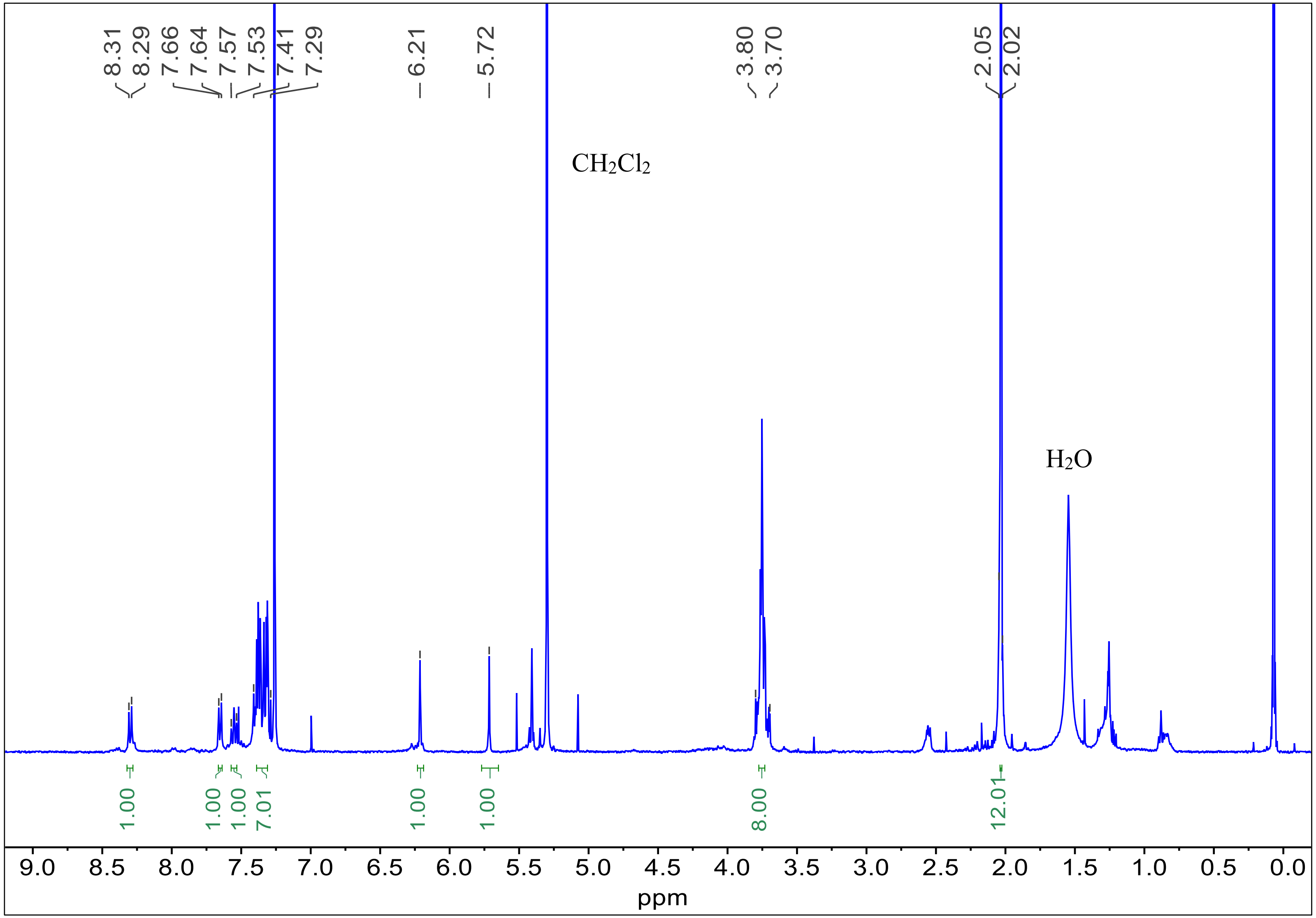

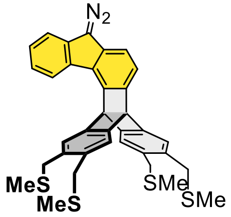

^{1}H NMR (400 MHz, $CDCl_3$): 2-Methyl-2,3-dihydro-1H-benz[e]inden-1-thione (**24**), crude product

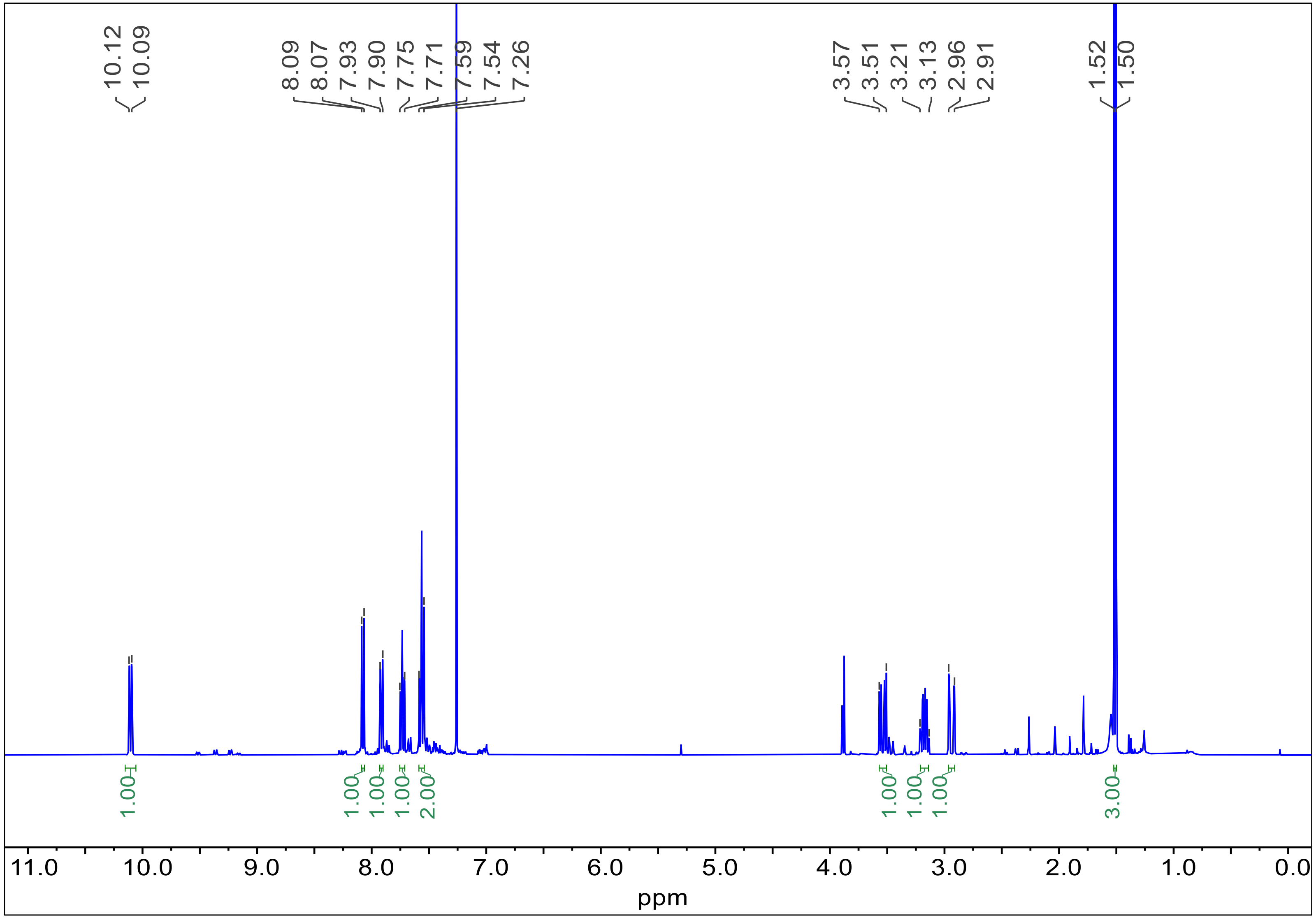

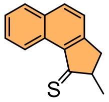

^{1}H NMR (500 MHz, $CDCl_3$): 2-Methyl-2,3-dihydro-1H-benz[e]inden-1-one (**25**)

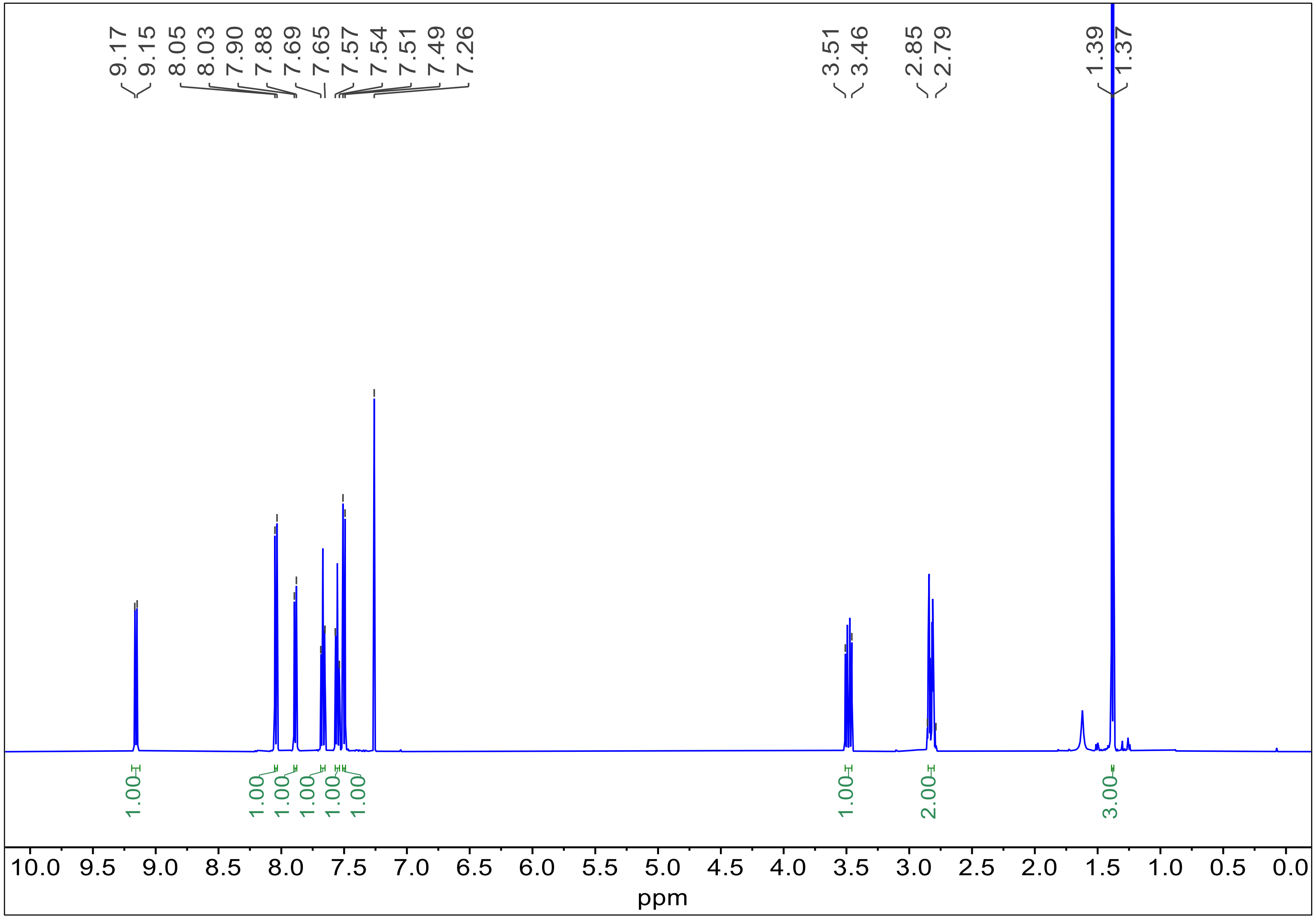

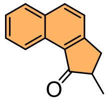

$^{13}C\ \{^1H\}$ NMR (100 MHz, $CDCl_3$): 2-Methyl-2,3-dihydro-1H-benz[e]inden-1-one (**25**)

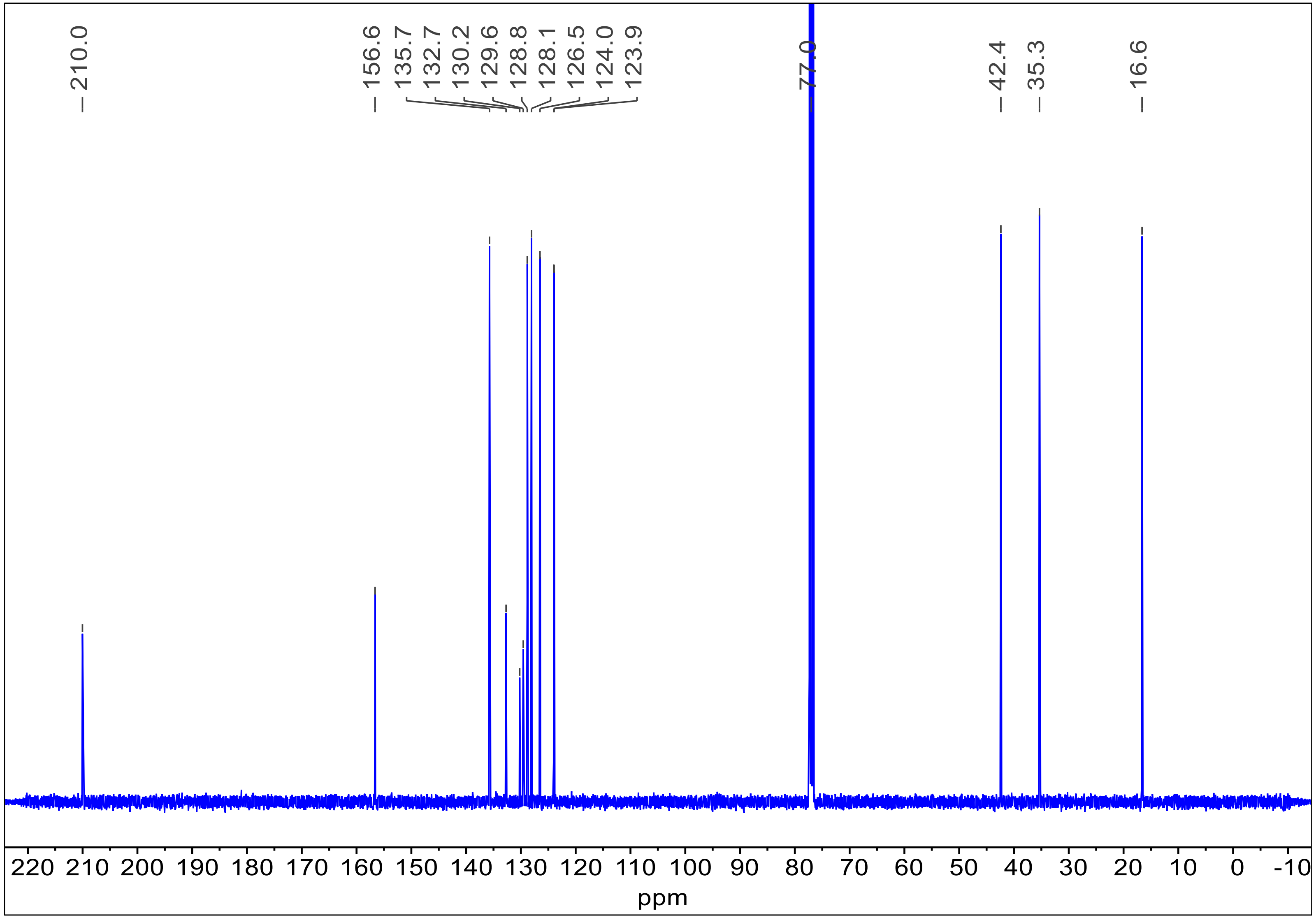

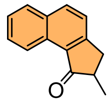

$^{13}C\ \{^{1}H\}$ APT NMR (126 MHz, $CDCl_3$): 2-Methyl-2,3-dihydro-1H-benz[e]inden-1-one (**25**)

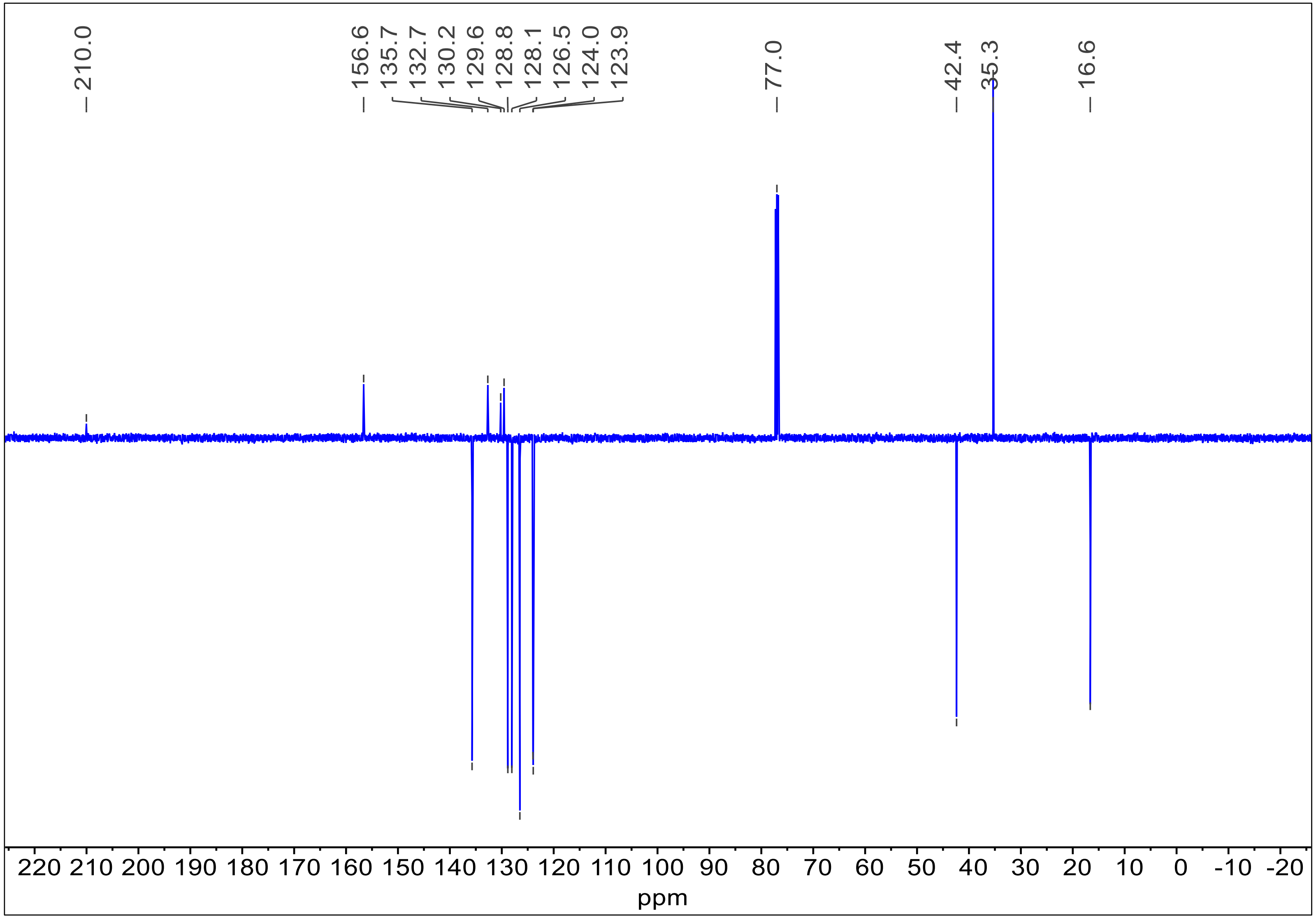

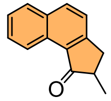

^{1}H – ^{1}H COSY ($CDCl_3$): 2-Methyl-2,3-dihydro-1H-benz[e]inden-1-one (**25**)

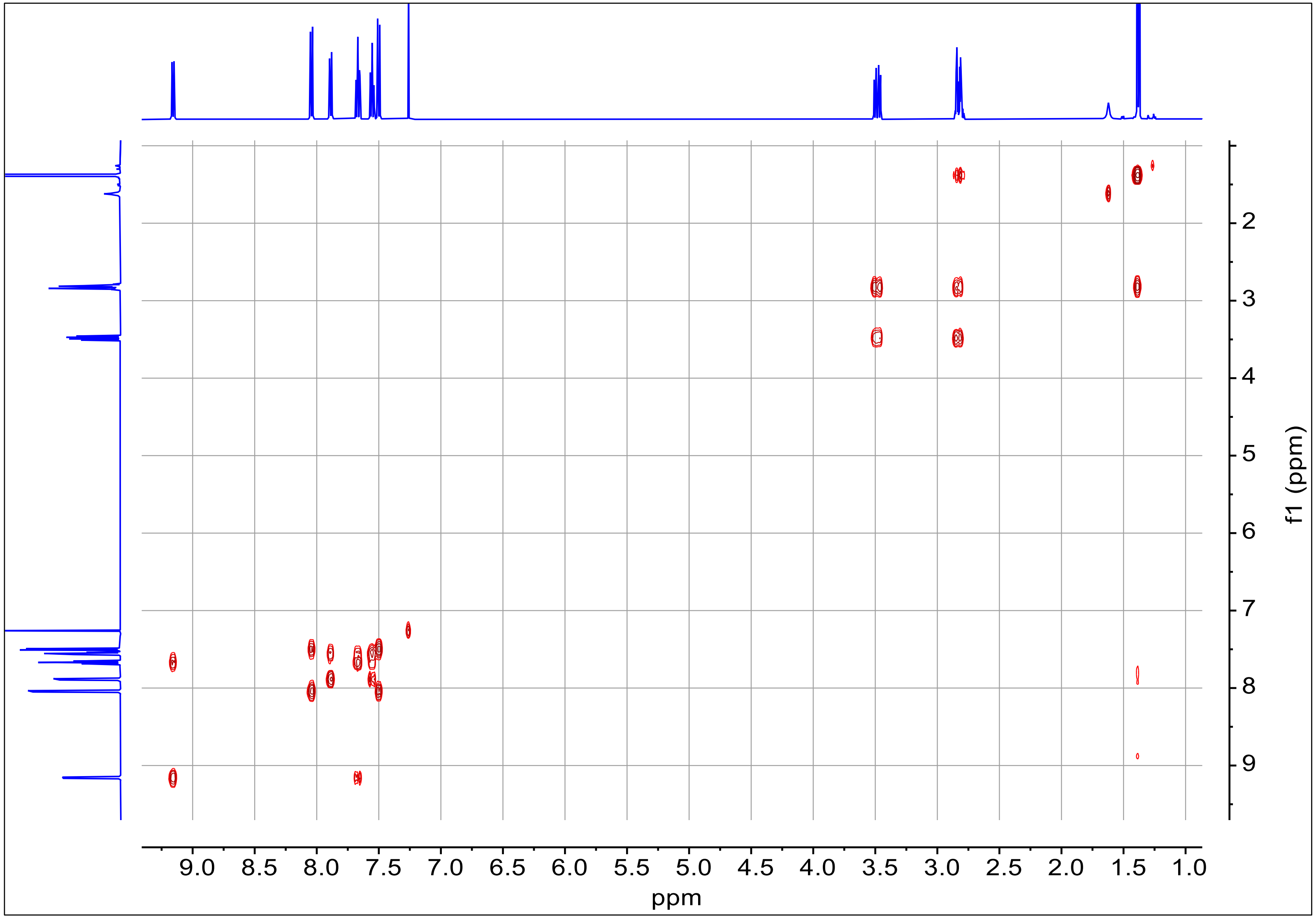

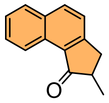

HSQC ($CDCl_3$): 2-Methyl-2,3-dihydro-1H-benz[e]inden-1-one (**25**)

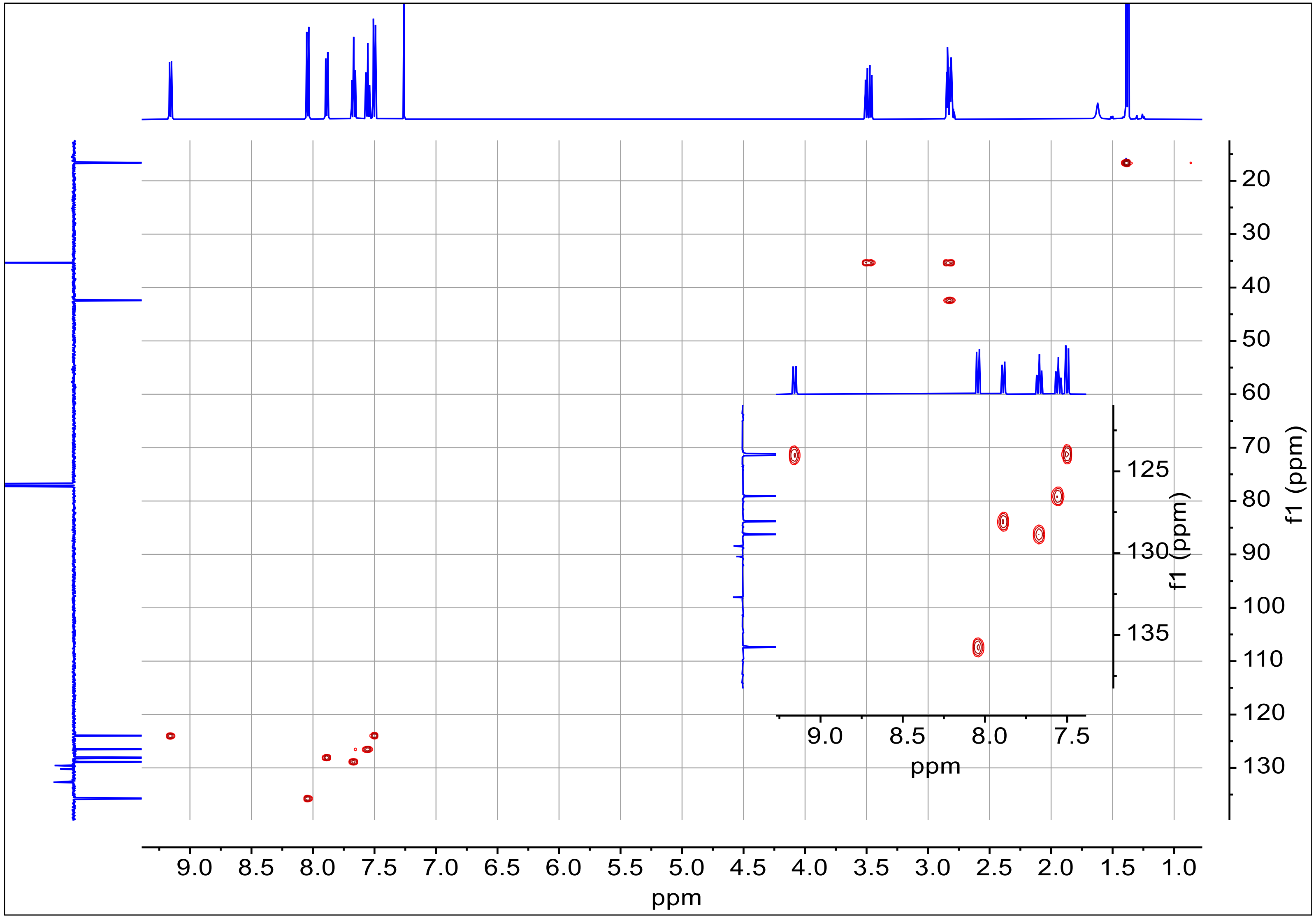

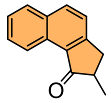

HMBC ($CDCl_3$): 2-Methyl-2,3-dihydro-1H-benz[e]inden-1-one (**25**)

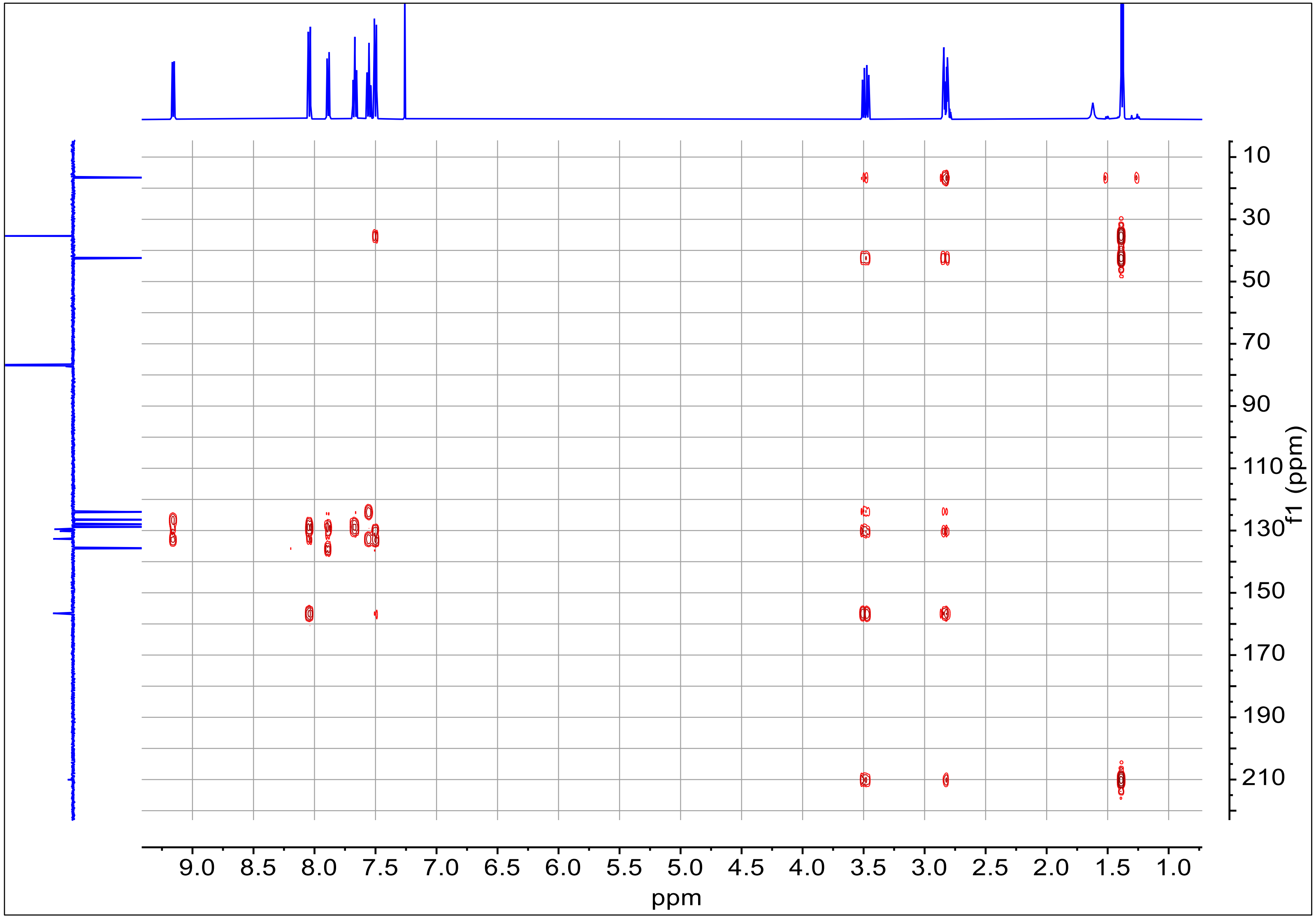

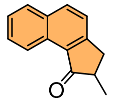

^{1}H NMR (400 MHz, DMSO-d_6): 2-Amino-5-bromo-4-(methoxycarbonyl)benzoic acid (**6**)

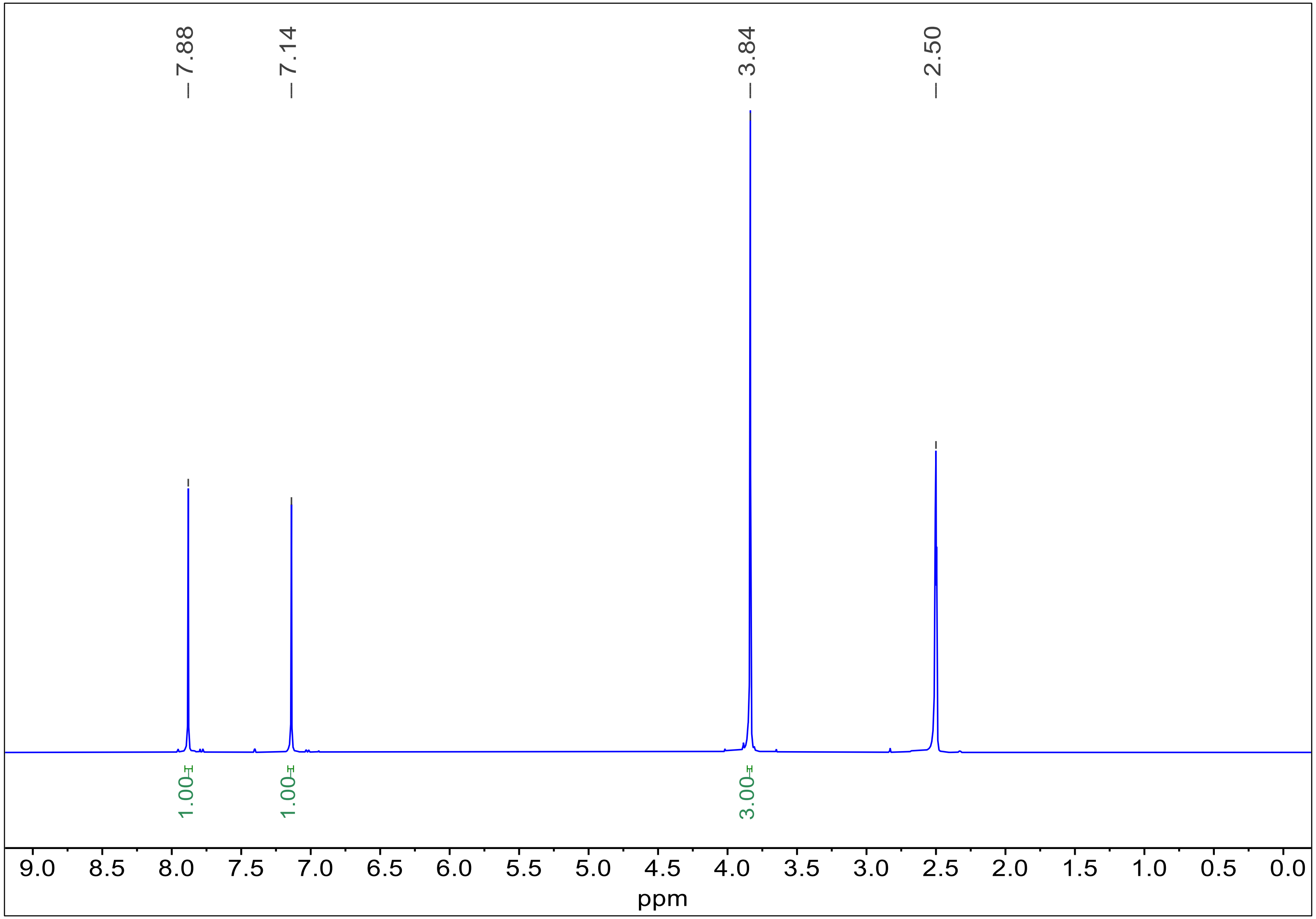

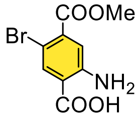

$^{13}C\ \{^{1}H\}$ NMR (100 MHz, DMSO-d_6): 2-Amino-5-bromo-4-(methoxycarbonyl)benzoic acid (**6**)

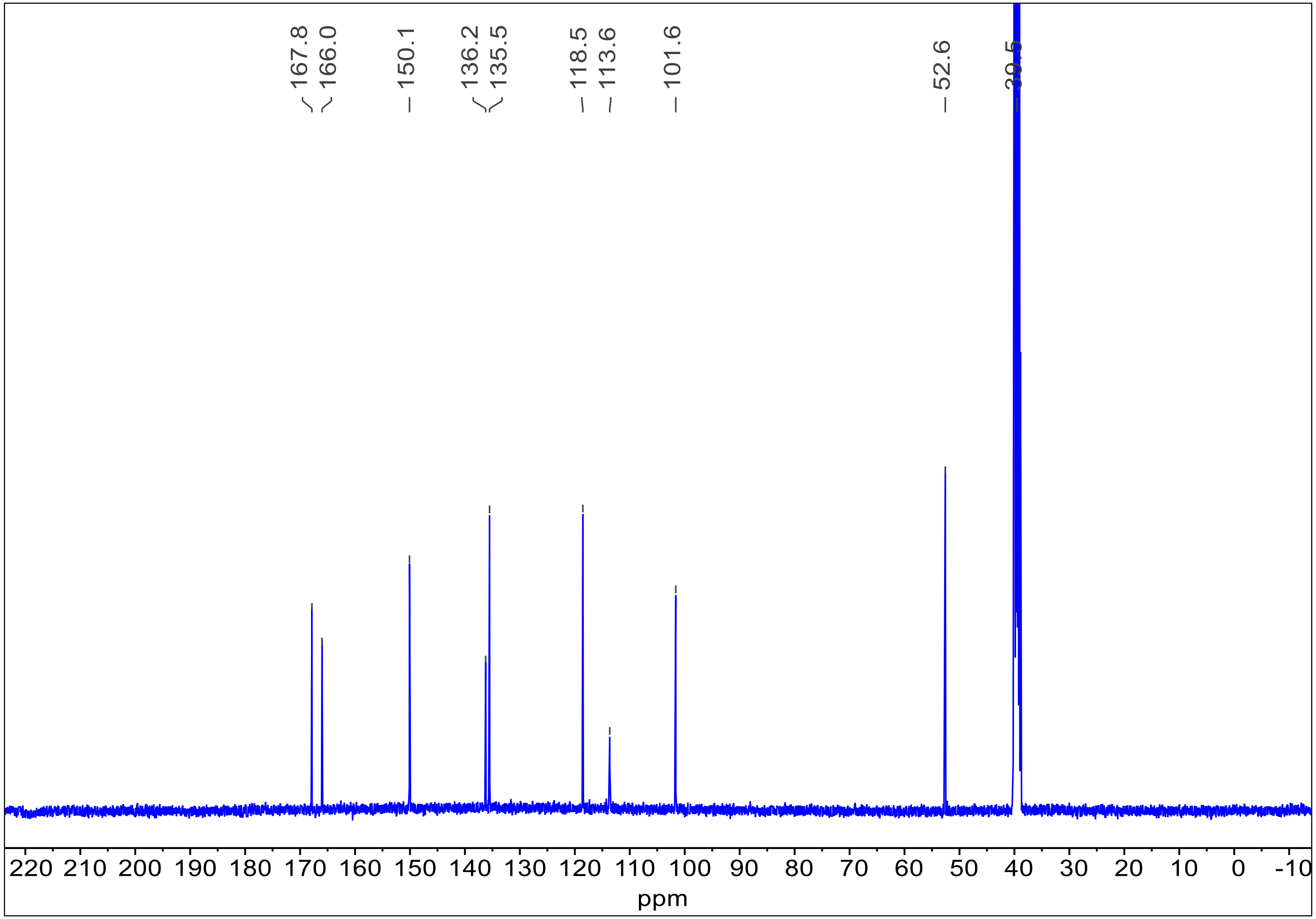

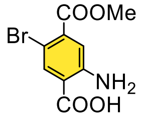

^{13}C {^{1}H} APT NMR (100 MHz, DMSO-d_6): 2-Amino-5-bromo-4-(methoxycarbonyl)benzoic acid (**6**)

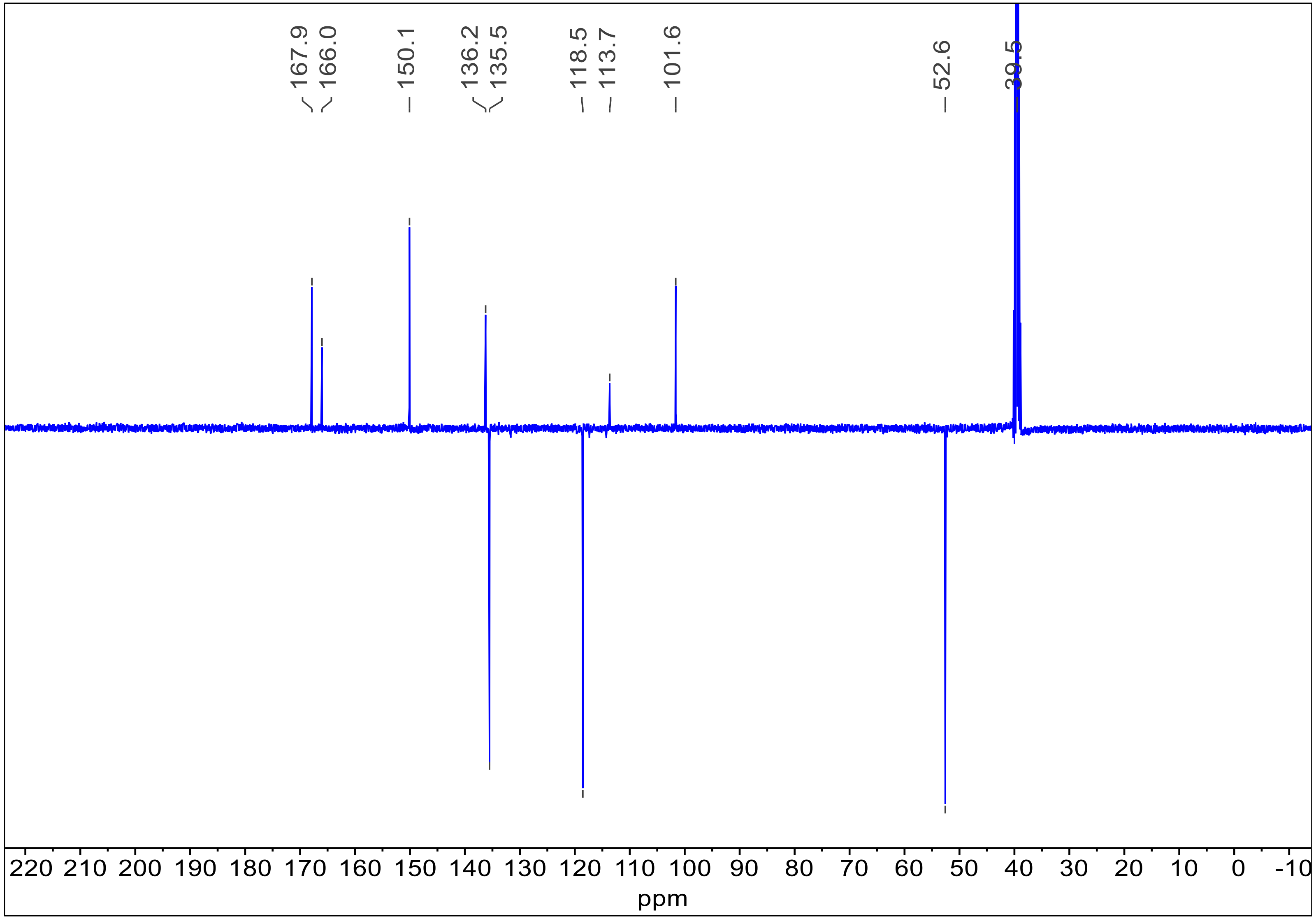

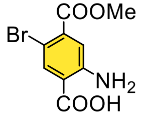

^{1}H – ^{1}H COSY (DMSO-d_6): 2-Amino-5-bromo-4-(methoxycarbonyl)benzoic acid (**6**)

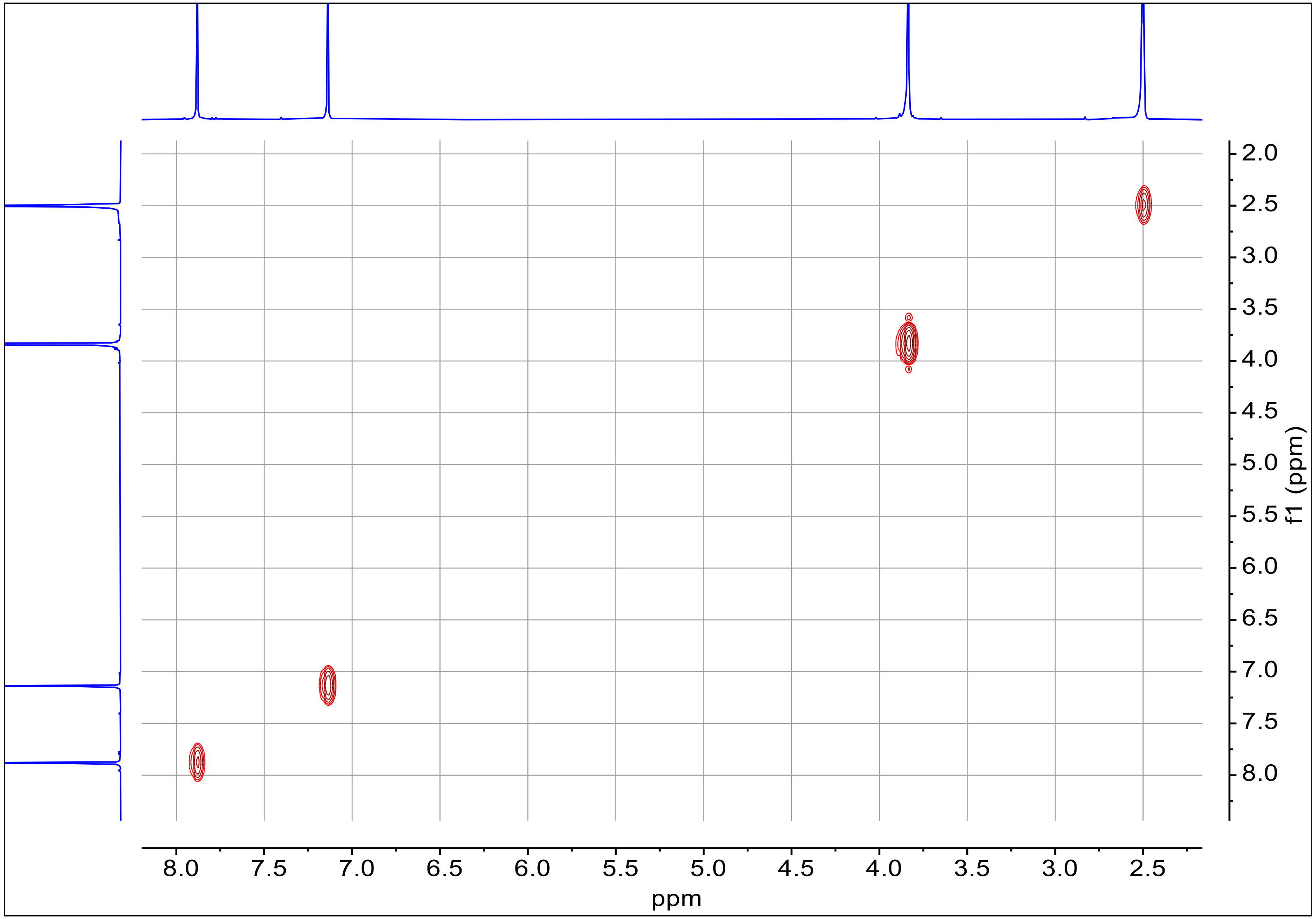

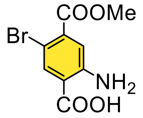

HSQC (DMSO-d_6): 2-Amino-5-bromo-4-(methoxycarbonyl)benzoic acid (**6**)

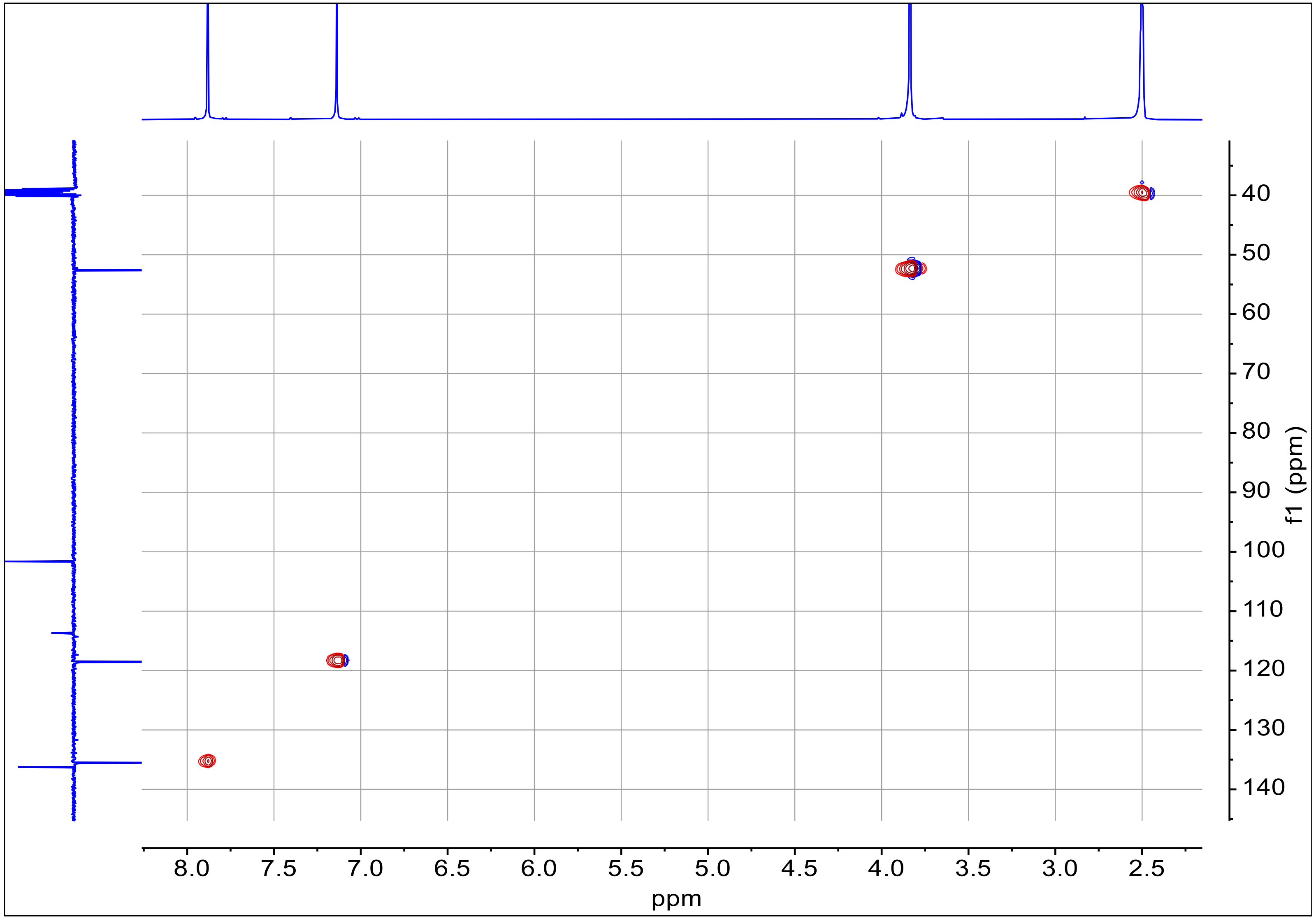

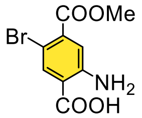

HMBC (DMSO-d_6): 2-Amino-5-bromo-4-(methoxycarbonyl)benzoic acid (**6**)

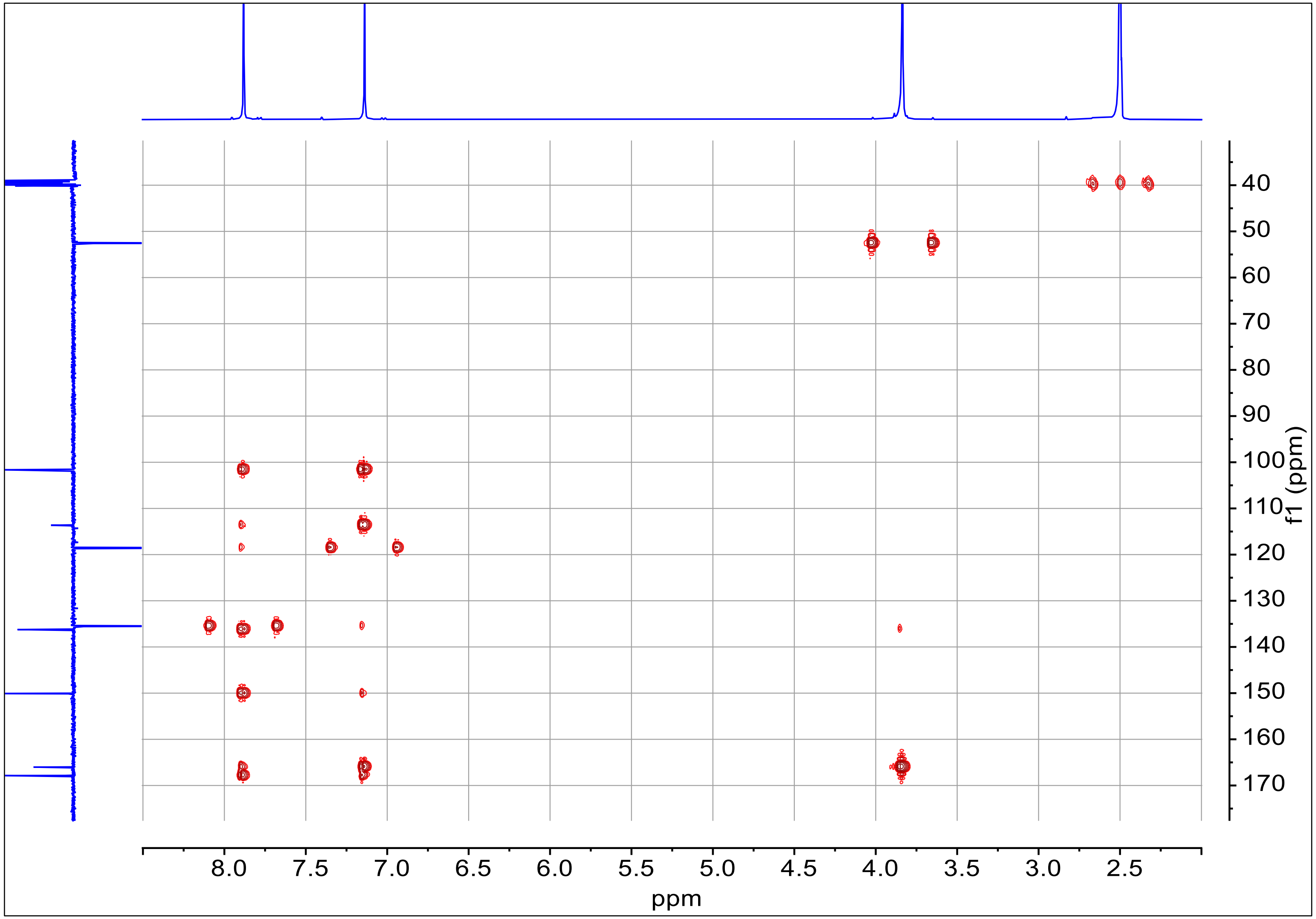

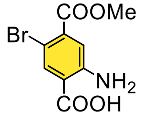

^{1}H NMR (400 MHz, $CDCl_3$): Compound **S2** (crude)

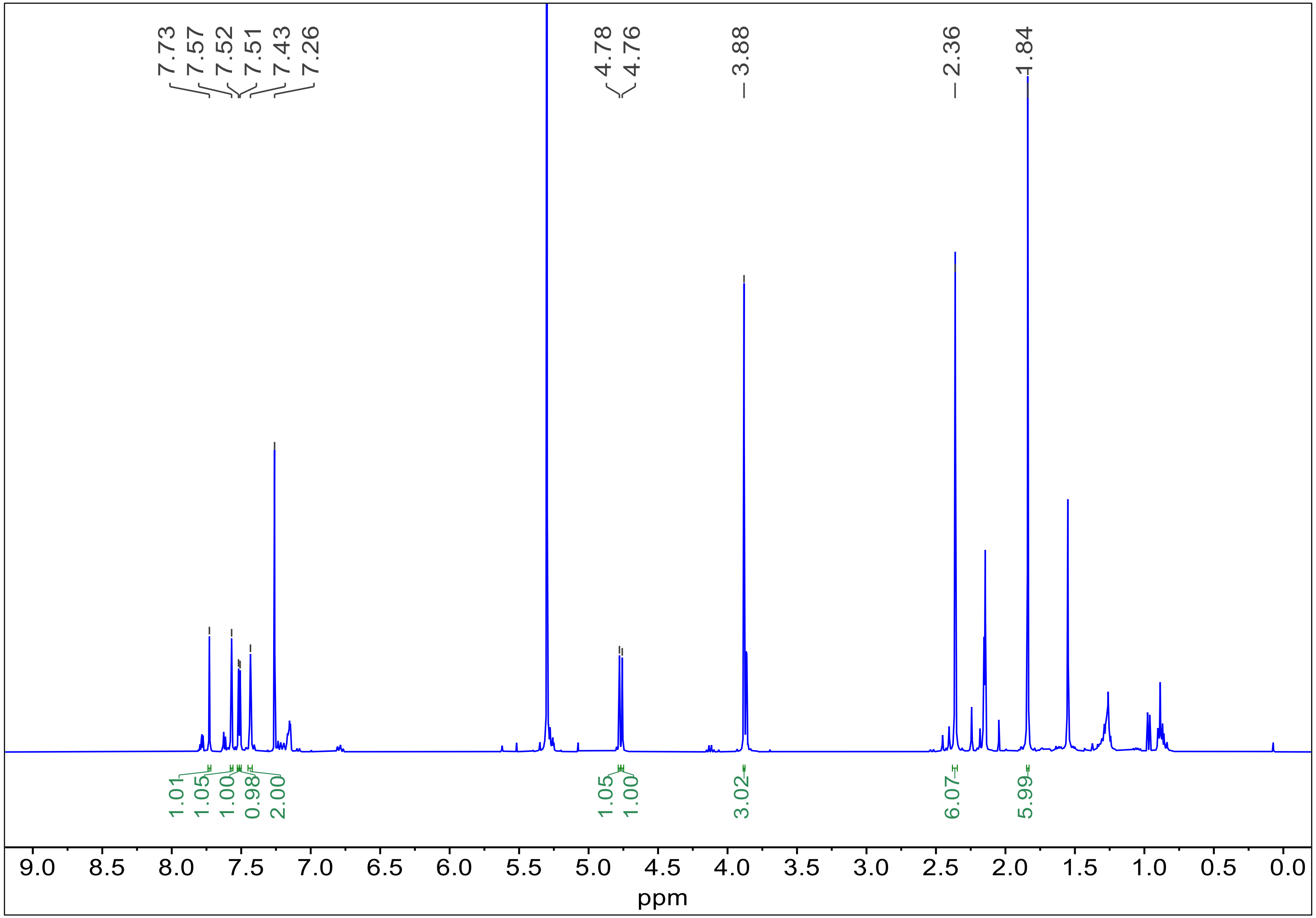

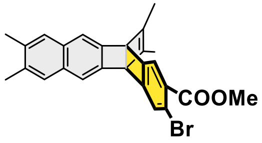

^{1}H NMR (400 MHz, $CDCl_3$): Compound **S2**

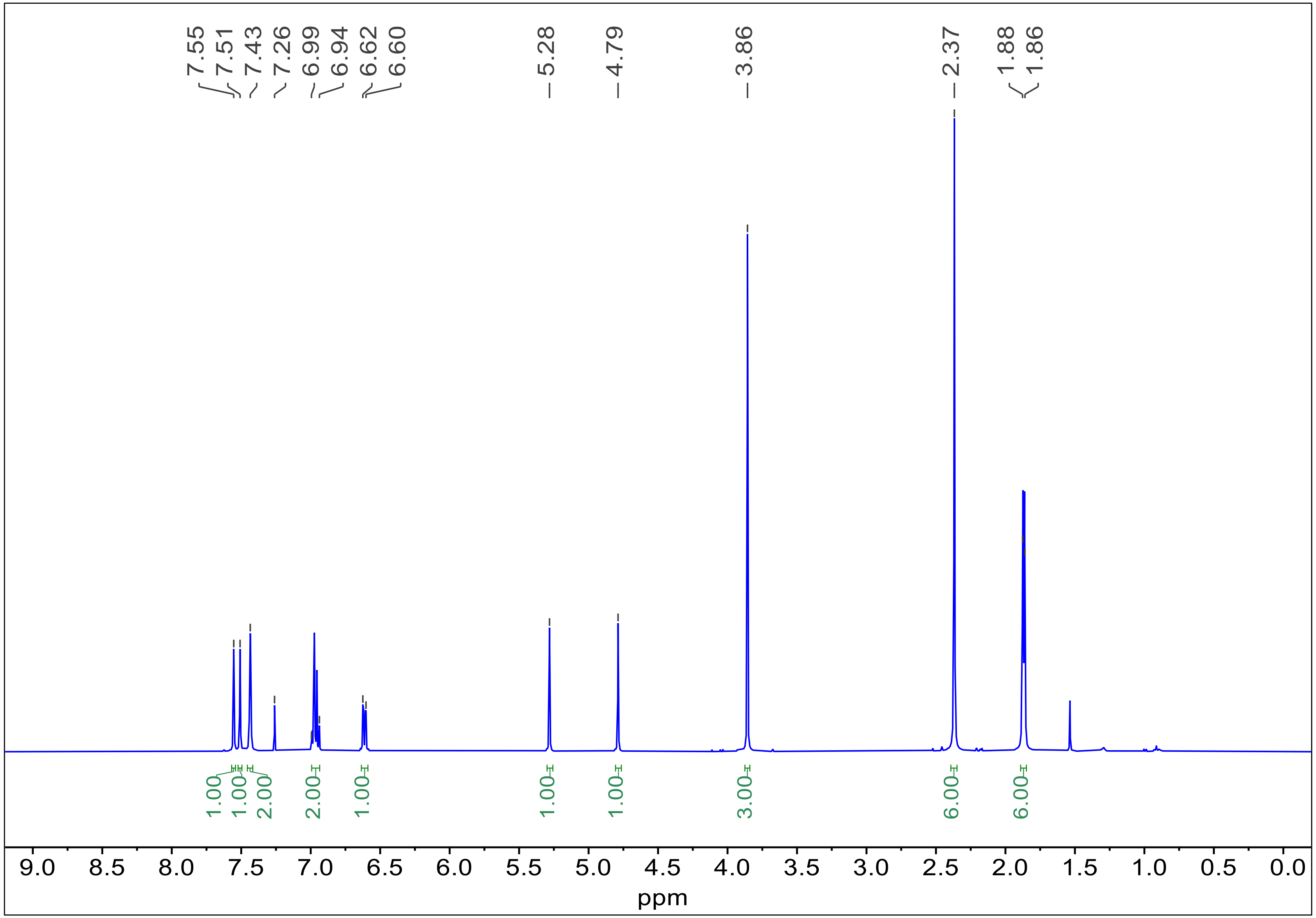

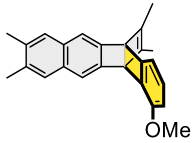

^{13}C {^{1}H} NMR (100 MHz, $CDCl_3$): Compound **S2**

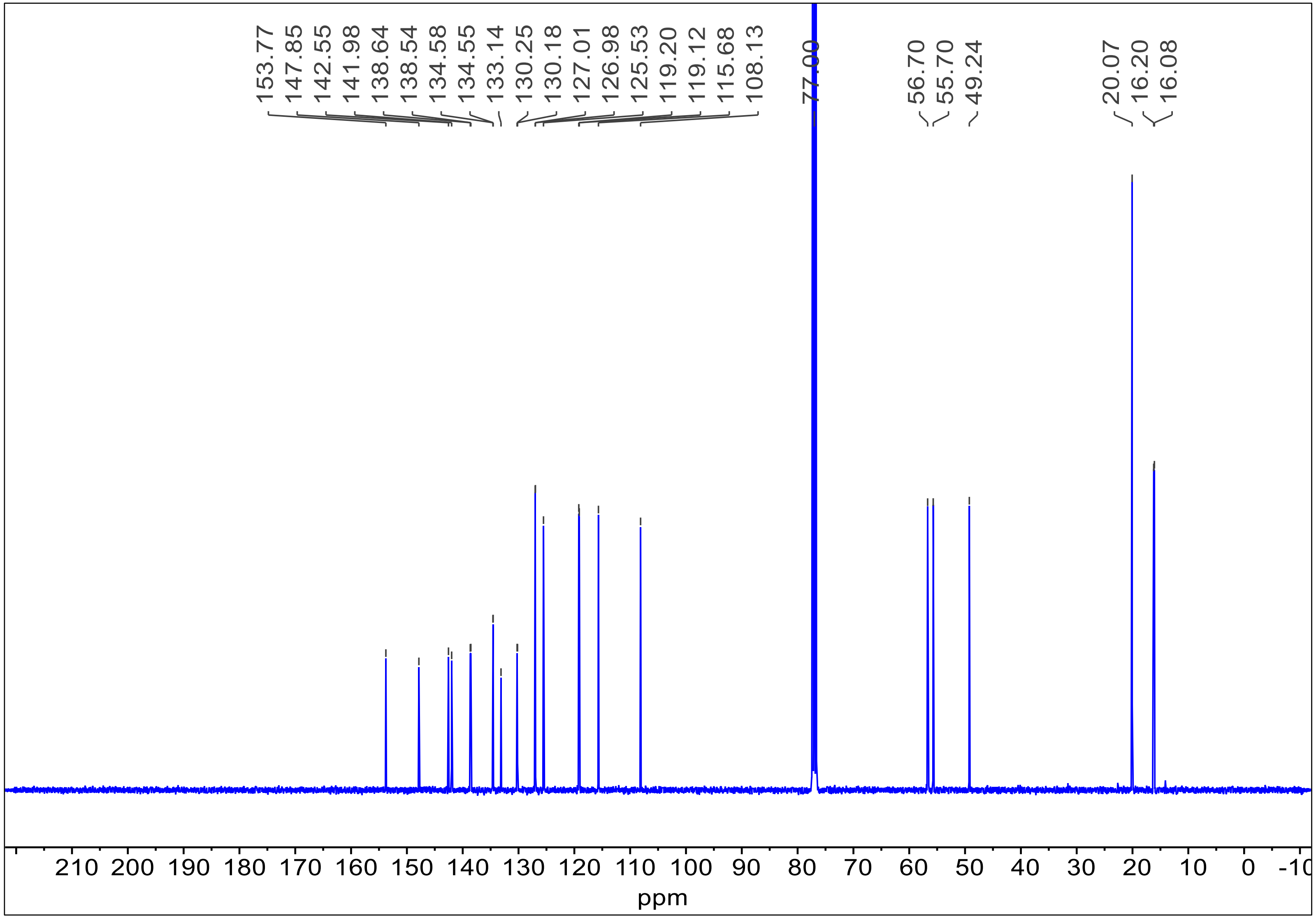

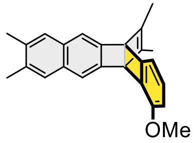

$^{13}C\{^{1}H\}$ APT NMR (100 MHz, $CDCl_3$): Compound **S2**

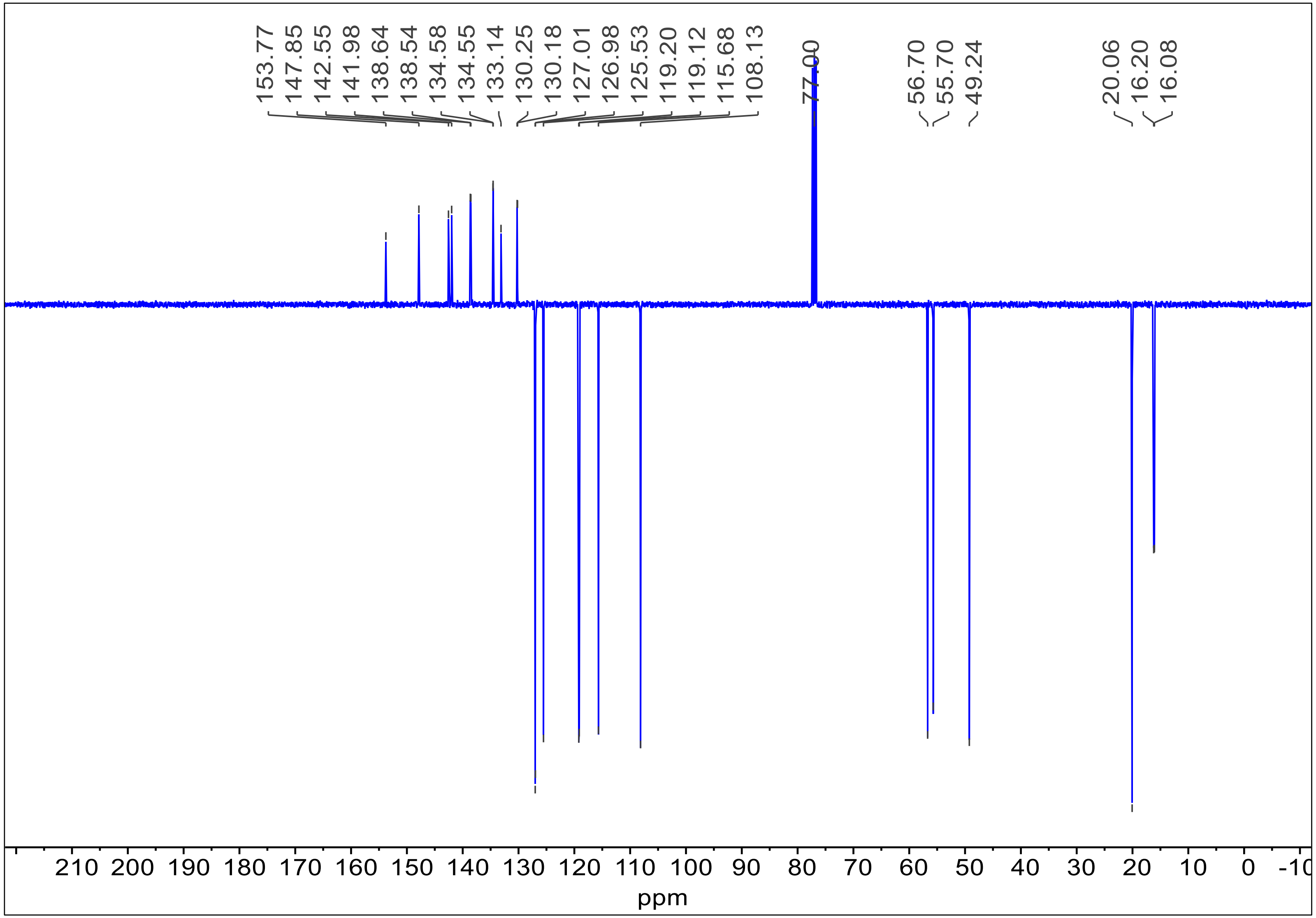

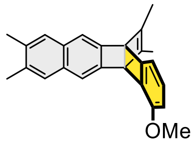

^{1}H – ^{1}H COSY ($CDCl_3$): Compound **S2**

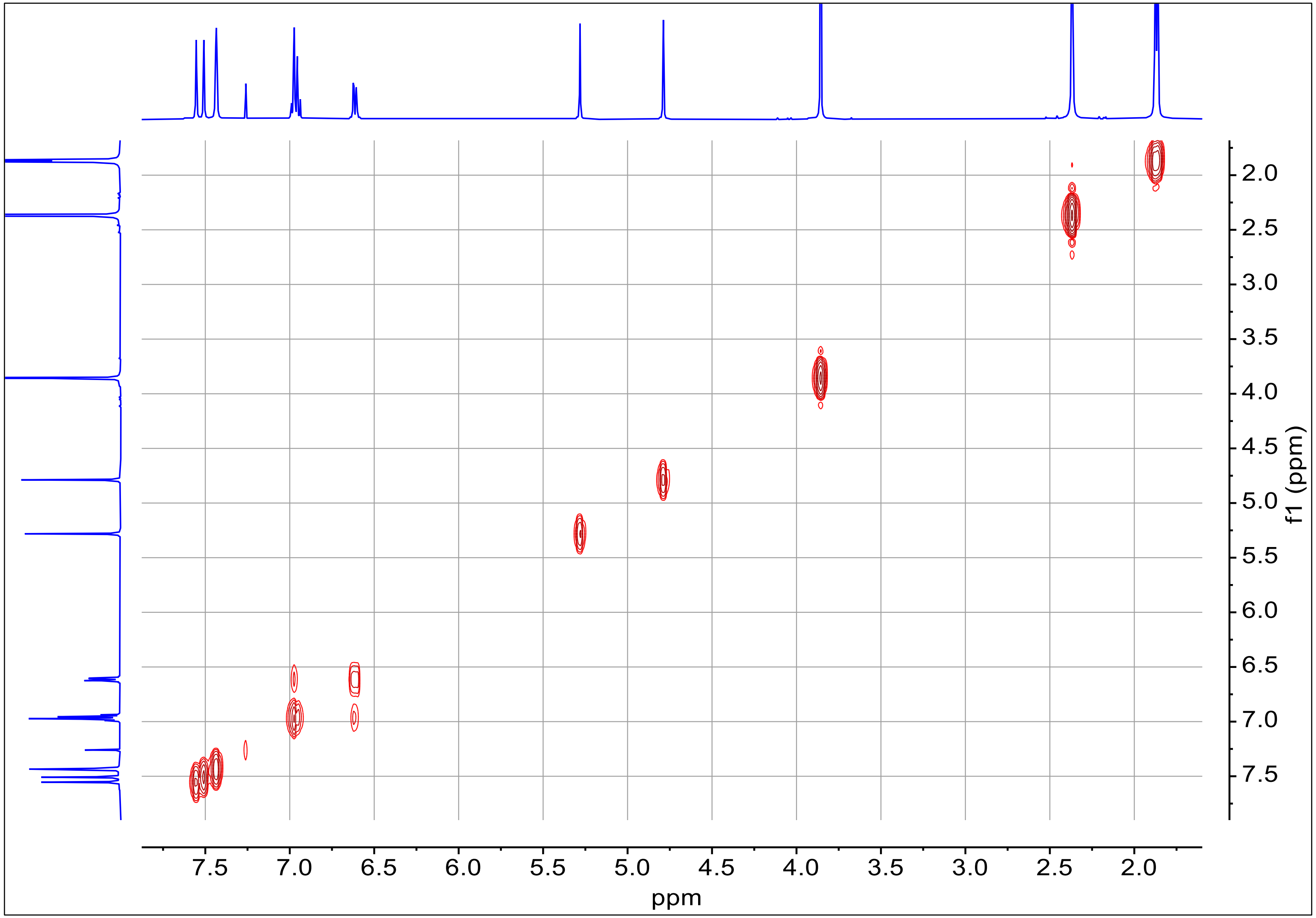

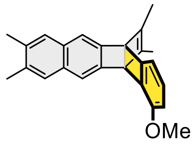

HSQC ($CDCl_3$): Compound **S2**

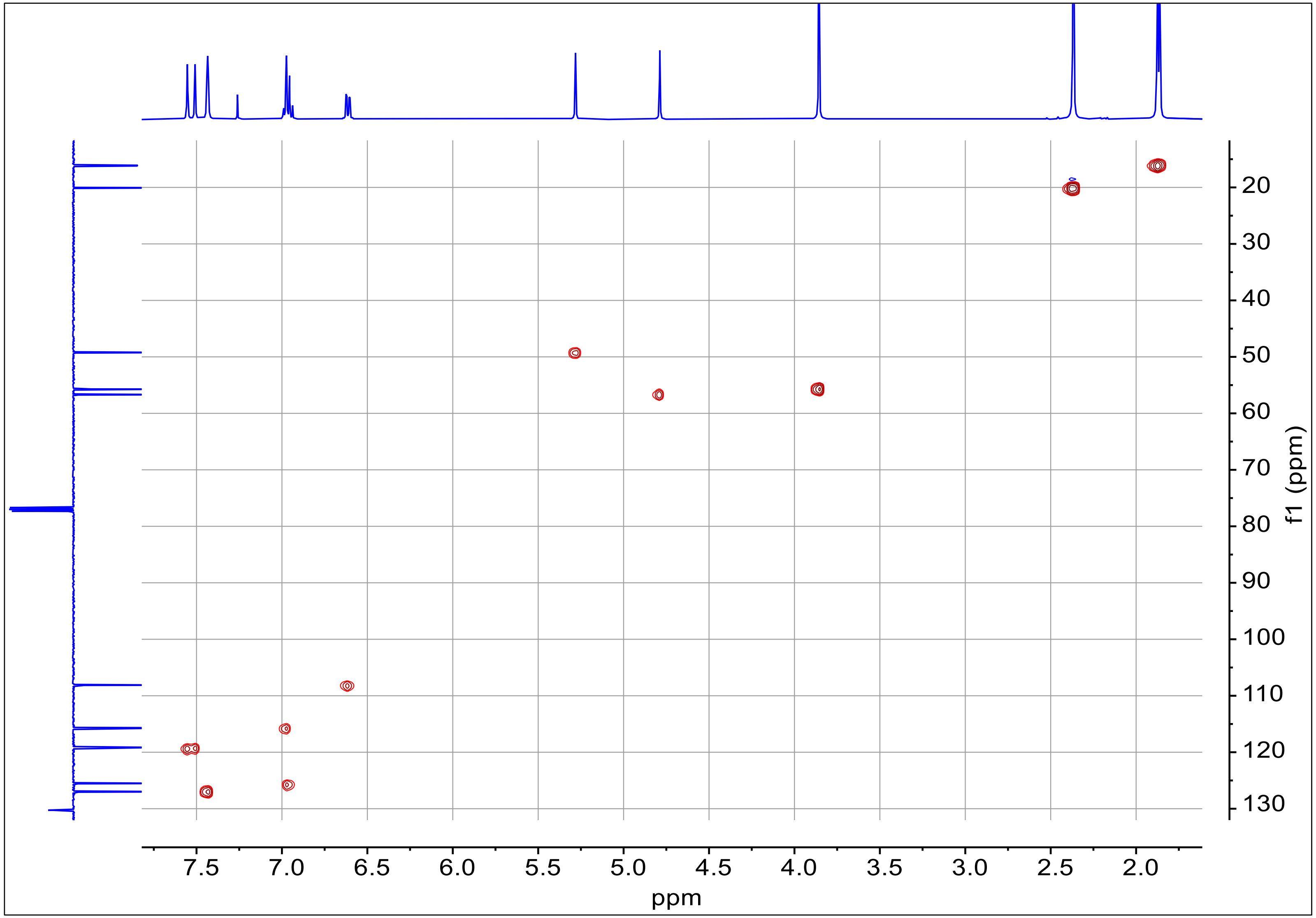

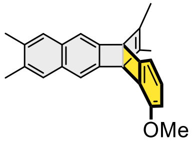

HMBC ($CDCl_3$): Compound **S2**

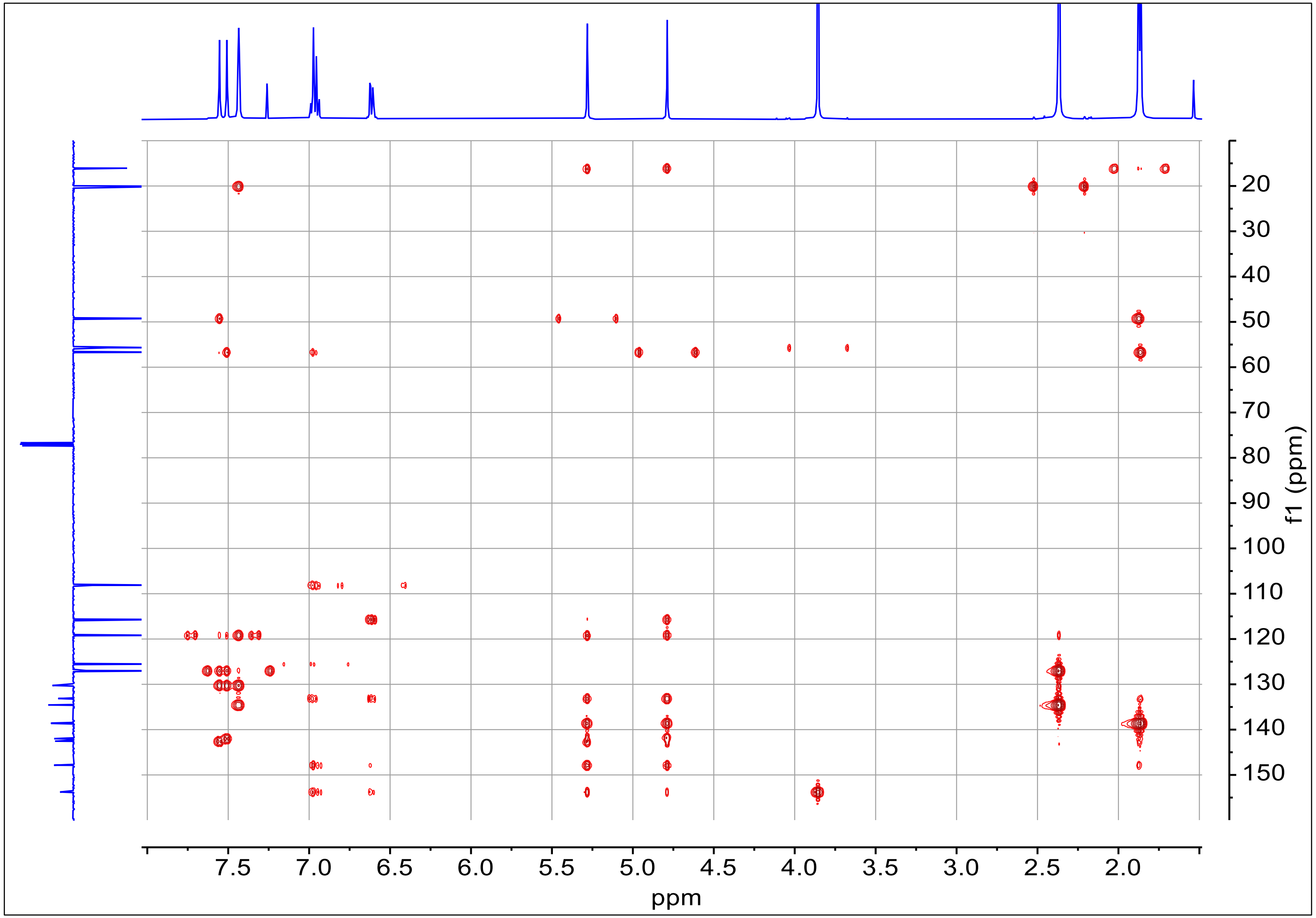

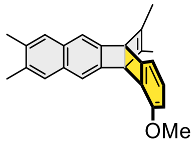

^{1}H NMR (400 MHz, CDCl3): Dimethyl 2-amino-5-bromoterephthalate (**S3**)

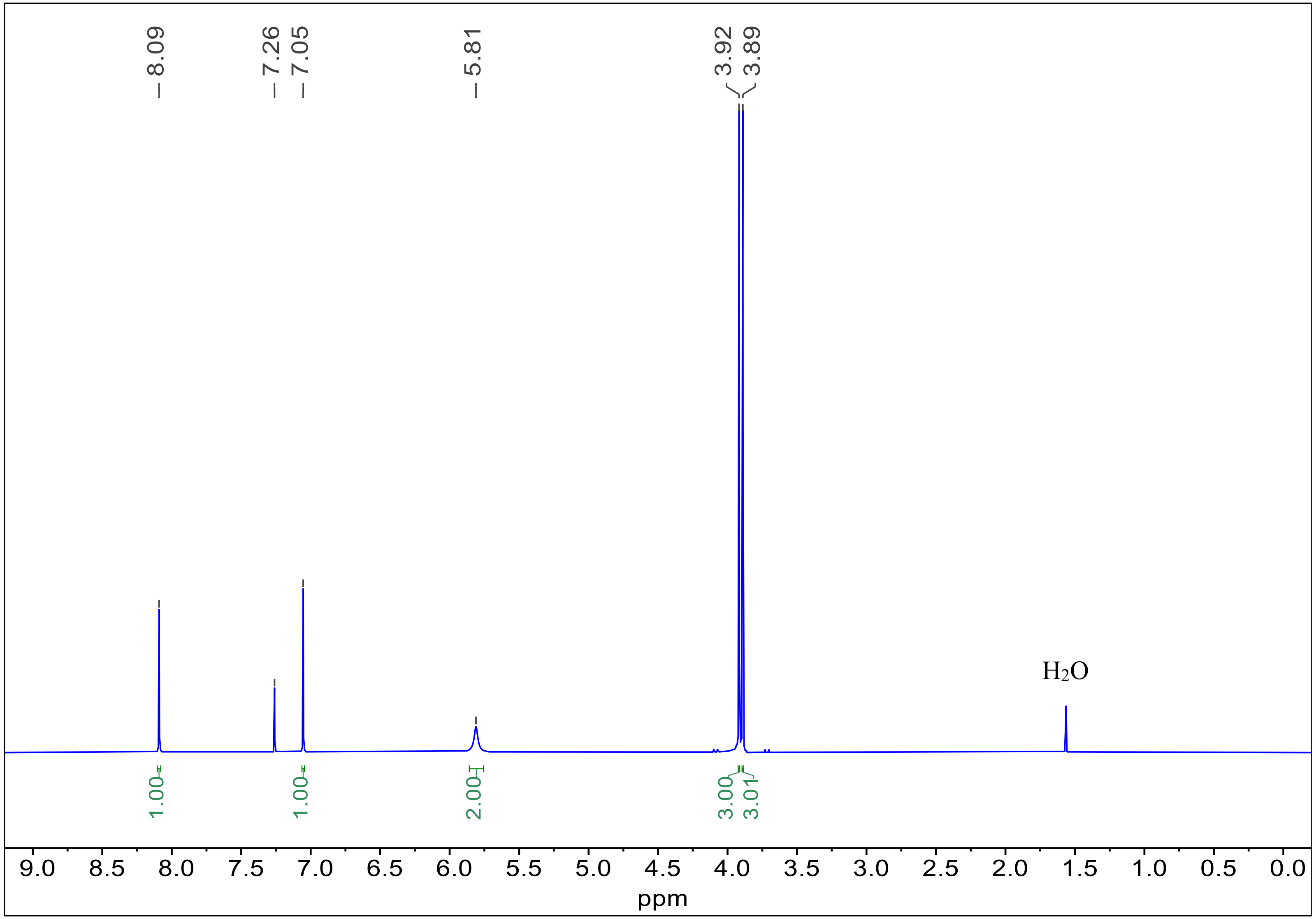

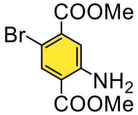

^{13}C {^{1}H} NMR (100 MHz, $CDCl_3$): Dimethyl 2-amino-5-bromoterephthalate (**S3**)

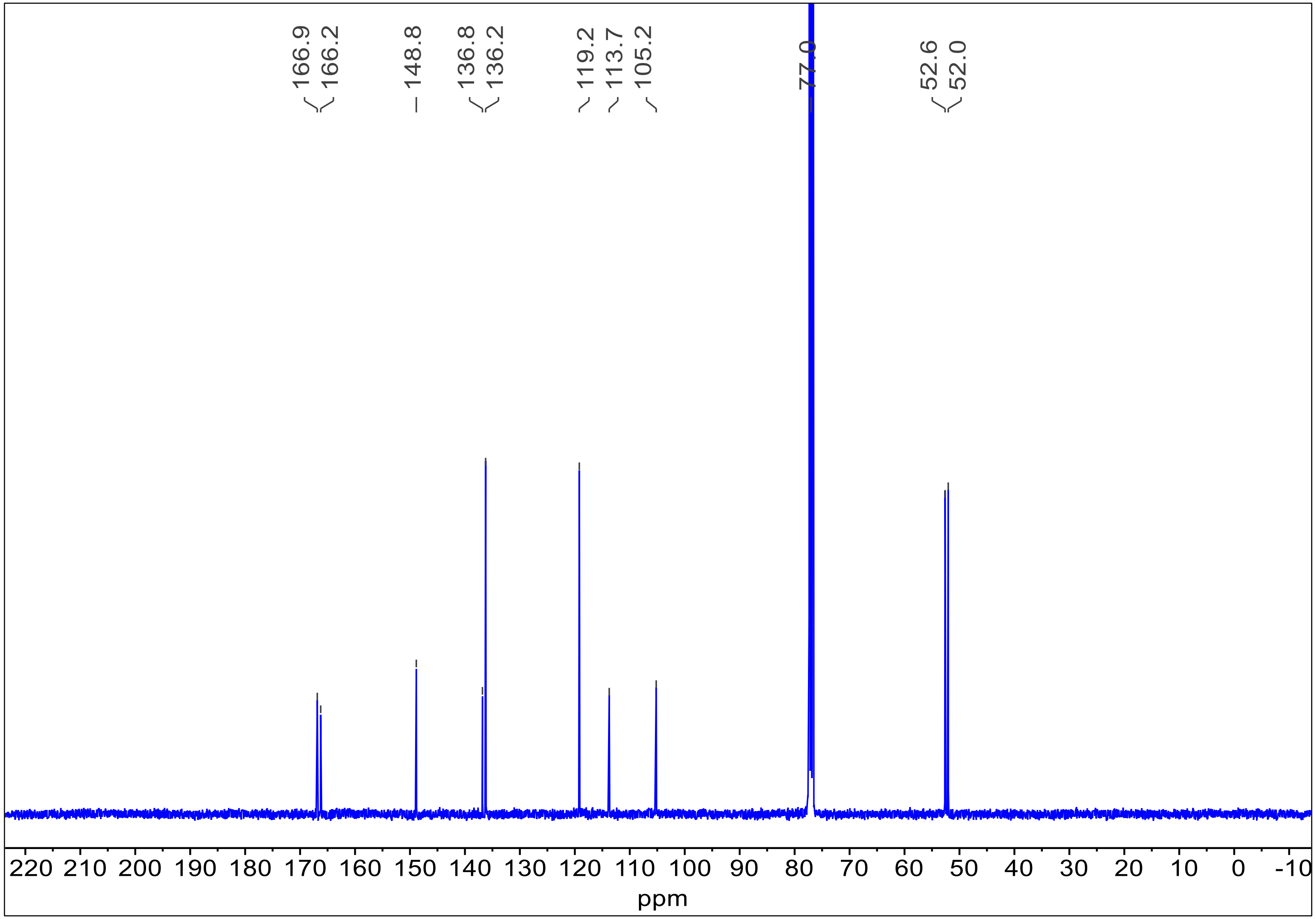

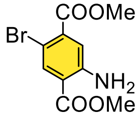

$^{13}C\ \{^1H\}$ APT NMR (100 MHz, $CDCl_3$): Dimethyl 2-amino-5-bromoterephthalate (**S3**)

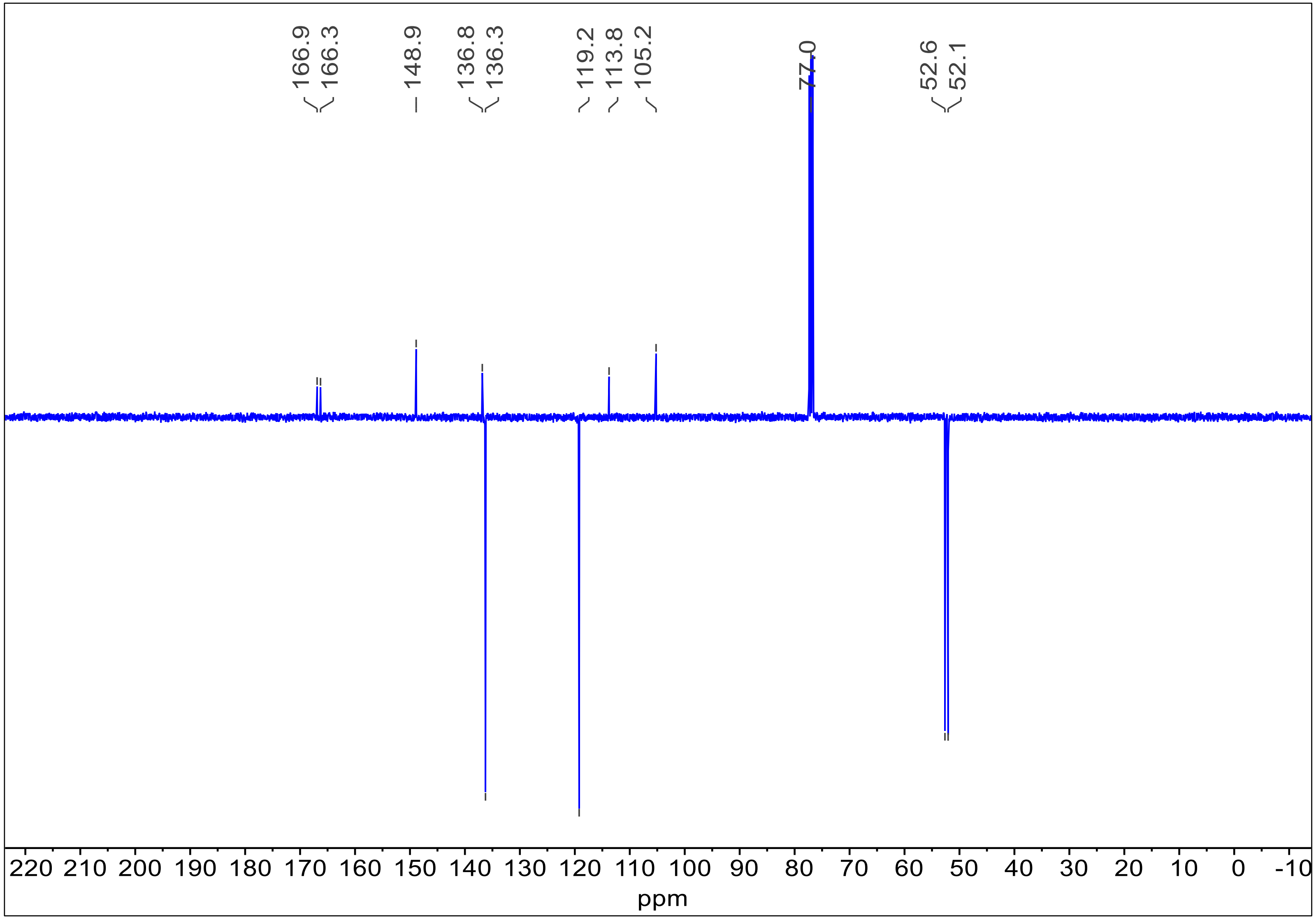

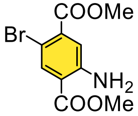

^{1}H – ^{1}H COSY ($CDCl_3$): Dimethyl 2-amino-5-bromoterephthalate (**S3**)

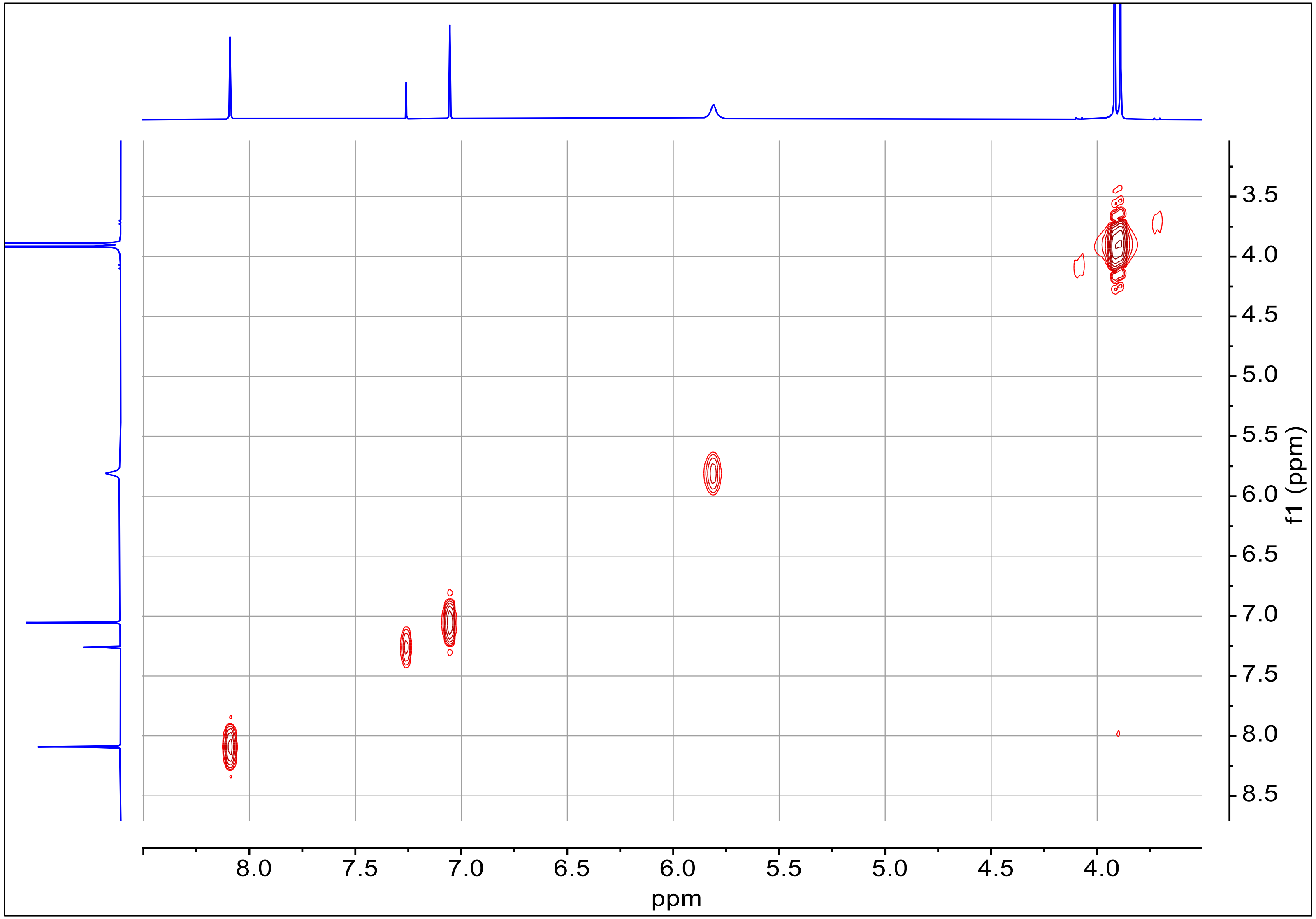

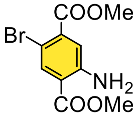

HSQC ($CDCl_3$): Dimethyl 2-amino-5-bromoterephthalate (**S3**)

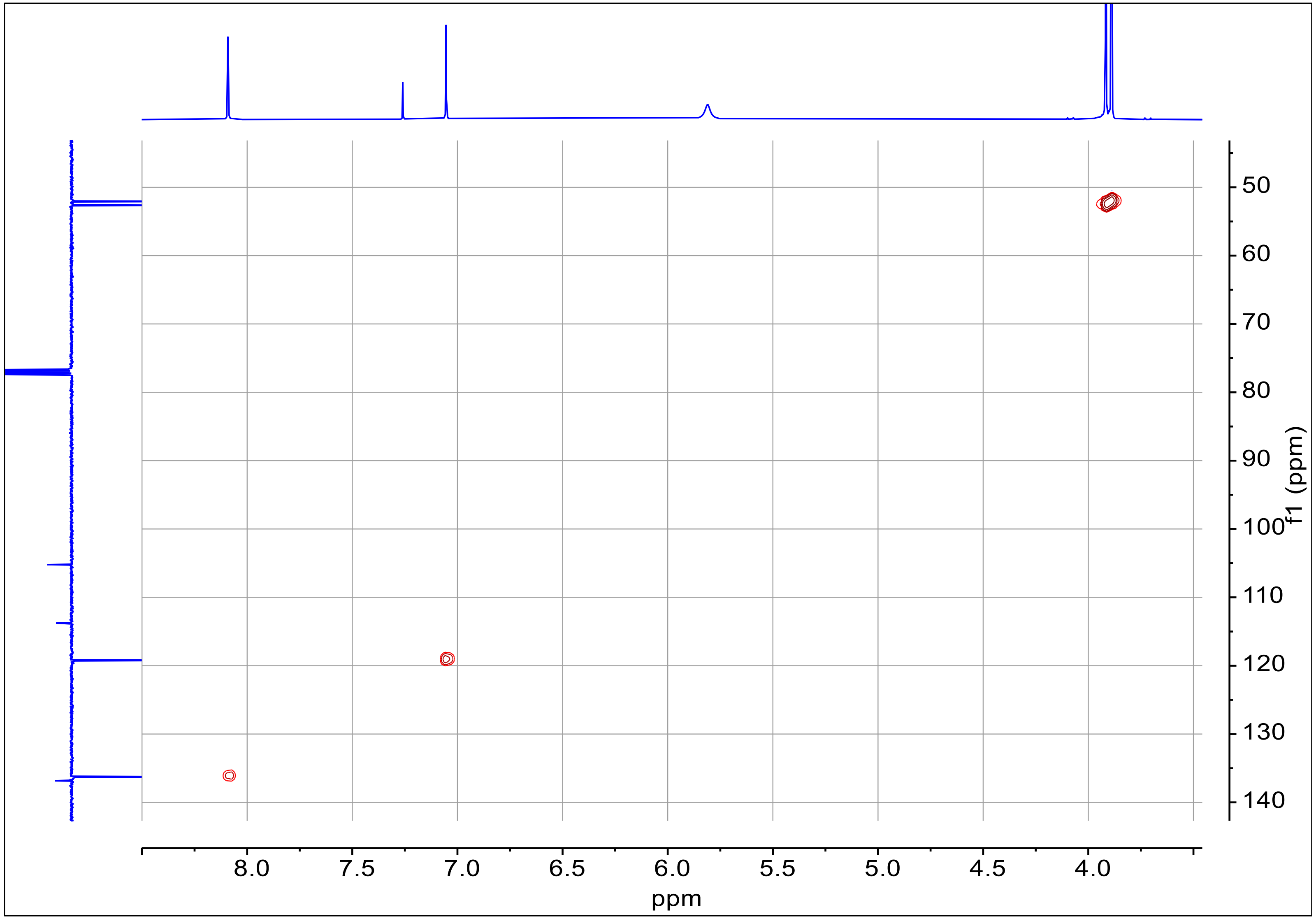

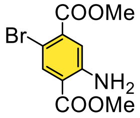

HMBC ($CDCl_3$): Dimethyl 2-amino-5-bromoterephthalate (**S3**)

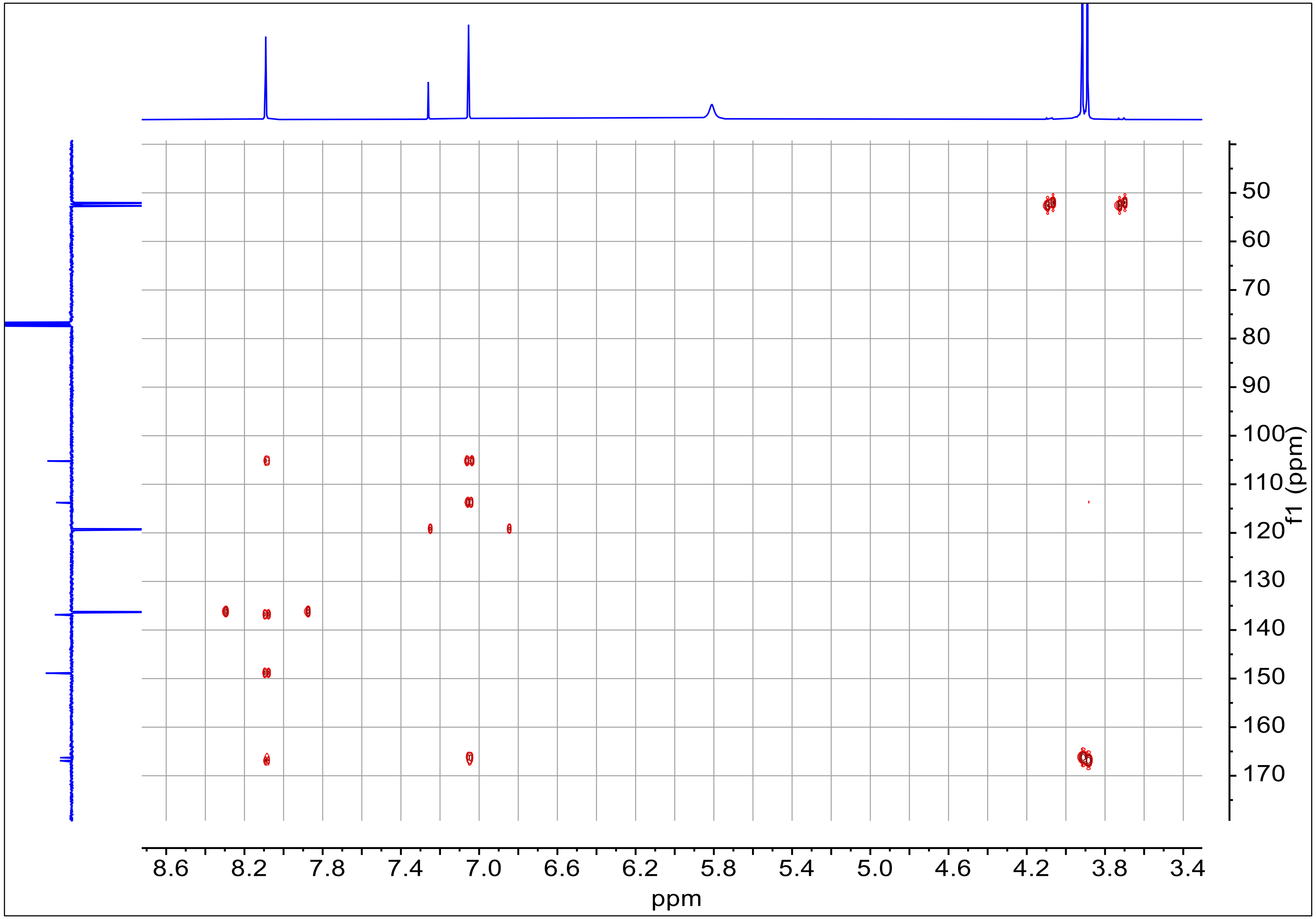

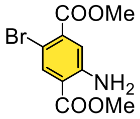

^{1}H NMR (400 MHz, $CDCl_3$): Dimethyl 2-amino-3-bromoterephthalate (**S4**)

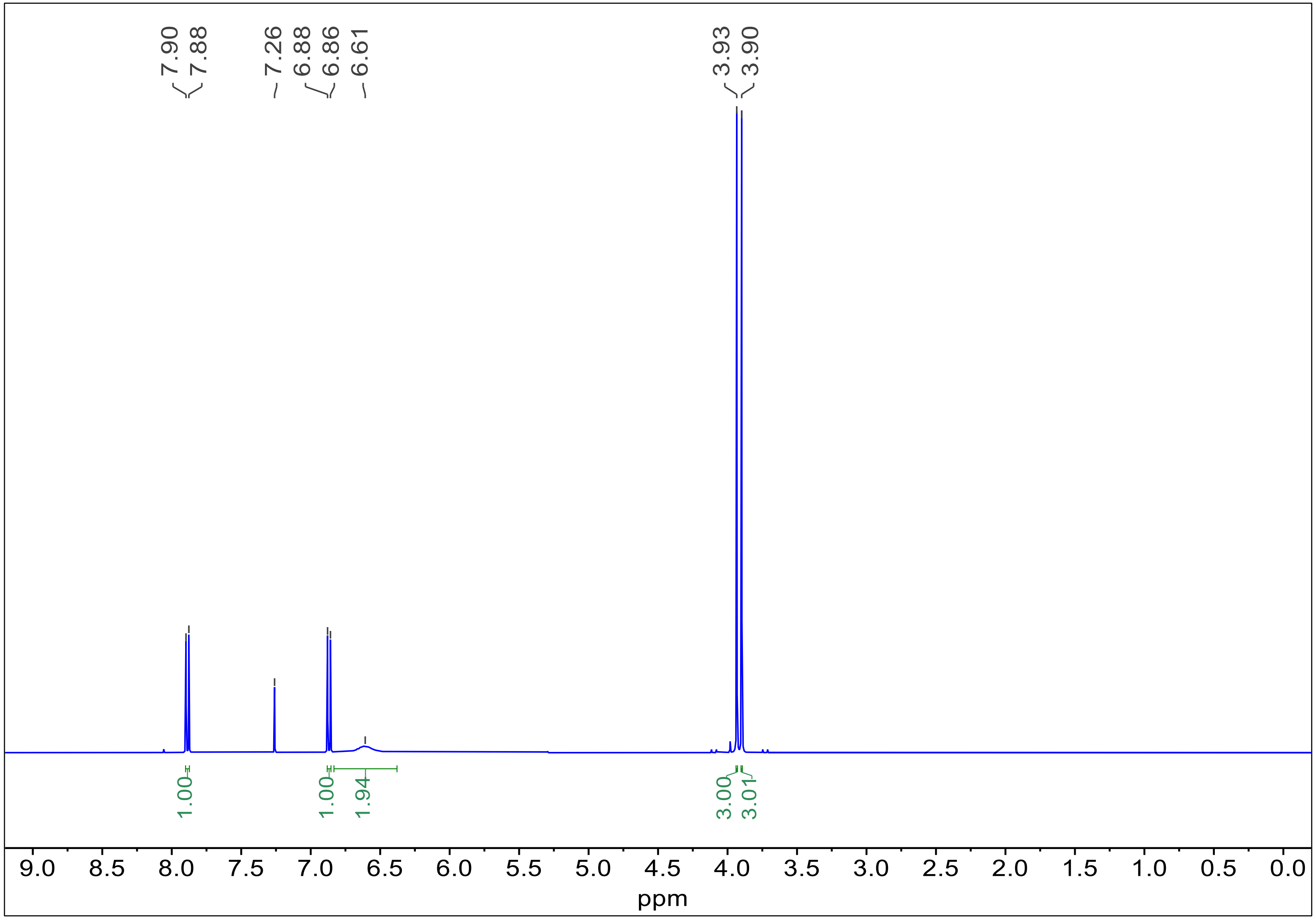

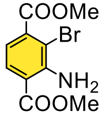

$^{13}C\{^{1}H\}$ APT NMR (100 MHz, $CDCl_3$): Dimethyl 2-amino-3-bromoterephthalate (**S4**)

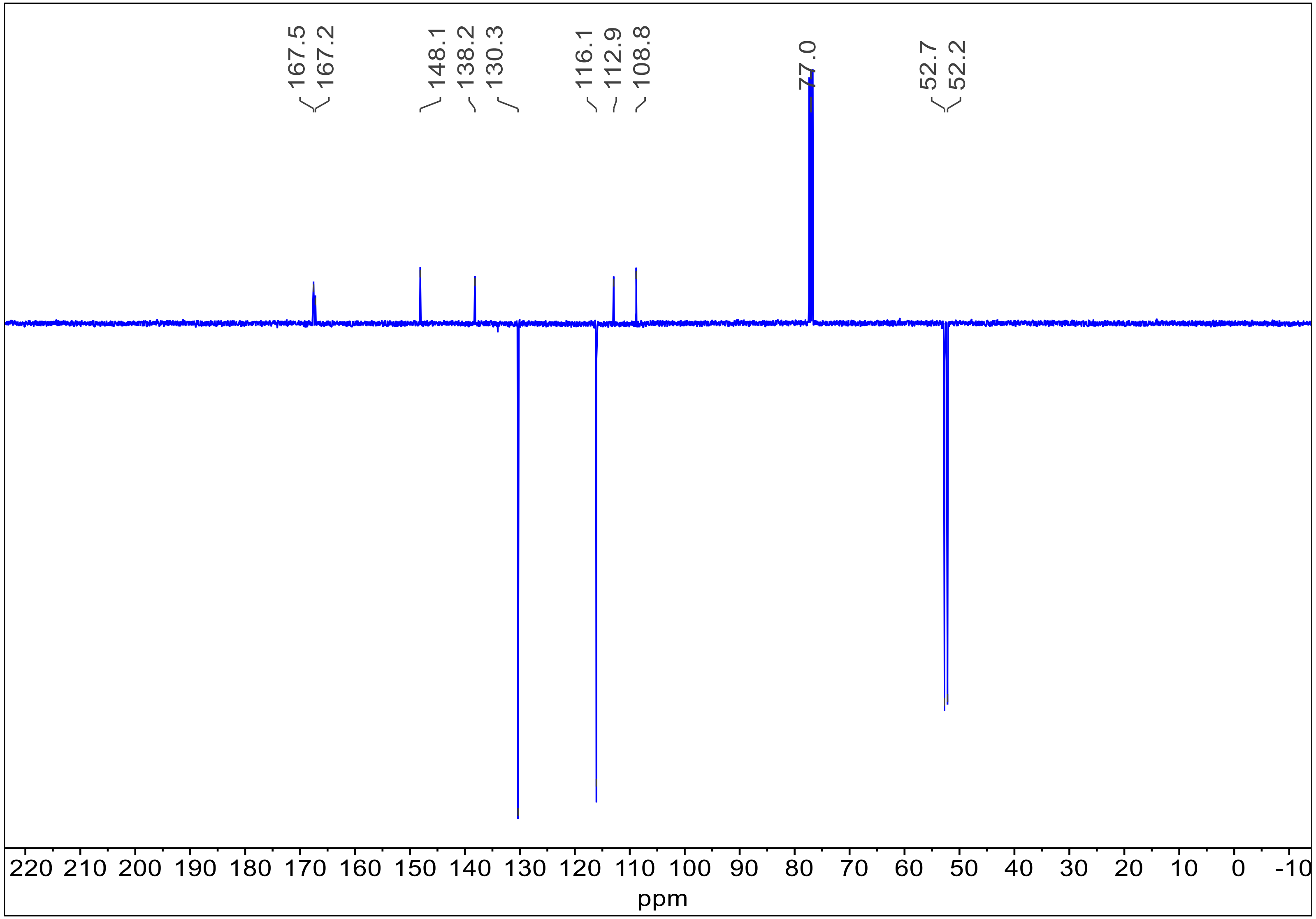

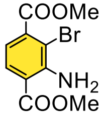

^{1}H – ^{1}H COSY ($CDCl_3$): Dimethyl 2-amino-3-bromoterephthalate (**S4**)

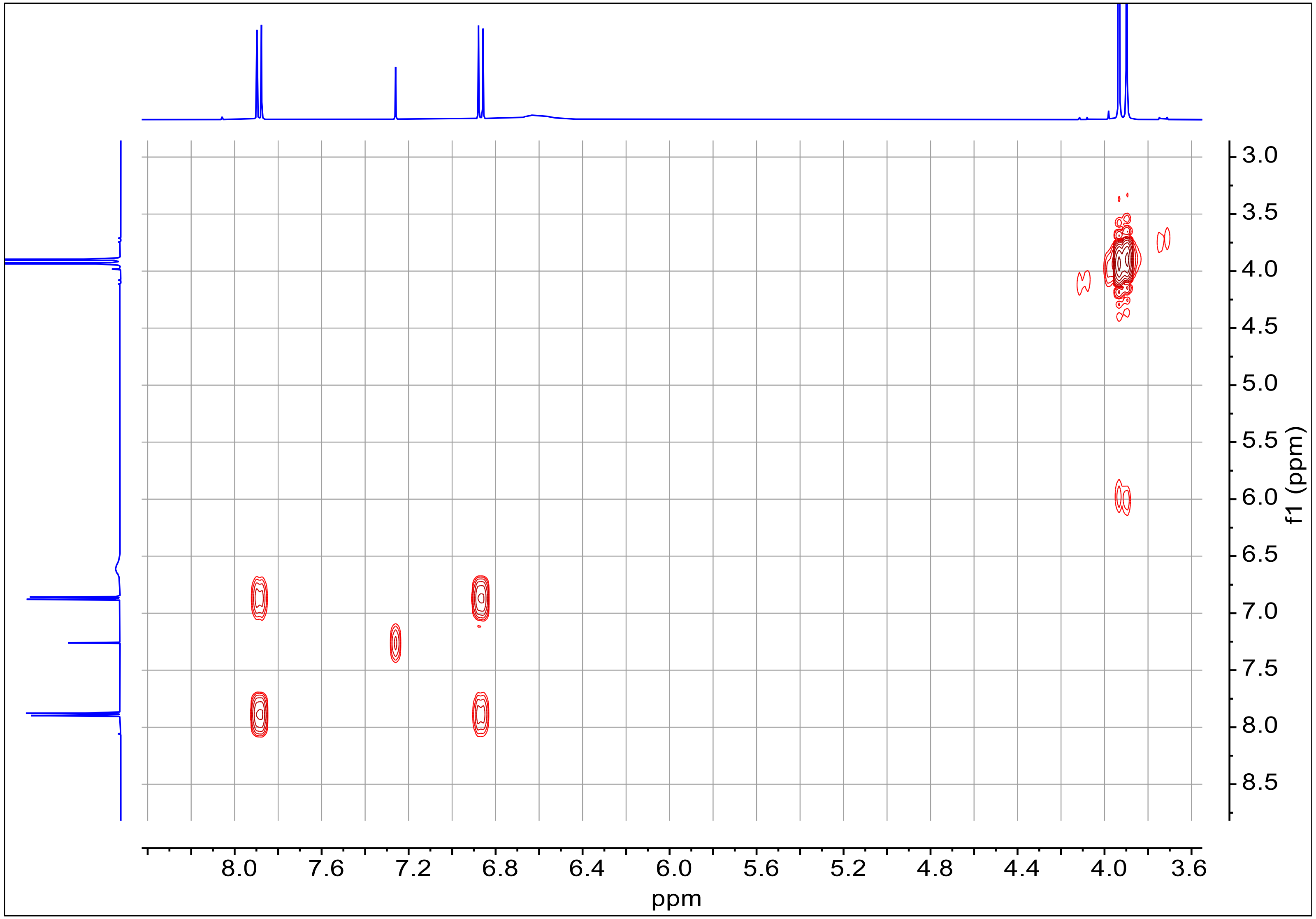

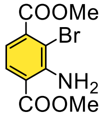

HSQC ($CDCl_3$): Dimethyl 2-amino-3-bromoterephthalate (**S4**)

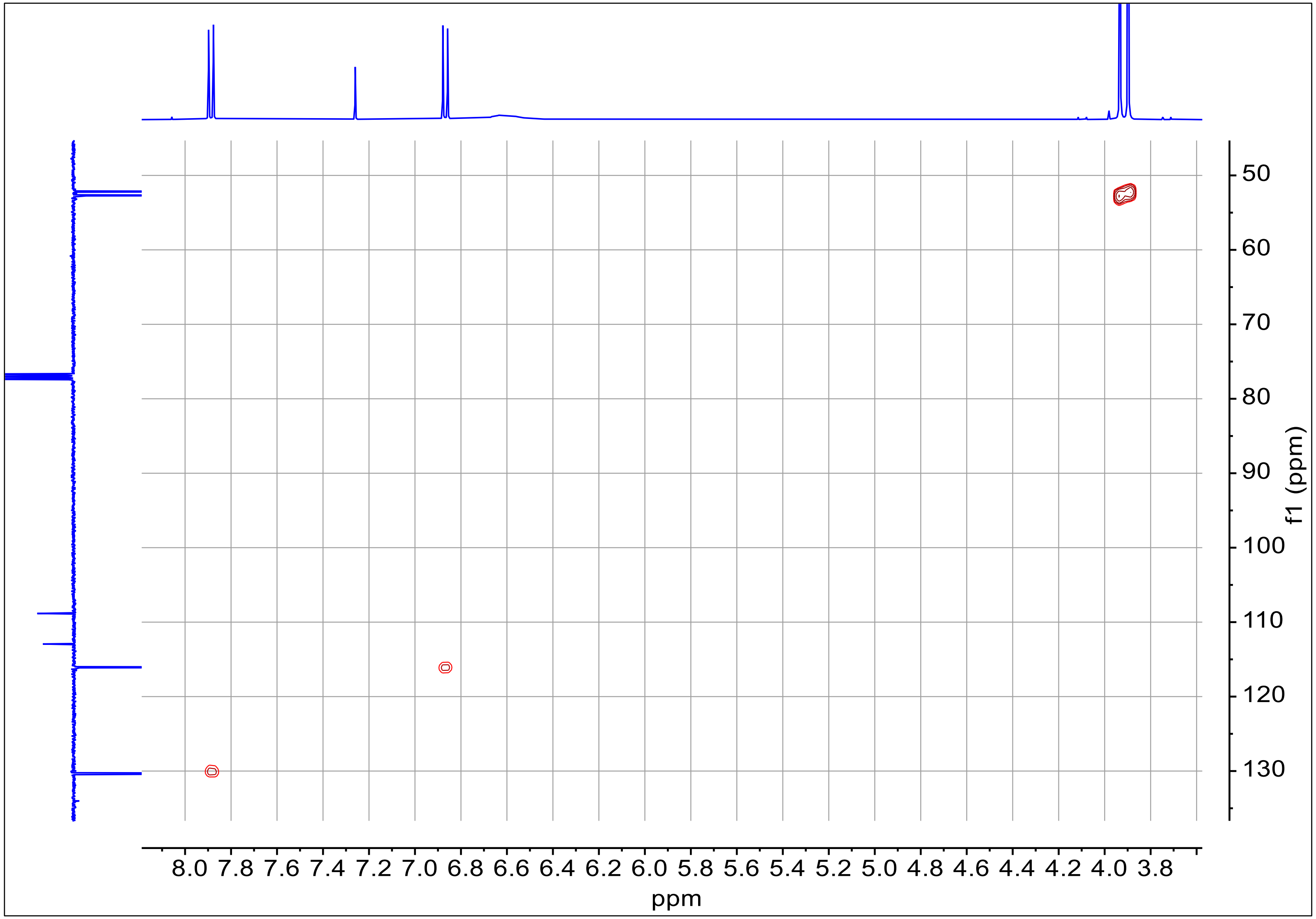

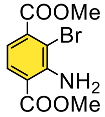

HMBC ($CDCl_3$): Dimethyl 2-amino-3-bromoterephthalate (**S4**)

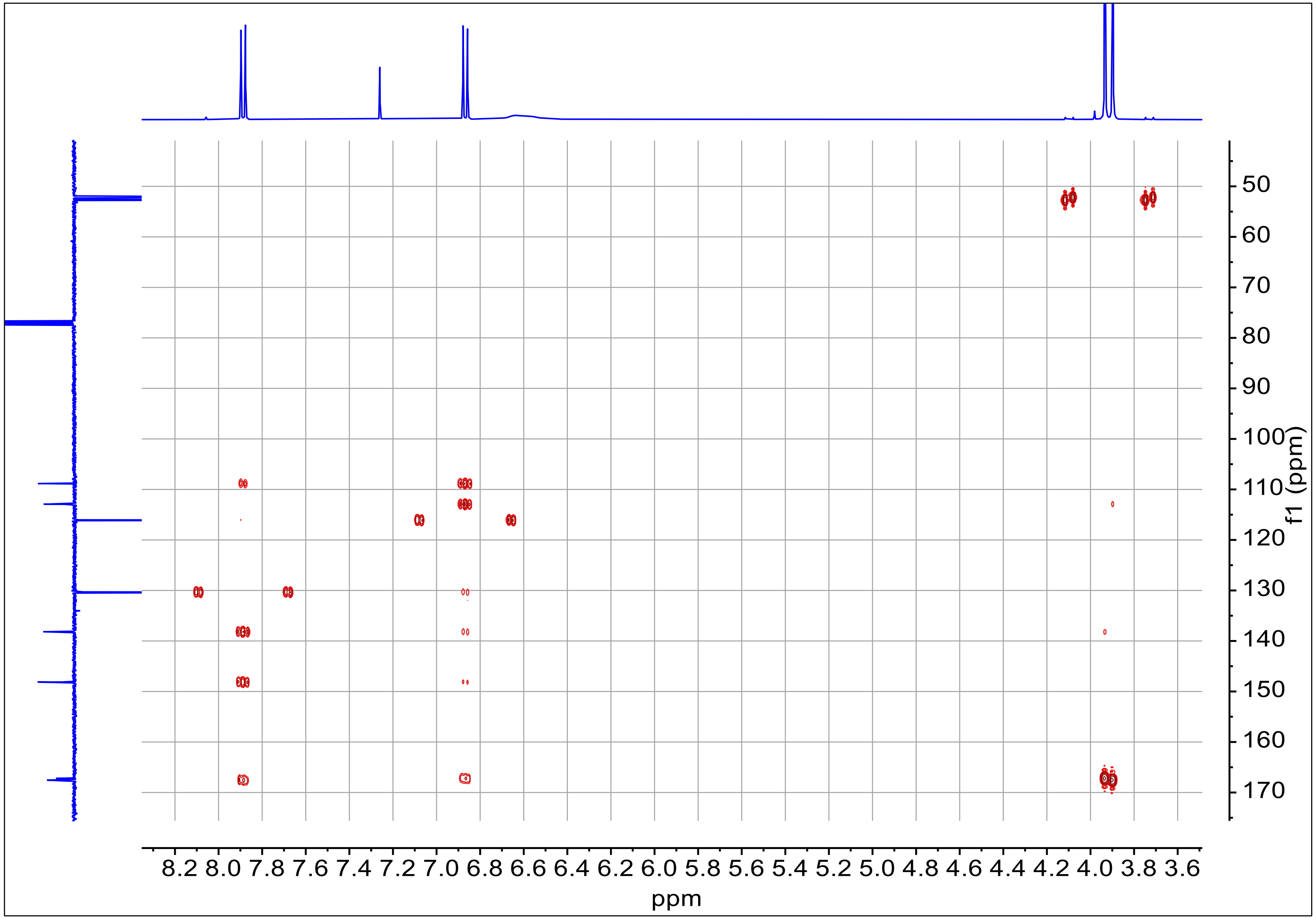

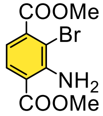

^{1}H NMR (400 MHz, $CDCl_3$): Dimethyl 2-amino-3,5-dibromoterephthalate (**S5**)

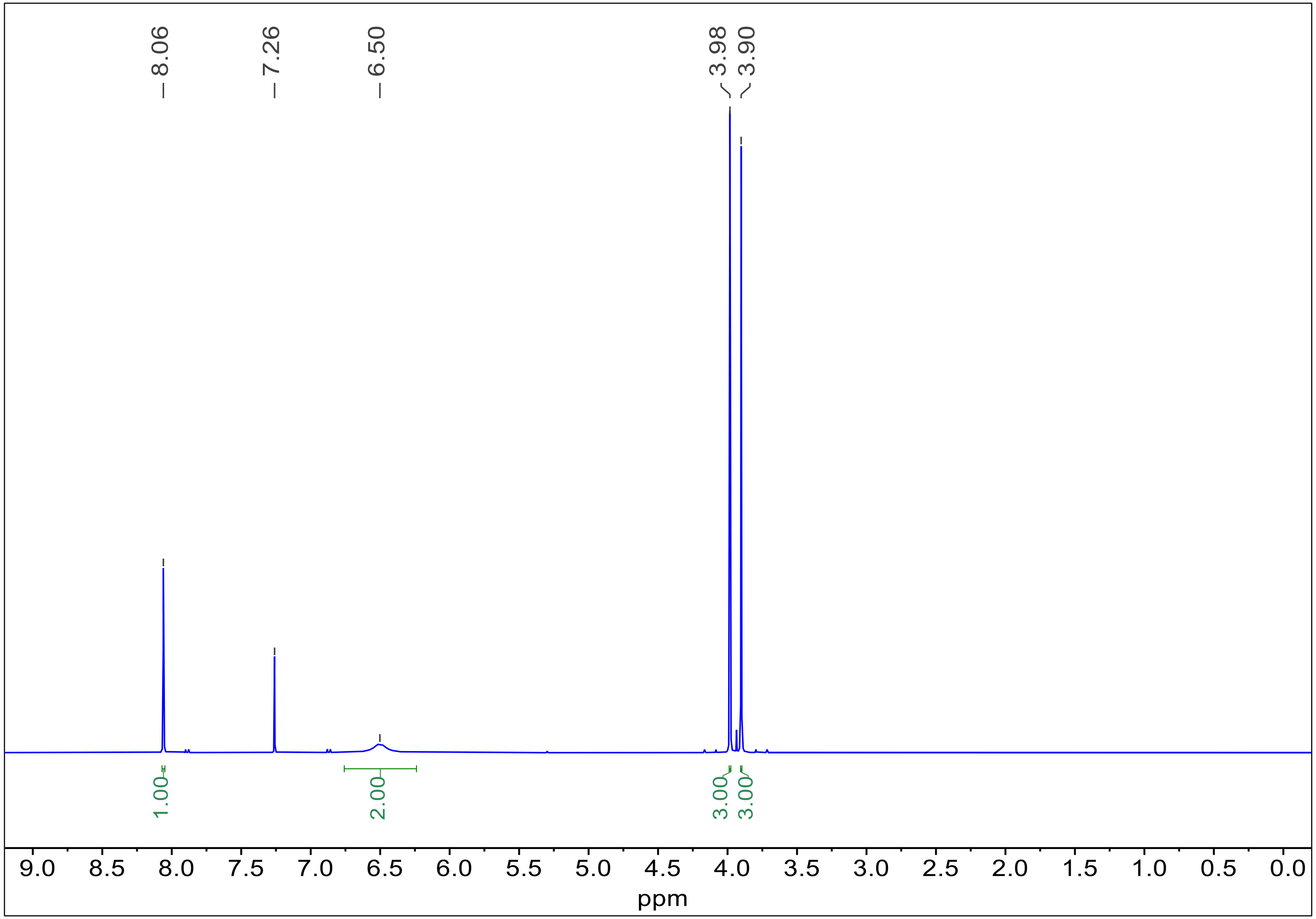

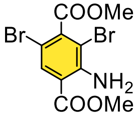

$^{13}C\ \{^{1}H\}$ APT NMR (100 MHz, $CDCl_3$): Dimethyl 2-amino-3,5-dibromoterephthalate (**S5**)

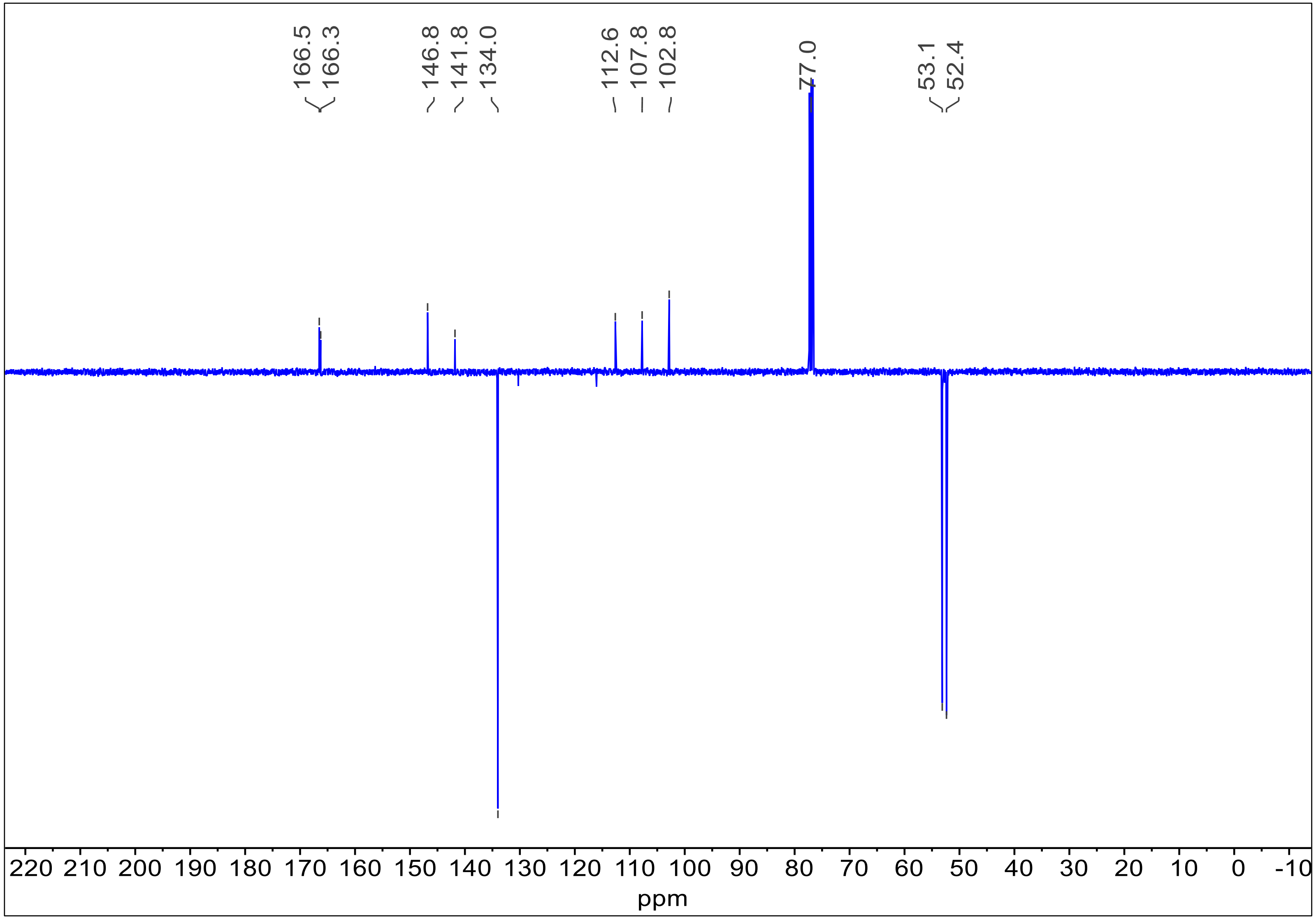

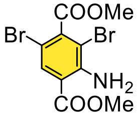

^{1}H – ^{1}H COSY ($CDCl_3$): Dimethyl 2-amino-3,5-dibromoterephthalate (**S5**)

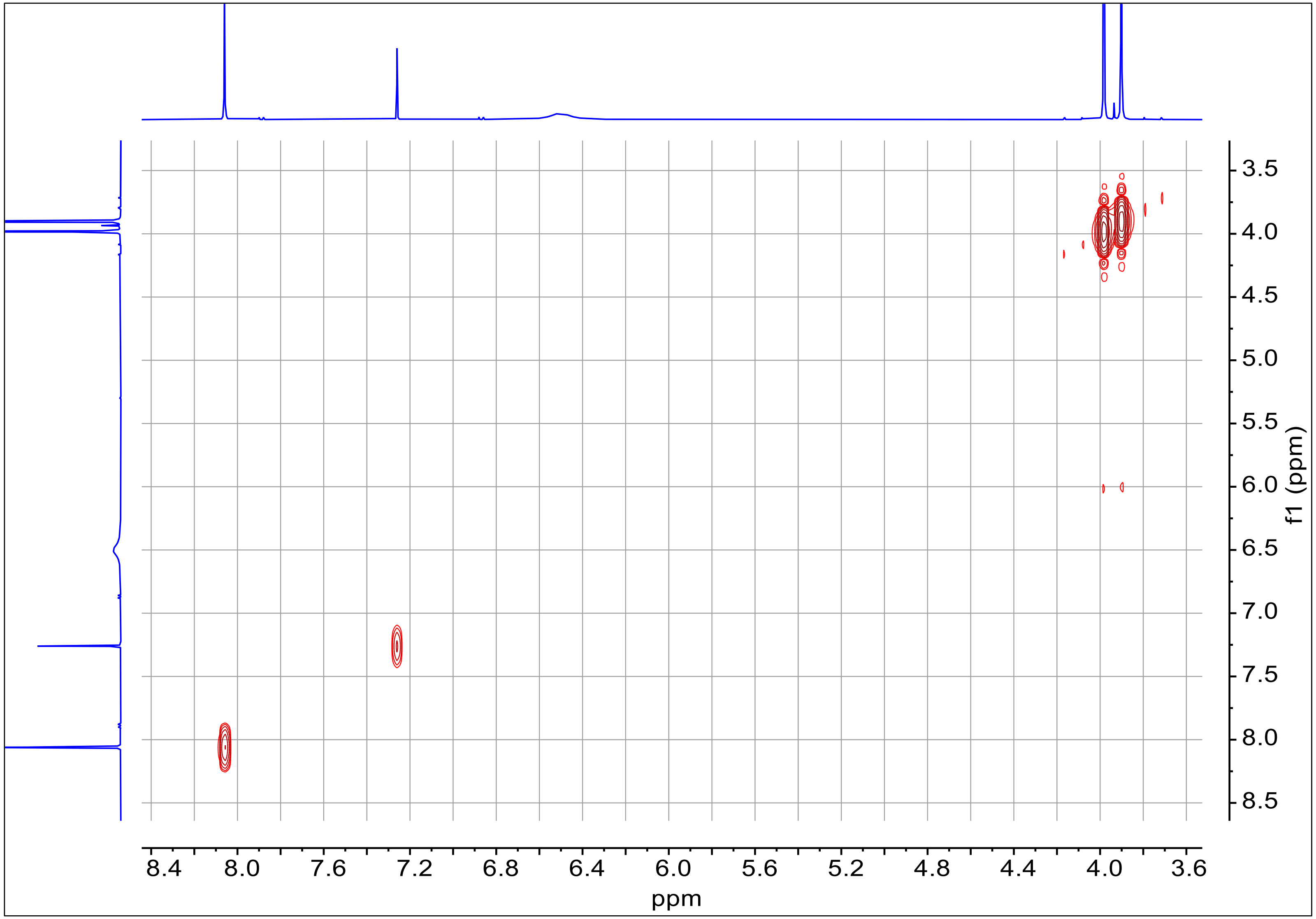

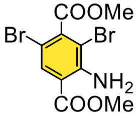

HSQC ($CDCl_3$): Dimethyl 2-amino-3,5-dibromoterephthalate (**S5**)

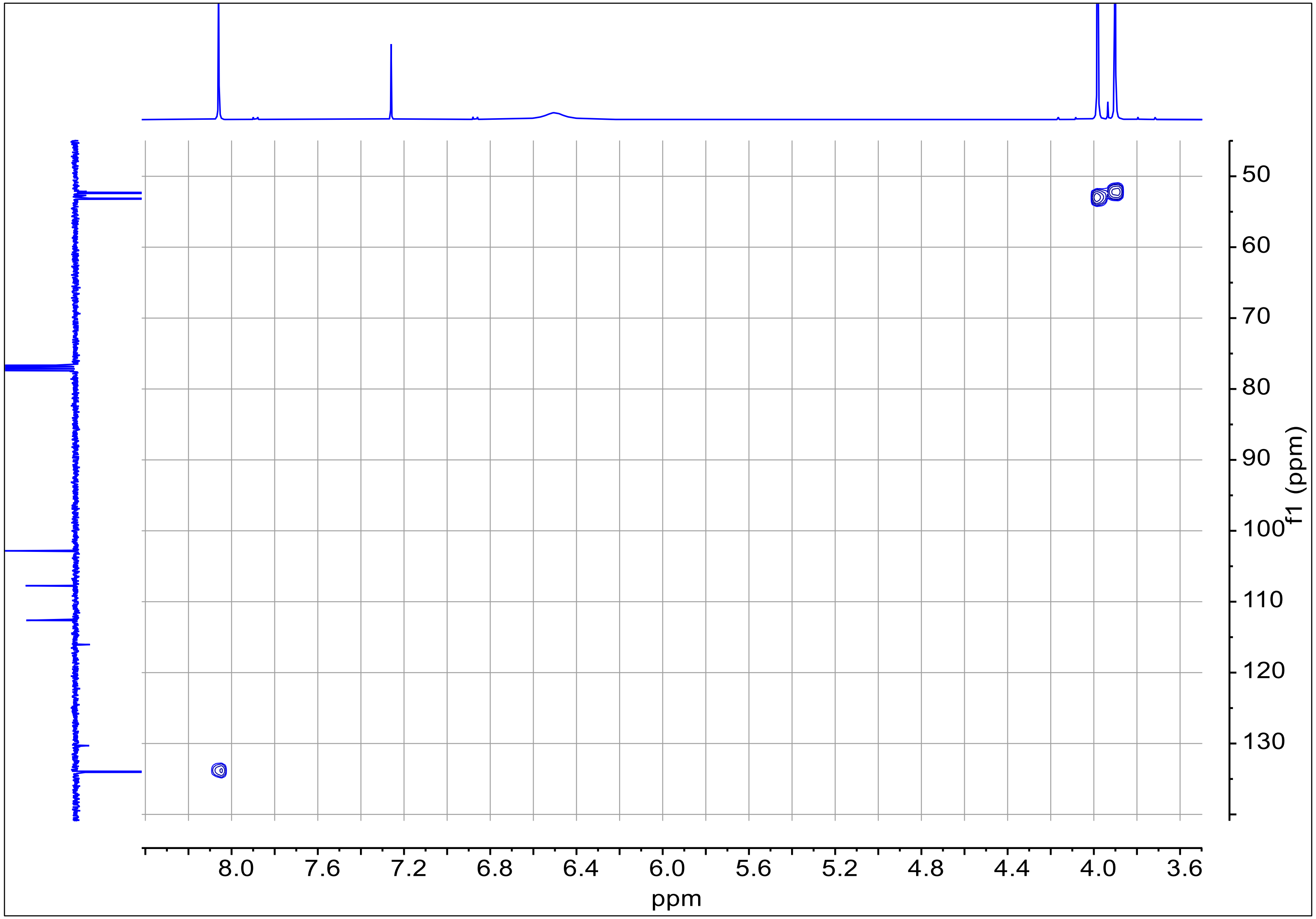

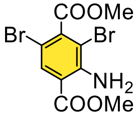

HMBC ($CDCl_3$): Dimethyl 2-amino-3,5-dibromoterephthalate (**S5**)

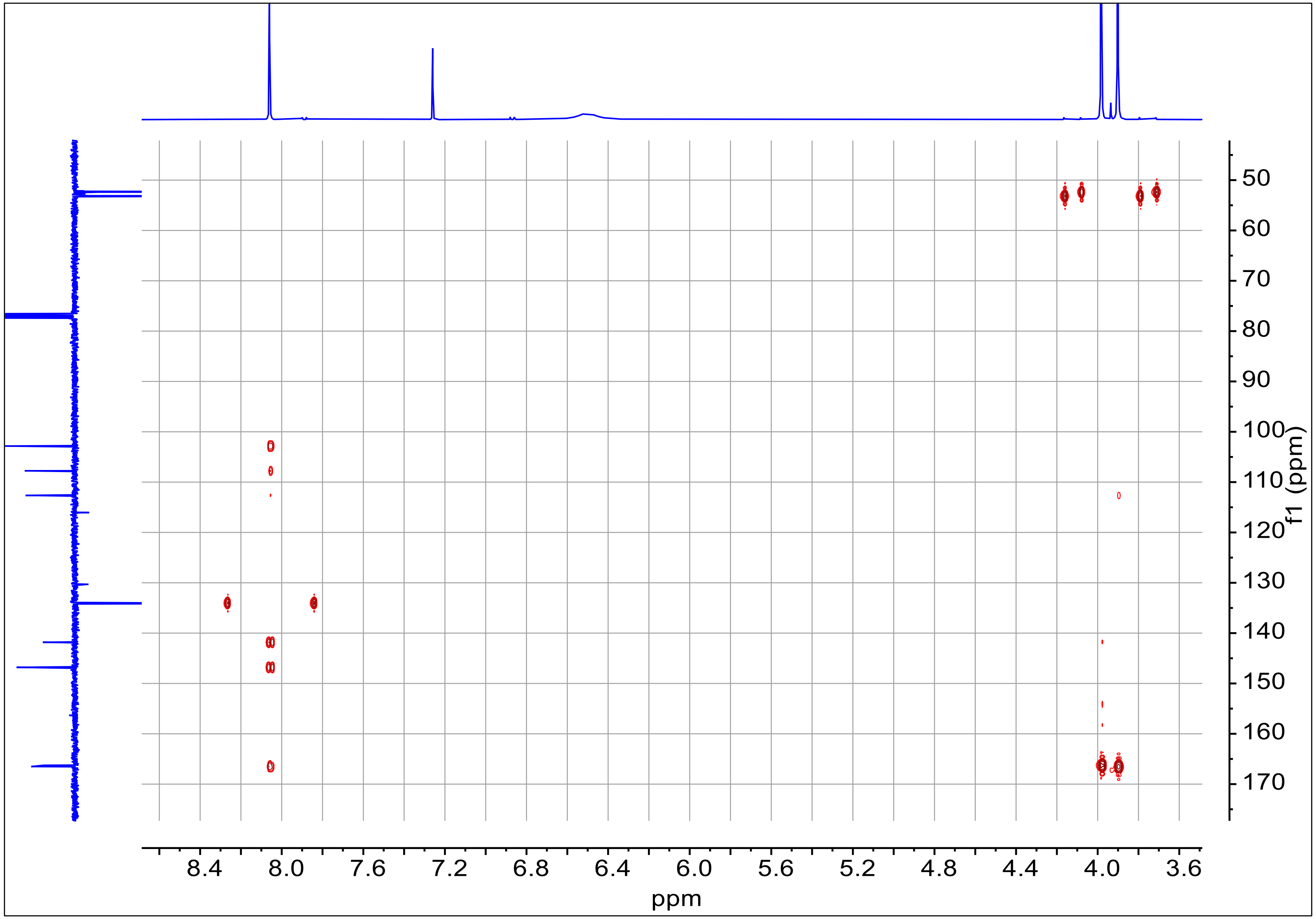

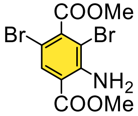

^{1}H NMR (400 MHz, DMSO-d_6): 2-Amino-5-bromo-1,4-benzenedicarboxylic acid (**S6**)

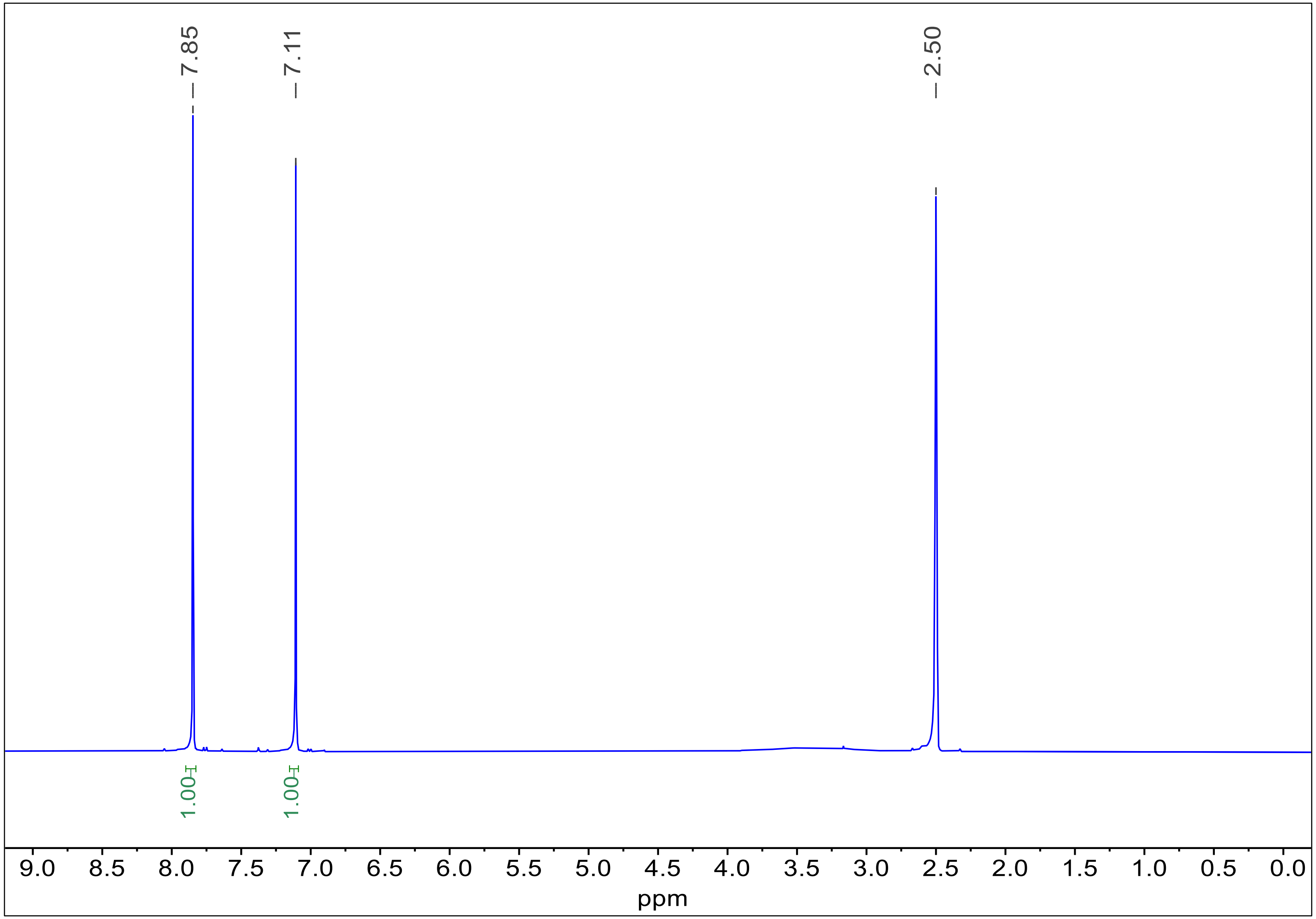

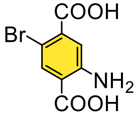

$^{13}C\{^1H\}$ NMR (100 MHz, DMSO-d_6): 2-Amino-5-bromo-1,4-benzenedicarboxylic acid (**S6**)

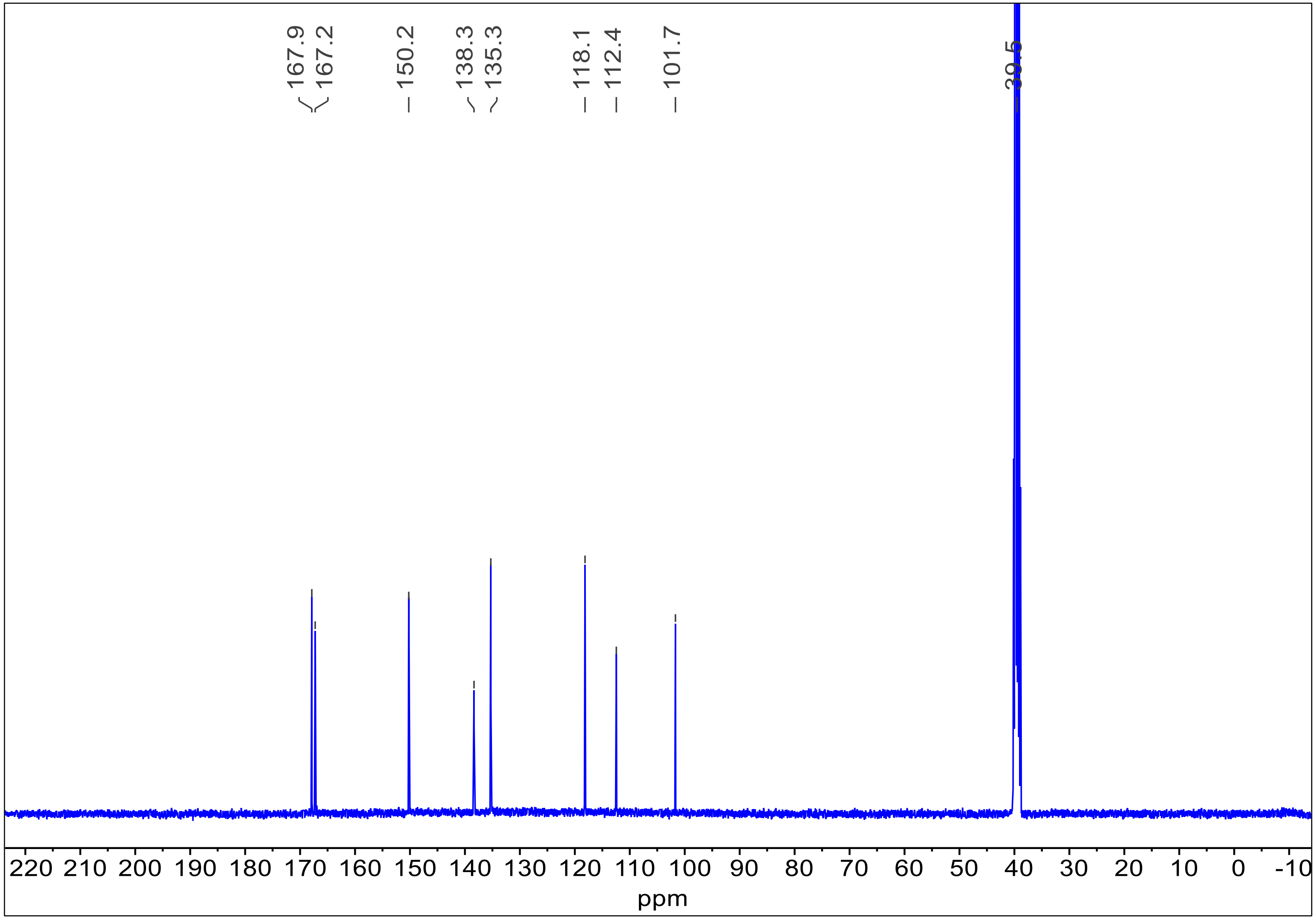

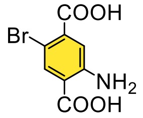

^{13}C {^{1}H} APT NMR (100 MHz, DMSO-d_6): 2-Amino-5-bromo-1,4-benzenedicarboxylic acid (**S6**)

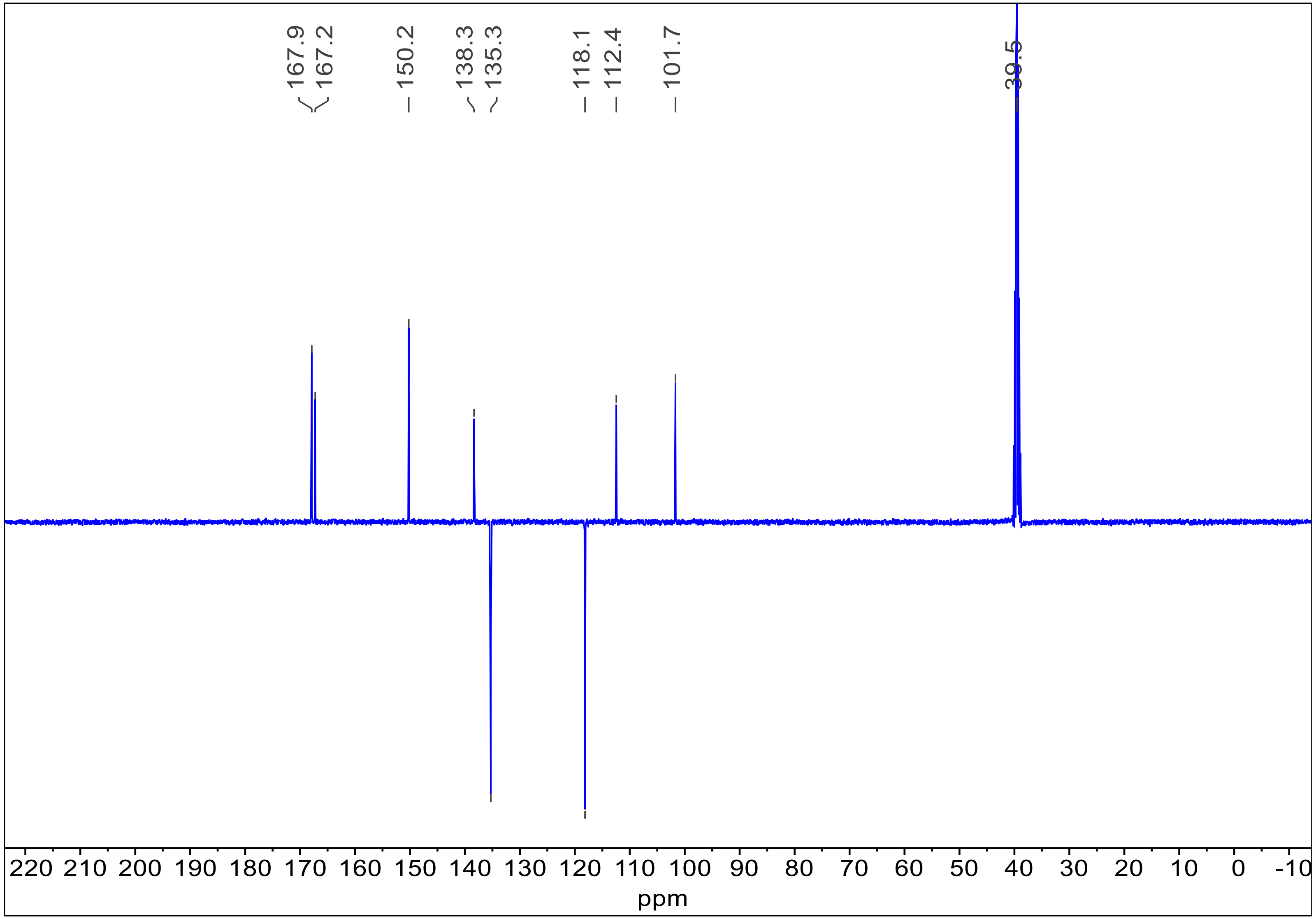

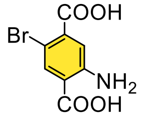

^{1}H – ^{1}H COSY (DMSO-d_6): 2-Amino-5-bromo-1,4-benzenedicarboxylic acid (**S6**)

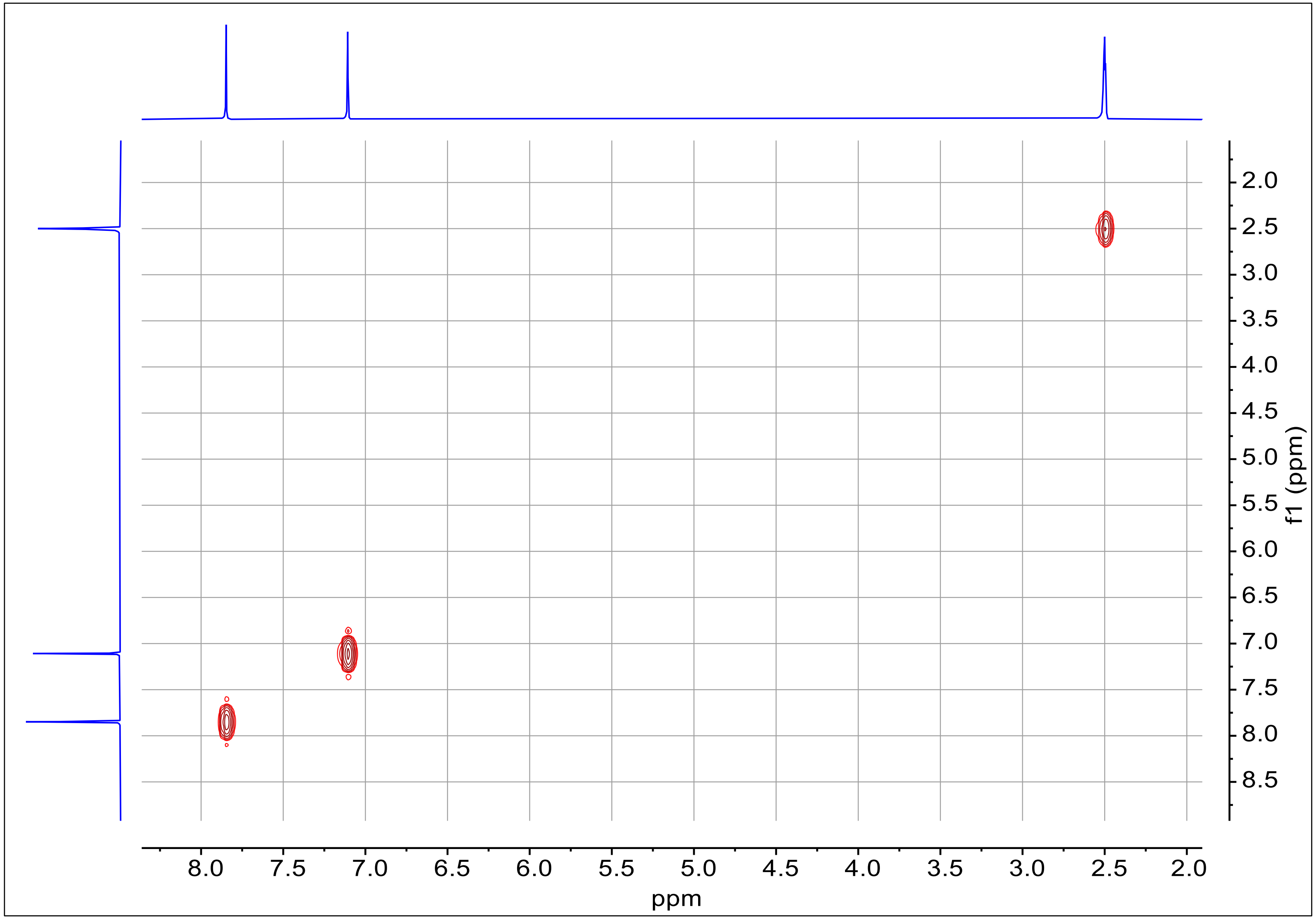

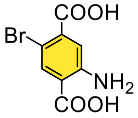

HSQC (DMSO-d_6): 2-Amino-5-bromo-1,4-benzenedicarboxylic acid (**S6**)

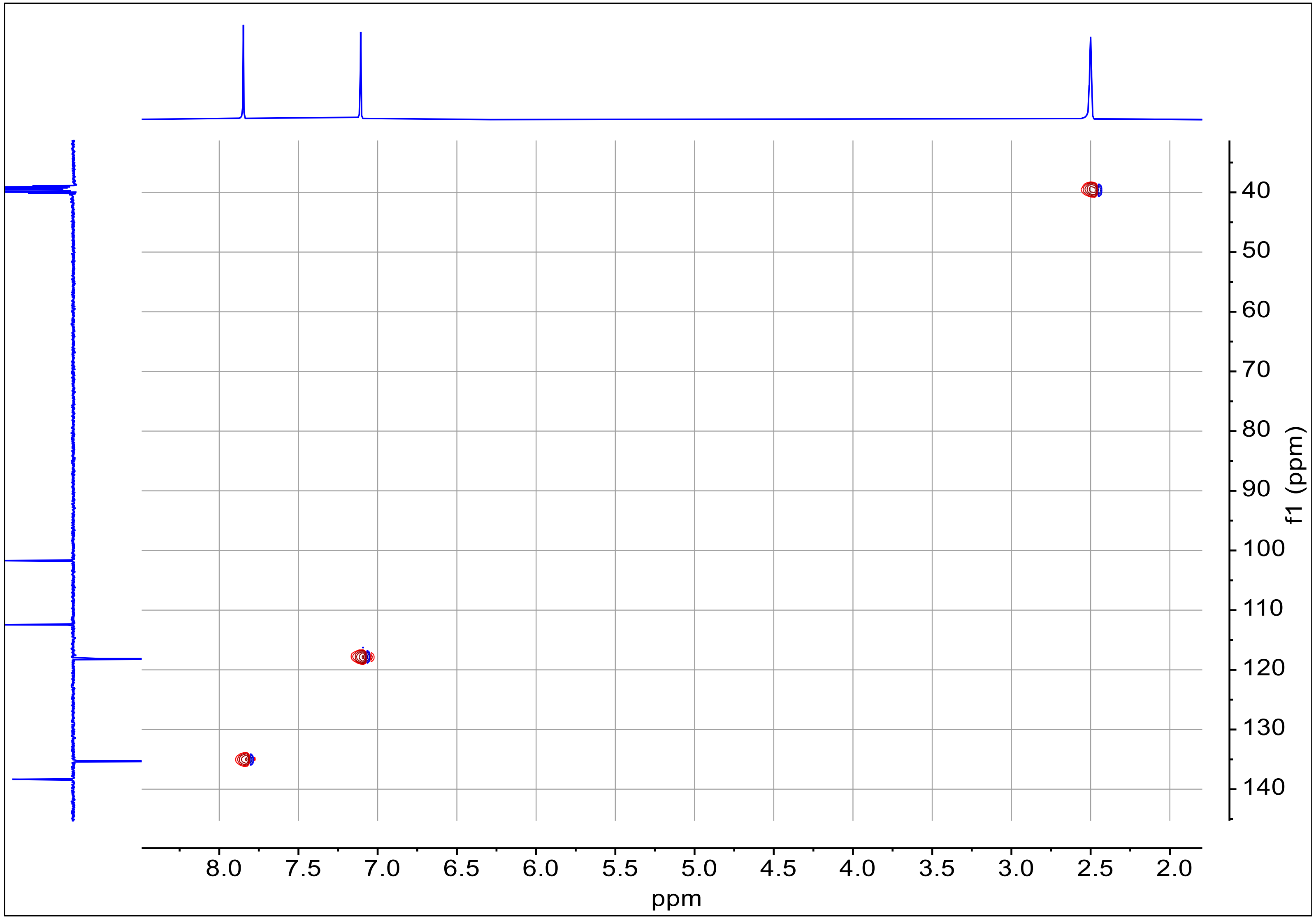

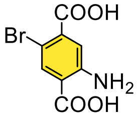

HMBC (DMSO-d_6): 2-Amino-5-bromo-1,4-benzenedicarboxylic acid (**S6**)

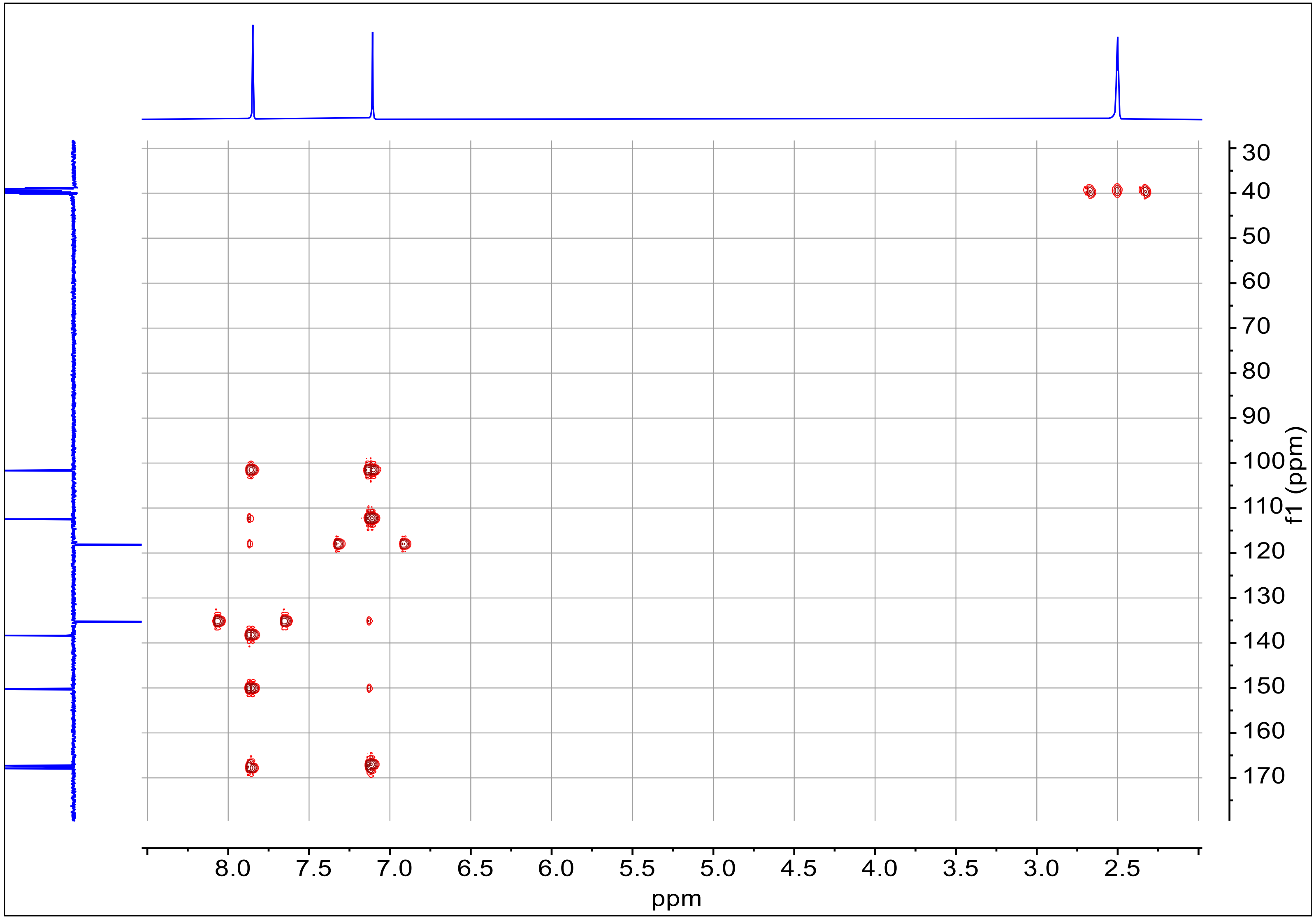

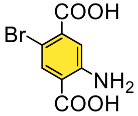

18. X-Ray Crystallographic Data

X-ray: Compound **1C** (displacement ellipsoids are shown at the 50% probability level, disorder omitted for clarity)

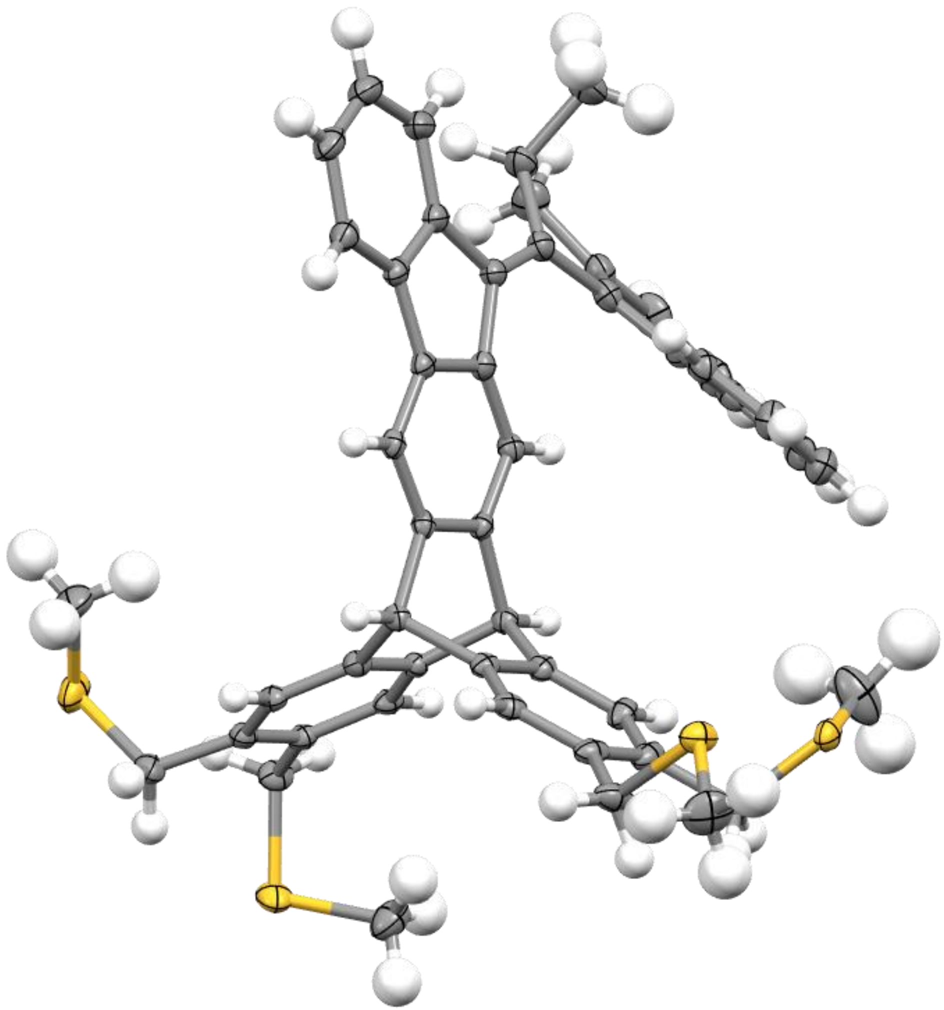

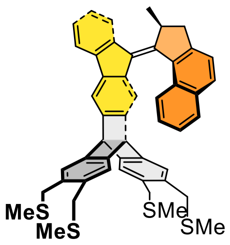

X-ray: Crystal packing of compound **1C** (hydrogen atoms are omitted for clarity)

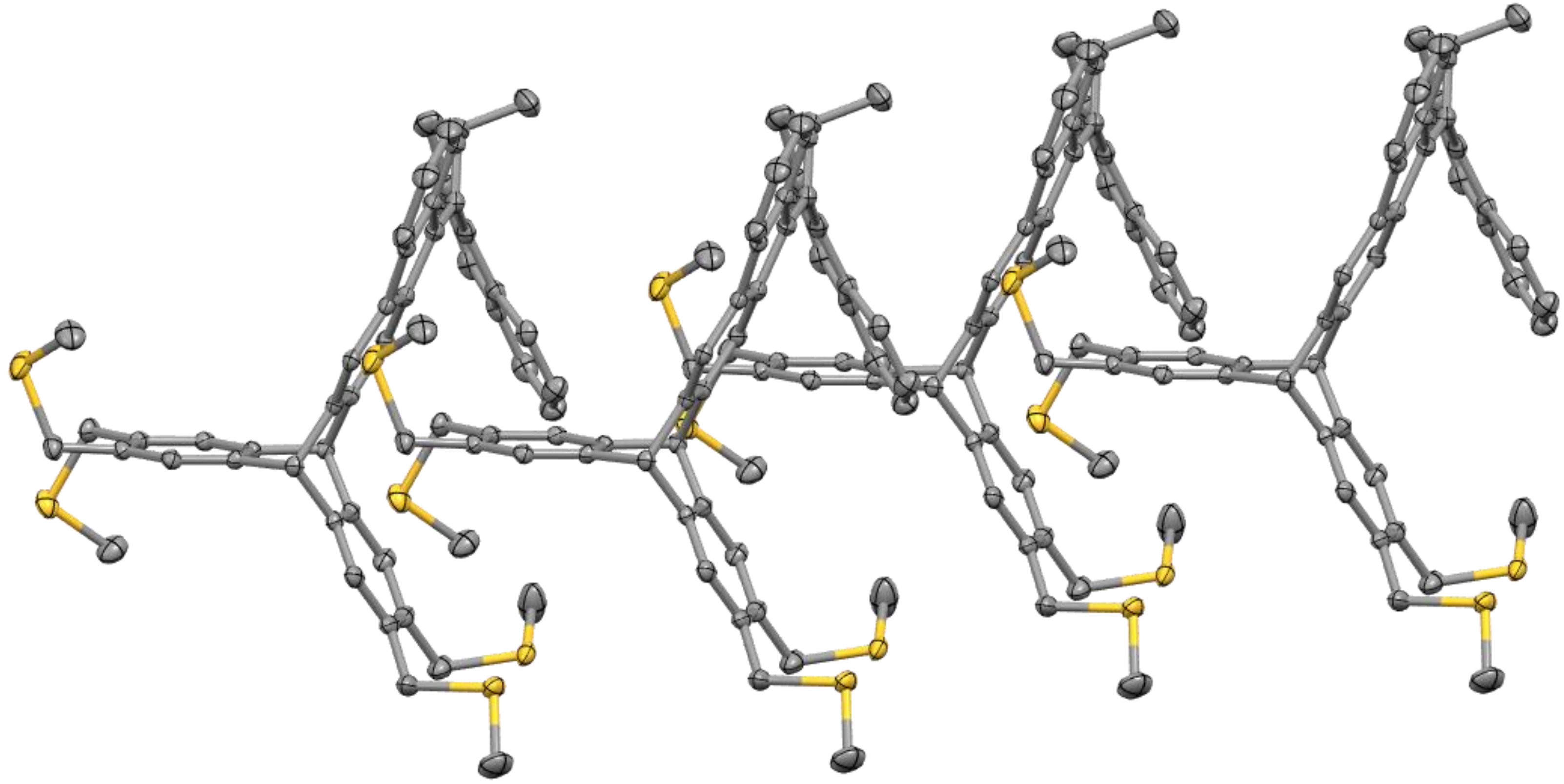

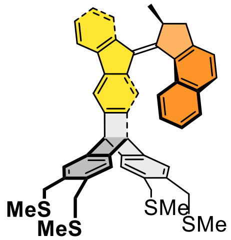

X-ray: Compound **4** (displacement ellipsoids are shown at the 50% probability level)

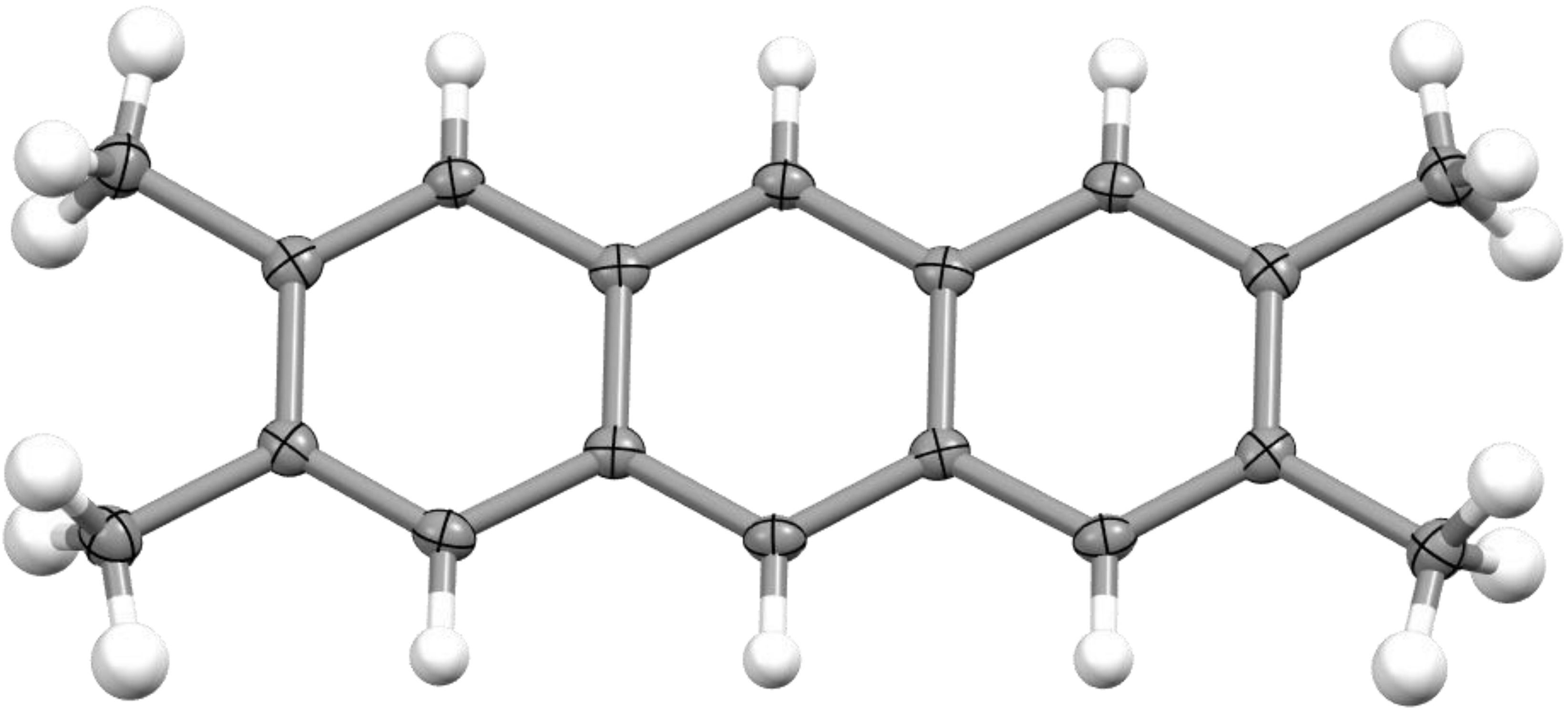

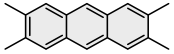

X-ray: Crystal packing of compound **4** (hydrogen atoms are omitted for clarity)

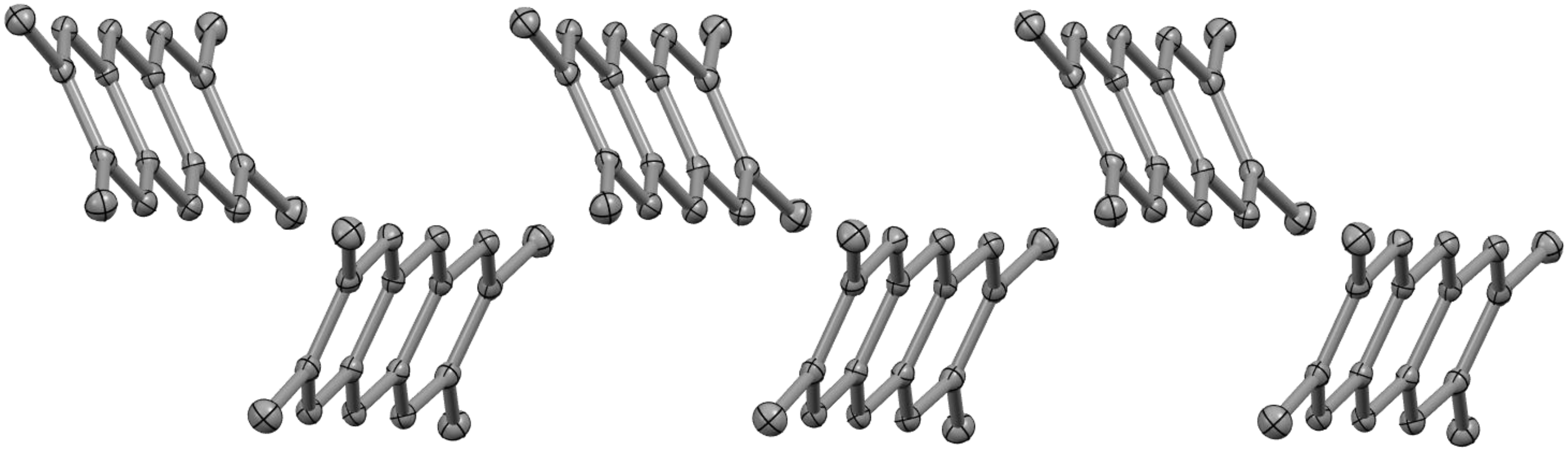

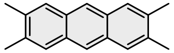

X-ray: Compound **5** (displacement ellipsoids are shown at the 50% probability level)

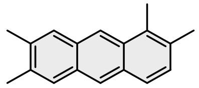

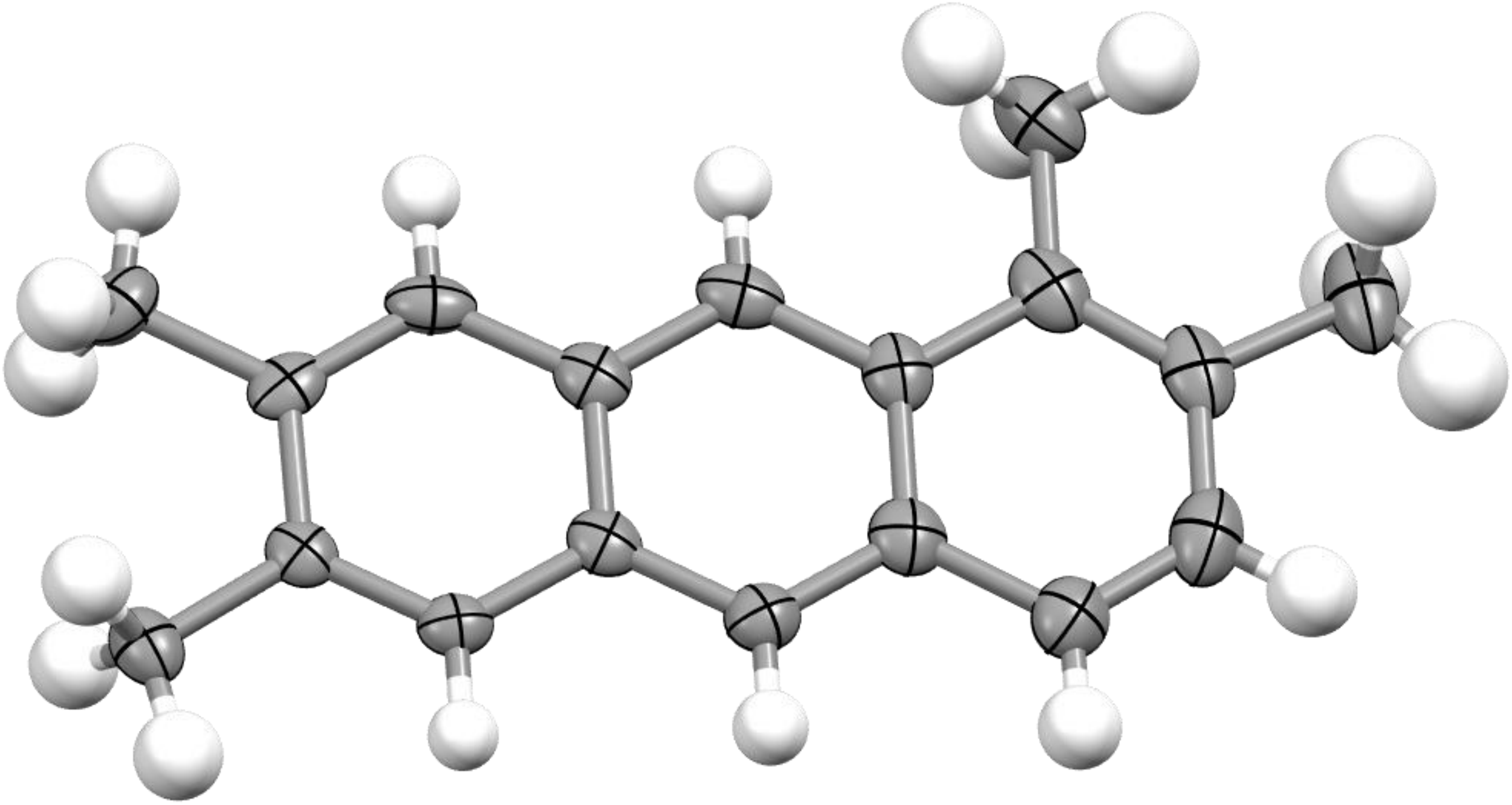

X-ray: Crystal packing of compound 5 (hydrogen atoms are omitted for clarity)

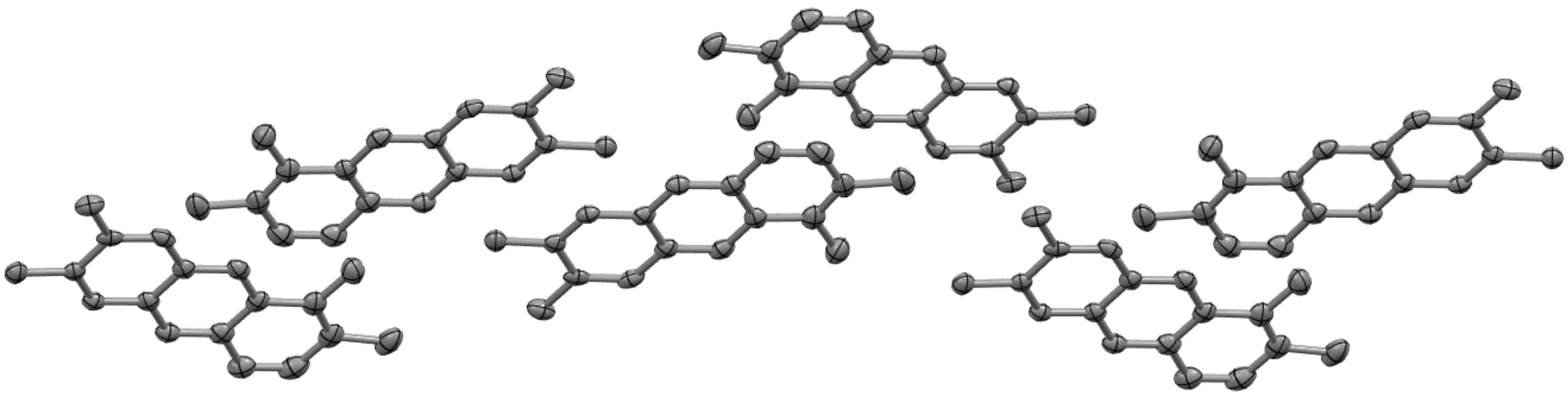

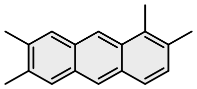

X-ray: Compound **9** (displacement ellipsoids are shown at the 50% probability level, disorder omitted for clarity)

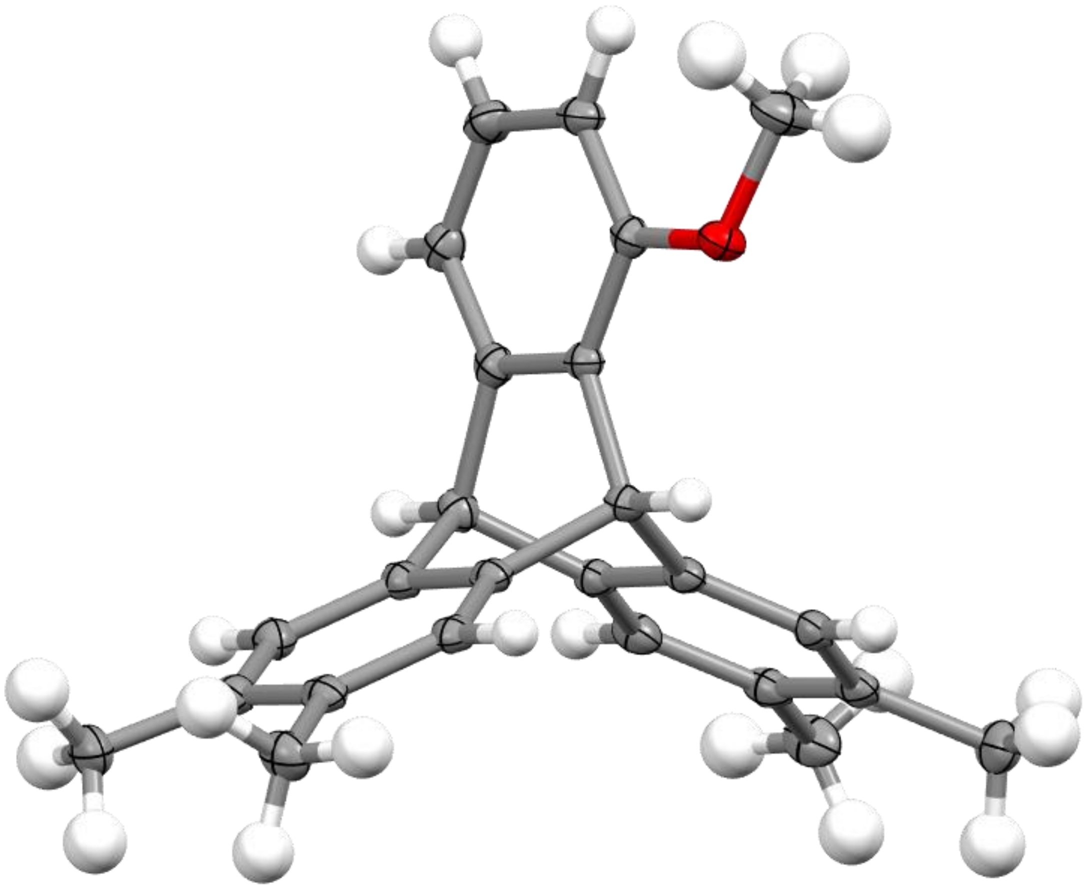

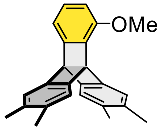

X-ray: Crystal packing of compound **9** (hydrogen atoms are omitted for clarity)

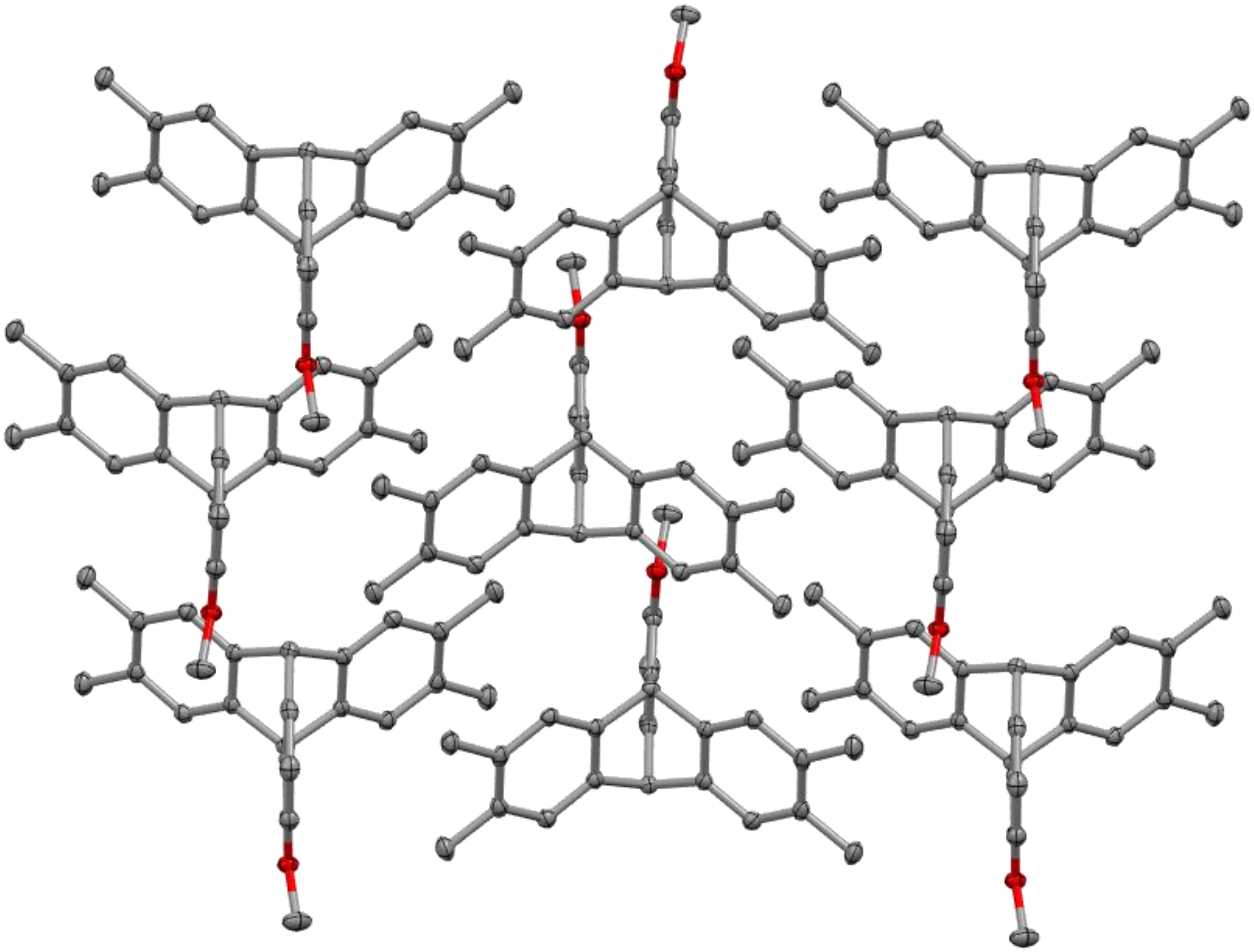

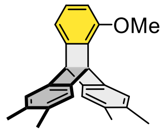

X-ray: Compound **13** (displacement ellipsoids are shown at the 50% probability level)

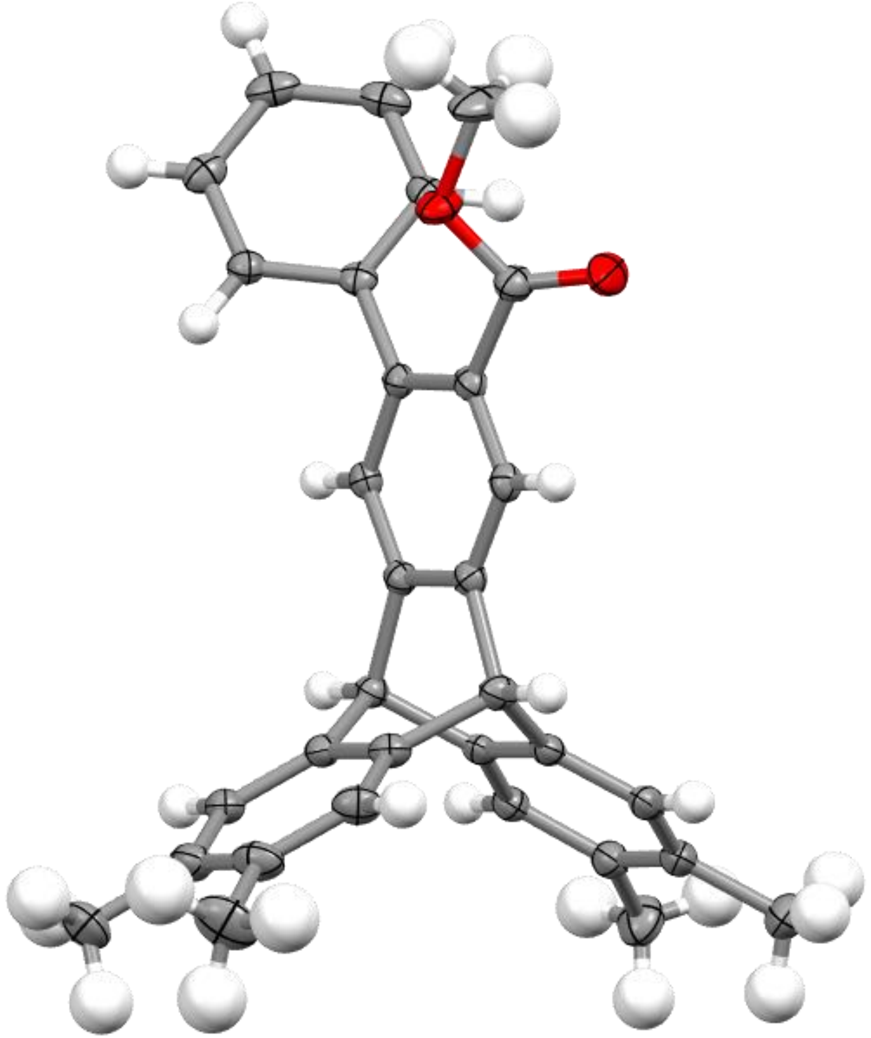

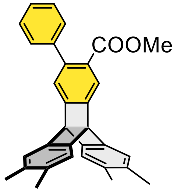

X-ray: Crystal packing of compound **13** (hydrogen atoms are omitted for clarity)

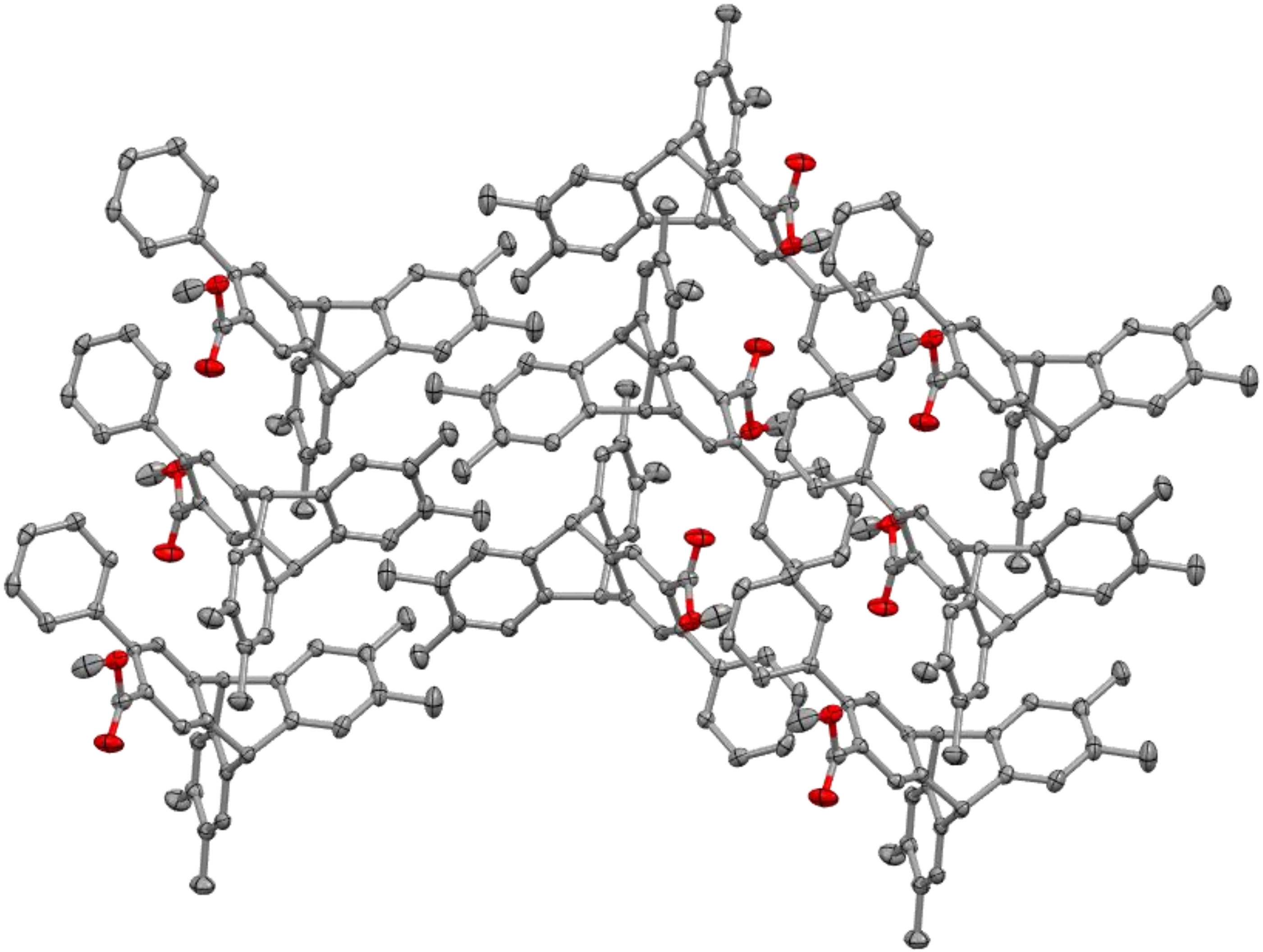

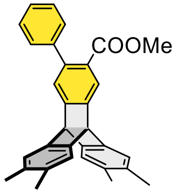

X-ray: Compound **15** (displacement ellipsoids are shown at the 50% probability level)

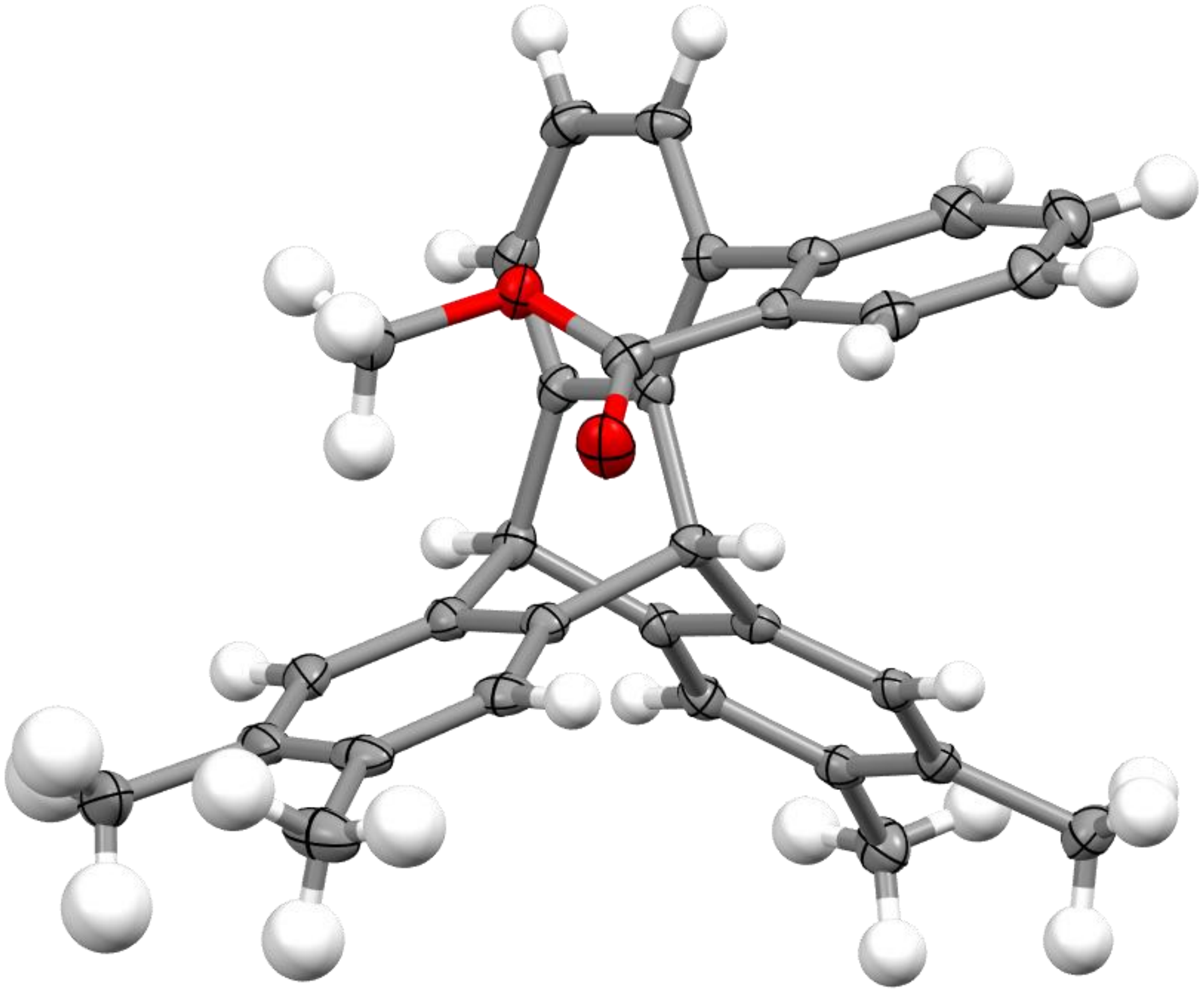

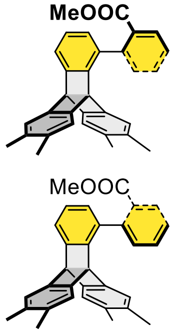

X-ray: Crystal packing of compound **15** (hydrogen atoms are omitted for clarity)

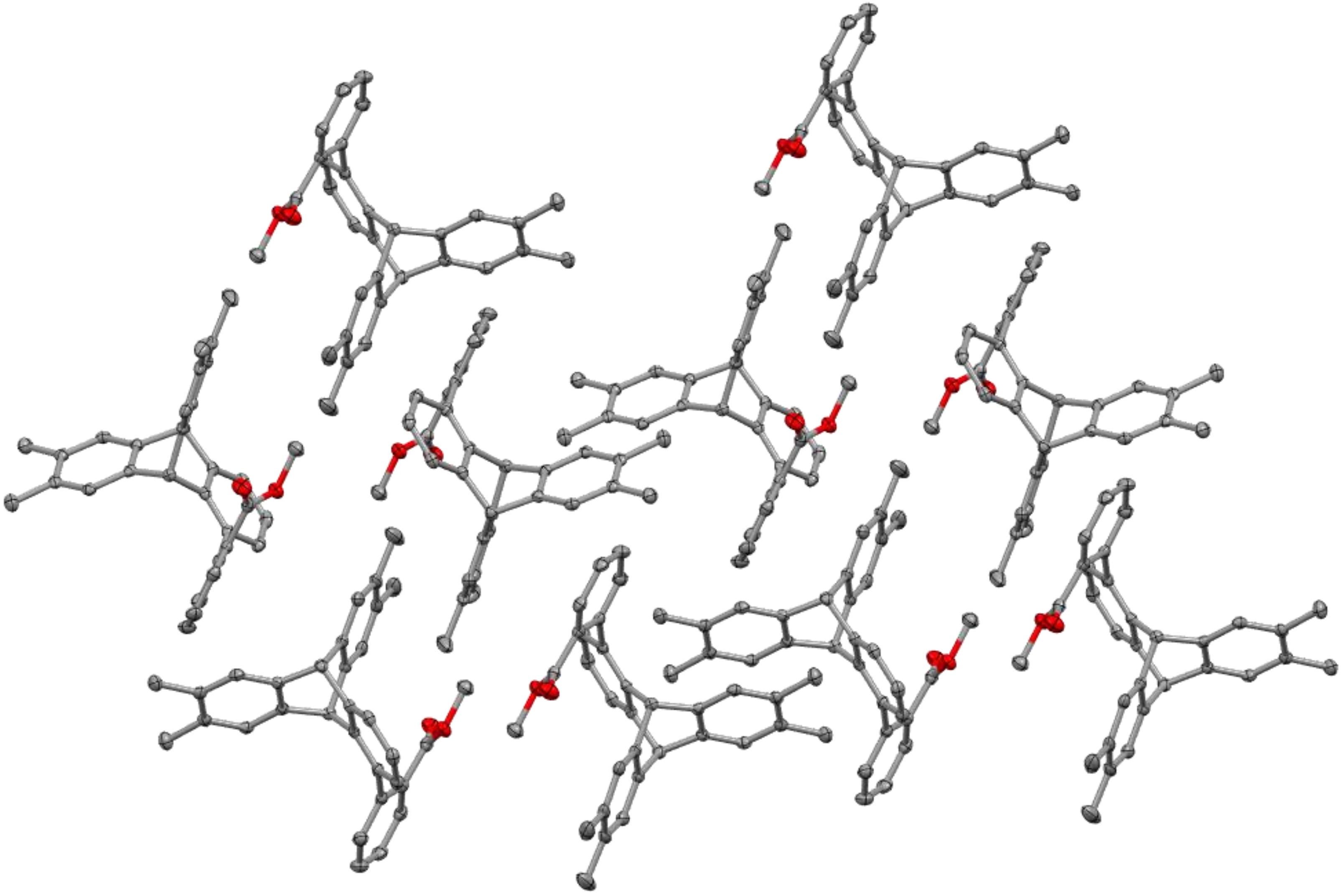

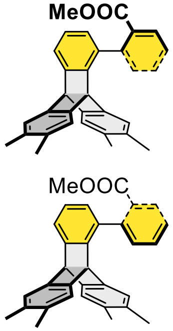

X-ray: Compound **16** (displacement ellipsoids are shown at the 50% probability level, solvent omitted for clarity)

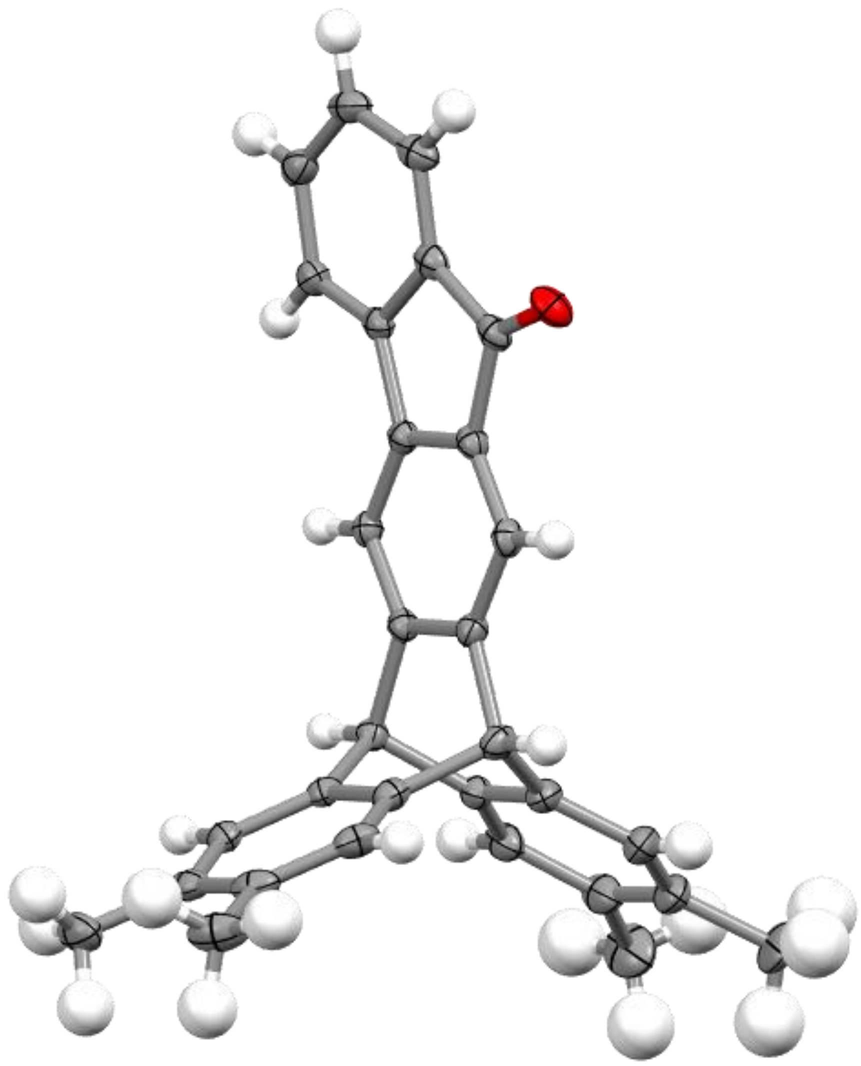

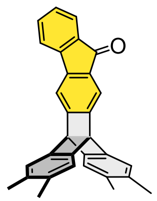

X-ray: Crystal packing of compound **16** (hydrogen atoms are omitted for clarity)

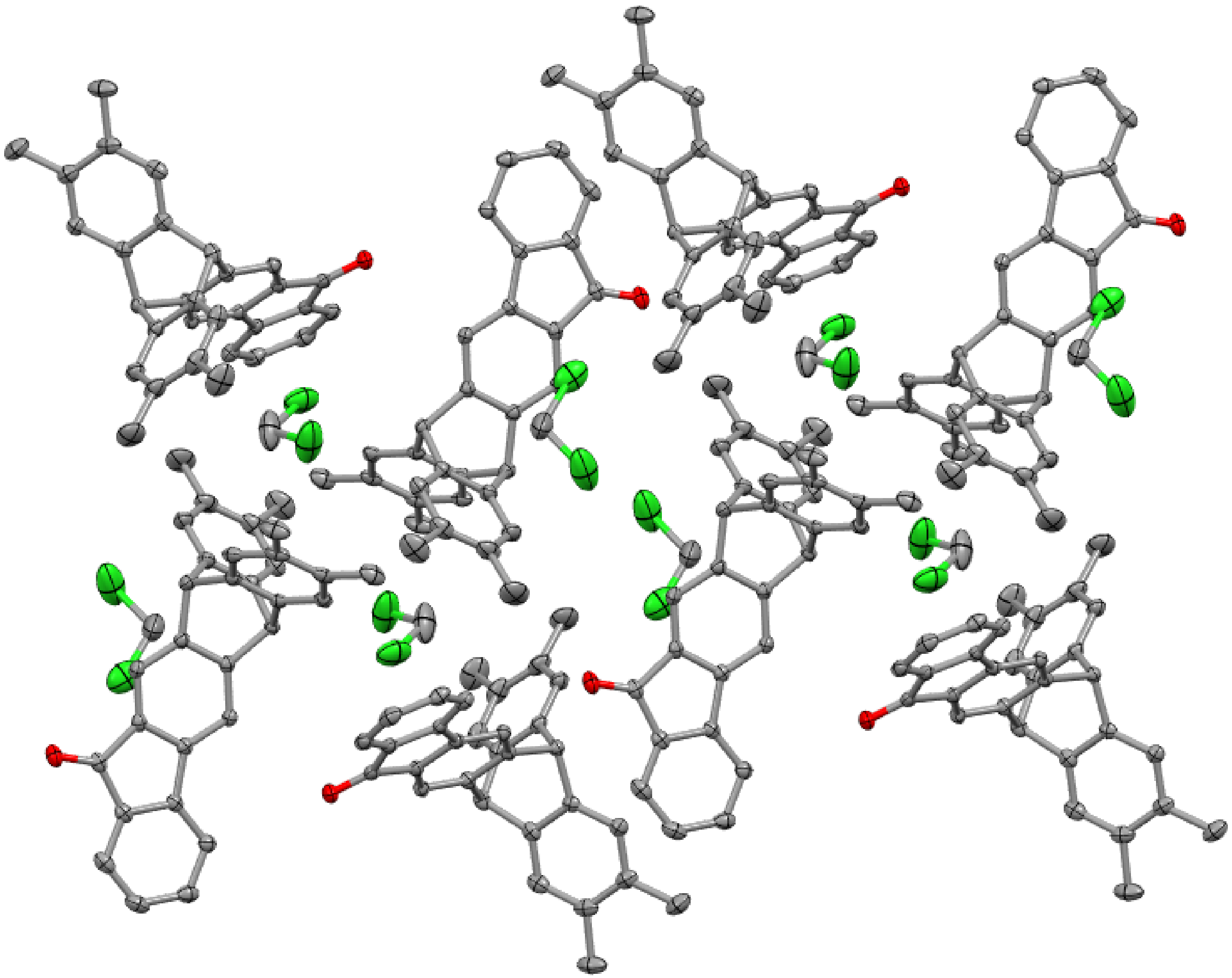

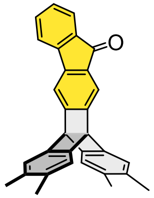

X-ray: Compound **17** (displacement ellipsoids are shown at the 50% probability level, solvent omitted for clarity)

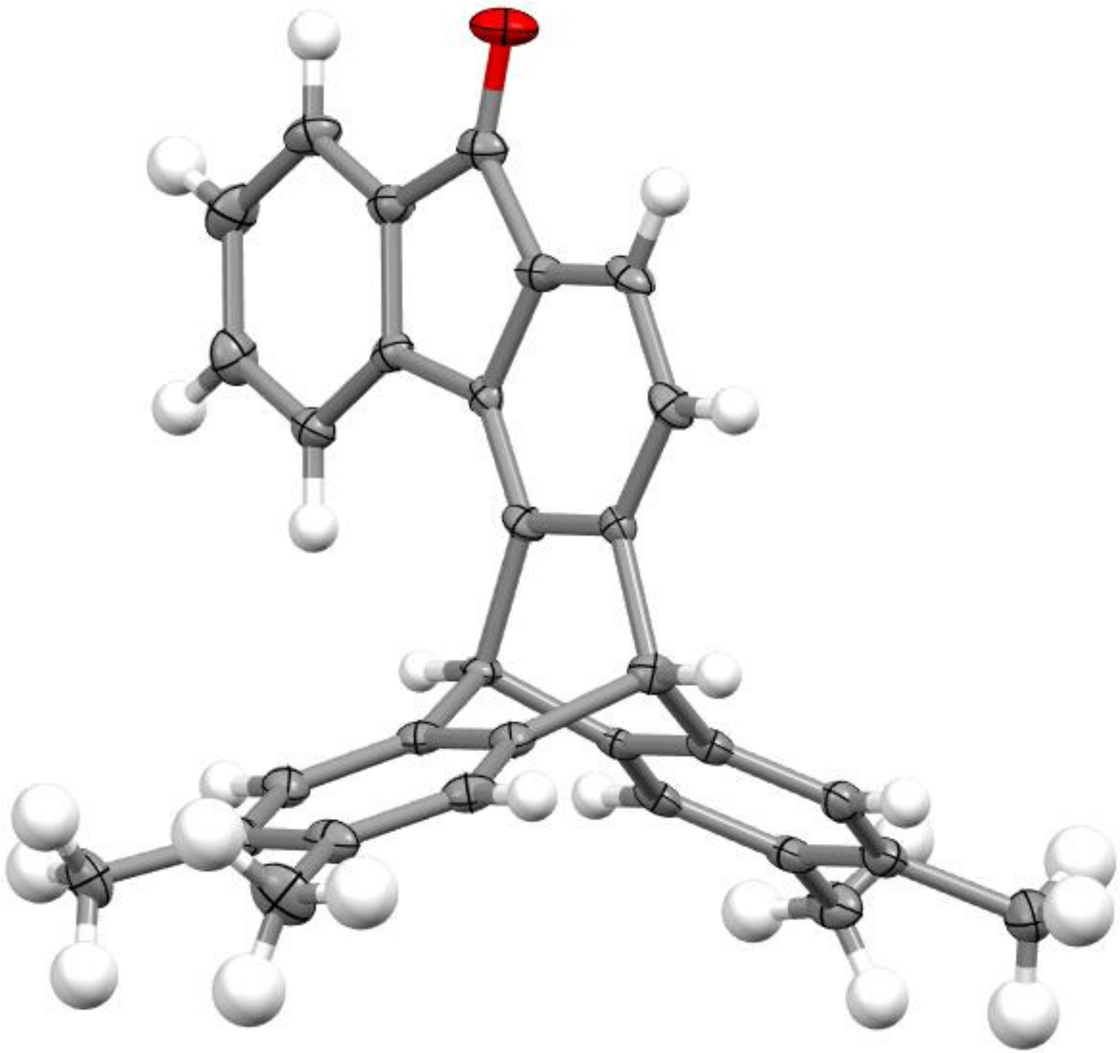

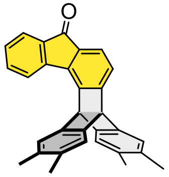

X-ray: Crystal packing of compound **17** (hydrogen atoms are omitted for clarity)

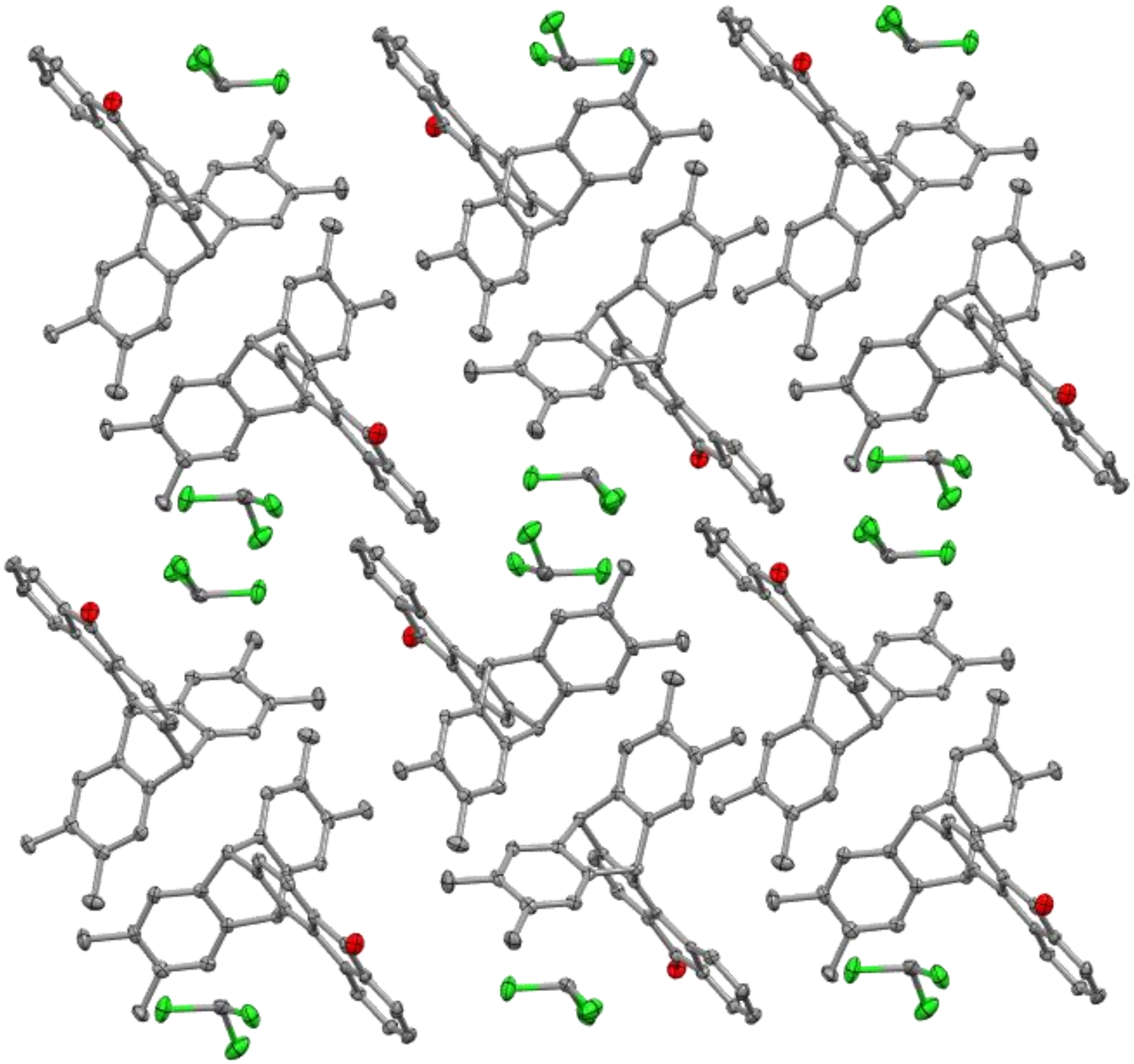

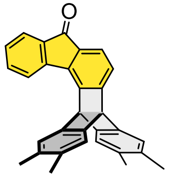

X-ray: Compound **18** (displacement ellipsoids are shown at the 50% probability level, disorder omitted for clarity)

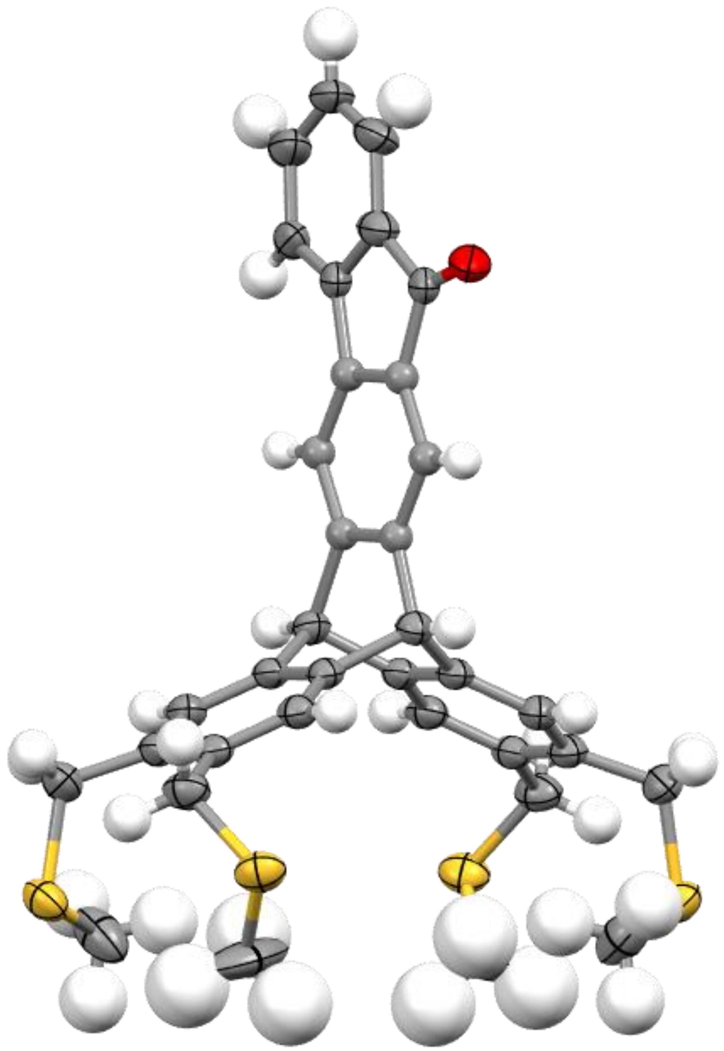

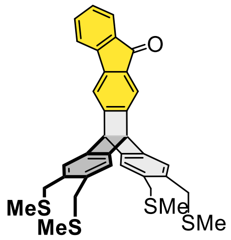

X-ray: Crystal packing of compound **18** (hydrogen atoms are omitted for clarity)

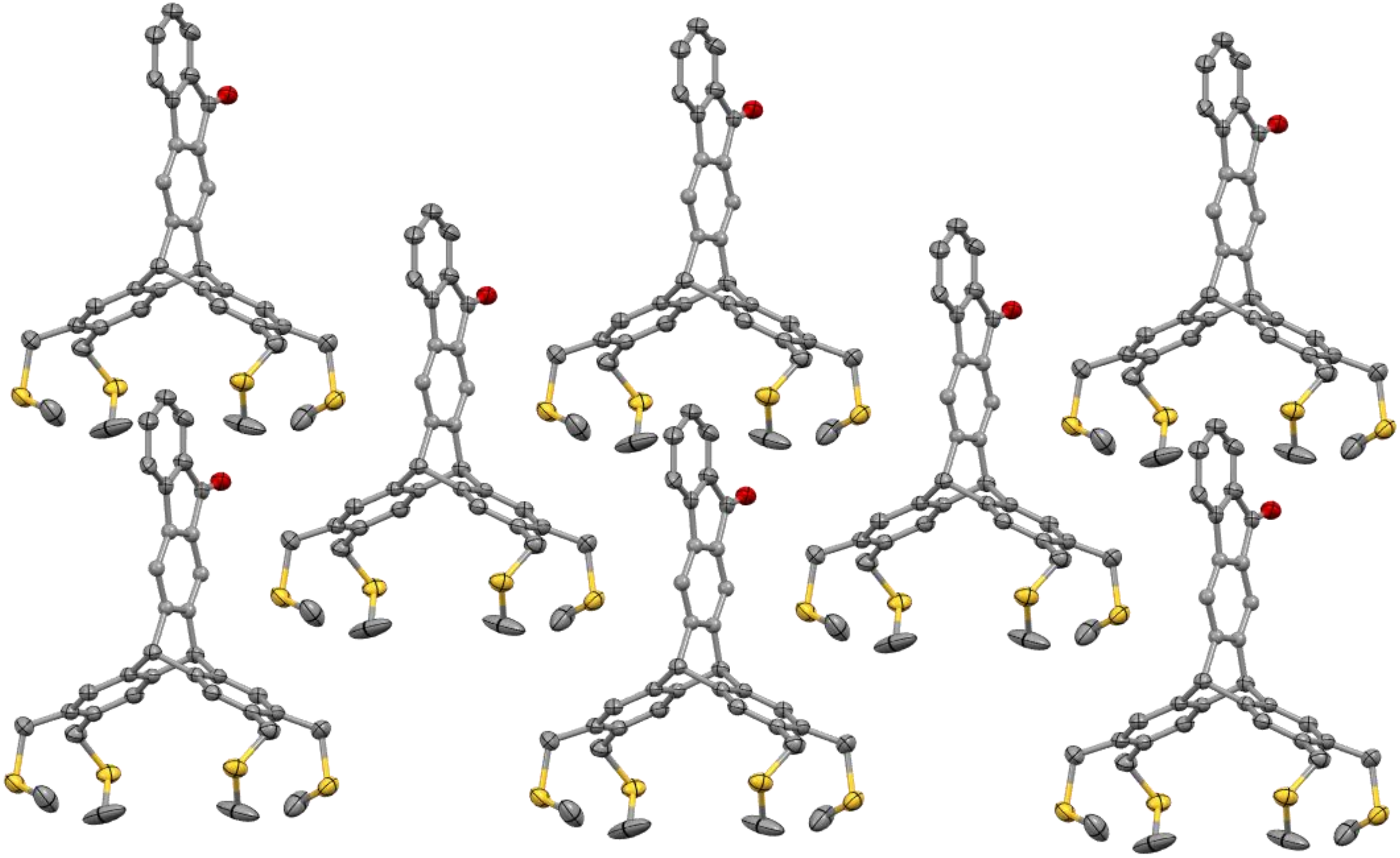

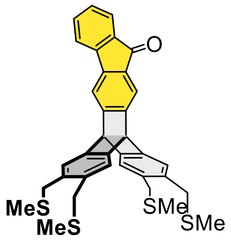

X-ray: Compound **S2** (displacement ellipsoids are shown at the 50% probability level)

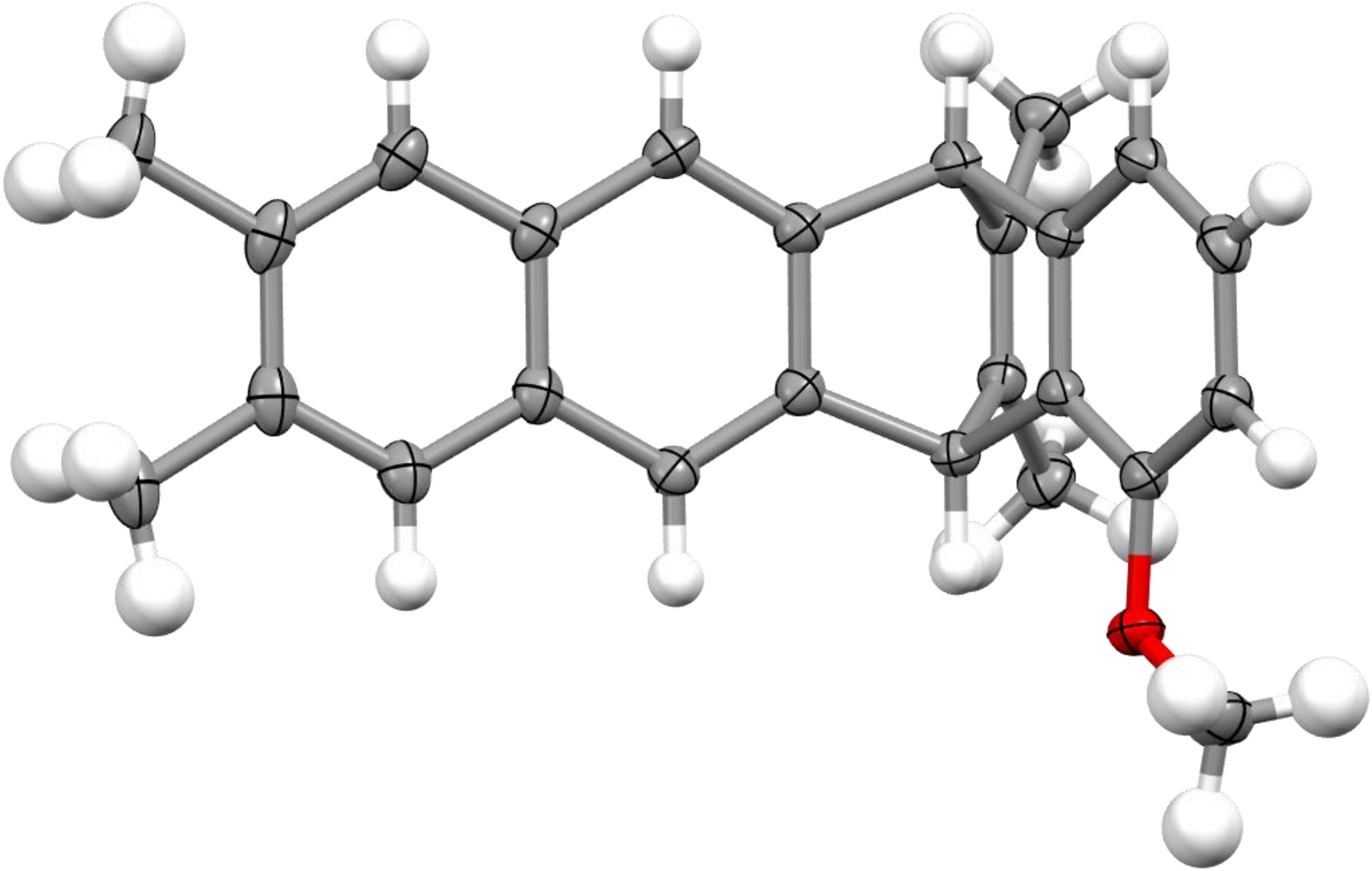

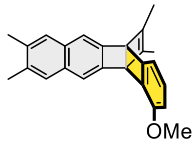

X-ray: Crystal packing of compound **S2** (hydrogen atoms are omitted for clarity)

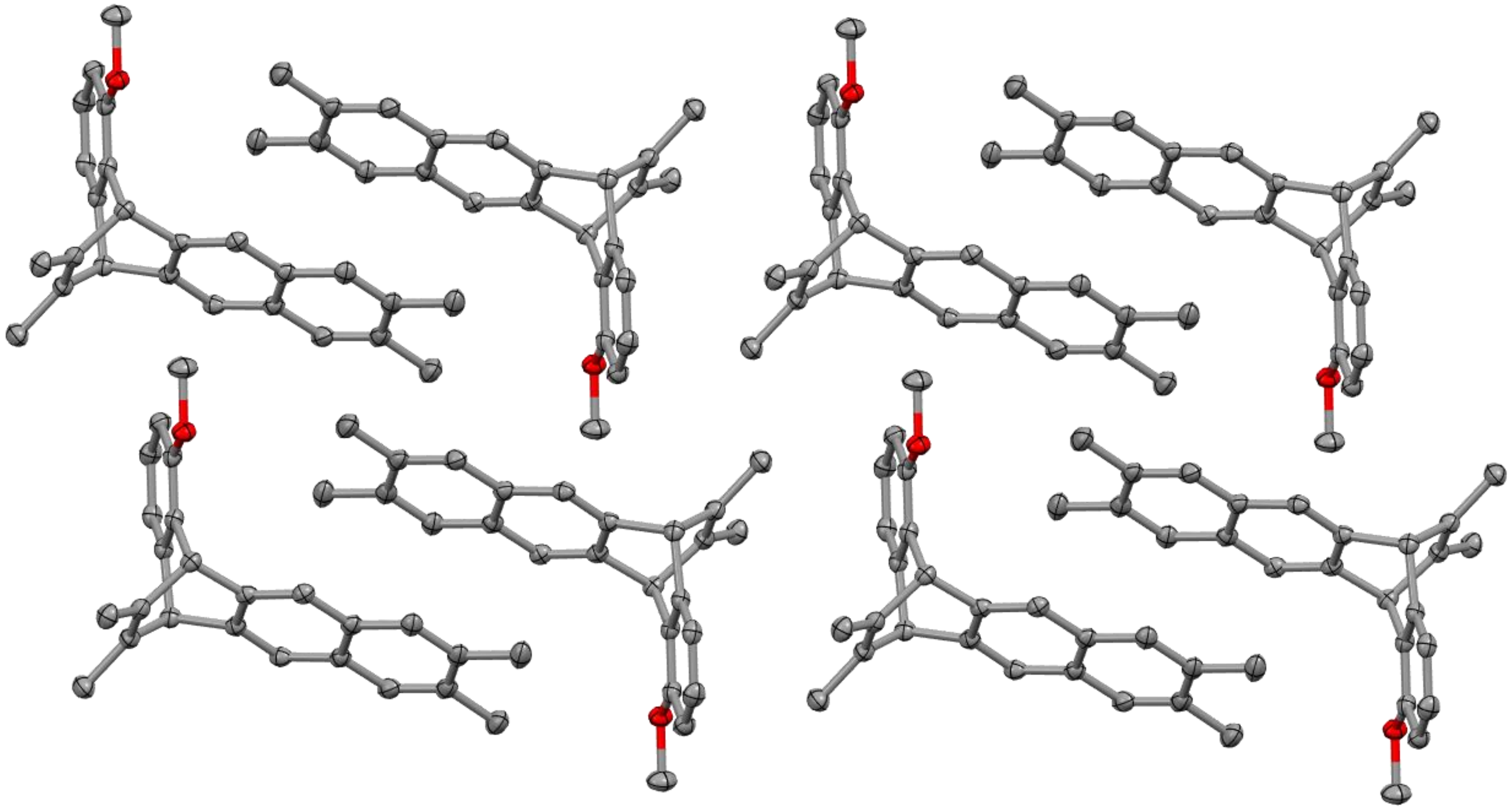

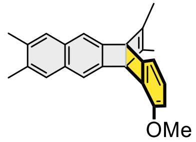

Supplement: Supplementary file 1 — Supporting Information [file ANIE-65-e202513922-s002.pdf]
